# Genomewide identification of genes involved in the potato response to drought indicates functional evolutionary conservation with *Arabidopsis* plants

**DOI:** 10.1111/pbi.12800

**Published:** 2017-08-14

**Authors:** Marcin Pieczynski, Anna Wyrzykowska, Kaja Milanowska, Dominika Boguszewska‐Mankowska, Barbara Zagdanska, Wojciech Karlowski, Artur Jarmolowski, Zofia Szweykowska‐Kulinska

**Affiliations:** ^1^ Department of Gene Expression Faculty of Biology Institute of Molecular Biology and Biotechnology Adam Mickiewicz University Poznan Poland; ^2^ Potato Agronomy Department, Plant Breeding and Acclimatization Institute National Research Institute Division Jadwisin Poland; ^3^ Department of Biochemistry Faculty of Agriculture and Biology Warsaw University of Life Sciences Warsaw Poland; ^4^ Department of Computational Biology Faculty of Biology Institute of Molecular Biology and Biotechnology Adam Mickiewicz University Poznan Poland

**Keywords:** potato drought‐responsive genes, transcriptomics, RNA‐seq, *Arabidopsis* homologues, functional confirmation of the selected gene importance to drought, *Solanum tuberosum*

## Abstract

Potato is one of the four most important food crop plants worldwide and is strongly affected by drought. The following two pairs of potato cultivars, which are related in ancestry but show different drought tolerances, were chosen for comparative gene expression studies: Gwiazda/Oberon and Tajfun/Owacja. Comparative RNA‐seq analyses of gene expression differences in the transcriptomes obtained from drought‐tolerant versus drought‐sensitive plants during water shortage conditions were performed. The 23 top‐ranking genes were selected, 22 of which are described here as novel potato drought‐responsive genes. Moreover, all but one of the potato genes selected have homologues in the *Arabidopsis* genome. Of the seven tested *A. thaliana* mutants with altered expression of the selected homologous genes, compared to the wild‐type *Arabidopsis* plants, six showed an improved tolerance to drought. These genes encode carbohydrate transporter, mitogen‐activated protein kinase kinase kinase 15 (MAPKKK15), serine carboxypeptidase‐like 19 protein (SCPL19), armadillo/beta‐catenin‐like repeat‐containing protein, high‐affinity nitrate transporter 2.7 and nonspecific lipid transfer protein type 2 (nsLPT). The evolutionary conservation of the functions of the selected genes in the plant response to drought confirms the importance of these identified potato genes in the ability of plants to cope with water shortage conditions. Knowledge regarding these gene functions can be used to generate potato cultivars that are resistant to unfavourable conditions. The approach used in this work and the obtained results allowed for the identification of new players in the plant response to drought.

## Introduction

Potato (*Solanum tuberosum* L.) is a crop plant cultivated worldwide that is of great economic importance. There are more than 4500 potato varieties in the World Catalogue of Potato Varieties 2009/10 (www.euroseeds.eu/potatoes). These potato cultivars differ in their bulking time, growth rate and sensitivity to pathogens and various abiotic stresses. Many classical and molecular studies have been performed to identify the genetic loci responsible for agricultural traits using diploid potato plants (Anithakumari *et al*., 2011, 2012; Gebhardt, 2016; Khan *et al*., 2015; Mani and Hannachi, 2015). Recently, due to the development of high‐throughput RNA sequencing techniques, which enable qualitative and quantitative global analyses of gene expression, novel approaches have been proposed to understand many physiological and agronomical traits in crop plants (Chen *et al*., 2015; Gramazio *et al*., 2016; Prince *et al.,* 2015; Shankar *et al*., 2016; Wang *et al*., 2016; Zhang *et al*., 2014a,b). This methodology enables the study of global gene expression in tetraploid plants, such as potato plants. The identification of gene expression profiles in potato cultivars that are closely related but differ in a particular trait appears to be a promising approach for understanding the mechanisms involved in plant stress tolerance regulation.

Drought is one of the main climate threats limiting crop plant production. Plants are sessile organisms that can tolerate and survive even severe drought by developing escape, avoidance and tolerance strategies that are not mutually exclusive and may act synergistically (Blum, 1996; Levitt, 1980 1980). The strategy employed by plants strongly depends on the plant species, phase of plant development, and intensity and duration of the drought progression (Pinheiro and Chaves, 2011). Potato belongs to a group of crop plants that are considered sensitive to water shortage (Hijmans, 2003; Obidiegwu *et al*., 2015). To diminish the effect of the forecasted potato harvest losses, it is crucial to identify the strategies used by potato plants to withstand long drought periods during the vegetative season. Therefore, we decided to analyse transcriptome differences in the following two selected pairs of potato cultivars: Gwiazda/Oberon and Tajfun/Owacja. The cultivars in each pair are closely related to each other but differ in their sensitivity to drought conditions (Nowacki, 2012).

In this study, by comparing closely related cultivars, we identified 23 potato genes with significantly different expression profiles during drought. From the list of homologous *Arabidopsis* genes, we selected seven genes and obtained a homozygous mutant of each gene. The *A. thaliana* mutants with altered expression of six of the seven genes tested showed a significant improvement in tolerance to drought, demonstrating the evolutionary conservation of the functions of the genes selected between potato and *Arabidopsis*. Thus, the approach applied and results obtained allowed us to identify novel important and conserved players in the plant response to drought.

## Results

### Selection of closely related potato cultivars that differ in water shortage tolerance

**Figure 1:**
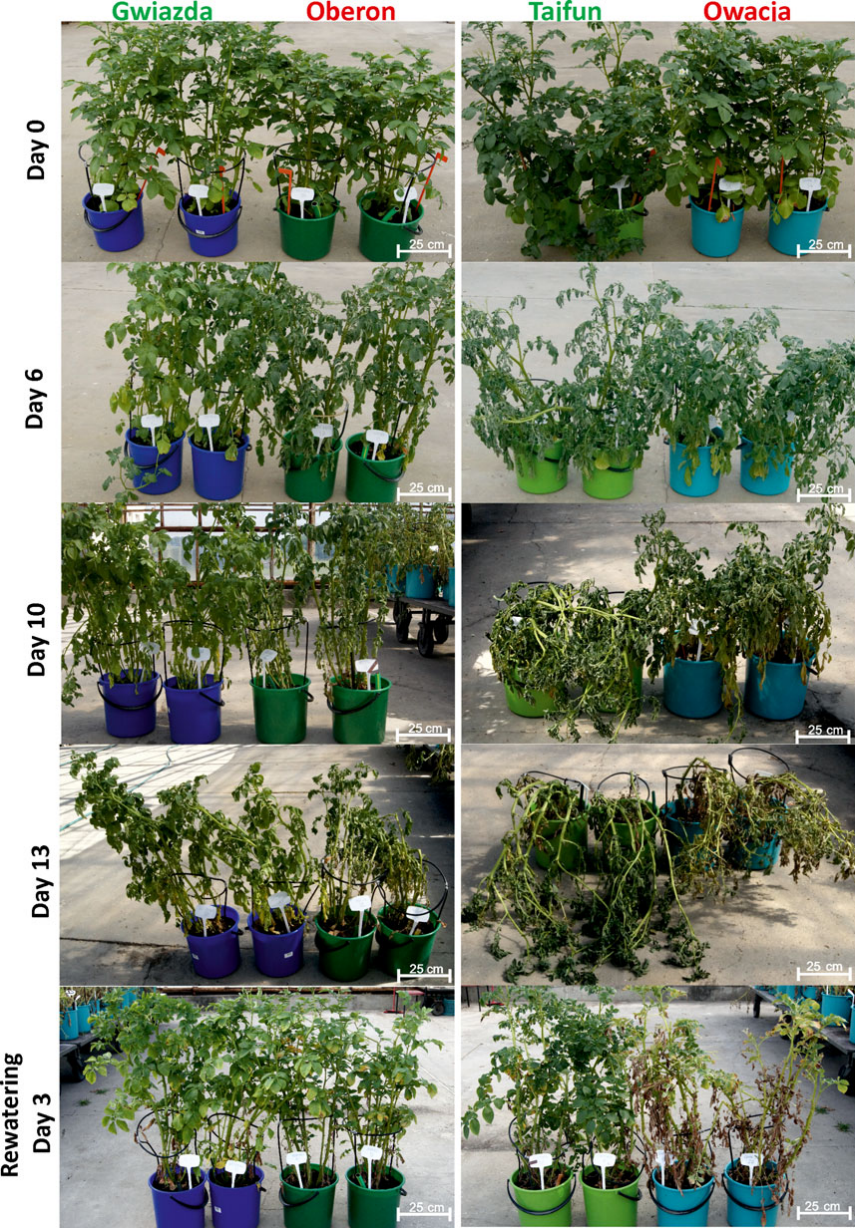
Potato cultivars Gwiazda and Tajfun show an improved tolerance to drought. Two‐month‐old well‐watered plants of the four studied cultivars were subjected to water stress. Plants before the application of water stress (upper panel) and after 6, 10 and 13 days without watering are shown (lower panels). After 13 days of drought, the plants were rewatered. The plants are shown three days after rewatering (the lowest panel).

**Figure 2:**
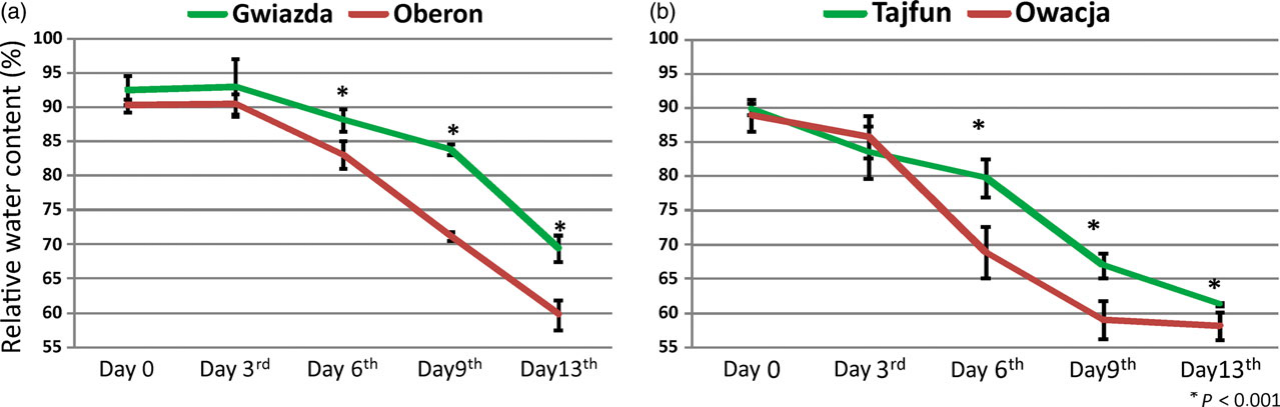
Potato plants from selected cultivars show differences in the RWC in the subsequent days of drought. RWC was measured in leaves from the four studied cultivars after drought stress: (a) cultivars Gwiazda and Oberon and (b) cultivars Tajfun and Owacja. RWC was measured on day 0, 3, 6, 9 and 13 of drought stress. The measurements taken at various drought time points are presented as a percentage of the RWC of the plant at day 0. The leaves were detached and weighed for the initial FW (fresh weight), SW (saturated weight) and DW (dry weight) values. Calculations were performed as described in the Experimental Procedures section, and values are shown as mean ± SD (*n* = 3) of three independent experiments. *P < 0.001, Mann–Whitney *U*‐test.

Previous studies on various potato cultivars led us to select four cultivars: Gwiazda, Tajfun, Oberon and Owacja, which differ in their tolerance to drought (Nowacki, 2012). In addition, they can be grouped as pairs of cultivars based on their origins as follows: Gwiazda and Oberon have one parent in common and, thus, a similar genetic background, while Tajfun and Owacja originated from the clone PS646, which is a parent of the Tajfun cultivar and a grandparent of the Owacja cultivar (Figure S1 and Data S1). The potato cultivars within each pair differ in their ability to withstand drought conditions. Figure 1 (also Figure S2) shows a comparison of the growth of the Gwiazda/Oberon and Tajfun/Owacja cultivar plants under 13 days of drought conditions and after rewatering. As expected, Gwiazda and Tajfun withstood drought better than Oberon and Owacja. A strong wilting phenotype was observed in the Oberon and Owacja cultivars by the sixth day of drought, while on the 13th day of the experiment, all cultivars suffered from the water scarcity. On the 10th day without watering, the lower leaves of the Owacja plants began to yellow intensely, while on day 13, approximately half of the Owacja leaves were yellow or dry. This finding was not observed in the plants of the other three cultivars. Rewatering allowed the plants from the three cultivars to recover completely; however, the Owacja plants were unable to fully regenerate because some of the leaves were irreversibly damaged by the drought stress (Figure 1). After 6, 9 and 13 days of drought stress, the transpirational water loss in the drought‐tolerant cultivars (Gwiazda and Tajfun) was significantly lower than that observed in the drought‐sensitive cultivars (Oberon and Owacja) (Figure 2). The higher RWC levels in the Gwiazda and Tajfun plants indicate that their drought tolerance is superior to that of the Oberon and Owacja plants. Gwiazda developed better mechanisms to reduce transpirational water loss than Tajfun. This finding is also supported by the phenotypic observations in the Gwiazda and Tajfun plants under the drought conditions (Figure 1; the 6th, 10th and 13th days of drought stress).

### Establishing water deficit conditions: Molecular monitoring during the drought experiment

**Figure 3:**
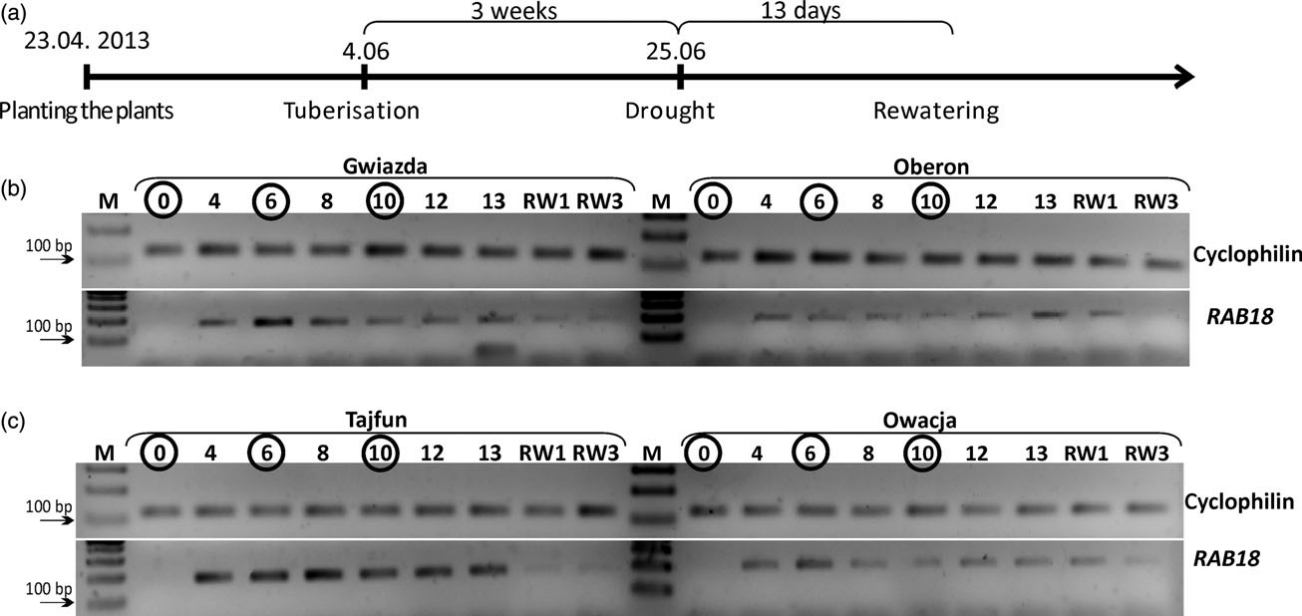
The scheme and molecular marker monitoring of the drought experiment. (a) Time course of the drought experiment. Drought was applied three weeks after the initiation of the tuberization process and was carried out for 13 days. On the 13th day, the plants were rewatered. (b and c) Expression profile of the *RAB18* gene. Gel electrophoresis of *RAB18* (PGSC0003DMG400003531) and cyclophilin (PGSC0003DMG400001630) cDNA RT‐PCR products on selected days of drought is shown. The results of the cultivar pairs Gwiazda/Oberon and Tajfun/Owacja are shown in panels b and c, respectively. The numbers above the gels indicate the subsequent days of drought; the numbers in circles show the time points when the plant RNA was isolated and subjected to the RNA‐seq analysis. RW—days after plants were rewatered; M—DNA molecular weight marker.

The main aim of these studies was to identify new genes that are involved in the potato plant response to drought. Therefore, we searched for differentially expressed genes during a drought period in potato plants representing closely related pairs of cultivars that differ in their sensitivity to water shortage. To perform this analysis, plants of the four cultivars studied were subjected to drought three weeks after tuberization began. At this stage of development, potato plants are extremely sensitive to a lack of water (Głuska, 2004). The plants were subjected to drought, and their leaves were collected before the experiment and on the 4th, 6th, 8th, 10th, 12th and 13th days of the experiment (Figure 3a).

The induction of *RAB18* gene expression (*Responsive to ABA 18*; *PGSC0003DMG400003531*) at the mRNA level has been shown to occur in plants exposed to various abiotic stresses or exogenous ABA treatment (Hong *et al*., 2008; Lang and Palva, 1992). *RAB18* expression is strongly induced in potato leaves upon drought and dehydration stress (Pieczynski *et al*., 2013). We performed a semiquantitative RT‐PCR analysis to determine the *RAB18* gene expression during our drought experiment using RNA isolated from potato leaves derived from plants representing the four cultivars studied (Figure 3b and c). *RAB18* mRNA accumulation was detectable starting on the fourth day of water deficit in all cultivars studied. Generally, *RAB18* mRNA accumulated in the drought‐tolerant cultivars to a much higher extent than that in the drought‐sensitive cultivars (Figure 3b and c). In the Gwiazda plants, the highest level of *RAB18* expression was observed on the sixth day of drought. During the subsequent days of water deficit, the level of *RAB18* mRNA in the Gwiazda plants continuously decreased (Figure 3b). However, in the Oberon cultivar, the level of *RAB18* accumulation was relatively stable during all days of drought (Figure 3b). In the Tajfun plants, *RAB18* showed the highest expression level on days 6 and 8 and then slightly decreased and remained at the same level until the end of the drought experiment (Figure 3c). The Owacja plants exhibited the highest *RAB18* mRNA expression level on the sixth day, and then, the *RAB18* expression remained relatively stable until the last day of our drought experiment (Figure 3c).

On the first and third days after the plants were rewatered (Figure 3b and c, RW1 and RW3), a strong reduction in the *RAB18* mRNA level was observed in the drought‐tolerant cultivars (Gwiazda and Tajfun), while in the drought‐sensitive cultivars (Oberon and Owacja), the down‐regulation of *RAB18* mRNA was slower and was observed on the third day after rewatering (RW3). RT‐PCR experiments were carried out using two additional biological replicates on days 0, 6 and 10, and the results were the same as those obtained in the RT‐PCR experiment using the first biological replicate. Moreover, RT‐qPCR was performed in all time points (days 0, 4, 8, 10, 12, 13, RW1 and RW3) to confirm the semiquantitative RT‐PCR results obtained in the Gwiazda/Oberon cultivars (Figure S3). This RT‐qPCR experiment was performed using one biological replicate with three technical repeats. The results mirrored those obtained using RT‐PCR (Figures 3 and S3). The analysis of the *RAB18* mRNA level during the drought experiment allowed us to select the days of drought stress to be used for the RNA‐seq. We performed the RNA‐seq on days 0 (no *RAB18* mRNA; control conditions), 6 (the maximum level of *RAB18* mRNA expression; strong plant response to drought) and 10 (weaker *RAB18* mRNA expression; plant response to extended drought).

### Identification of differentially expressed genes during drought stress in the Gwiazda/Oberon and Tajfun/Owacja cultivars

Total RNA was isolated from plants leaves before being subjected to the drought conditions and on the 6th and 10th days of the experiment. Differential analyses of the whole transcriptome sequencing data obtained were carried out by comparing the expression levels of the same transcripts in the Gwiazda/Oberon and Tajfun/Owacja pairs. Normalized reads for all splicing isoforms found for a particular transcript were summed and treated as the final expression of a particular gene as described in detail in the Experimental Procedures section.

Table S2 presents the number of clean and mapped reads obtained for all RNA libraries sequenced, the distribution and percentage of RNA‐seq reads mapped onto genomic loci, and the number and percentage of reads mapped onto multiple loci for the two pairs of drought‐tolerant and drought‐sensitive cultivars under control (day 0) and drought conditions (days 6 and 10). After the sequencing and removing the low‐quality reads, the total number of reads that varied between the samples ranged from 39 to 43 million. The obtained reads were mapped onto the reference *S. tuberosum* group Phureja potato genome. Depending on the RNA library, the percentage of reads that mapped onto the potato genome varied from 67.9 to 83.8. The percentage of reads that mapped onto a single position within the genome varied from 80.2 to 94.8, while the percentage of reads that mapped onto more than one locus varied from 5.2 to 19.8.

To select the differentially expressed genes upon drought conditions in both pairs of drought‐tolerant/drought‐sensitive cultivars, we designed a pipeline that allowed us to limit the number of genes selected to the best candidate genes. First, of the differentially expressed genes in the pairs of cultivars studied, only those that exhibited the same level of expression on day 0 in either the Gwiazda/Oberon or Tajfun/Owacja cultivars (the differences in the expression of these genes were statistically insignificant) were selected for further study (Figure S4A, B; horizontal two‐headed arrows on day 0). Then, from this selected starting pool of genes, only the genes that displayed statistically significant differences in their expression pattern between days 0 and 6 or days 0 and 10, independently in each drought‐tolerant cultivar (Gwiazda or Tajfun) (Figure S4A, B; vertical arrows), passed the filtering step. In parallel, the selected genes had to display statistically significant differences between the drought‐tolerant and drought‐sensitive cultivars on any given day of drought (Figure S4A, B; horizontal two‐headed arrows on days 6 and 10). These analyses resulted in the selection of genes that significantly differed in their expression levels under drought conditions. In the Gwiazda/Oberon comparison, 134 genes were selected (66 genes were up‐regulated, and 68 genes were down‐regulated in the Gwiazda versus Oberon cultivars), and in the Tajfun/Owacja comparison, 460 genes were selected (261 genes were up‐regulated, and 199 genes were down‐regulated in the Tajfun versus Owacja cultivars) (Figure S5A).

We also selected genes that exhibited a low fold change (0.8 < fold change < 1.2) in their expression patterns during the time course of our drought experiment in plants representing all four cultivars (Figure S4C). We identified eight genes with stable expression under the control and drought conditions. These genes represent a pool of genes that can potentially serve as gene expression controls in drought experiments. The stable expression of these genes was confirmed by RT‐PCR (Figure S6).

The genes that showed statistically significant changes in their expression levels in the drought‐tolerant versus drought‐sensitive cultivars were subjected to further selection steps. The second round of filtering consisted of the selection of those genes showing the highest differences in their expression profiles obtained in the RNA‐seq data during the time course of the drought experiment (days 6 and 10). The comparison of the normalized number of reads for transcripts derived from all genes identified in the first round of selection (594 genes) during the time course of the drought experiment is presented in Table S3. In addition to this tabular data presentation, the expression profile of each gene is shown in a separate chart containing the normalized number of reads from the RNA‐seq data for the Gwiazda/Oberon or Tajfun/Owacja pair of cultivars (Tables S1, S3).

Of the 33 drought‐related genes obtained in second round of selection for Gwiazda/Oberon, the expression profiles of 18 genes were up‐regulated, while the expression profiles of 15 genes were down‐regulated in the comparison of the Gwiazda and Oberon cultivars (Figure S4A). In the Tajfun/Owacja cultivars (53 selected genes in second round), the expression profiles of 28 genes were up‐regulated, and 25 genes were down‐regulated when the Tajfun cultivar was compared to the Owacja cultivar (Figure S5A). All genes from the second round of selection were further assessed by confirming the gene expression profile differences in each gene in the drought‐tolerant versus drought‐sensitive cultivars using RT‐PCR (third round of selection). Only genes showing consistency in their expression profiles between the RNA‐seq and RT‐PCR results were used for further analysis.

**Figure 4:**
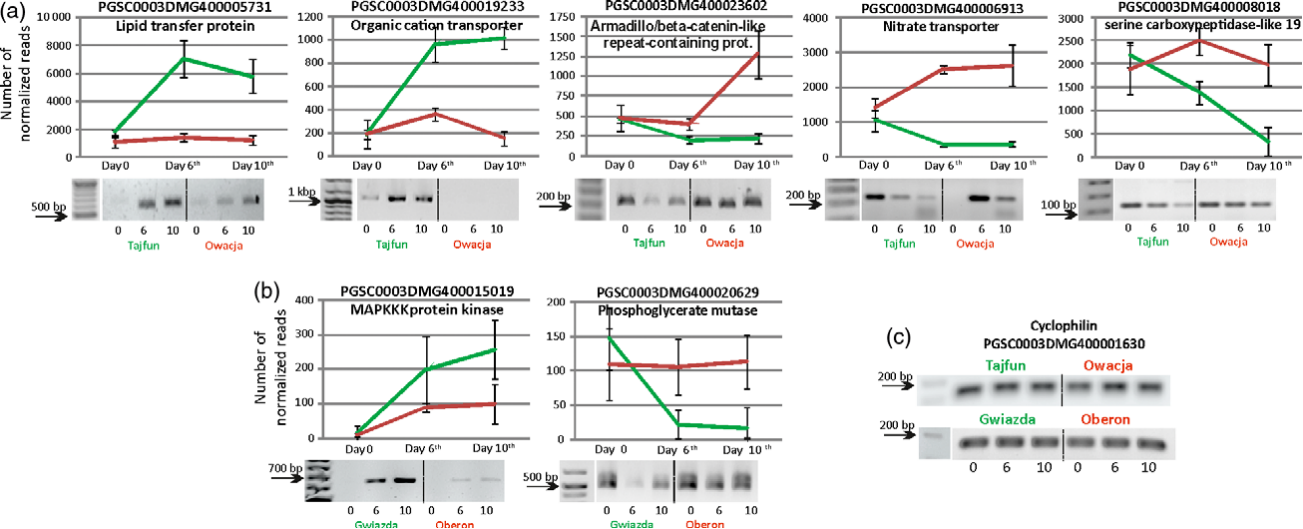
Seven selected potato genes showing the highest differences in gene expression between the studied cultivars during the drought experiment. Above each graph, the accession number of a given gene and its function are displayed. (a) Graphs showing the normalized number of reads for genes from Tajfun/Owacja cultivars (green/red lines) and (b) Gwiazda/Oberon cultivars (green/red lines). Values are shown as mean ± SD (*n* = 3), *P* < 0.05, of three independent RNA‐seq experiments. Statistical methods for the differential gene expression analysis are described in the Experimental Procedures section. The homologues of these genes were further studied in *Arabidopsis* plants. Gel electrophoresis analyses of the RT‐PCR products for all seven genes are presented below each graph, confirming the RNA‐seq results. (c) Gel electrophoresis of the stably expressed cyclophilin gene during the drought experiment in all cultivars studied. The numbers at the bottom of each gel indicate the days of the drought experiment.

The third selection stage allowed us to reduce the number of selected genes to the nine top‐ranking genes in the Gwiazda/Oberon pair and 15 genes in the Tajfun/Owacja pair (Figure S5A). The expression profiles of six genes were up‐regulated, while the expression profiles of three genes were down‐regulated when the Gwiazda plants were compared to the Oberon plants, and the expression of nine genes was up‐regulated, while the expression of six genes was down‐regulated when the Tajfun plants were compared to the Owacja plants (Figure S5A). Notably, one of the selected genes (*PGSC0003DMG400014293*) was up‐regulated by drought in both drought‐tolerant cultivars (Gwiazda and Tajfun), and thus, the overall number of selected genes was 23 (the accession numbers of these genes are presented in Fig. S5B). The results of the third round of selection are shown in Figure S7. RT‐PCRs were carried out to test the expression profiles of the 23 selected genes in three biological replicates. The RNA‐seq data and RT‐PCR results of seven potato genes (two in Gwiazda/Oberon and five in Tajfun/Owacja) displaying the highest differences in gene expression during the water shortage conditions in the drought‐tolerant and drought‐sensitive cultivars are shown in Figure 4, and the expression data of the remaining 17 genes are shown in Figure S7.

### *A. thaliana* genes homologous to the selected drought‐related potato genes also affect the *Arabidopsis* response to water deficit conditions

**Figure 5:**
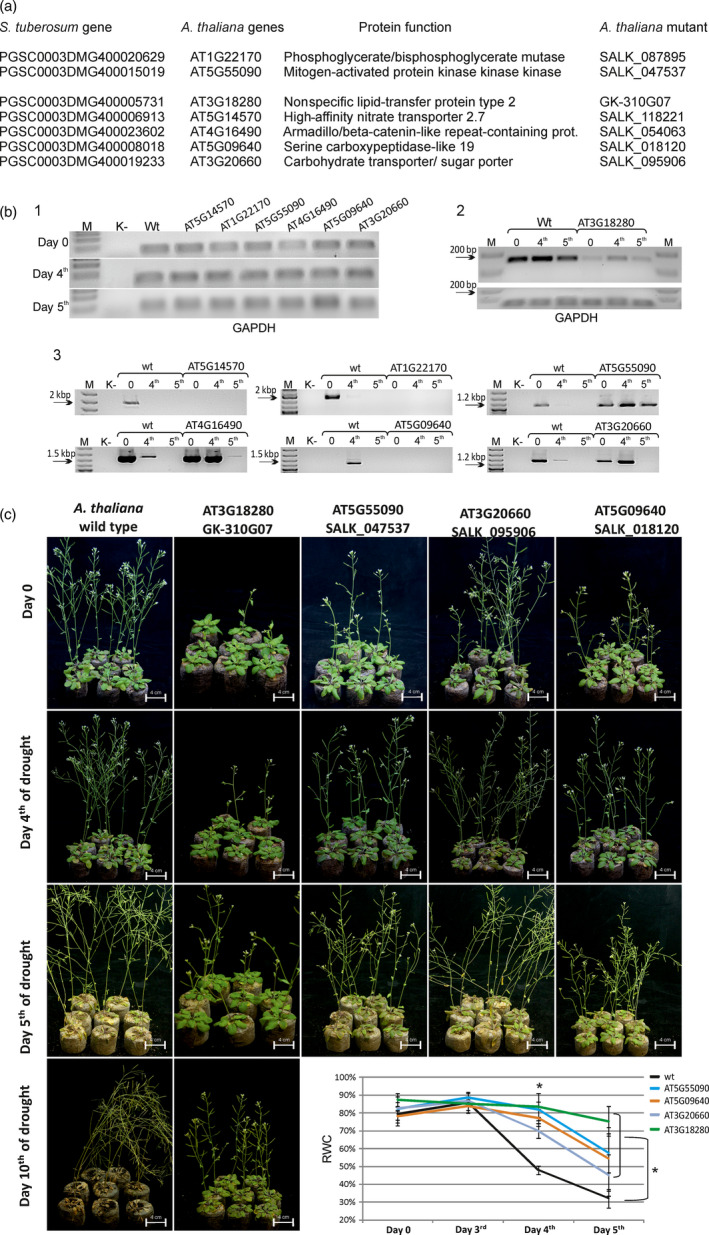
Analysis of *Arabidopsis* mutant plants with altered expression of genes homologous to the selected drought‐related potato genes. (a) Accession numbers of the seven top‐ranking drought‐related potato genes (left column) and the accession numbers and protein functions of their *Arabidopsis* homologues (middle columns) were obtained from the Spud DB and TAIR database (Hirsch *et al*., 2014; Huala *et al*., 2001). The right column presents the accession numbers of the selected *Arabidopsis* SALK or GABI‐Kat mutants of each gene (Alonso *et al*., 2003; Kleinboelting *et al*., 2012). (b) RT‐PCR analysis of the expression of selected *Arabidopsis* genes under control (day 0) and drought conditions (days 4 and 5). (1) Gel electrophoresis of the RT‐PCR products of the *GAPDH* gene in wild‐type and selected *Arabidopsis* SALK line plants on days 0, 4 and 5 of the drought experiment; (2 and 3) gel electrophoresis of the RT‐PCR products of selected *Arabidopsis* genes in wild‐type and mutant plants grown under control and drought conditions, respectively. SALK accession numbers are presented above each electrophoretic line. M—DNA marker; K—RT‐PCR nontemplate control; wt—wild‐type plant. (c) *Arabidopsis* plants subjected to water deficit stress. Plants are shown on days 0, 4, 5 and 10 of the drought experiment. Only wild‐type and four mutant plants are shown (the other three are presented in Fig. S8). Lower bottom right panel of (c)—RWC analysis in the leaves of wild‐type and selected mutant *Arabidopsis* plants. Mutant plants show higher water content than wild‐type plants. RWC was measured 0, 4 and 5 days after the introduction of drought stress. Values are shown as mean ± SD (*n* = 3) of three independent experiments. *P < 0.001, Mann–Whitney *U*‐test.

Twenty‐two *A. thaliana* genes that are homologous to the best potato drought‐related candidate genes were identified (one potato gene—*PGSC0003DMG400024093*—has no homologue in the *Arabidopsis* genome; Figure 5a). Of these 22 genes, seven arbitrarily chosen *Arabidopsis* homozygous mutants were obtained from the SALK and GABI‐Kat mutant collections. These mutants were genotyped to confirm that they were homozygous lines (Alonso *et al*., 2003 **;** Kleinboelting *et al*., 2012). The RT‐PCR analyses revealed that in the case of three genes, T‐DNA insertions resulted in the complete abolishment of gene expression (*AT5G14570*, A*T1G22170* and *AT5G09640*). A down‐regulation of gene expression was observed only in one mutant (*AT3G18280*), and in the case of the other three genes, T‐DNA insertions resulted in the up‐regulation of the expression of each gene analysed (*AT3G20660*, *AT4G16490* and *AT5G55090*) (Figure 5b).

Four‐week‐old *Arabidopsis* wild‐type and mutant plants were subjected to drought, which was continued until plant death (up to 10 days). Comparative RT‐PCR analyses of the gene expression in wild‐type and mutant plants under drought conditions showed that in the null mutants analysed, there was no expression of the genes tested (*AT5G14570*, A*T1G22170* and *AT5G09640*), although the expression was detectable in the wild‐type plants (Figure 5b). Interestingly, the expression in the control wild‐type plants was detectable only on day 0 in the case of two genes (*AT5G14570* and *AT1G22170*), but during drought stress, the expression was abolished, while in the case of the *AT5G09640* gene, its expression was observed only on day 4 of drought stress. In the case of the *AT3G18280* gene, we observed a slight up‐regulation of its expression in the wild‐type plants upon drought, and in the SALK mutant of this gene, the expression was also up‐regulated, although at a much lower level than that in the wild‐type plants (Figure 5B). The expression of the *AT3G20660*, *AT4G16490* and *AT5G55090* genes was strongly down‐regulated by drought in the wild‐type plants, while in the mutant plants, up‐regulated gene expression was observed, although for the *AT4G16490* and *AT3G20660* genes, there was no detectable expression on the fifth day of drought (Figure 5B). Compared with the wild‐type plants, all mutant plants, except for one (*AT1G22170*), showed an improved tolerance to drought starting on day 4 of drought; additionally, the wild‐type plants presented a strong wilting phenotype on the same day (Figures 5C and S8B). In almost all mutant plants, the wilting phenotype was visible on the fifth day of the stress conditions; however, in the case of the *AT3G18280* gene mutant, the plants still showed a strong resistance to the lack of water on the 10th day of the stress experiment.

The best drought‐tolerant *Arabidopsis* mutant plants are shown in Figure 5C, while the remaining three mutants are presented in (Figure S8). The drought‐tolerant phenotypic observations of the *Arabidopsis* mutants were confirmed by the RWC measurements. Figure 5C (the bottom right panel) and Figure S8C indicate a strong transpirational water loss reduction in six of the seven *Arabidopsis* mutants tested compared to that in the wild‐type *Arabidopsis* plants. The *AT1G22170* mutant exhibited higher RWC values than the wild‐type plants on the fourth day of drought; however, on the fifth day of drought stress, the leaf RWC in the mutant was the same as that in the wild‐type plants.

These experiments demonstrated that the selected genes exhibit evolutionary conservation in their functions in the plant response to drought, at least in potato and *Arabidopsis* plants.

## Discussion

### Comparative transcriptomic studies identified novel genes involved in the potato response to drought

Next‐generation sequencing (NGS) of transcriptomes provides a useful tool for exploring differential gene expression between closely related plant species, cultivars or even individuals to identify genes that are responsive to different environmental stresses (Prince *et al.,* 2015; Chen *et al*., 2015; Wang *et al*., 2016; Gramazio *et al*., 2016; Shankar *et al*., 2016). The studies presented herein provide in‐depth insight into the responses of potato plants of closely related cultivars with contrasting tolerances to drought stress during tuberization. A large number of genes were found to be differentially expressed in the cultivars studied. This number was higher in the Tajfun/Owacja pair than in the Gwiazda/Oberon pair. We cannot exclude the possibility that this difference is due to the more distant relationship between Tajfun and Owacja than that between the Gwiazda and Oberon cultivars, resulting in a higher variability of allele content (see Figure S1).

We limited the number of compared genes to those related to water shortage conditions by selecting only those genes that displayed a similar level of expression on day 0 and for which the induction or down‐regulation of gene expression was observed in the drought‐tolerant cultivar compared to the level in the drought‐sensitive cultivar within a given pair on days 6 and 10. This selection process allowed us to avoid any ambiguities in whether the differential gene expression was caused by reasons other than drought. Using this strategy, we identified the 23 top‐ranking drought‐related genes that successfully passed the designed filtering pipeline. One gene was found to be selected in both pairs of analysed cultivars (*PGSC0003DMG400014293*); this gene encodes the CAP160 protein, which is already known to be stress‐related. Interestingly, only this single gene of the 23 identified here was also found by the Wang group (Zhang *et al*., 2014a,2014b,c) in their transcriptome‐wide studies on drought‐responsive genes in the drought‐tolerant potato cultivar Longshu3 (Cheng *et al*., 2013; Zhang *et al*., 2014a,2014b,c). Their results on the *CAP160* gene are consistent with our data showing a strong up‐regulation of this gene in drought‐tolerant cultivars under water deficit conditions. Although we also found that the potato *CAP160* mRNA level was up‐regulated when water was scarce in the drought‐sensitive cultivars, the induction of *CAP160* expression was much lower in the drought‐sensitive cultivars than in the drought‐tolerant cultivars (see Figure S7). Interestingly, *CAP160* expression is also up‐regulated in spinach and pepper by both water and low‐temperature stresses (Kaye *et al*., 1998; Park *et al*., 2016). These results suggest that the up‐regulation of *CAP160* expression during water deficit in drought‐tolerant potato cultivars may be related to the improved response of these cultivars to drought stress.

### Transcriptomic analyses show that the identified potato genes were also related to drought in other plant species

We compared the expression patterns of the 23 selected potato genes with the expression of their homologues in *Arabidopsis* and rice under drought conditions using available high‐throughput data (Table 1). Surprisingly, the analysis of these data revealed that 17 of the genes show expression changes in either all or two of the species compared. Six selected genes show the same direction of expression change in potato, *Arabidopsis* and rice; nine genes show the same direction of expression change only in rice and potato; and one gene shows similar expression changes in potato and *Arabidopsis*. Two genes show changes that are opposite to the potato homologue expression: one in rice and one in *Arabidopsis* (Table 1; Winter *et al*., 2007; Kapushesky *et al*., 2012; Petryszak *et al*., 2016; Moumeni *et al*., 2015; Petryszak *et al.,* 2016; Zielezinski *et al*., 2017). To the best of our knowledge, the identified common drought‐responsive genes have not been studied in rice, *Arabidopsis* or potato regarding their roles in the plant response to a water deficit.

**Table 1:** Comparison of the 23 top‐ranking drought‐related genes in *S. tuberosum* and their homologues in *Arabidopsis thaliana* and *Oryza sativa* and the direction of the expression change during drought stress

No.	Names of the cultivars	Potato gene	*Arabidopsis* gene	Rice gene	Common drought‐related genes	
					Potato vs. *Arabidopsis*	Potato vs. rice
1	Gwiazda/Oberon	PGSC0003DMG400001621	AT4G38690	LOC_Os09g36520	↗	↗
2		PGSC0003DMG400012174	AT1G64110	LOC_Os01g12660	–	↗
3		PGSC0003DMG400014293*	AT5G52300	LOC_Os10g36180	–	↗
4		PGSC0003DMG400015019	AT5G55090	LOC_Os02g21700	–	nd
5		PGSC0003DMG400024849	AT1G77120	LOC_Os11g10480	–	↗
6		PGSC0003DMG401015935	AT4G33150	LOC_Os02g54254	–	↗
7		PGSC0003DMG400000723	AT4G34490	LOC_Os03g51250	–	nd
8		PGSC0003DMG400002943	AT3G11170	No homologue	–	nd
9		PGSC0003DMG400020629	AT1G22170	LOC_Os02g51590	–	nd
10	Tajfun/Owacja	PGSC0003DMG400001771	AT3G09720	LOC_Os07g45360	–	≠
11		PGSC0003DMG400003688	AT1G19530	LOC_Os03g61150	↗	↗
12		PGSC0003DMG400005731	AT3G18280	LOC_Os05g47700	↗	↗
13		PGSC0003DMG400005917	AT3G49400	LOC_Os01g50690	↗	nd
14		PGSC0003DMG400008497	AT3G51810	LOC_Os05g28210	–	↗
15		PGSC0003DMG400019233	AT3G20660	LOC_Os04g53930	↗	↗
16		PGSC0003DMG400024093	No homologue	LOC_Os02g33820	nd	↗
17		PGSC0003DMG400039484	AT1G59860	LOC_Os03g16030	–	↗
18		PGSC0003DMG400006913	AT5G14570	LOC_Os01g50820	–	↘
19		PGSC0003DMG400007427	AT3G05410	LOC_Os01g70820	↘	↘
20		PGSC0003DMG400008018	AT5G09640	LOC_Os11g27264	–	nd
21		PGSC0003DMG400014954	AT4G31940	No homologue	–	nd
22		PGSC0003DMG400022225	AT4G27030	LOC_Os08g08850	↘	↘
23		PGSC0003DMG400023602	AT4G16490	LOC_Os07g39590	≠	↘

Homologues were identified using Orcan software in the ComBio platform for *Arabidopsis* and the UniProt database for rice (http://bar.utoronto.ca/efp2/Arabidopsis/Arabidopsis_eFPBrowser2.html; http://www.combio.pl/orcan; http://www.ebi.ac.uk/gxa/home), with the potato protein as a blastp query (The UniProt Consortium, 2017; Zielezinski *et al*., 2017). *—transcripts found to be affected in both pairs of potato cultivars; ↗—genes up‐regulated in the three compared plant species; ↘—genes down‐regulated in the three compared plant species; nd—no data available;–—no significant changes in *Arabidopsis*; ≠—different directions of change in the compared plant species.

Altogether, 70% of the selected potato genes show evolutionary conservation with rice genes, and 35% of the selected potato genes show evolutionary conservation with *Arabidopsis* genes. This finding suggests that the identified genes play a pivotal role in shaping the plant cell metabolism during the drought stress response.

The GO database annotation in the biological processes category shows that among the 22 top‐ranking *Arabidopsis* homologues, eight are linked to various abiotic stresses (Table S4). Five homologues are associated with water deprivation, heat stress, salt stress, osmotic stress and response to ABA (*AT5G52300*, *AT1G77120*, *AT3G51810*, *AT3G20660* and *AT1G59860*). Drought stress is accompanied by salinity, by osmotic stresses and often by heat in natural conditions of plant growth. Plant metabolic pathways responding to different environmental cues often overlap (Barciszewska‐Pacak *et al*., 2015; Xiong *et al*., 1999). Consequently, genes with induced expression under these stresses were selected in the potato cultivars in the drought experiment performed here (Krasensky and Jonak, 2012; Zhang *et al*., 2014a,2014b,c). ABA signalling is one of the main pathways that integrate various stress signals and control downstream stress responses (Tuteja, 2007). Therefore, it is not surprising that three of the 23 genes analysed were found to be responsive to ABA or even participate in ABA‐activated signalling pathways (*AT1G77120*, *AT5G52300* and *AT3G51810*).

Four of the 23 selected genes were involved in oxidation–reduction processes (*AT1G77120*, *AT3G11170*, *AT4G31940* and *AT4G27030*), and it might be connected with accumulation of reactive oxygen species (ROS) that play a central role in coordinating defence signalling in plants in response to many abiotic stresses (Iyer *et al*., 2012; Mittler, 2002). Cell membrane composition changes, such as changes in phospholipids and proteins, help to maintain membrane integrity, preserve cell compartmentalization and activate phospholipid signalling pathways in response to drought (Jarzyniak and Jasinski, 2014; Liu *et al*., 2013; Ruelland *et al*., 2014; Schulz, 2011). Five of the 23 genes studied are involved in unsaturated fatty acid and fatty acid biosynthetic processes and transmembrane and lipid transport (*AT3G11170*, *AT3G18280*, *AT3G20660*, *AT5G14570* and *AT4G27030*).

### Functional analysis of selected potato homologous genes in *Arabidopsis* confirms their importance in plant tolerance to drought

Six of the seven studied *Arabidopsis* mutants exhibited an improved tolerance to drought compared to the wild‐type *Arabidopsis* plants that were grown under the same conditions. The only mutant that did not show an improved response to drought in *Arabidopsis* plants contained a T‐DNA insertion within the phosphoglycerate/bisphosphoglycerate mutase gene (*AT1G22170*). For five of the homologous genes (*AT3G20660*/*PGSC0003DMG400019233*, *AT5G09640*/*PGSC0003DMG400008018*, *AT5G55090*/*PGSC0003DMG400015019, AT4G16490*/*PGSC0003DMG400023602* and *AT5G14570*/*PGSC0003DMG400006913*), the gene expression changes in the drought‐tolerant cultivar were in the same direction as those in the *Arabidopsis* mutant plants tested. These genes encode carbohydrate transporter, mitogen‐activated protein kinase kinase kinase 15 (MAPKKK15), serine carboxypeptidase‐like 19 protein (SCPL19), armadillo/beta‐catenin‐like repeat‐containing protein and high‐affinity nitrate transporter 2.7 (transmembrane transport, nitrate assimilation/transport), and three of these genes have already been reported in high‐throughput studies as genes responsive to drought in other plants (carbohydrate transporter, MAPKKK15 and NRT2.7).

Carbohydrate transporters are reduced in maize under drought stress, leading to a decreased carbohydrate supply in developing reproductive plant parts. Moreover, the phosphorylation level of the maize carbohydrate transporter/sugar porter transporter (B6U8S7) was up‐regulated under heat stress (Aslam *et al*., 2015; Hu *et al*., 2015).

Mitogen‐activated protein kinases (MAPKs) are involved in signal transduction. The functions of many of these kinases in *Arabidopsis* are unknown. The MAPKKKs form the largest family of MAP kinases. Very little is known about MAPKKK15. However, MAPKKK15 has been already shown to be activated at the transcriptional level under drought conditions (Menges *et al*., 2008).

Serine carboxypeptidase‐like protein 49 (SCPL49), which is involved in protein degradation (proteolysis), was identified among the drought‐adaptive genes in a cross‐species meta‐analysis comparing *Arabidopsis*, rice, wheat and barley plants (Shaar‐ Moshe *et al*., 2015). To the best of our knowledge, *SCPL19* has not previously been reported as a drought‐responsive gene. However, our studies clearly show that the down‐regulated expression of this gene in drought‐tolerant potato cultivars and the knockout of this gene activity in the *A. thaliana* mutant correlate with the enhanced tolerance to water deficit stress (see Figures 5 and S8).

Armadillo/beta‐catenin‐like repeat‐containing proteins are involved in developmental processes and stress signalling. In rice, several members of this family were found to be up‐regulated by drought, salt and/or cold stress (Sharma *et al*., 2014). In addition, several members of this family in rice and *Arabidopsis* contain a U‐Box motif and are thought to be implicated in protein degradation and response to drought stress (Cho *et al*., 2008; Seo *et al*., 2012; Sharma *et al*., 2014). However, proteins encoded by the *Arabidopsis AT4G16490* and potato *PGSC0003DMG400023602* genes do not contain the U‐Box motif. Expression of this gene was down‐regulated in the drought‐tolerant Tajfun cultivar and was also strongly down‐regulated on the fifth day of drought conditions in the corresponding *Arabidopsis* mutant (see Figures 5 and S8). The exact function of this gene product remains to be elucidated.

The expression of the homologous genes *AT5G14570*/*PGSC0003DMG400006913* in *Arabidopsis* and potato is strongly down‐regulated in the *Arabidopsis* mutant (no detectable expression) and the drought‐tolerant potato cultivar Tajfun. These genes encode NTR2.7, which is known to control nitrate content in dry seeds. One of the high‐affinity nitrate transporters (*MdNRT2.4)* has been found to be involved in the drought response in apple roots. The expression of this gene was elevated in plant roots likely due to the reduction of the nitrate concentration in response to drought (Bassett *et al*., 2014). However, AtNTR2.7 mRNA was absent in the drought‐tolerant *Arabidopsis* mutant, and in the drought‐tolerant potato cultivar Tajfun, the *NTR2.7* transcript level was down‐regulated. The exact role of *NTR2.7* in the plant response to drought has yet to be elucidated.

In the case of the *AT3G18280*/*PGSC0003DMG400005731* homologous genes, in the drought‐tolerant Tajfun cultivar, the expression of the potato gene was up‐regulated compared to that in the drought‐sensitive Owacja plants. However, in the *Arabidopsis* mutant of this gene, the expression was strongly down‐regulated, although the mutant was extremely resistant to drought stress. This gene encodes nonspecific lipid transfer protein type 2 (nsLPT). This gene has been previously identified in microarray studies as a marker for drought stress in *Arabidopsis* plants. Its expression was up‐regulated twofold by drought but was not affected by salt, cold or heat stress, suggesting that nsLPT may play a very specific role in the plant response to water shortage (El Ouakfaoui and Miki, 2005). nsLPTs play a role in phospholipid and fatty acid transfer between membranes (Kader, 1996). Some members of this family in plants have been shown to participate in wax and/or cutin monomer transport (Kim *et al*., 2013; Sterk *et al*., 1991). Detailed studies on the nsLPT gene family in maize showed induced but also down‐regulated expression of particular family members in response to drought. Our studies on potato and *Arabidopsis* support the importance of the nsLPT gene family in the plant response to drought. To explain the observed discrepancies in the gene expression profiles between the drought‐tolerant potato cultivar and the relevant *Arabidopsis* mutant, more detailed studies are required to reveal the functions of particular genes in this large family in both species studied.

The data presented in this study show a comparison of the gene expression profiles between closely related cultivars, which represents a successful approach for identifying genes responsive to drought with interspecies conserved functions.

## Experimental procedures

### Potato plant material and growth conditions

The following four potato cultivars were used: Gwiazda and Oberon (bred by Potato Breeding Zamarte Ltd., Co. IHAR‐PIB Group, Poland) and Tajfun and Owacja (bred by the Pomeranian‐Mazurian Potato Breeding Company, Strzekecino, Poland). Plants were grown as described in Boguszewska *et al*. (2010) (Data S1). All experiments were performed in three biological replicates. During the drought experiment, leaf samples were taken every day from three different plants for each cultivar at a given time point and frozen in liquid nitrogen. The third, fourth and fifth whirls from the top of the compound leaves were collected from each plant.

### *Arabidopsis* plant material and growth conditions

The following *A. thaliana* plants were used in this study: ecotype Columbia‐0 (wild‐type plants) and the homozygous T‐DNA insertion mutants GK‐310G07, SALK_118221, SALK_087895, SALK_047537, SALK_054063, SALK_018120 and SALK_095906 (Alonso *et al*., 2003; Kleinboelting *et al*., 2012). The plants were grown in soil (Jiffy‐7 42 mm; Jiffy Products International AS, Norway) in a growth chamber as described in Szarzynska *et al*. (2009). Four‐week‐old *Arabidopsis* plants were subjected to drought conditions by the cessation of watering for ten days without further rewatering.

### DNA and RNA isolation, cDNA synthesis and PCR and RT‐PCR amplification

Genomic DNA and total RNA were isolated from plant leaves as described in Szarzynska *et al*. (2009) (Data S1). All primer sequences are shown in Figure S2. The PCR and RT‐qPCR were performed as previously described (Pieczynski *et al*., 2013; Szarzynska *et al*., 2009).

### Relative water content (RWC) measurements

The RWC was calculated as described in Boguszewska *et al*. (2010) (Data S1).

### RNA‐seq

Total RNA was extracted from the plant tissues using TRIzol reagent as described in Pant *et al*. (2009) and Szarzynska *et al*. (2009) (Data S1). The strand‐specific library construction and RNA sequencing (RNA‐seq) (PE100) using an Illumina HiSeq 2500 were conducted by BGI Tech Solutions Co., Ltd (Hong Kong). Basic information regarding the RNA‐seq data is provided in Table S2. All row data have been deposited in the GEO database (GSE97776) under the link: https://www.ncbi.nlm.nih.gov/geo/query/acc.cgi?acc=GSE97776.

### Bioinformatic analyses of RNA deep sequencing data

Analysis of bioinformatic data is described in Data S1.

### Primer design

All PCR primers used for the expression analysis of selected genes in potato were designed as described in Data S1.

## Supporting information


**Figure S1** Genetic background of the four studied potato cultivars Gwiazda and Oberon (A) and Tajfun and Owacja (B).
**Figure S2** Gwiazda/Oberon and Tajfun/Owacja cultivar plants grown in the half‐open glasshouse on day 13 of drought.
**Figure S3** RT‐qPCR analysis of *RAB18* gene expression in leaves detached from Gwiazda and Oberon cultivar plants during the drought experiment.
**Figure S4** Schematic representation of drought‐related gene selection using RNA‐seq data from two pairs of potato cultivars, Gwiazda/Oberon and Tajfun/Owacja.
**Figure S5** Drought‐related gene selection pipeline.
**Figure S6** Eight stably expressed genes in all potato cultivars studied during drought stress.
**Figure S7** Seventeen of 23 potato genes selected after the third round of selection showing the highest differences in gene expression between the studied drought‐tolerant and drought‐sensitive cultivars during the drought experiment.
**Figure S8** Phenotypic and RWC analyses of three Arabidopsis mutant plants with altered expression of genes homologous to the selected drought‐related potato genes.
**Table S2** GO database annotation of the biological process, molecular function and cellular component categories of 8 genes selected from among the 22 top‐ranking *Arabidopsis* genes.
**Table S3** Primer sequences.
**Table S4** Summary of RNA‐seq Data.
**Data S1** Experimental procedures ancillary information.

**Table S1** Comparison of the normalized number of reads for transcripts derived from genes identified in the first round of selection (594 genes) during the time course of the drought experiment.
