# Supplementary material for: Genomewide identification of genes involved in the potato response to drought indicates functional evolutionary conservation with Arabidopsis plants
Source: Plant Biotechnol J. 2017 Aug 14;16(2):603–14. doi: 10.1111/pbi.12800 (PMC5787840; doi:10.1111/pbi.12800)
Supplement: Supplementary file 1 — Figure S1 Genetic background of the four studied potato cultivars Gwiazda and Oberon (A) and Tajfun and Owacja (B). Figure S2 Gwiazda/Oberon and Tajfun/Owacja cultivar plants grown in the half‐open glasshouse on day 13 of drought. Figure S3 RT‐qPCR analysis of RAB18 gene expression in leaves detached from Gwiazda and Oberon cultivar plants during the drought experiment. Figure S4 Schematic representation of drought‐related gene selection using RNA‐seq data from two pairs of potato cultivars, Gwiazda/Oberon and Tajfun/Owacja. Figure S5 Drought‐related gene selection pipeline. Figure S6 Eight stably expressed genes in all potato cultivars studied during drought stress. Figure S7 Seventeen of 23 potato genes selected after the third round of selection showing the highest differences in gene expression between the studied drought‐tolerant and drought‐sensitive cultivars during the drought experiment. Figure S8 Phenotypic and RWC analyses of three Arabidopsis mutant plants with altered expression of genes homologous to the selected drought‐related potato genes. Table S2 GO database annotation of the biological process, molecular function and cellular component categories of 8 genes selected from among the 22 top‐ranking Arabidopsis genes. Table S3 Primer sequences. Table S4 Summary of RNA‐seq Data. Data S1 Experimental procedures ancillary information. [file PBI-16-603-s001.docx]

**Supporting Info**

**Experimental procedures ancillary information**

**Potato plant material and growth conditions**

All potato plants were grown in 14-liter pots, each of which was filled with a thin layer of gravel at the bottom and 12 liters of universal vegetable soil substrate with the addition of chalk, pH range 5.5-6.5 (Hollas Company). Gum pipe was installed inside of each pot for better soil aeration (Boguszewska et al, 2010). Pest and disease control was carried out as follows: 3 times against Colorado beetles and 4 times against *Phytophthora infestans*. The plants were watered daily with an optimal water amount according to the volumetric water content (VWC) in the soil measured using 5TM Decagon sensors (Decagon Devices, Inc., Pullman, WA, USA). For the control conditions, the VWC was 0.361 m^3^/m^3^. Three weeks after the initiation of tuberization, half of the plant population was subjected to soil drought stress by the withdrawal of irrigation, while the control plants continued to be watered (Głuska, 2004). The VWC measurements on subsequent days of drought were as follows: 0.121, -0.040, -0.066 and -0.106 m^3^/m^3^ on the 3rd, 6th, 9th and 13th day of drought, respectively. After re-watering the plants, the VWC increased to the control level. All experiments were performed in three biological replicates.

During the drought experiment, leaf samples were collected daily from three different plants of each cultivar at a given time point and immediately frozen in liquid nitrogen for storage and further use. The third, fourth and fifth whirl from the top of the compound leaves were collected from each plant. Plants grown in pots were set on mobile platforms in a half-open greenhouse. In case of any weather changes, including rainfall, all platforms were moved inside the closed portion of the greenhouse; therefore, the plants were sheltered under the glass roof, and the soil and plants remained dry during the drought experiment while being maintained natural growth conditions.

**DNA and RNA isolation, cDNA synthesis, and PCR and RT-PCR amplification**

Genomic DNA and total RNA were isolated from plant leaves using the DNeasy Plant Mini Kit (Qiagen, Valencia, CA, USA) and Direct-zol RNA MiniPrep Kit (Zymo Research, Irvine, CA, USA). Total RNA was used for the reverse transcription reaction, which was performed using Superscript III RT (Invitrogen, Grand Island, NY, USA) and oligo-dT as a primer (Szarzynska *et al.,* 2009). All primer sequences are shown in Supplementary Table S1. The PCR was performed as previously described (Pieczynski *et al.,* 2013; Szarzynska *et al.,* 2009). The RT-qPCR was performed as previously described (Pieczynski *et al.,* 2013).

**Relative water content (RWC) measurements**

The RWC was calculated as follows: RWC (%) = [(FW - DW)/(SW - DW)] x 100, where FW, DW, and SW are the fresh, dry and saturated weights of the leaf tissues, respectively. The saturated leaf weight was measured by maintaining the leaf in water under light (150-200 μmol m^-2^s^-1^) at 22°C for 4 h. Then, the leaf was dried by maintaining the leaf at 80°C in an oven for 16 h. The RWC was measured 0, 3, 6, 9 and 13 days after the drought application. The RWC was measured from three leaves that were comparable in size and collected from the third level at the top of the plant (Boguszewska *et al*., 2010). Six biological replicates were analyzed for each treatment. The statistical analysis was performed using the Mann–Whitney *U*-test. For the Arabidopsis plants, we used the same procedure. The RWC was measured from three leaves that were comparable in size and collected from a plant rosette from a four-week-old plant. The RWC was measured on days 0, 3, and 6 after the drought application.

**RNA-seq**

Total RNA was extracted from the plant tissues using TRIzol reagent and chloroform reagents, followed by ethanol precipitation as previously described (Pant *et al.,* 2009; Szarzynska *et al.,* 2009). The quality of the RNA samples was determined using an Agilent RNA 6000 Nano Kit Guide (Agilent Technologies), and only the samples meeting the following criteria were used: 28S:18S > 1.0, RIN ≥ 6.5, OD 260/280 ≥ 1.8 and OD 260/230 ≥ 1.8.

The strand-specific library construction and RNA-sequencing (RNA-seq) (PE100) using an Illumina HiSeq 2500 were conducted by BGI Tech Solutions Co., Ltd. (Hong Kong). Basic information regarding the RNA-seq data is provided in TableS2. All row data have been deposited in the GEO database (GSE97776) under the link: <https://www.ncbi.nlm.nih.gov/geo/query/acc.cgi?acc=GSE97776>.

**Bioinformatic analyses of RNA deep sequencing data**

First, we tested the quality of the clean reads using FASTQC (http://www.bioinformatics.babraham.ac.uk/projects/fastqc/), and, if the quality was not questionable, the reads were mapped onto the reference potato genome (Solanaceae Genomics Resource, S. tuberosum Group Phureja DM1-3 Assembly Version 3 DM, PGSC Version 4.03 Pseudomolecule Sequence) using TopHat2 (Kim *et al.,* 2013) software. The number of mapped reads was obtained using Flux Capacitor v. 1.6.1 (Montgomery *et al.,* 2010) and the Python script fluxgtf2counts.py, which retrieves and concatenates transcript counts from Flux Capacitor GTF files (available at https://github.com/lpryszcz/bin/blob/master/fluxgtf2counts.py).

Flux Capacitor is a program that predicts the abundances of transcript molecules and alternative splicing events from RNA-seq experimental data. The input for the Flux Capacitor includes an annotation of a reference transcriptome (Solanaceae Genomics Resource, PGSC_DM_V403_genes.gtf - gene annotation for the v4.03 Pseudomolecules in GFF3 format) and reads from RNA-seq technologies aligned to the genome. Tables are then prepared for use in the statistical analysis. The statistical analysis was performed to identify the gene expression differences between the samples studied (package R [R Core Team (2014). R: A language and environment for statistical computing. R Foundation for Statistical Computing, Vienna, Austria. URL http://www.R-project.org/]. Flux Capacitor was run for each fastq file of every biological replicate from every condition. Then, the results were gathered in tables using the above-mentioned scripts, with one table per comparison (e.g., a table with 3 biological replicates from one variety exposed to drought stress for 6 days and 3 replicates of the same variety exposed to control conditions). These tables were then analyzed by DeSeq, which is an R package suitable for the analysis of differential gene expression based on a negative binomial distribution (Anders and Huber, 2010; Hastings *et al*., 2016).

**Primer design**

All PCR primers used for the expression analysis of selected genes in potato were designed as follows: (i) the sequences of all transcripts derived from the selected genes were collected; (ii) within the transcript sequences derived from each selected gene, a common fragment was identified as a target sequence for the primer design (to amplify all transcripts derived from each gene); (ii) common fragments were also analyzed by BLAST software to identify transcripts that are similar in sequence but are not derived from the selected genes; and (iv) all primers were designed to amplify common fragments in the selected genes without possibly amplifying similar transcripts. All primers were designed to amplify products ranging from 150 to 250 bp with a melting temperature of approximately 60°C. All primer sequences used in this study are presented in Table S1.

**Reference list:**

Anders, S., Huber, W., (2010) *Differential expression analysis for sequence count data.* Genome Biology **11**(10):R106.

[Hastings, E](https://www.ncbi.nlm.nih.gov/pubmed/?term=Hastings%20E%5BAuthor%5D&cauthor=true&cauthor_uid=26481351)., [Weisser, H](https://www.ncbi.nlm.nih.gov/pubmed/?term=Weisser%20H%5BAuthor%5D&cauthor=true&cauthor_uid=26481351)., [Wright, J](https://www.ncbi.nlm.nih.gov/pubmed/?term=Wright%20J%5BAuthor%5D&cauthor=true&cauthor_uid=26481351)., [Jaiswal, P](https://www.ncbi.nlm.nih.gov/pubmed/?term=Jaiswal%20P%5BAuthor%5D&cauthor=true&cauthor_uid=26481351)., [Huber, W](https://www.ncbi.nlm.nih.gov/pubmed/?term=Huber%20W%5BAuthor%5D&cauthor=true&cauthor_uid=26481351)., [Choudhary, J](https://www.ncbi.nlm.nih.gov/pubmed/?term=Choudhary%20J%5BAuthor%5D&cauthor=true&cauthor_uid=26481351)., [Parkinson, H. E](https://www.ncbi.nlm.nih.gov/pubmed/?term=Parkinson%20HE%5BAuthor%5D&cauthor=true&cauthor_uid=26481351)., [Brazma, A](https://www.ncbi.nlm.nih.gov/pubmed/?term=Brazma%20A%5BAuthor%5D&cauthor=true&cauthor_uid=26481351)., (2016) *Expression atlas update- an integrated database of gene and protein expression in humans, animals and plants.* Nucleic Acids Research **44** (D1): D746-52.

Kim, D., Pertea, G., Trapnell, C., Pimentel, H., Kelley, R., Salzberg, S. L.,(2013) *TopHat2: accurate alignment of transcriptomes in the presence of insertions, deletions and gene fusions.* Genome Biology **14**(4):R36.

Montgomery, S. B., Sammeth, M., Gutierrez- Arcelus, M., Lach, R. P., Ingle, C., Nisbett, J., Guigo, R., and Dermitzakis, E. T., (2010) *Transcriptome genetics using second generation sequencing in a Caucasian population.* Nature **464** (7289):10.

**Supplemental figures**


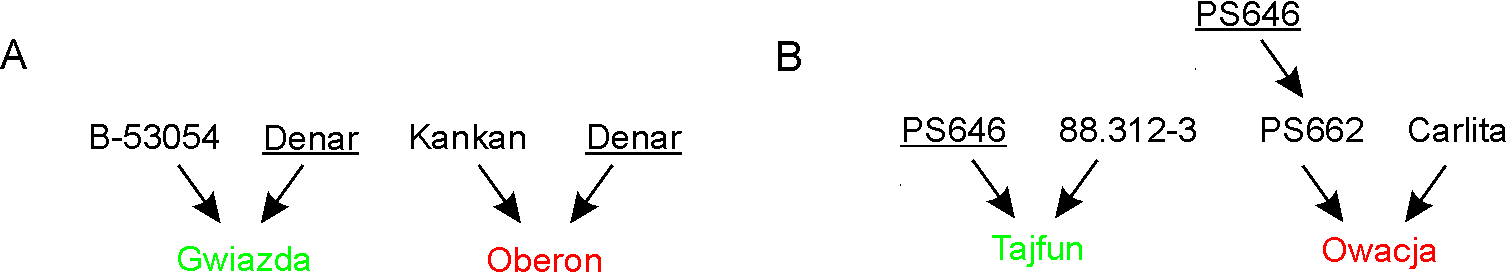


**Figure S1 Genetic background of the four studied potato cultivars Gwiazda and Oberon (A) and Tajfun and Owacja (B)**. The drought-tolerant cultivars are marked in green, while the drought-sensitive cultivars are marked in red.


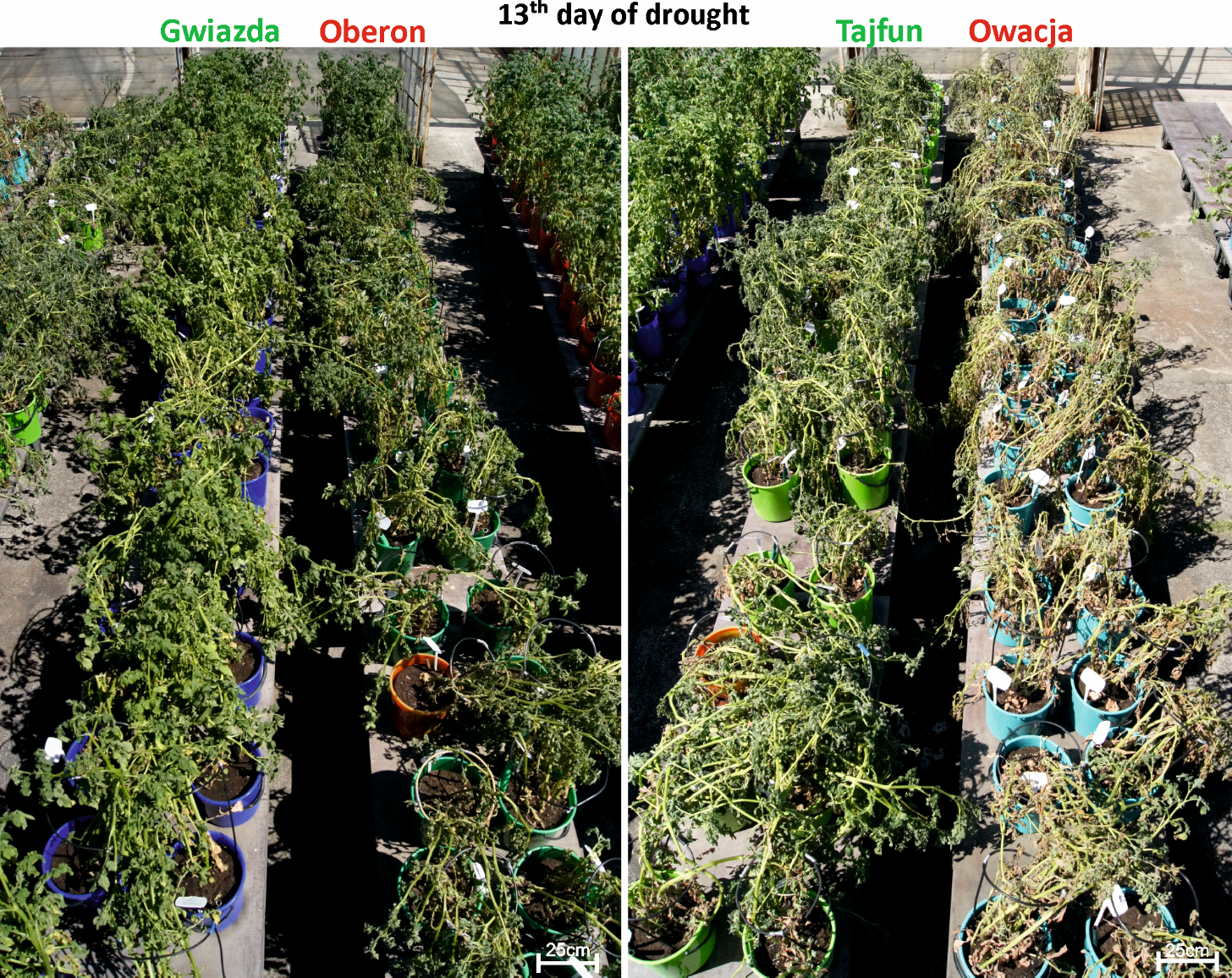


**Figure S2 Gwiazda/Oberon and Tajfun/Owacja cultivar plants grown in the half-open greenhouse on the day 13 of drought.**

**
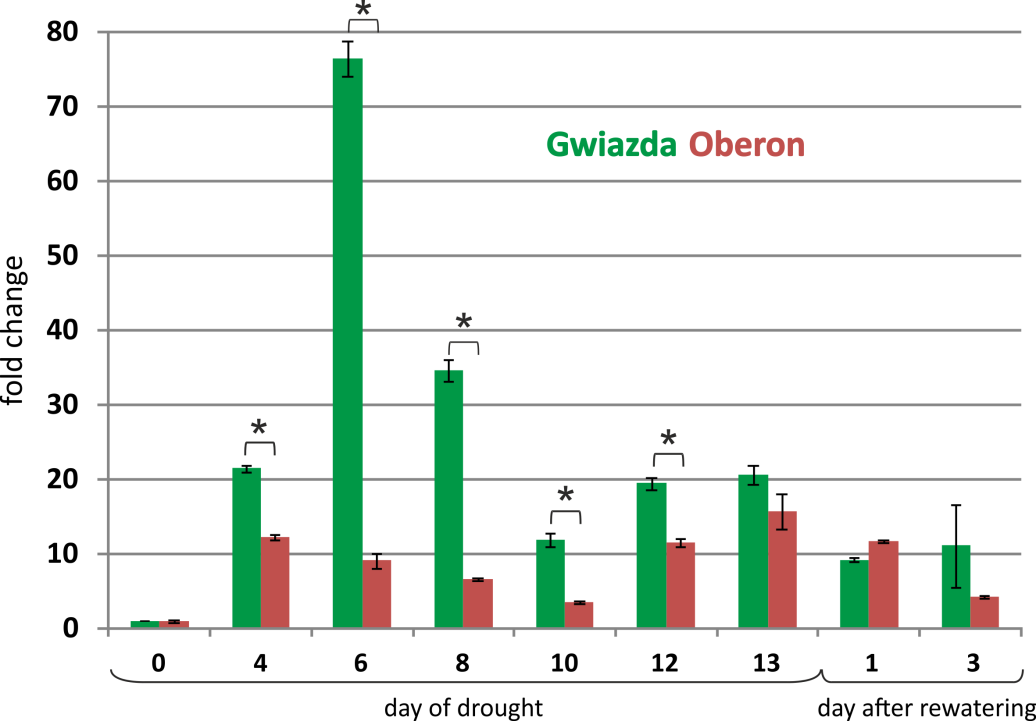
**

**Figure S3 RT-qPCR analysis of *RAB18* gene expression in leaves detached from Gwiazda and Oberon cultivar plants during the drought experiment.** Fold change was calculated using the day 0 expression data as a control. Green bars – Gwiazda *RAB18* expression data; red bars – Oberon *RAB18* expression data. Statistical analysis was performed using the Mann-Whitney *U*-test. * *P* < 0.05.


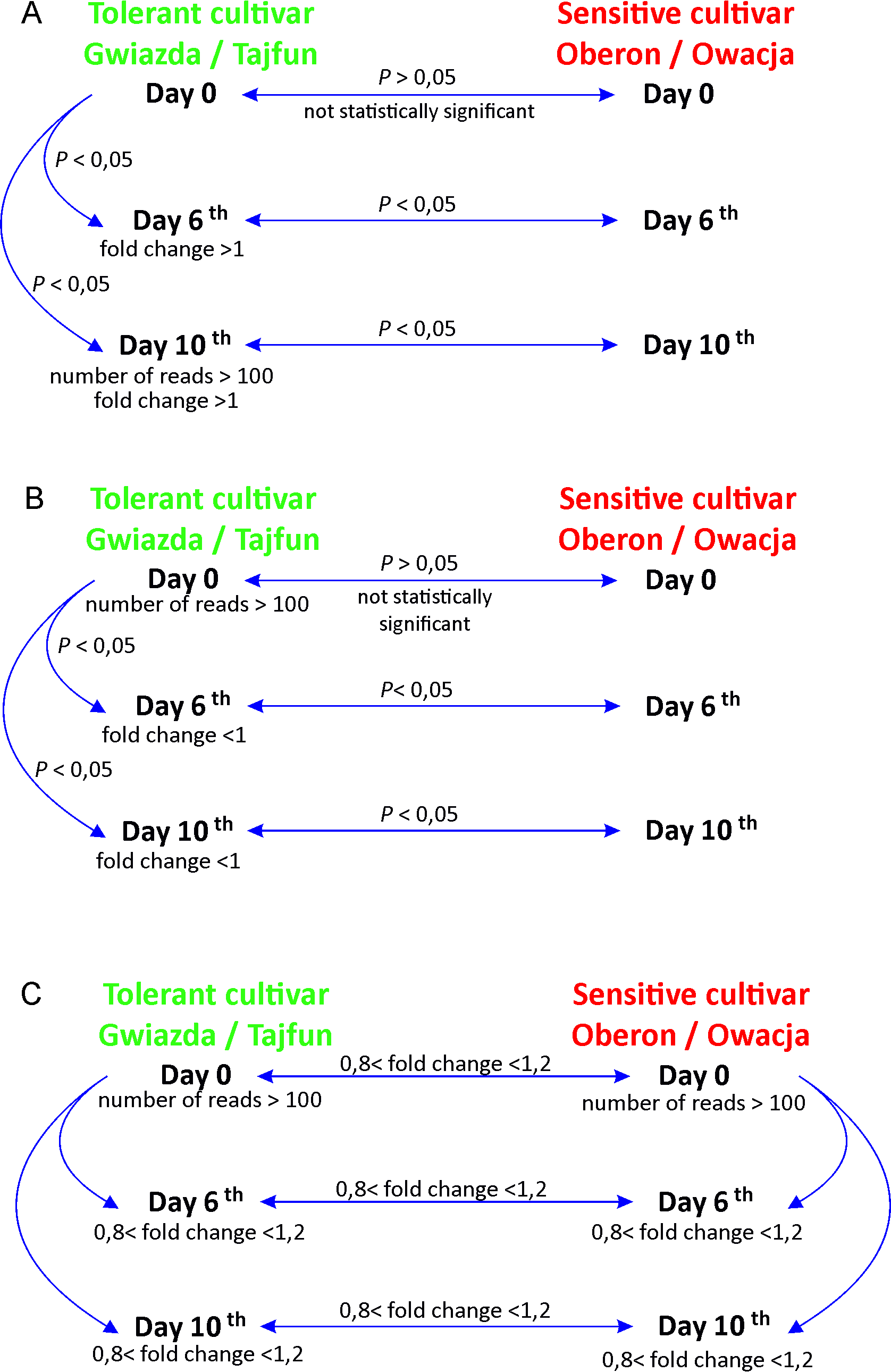


**Figure S4 Schematic representation of drought-related gene selection using RNA-seq data from two pairs of potato cultivars, Gwiazda/Oberon and Tajfun/Owacja.** The selection procedure for differentially expressed genes was performed separately within each pair of drought-tolerant/drought-sensitive cultivars. (A) Selection of upregulated genes in tolerant cultivars compared with sensitive cultivars. (B) Selection of downregulated genes in tolerant cultivars compared with sensitive cultivars. (C) Selection of genes stably expressed in all four cultivars. All arrows depict two data sets derived from different RNA-seq samples (from a selected cultivar and a selected day of drought), which were processed during gene selection. During the selection process, only the number of normalized reads for the analyzed genes was considered. (A) and (B): only genes showing non-significant differences in their expression on day 0 were considered (*P* > 0.05, horizontal two-headed arrows). Only genes showing statistically significant differences in their expression between tolerant and sensitive cultivars on days 6 and 10 were considered (*P* < 0.05, horizontal two-headed arrows). Only genes showing statistically significant differences in their expression in the tolerant cultivars between days 0 and 6, and days 0 and 10 were considered (*P* < 0.05, vertical arrows). (C) Only genes showing statistically non-significant differences in their expression between the tolerant and sensitive cultivars on days 0, 6 and 10 were considered (fold change between 0.8 and 1.2, vertical arrows and horizontal two-headed arrows).


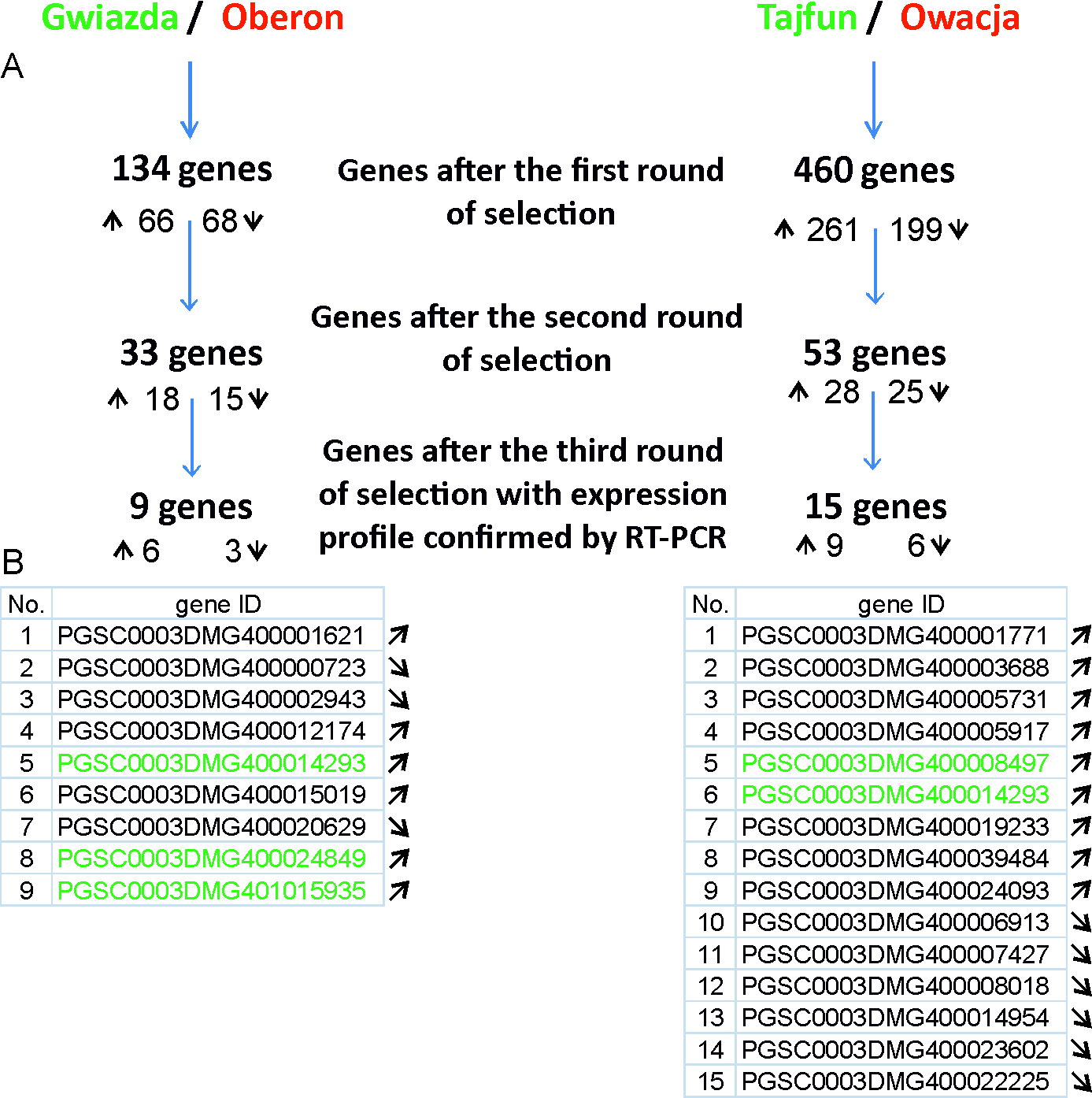


**Figure S5 Drought-related gene selection pipeline.** (A) The first round of selection shows the number of genes selected after the RNA-seq analyses whose expression was significantly altered (Table S3). Genes from the first round of selection were filtered based on the highest differences in expression profiles obtained from the RNA-seq data on the 6^th^ and 10^th^ days of the drought experiment (see also Table S3). Genes from the second round of selection were filtered based on the confirmation of the gene expression differences in particular genes in the drought-tolerant and drought-sensitive cultivars using RT-PCR (Fig. 7 and Fig. S4). The numbers of genes shown in bold represent genes with altered expression. Gene numbers below the down arrowheads represent genes with downregulated expression in the drought-tolerant versus drought-sensitive cultivars. Gene numbers with up arrowheads represent genes with upregulated expression in the drought-tolerant versus drought-sensitive cultivars. (B) Accession numbers of the top selected genes taken from the Potato Genomic Resource (Hirsch *et al*., 2014). Green indicates genes that are already known to be involved in the potato drought response.


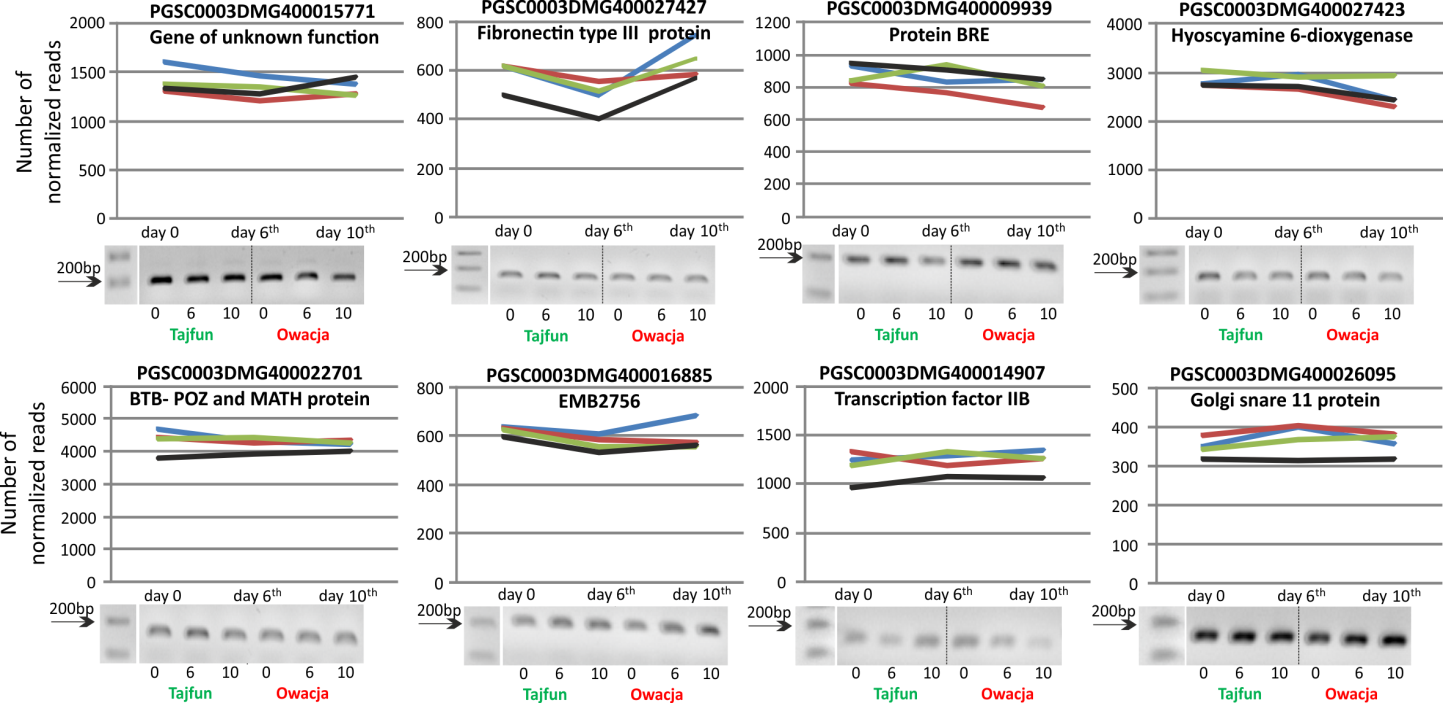


**Figure S6 Eight stably expressed genes in all potato cultivars studied during drought stress.** The upper panels present graphs with the normalized number of reads of genes from the Tajfun/Owacja and Gwiazda/Oberon cultivars. Above each graph, the accession number of a given gene and its function are displayed. Tajfun – green lines, Owacja – black lines, Gwiazda – blue lines, Oberon – red lines. The lower panel shows the gel electrophoresis analysis of the RT-PCR products of all eight genes, confirming the RNA-seq results from plants collected on days 0, 6, and 10.

**
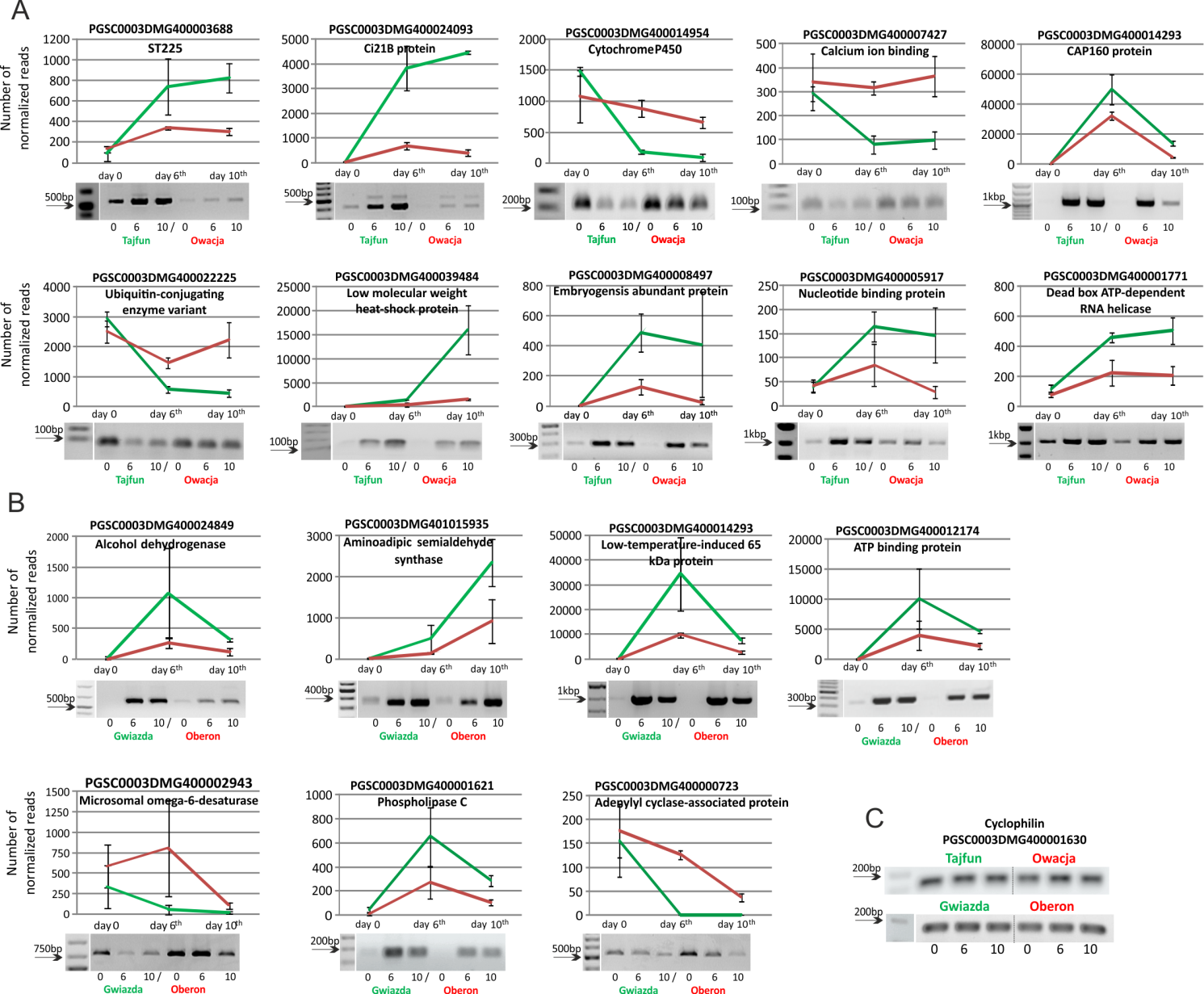
**

**Figure S7 Seventeen of twenty-three potato genes selected after the third round of selection showing the highest differences in gene expression between the studied drought-tolerant and drought-sensitive cultivars during the drought experiment.** (A, B) The data for Tajfun/Owacja and Gwiazda/Oberon, respectively. Above each graph, the accession number of a given gene and its function are displayed. Graphs show the normalized number of reads for each gene. Values are shown as the mean ± SD (n = 3) of three independent RNA-seq experiments, *P* < 0.05. Statistical methods for the differential gene expression analysis are described in the Materials and Methods section. Below each graph, the gel electrophoresis analysis of the RT-PCR products of all 17 genes is presented, confirming the RNA-seq results. (C) All RT-PCR experiments were performed using cyclophilin gene expression as a control. The numbers at the bottom of each gel indicate the days of the drought experiment.


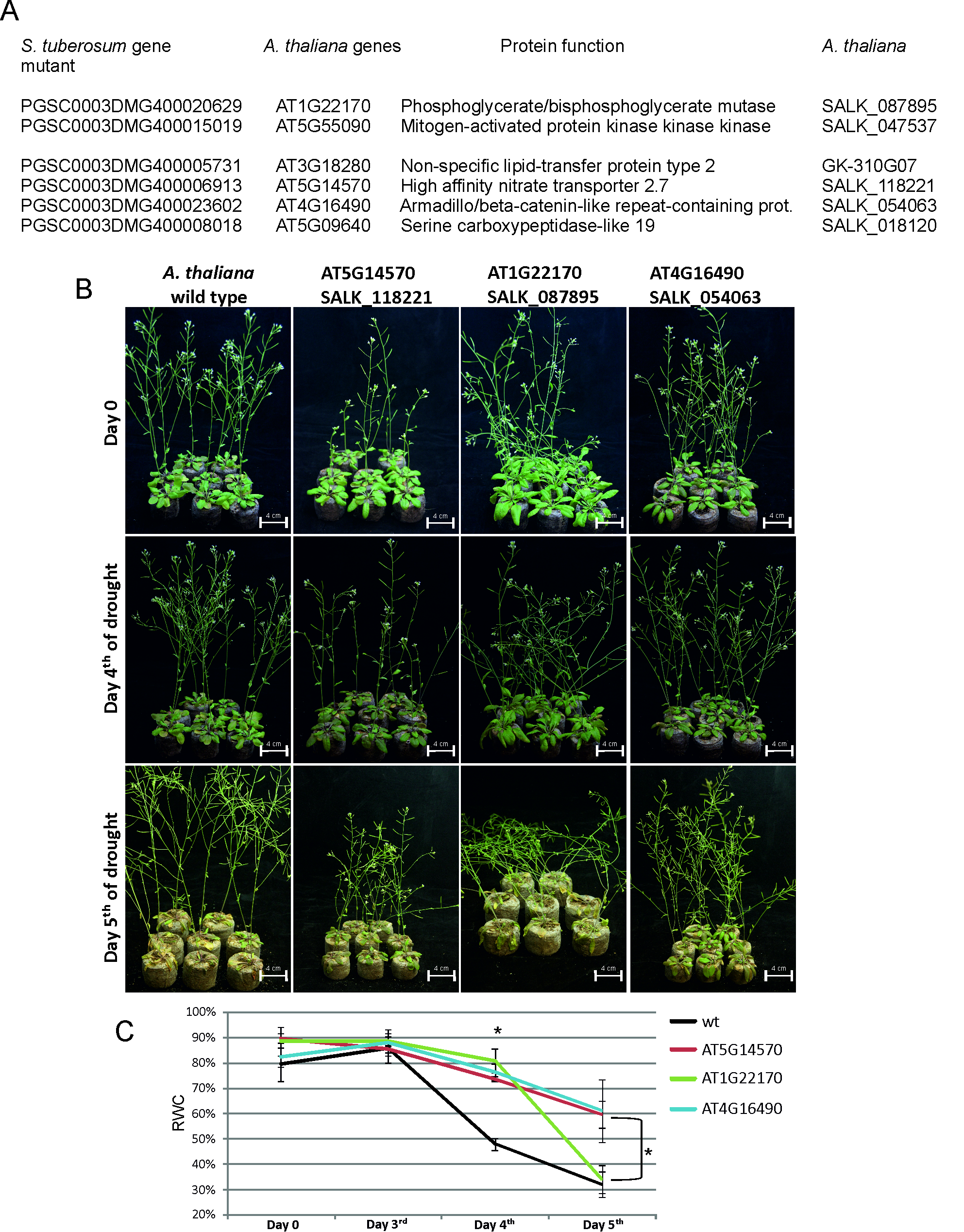


**Figure S8 Phenotypic and RWC analyses of three Arabidopsis mutant plants with altered expression of genes homologous to the selected drought-related potato genes.** (A) Accession numbers of the seven top-ranking drought-related potato genes (left column) and the accession numbers and protein functions of their Arabidopsis homologs (middle columns) were obtained from the Spud DB and TAIR database, respectively (Hirsch *et al*., 2014; Huala *et al*., 2001). The right column presents the accession numbers of selected Arabidopsis SALK or GABI-Kat mutants of each presented gene (Alonso *et al*., 2003; Kleinboelting *et al*., 2012). (B) Arabidopsis plants subjected to water stress. Plants are shown on days 0, 3, 6, and 10 of the drought experiment. Only wild-type and three mutant plants are shown (the other four mutants are presented in Fig. 8). (C) Two of three Arabidopsis mutant plants show higher water content than the wild-type plants on the 5^th^ day of drought. RWC was measured 0, 3, 4 and 5 days after the introduction of drought stress. Values are shown as the mean ± SD (n = 3) of three independent experiments. * *P* < 0.001, Mann–Whitney *U*-test.

**Table S1 Comparison of the normalized number of reads for transcripts derived from genes identified in the first round of selection (594 genes) during the time course of the drought experiment.** Gray color indicates the selected genes after the second round of selection. The mean value of the normalized reads per million is shown for selected potato transcripts that were downregulated in the Gwiazda plants compared to those in the Oberon plants. The data are shown for days 0, 6, and 10 of the drought experiment and were obtained from three biological replicates. The SD at day 0 was never statistically significant (*P* > 0.05), while the SDs at day 6 and day 10 were always statistically significant (*P* < 0.05, see also Fig. 5). Potato gene accession numbers were obtained from the Spud DB (www.potato.plantbiology.msu.edu). SD - standard deviations. (see separate file Table S3).

**Table S2 GO database annotation of the biological process, molecular function and cellular component categories of 8 genes selected from among the 22 top-ranking Arabidopsis genes**. g.f. - gene function (according to the TAIR database); different colors depict various abiotic stresses. Only genes that were found to be annotated as abiotic stress-responsive in the GO database are shown.

|  | **AT5G52300** | **AT1G77120** | **AT1G59860** | **AT3G20660** | **AT3G51810** | **AT4G27030** | **AT4G33150** | **AT3G11170** |
| --- | --- | --- | --- | --- | --- | --- | --- | --- |
| **g.f**. | Low-Temperature-Induced 65 | Alcohol Dehydrogenase | HSP20-like | Carbohydrate transporter/sugar porter | Late Embryogenesis Abundant 1 | Fatty Acid Desaturase 4 | Lysine-Ketoglutarate Reductase/ Saccharopine Dehydrogenase, | Fatty Acid Desaturase 7 |
| **biological process** | response to water deprivation | response to water deprivation | response to osmotic stress | cellular response to water deprivation | response to abscisic acid | response to karrikin | response to karrikin | response to cold |
|  | response to abscisic acid | response to abscisic acid | response to heat | transmembrane transport | embryo development ending in seed dormancy | unsaturated fatty acid biosynthetic process | saccharopine dehydrogenase (NADP+, L-lysine-forming) activity | oxidation-reduction process |
|  | response to salt stress | response to osmotic stress | response to salt stress | carbohydrate transmembrane transporter activity |  | oxidation-reduction process | L-lysine catabolic process | fatty acid biosynthetic process |
|  | response to cold | response to cold | protein oligomerization | substrate-specific transmembrane transporter activity |  | delta 3-trans-hexadecenoic acid phosphatidylglycerol desaturase activity | saccharopine dehydrogenase activity | unsaturated fatty acid biosynthetic process |
|  | abscisic acid-activated signaling pathway | response to salt stress | protein folding | sugar-proton symporter activity |  | ubiquitin protein ligase activity |  | oxidoreductase activity, acting on paired donors, with oxidation of a pair of donors resulting in the reduction of molecular oxygen to two molecules of water |
|  | leaf senescence | oxidation-reduction process |  | ion transport |  | phosphatidylglycerol metabolic process |  |  |
| **molecular function** | Molecular function | nucleotide binding | protein binding | carbohydrate transmembrane transporter activity | Molecular function | delta 3-trans-hexadecenoic acid phosphatidylglycerol desaturase activity | saccharopine dehydrogenase (NADP+, L-lysine-forming) activity | oxidoreductase activity, acting on paired donors, with oxidation of a pair of donors resulting in the reduction of molecular oxygen to two molecules of water |
|  |  | alcohol dehydrogenase (NAD) activity |  | substrate-specific transmembrane transporter activity |  | ubiquitin protein ligase activity | saccharopine dehydrogenase activity |  |
|  |  | zinc ion binding |  | ATP binding |  | ubiquitin protein ligase binding |  |  |
|  |  | protein homodimerization activity |  | Sugar - proton symporter activity |  |  |  |  |
| **cellular component** | cytoplasm | cytosol | cytoplasm | plant-type vacuole membrane | cytosol | integral component of membrane | cytosol | integral component of membrane |
|  |  | cytoplasm |  | integral component of membrane | cytoplasm | chloroplast | mitochondrion | chloroplast envelope |
|  |  | plasma membrane |  | plasma membrane |  | chloroplast membrane | cytoplasm | plastid membrane |
|  |  |  |  | membrane |  |  |  | chloroplast |

**Table S3 Primer sequences.**

(A) Primer sequences used for the RT-PCR amplification of the selected potato transcripts.

GW - Gwiazda /Oberon cultivars, TA - Tajfun /Owacja cultivars, Stub - specific primers used for all varieties. F - forward, R - reverse. Accession numbers were obtained from the Potato Genomic Resource (Hirsch *et al*., 2014).

(B) Primer sequences used for the RT-PCR amplification of selected *A. thaliana* transcripts. F - forward, R - reverse. Accession numbers were obtained from the TAIR database (Huala *et al*., 2001).

(C) Primer sequences used for *A. thaliana* SALK mutant genotyping. F - forward, R - reverse. Accession numbers were obtained from the TAIR database and SALK database (Huala *et al*., 2001; Alonso *et al*., 2003).

(A)

| **ID** | **target gene** | **primer name** | **Primer sequence (5'-3')** |
| --- | --- | --- | --- |
| 1 | PGSC0003DMG400000723 | GW_0723_F | CTGTCGAAAGCTCCTTCACC |
| 2 | PGSC0003DMG400000723 | GW_0723_R | TAGGAGCTGAGCCCTGACAT |
| 3 | PGSC0003DMG400002943 | GW_2943_F | ACAAACCAAAATGGGAGGTG |
| 4 | PGSC0003DMG400002943 | GW_2943_R | TTTGAAGGGGCGCTACATAC |
| 5 | PGSC0003DMG400020629 | GW_20629_F | TATGCTTCACTGCCAACGAG |
| 6 | PGSC0003DMG400020629 | GW_20629_R | TGGCTTGTTCAGTCTCATCG |
| 7 | PGSC0003DMG400015688 | GW_15688_F | AGGACTCTGCTCAACGGAGA |
| 8 | PGSC0003DMG400015688 | GW_15688_R | GACCACCTCAAAACGTCCAT |
| 9 | PGSC0003DMG400004709 | GW_04709_F | CAACTTCTCCCCATCCACAT |
| 10 | PGSC0003DMG400004709 | GW_04709_R | TGATCATGCTGTTCCTGAGC |
| 11 | PGSC0003DMG400015157 | GW_15157_F | AGAGAAGACCAACGCCGATA |
| 12 | PGSC0003DMG400015157 | GW_15157_R | TCATGCGAAAAACGACTCAC |
| 13 | PGSC0003DMG402031322 | GW_31322_F | TTCGCTTCCTTCTGGACCTA |
| 14 | PGSC0003DMG402031322 | GW_31322_R | CCGAAAATCCTCCTGATGAA |
| 15 | PGSC0003DMG400025009 | GW_25009_F | TCGTCTGAGACTGGACGTTG |
| 16 | PGSC0003DMG400025009 | GW_25009_R | AAGGCGATCGGAGTAGGAAT |
| 17 | PGSC0003DMG402003815 | GW_03815_F | GCCTCTCCCAATTGAGTTACA |
| 18 | PGSC0003DMG402003815 | GW_03815_R | GCTTGCTATCGATAAGGGTCA |
| 19 | PGSC0003DMG402006147 | GW_6147_F | TTTTGGAGTCGGGTGTCTTC |
| 20 | PGSC0003DMG402006147 | GW_6147_R | TCTCAGCACCAATGATCGAG |
| 21 | PGSC0003DMG402020908 | GW_20908_F | TTCCAAGCCTTGTGATCTCC |
| 22 | PGSC0003DMG402020908 | GW_20908_R | TGTTTTTGCTGCTACTGATCCT |
| 23 | PGSC0003DMG400018328 | GW_18328_F | AGTGGGAAGCAATTATGTGGA |
| 24 | PGSC0003DMG400018328 | GW_18328_R | TTGCAGAGCCATCTCTACGG |
| 25 | PGSC0003DMG400005038 | GW_5038_F | CACTGGTGACGTCAATTTCG |
| 26 | PGSC0003DMG400005038 | GW_5038_R | ATTTGGCGCTTTTTCTTCCT |
| 27 | PGSC0003DMG400007079 | GW_07079_F | GCCCATTGCTTTTGTGATTT |
| 28 | PGSC0003DMG400007079 | GW_07079_R | CCTCAGCAGATTTGCTTTCC |
| 29 | PGSC0003DMG400001368 | GW_01368_F | CCCATGGCAACCATTACTCT |
| 30 | PGSC0003DMG400001368 | GW_01368_R | TAAGCCGGGGTTTTAGGAGT |
| 31 | PGSC0003DMG400001621 | Gw_01621_F | CCTATTCCAGTTCTGCTTCAAG |
| 32 | PGSC0003DMG400001621 | Gw_01621_R | GTCAAACTTCTCGAACAATCACTCA |
| 33 | PGSC0003DMG400014293 | Gw_14293_F | CCGAAACTCGCGAAACTAAG |
| 34 | PGSC0003DMG400014293 | Gw_14293_R | AAACCGGAGCAAGTTTTCCTG |
| 35 | PGSC0003DMG400015019 | Gw_15019_F | GCCAATTGGAGGGACAATGG |
| 36 | PGSC0003DMG400015019 | Gw_15019_R | GCCTCGGAAAATTGAGACTG |
| 37 | PGSC0003DMG400024849 | Gw_24849_F | ATGCCAAGAAATTCGGTGTC |
| 38 | PGSC0003DMG400024849 | Gw_24849_R | TCGGGCCTTTTAGAACACAC |
| 39 | PGSC0003DMG401015935 | Gw_15935_F | GTAGCCACCTGCCAAGACAT |
| 40 | PGSC0003DMG401015935 | Gw_15935_R | GATGCCTCTTTCGCAAACTC |
| 41 | PGSC0003DMG400004999 | Gw_04999_F | CGTTCAATCCAAGGTTTACCA |
| 42 | PGSC0003DMG400004999 | Gw_04999_R | GTGACAGTCTTCATTTCCCATTG |
| 43 | PGSC0003DMG400013417 | Gw_13417_F | TGGCAGCTTCTACAATTGCTC |
| 44 | PGSC0003DMG400013417 | Gw_13417_R | CCTTTTATCAAATGATTGGAGGAC |
| 45 | PGSC0003DMG400020708 | Gw_20708_F | ATGGGAATAGCAGCAGTTGG |
| 46 | PGSC0003DMG400020708 | Gw_20708_R | TTAATGGTGGGATTGGCATT |
| 47 | PGSC0003DMG400027945 | Gw_27945_F | AGGCAAACTCGTGGTACTG |
| 48 | PGSC0003DMG400027945 | Gw_27945_R | ACTCCCCGAATCTTGGAACT |
| 49 | PGSC0003DMG402022579 | Gw_22579_F | TCGTTGCAATTTCAGGGATA |
| 50 | PGSC0003DMG402022579 | Gw_22579_R | CTGAAATTGCAACGAAAAGACG |
| 51 | PGSC0003DMG400047155 | Gw_47155_F | AGAAGGTGTTTCCATGGTGGAGG |
| 52 | PGSC0003DMG400047155 | Gw_47155_R | CACCAGGACCATGAGGAGGTTTGT |
| 53 | PGSC0003DMG400022591 | Gw_22591_F | AAATTGAAAGGGATCGAGCA |
| 54 | PGSC0003DMG400022591 | Gw_22591_R | TGAGTTGCCATTGAGTTCGT |
| 55 | PGSC0003DMG400019944 | Gw_19944_F | GAGCAGTGGATGCGTTCAGT |
| 56 | PGSC0003DMG400019944 | Gw_19944_R | CTATGCAAGGTTGAGATGAAGA |
| 57 | PGSC0003DMG400014226 | Gw_14226_F | GGGTGATTGTTTGCCAGAGT |
| 58 | PGSC0003DMG400014226 | Gw_14226_R | GTTTTCAACCGATGCCAAGT |
| 59 | PGSC0003DMG400012228 | Gw_12228_F | GGAATTCATCCGACACCAAG |
| 60 | PGSC0003DMG400012228 | Gw_12228_R | TGCAGAAACCCAAAATCACA |
| 61 | PGSC0003DMG400008833 | Gw_08833_F | AAGCGAGCGAGTATGAGGAA |
| 62 | PGSC0003DMG400008833 | Gw_08833_R | GGCGGTAAGTAACAGGGACA |
| 63 | PGSC0003DMG400011630 | Gw_11630_F | TCGAATGGCTTGTTCTCAGAT |
| 64 | PGSC0003DMG400011630 | Gw_11630_R | CAGACATTATTGACAAAGGGCC |
| 65 | PGSC0003DMG400006458 | Gw_06458_F | TTACCGGACACCATTGGATT |
| 66 | PGSC0003DMG400006458 | Gw_06458_R | AAGACGACGAGGAGAAAGCA |
| 67 | PGSC0003DMG400007427 | Ta-7427-F | CGCGGGGAAAGTAGAACGAT |
| 68 | PGSC0003DMG400007427 | Ta-7427-R | CTCCCCATTTGATCCCGGAG |
| 69 | PGSC0003DMG400008018 | Ta-8018-F | CTACTATTTTTTGGAATCAGAATCC |
| 70 | PGSC0003DMG400008018 | Ta-8018-R | TGGTATCATCTTGCCAGCATA |
| 71 | PGSC0003DMG400014954 | Ta-14954-F | TCGAACCCTAGGACTCATGG |
| 72 | PGSC0003DMG400014954 | Ta-14954-R | AGTAAATCGCTTCCCAGCAA |
| 73 | PGSC0003DMG400022225 | Ta-22225-F | TCCAACATTGCCACAATCAC |
| 74 | PGSC0003DMG400022225 | Ta-22225-R | CAATGTGAAGCAGGAATTGA |
| 75 | PGSC0003DMG400008262 | Ta-8262-F | CCATCGGAGAAAACACCACT |
| 76 | PGSC0003DMG400008262 | Ta-8262-R | GCACGCCAAGGTACTCTCTC |
| 77 | PGSC0003DMG400020118 | Ta-20118-F | GACATGCACTGTGCTCATCA |
| 78 | PGSC0003DMG400020118 | Ta-20118-R | TGAGCTCATCGTGGAGAGAA |
| 79 | PGSC0003DMG400011321 | Ta-11321-F | CCTTGATGGGCATTCTTCGA |
| 80 | PGSC0003DMG400011321 | Ta-11321-R | ATCACGAGCTGCTCCACCA |
| 81 | PGSC0003DMG400016319 | Ta-16319-F | CCCTTGAGACGAGACTCCAC |
| 82 | PGSC0003DMG400016319 | Ta-16319-R | CAACTAGATCAAGATCAAGATTCCAT |
| 83 | PGSC0003DMG400030905 | Ta-30905-F | TTCGACTGTTGCAGAGGTTG |
| 84 | PGSC0003DMG400030905 | Ta-30905-R | ACTCTGACGCCAGCAGTTG |
| 85 | PGSC0003DMG400031081 | Ta-31081-F | GCATGCAATCCAGTAACGAA |
| 86 | PGSC0003DMG400031081 | Ta-31081-R | CAATCCATGAGTCGGAACCT |
| 87 | PGSC0003DMG400031262 | Ta-31262-F | TTATGCAAGACATTCCACCA |
| 88 | PGSC0003DMG400031262 | Ta-31262-R | CAGAGACTATATATCGAGGGAAGAA |
| 89 | PGSC0003DMG401004637 | Ta-4637-F | AGAACGACGGAGAAGAGGGT |
| 90 | PGSC0003DMG401004637 | Ta-4637-R | GTCTGGGGCAGCTCTAAGTG |
| 91 | PGSC0003DMG401012244 | Ta-1012244-F | GAATCATCACCGCCATTACC |
| 92 | PGSC0003DMG401012244 | Ta-1012244-R | GCTGTTGGACAAGCAGCATA |
| 93 | PGSC0003DMG400028164 | Ta-28164-F | AACCACTGAACGCGACATCA |
| 94 | PGSC0003DMG400028164 | Ta-28164-R | GGTGCATGTTGGAAGCTTGG |
| 95 | PGSC0003DMG400005035 | Ta-5035-F | CTCATGCCTGCTACGATGAA |
| 96 | PGSC0003DMG400005035 | Ta-5035-R | TTCCCTGTTATGCTCAACACC |
| 97 | PGSC0003DMG400001771 | Ta-1771-F | ACGAAATCTGGCAAAACTGG |
| 98 | PGSC0003DMG400001771 | Ta-1771-R | TGTCCACTCCTTCCTGCTCT |
| 99 | PGSC0003DMG400003688 | Ta-3688-F | TATGGCAGGTTGGGACTCTC |
| 100 | PGSC0003DMG400003688 | Ta-3688-R | ACAATCCCGCGAACAAATAA |
| 101 | PGSC0003DMG400005731 | Ta-5731-F | TGCACTAATGGTACTTTCTGAACAA |
| 102 | PGSC0003DMG400005731 | Ta-5731-R | TGTAAATCCAAGGCCAATGA |
| 103 | PGSC0003DMG400005917 | Ta-5917-F | ACCTAGTGGTTCGCCCTTTT |
| 104 | PGSC0003DMG400005917 | Ta-5917-R | ATGAGAAGCAGAGGCGACAT |
| 105 | PGSC0003DMG400008497 | Ta-8497-F | GGCATCAGAACAAGAAGAACG |
| 106 | PGSC0003DMG400008497 | Ta-8497-R | GCCTTTTCGACCCATTTCTT |
| 107 | PGSC0003DMG400014293 | Ta-14293-F | CCGAAACTCGCGAAACTAAG |
| 108 | PGSC0003DMG400014293 | Ta-14293-R | TGCAAATTCAGTTGCTGAGG |
| 109 | PGSC0003DMG400019233 | Ta-19233-F | GCCATGAAGATCATGCGTAA |
| 110 | PGSC0003DMG400019233 | Ta-19233-R | CGCCATAGAGCACAATTTCA |
| 111 | PGSC0003DMG400021158 | Ta-21158-F | GCCGTTTCAGCAGAAGTCTC |
| 112 | PGSC0003DMG400021158 | Ta-21158-R | ATGCAAAAATGTGAGCGACA |
| 113 | PGSC0003DMG400024093 | Ta-24093-F | CACAACATTATCTCATCACTCACG |
| 114 | PGSC0003DMG400024093 | Ta-24093-R | CAAGCACTCTGAAGGGAAACA |
| 115 | PGSC0003DMG400039484 | Ta-39484-F | CTTTGGTCGGAGCAATATCGTCG |
| 116 | PGSC0003DMG400039484 | Ta-39484-R | CAGAGGATGAAGGGTTAGCAATTGG |
| 117 | PGSC0003DMG400000839 | Ta-839-F | ATGCCTCAAAGAAGCTCGAA |
| 118 | PGSC0003DMG400000839 | Ta-839-R | TTTCCCAGGCACCATCTTAG |
| 119 | PGSC0003DMG400010498 | Ta-10498-F | GAAAGGTTATAGTCGCCCCTAGT |
| 120 | PGSC0003DMG400010498 | Ta-10498-R | TCTCTTTTAATGATTCACACGAAAC |
| 121 | PGSC0003DMG400011646 | Ta-11646-F | CGATAAGGCCGTTCTCTTTC |
| 122 | PGSC0003DMG400011646 | Ta-11646-R | TCGCTGACTACAACTGCTACAA |
| 123 | PGSC0003DMG400012408 | Ta-12408-F | ACATGGTTGAGGGAAAATCG |
| 124 | PGSC0003DMG400012408 | Ta-12408-R | GACAGCTGCTTGGCTACTCC |
| 125 | PGSC0003DMG400022844 | Ta-22844-F | AAAGCCCAGAAGCTCTTTCC |
| 126 | PGSC0003DMG400022844 | Ta-22844-R | CTGGCTAGGGTCTCATCAGC |
| 127 | PGSC0003DMG400029732 | Ta-29732-F | GCAAACTCAGAAAGAGCCTCA |
| 128 | PGSC0003DMG400029732 | Ta-29732-R | GCCGGCCATTAAAGCTAATA |
| 129 | PGSC0003DMG400009303 | Ta-9303-F | CGCGTTGCAACATTTGAATA |
| 130 | PGSC0003DMG400009303 | Ta-9303-R | GGGGGAGGAAGAAATTGAGA |
| 131 | PGSC0003DMG400002684 | Ta-2684-F | TACAGCCTGGAGATGGGATG |
| 132 | PGSC0003DMG400002684 | Ta-2684-R | TCGAGTGAGAATCGAGAGCA |
| 133 | PGSC0003DMG401020509 | Ta-1020509-F | CCCTTCCTTCATCGAGAACA |
| 134 | PGSC0003DMG401020509 | Ta-1020509-R | TGAAGCTAATCTGCGAACTCAA |
| 135 | PGSC0003DMG400033140 | Ta-33140-F | CAGGCCATGGAGATTTACCT |
| 136 | PGSC0003DMG400033140 | Ta-33140-R | TCCTTAAAAGGACCTCAGTGAA |
| 137 | PGSC0003DMG400025852 | Ta-25852-F | CGCGGTTACCCTGAATTTTA |
| 138 | PGSC0003DMG400025852 | Ta-25852-R | GCCCCGAACATAGCATACAT |
| 139 | PGSC0003DMG400026262 | Ta-26262-F | GCAGCCATTTTCTTCCTTCA |
| 140 | PGSC0003DMG400026262 | Ta-26262-R | TAGGGGAACATGGATTCGAG |
| 141 | PGSC0003DMG400026361 | Ta-26361-F | ATACTAAGCCACCCTTCGTCAC |
| 142 | PGSC0003DMG400026361 | Ta-26361-R | AACCATTATGAATGCAGCAGTG |
| 143 | PGSC0003DMG400028744 | Ta-28744-F | TCAAGCAGCAGCAGCAGTAG |
| 144 | PGSC0003DMG400028744 | Ta-28744-R | ATCACCTTGGCATTTTCCAT |
| 145 | PGSC0003DMG400015275 | Ta-15275-F | ATTCCATGAGAGCCACCTTG |
| 146 | PGSC0003DMG400015275 | Ta-15275-R | CTGGTCTGGGATCATCGTCT |
| 147 | PGSC0003DMG400015358 | Ta-15358-F | CACCGGAGAAAGAGAAGTCG |
| 148 | PGSC0003DMG400015358 | Ta-15358-R | CATAGGGAGATCAGGGTCCA |
| 149 | PGSC0003DMG400015407 | Ta-15407-F | TCGGTCTTCAGAAAGGATGAAT |
| 150 | PGSC0003DMG400015407 | Ta-15407-R | CAATTGAAGGCAACACATTGAC |
| 151 | PGSC0003DMG400015992 | Ta-15992-F | TGGAGAGTGGAGAAAGATCTGC |
| 152 | PGSC0003DMG400015992 | Ta-15992-R | AAGCACTGATTCTCAGGGATCT |
| 153 | PGSC0003DMG400015771 | Stub-15771-F | CCCCATCTACCCAAACTCCA |
| 154 | PGSC0003DMG400015771 | Stub-15771-R | GCATTTCCAGACCCATCAGC |
| 155 | PGSC0003DMG400027427 | Stub-27427-F | AACGGAGCAAGAACGTAGGG |
| 156 | PGSC0003DMG400027427 | Stub-27427-R | AAACCATTCCGTGGCCTCTT |
| 157 | PGSC0003DMG400009939 | Stub-9939-F | TGCCATCCCACTTCAGTTCC |
| 158 | PGSC0003DMG400009939 | Stub-9939-R | TCAAAGATCCGCTCAGCCAT |
| 159 | PGSC0003DMG400027423 | Stub-27423-F | TCAGGTCAACACTGCATCGG |
| 160 | PGSC0003DMG400027423 | Stub-27423-R | AGGATACCTCGGTGGGTTGA |
| 161 | PGSC0003DMG400022701 | Stub-22701-F | GTCGATACCTTTGCTGTTGCC |
| 162 | PGSC0003DMG400022701 | Stub-22701-R | CTTACAGAGTTCCGGGAGCA |
| 163 | PGSC0003DMG400016885 | Stub-16885-F | ACACTCAGGGCTTTCTCCAA |
| 164 | PGSC0003DMG400016885 | Stub-16885-R | GGCTCTCCCTATACCACCAG |
| 165 | PGSC0003DMG400014907 | Stub-14907-F | TCTTCCTCTGTCGTGGTGGA |
| 166 | PGSC0003DMG400014907 | Stub-14907-R | GCGCTTCAACACTCCGATTC |
| 167 | PGSC0003DMG400026095 | Stub-26095-F | ACACTGACTCGCCATCAAGA |
| 168 | PGSC0003DMG400026095 | Stub-26095-R | TGGAGGGTAGCACGTTCATT |
| 169 | PGSC0003DMG400046798 | Ta-46798-F | CGAGACTCCGATACCTGGAA |
| 170 | PGSC0003DMG400046798 | Ta-46798-R | GATAAATCCGCCATCTCTATCA |
| 171 | PGSC0003DMG400029727 | Ta-29727-F | GTCACCGGATTTTGACGTGT |
| 172 | PGSC0003DMG400029727 | Ta-29727-R | CTTCGCTTGCTTTCGCTAAT |
| 173 | PGSC0003DMG400002860 | Ta-2860-F | TTACCTTCTCCGCGATCTCT |
| 174 | PGSC0003DMG400002860 | Ta-2860-R | AGGCCAGTACTACAAGCCAAA |
| 175 | PGSC0003DMG400000187 | Ta–187–F | GGGATTGCTGCATCAGTTTT |
| 176 | PGSC0003DMG400000187 | Ta–187–R | CTTTCAGCAACTGGTTATACAG |
| 177 | PGSC0003DMG400003887 | Ta–3887–F | TCGATTGCATTGTGATGGAT |
| 178 | PGSC0003DMG400003887 | Ta–3887–R | GTGGCGTAATGTGTTTGTGC |
| 179 | PGSC0003DMG400023602 | Ta–23602–F | CGAGTTCTCTCTCAGTTCCAC |
| 180 | PGSC0003DMG400023602 | Ta–23602–R | CGGAACTCTGATTCGGAT |
| 181 | PGSC0003DMG400007693 | Ta–7693–F | TTTGTGGTTTGTTTGTGCAG |
| 182 | PGSC0003DMG400007693 | Ta–7693–R | AGCCCATGTAAATGCTGATT |
| 183 | PGSC0003DMG400025814 | Ta–25814–F | TGAGAGACAGGGCTTGGACT |
| 184 | PGSC0003DMG400025814 | Ta–25814–R | CTTTTTCGATGCCATTCACA |
| 185 | PGSC0003DMG400017560 | Ta–17560–F | CCGCCATTTCAGTTCGTAAT |
| 186 | PGSC0003DMG400017560 | Ta–17560–R | AACCCAATTCCGTGACTCTG |
| 187 | PGSC0003DMG400006913 | Ta–6913–F | GTGGTGCTTGGAAGCTCTGT |
| 188 | PGSC0003DMG400006913 | Ta–6913–R | GGAATTCTGTGGCTTTGTGA |
| 189 | PGSC0003DMG400000545 | Ta–545–F | GAACGGTTATTTGGATGGTAAA |
| 190 | PGSC0003DMG400000545 | Ta–545–R | CAAGCATGTTCCATAGCTCA |
| 191 | PGSC0003DMG400030905 | Ta–30905–F | GAGATGAACATGATCACAAAGGTTG |
| 192 | PGSC0003DMG400030905 | Ta–30905–R | TGAAGAAGCAGTTCCAGCAA |
| 193 | PGSC0003DMG400011842 | Ta–11842–F | GTAAGCGAATGGAGGGTTGA |
| 194 | PGSC0003DMG400011842 | Ta–11842–R | TGTCATCAAAGCCAATCCAA |
| 195 | PGSC0003DMG400019872 | Ta–19872–F | GCAAGGTTTTCTCCCTTTACC |
| 196 | PGSC0003DMG400019872 | Ta–19872–R | CCAAAGTAACGTGCCGAGTT |
| 197 | PGSC0003DMG400001630 | F_cyclofilin | CTCTTCGCCGATACCACTCC |
| 198 | PGSC0003DMG400001630 | R_cyclofilin | TCACACGGTGGAAGGTTGAG |
| 199 | PGSC0003DMG400003531 | Dhn1(RAB18) | GTAATCTGAGGATGATGG |
| 200 | PGSC0003DMG400003531 | Dhn1(RAB18) | GGGAGCTTCTCTTTGATCTT |

(B)

| **ID** | **target gene** | **primer name** | **Primer sequence (5'-3')** |
| --- | --- | --- | --- |
| 1 | AT5G55090 | long_AT5G55090F | ACCAATCATAGGTCGAGGCT |
| 2 | AT5G55090 | long_AT5G55090R | AGCTGTCTTGTTGATCCCAGA |
| 3 | AT1G22170 | long_AT1G22170F | TCTGTCTCTCTTGTCGTAGTGA |
| 4 | AT1G22170 | long_AT1G22170R | AACAGTGGTGGTTATGACACA |
| 5 | AT3G18280 | long_AT3G18280F | TGGTGATGATCAAGACCACAA |
| 6 | AT3G18280 | long_AT3G18280R | CAAAGCATCACCAAGAGTACCA |
| 7 | AT5G14570 | long_AT5G14570F | TGATTGAGCATTTGGGTTTGAA |
| 8 | AT5G14570 | long_AT5G14570R | TGCAATGTTGTCAGCTCTAGTG |
| 9 | AT5G09640 | long_AT5G09640F | TGAGAAACCTTAGCTTCATCGTC |
| 10 | AT5G09640 | long_AT5G09640R | ATACTCCGCTGTGTGTCCTC |
| 11 | AT4G16490 | long_AT4G16490F | AGCCTCCCTCCTCCTCTTTA |
| 12 | AT4G16490 | long_AT4G16490R | CACCCTCACAAACCAGAAGC |
| 13 | AT3G20660 | long_AT3G20660F | GACAACCGCTGCTAGAGAAA |
| 14 | AT3G20660 | long_AT3G20660R | TCGTGCATCCCAAACATTGT |
| 15 | AT1G13440 | GAPDH5'_F | TCTCGATCTCAATTTCGCAAAA |
| 16 | AT1G13440 | GAPDH5'_R | CGAAACCGTTGATTCCGATTC |

(C)

| **ID** | **target gene** | **primer name** | **Primer sequence (5'-3')** |
| --- | --- | --- | --- |
| 1 | AT5G55090 | SALK_047537LP | ATTATGTGCCATTCGACCAAC |
| 2 | AT5G55090 | SALK_047537RP | TTTAGCACAACCCAAATCGAC |
| 3 | AT1G22170 | SALK_087895LP | AAGGCTTTGAAGAGGTGCTTC |
| 4 | AT1G22170 | SALK_087895RP | GGGATTGTCTTGGAAGGAGAC |
| 5 | AT3G20660 | SALK_095906LP | GTAGATGAGGCGATGAAGCTG |
| 6 | AT3G20660 | SALK_095906RP | CCTCTCCTGTTGCTGAAGATG |
| 7 | AT5G14570 | SALK_118221LP | AAAGGAGACACATTGCGAGTG |
| 8 | AT5G14570 | SALK_118221RP | GAATCGCTGAGGAAGTACGTG |
| 9 | AT5G09640 | SALK_018120LP | CTGGAGTATACTCCGCTGTGTG |
| 10 | AT5G09640 | SALK_018120RP | TGTGGAGGCAAATTCTTCAAC |
| 11 | AT4G16490 | SALK_054063LP | CGATTCACTGGAAAAATCACG |
| 12 | AT4G16490 | SALK_054063RP | GAAACTCCGACGCATACTCTG |
| 13 | AT3G18280 | GK-310G07_T-DNA | ATAATAACGCTGCGGACATCTACATTTT |
| 14 | AT3G18280 | GK-310G07_Locus-spec | GCATACAAAGCATCACCAAGAGTA |
| 15 | AT3G18280 | GK-310G07_F | TTTTGAATACTTTCCCCAATGTTA |
| 16 | AT3G18280 | GK-310G07_R | TTTCTGACTTTTTAACACCTTGGG |

**Table S4 Summary of RNA-seq Data.**

| **Sample Name** | **Library tag** | **Number of**  **clean reads** | **Number of mapped reads** | **Percentage of mapped reads** | **Number of reads mapped in single loci** | **Percentage of reads mapped in single loci** | **Number of reads mapped in multiple loci** | **Percentage of reads mapped in multiple loci** |
| --- | --- | --- | --- | --- | --- | --- | --- | --- |
| **1** | Gwiazda 1 D0 | 40822276 | 33318248 | 81.6% | 31529045 | 94.6% | 1789203 | 5.4% |
| **2** | Gwiazda 2 D0 | 40735106 | 32957788 | 80.9% | 30973115 | 94.0% | 1984673 | 6.0% |
| **3** | Gwiazda 3 D0 | 40142054 | 32431488 | 80.8% | 29962588 | 92.4% | 2468900 | 7.6% |
| **4** | Gwiazda 1 D6 | 40583272 | 32274433 | 79.5% | 30116970 | 93.3% | 2157463 | 6.7% |
| **5** | Gwiazda 2 D6 | 40865444 | 34258402 | 83.8% | 27490880 | 80.2% | 6767522 | 19.8% |
| **6** | Gwiazda 3 D6 | 40778350 | 33074580 | 81.1% | 31360101 | 94.8% | 1714479 | 5.2% |
| **7** | Gwiazda 1 D10 | 40934706 | 32897961 | 80.4% | 30962516 | 94.1% | 1935445 | 5.9% |
| **8** | Gwiazda 2 D10 | 41216874 | 33480807 | 81.2% | 31531097 | 94.2% | 1949710 | 5.8% |
| **9** | Gwiazda 3 D10 | 40439124 | 31631516 | 78.2% | 29859191 | 94.4% | 1772325 | 5.6% |
| **10** | Oberon 1 D0 | 40352776 | 30194035 | 74.8% | 28108164 | 93.1% | 2085871 | 6.9% |
| **11** | Oberon 2 D0 | 40106264 | 29673762 | 74.0% | 28001544 | 94.4% | 1672218 | 5.6% |
| **12** | Oberon 3 D0 | 41268334 | 28804012 | 69.8% | 27139166 | 94.2% | 1664846 | 5.8% |
| **13** | Oberon 1 D6 | 40713850 | 29947373 | 73.6% | 28388428 | 94.8% | 1558945 | 5.2% |
| **14** | Oberon 2 D6 | 43232122 | 29333650 | 67.9% | 27475362 | 93.7% | 1858288 | 6.3% |
| **15** | Oberon 3 D6 | 42471276 | 31485894 | 74.1% | 29494569 | 93.7% | 1991325 | 6.3% |
| **16** | Oberon 1 D10 | 41344780 | 31933380 | 77.2% | 29952250 | 93.8% | 1981130 | 6.2% |
| **17** | Oberon 2 D10 | 43839554 | 31029666 | 70,8% | 29333777 | 94.5% | 1695889 | 5,5% |
| **18** | Oberon 3 D10 | 41441140 | 32307340 | 78.0% | 30318497 | 93.8% | 1988843 | 6.2% |
| **19** | Tajfun 1 D0 | 41364728 | 34291602 | 82.9% | 31729028 | 92.5% | 2562574 | 7.5% |
| **20** | Tajfun 2 D0 | 41058638 | 33320141 | 81.2% | 30947172 | 92.9% | 2372969 | 7.1% |
| **21** | Tajfun 3 D0 | 41242518 | 34107225 | 82.7% | 31999823 | 93.8% | 2107402 | 6.2% |
| **22** | Tajfun 1 D6 | 41371432 | 33647319 | 81.3% | 31361719 | 93.2% | 2285600 | 6.8% |
| **23** | Tajfun 2 D6 | 41461212 | 34178187 | 82.4% | 32060279 | 93.8% | 2117908 | 6.2% |
| **24** | Tajfun 3 D6 | 41110642 | 33536868 | 81.6% | 30410251 | 90.7% | 3126617 | 9.3% |
| **25** | Tajfun 1 D10 | 41273690 | 33113990 | 80.2% | 30930714 | 93.4% | 2183276 | 6.6% |
| **26** | Tajfun 2 D10 | 41611500 | 33153411 | 79.7% | 31005890 | 93.5% | 2147521 | 6.5% |
| **27** | Tajfun 3 D10 | 39723298 | 31196763 | 74.3% | 29157061 | 93.5% | 2039702 | 6.5% |
| **28** | Owacja 1 D0 | 40563894 | 31963920 | 78.8% | 29033317 | 90.8% | 2930603 | 9.2% |
| **29** | Owacja 2 D0 | 41635240 | 34289170 | 82.4% | 32000829 | 93.3% | 2288341 | 6.7% |
| **30** | Owacja 3 D0 | 40754872 | 32156498 | 78.9% | 29479821 | 91.7% | 2676677 | 8.3% |
| **31** | Owacja 1 D6 | 41218562 | 32910849 | 79.8% | 30827730 | 93.7% | 2083119 | 6.3% |
| **32** | Owacja 2 D6 | 41386880 | 33310961 | 80.5% | 31444404 | 94.4% | 1866557 | 5.6% |
| **33** | Owacja 3 D6 | 41262192 | 32927942 | 79.8% | 30892719 | 93.8% | 2035223 | 6.2% |
| **34** | Owacja 1 D10 | 41224220 | 32655324 | 79.2% | 30632962 | 93.8% | 2022362 | 6.2% |
| **35** | Owacja 2 D10 | 40920912 | 31928719 | 78.0% | 29918534 | 93.7% | 2010185 | 6.3% |
| **36** | Owacja 3 D10 | 41275634 | 29178157 | 70.7% | 26165846 | 89.7% | 3012311 | 10.3% |
