# Supplementary material for: Genomewide identification of genes involved in the potato response to drought indicates functional evolutionary conservation with Arabidopsis plants
Source: Plant Biotechnol J. 2017 Aug 14;16(2):603–14. doi: 10.1111/pbi.12800 (PMC5787840; doi:10.1111/pbi.12800)

Table S1 The comparison of normalized number of reads for transcripts derived from genes searched out in the first round of selection (594 genes) during the time course of drought experiment. The gray color indicates the selected genes after the second round of selection.

Mean value of normalised reads per million for selected potato transcripts that were up-regulated in Tajfun comparing to Owacja. Data are shown for days: 0, 6, and 10 of drought experiment and were taken from three biological replicates. SD at D0 was always not statisticly significant (*P* >0,05), while SD at D6 and D10 were always statisticly significant (*P* < 0,05, see also Fig 5). Potato gene accesion numbers were taken from Spud DB (www.potato.plantbiology.msu.edu). SD - standard deviations.

Gene accesion numbers	Tajfun reads mean value			Owacja reads mean value			Tajfun SD			Owacja SD		
	D0	D6	D10	D0	D6	D10	D0	D6	D10	D0	D6	D10
**PGSC0003DMG400000088**	928	5532	3208,67	708	3808	1269,3	365,58	631,15	423,36	213,38	446,34	399,55
**PGSC0003DMG400000089**	65,33	1929,3	1391,33	50	2632,7	2072	36,02	15,53	215,22	18,33	212,74	391,45
**PGSC0003DMG400000101**	172,33	776	6532	178	1633,7	28693	77,57	90	4383,5	53,33	283,86	1723,21
**PGSC0003DMG400000337**	254	549,33	713,33	166,67	338,67	360,67	32	84,32	184	73,66	55	117,19
**PGSC0003DMG400000350**	1184,67	4323,3	2707,33	1087,33	2451,3	1037,3	240,8	399,8	321,06	303,53	594,89	72,73

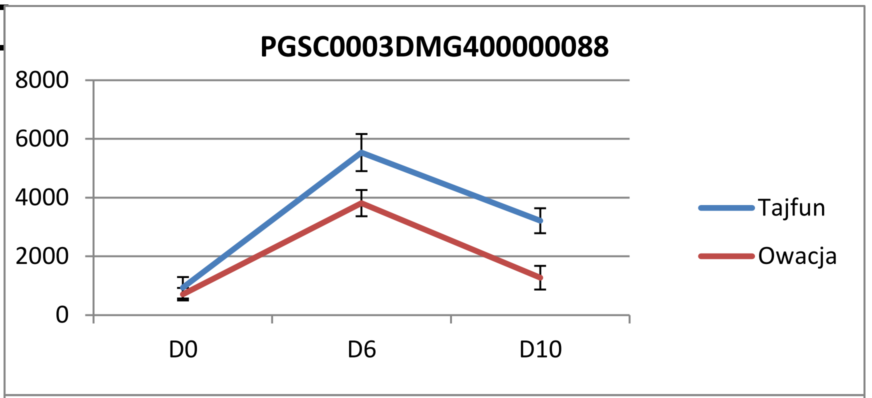

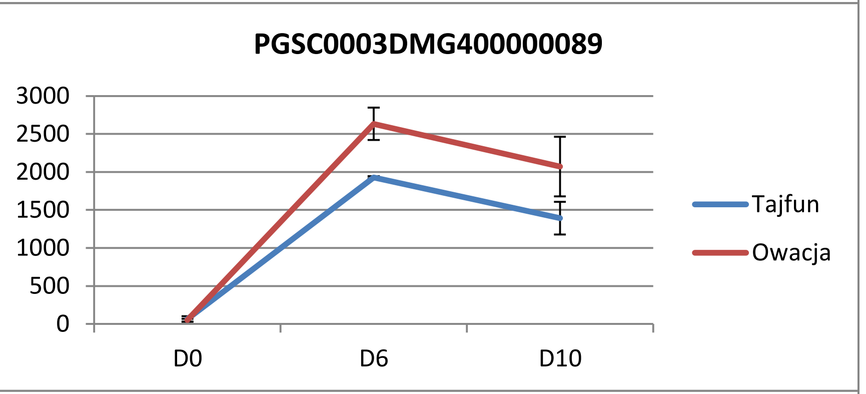

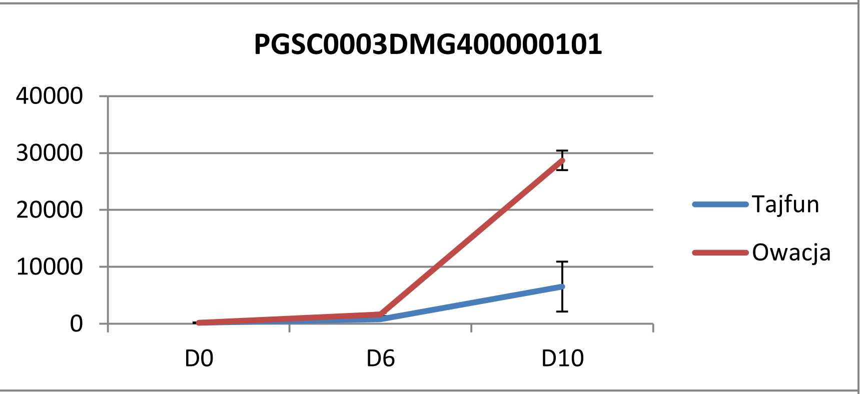

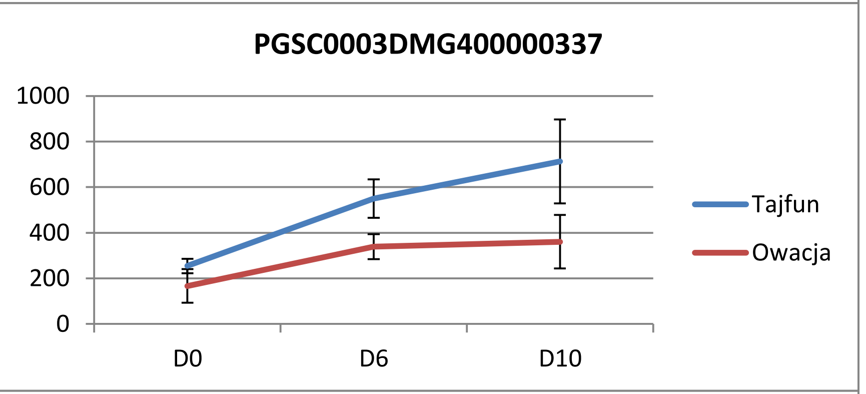

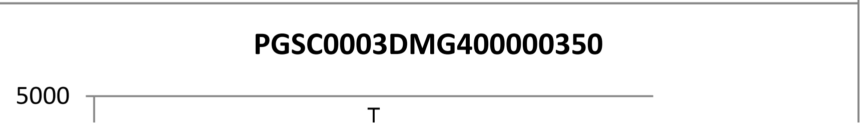

**PGSC0003DMG400000396**	1036,67	3428,7	3790	780	5538,7	7976	189,37	377,33	1105,1	143,93	447,52	330,68
**PGSC0003DMG400000411**	104,67	1057,3	3540	93,33	1700	4994,7	17,47	235,14	1091,3	18,04	55,43	548,92
**PGSC0003DMG400000457**	2879,33	5918,7	14667,3	2607,33	9888,7	22069	536,14	665,01	1628	1005,1	442,41	3080,08
**PGSC0003DMG400000618**	3916,67	10609	13733,3	4740	6800	7520,7	622,67	1298,7	1101,1	1214,5	938,27	565,34
**PGSC0003DMG400000654**	519,33	1518,7	1556,67	378	764,67	442	169,9	250,71	333,24	45,21	118,28	149,01

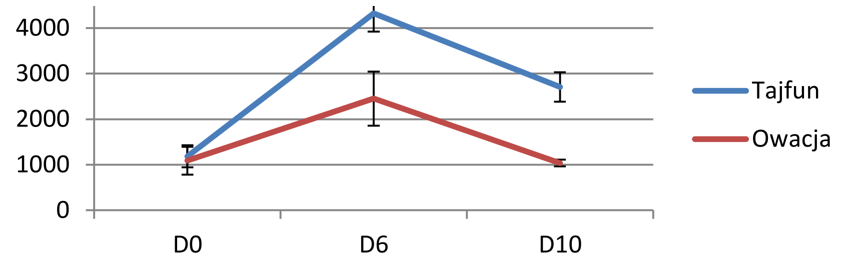

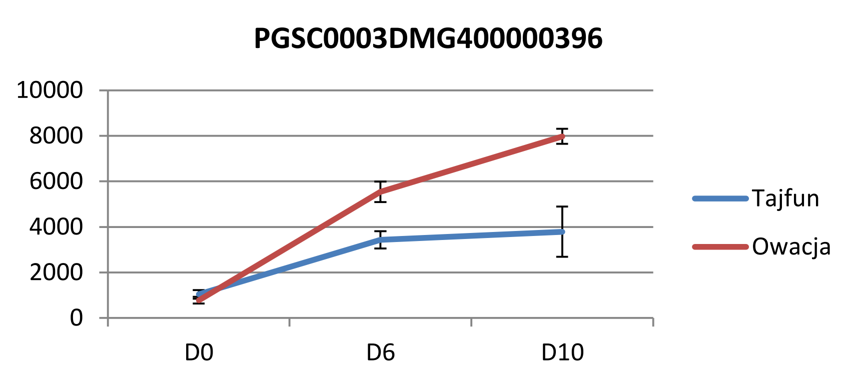

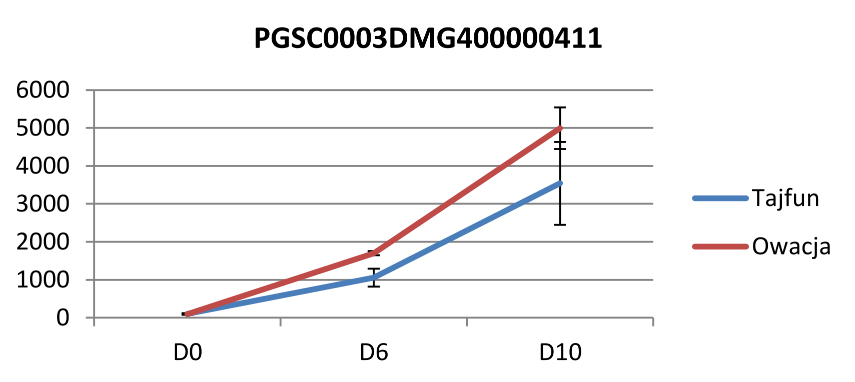

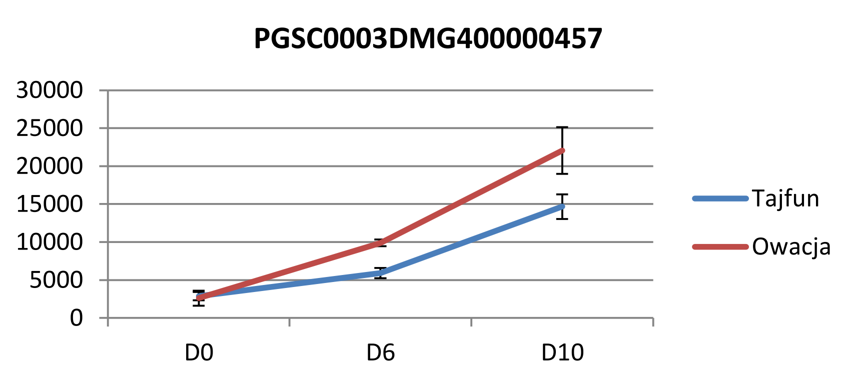

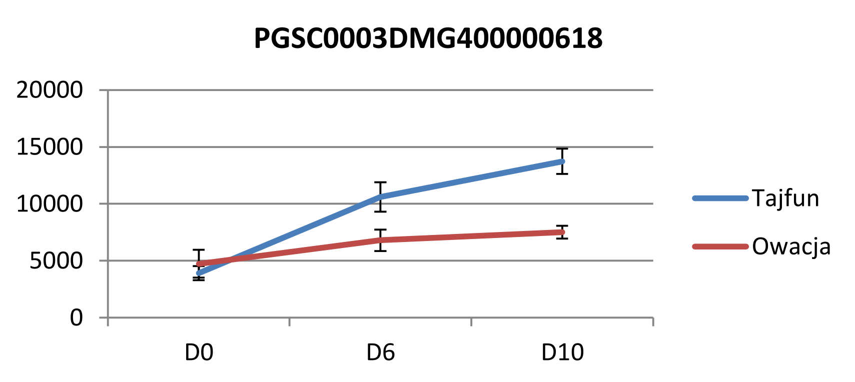

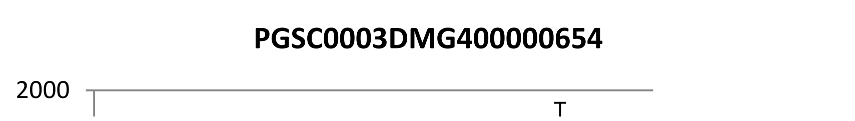

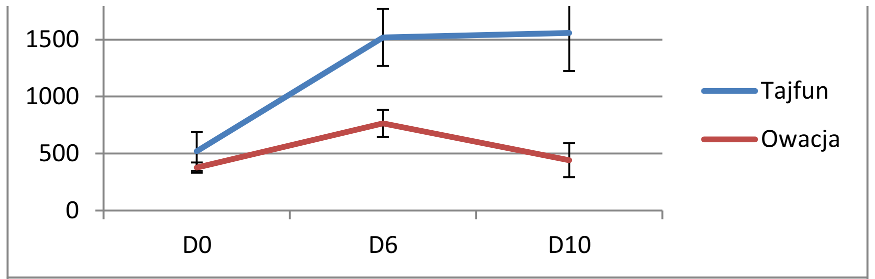

**PGSC0003DMG400000758**	3,33	265,33	242,67	9,33	17,33	56	5,77	40,22	62,01	11,37	15,01	17,09
**PGSC0003DMG400000839**	85,33	353,33	306,67	53,33	144	42	52,62	83,58	56,62	26,1	22,27	7,21
**PGSC0003DMG400000944**	655	1667	2442,33	445	1120,7	826	205,01	309,4	440,85	73,67	316,96	239,76
**PGSC0003DMG400000978**	822,67	3087,3	4250	1077,33	4352,7	5794	24,19	67,09	1085,1	142,44	43,92	410,13
**PGSC0003DMG400000999**	115,33	508	422	71,33	303,33	188,67	23,18	100,54	161,98	39,72	21,57	10,26

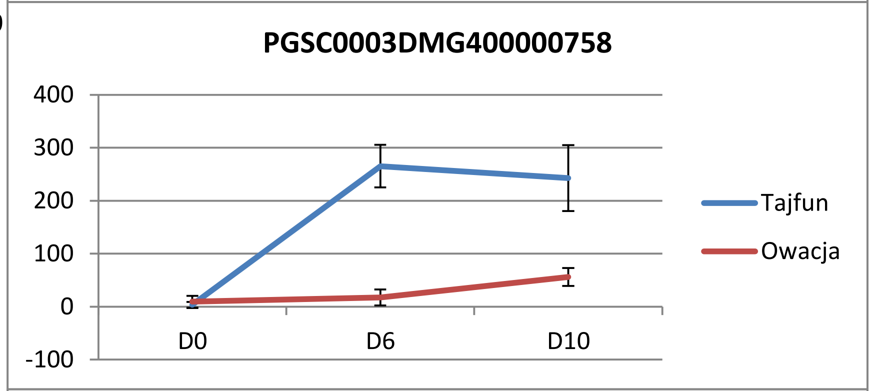

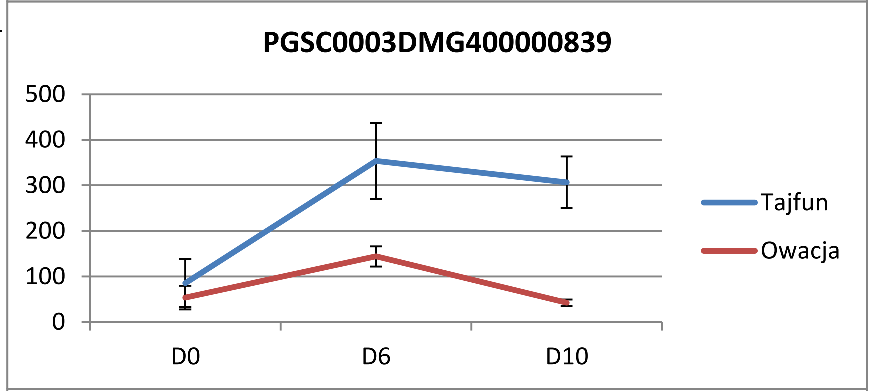

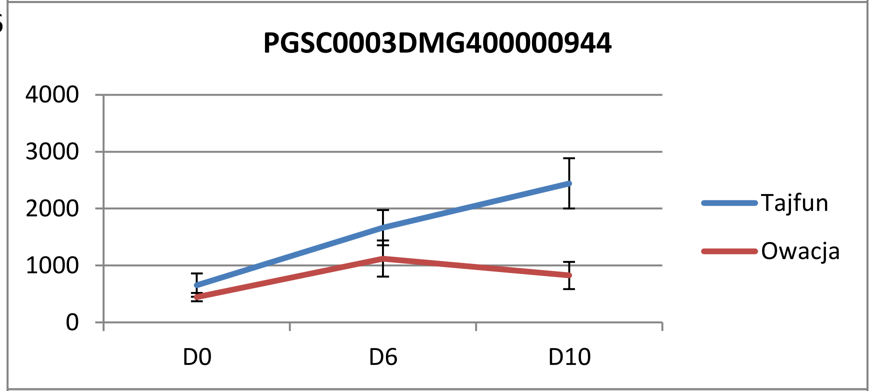

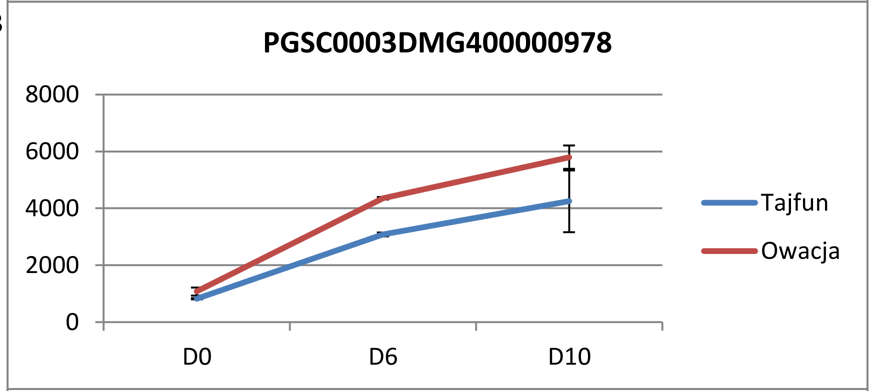

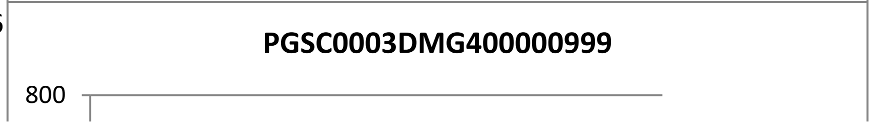

**PGSC0003DMG400001152**	1153,33	2399,3	2892	815,33	1418,7	1400,7	172,1	142,57	435,57	95,09	312,26	224,5
**PGSC0003DMG400001474**	640,67	2635,3	2451,33	862	3864,7	4086	89,49	300,95	568,46	235,25	650,11	314,69
**PGSC0003DMG400001506**	663,33	2152,7	2500	564,67	1356,7	1203,3	142	91,53	322,3	92,61	368,92	327,44
**PGSC0003DMG400001770**	138	421,33	310,67	78	195,33	133,33	54,44	125,93	27,3	39,34	8,33	9,87
**PGSC0003DMG400001771**	112	459,33	504	76,67	221,33	205,33	28,35	32,88	88,48	18,58	83	61,33

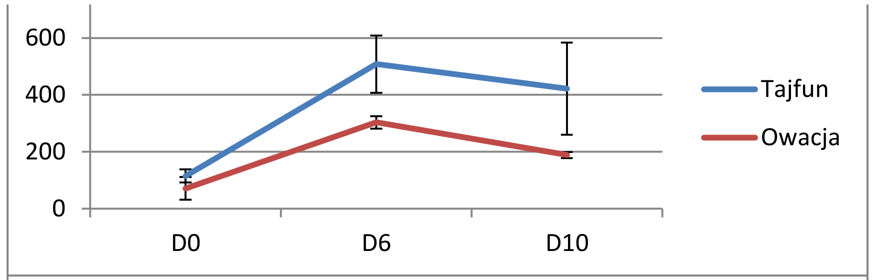

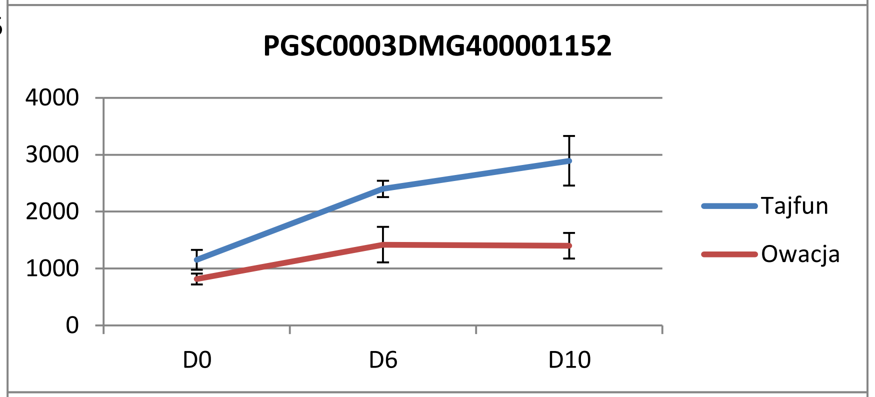

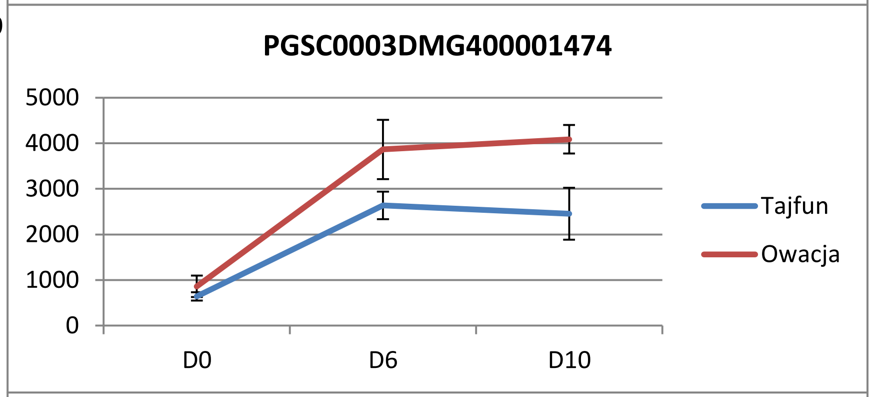

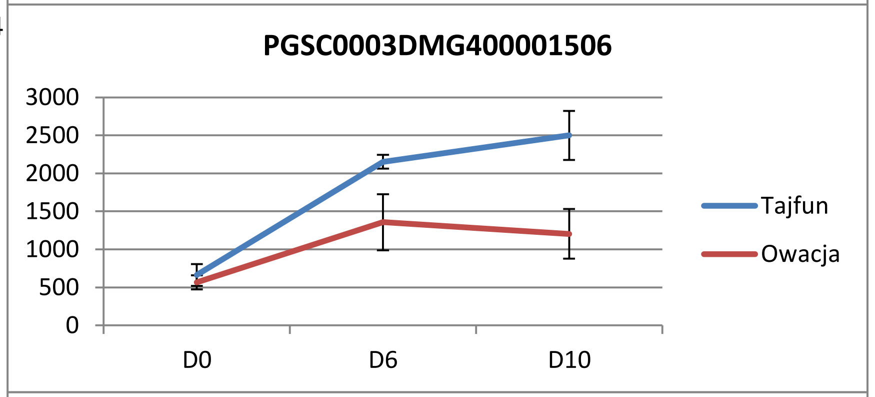

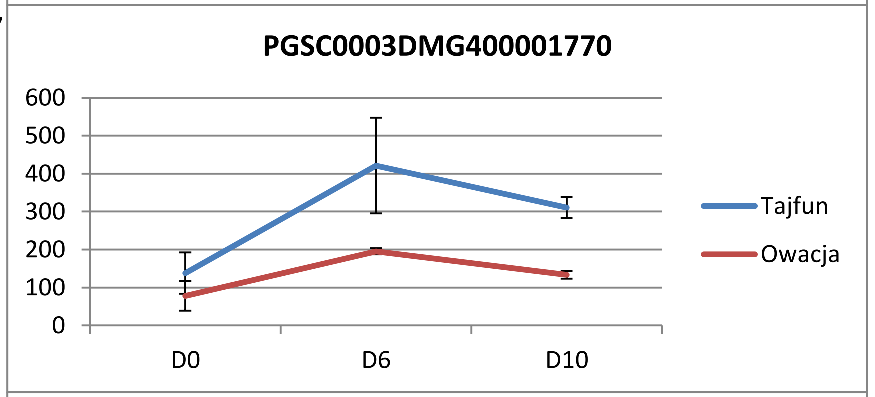

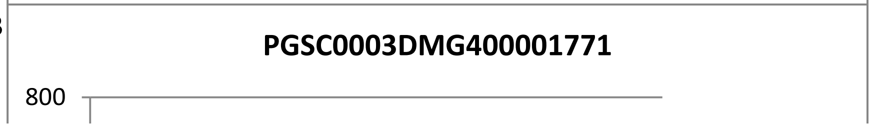

**PGSC0003DMG400001788**	274	934,67	654,67	177,33	391,33	228	85,58	103	128,78	74,63	94,96	27,78
**PGSC0003DMG400002489**	106	270,67	242,67	93,33	397,33	360,67	6	14,05	28,59	33,31	78,55	36,95
**PGSC0003DMG400002585**	140,67	364,67	351,33	109,33	209,33	130,67	18,04	37	81,4	11,72	5,03	32,58
**PGSC0003DMG400002613**	1840,67	7063,3	6537,33	1867,33	13165	9333,3	591	1841,1	332,62	277,48	691,62	3071,99
**PGSC0003DMG400002684**	3008	6083,3	7408,67	2821,33	3648	3002,7	767	558,32	1586,8	153,31	360,41	243,07

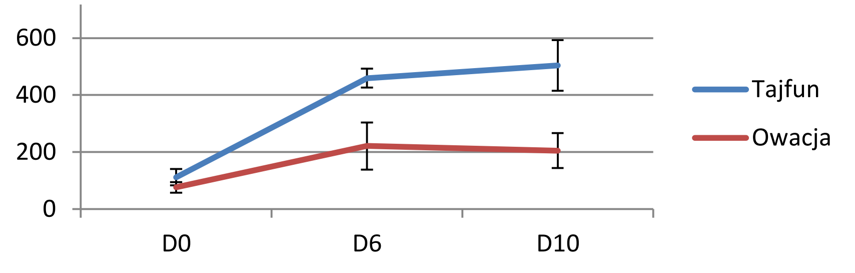

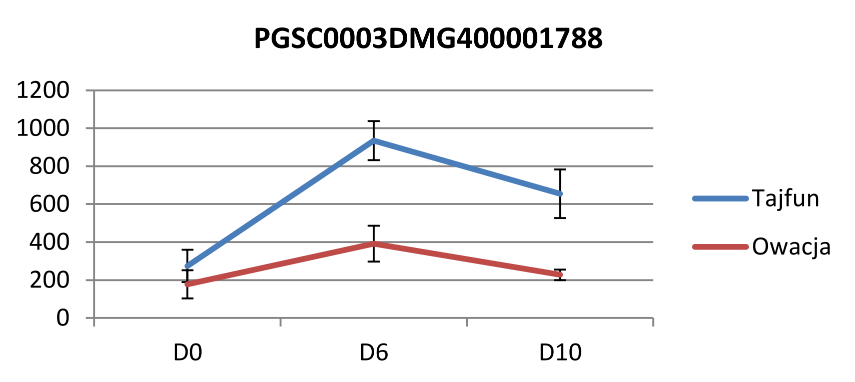

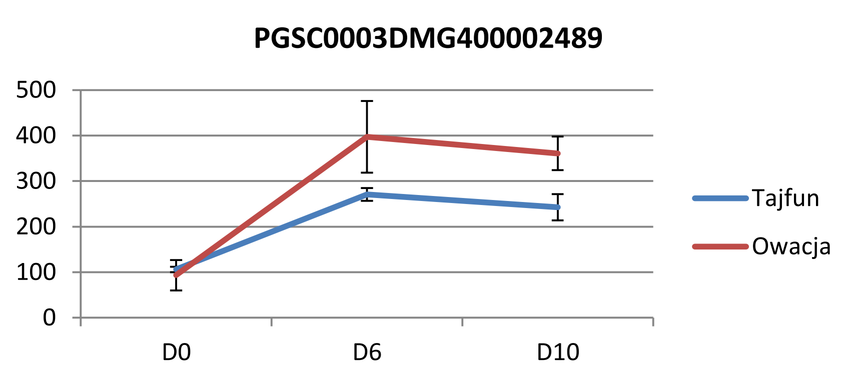

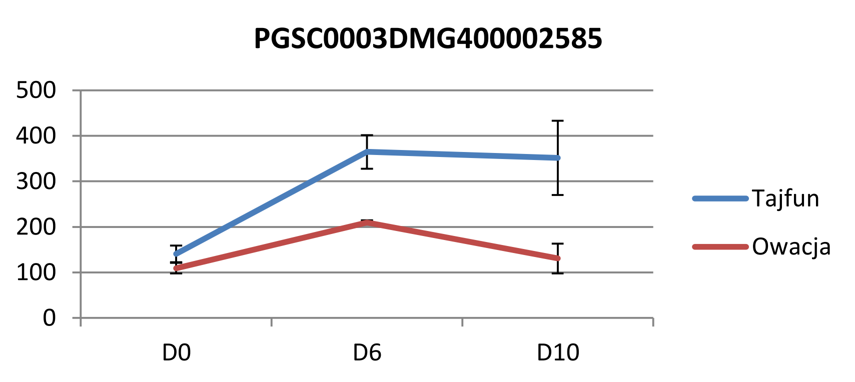

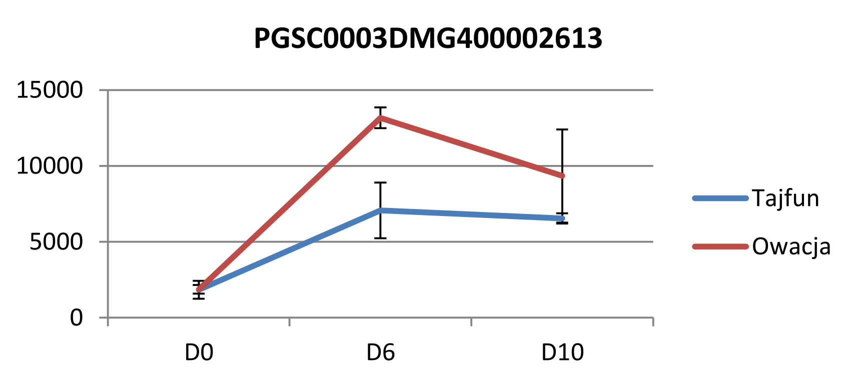

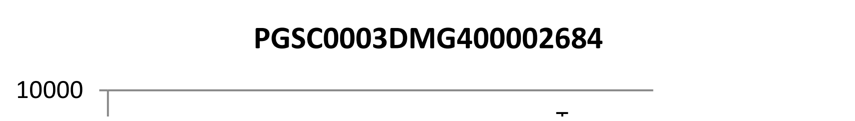

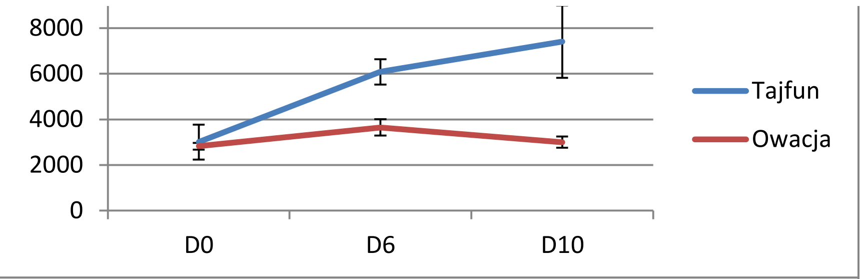

**PGSC0003DMG400002746**	498	1108,7	1351,33	324,67	621,33	518,67	215,03	196,67	196,57	64,29	143,42	85,54
**PGSC0003DMG400002845**	958	2300	2151,33	1244	3130	3969,3	317,21	172,31	364,93	369,04	189,07	477,42
**PGSC0003DMG400002860**	1671,33	3322	4222,67	1846,67	2443,3	2576,7	265,1	428,53	593,03	311,75	459,58	238,01
**PGSC0003DMG400002929**	7952,67	17227	64494	7414,33	10979	13005	584,11	1245,2	14591	867,7	552,18	3124,03
**PGSC0003DMG400003048**	3673,33	8775,3	9390,33	3430,67	12035	12483	522,68	952,49	1888,5	589,62	1089,8	1463,31

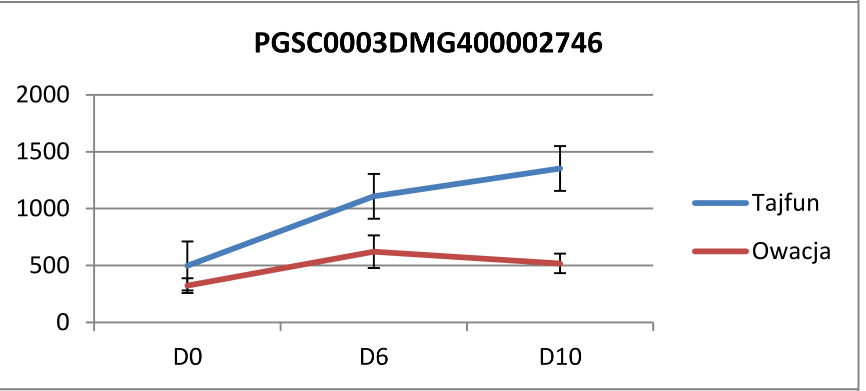

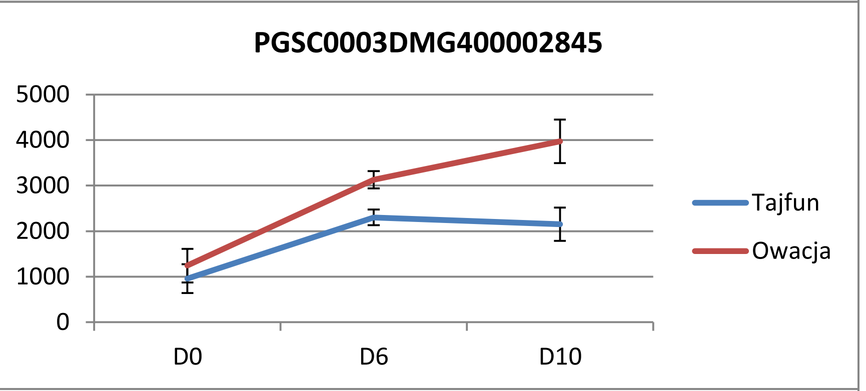

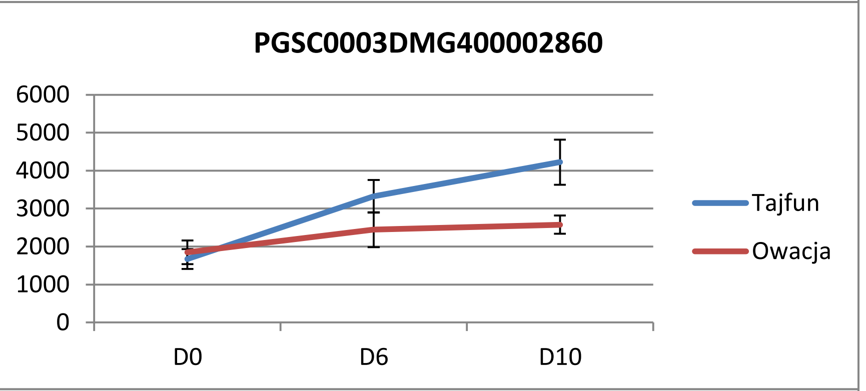

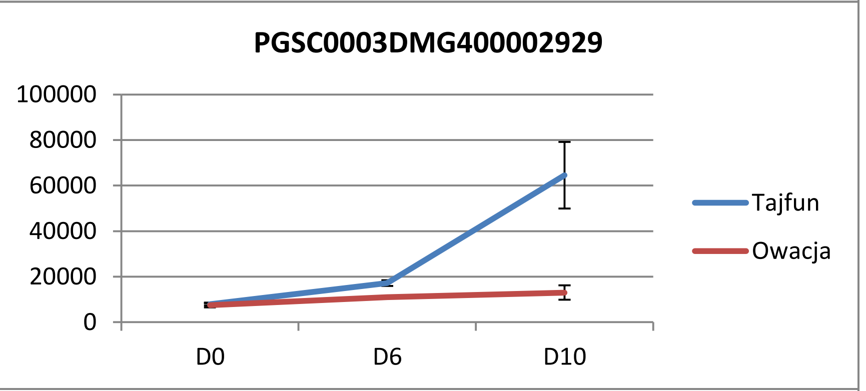

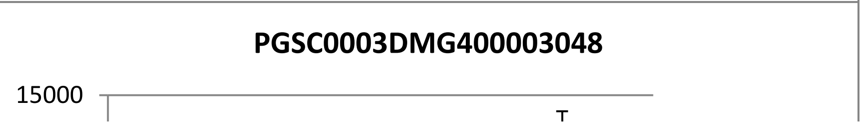

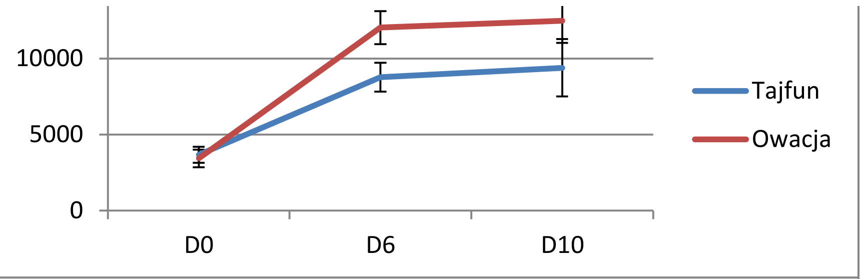

**PGSC0003DMG400003051**	1232,67	2597,3	2486,67	1067,33	1815	984,67	240,01	239,45	397,14	73,28	141,9	134,6
**PGSC0003DMG400003083**	3986	40014	37721,3	3356,67	52951	22857	194,08	1304,5	8160,7	1562,8	10787	837,07
**PGSC0003DMG400003528**	336,67	978	3186,67	413,33	1364	4910,7	126,62	35,38	1342,2	130,73	288,17	695,11
**PGSC0003DMG400003649**	44,67	1286,7	1564,67	14	793,33	250,67	32,15	285,41	589	10,39	121,92	46,06
**PGSC0003DMG400003688**	86	742	825,33	131,33	340	306	73,51	273,27	144,42	32,58	15,87	35,16

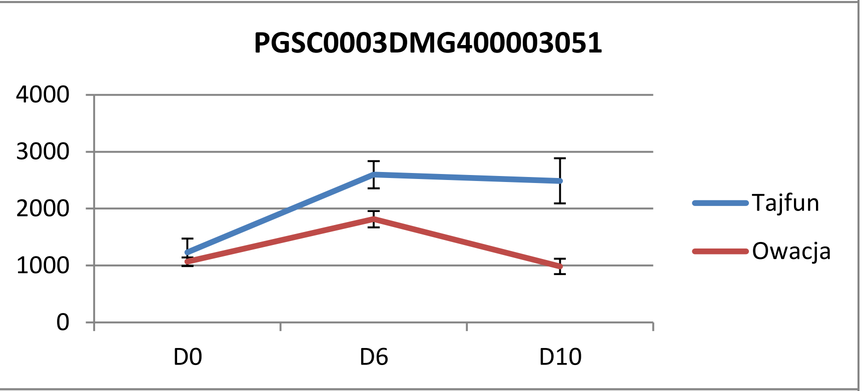

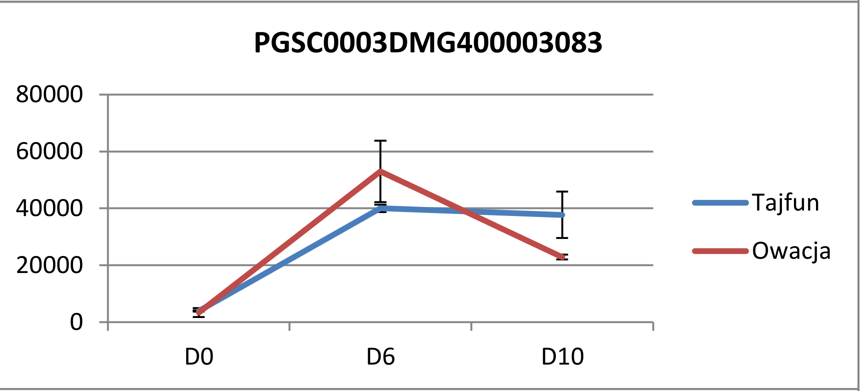

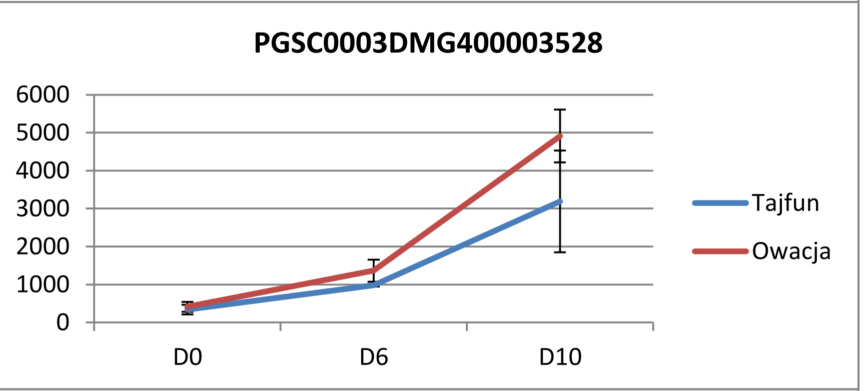

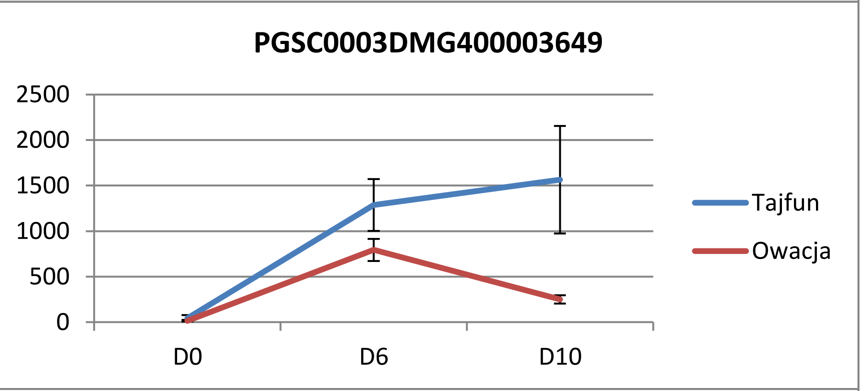

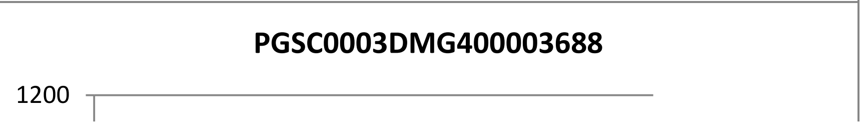

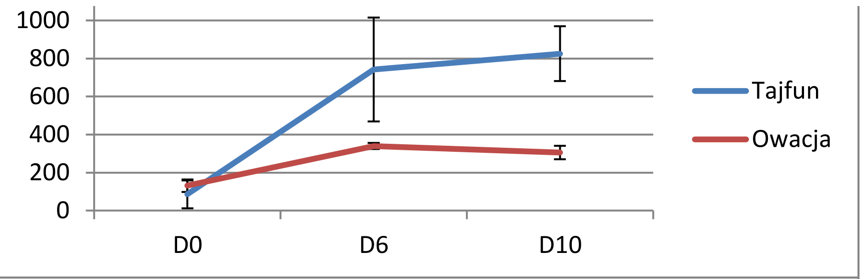

**PGSC0003DMG400004010**	23,33	589,33	476	20	273,33	127,67	40,41	185,32	170,13	17,32	99,38	36,09	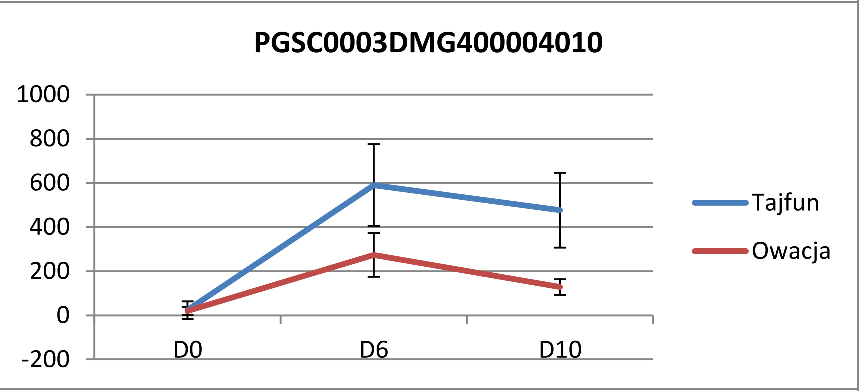
**PGSC0003DMG400004029**	10	195,33	13669,3	44,67	1944,7	43965	11,14	98,48	14673	43,1	484,93	5231,87	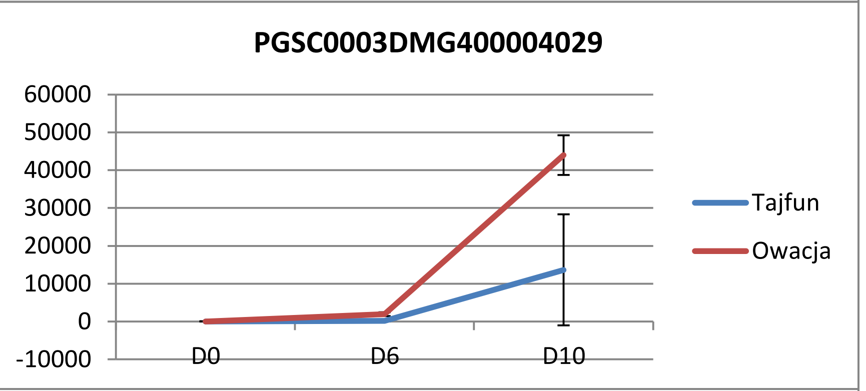
**PGSC0003DMG400004233**	112,67	390,67	326,67	152,67	1630	2116,7	108,3	78,42	103,58	75,96	281,05	300,88	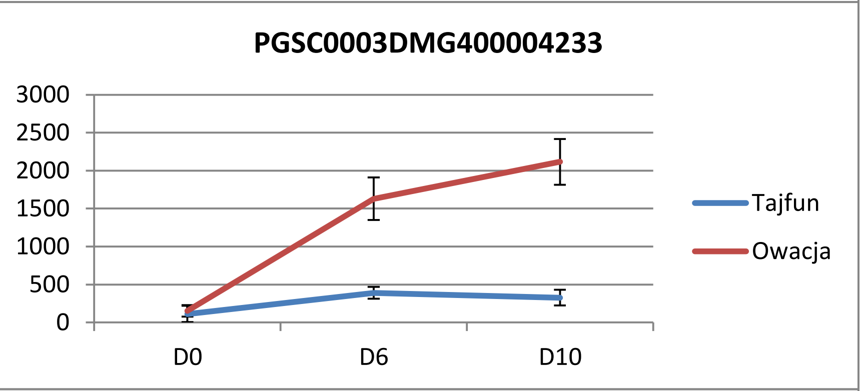
**PGSC0003DMG400004286**	150	456	641,33	78,67	273,33	312,67	44,23	5,29	104,64	55,51	24,68	111,79	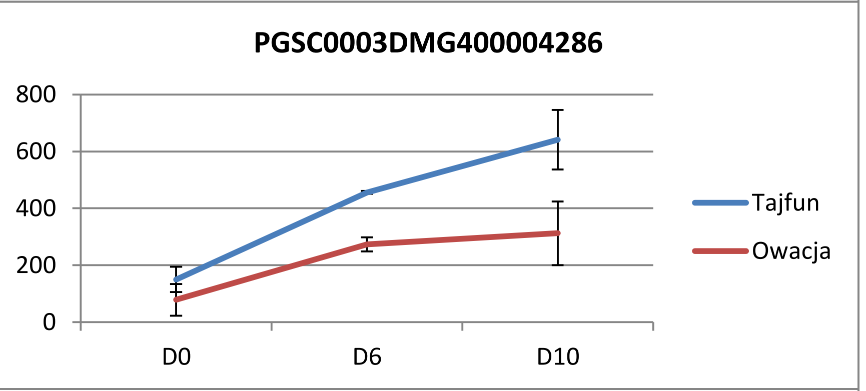
**PGSC0003DMG400004532**	2,67	27,33	217,33	16	72	580	3,06	20,03	182,49	9,17	18,33	227,17	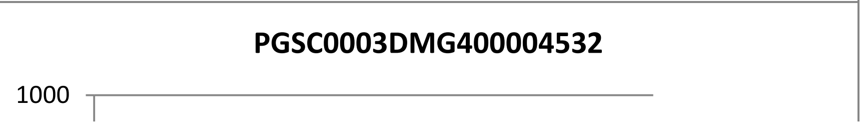

**PGSC0003DMG400004630**	2255,33	12929	30312	1720,67	19443	52417	583,85	990,31	8871,9	585,53	3018,6	3330,37
**PGSC0003DMG400004802**	1780	4617,3	7805,33	1567,33	6752,7	11383	159,76	1210,4	3551,2	667,79	557,43	679,42
**PGSC0003DMG400005009**	72	342,67	266	22	178	126	45,3	47,51	77,95	19,08	77,25	6,93
**PGSC0003DMG400005056**	260	513,33	2052,67	173,67	828,67	4394	143,37	63,29	752,87	67,28	67,42	1581,64
**PGSC0003DMG400005247**	976	1874	1952	1096,67	1339,3	1076,7	66,84	211,4	45,21	136,27	132,02	175,78

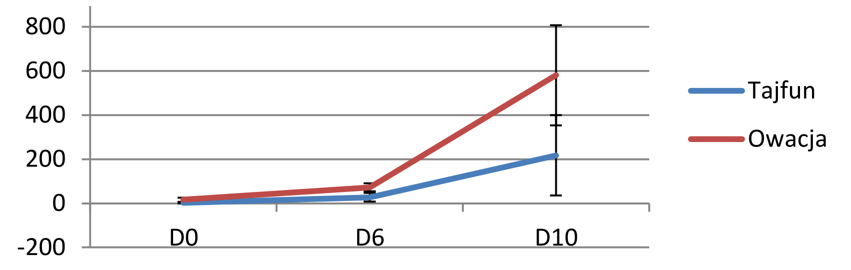

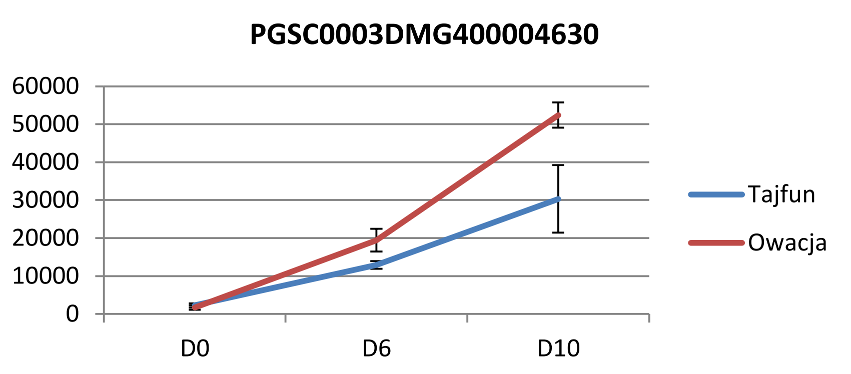

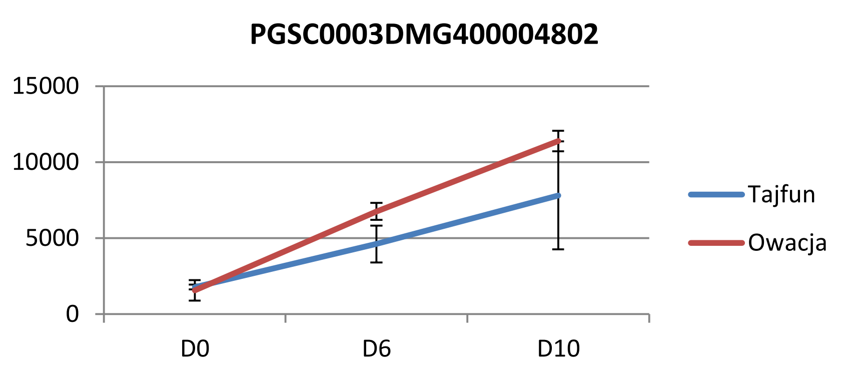

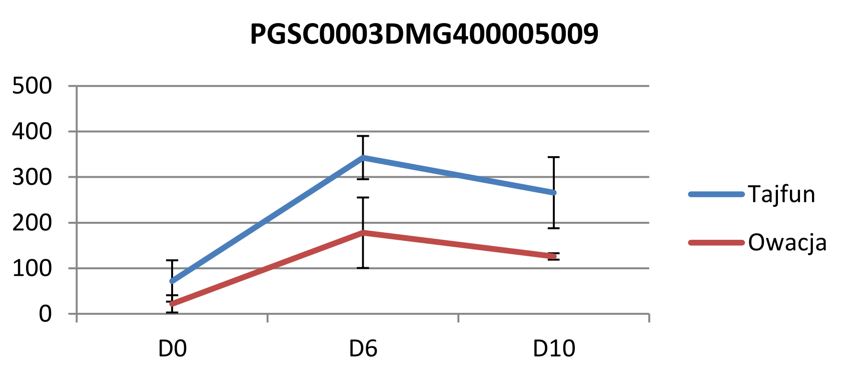

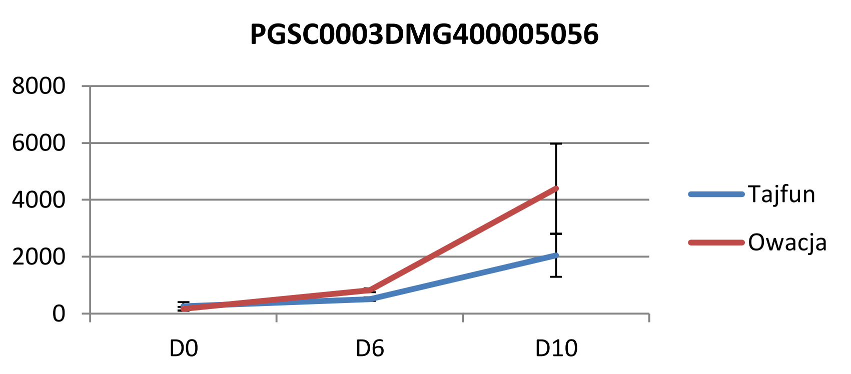

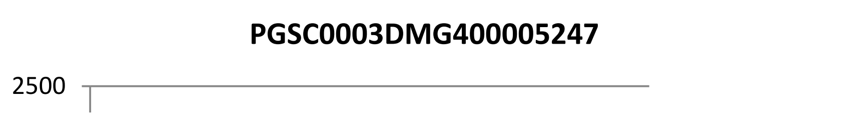

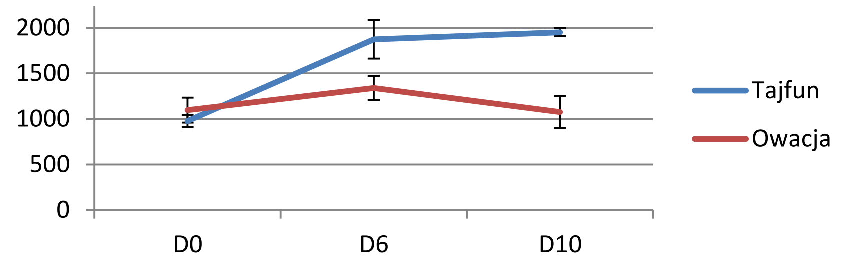

**PGSC0003DMG400005731**	1774	7064,7	5801,33	1082	1407,3	1202,7	207,18	1313,2	1218,1	378,48	303,16	356,23
**PGSC0003DMG400005738**	1267,33	7583,3	6865,67	1076,67	5153,3	3842	143,34	307,56	227,5	403,39	926,51	397,14
**PGSC0003DMG400005917**	39,33	164,67	146,67	41,33	84,67	28,67	9,45	31,01	56,86	13,01	43	12,22
**PGSC0003DMG400006221**	172,67	605,33	1731,33	190,67	899,33	2574,7	18,58	43,1	952	31,9	46,92	100,03
**PGSC0003DMG400006295**	276,67	692	810,67	170,67	427,33	438	28,31	160,34	178,58	72,92	86,03	108,06

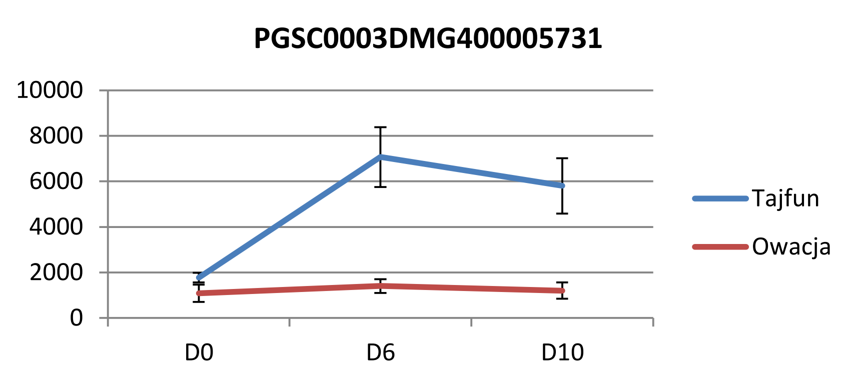

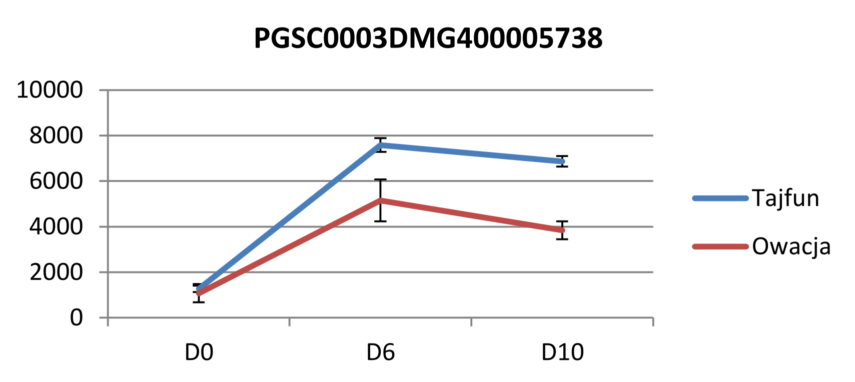

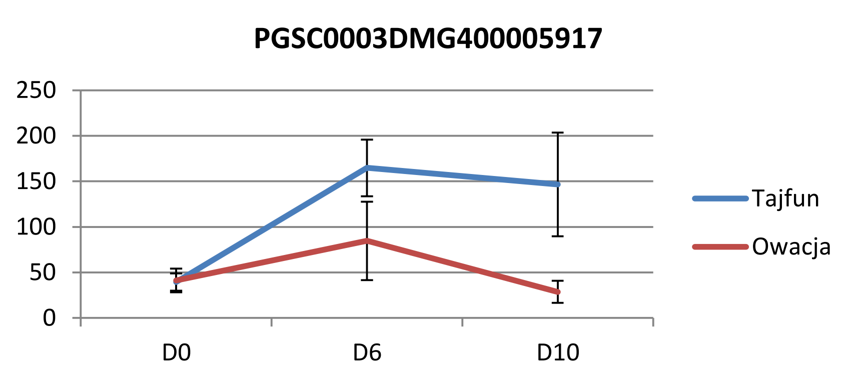

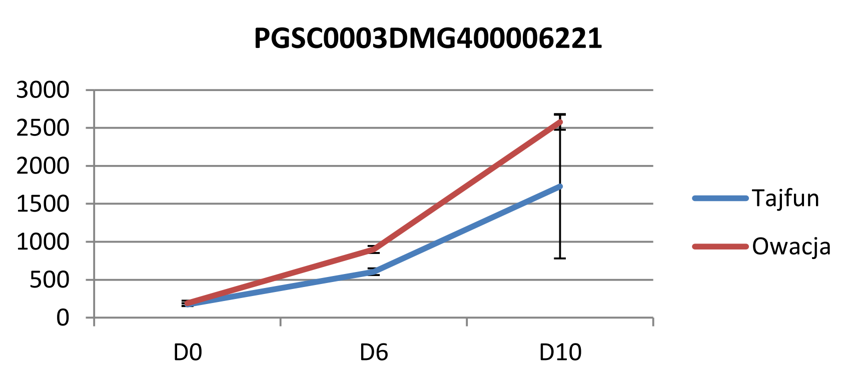

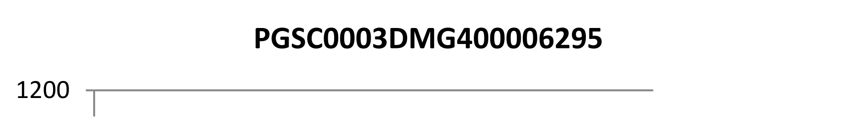

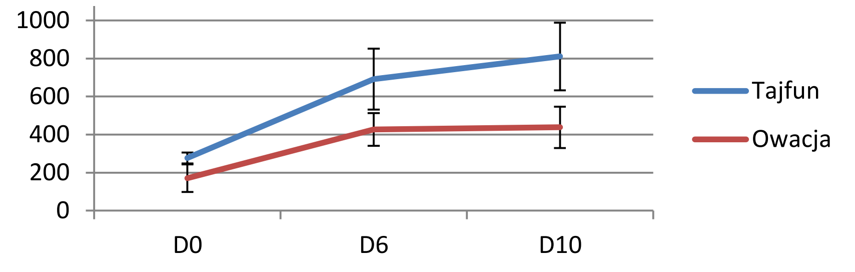

**PGSC0003DMG400006555**	499,33	902	1030,67	492	1234	1447,3	81	80,52	171,61	228	74	61,33
**PGSC0003DMG400006661**	9977,33	132489	71940	4808	84990	15554	4493,5	19346	18683	2446,8	30001	3552,95
**PGSC0003DMG400006768**	3922,33	15150	28235	4427,33	22246	54433	1140,4	2267,6	8845,5	848,22	1518,4	3901,78
**PGSC0003DMG400006784**	537,33	1710,7	1311,33	496,67	1108,7	710,67	179,29	362,6	267,33	202,53	132,08	50,21
**PGSC0003DMG400007200**	1352,67	4693,3	5872,67	1049,33	2567,3	1878,7	775,39	1308,9	1760,3	430,35	926,59	682,17

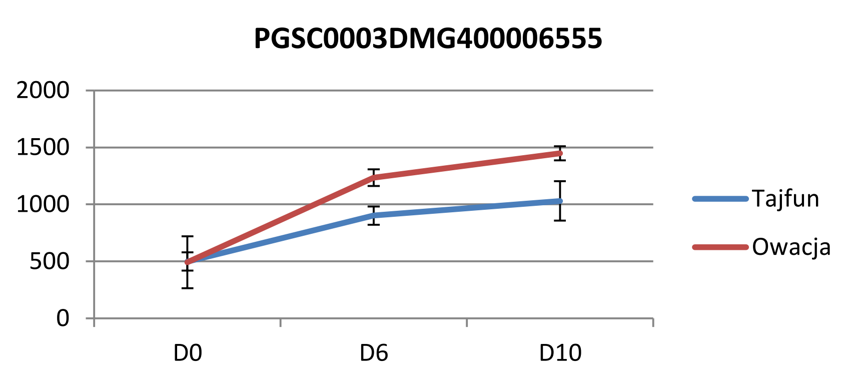

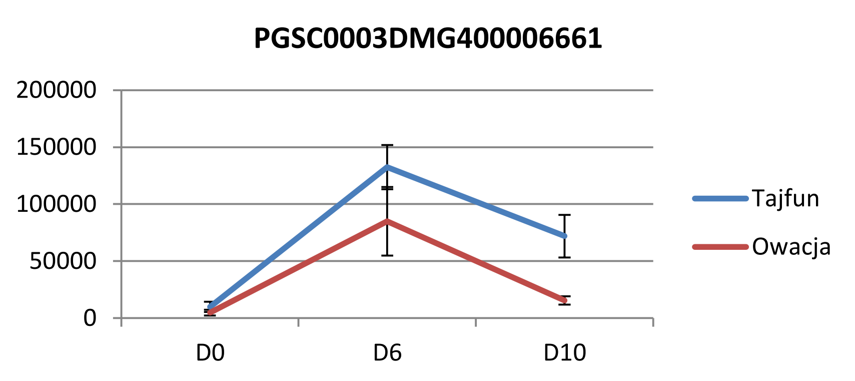

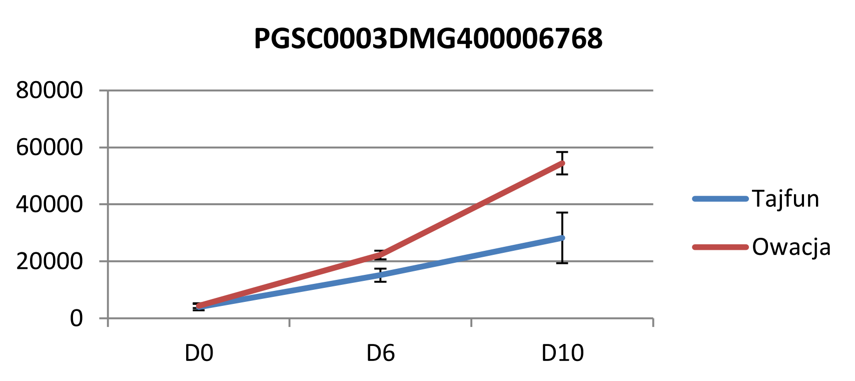

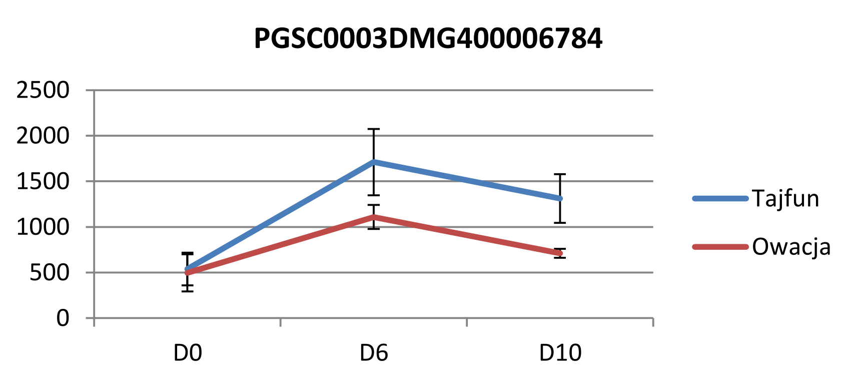

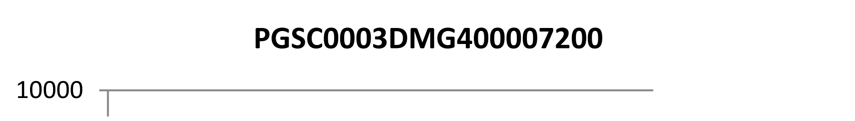

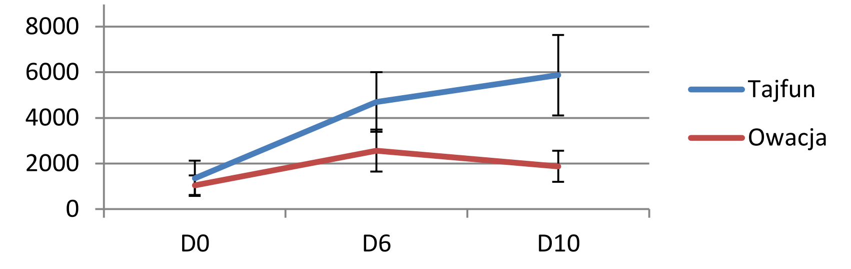

**PGSC0003DMG400007373**	876	4504	10660,7	874	6471,3	16332	294,69	951,17	4672,7	135,54	1238,9	1958,12
**PGSC0003DMG400008148**	289,33	10757	4875,33	200,67	7290	1746	101,24	2230	1300,5	20,23	582	463,13
**PGSC0003DMG400008176**	234	5652,7	3389,33	177,33	4144,7	1208,7	74,65	1047,7	742,18	21,94	327,78	355,18
**PGSC0003DMG400008348**	448	1446	2620,67	334,67	2429,3	3952	40,45	474	1384,8	72,01	252,46	281,57
**PGSC0003DMG400008434**	77,33	290	332,67	34,67	151,33	748,67	18,15	82,49	71,7	30,09	57,87	273

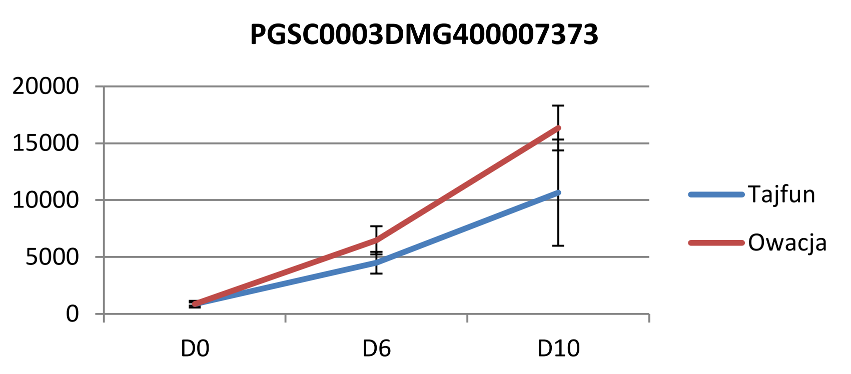

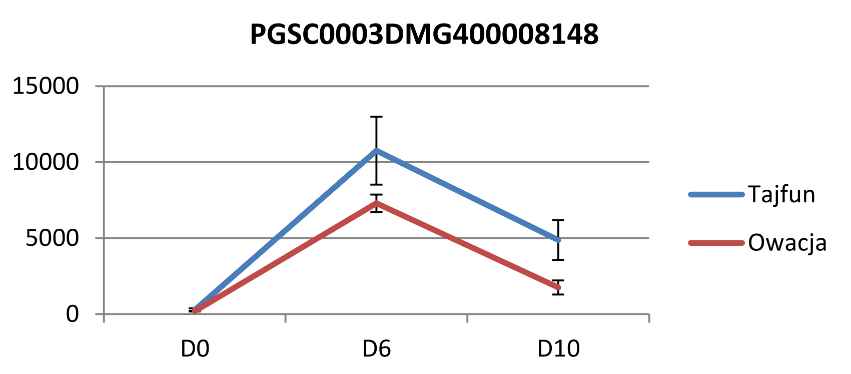

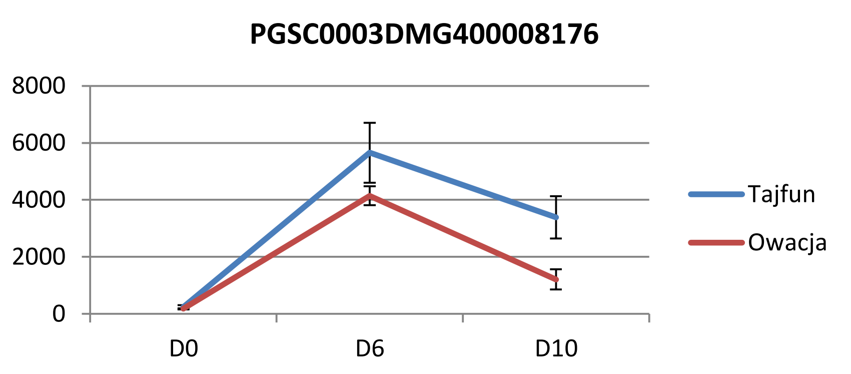

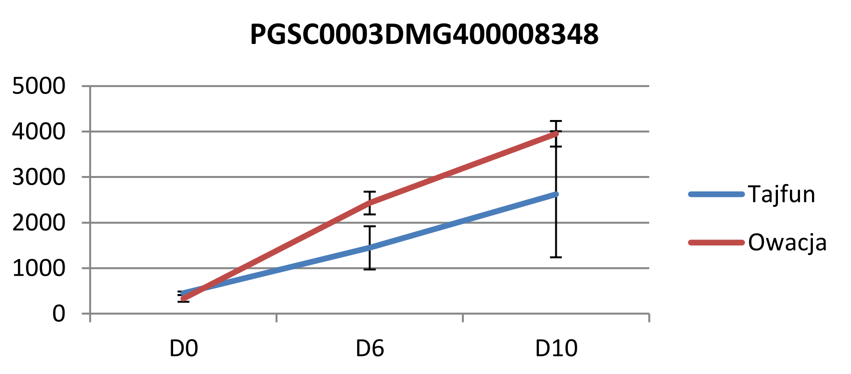

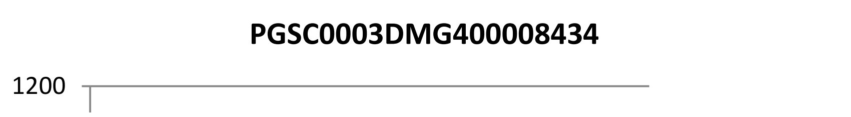

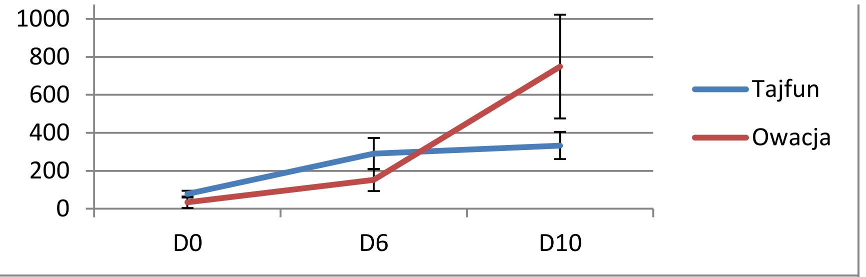

**PGSC0003DMG400008497**	0,67	485,33	408,67	2	128	27,33	1,15	127,03	346,89	3,46	49,76	18,04
**PGSC0003DMG400008502**	542	1400,7	1071,33	665,33	854,67	623,33	103,13	168,31	284,85	144,46	132,16	42,44
**PGSC0003DMG400008562**	78,67	733,33	907,33	117,33	1416,7	1248	76,14	159,21	271,09	101,93	184,85	139,27
**PGSC0003DMG400008644**	257,33	842	695,33	145,33	504	324,67	108,01	127,67	91,66	59,68	81,68	132,01
**PGSC0003DMG400009005**	196,67	4315,3	16420	227,33	6504,7	29582	48,88	831,25	7274,7	71,11	363,92	4491,32

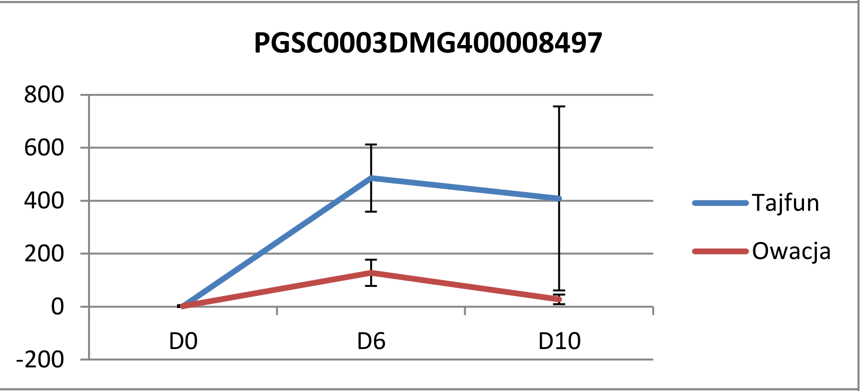

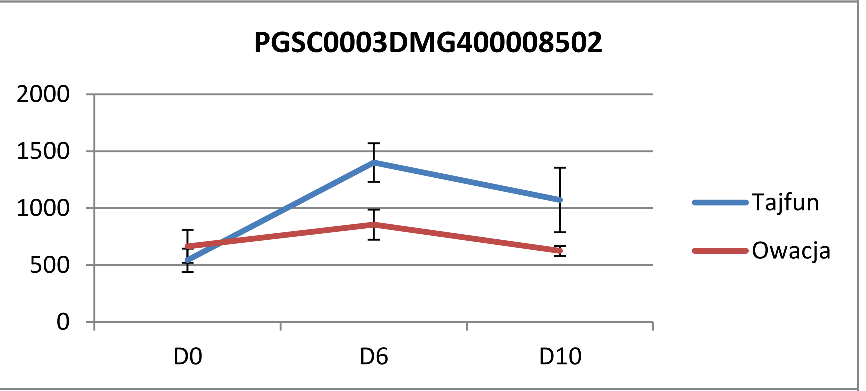

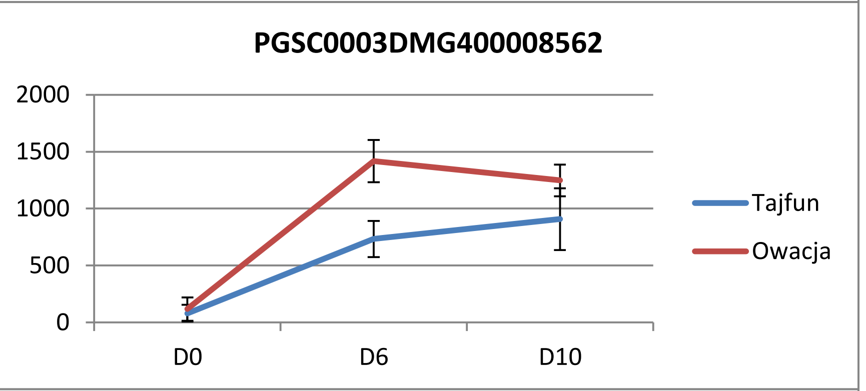

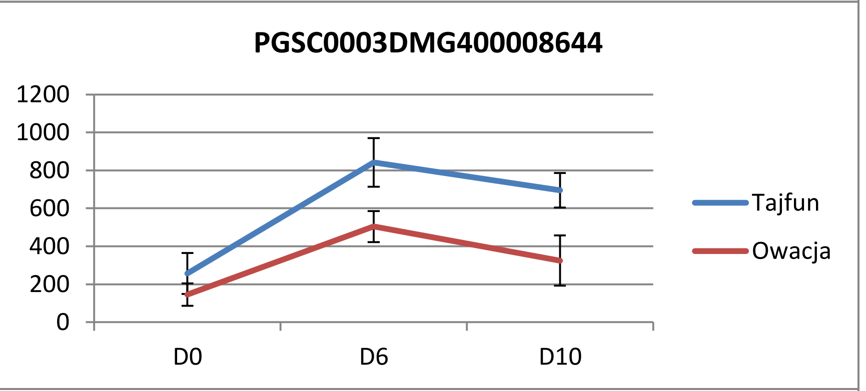

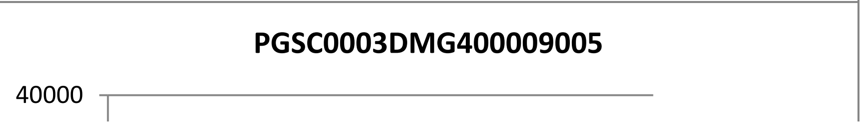

**PGSC0003DMG400009095**	1800,67	4548	6589,33	1991	8694,3	12729	497,68	1116,7	2647,4	1120,2	1488,9	3253,25
**PGSC0003DMG400009295**	2020	3774,7	6670	1673,33	2592	3050	477,52	713,05	357,51	45,71	338,96	822,87
**PGSC0003DMG400009303**	135,33	506,67	508,67	114	275,67	288,67	66,52	72,45	66,94	69,31	55,63	28,02
**PGSC0003DMG400009330**	33,33	556	1193,33	34,67	326	570,67	57,74	66,3	340,57	60,04	49,52	75,16
**PGSC0003DMG400009414**	3542,67	12581	17913,3	2466	20215	35660	544,71	209,25	4162,1	889,23	1862,2	1897,39

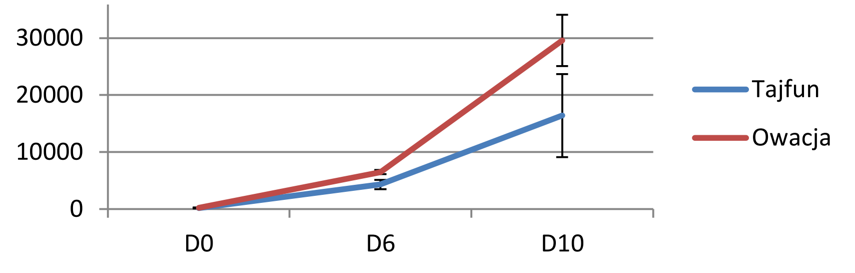

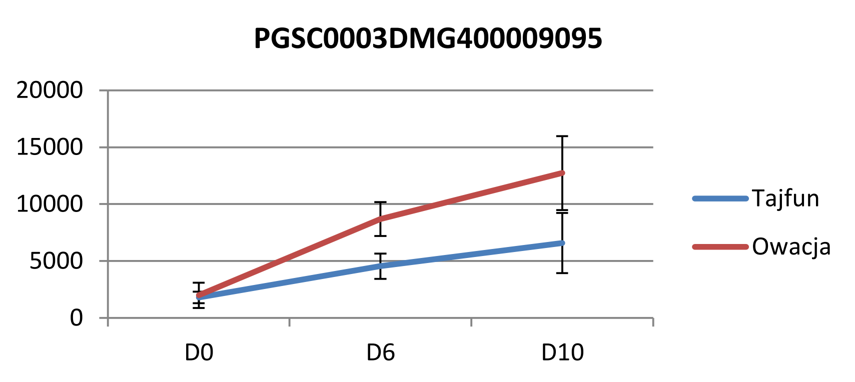

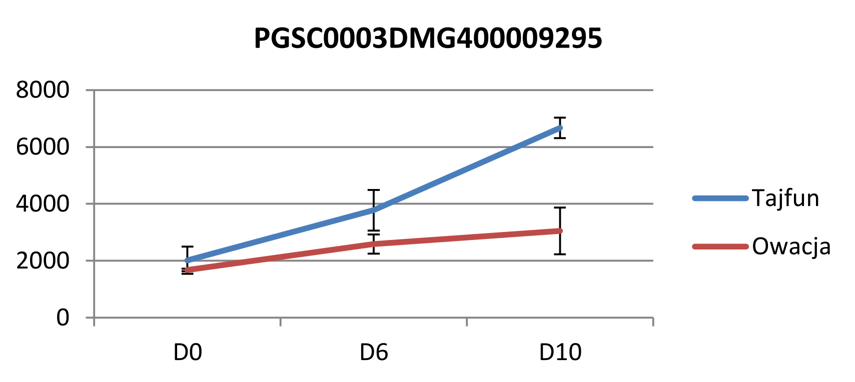

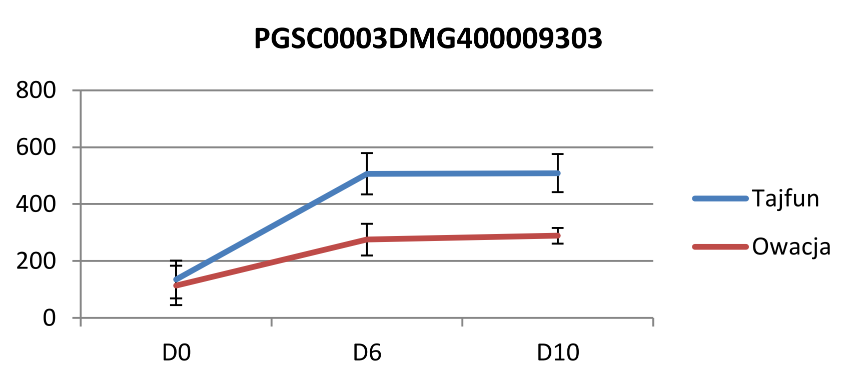

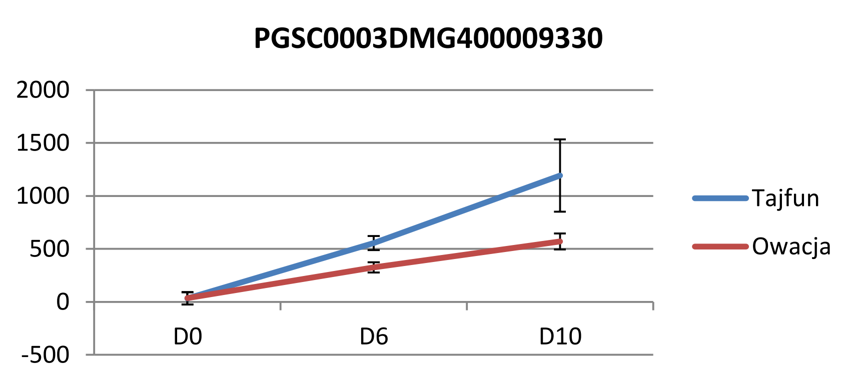

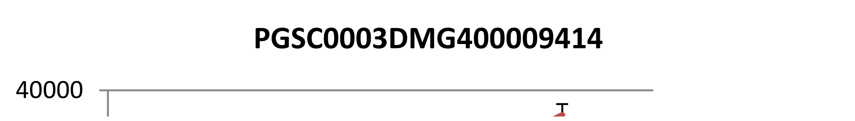

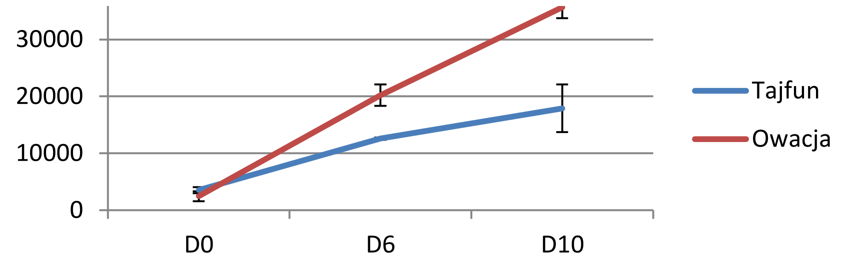

**PGSC0003DMG400009473**	504	1413,3	1425,33	383,33	929,67	580	154,62	138,4	309,59	107,23	88,36	140,34
**PGSC0003DMG400009482**	578	4080	1300	641	2253,7	341,33	104,89	176,68	413,03	68,24	394,05	69,55
**PGSC0003DMG400009599**	5739,33	23041	16062	6896	37891	23287	1102,6	920,44	3189,9	3701,1	1207,7	1601,33
**PGSC0003DMG400009714**	179,33	652,67	504	151,33	452,67	288,67	111,79	93	54,15	62,01	129,53	21,2
**PGSC0003DMG400009927**	102	1577,3	575,33	94	825,33	119,33	88,61	346,84	325,33	84,07	161,71	44,74

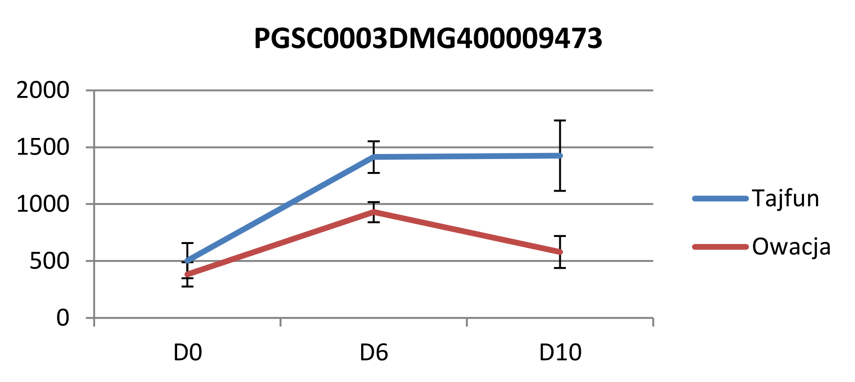

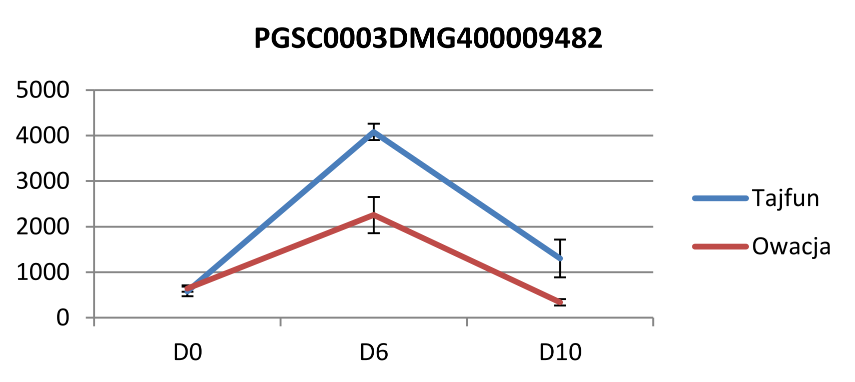

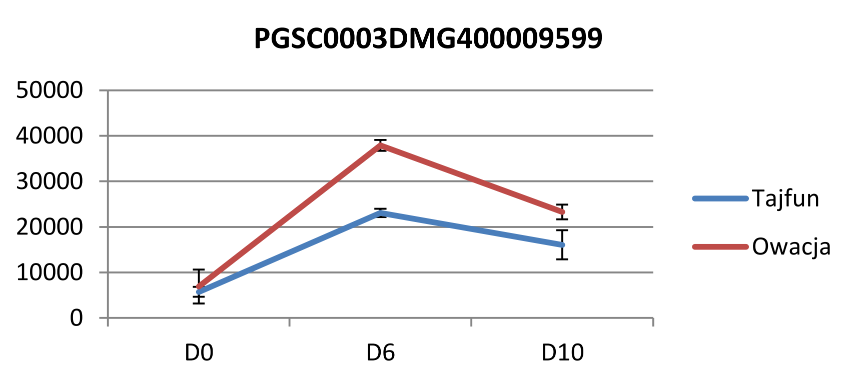

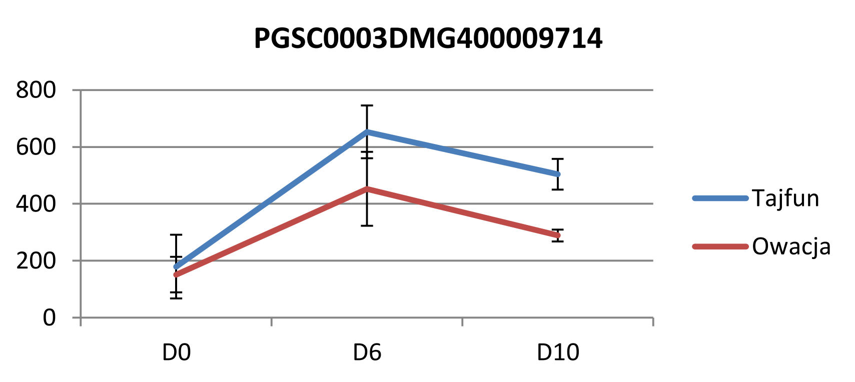

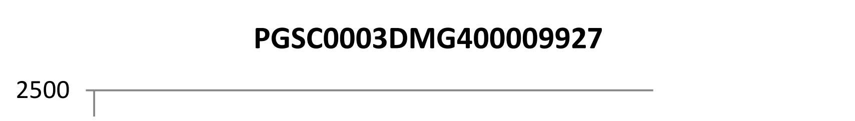

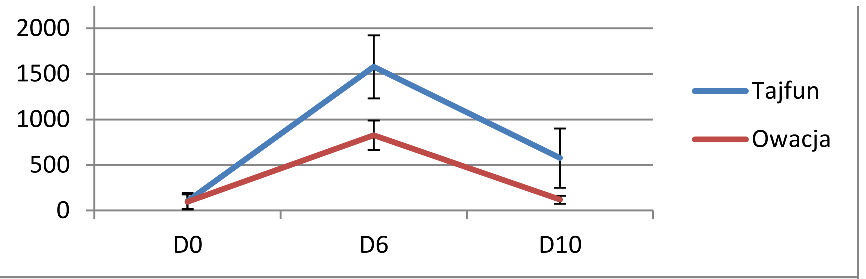

**PGSC0003DMG400010172**	42	687,33	577,33	20,67	349,33	86	52,12	228,71	46,19	3,06	57,77	27,78
**PGSC0003DMG400010207**	0,67	247,33	801,33	6	679,33	8586,7	1,15	191,42	1187	5,29	126,24	4809,49
**PGSC0003DMG400010270**	248	775,33	761,33	142	354	350	58,62	117,17	116,21	52,57	36,39	50
**PGSC0003DMG400010318**	283,33	1147,3	1021,33	164	820	338	38,85	126,64	61,78	38,16	114,75	136,84
**PGSC0003DMG400010498**	3,33	90,67	926,67	2	8,67	77,33	1,15	26,1	370,51	3,46	4,16	28,73

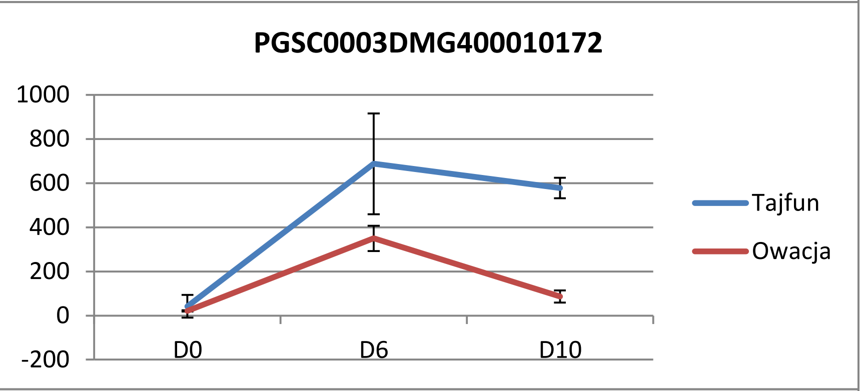

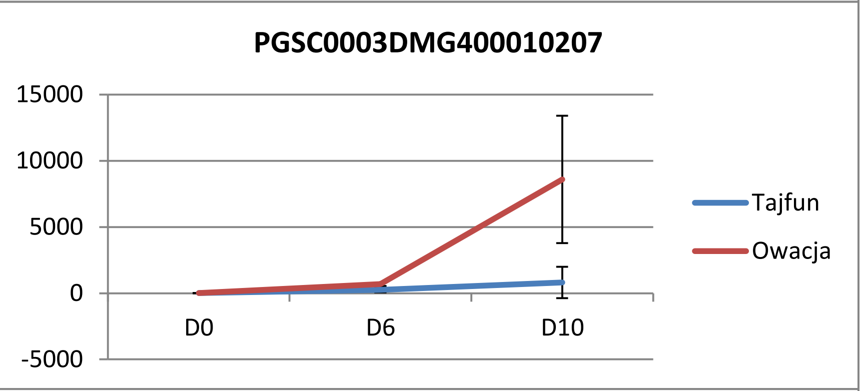

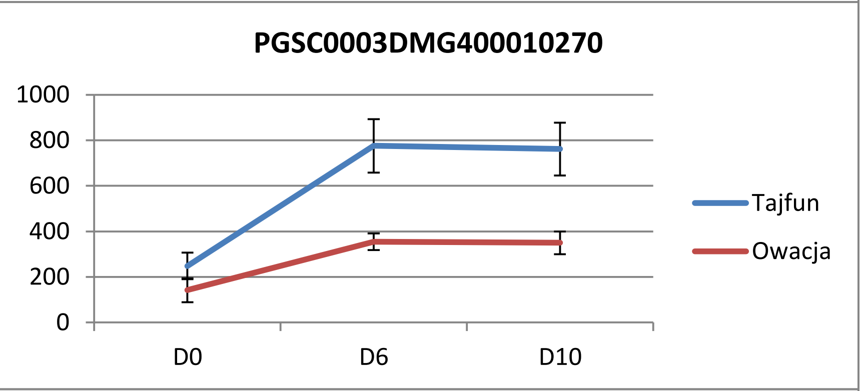

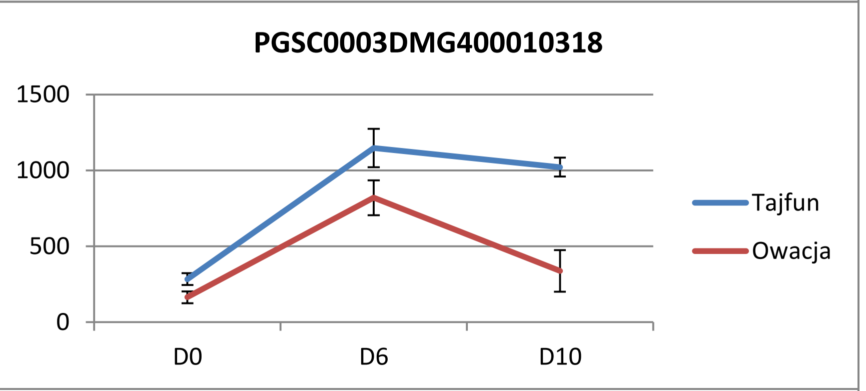

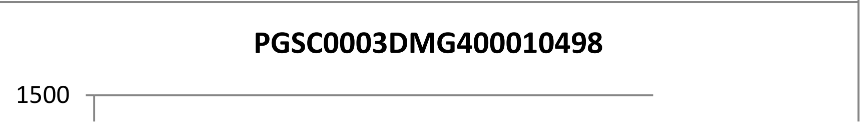

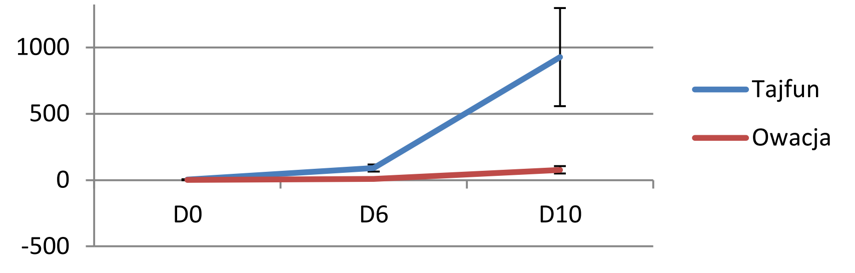

**PGSC0003DMG400010622**	12,67	390,67	241,33	6,67	137,33	30	16,77	23,86	51,39	3,06	22,3	16,37
**PGSC0003DMG400010698**	124	451,33	310	98	235,33	132,67	32,19	90,72	54,99	18,33	21,94	19,43
**PGSC0003DMG400010751**	106,67	290	292,67	66	177,33	122	32,08	65,48	40,07	32,92	27,15	40,15
**PGSC0003DMG400011394**	181	11402	10548,7	136	16565	5192,3	54,74	1190,7	3697	46,86	3098,4	609,57
**PGSC0003DMG400011570**	184,67	652,67	1054,67	104	448,67	611,33	49,08	124,1	211,3	53,33	48,39	35,8

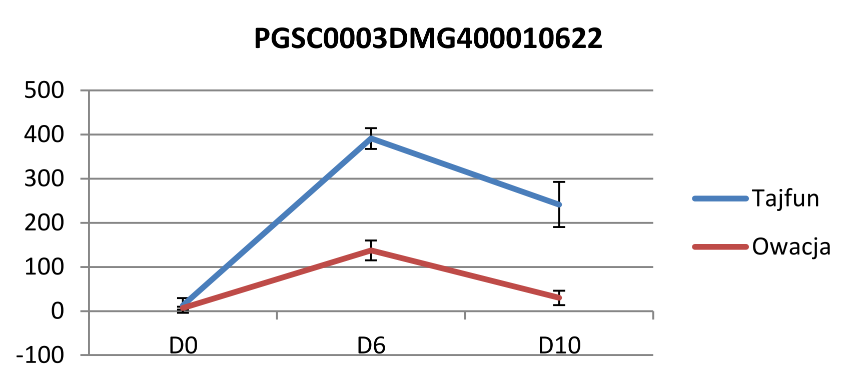

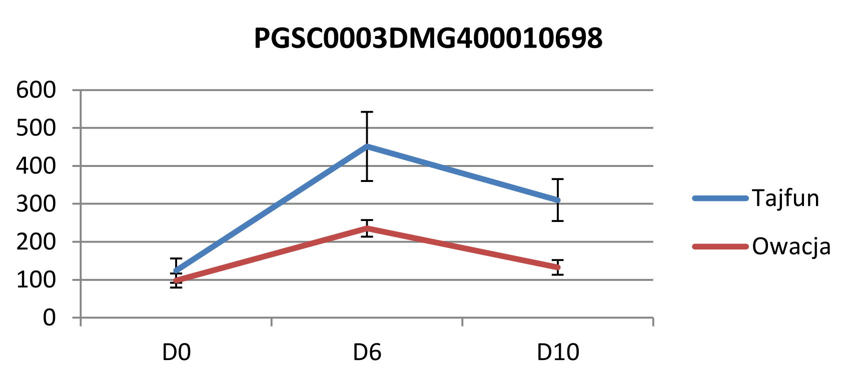

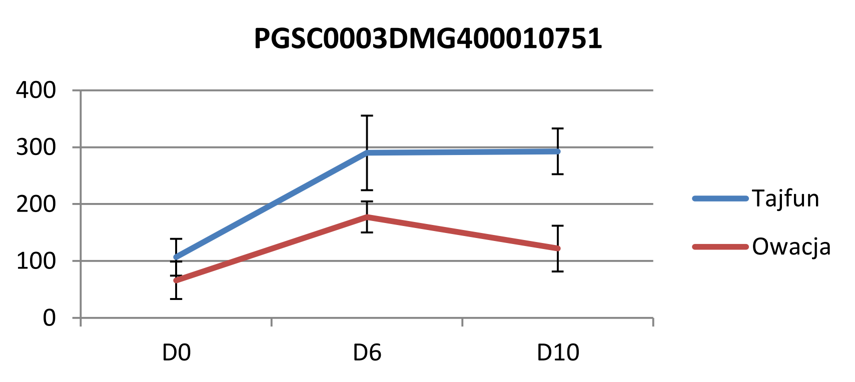

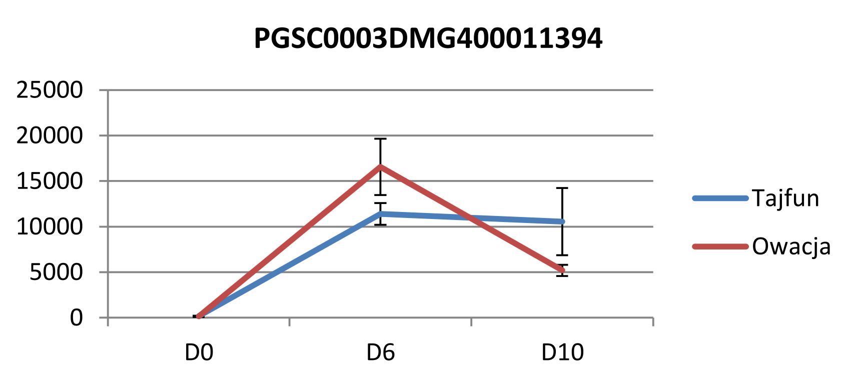

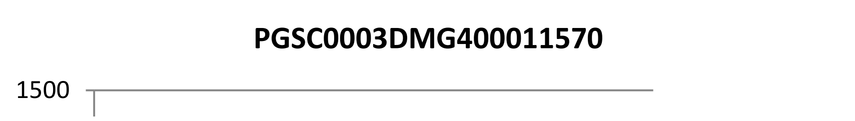

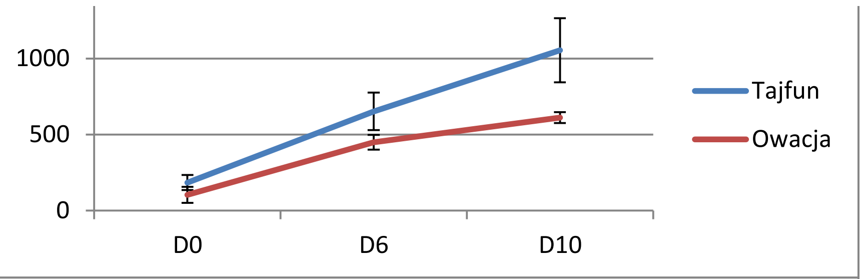

**PGSC0003DMG400011646**	60,67	232,67	256,67	74,67	126	114	13,32	32,08	99,45	24,44	16,37	21,63
**PGSC0003DMG400011704**	32	292,67	381,33	15,33	157,33	82,67	20	60,18	40,27	8,08	31,64	21,39
**PGSC0003DMG400011729**	27,33	109,33	699	43,33	206	1441,3	23,86	48,06	740,58	25,32	37,51	212,53
**PGSC0003DMG400011792**	127,33	455,33	375,33	93,33	214	88	12,22	82,37	89,92	36,3	39,04	30
**PGSC0003DMG400011868**	265,33	2096	5808	178,67	2944,7	3255,3	118,01	298,24	680,2	70,01	677,52	806,41

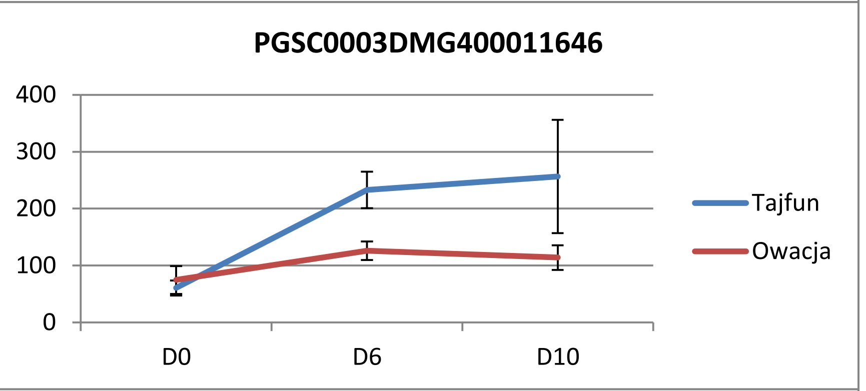

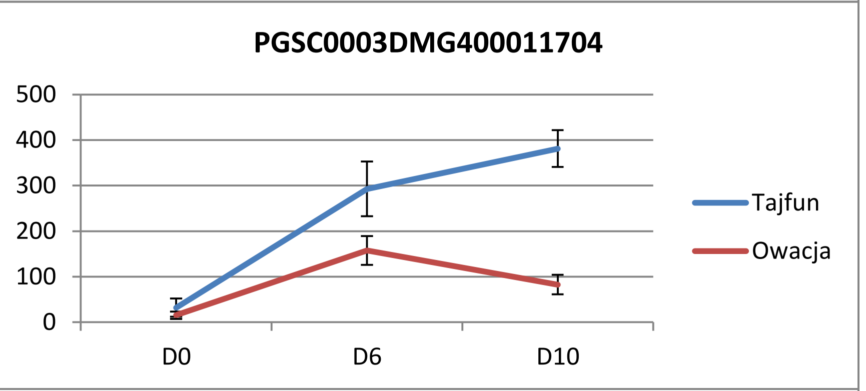

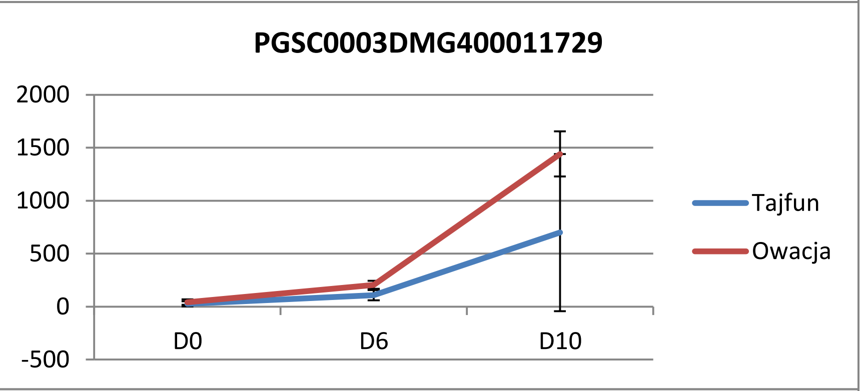

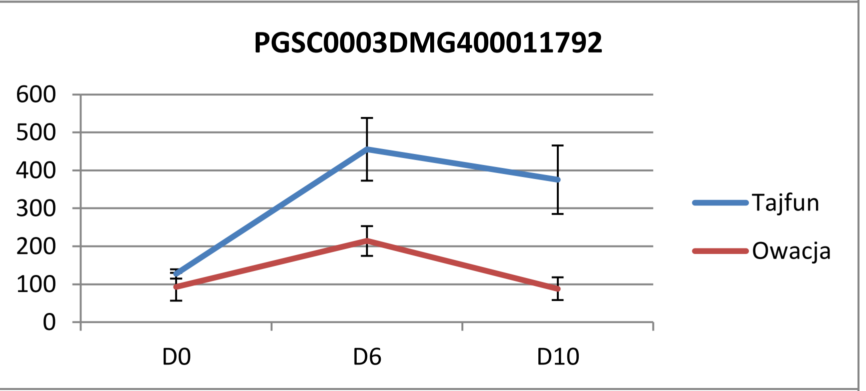

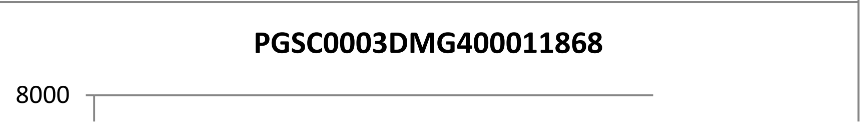

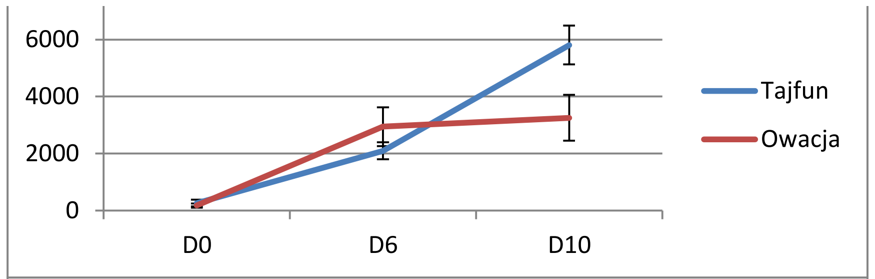

**PGSC0003DMG400012213**	204,67	541	937	121,67	342	482,67	43,73	52,26	328,86	56,89	96,57	85,32
**PGSC0003DMG400012248**	63,33	847,33	904,67	80	1404,7	488,67	31,39	73,06	215,54	34,12	109,71	107,43
**PGSC0003DMG400012408**	530	3274,7	2929,33	454	1747,3	1480,7	231,9	262,12	601,81	136,92	150,76	68,16
**PGSC0003DMG400012681**	1094,33	2740,7	2251,33	753,33	1579,7	1193	225,38	594,51	137,49	337,13	303,98	175,76
**PGSC0003DMG400012838**	332	9884	18098,3	255,67	15027	10659	111,66	2791,5	1709,9	8,14	3226,5	3394,36

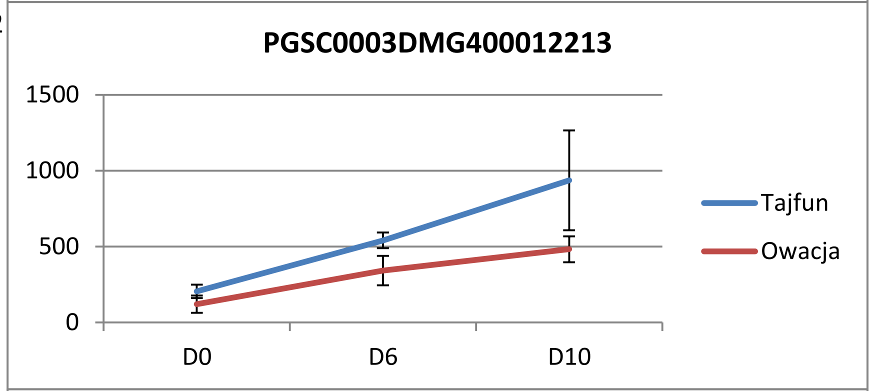

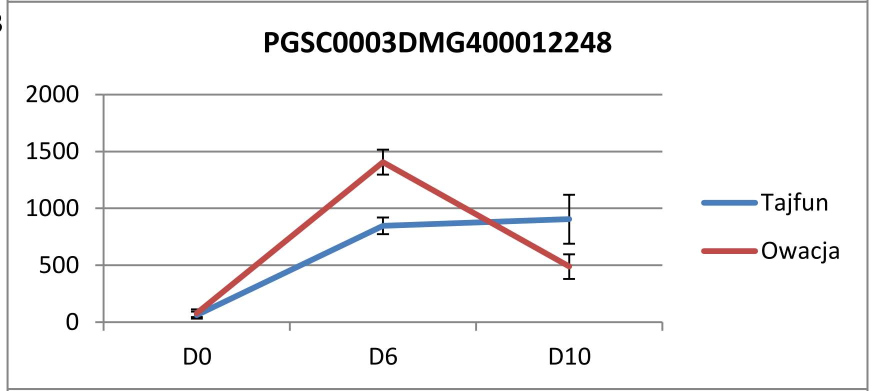

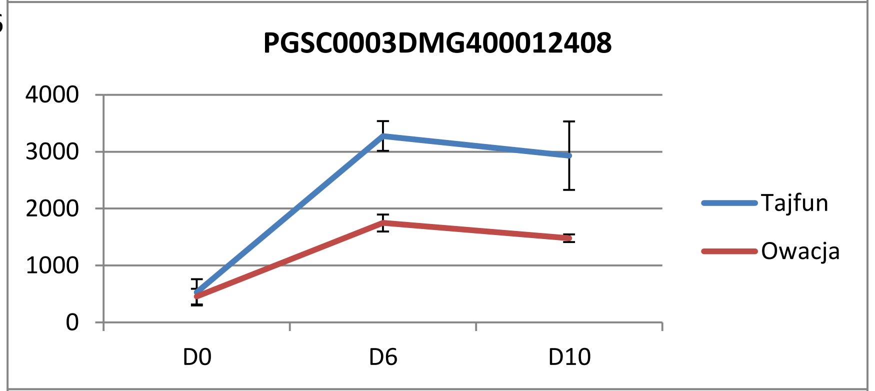

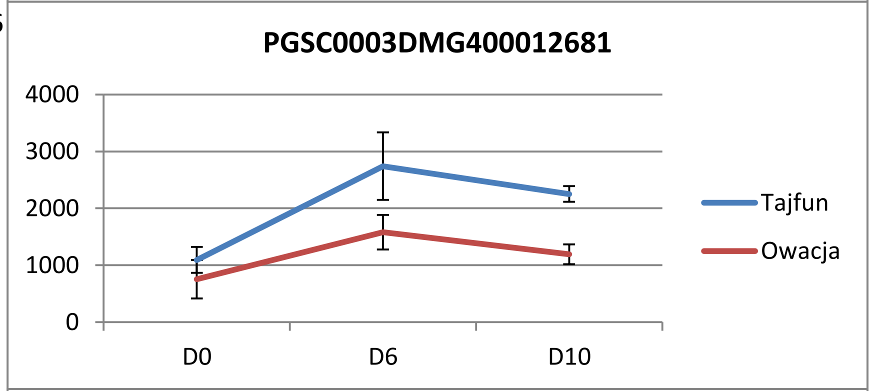

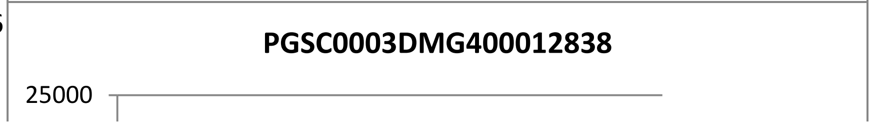

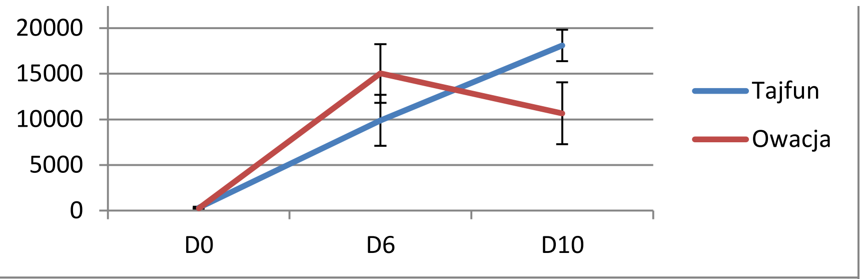

**PGSC0003DMG400013088**	1051,33	6133,3	7932,67	861,33	10487	17322	269,05	1198,6	3929,1	305,51	947,75	4585,42
**PGSC0003DMG400013662**	310,67	611	780,33	228,67	387,67	364	58,73	51,8	39,37	61,16	60,8	140,33
**PGSC0003DMG400013663**	126	312,67	277,33	109,33	188	146,67	52,12	101,91	83,34	25,01	42,33	23,18
**PGSC0003DMG400013918**	308,67	862,67	1076,67	260,67	526,67	442,67	79,25	109,18	79	13,32	88,1	88,37
**PGSC0003DMG400014166**	1538,67	4914,7	8414,67	1359,33	6798	19017	262,92	295,53	2814	350,69	824,82	1482,27

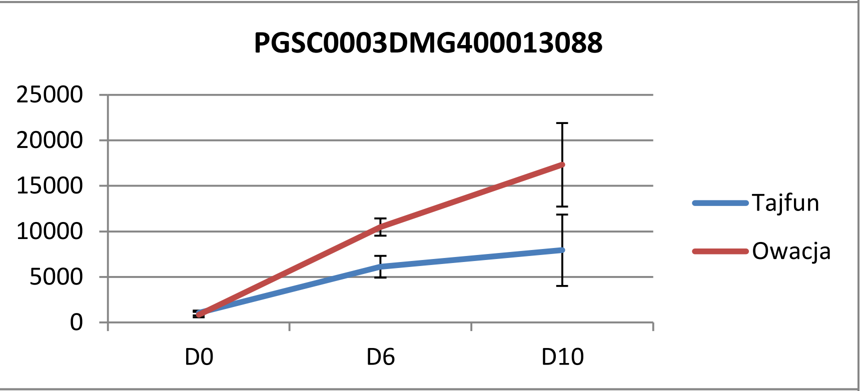

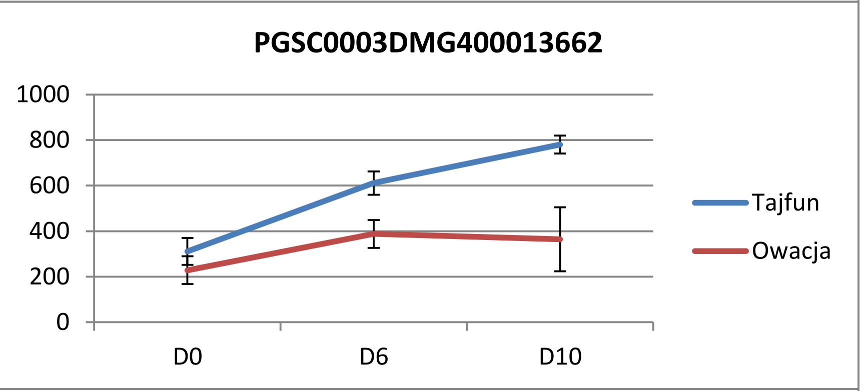

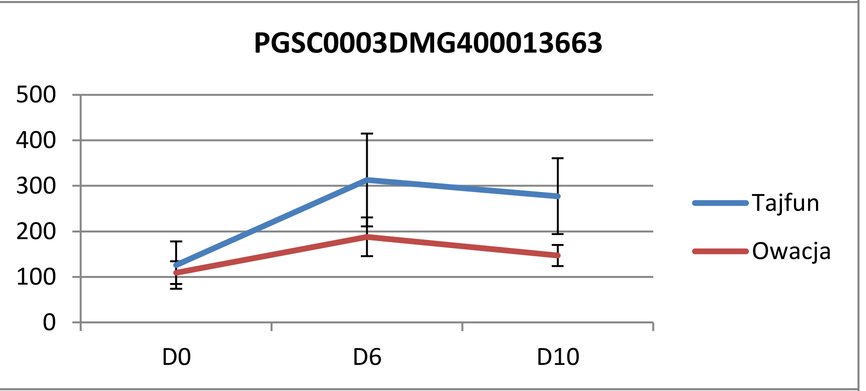

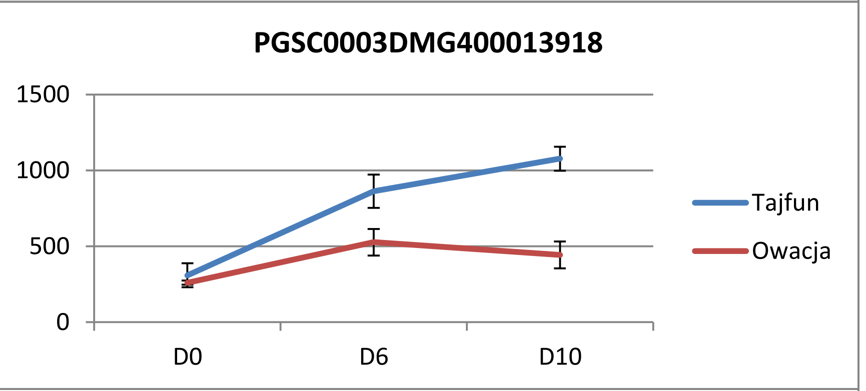

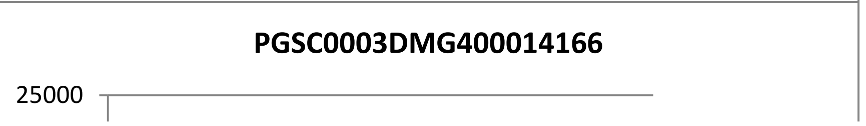

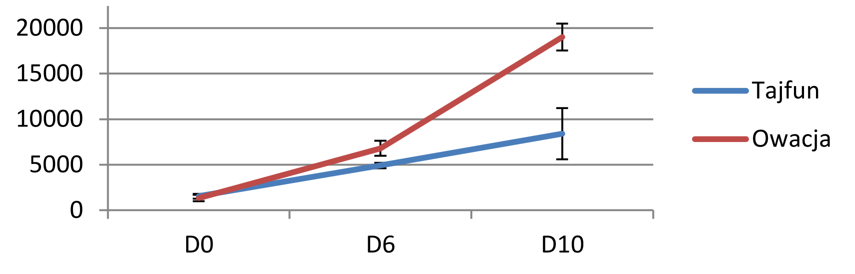

**PGSC0003DMG400014191**	1098,67	2000	2432	790,67	1417,3	1134	264,06	275,96	353,21	120,5	162,73	316

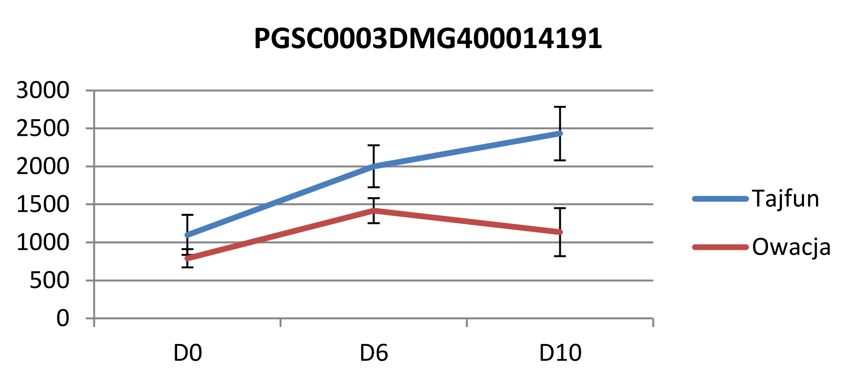

**PGSC0003DMG400014225**	7216	22666	37012,7	6162	35648	99227	1352,8	2368	11524	1932	5528,8	2625,38

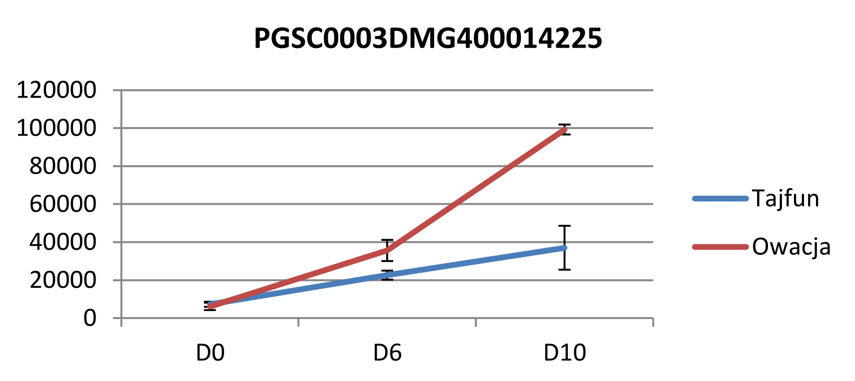

**PGSC0003DMG400014248**	3060,67	14861	69237,3	3083,33	42547	141899	622,74	2076,9	23110	663,97	1831,4	19504,9

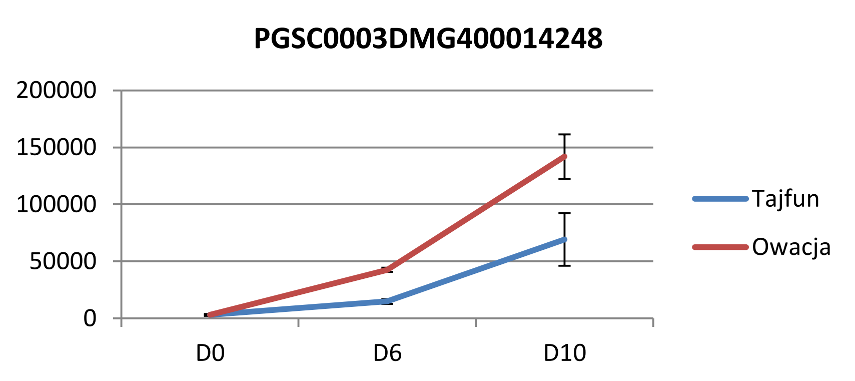

**PGSC0003DMG400014258**	28,67	88	186	9,33	34,67	59,33	8,08	12,49	45,83	1,15	10,26	12,86

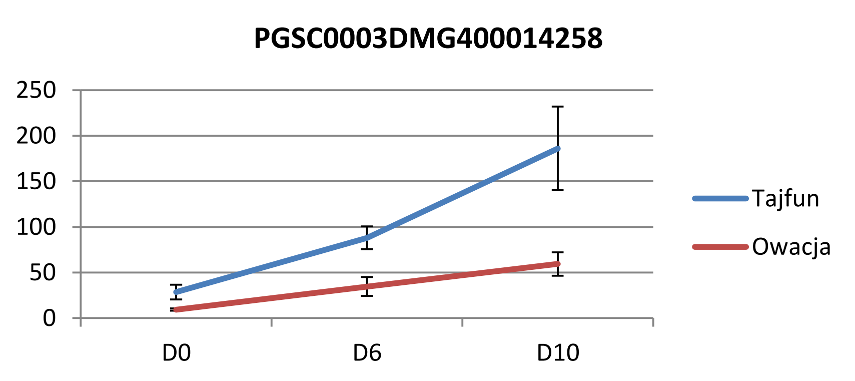

**PGSC0003DMG400014292**	345,33	9502	6078	274,67	12348	3576,7	121,73	1116,7	809,07	50,65	1464,7	1078,3

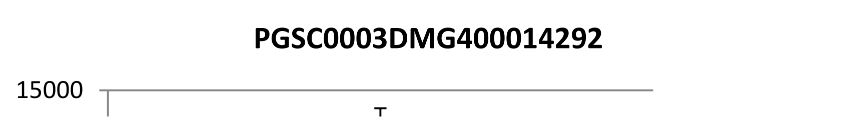

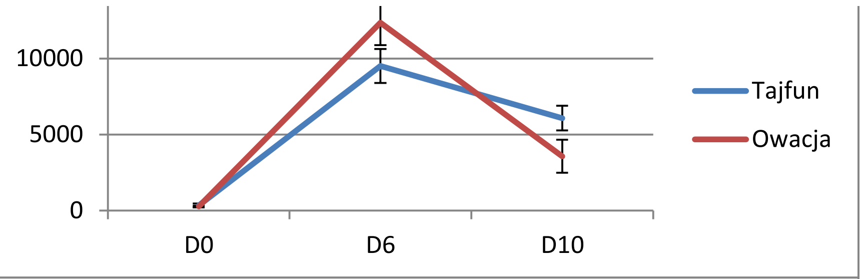

**PGSC0003DMG400014293**	67,33	49635	13875,3	32,67	32161	4626	50,85	9895,4	1592,6	17,93	2489,7	293,71
**PGSC0003DMG400014381**	1999,33	4468	4948	1898,67	2999,3	2382	311,06	223	752,52	191,26	104,54	648,06
**PGSC0003DMG400014528**	24,67	109,33	112,67	4	46	34,67	19,63	7,02	15,53	5,29	7,21	11,02
**PGSC0003DMG400014624**	399,33	958	948	346	664,67	458	92,29	134,89	173,22	144,54	70,89	94,3
**PGSC0003DMG400015238**	802,67	1902	1586	599,33	1121,3	790,67	179,6	147,5	163,6	53,45	88,01	224,45

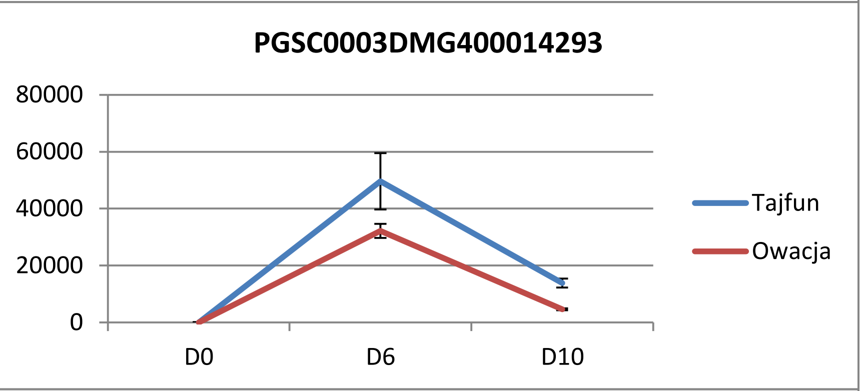

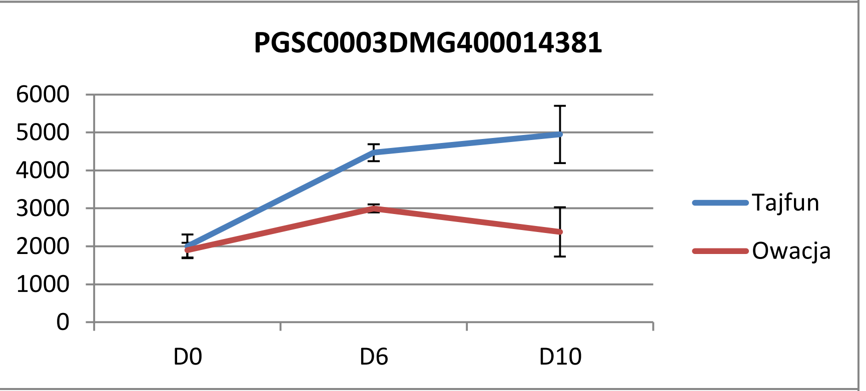

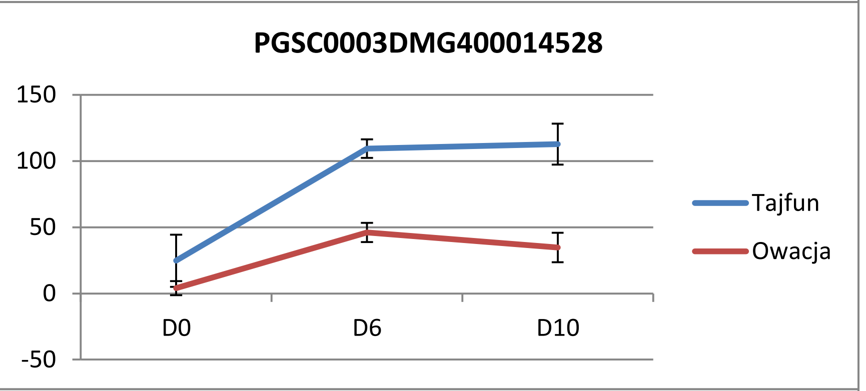

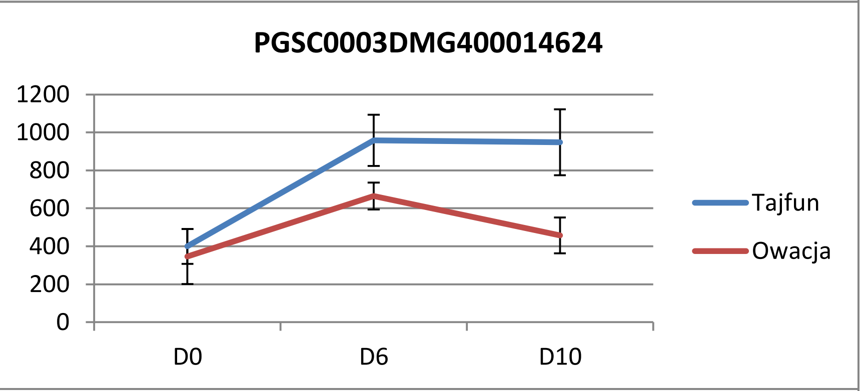

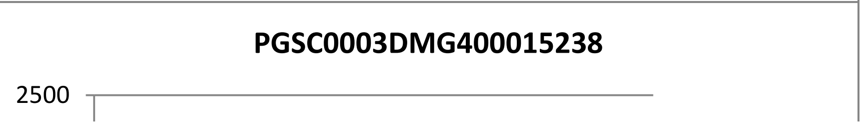

**PGSC0003DMG400015263**	658,33	3286	3934,33	514	4467,7	8013,3	217,16	619,7	1943	170,04	603,24	941,06
**PGSC0003DMG400015275**	0,67	2514,7	3359,33	3,33	1454,7	121,33	1,15	288,86	1186,7	3,06	307,94	41,49
**PGSC0003DMG400015330**	757,33	4202,7	11845,3	933,33	8367,3	19579	98,68	489,31	5235,4	160,65	1486,6	854,09
**PGSC0003DMG400015358**	338	743,33	1266	315,33	475,33	550,67	80,3	153,74	556,57	20,82	130,24	100,41
**PGSC0003DMG400015364**	24097,3	59476	52239,3	14438	35301	15497	7693,5	3923,2	16582	2589,6	5127,9	819,71

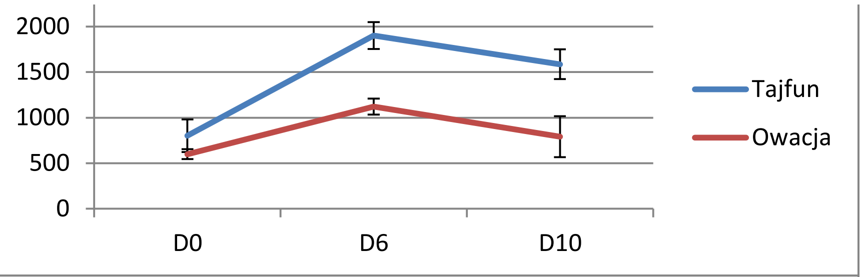

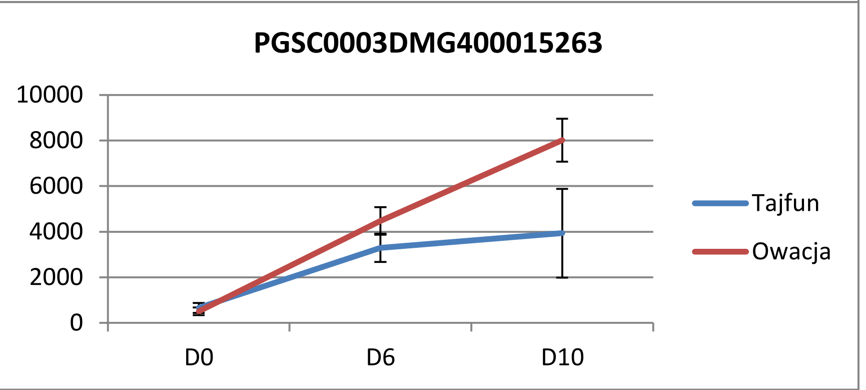

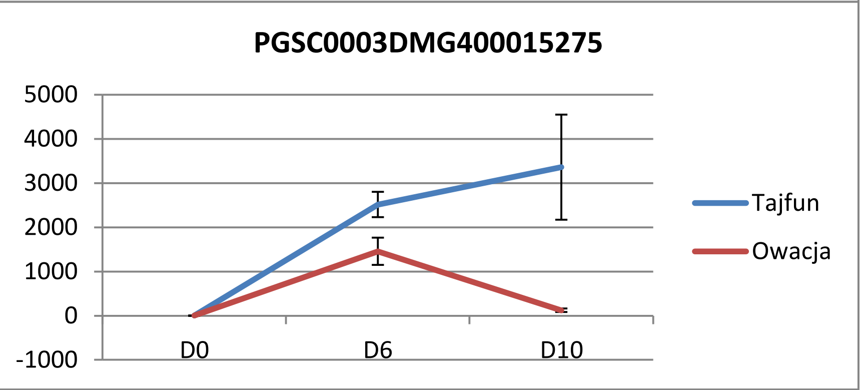

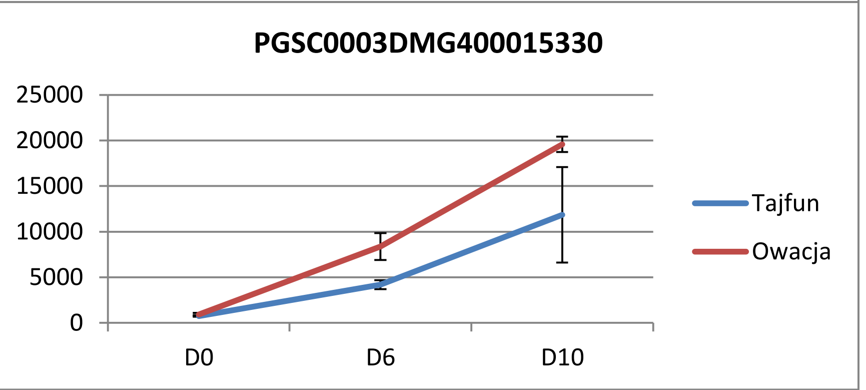

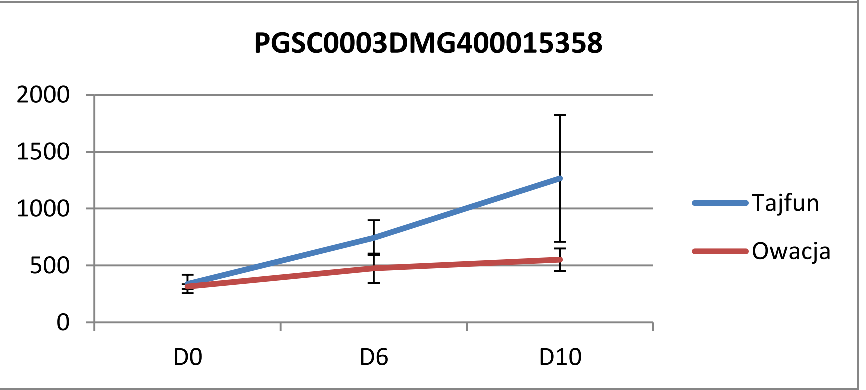

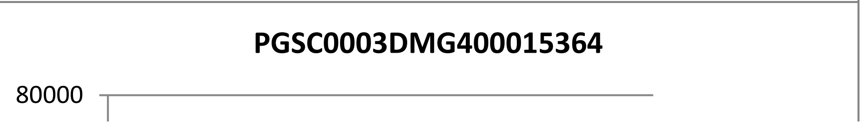

**PGSC0003DMG400015367**	495,33	1195,3	1268	436	612	474	37,81	37,81	76,63	126,76	99,02	40,84
**PGSC0003DMG400015406**	7039,33	14169	14820,7	6853,33	9422,7	8201,3	1013,3	846,51	2159,7	806,04	978,68	1084,21
**PGSC0003DMG400015407**	98	462,67	436	120	297,33	217,33	85,35	40,02	92,59	55,46	57,98	58,05
**PGSC0003DMG400015754**	2329,33	6394	4392	2159,33	4142	2175,3	842,15	472,83	281,25	293,91	1127,5	418,08
**PGSC0003DMG400015992**	4319,33	7384,7	11068	5185,33	5277,3	5879,3	749,29	471,57	2424,1	1491,6	193,87	769,56

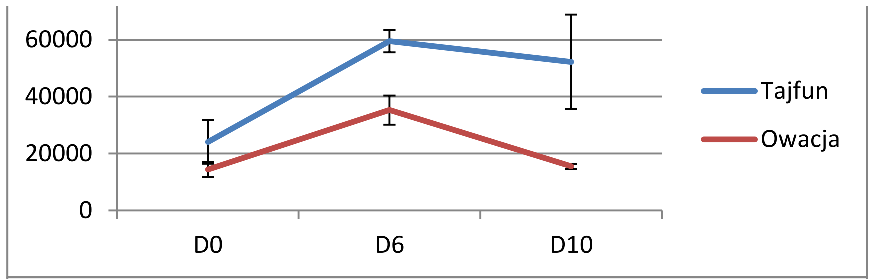

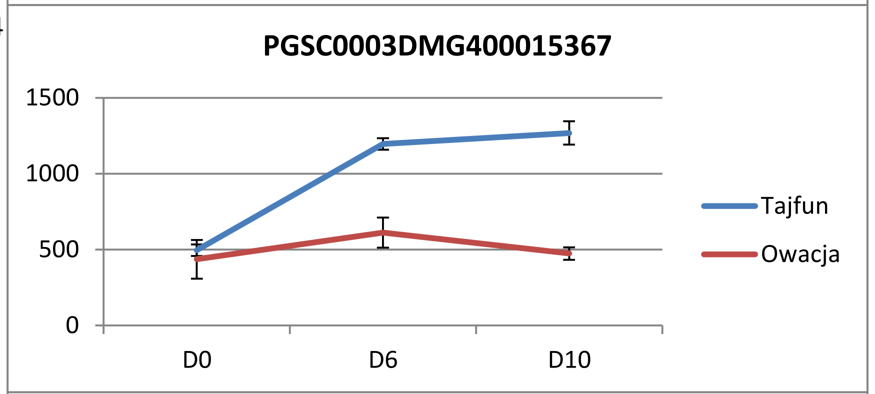

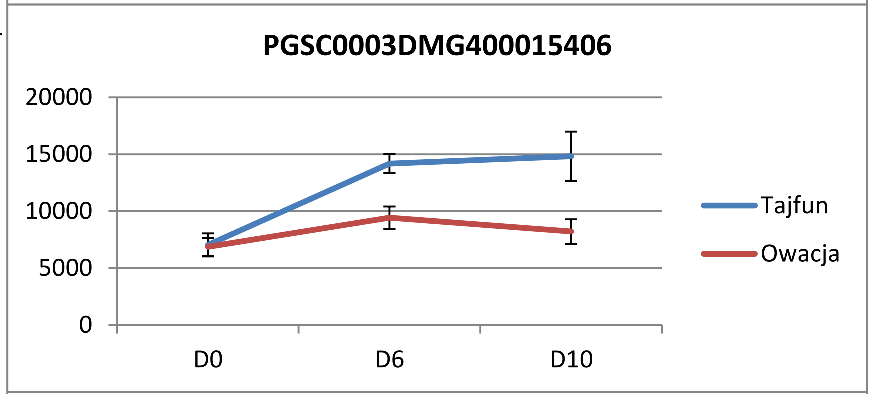

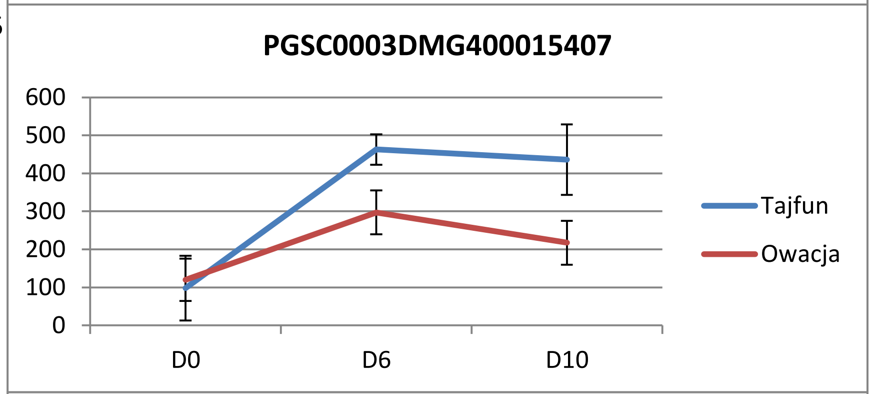

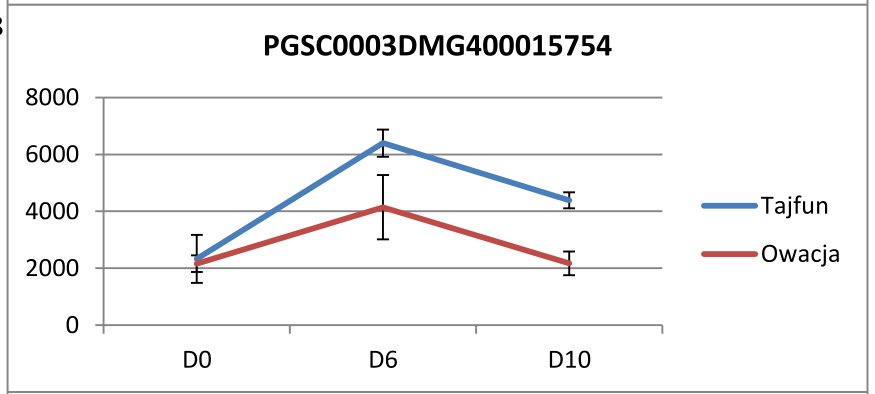

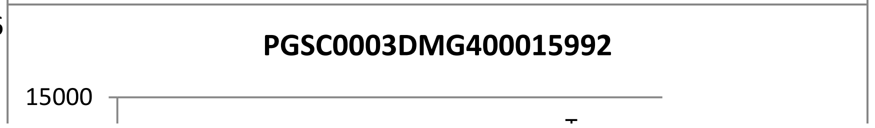

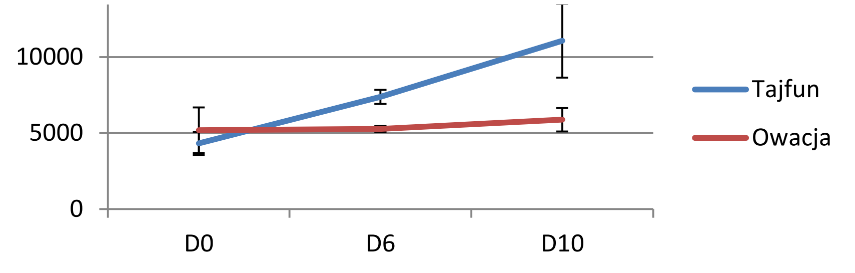

**PGSC0003DMG400016223**	64	191,33	200,67	31,33	80,67	77,33	34,7	37,11	68,86	14,47	37,17	27,15
**PGSC0003DMG400016518**	72,67	520	1010,67	0	108	158	125,86	69,54	338,24	0	36,5	40,6
**PGSC0003DMG400016535**	106,67	3472	3044	66,67	4937,3	4144	30,55	823,96	604,41	27,3	836,8	425,13
**PGSC0003DMG400016548**	120,67	291,33	844,67	142,67	569,33	470,67	55,08	80,03	169,17	44,56	154,3	216,37
**PGSC0003DMG400017207**	115,33	326	370	127,33	206,33	146,67	56,9	71,58	72,99	55,15	21,59	48,18

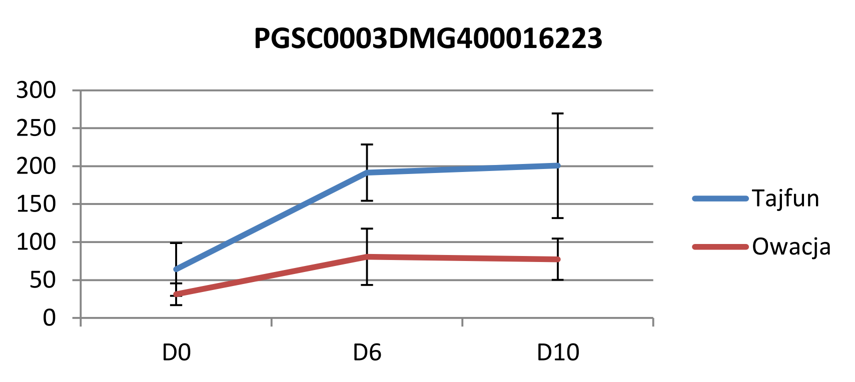

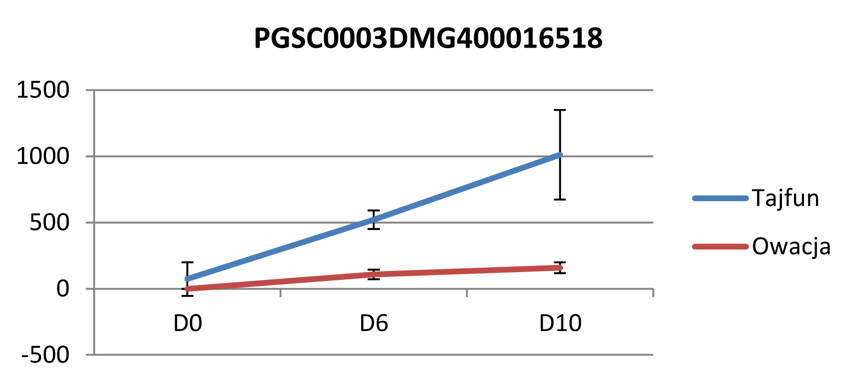

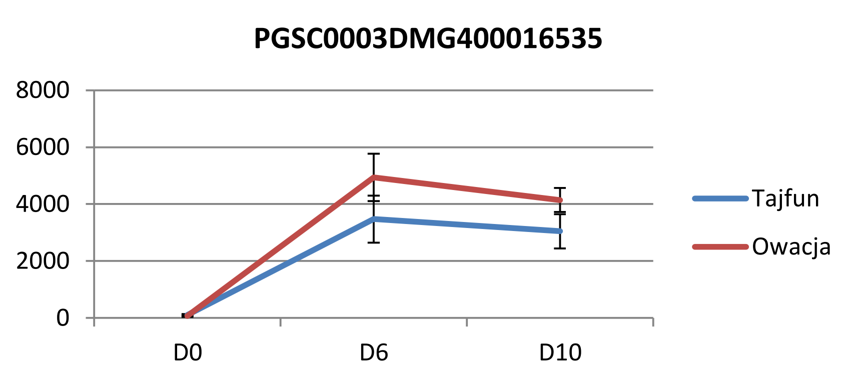

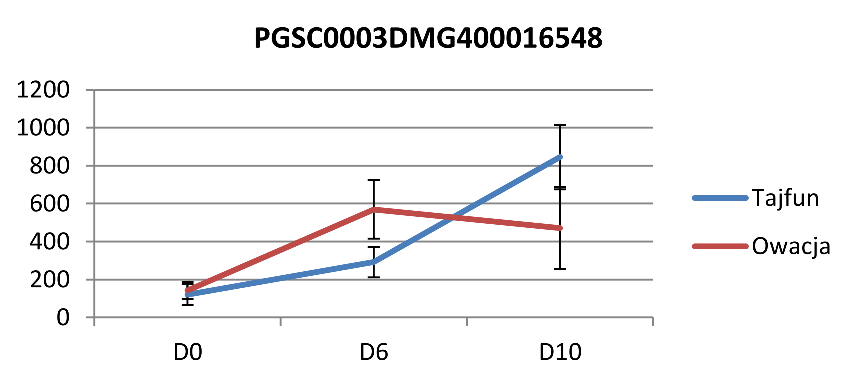

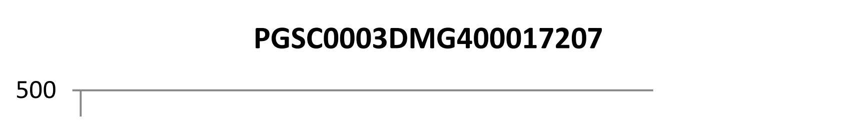

**PGSC0003DMG400017714**	4254	15363	10545,3	3424	10195	4658	1136,9	1122,4	1885	295,41	1801,9	876,17
**PGSC0003DMG400017730**	242	11953	30262	134,67	7121,3	7660,7	12	2279,4	12370	79,86	1989,9	558,5
**PGSC0003DMG400017933**	8034	17346	17232	7575,33	9317,3	7007,3	2114,4	1501,2	2526,8	891,55	2267,5	871,58
**PGSC0003DMG400018096**	269,33	874,67	784,67	190,67	406,67	277,33	93,43	146,29	79,76	31,01	64,04	34,78
**PGSC0003DMG400018107**	301,33	1154	661,33	216,67	558,67	232,67	81,1	143,68	164,25	25,48	51,78	21,2

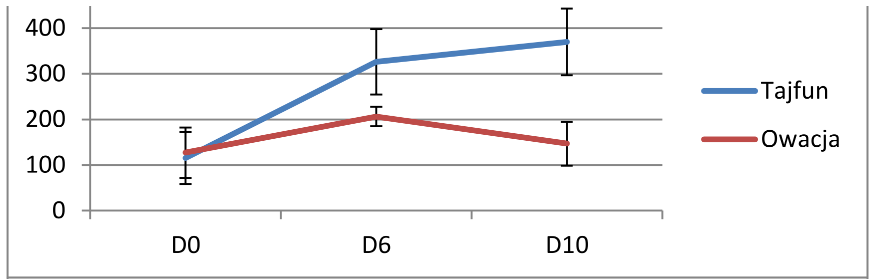

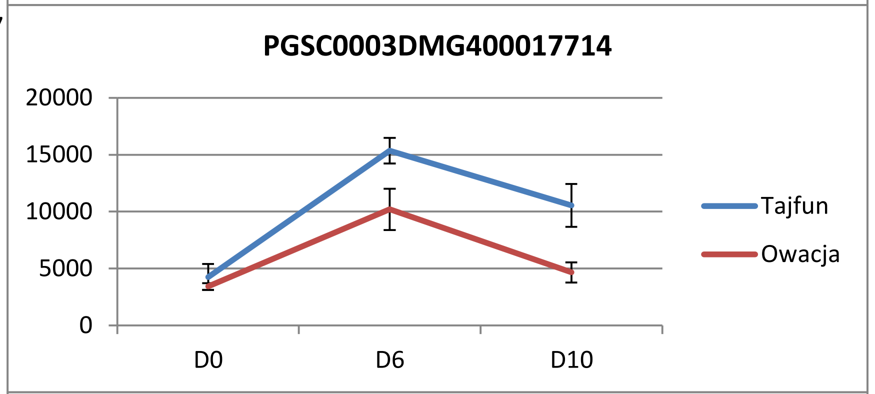

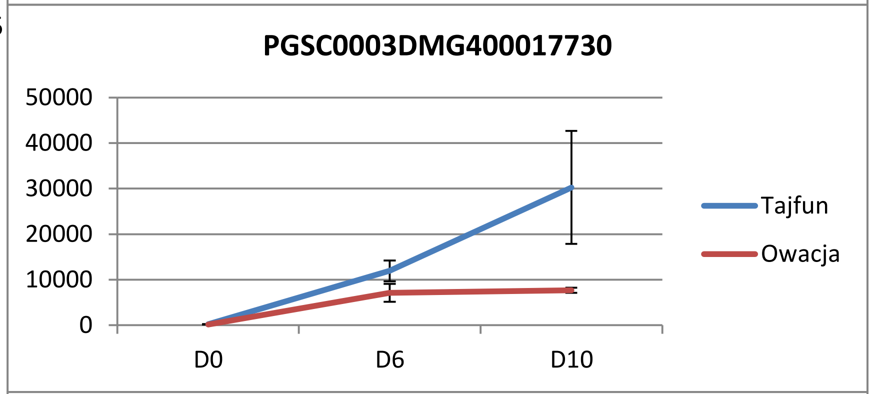

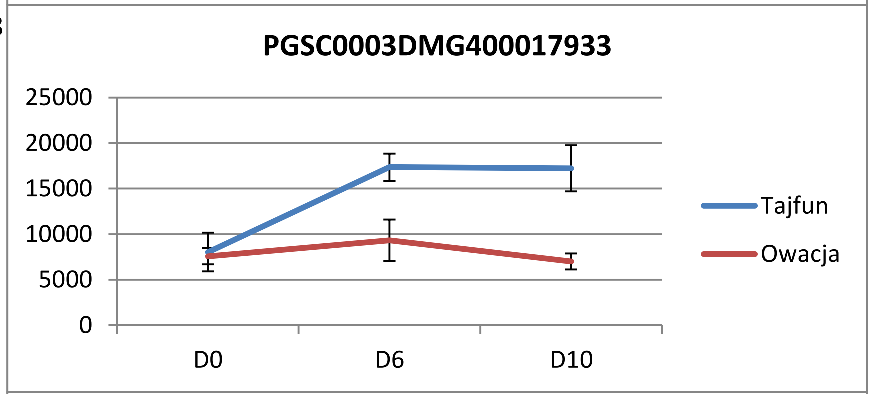

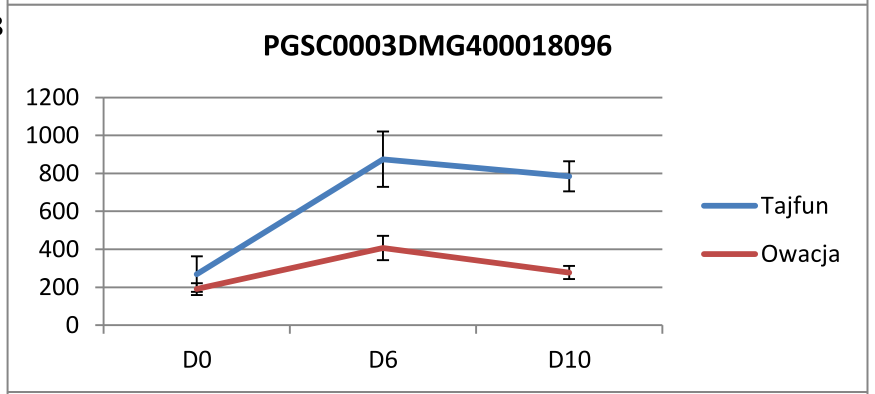

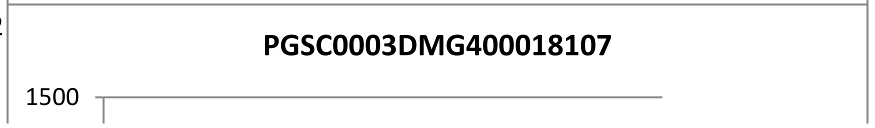

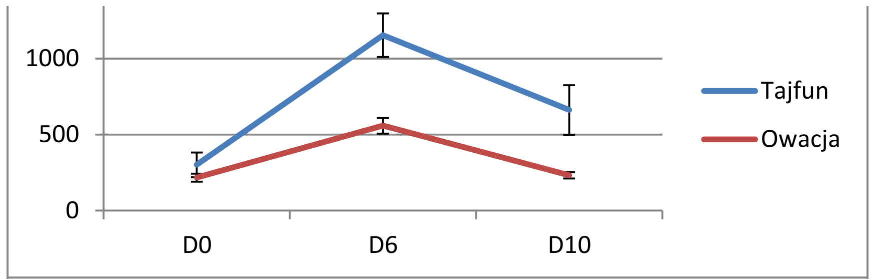

**PGSC0003DMG400018172**	179,33	486,67	641,33	111,33	270,67	262	111,16	150,36	125,07	61,07	31,77	71,36
**PGSC0003DMG400018236**	2407	7359,3	9803,67	1968	11082	14002	550,79	592,34	2838,8	596,91	338,6	1160,77
**PGSC0003DMG400018670**	549,33	1536	1440	315,33	2221,3	2186	166,3	304,41	638,44	131,15	314	250,1
**PGSC0003DMG400018964**	1488,67	4097,3	5137,33	1154	2590,7	2714,7	567,05	1040	728,49	217,15	607,62	840,15
**PGSC0003DMG400019077**	2742,67	6240	5746,67	5222	13117	14631	611,57	545,86	875,92	3052,9	1141,2	1523,15

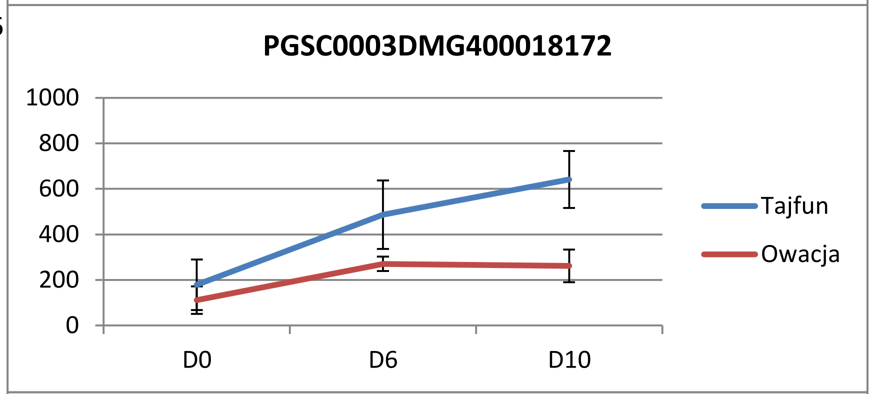

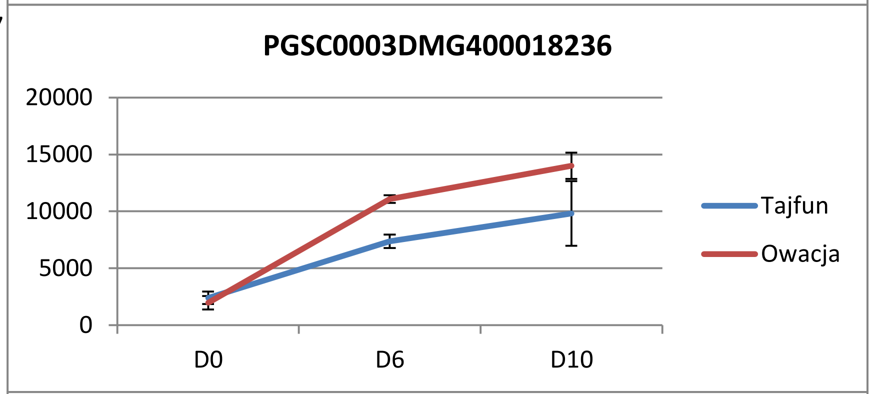

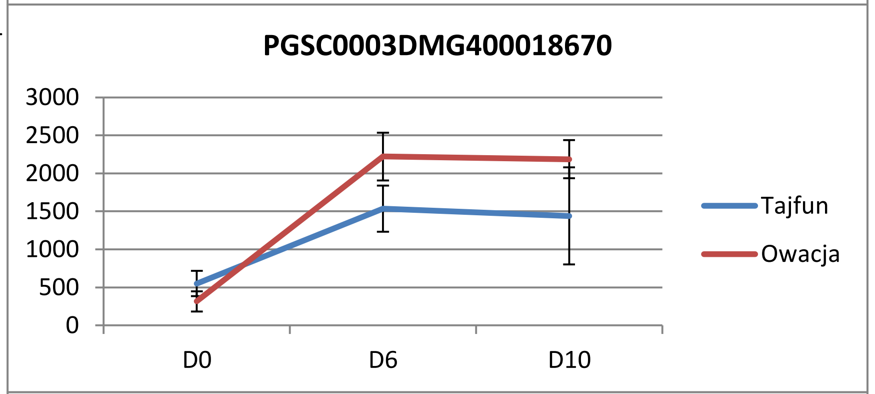

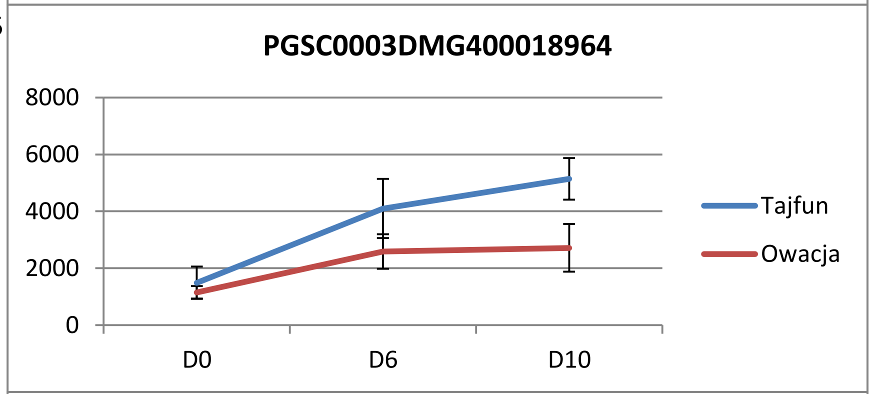

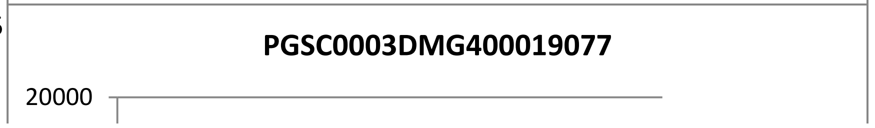

**PGSC0003DMG400019233**	187,33	962	1014	191,33	363,33	151,33	122,81	150,21	93,74	37,17	60,08	61,85
**PGSC0003DMG400019270**	1427,33	6622	4271,33	926,67	2729,3	542	400,25	1329,4	525,63	204	227,39	74,08
**PGSC0003DMG400019288**	1168,67	8676	6446	787,33	6403,3	2226,7	254,01	431,18	1002,2	240,47	84,13	191,69
**PGSC0003DMG400019315**	1128,33	8415,3	10216,3	740	12724	25663	372,69	361,63	5323,4	415,12	2080,3	3551,3
**PGSC0003DMG400019604**	69,67	1163,3	980	105,33	2846,7	2168,3	48	170,46	232,31	25,17	636,52	932,55

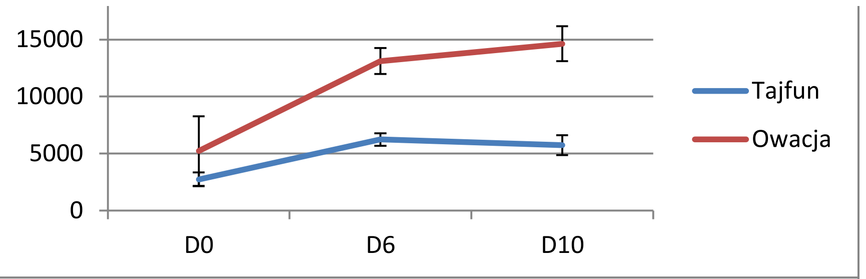

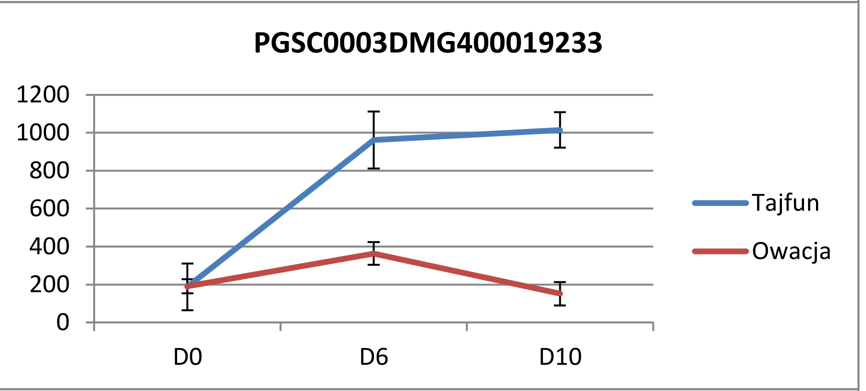

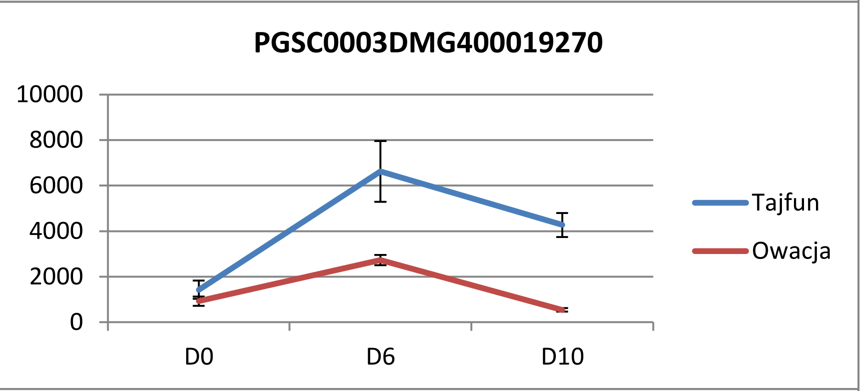

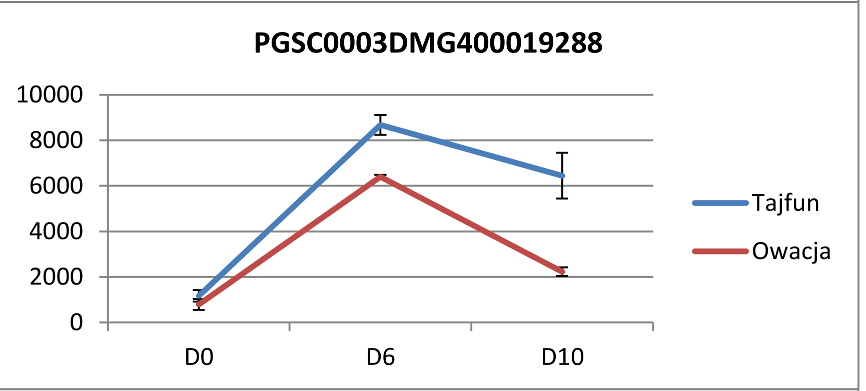

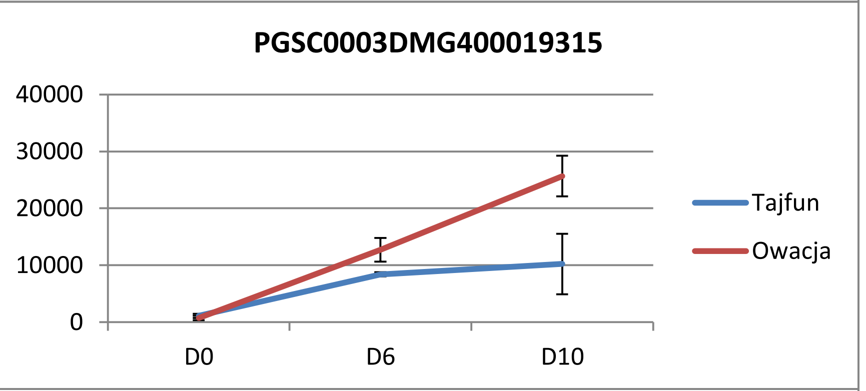

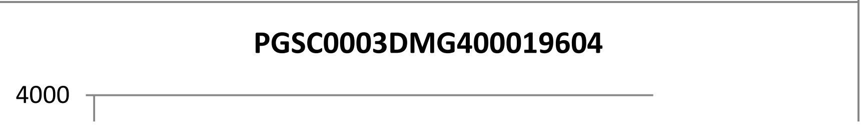

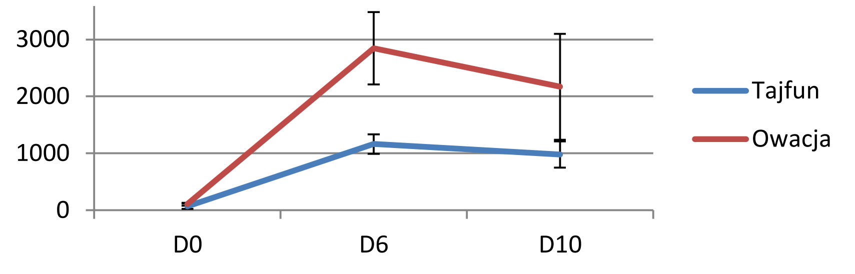

**PGSC0003DMG400019829**	178,67	574,67	642,67	126,67	364,67	170,67	170,66	43,47	39,11	32,02	44,56	58,97
**PGSC0003DMG400019944**	230,67	967,33	10494,7	218	1618	24209	84,67	62,36	9816,4	81,46	143,09	5123,49
**PGSC0003DMG400019952**	3,33	26	680,67	8	253,33	2120	5,77	5,29	544,65	5,29	31,01	419,73
**PGSC0003DMG400020172**	1084	5992,7	3465,67	804,33	4170,7	1615	226,11	303,58	204,84	205,22	491,08	207,28
**PGSC0003DMG400020248**	2092,67	5705,3	14486,7	2848	11689	24862	165,56	838,21	5933	591,37	2993,5	3433,5

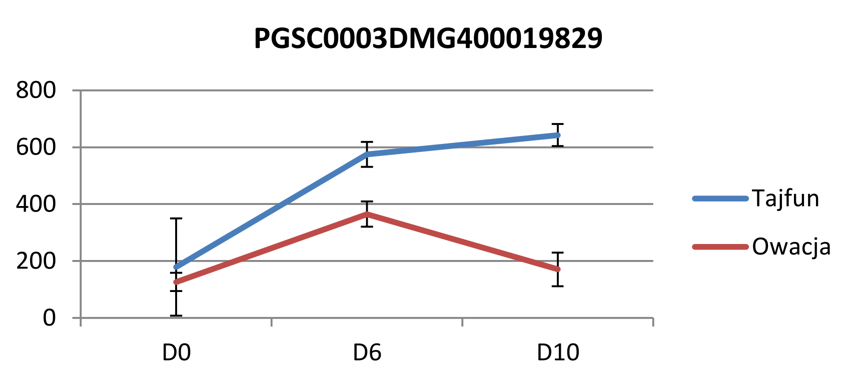

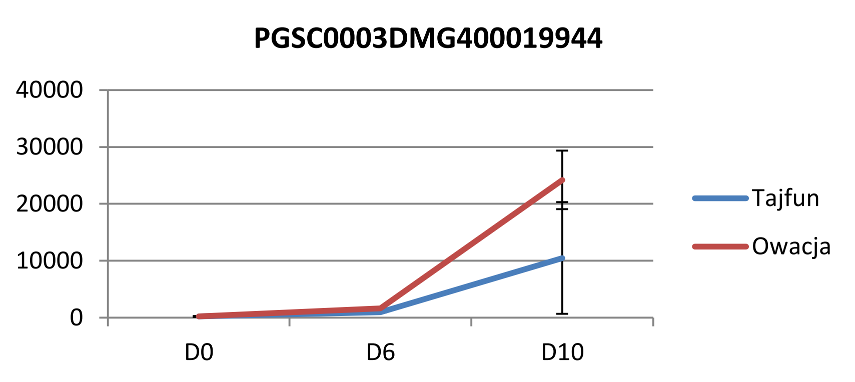

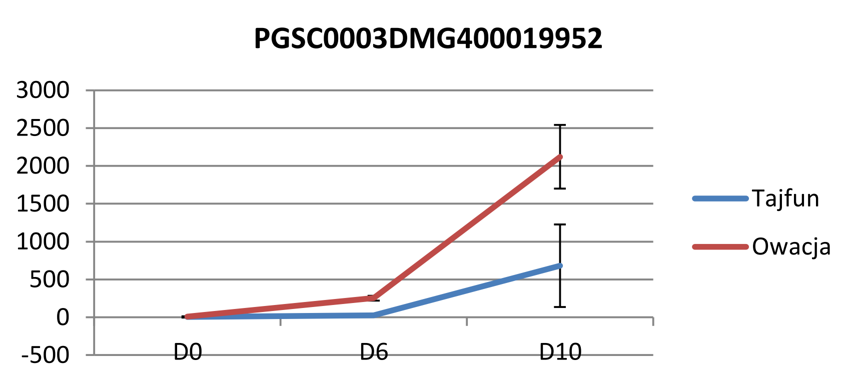

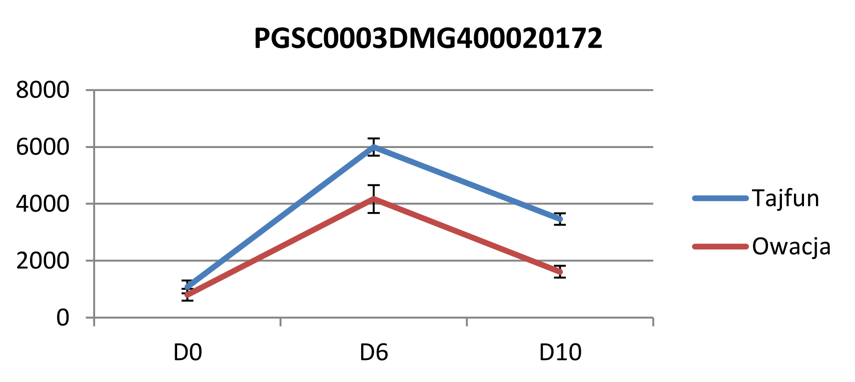

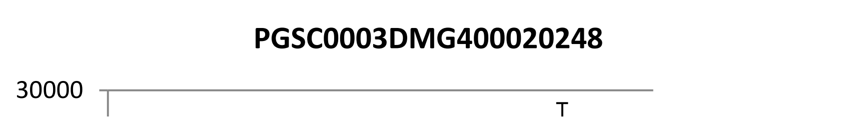

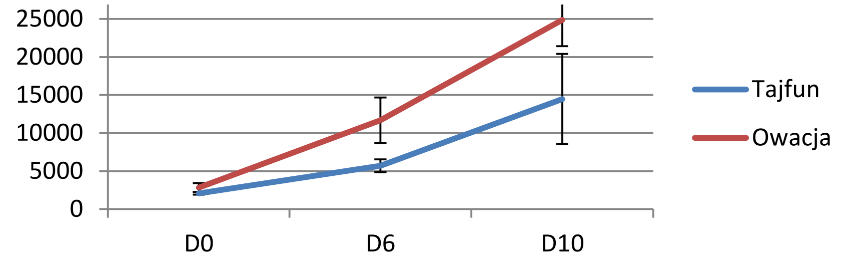

**PGSC0003DMG400020542**	1220	2617,3	2621,33	968,67	1903,3	1379,3	232,4	302,67	302,14	215,22	110,82	242,18
**PGSC0003DMG400020603**	552,33	1128	1151	285,33	458	432	246,5	41,9	270,24	112,22	34,18	57,09
**PGSC0003DMG400020620**	863,33	2414,7	2915,33	780,67	4250,7	6892	181,3	313,13	1184,3	482,33	598,54	98,97
**PGSC0003DMG400021039**	408,67	2178,7	9366	442,67	6059,3	20715	52,62	301,49	3064,5	88,55	400,55	2423,24
**PGSC0003DMG400021158**	3359,33	6069,3	7444	4096,67	4512,7	3968	427,29	729,32	1924,8	303,43	127,35	537,81

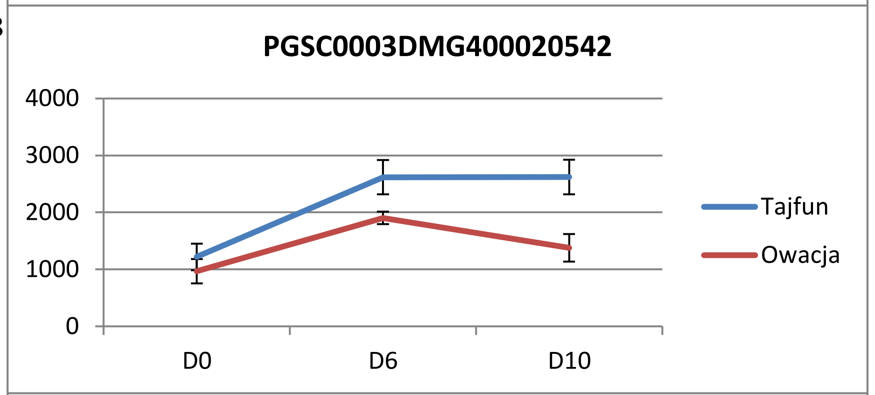

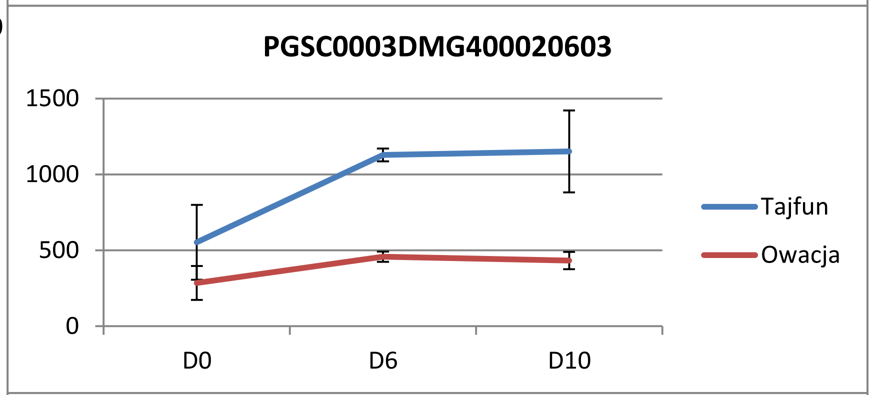

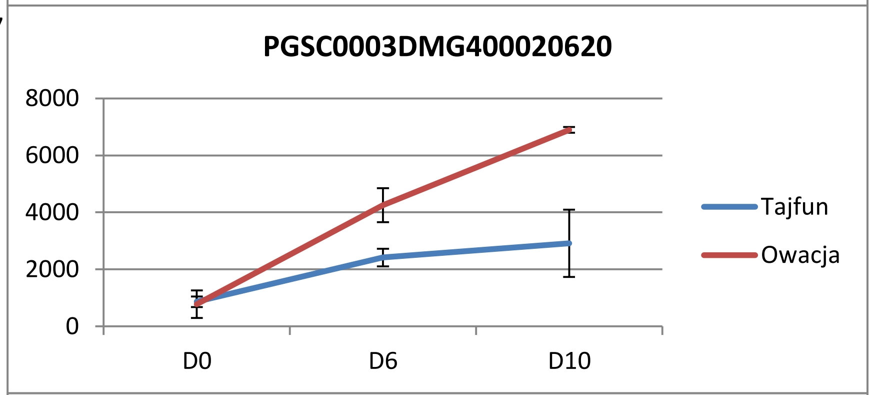

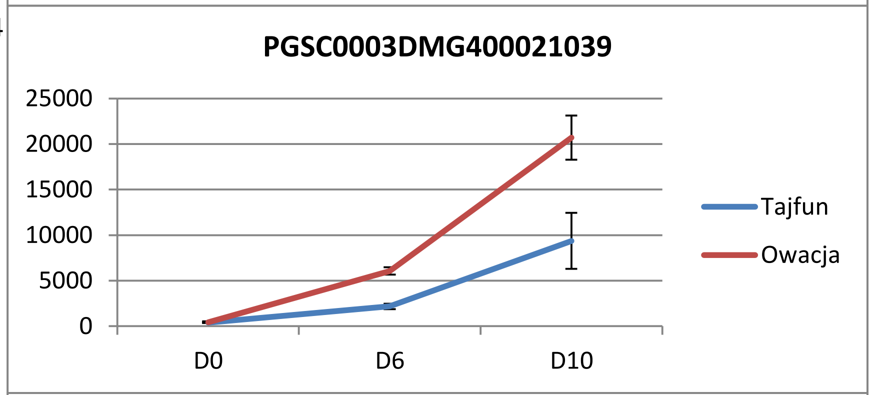

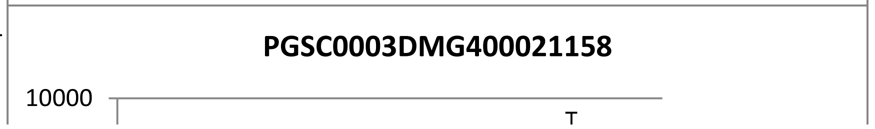

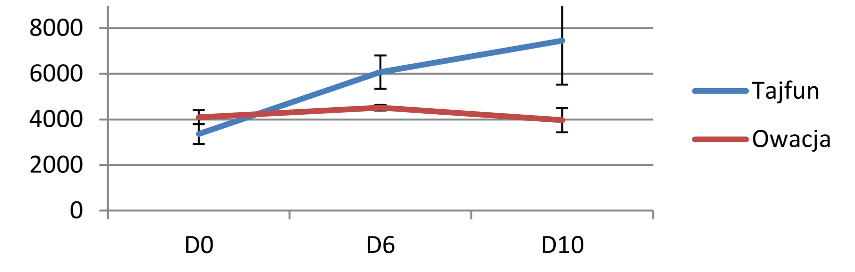

**PGSC0003DMG400021177**	266	854	644	132	547,33	278,67	117,78	85,06	115,31	71,53	35,85	82,01
**PGSC0003DMG400021346**	25,33	129,33	137,33	44	257,33	299,33	26,03	36,3	62,43	29,46	43	135,44
**PGSC0003DMG400021506**	590	1564	2799,33	807,33	2732,7	5010	163,41	236,58	1415,5	166,06	384,22	547,07
**PGSC0003DMG400022134**	1398,67	3157,3	9540	1028,67	4773,3	15647	404,25	1013,1	2809	186,72	544,3	1389,74
**PGSC0003DMG400022256**	2524,33	6988,3	13331,3	1783,33	3178,7	6825,3	583,28	230,44	4362	382,21	332,42	433,19

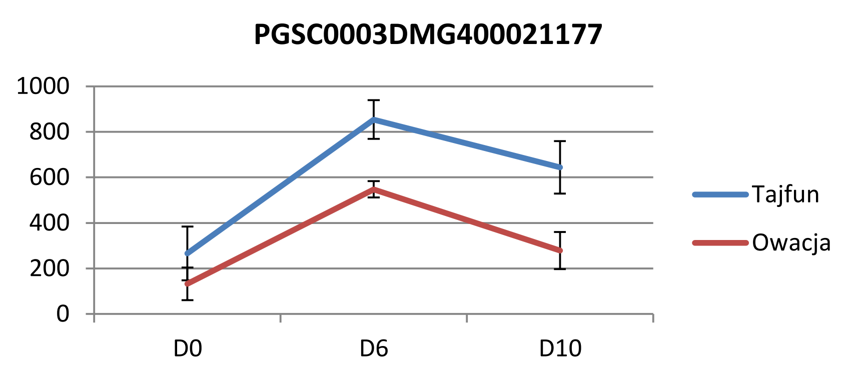

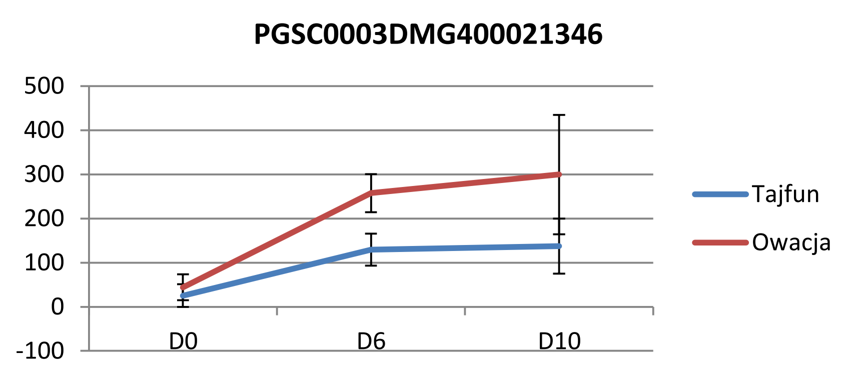

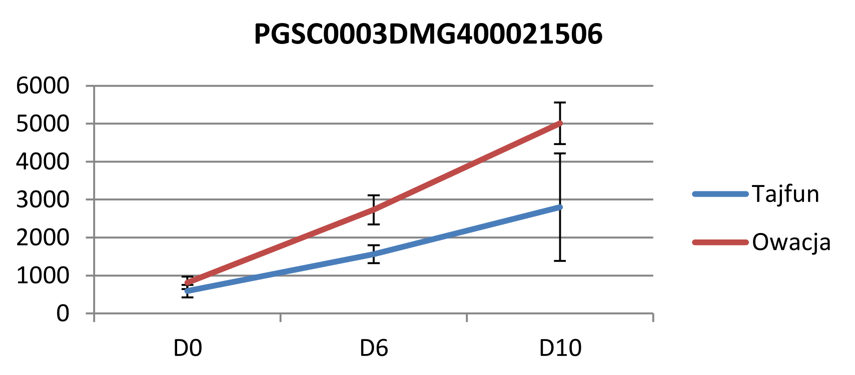

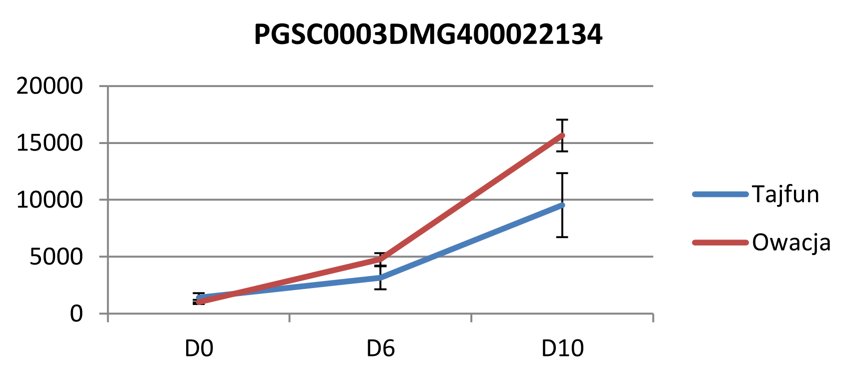

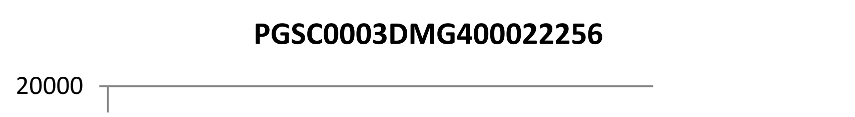

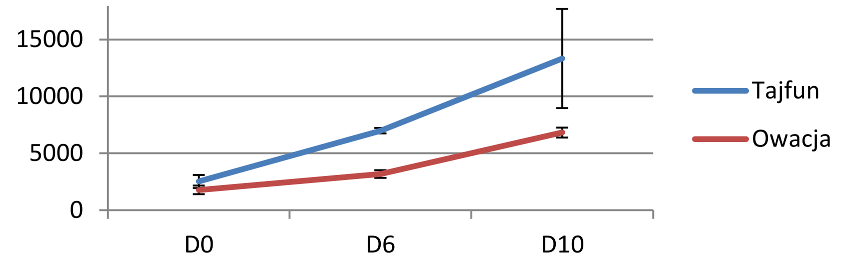

**PGSC0003DMG400022257**	7,33	348,67	383,33	6,67	121,33	28,67	6,43	61,78	82,4	1,15	25,32	6,43
**PGSC0003DMG400022408**	48,67	232,67	143,33	34,67	54,67	32	16,17	13,61	34,49	11,02	14,74	13,11
**PGSC0003DMG400022635**	615,33	2427,3	4064,67	632,67	3672,7	8398,7	117,46	197,02	802,4	282,06	1001	2326,89
**PGSC0003DMG400022641**	21,33	71,33	170	5,33	28,67	58,67	18,9	5,77	51,07	9,24	25,32	27,3
**PGSC0003DMG400022844**	21,33	112	136,67	12,67	30	36,67	5,03	26,23	23,01	5,03	15,1	11,37

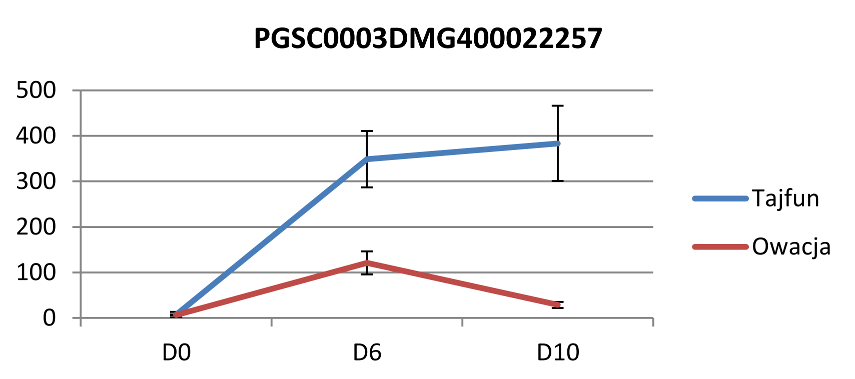

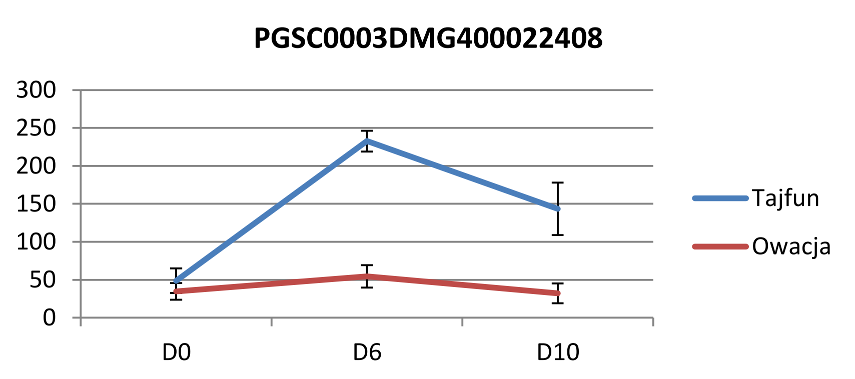

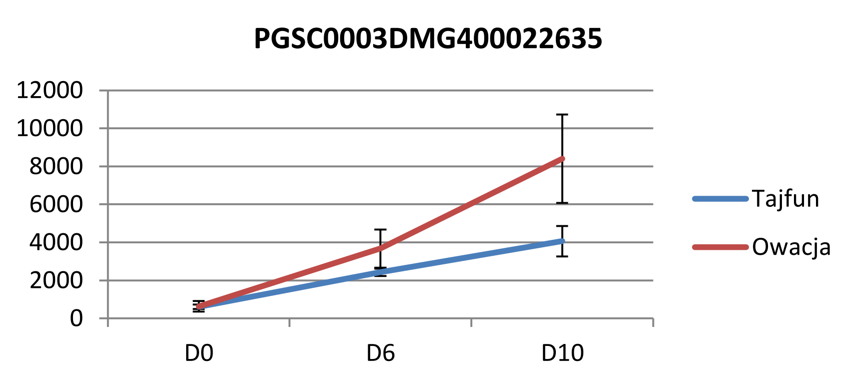

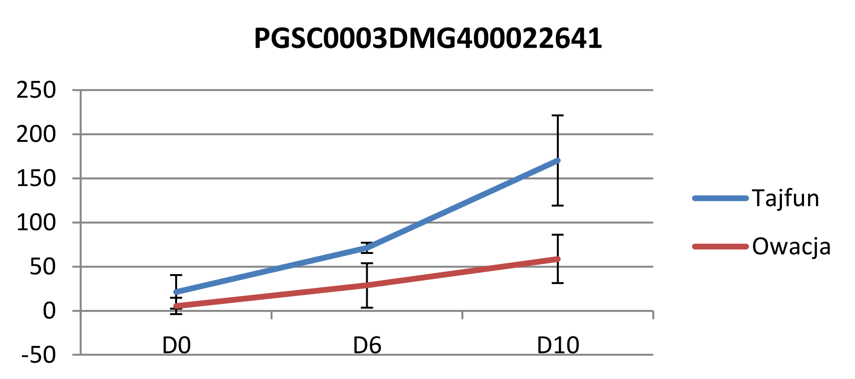

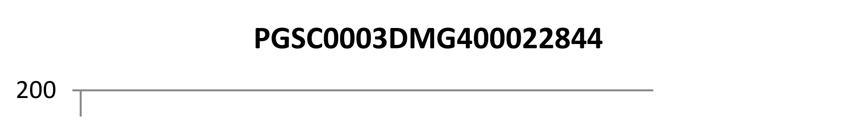

**PGSC0003DMG400022898**	562	1570	2149,33	622,67	2295,3	2968	91,65	167,22	616	227,62	239,37	173,59
**PGSC0003DMG400022950**	222	976	1781,33	224	1494	3391,3	122,52	153,26	1209,9	87,18	362,86	311,44
**PGSC0003DMG400022976**	435	871,67	993	294,67	547,33	362	130,66	142,48	157,04	19,63	168,49	8,89
**PGSC0003DMG400023453**	3662,67	13541	12042	2713,33	8696,7	5480,7	1123	946,61	2851	775,48	617,82	909,18
**PGSC0003DMG400023726**	106	332,67	296	74	145,33	89,67	26,15	53,15	100,46	32,19	12,5	21,08

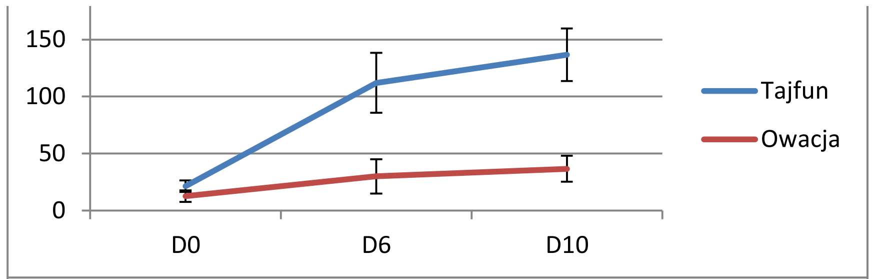

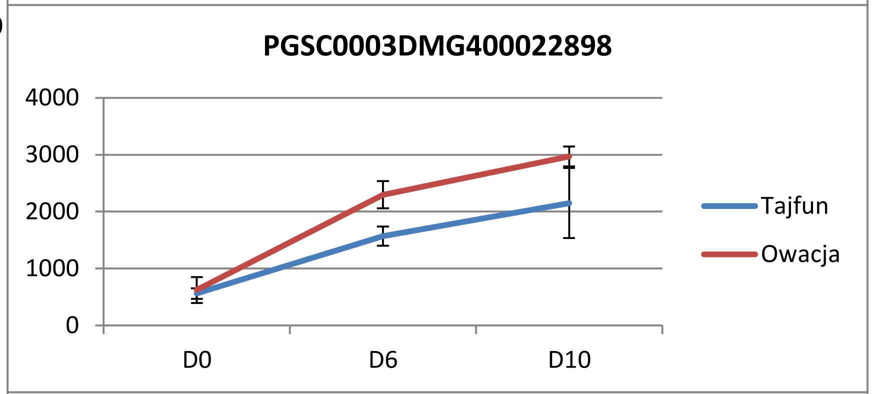

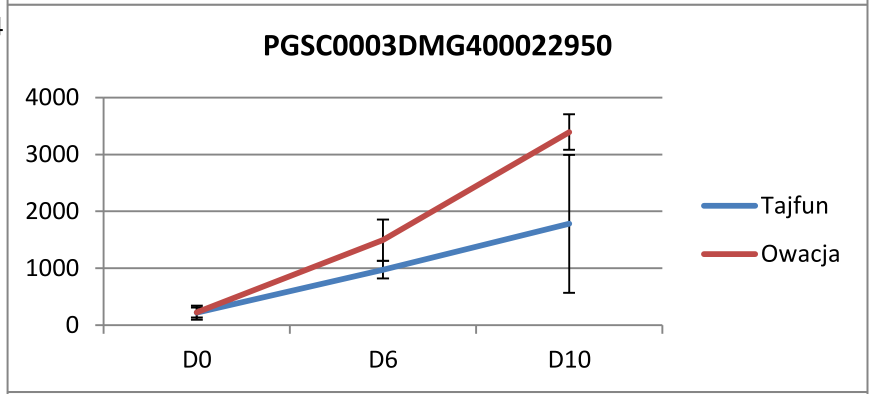

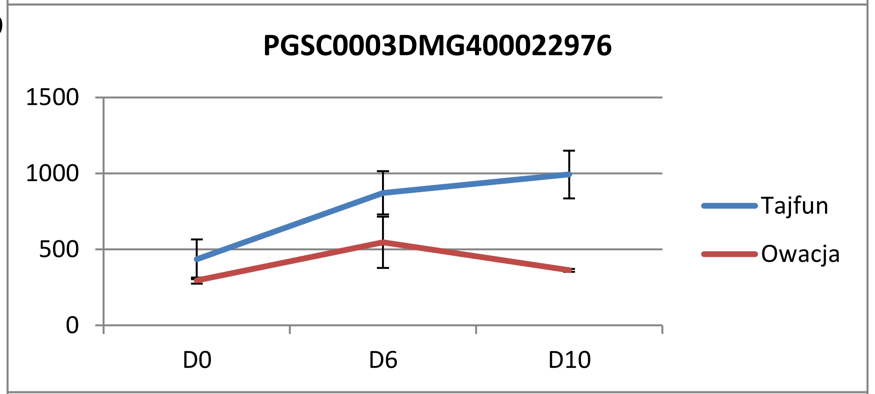

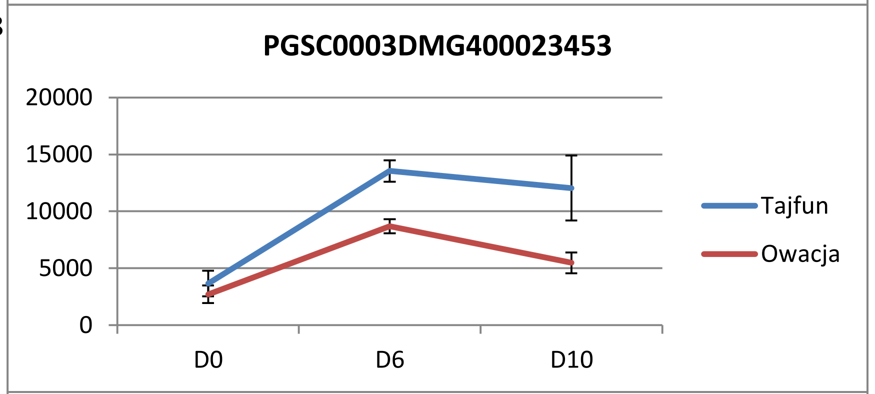

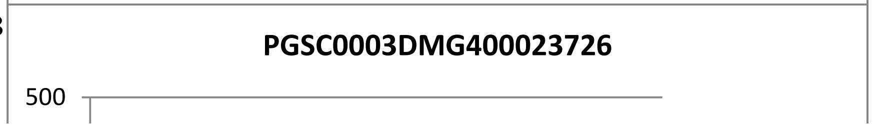

**PGSC0003DMG400023771**	11186	26657	32468	9689,33	38548	51276	2499,6	2129,6	12582	2826	5564,6	3632,19
**PGSC0003DMG400023945**	467,33	1528,7	3044,67	572,67	2023,3	1516	86,01	192,93	466,07	18,04	17,01	325,37
**PGSC0003DMG400023979**	88	1133,7	6516,67	144,67	1674,7	2511	81,19	273,35	2114,5	129,56	217,49	405,12
**PGSC0003DMG400024093**	23,33	3823,3	4459,33	9,33	672,67	390,67	5,03	905,54	66,16	3,06	156,43	130,79
**PGSC0003DMG400024860**	40,67	1156,7	2760	24	1873,3	4369,3	20,03	157,74	1637,8	17,44	211,68	1162,19

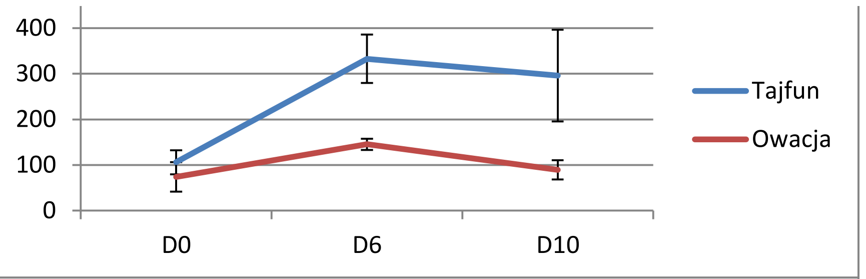

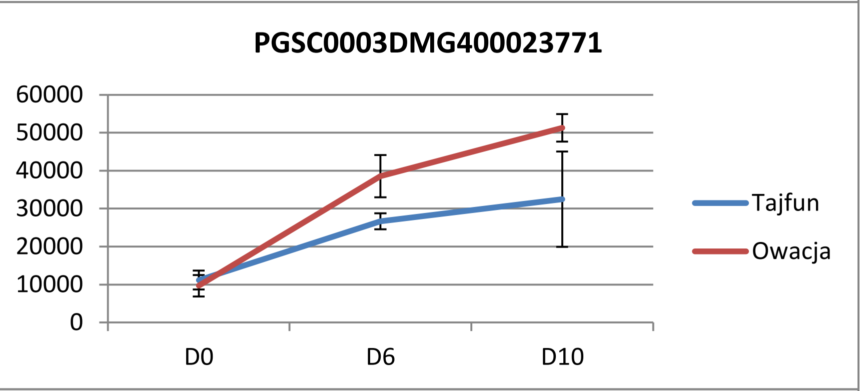

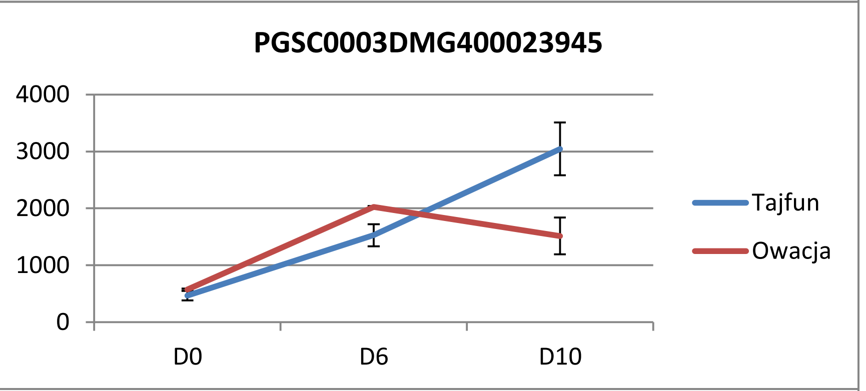

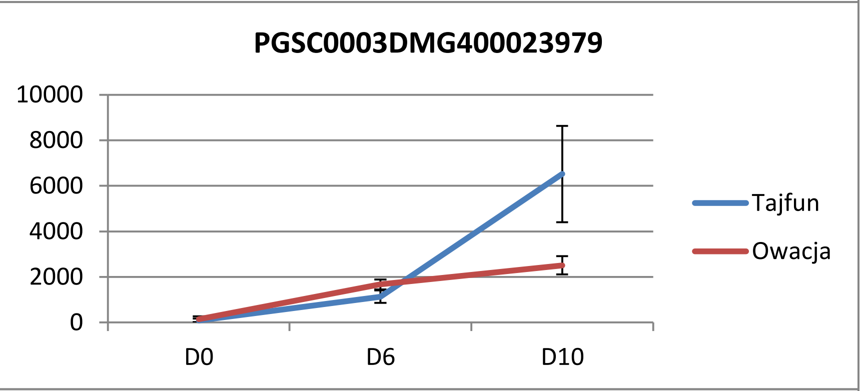

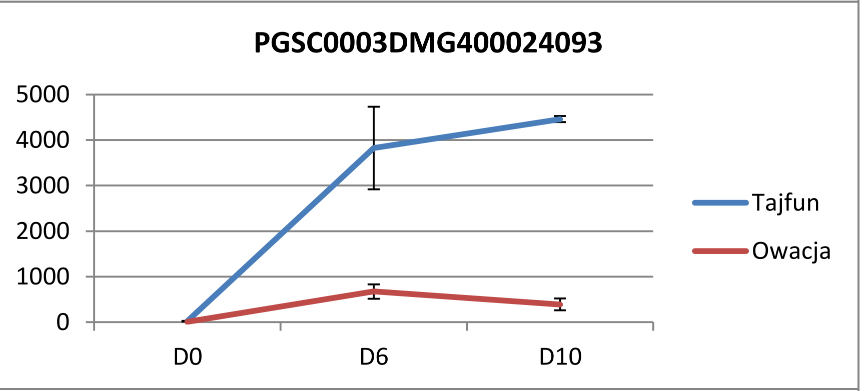

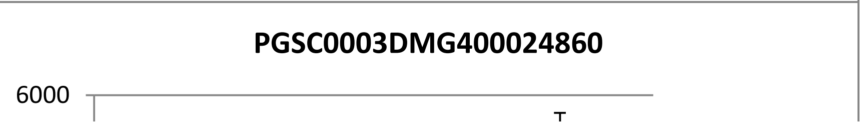

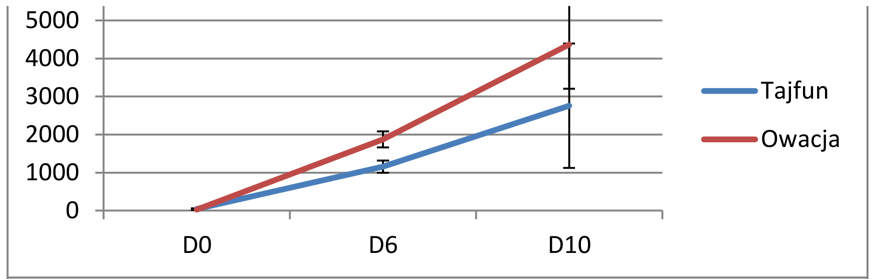

**PGSC0003DMG400024929**	1952,67	3831,3	4170	2058	2584,7	1675,3	335,63	661,11	647,56	772,35	380,15	258,66
**PGSC0003DMG400025154**	2117,33	3950	5724,67	1443,33	2608,7	2682	347,1	629,14	329,22	257,64	264,59	814,77
**PGSC0003DMG400025223**	354,67	734	982,67	420	1232	1516	32,88	43,41	165,44	144,04	28	92,97
**PGSC0003DMG400025798**	604	1102	2022	693,33	1841,3	3304,7	182,79	97,75	672,72	129,06	314,49	50,29
**PGSC0003DMG400025852**	651,33	1246	1930,67	450,67	717,33	521,33	94,71	42,76	513,47	48,01	142,9	110,37

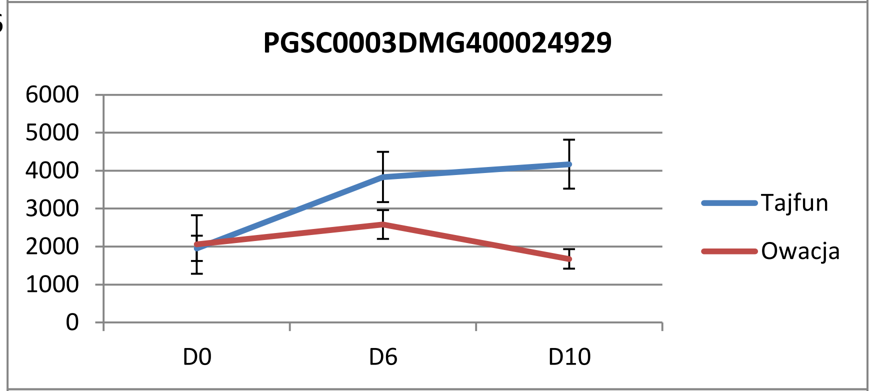

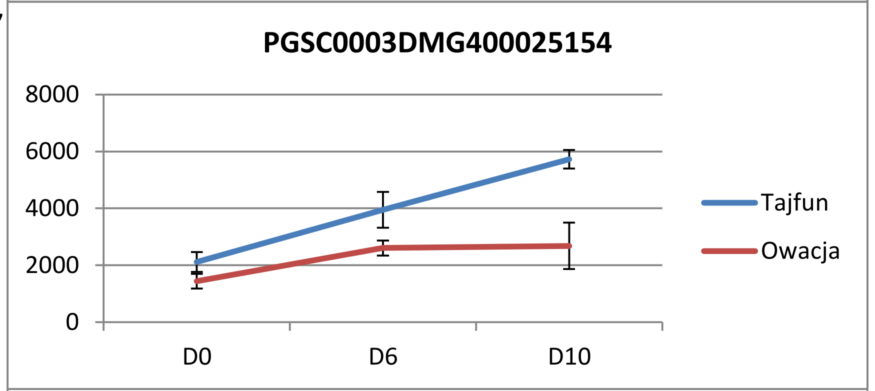

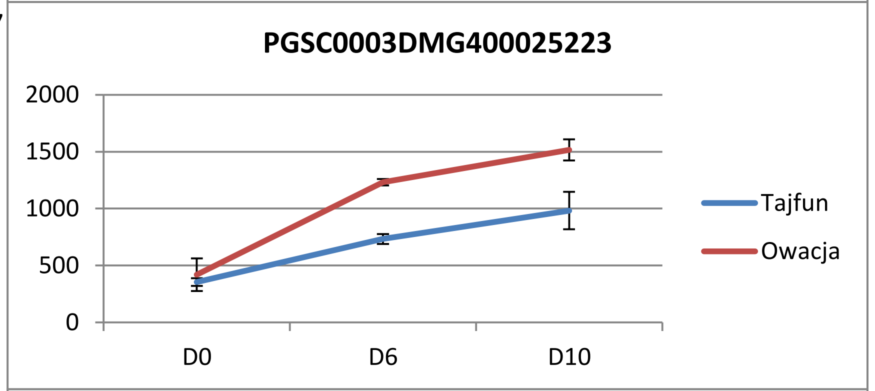

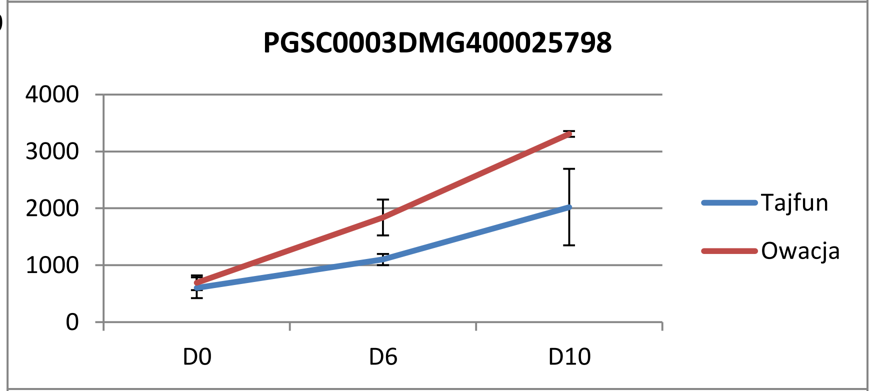

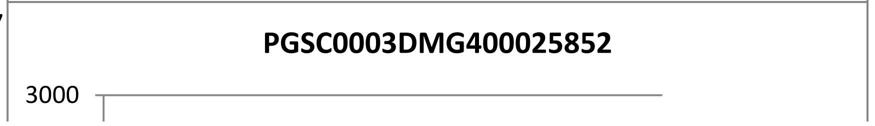

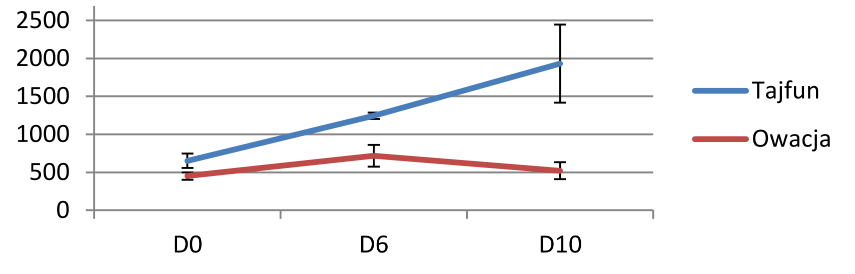

**PGSC0003DMG400025888**	969,33	3640,7	7650	1081,33	6255,7	10496	90,78	406,43	2020,2	132,07	413,68	357,79
**PGSC0003DMG400026262**	1604	2954,7	3234,67	1262,67	1401,3	1332,7	440,8	389,69	308,61	94,71	83,58	67
**PGSC0003DMG400026359**	193	689,33	1373,33	225,33	1212,7	2690,3	22,87	131,37	459,19	92,03	459,52	888,23
**PGSC0003DMG400026361**	4549,33	9502	12070,7	4657,33	5952,7	4478	366,62	1016,1	2293,1	193,02	786,65	981,34
**PGSC0003DMG400026405**	167,33	764	1166,67	154	487,33	538	19,43	48	315,78	36,72	55,22	85,86

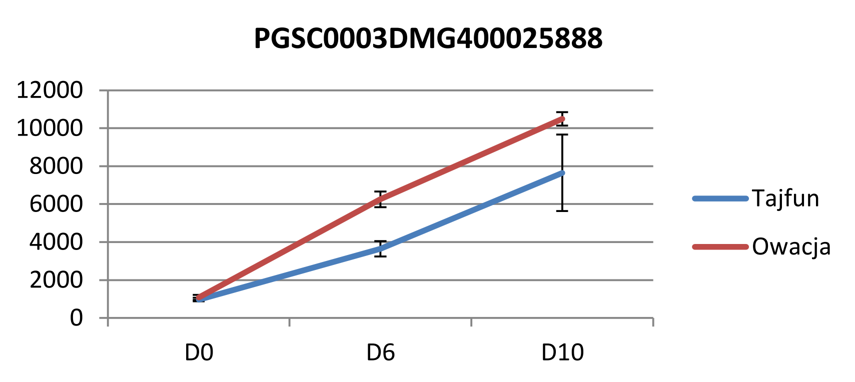

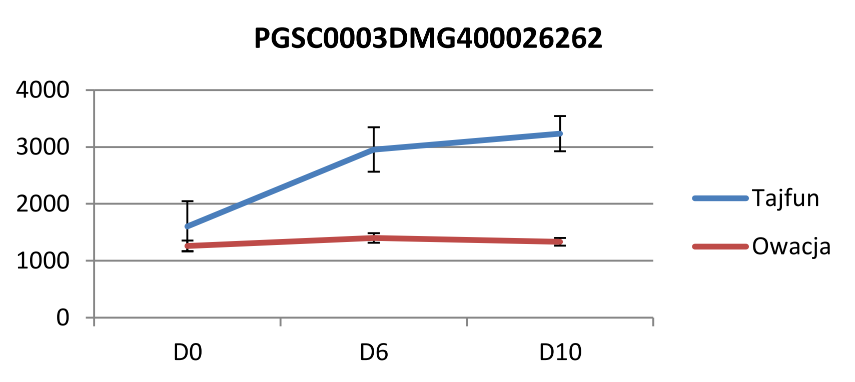

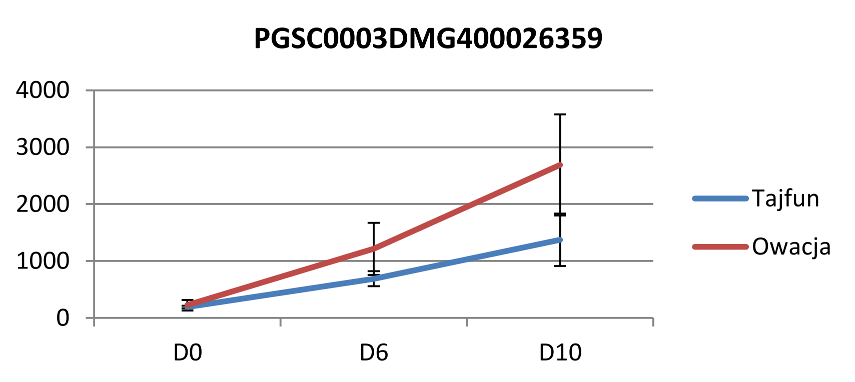

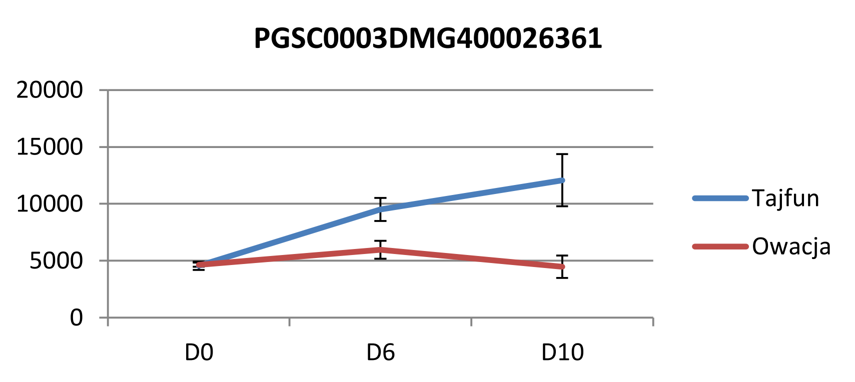

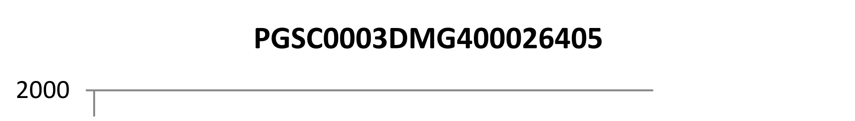

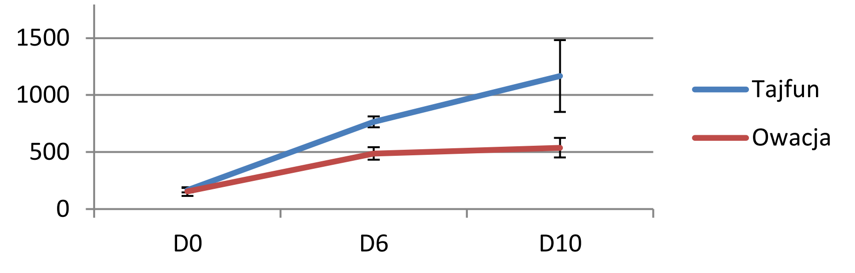

**PGSC0003DMG400026527**	3600	6580	6655,33	3479,33	9125,7	9444	1080,6	907,73	210,5	843,74	986,16	1477,43
**PGSC0003DMG400026646**	311,33	609,33	1353,33	394,67	911,33	2187,3	35,57	98,43	436,09	85,47	92,72	502,92
**PGSC0003DMG400026647**	227,33	529,33	1077,33	322,67	934	1778	34,78	107,04	299,63	51,16	80,22	397,64
**PGSC0003DMG400026778**	385,33	1724,7	2955,33	361,33	8280	11662	15,01	146,89	799,54	87,32	667,76	1789,26
**PGSC0003DMG400026818**	1761,33	3607,3	3898,67	1480,33	2542,7	2270	547,04	341,32	1072	550,76	305,03	508,78

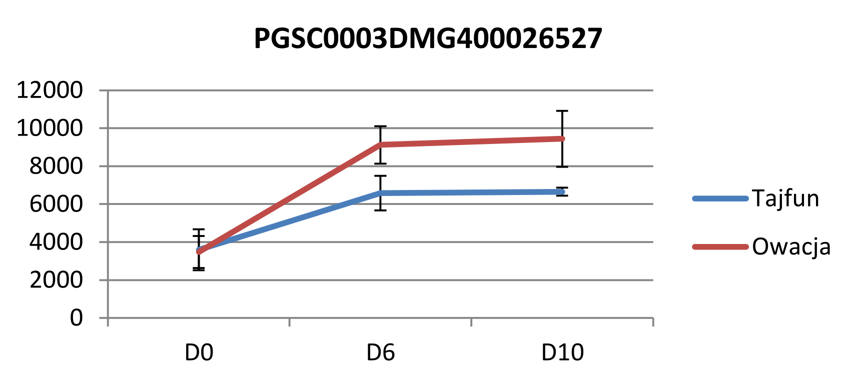

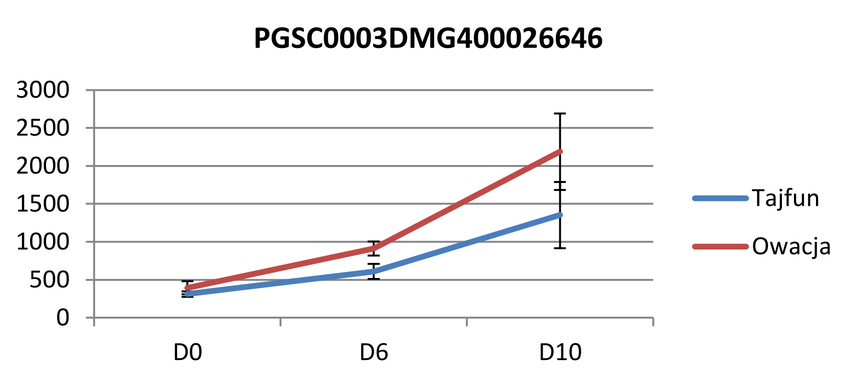

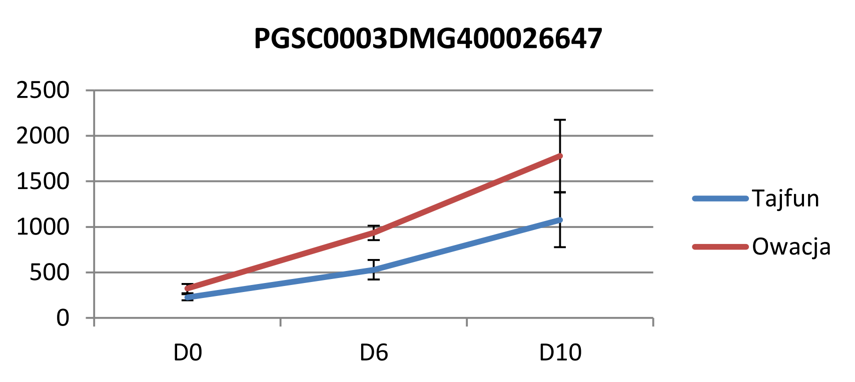

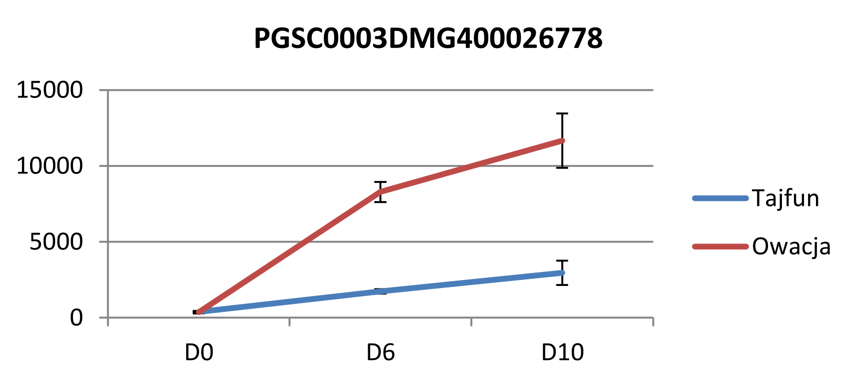

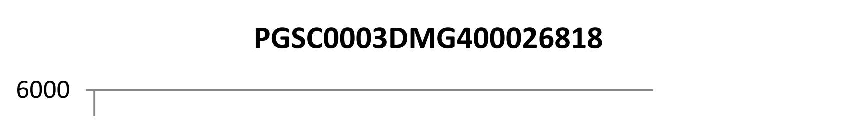

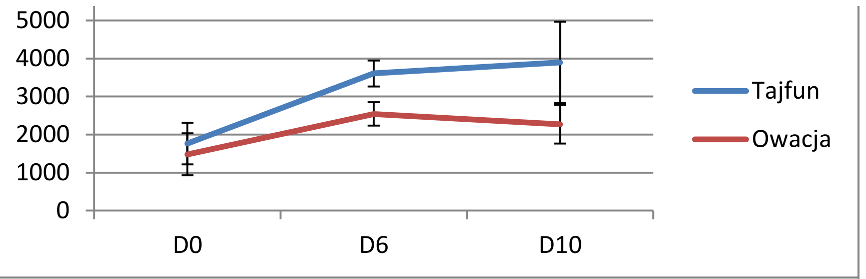

**PGSC0003DMG400027739**	2286,67	5685,3	6423,33	2742,67	9112,7	9678,7	415,43	750,45	1013,9	812,07	742,82	1097,02
**PGSC0003DMG400027743**	65,33	269,33	181,33	46	103,33	73,33	18,15	37,11	10,07	18,33	45,71	14,05
**PGSC0003DMG400027758**	153,33	362	578	220,67	934	1324,7	46,06	93,53	94,93	65,03	207,57	383,36
**PGSC0003DMG400028129**	186	560	430	142	281,33	223,33	10,39	55,24	46,6	43,86	44,47	20,03
**PGSC0003DMG400028166**	115,33	1158,3	252,67	70,67	468	8,67	53,08	133,43	21,39	18,15	45,21	15,01

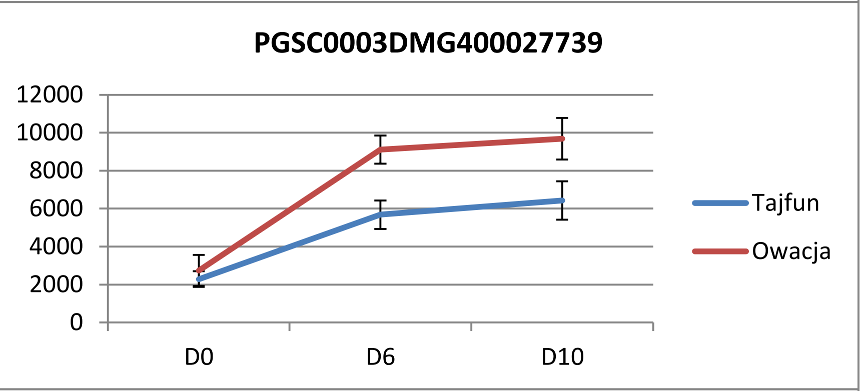

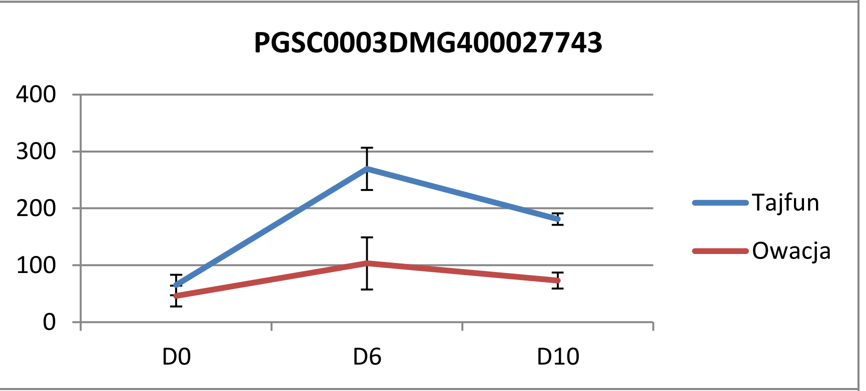

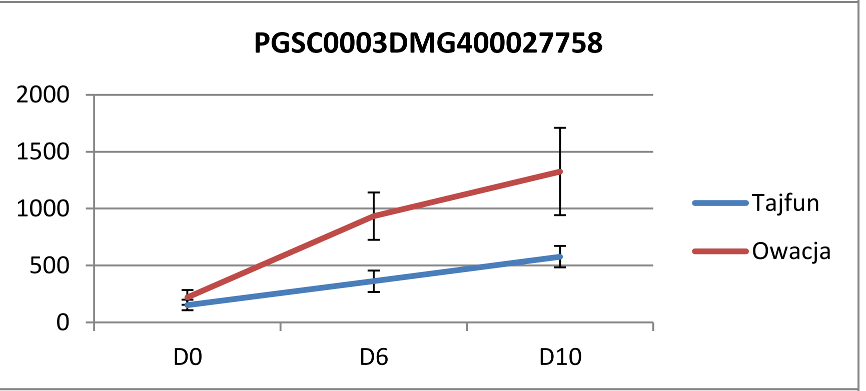

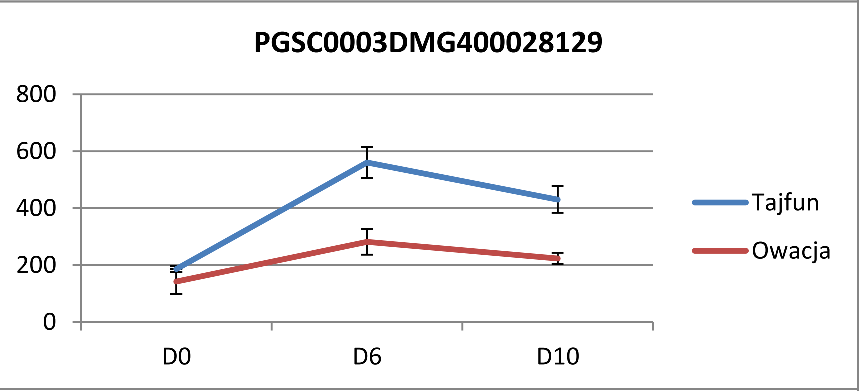

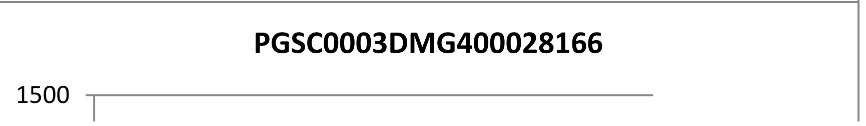

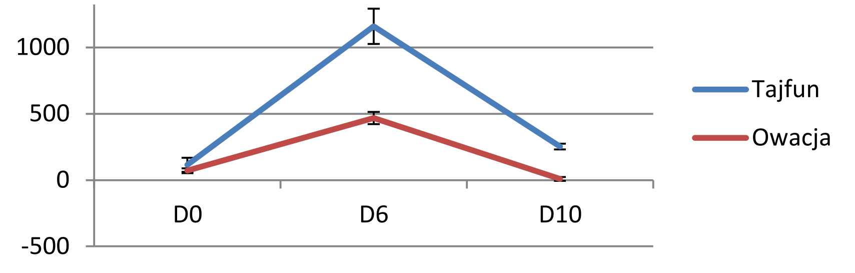

**PGSC0003DMG400028286**	175,33	977,33	1170,67	173,33	1761,3	697,33	62,14	96,67	242,83	26,1	165	101,61
**PGSC0003DMG400028364**	109,33	333,33	258,67	102	182	87,33	63,79	75,16	27,3	52,12	71,19	10,26
**PGSC0003DMG400028514**	1009,33	2888	2544,67	931,33	1958	1280	283,03	464,63	180,33	83,72	276,22	169,58
**PGSC0003DMG400028600**	26	228,67	356,67	24	138,67	137,33	45,03	28,73	103,47	41,57	54,2	28,94
**PGSC0003DMG400028635**	234,67	569,33	1166,67	204	1106	3680	23,18	155,77	845,97	39,95	165,78	1214,4

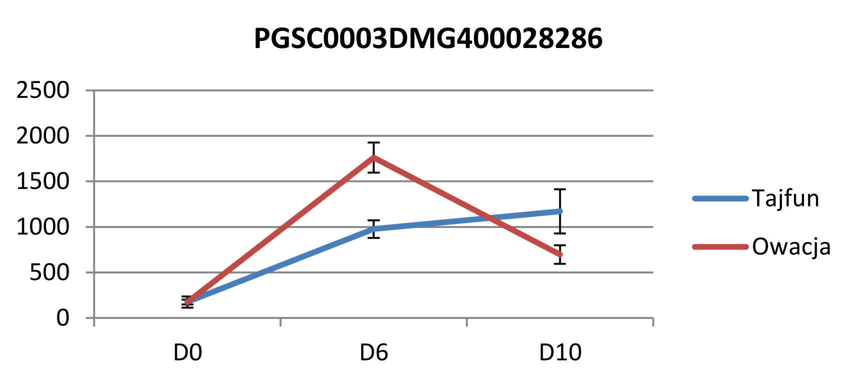

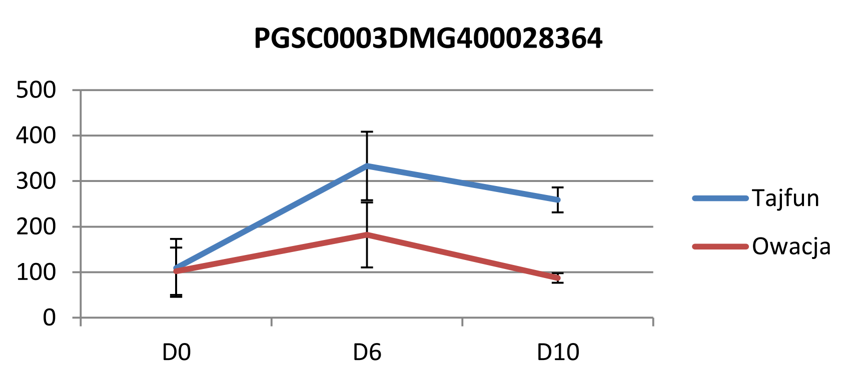

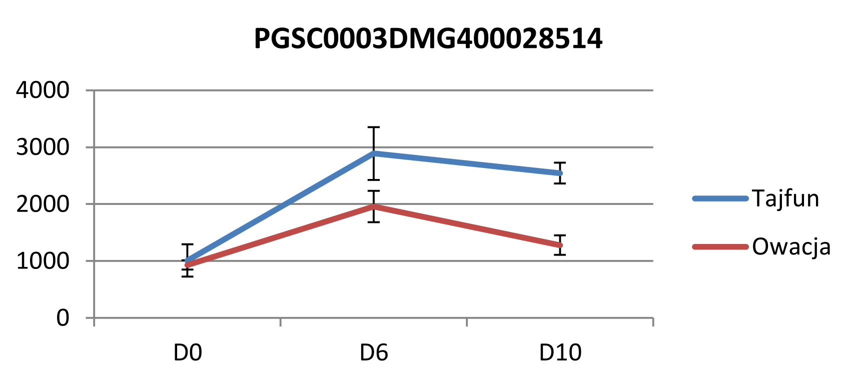

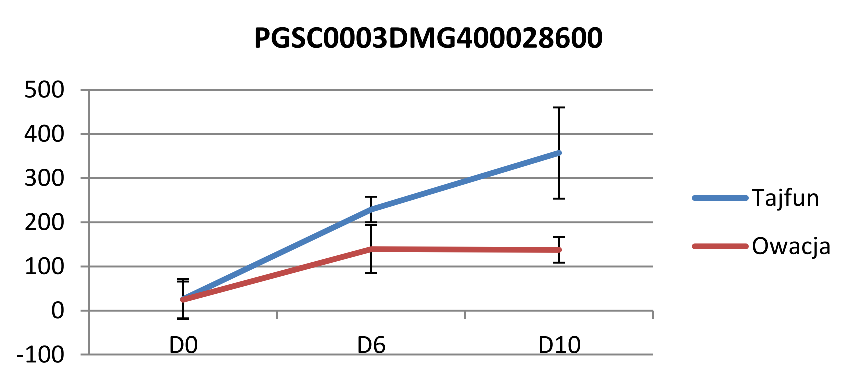

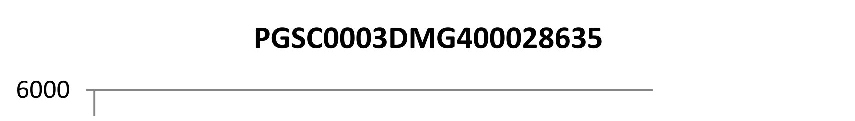

**PGSC0003DMG400028744**	434,67	820	1024	380	349,33	392	71,7	131,77	64,9	76,32	80,13	40,15
**PGSC0003DMG400028819**	524	7061,3	2096	350	2731,3	1044	161,15	397,12	631,02	125,2	140,3	107,68
**PGSC0003DMG400029074**	781,33	1714	3012,67	1257,33	2993,3	6078	55,15	190,78	877,81	310,91	281,94	1272,62
**PGSC0003DMG400029207**	1159,67	3293,7	6129,67	1029,33	1223,7	2005,3	365,74	950,68	3292,4	412,82	387,05	1384,72
**PGSC0003DMG400029396**	188,67	1940	566	217,33	1192	267,33	21,01	344,4	29,46	127,08	394,42	37,54

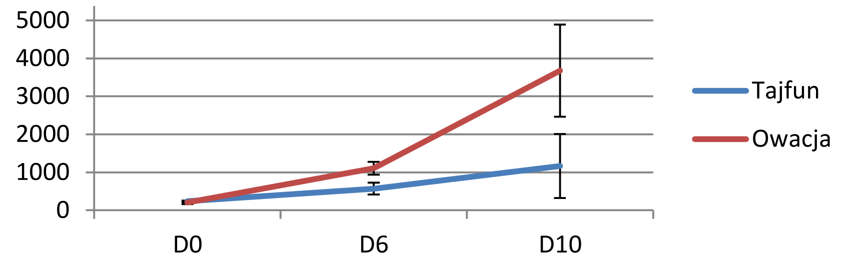

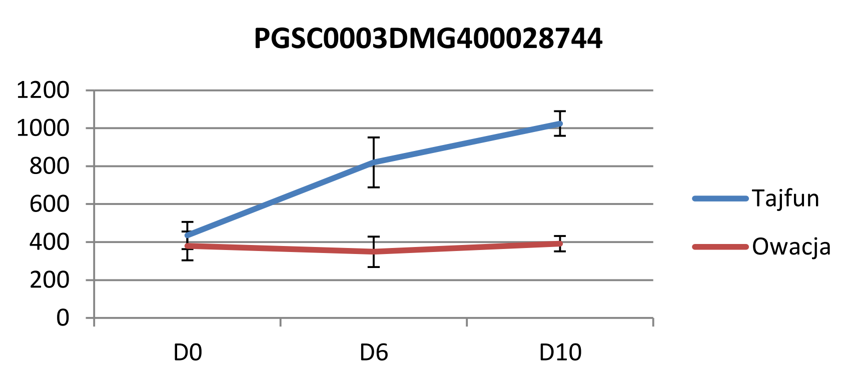

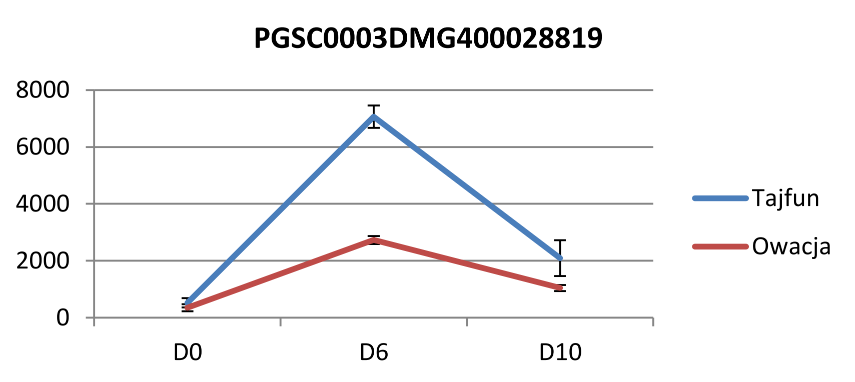

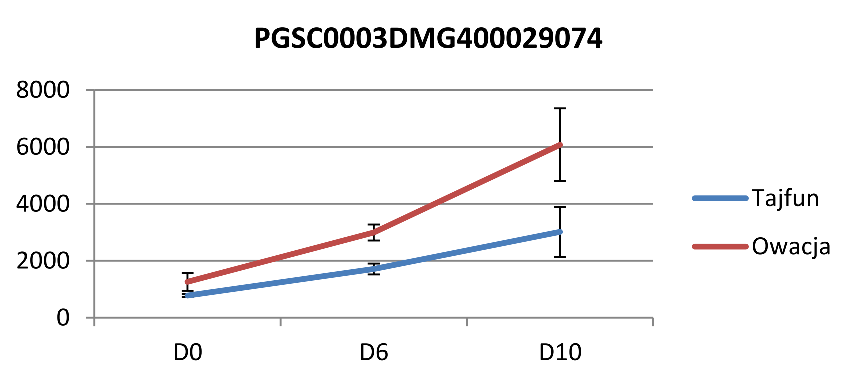

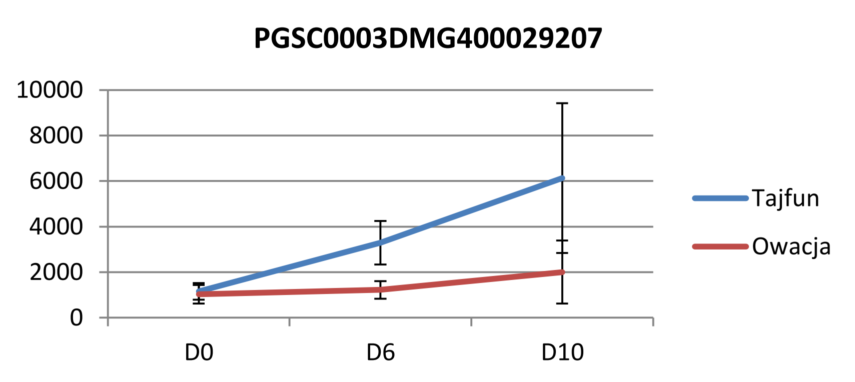

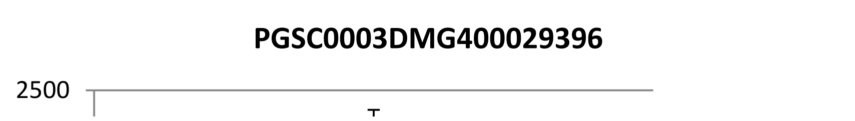

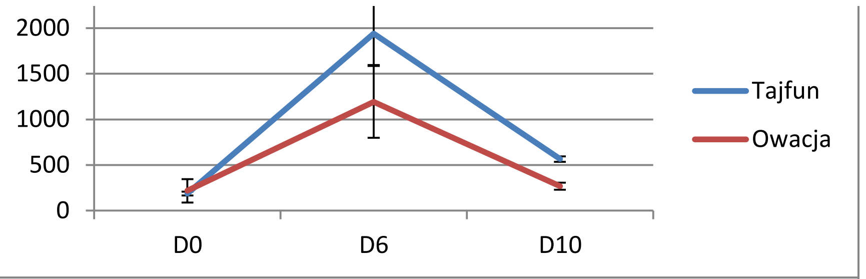

**PGSC0003DMG400029517**	3035,33	9693,3	28171,3	3007,33	20446	68051	176,19	621,93	16187	827,42	482,82	12260,5
**PGSC0003DMG400029557**	48	178	294	15,33	44,67	81	47,03	30,27	96,56	16,04	6,11	30,05
**PGSC0003DMG400029727**	170	2211,7	5671,33	67,33	361,67	800,67	163,45	1309	2821,8	40,86	82,1	340,1
**PGSC0003DMG400029732**	8484	38739	36219,3	7929,33	24672	9977,3	3679,8	6904,6	3462,1	3582,6	2523,1	596,93
**PGSC0003DMG400029841**	399,33	1029,3	2648	366	716,67	1519,3	221,31	151,4	97,02	121,8	13,01	278,29

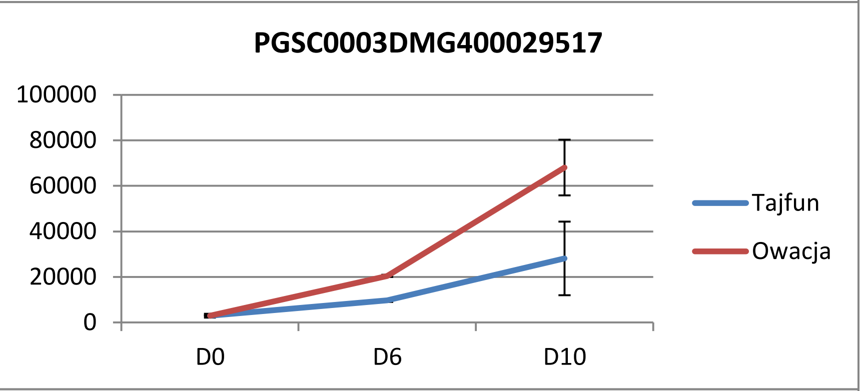

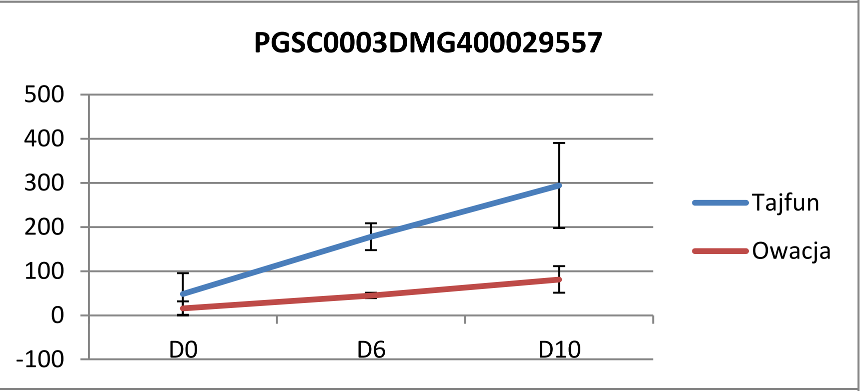

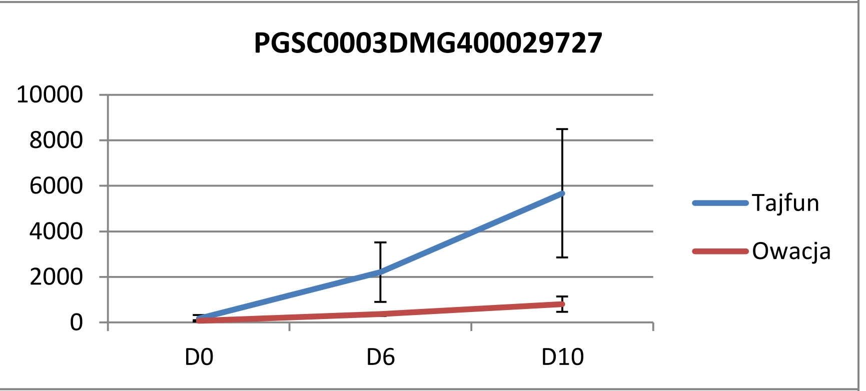

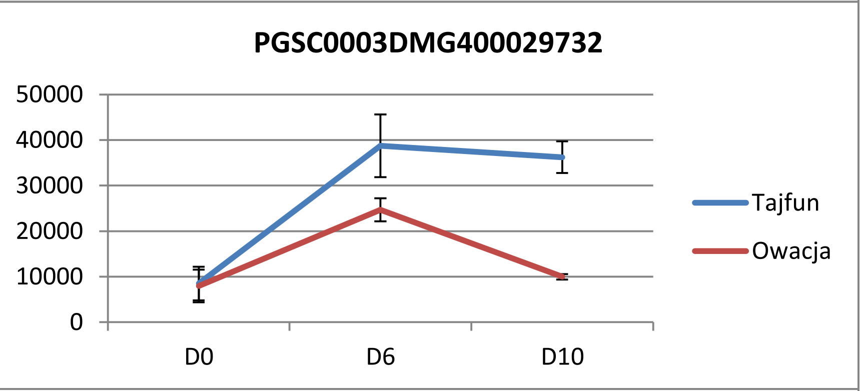

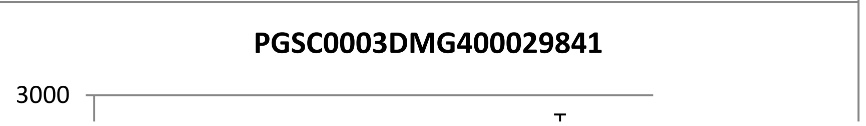

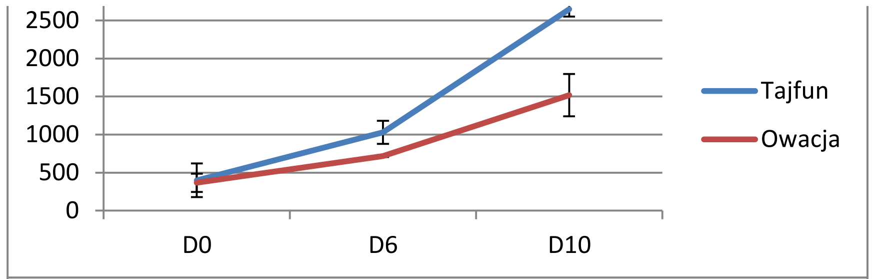

**PGSC0003DMG400029867**	375,33	854	846,67	411,33	1185,3	177,33	20,13	150,64	252,13	65,49	116,57	45,62
**PGSC0003DMG400029872**	218,67	798	2124	194,67	1192	4129,3	84,39	136,4	731,63	73,71	111,14	312,07
**PGSC0003DMG400030008**	14,67	962	827,33	0	2208,7	466,67	25,4	442,87	224,19	0	559,72	151,93
**PGSC0003DMG400030186**	637,33	1708	2389,33	557,33	1058	944	161,57	225,2	643,96	63,32	214,95	182,03
**PGSC0003DMG400030379**	866,67	2100	2140,67	668	1290	1002,7	81,05	137,88	268,96	117,93	60	156,75

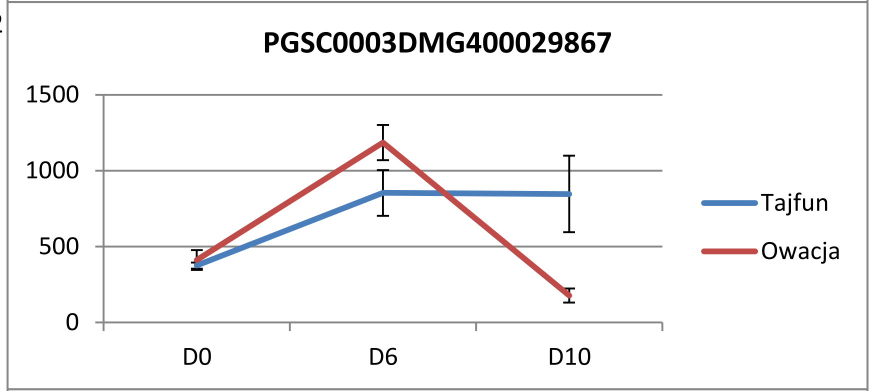

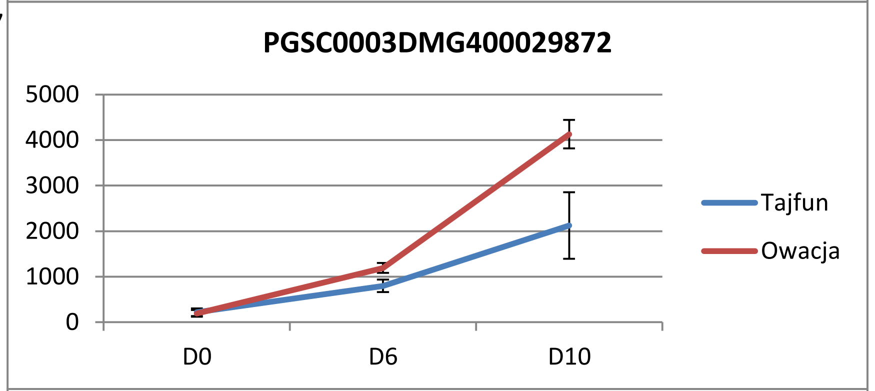

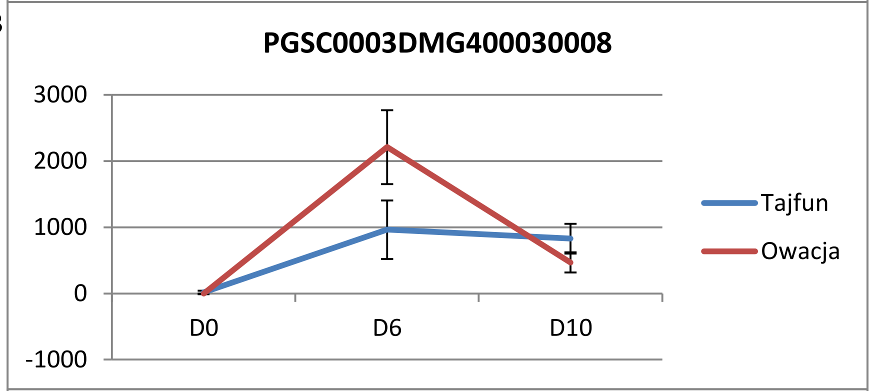

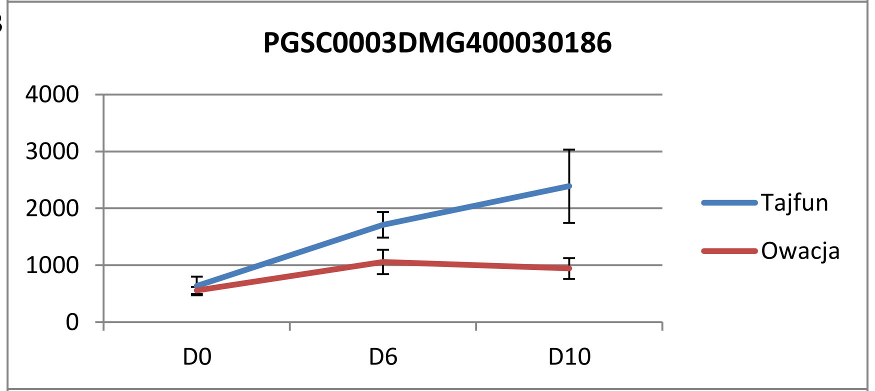

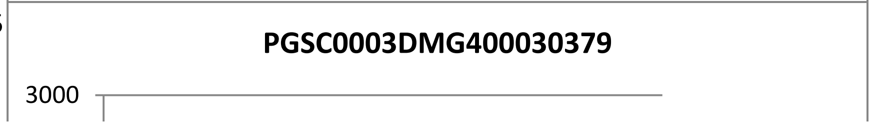

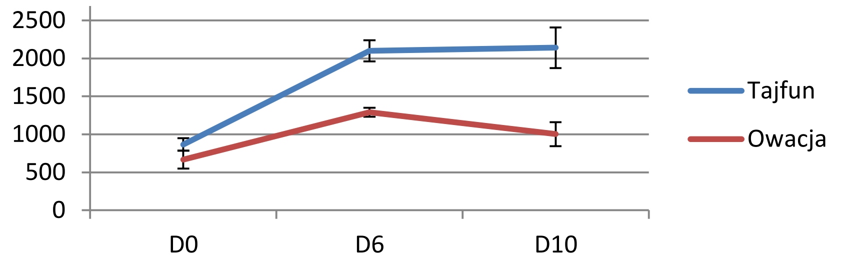

**PGSC0003DMG400030504**	288	690	1055,33	151,33	282	196	105,83	156,17	397,6	61,23	150,77	34,12
**PGSC0003DMG400030526**	340,67	952	904,67	325,33	607,33	349,33	81,05	63,59	158,31	85,47	113,04	95,21
**PGSC0003DMG400030617**	762,67	5535,3	7324,67	574,67	3368	1104	115,24	934,1	2361,3	55,51	397,88	114,07
**PGSC0003DMG400030635**	386,33	892	838,33	264,67	528,67	392,67	78,68	147,96	36,83	102,63	79,76	144,65
**PGSC0003DMG400030891**	1635,33	4733,3	22470	1708	12733	53769	175,62	226,29	14123	482,07	4004,9	11162,2

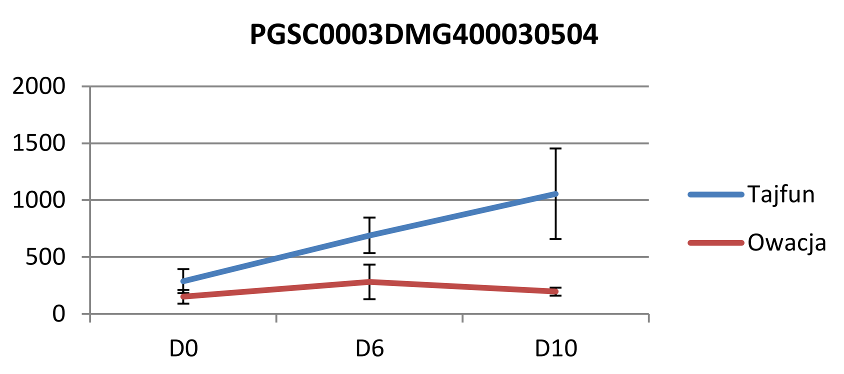

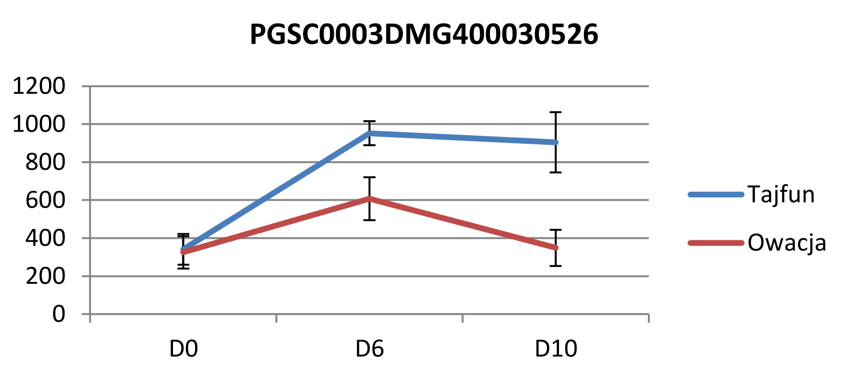

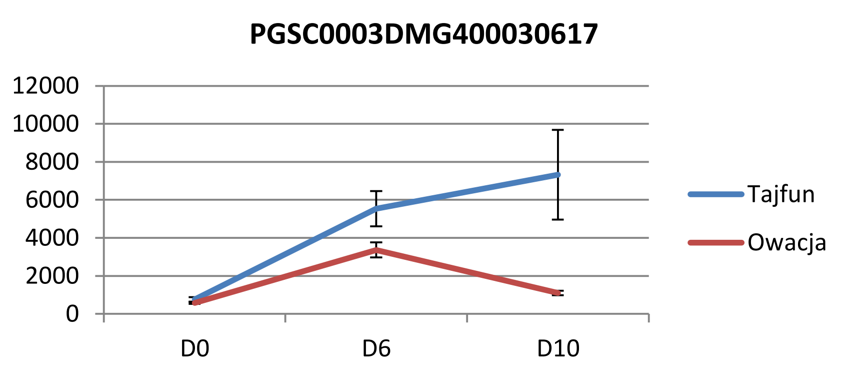

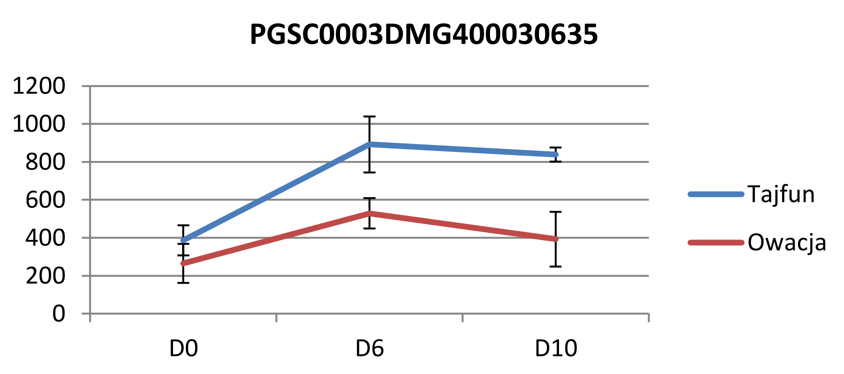

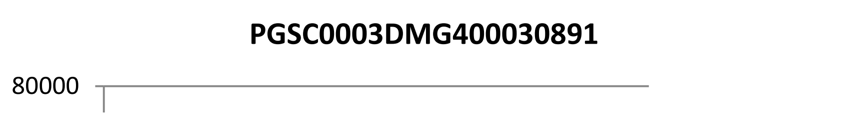

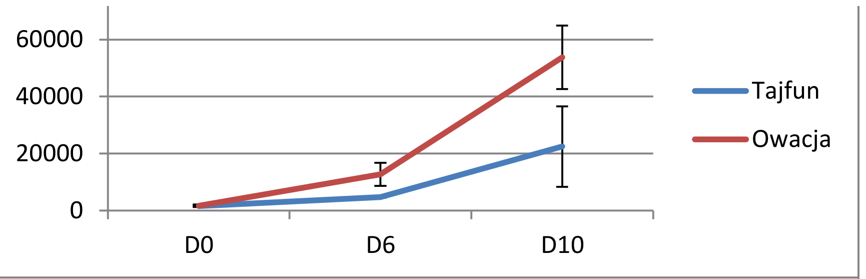

**PGSC0003DMG400031131**	4690	9151	13503	4160,33	13507	21043	370,36	792,02	1342,9	1531,4	1229,6	1269,44
**PGSC0003DMG400031213**	194,67	587,33	708,67	84,67	312,67	214	72,7	46,36	143,39	83,05	191,26	61,02
**PGSC0003DMG400031411**	1,33	492,67	1006	8	1535,3	1358,7	2,31	181,12	337,82	13,86	218,22	213,13
**PGSC0003DMG400031871**	1676	4408,7	4594	1154	2817,3	2287,3	133,28	718,59	295,7	62,35	195,36	330,49
**PGSC0003DMG400033140**	32,67	132	146,67	24	59,33	59,33	29,69	10	33,61	15,1	23,01	20,82

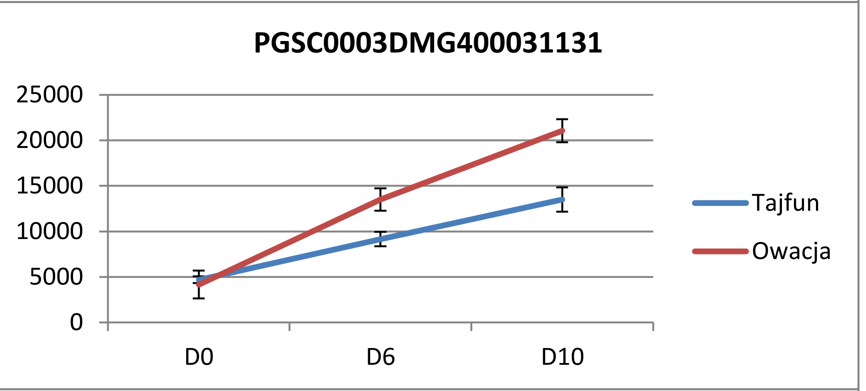

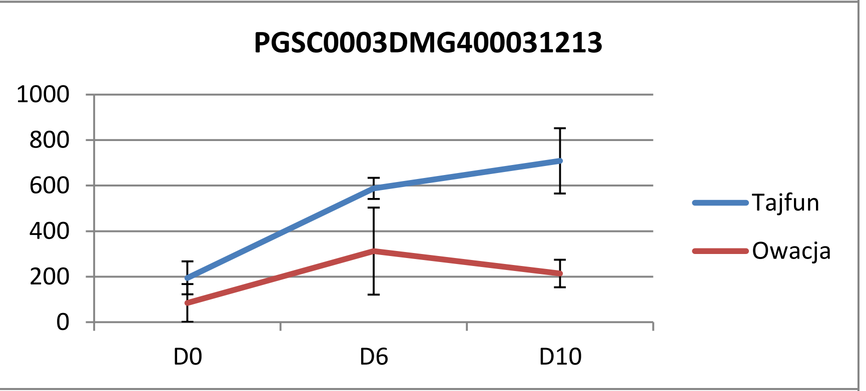

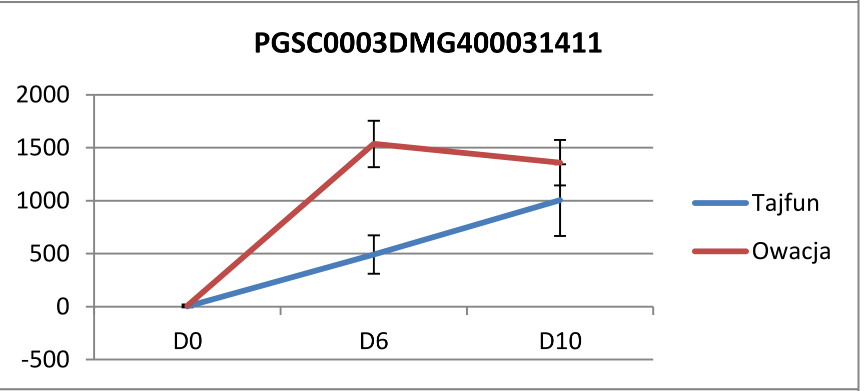

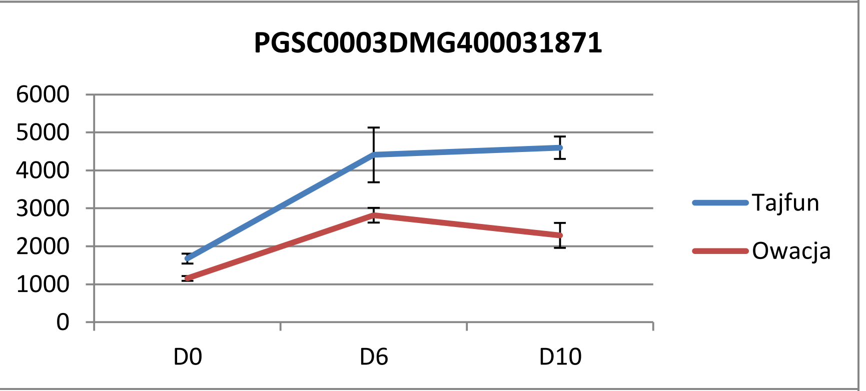

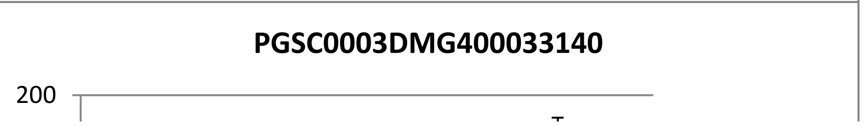

**PGSC0003DMG400033567**	4610,67	13966	21960	5976,33	27265	33247	477,22	2929	1880,5	1439,5	2484,3	6674,91
**PGSC0003DMG400033579**	1340	2307,3	2496,67	1315,33	1232,7	1473,3	86,19	186,72	150,1	204	143,11	219,93
**PGSC0003DMG400033876**	530	6070,7	3018	203,33	4556,3	1496,7	446,87	877,61	789,22	114,93	256,89	321,76
**PGSC0003DMG400033931**	4	310	111,33	0,67	103,33	28	4	123,47	15,53	1,15	67,72	24
**PGSC0003DMG400035200**	32,67	3902	4025,33	21,33	5581,3	1737,3	11,02	622,04	1536,8	7,02	1240,6	175,38

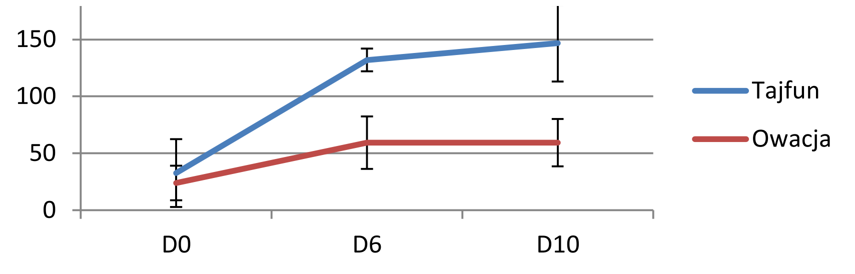

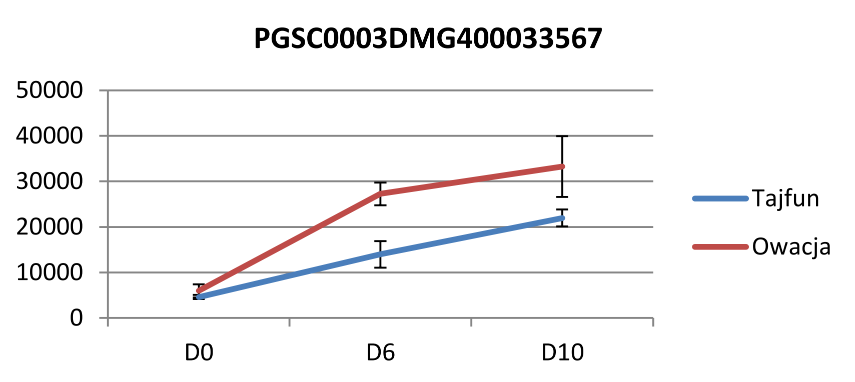

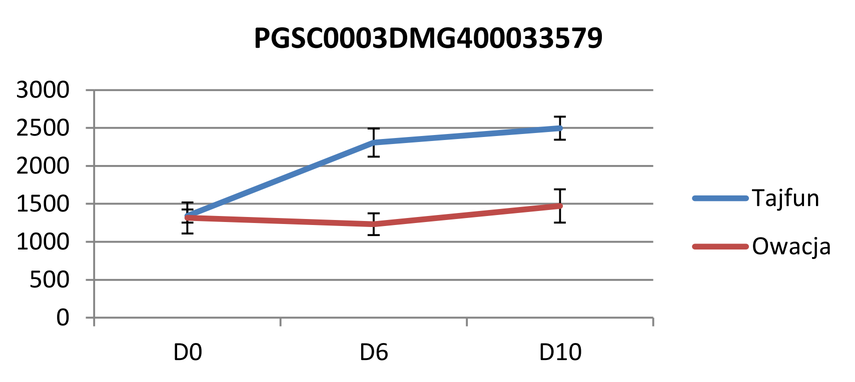

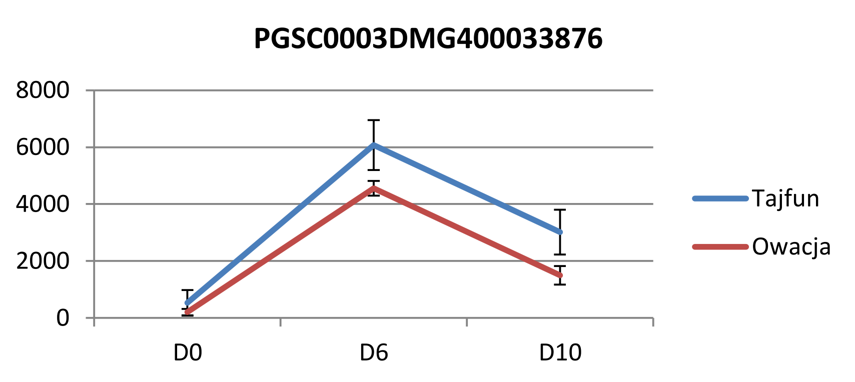

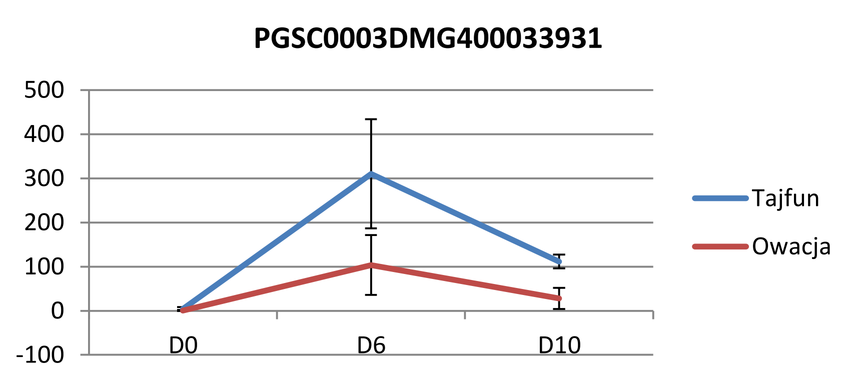

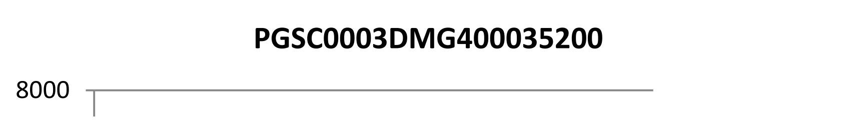

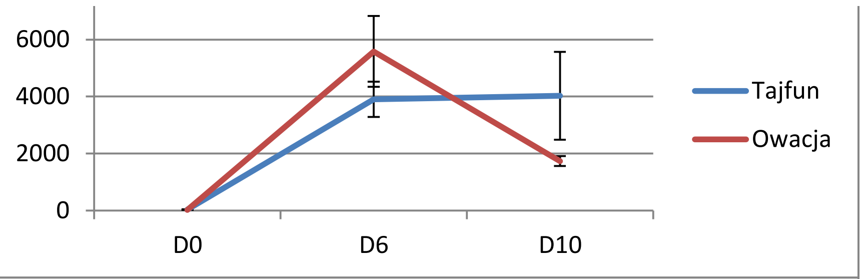

**PGSC0003DMG400035337**	1280	2834,7	2912	1202,67	1828,7	1637,3	199,19	85,8	385,56	182,54	615,53	75,75
**PGSC0003DMG400036082**	644	1550	1788	722	1095,3	886,67	51,26	116,26	381,58	82,44	119,84	213,28
**PGSC0003DMG400036477**	156,67	508,67	616,67	132	309,33	317,33	75,96	32,33	58,53	34,7	46,23	48,76
**PGSC0003DMG400039484**	56	1330,7	15989,3	29,33	377,33	1518	9,17	24,85	5131,9	4,16	388,85	201,37
**PGSC0003DMG400040535**	515,33	1854	1483,33	506	1117,3	690,67	121,99	281,37	383,05	161,7	69,06	72,15

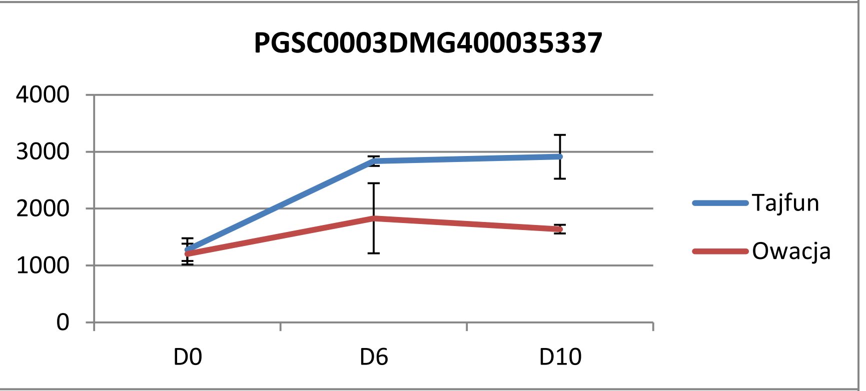

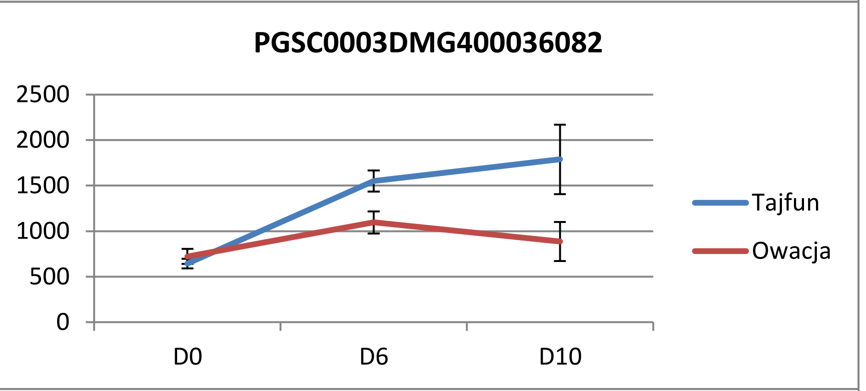

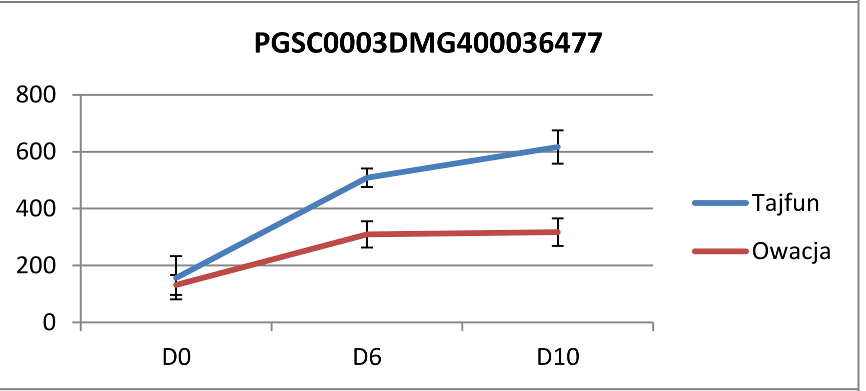

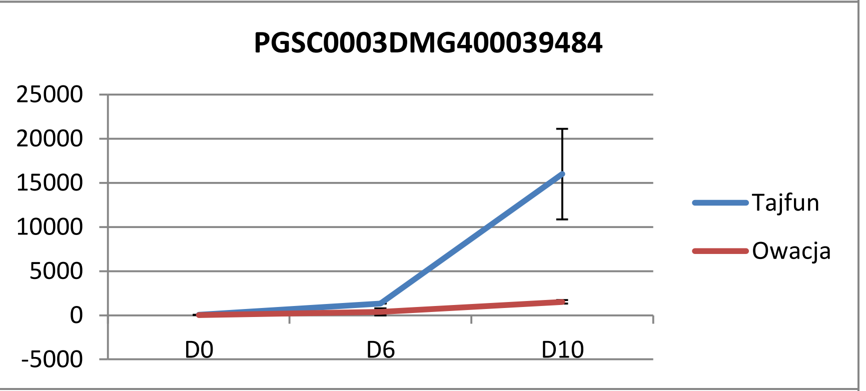

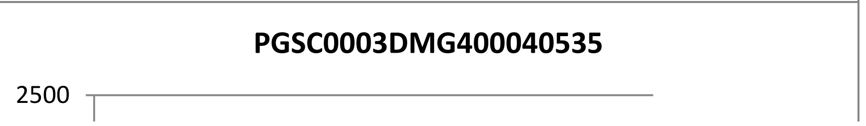

**PGSC0003DMG400042481**	15,33	228,67	178	7,33	124	58	12,86	54,93	84,45	6,43	41,33	34,18
**PGSC0003DMG400046798**	5,33	132,67	793,33	0	1,33	0	6,11	82,59	360,95	0	2,31	0
**PGSC0003DMG401001383**	144	531,33	677,33	86	331,33	265,33	63,91	51,16	211,27	41,62	17,01	66,25
**PGSC0003DMG401001731**	477,33	4502,7	20846	600	8024	27720	153,08	589,76	3288,2	202,59	3326,7	2474,55
**PGSC0003DMG401002721**	254,67	1462,7	2268,67	220	2018	1156	72,23	31,77	340,14	81,02	223,81	264,2

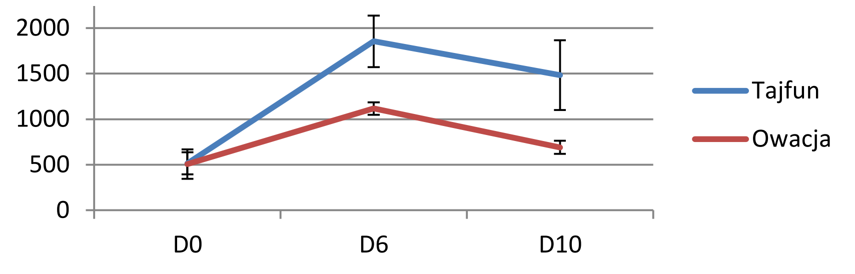

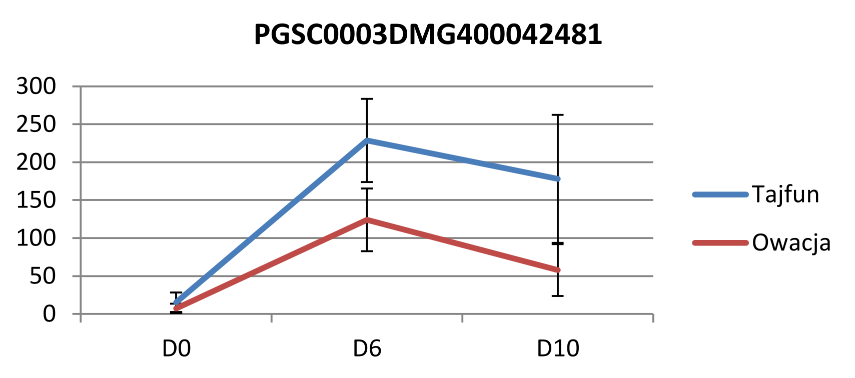

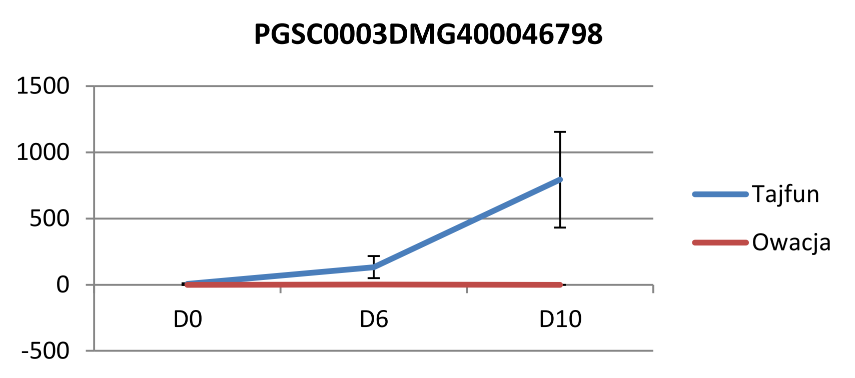

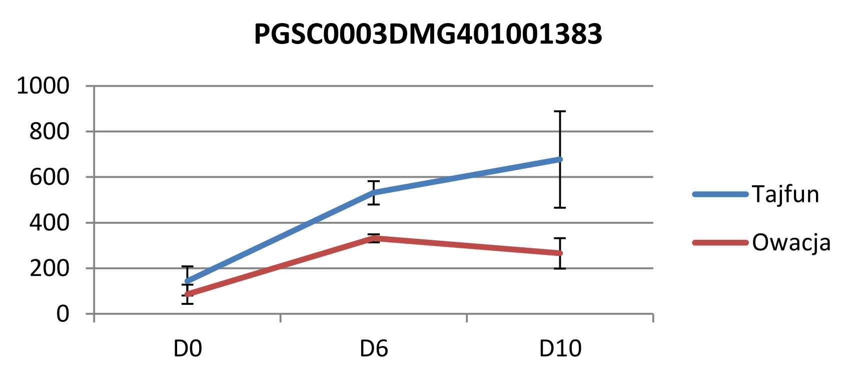

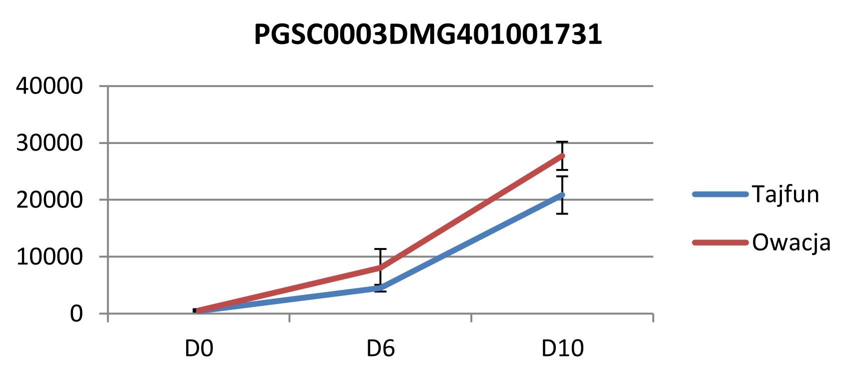

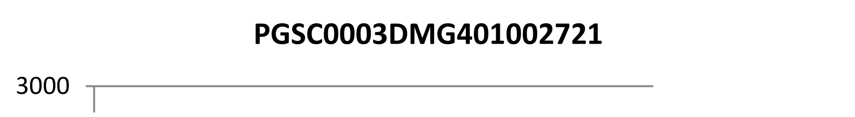

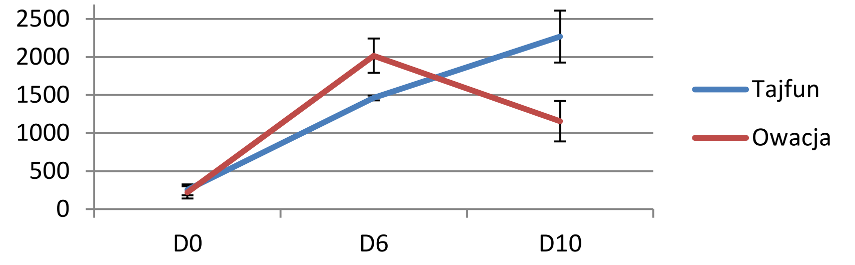

**PGSC0003DMG401005570**	1379,33	2396	2533,33	1568	3209,3	3660,7	308	199,03	594,65	252,95	366,31	568,02

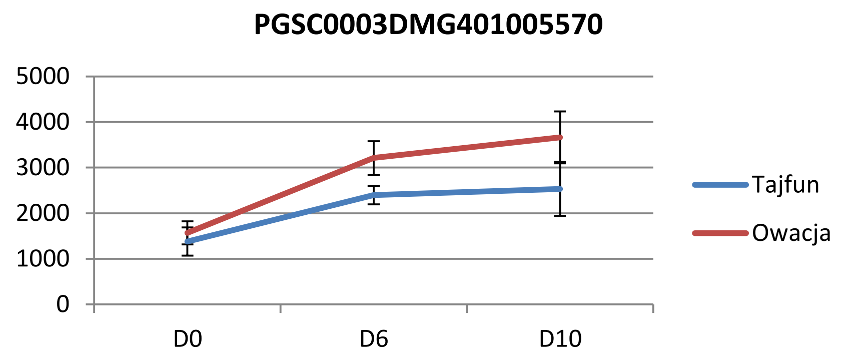

**PGSC0003DMG401007333**	898,67	2252,7	2804,67	575	1489,3	1132	121,36	453,54	1199,8	296,14	28,73	259,76

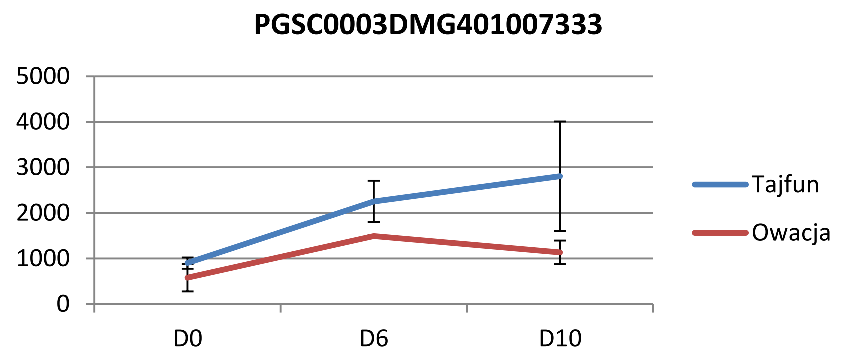

**PGSC0003DMG401009344**	3658,67	8758,7	12005,3	2639,33	12221	18241	313,69	1019,9	1274,4	897,95	1796,1	3108,91

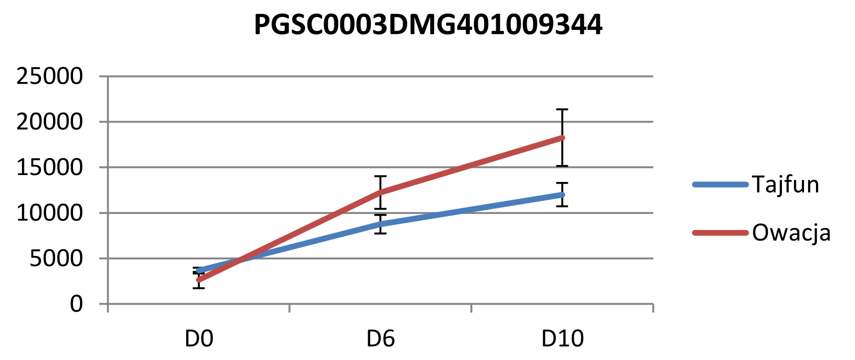

**PGSC0003DMG401016404**	1342,67	2825,3	3510	1336	4295,3	6460,7	251,61	176,64	326,24	387,26	230,97	502,19

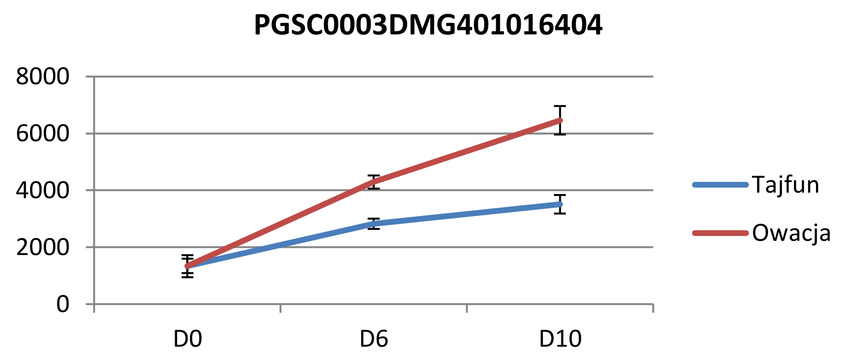

**PGSC0003DMG401017754**	657,33	1658	2133,33	543,33	1104	1022,7	64,01	231,14	279,4	73,33	55,46	112,33

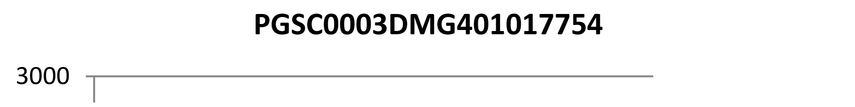

**PGSC0003DMG401018962**	40	730	322	22,67	326	126,67	21,07	102,43	26,23	3,06	48,66	51,43
**PGSC0003DMG401019352**	345	1048	3911,67	409,33	1524	5269	118,2	94,44	1051	142,13	199,87	462,52
**PGSC0003DMG401020509**	1318	2838,7	4026	1404	1912,7	1808	106,71	432,96	1498,7	202,67	238,26	423
**PGSC0003DMG401022202**	1370,67	7566,7	15180	1589,33	9966,7	24536	195	1140,9	4635	538,34	911,28	1530,4
**PGSC0003DMG401022285**	32	284,67	304,67	18	94,67	54,67	18	80,56	77,11	8,72	24,44	9,02

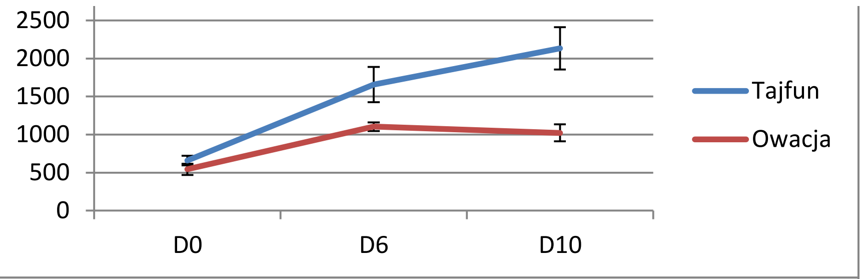

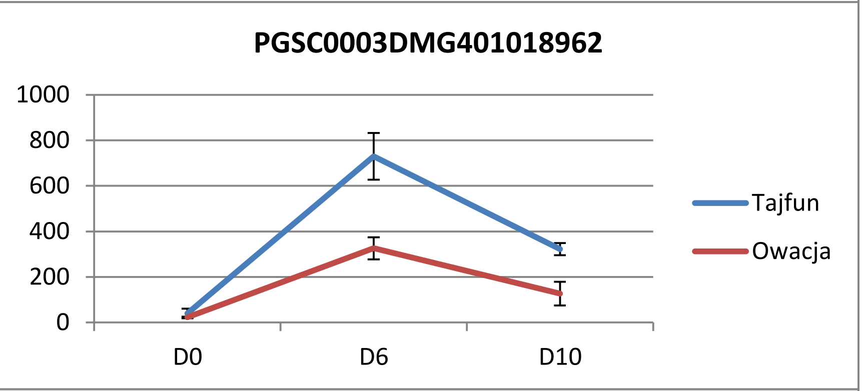

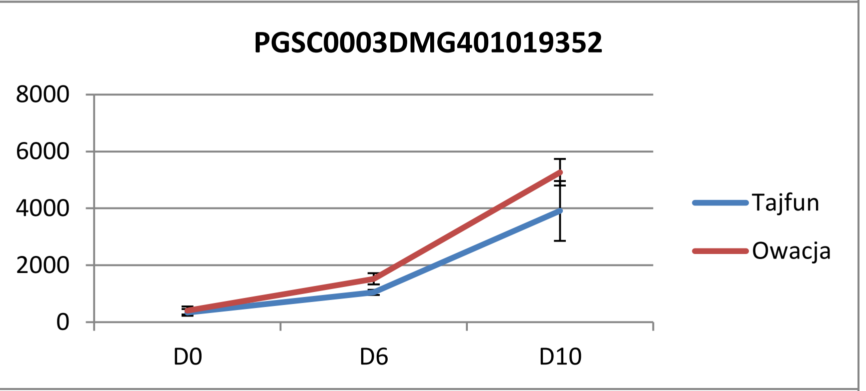

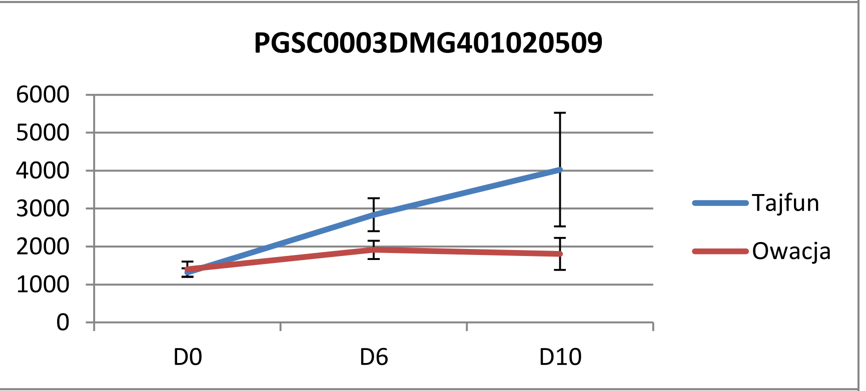

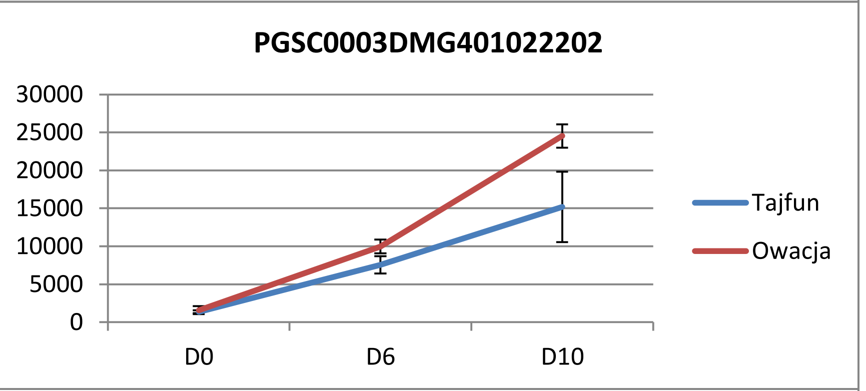

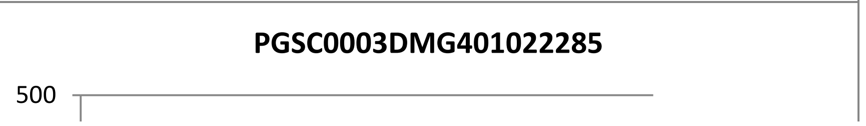

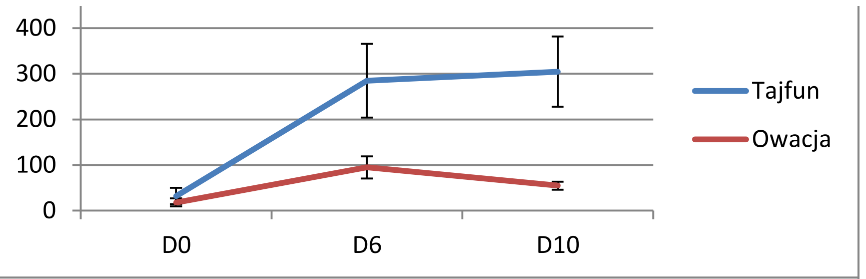

**PGSC0003DMG401023104**	75,33	237,33	754,33	54,33	140	251	10,07	18,77	108,91	0,58	33,41	68,51
**PGSC0003DMG401026519**	127	559	1275,33	111,33	1428,7	2885,3	27,62	265,16	280,4	50,96	451,33	141,74
**PGSC0003DMG401027528**	302,67	764,67	642	198,67	452,67	260,67	68,07	82,01	150,96	57,98	6,43	71,06
**PGSC0003DMG402001341**	2	52,67	290	0,67	100,67	480,67	2	15,14	181,89	1,15	36,02	20,03
**PGSC0003DMG402001383**	140,67	527,33	794,67	130,67	345,33	308,67	30,62	105,08	132,61	18,15	69,9	152,71

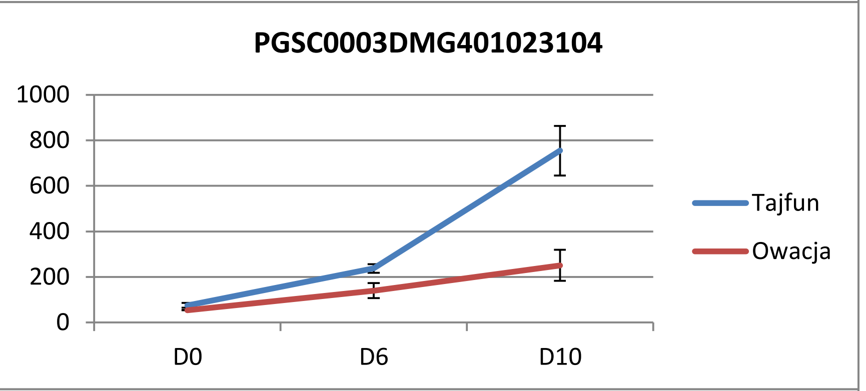

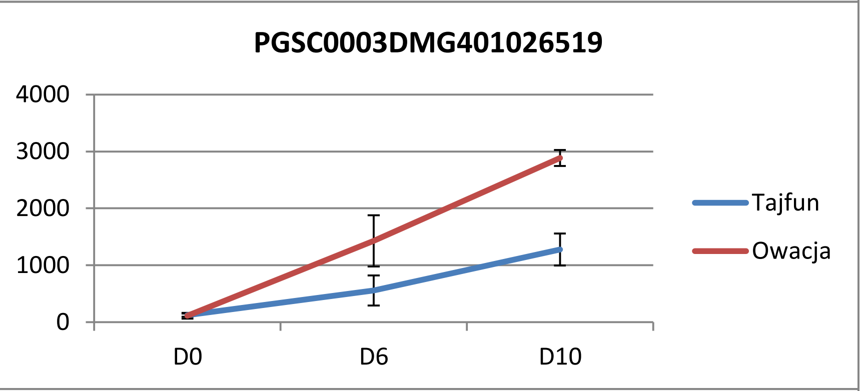

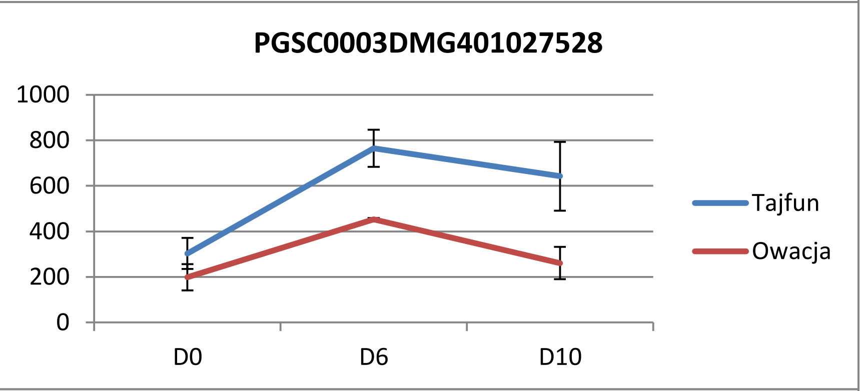

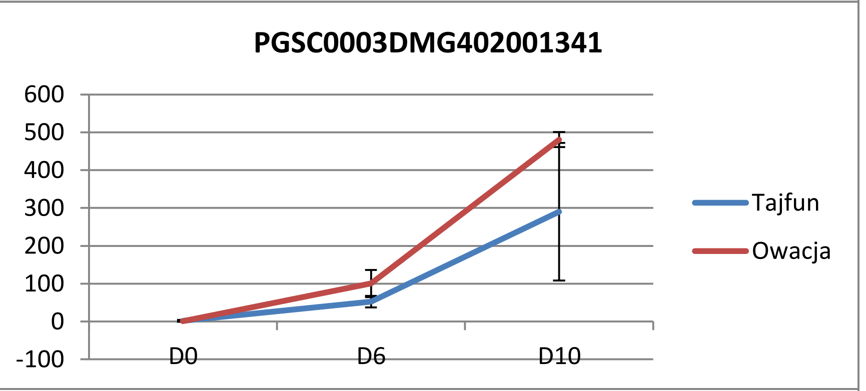

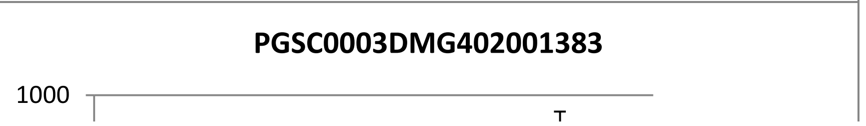

**PGSC0003DMG402001494**	310,67	2352	965,33	464,67	1462,7	496	42,44	225,31	329,1	184,58	340,74	107,63
**PGSC0003DMG402008946**	324,67	1486	8716,33	395,33	3861,3	19009	173,55	667,89	5267,6	220,55	1037,7	1803,21
**PGSC0003DMG402008965**	10,67	171,33	155,33	0	0	0	18,48	19,73	30,09	0	0	0
**PGSC0003DMG402009344**	812	2082	2950,67	649,33	2986	4200,7	33,29	175,86	715,89	161,05	407,18	795,6
**PGSC0003DMG402010614**	26,67	92,67	129	6	0	26,67	25,17	9,45	46,36	10,39	0	46,19

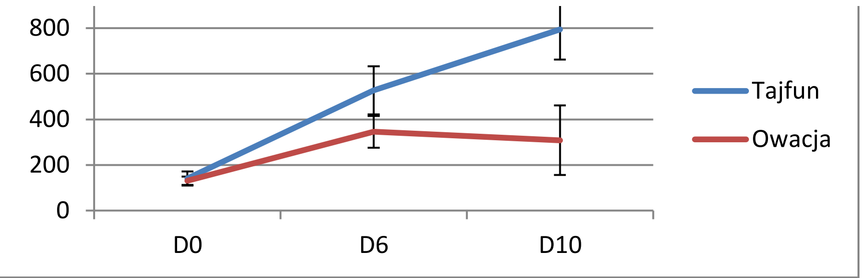

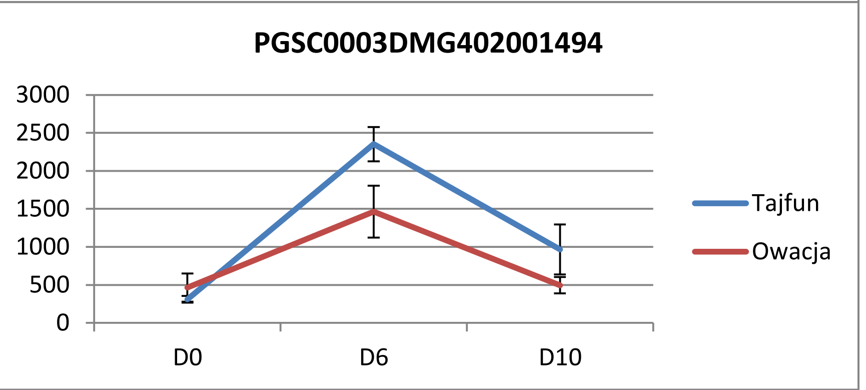

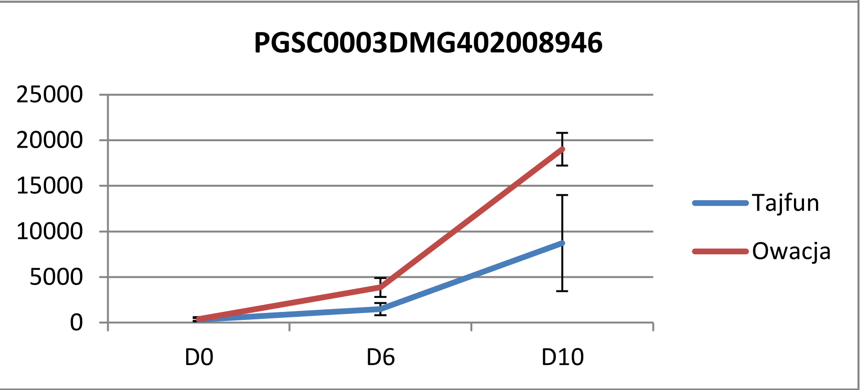

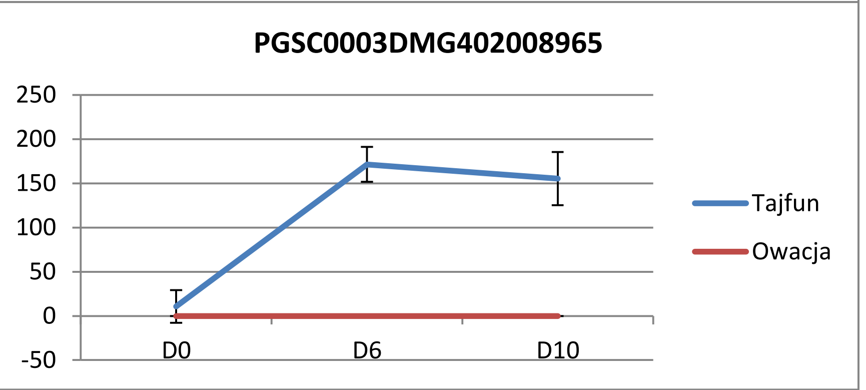

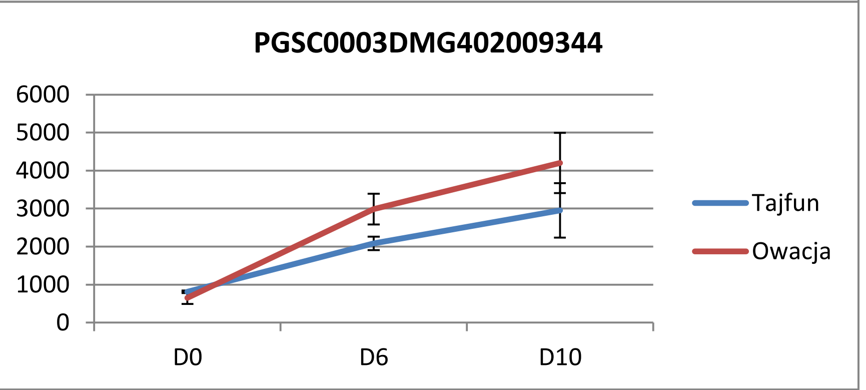

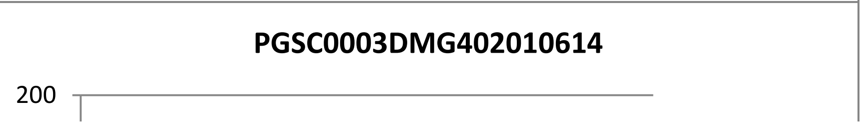

**PGSC0003DMG402015894**	543	2150	2341,33	744,67	3336	5894	63,41	696,55	1395,3	200,16	349,36	726,7
**PGSC0003DMG402016772**	288	798	765,33	196,67	474,67	378,67	72,33	135,63	79,03	22,12	173,37	52,55
**PGSC0003DMG402019209**	12069,7	27454	32368,7	7829,33	18578	15464	4437,3	5393,3	6293,2	1878,2	1004,8	953,41
**PGSC0003DMG402023446**	1037,33	4141,3	3826	786	5632,7	5606	88,82	940,05	1222,7	240	545,07	535,34
**PGSC0003DMG402027007**	2835,33	6021,3	6432	2110,67	4235,3	3504,7	141,88	708,9	795,05	525,09	918,44	549,26

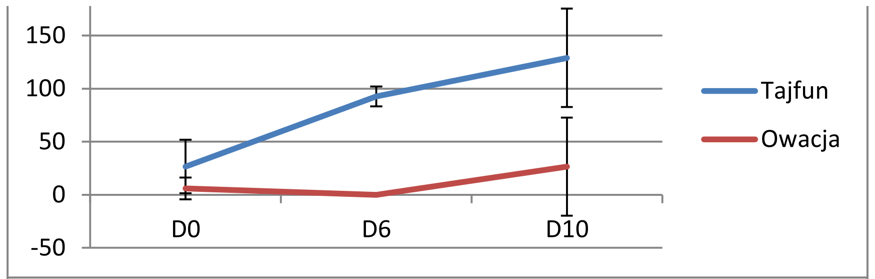

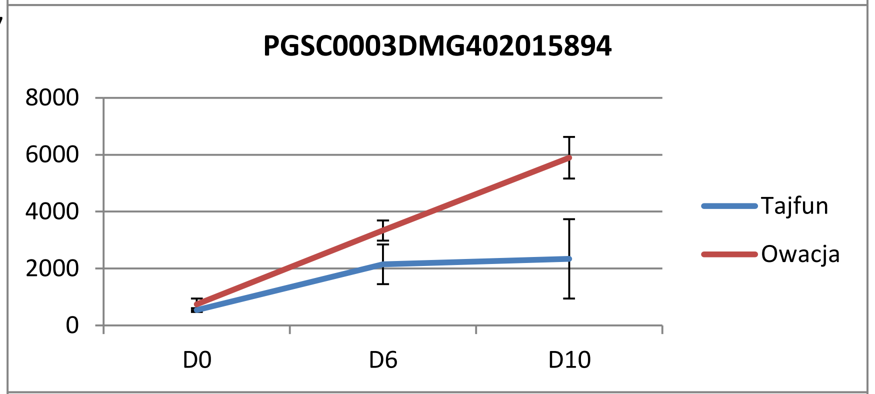

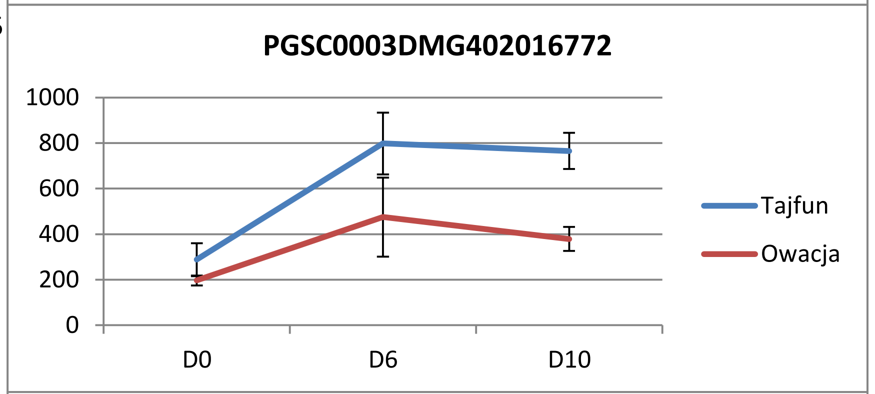

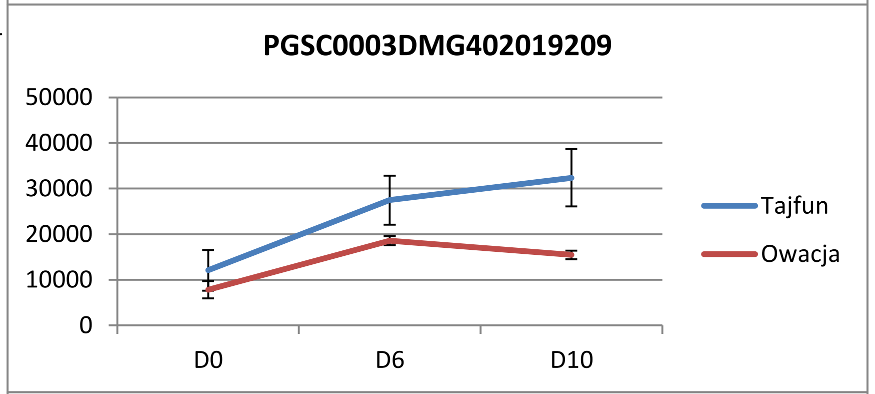

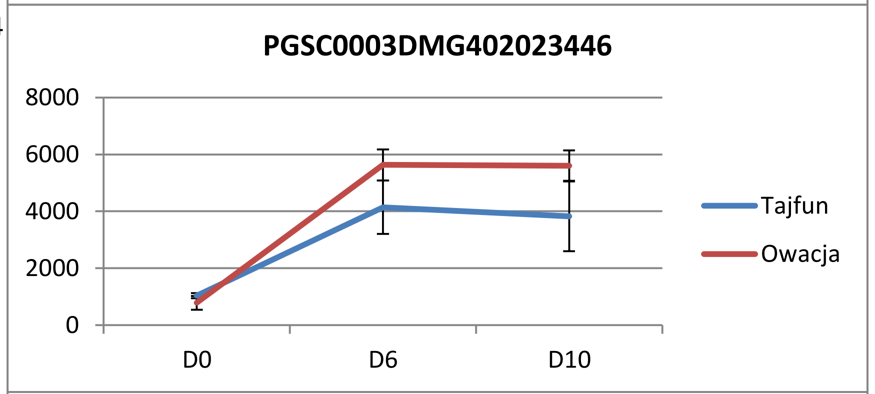

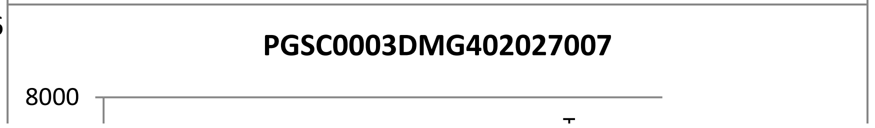

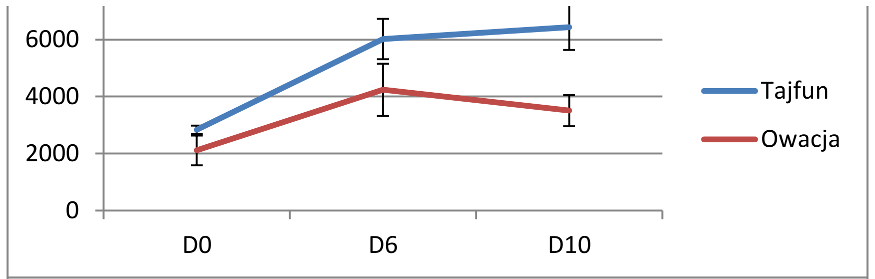

**PGSC0003DMG404000594**	22	741,67	330	25,33	405,67	26,67	38,11	140,01	70,55	5,03	29,14	12,86

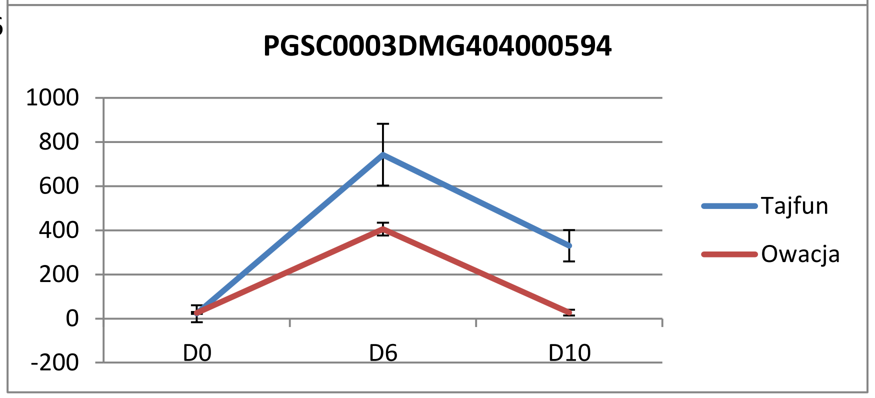

Table S1 The comparison of normalized number of reads for transcripts derived from genes searched out in the first round of selection (594 genes) during the time course of drought experiment. The gray color indicates the selected genes after the second round of selection.

Mean value of normalised reads per million for selected potato transcripts that were down-regulated in Tajfun comparing to Owacja. Data are shown for days: 0, 6, and 10 of drought experiment and were taken from three biological replicates. SD at D0 was always not statisticly significant ($P > 0{,}05$), while SD at D6 and D10 were always statisticly significant ($P < 0{,}05$, see also Fig 5). Potato gene accesion numbers were taken from Spud DB (www.potato.plantbiology.msu.edu). SD - standard deviations.

Gene accesion numbers	Tajfun reads mean value			Owacja reads mean value			Tajfun SD			Owacja SD		
	D0	D6	D10	D0	D6	D10	D0	D6	D10	D0	D6	D10
**PGSC0003DMG400000123**	1708	676,67	196,67	1796,7	1088	358,67	454,24	212	12,7	445,23	145,59	85,7
**PGSC0003DMG400000159**	7956	2288	825	6964	1648	222,67	608,17	260,18	412,81	2742	142,67	39,11
**PGSC0003DMG400000180**	3414	919,67	628,67	3034,7	1398,7	1026	668,44	80,34	54,45	922,19	132,81	75,18
**PGSC0003DMG400000185**	4746,7	2900	2795,33	4888,7	3986,7	4664	1252,6	326,23	376,86	1534,7	263,95	765,32
**PGSC0003DMG400000187**	1112	632	432	1488,7	1380,7	1347,3	175,86	100,18	66,09	340,24	409	98,11

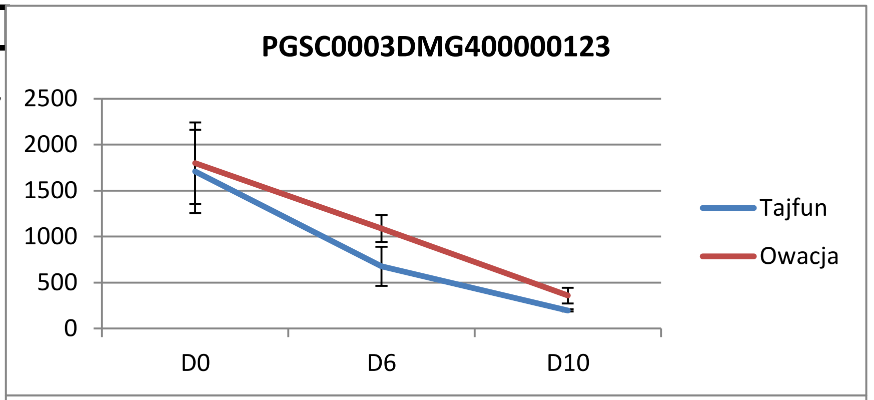

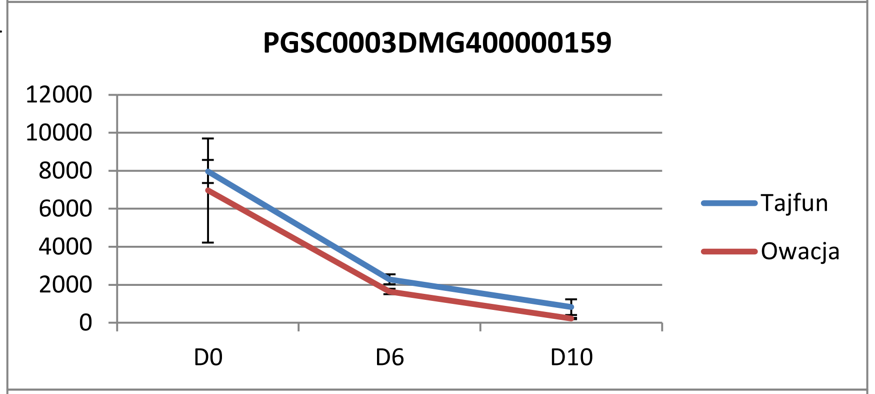

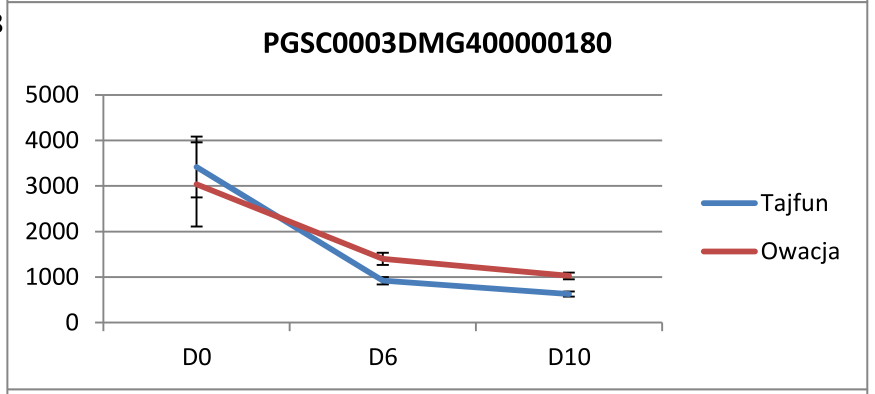

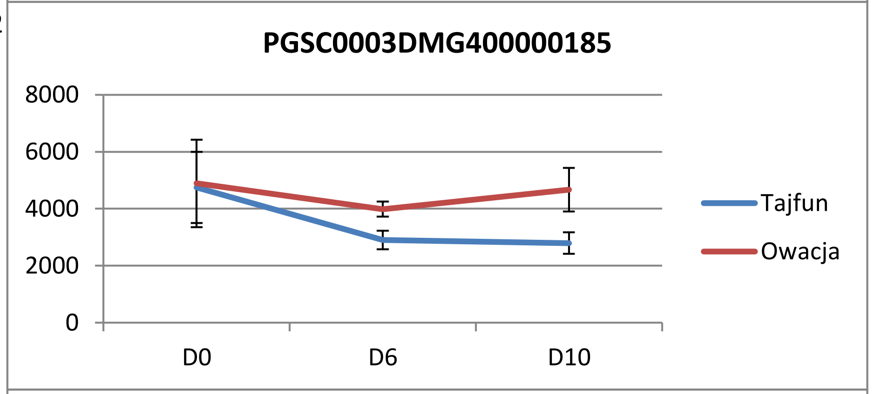

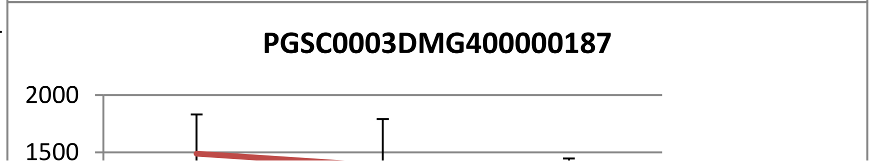

**PGSC0003DMG400000325**	3892	776	538,67	1900	278	112	1400,6	183,61	318,85	394,99	137,13	16,37
**PGSC0003DMG400000327**	13936	5042,7	2732	8923,3	3185,3	576,67	3795	1256,4	1168,08	2139,9	469,9	145,69
**PGSC0003DMG400000545**	589,33	165,33	119,33	359,33	384	826,67	378,64	36,9	55,22	95,63	129,29	195,53
**PGSC0003DMG400000640**	1956,7	1302,7	540	2285,3	1902	883,33	208,82	58,01	29,05	721,23	126,76	155,72
**PGSC0003DMG400000746**	166,67	14,67	75,33	206	110	262	39,46	13,61	1,15	64,37	26,15	12,49

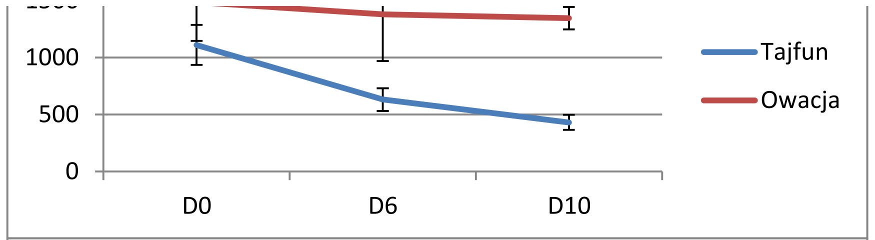

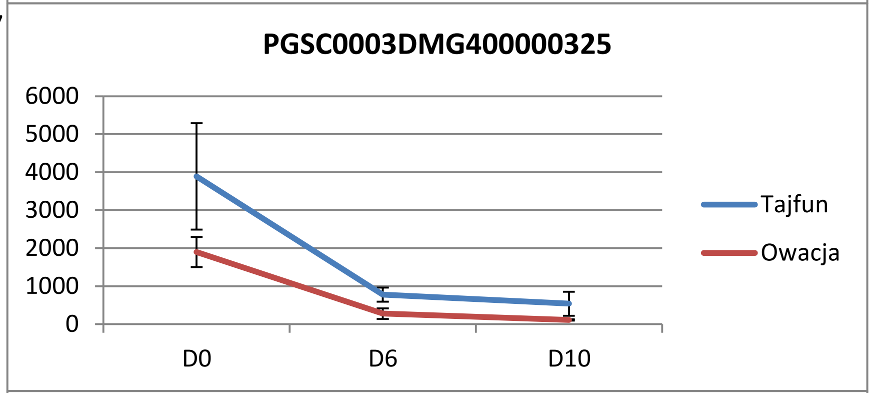

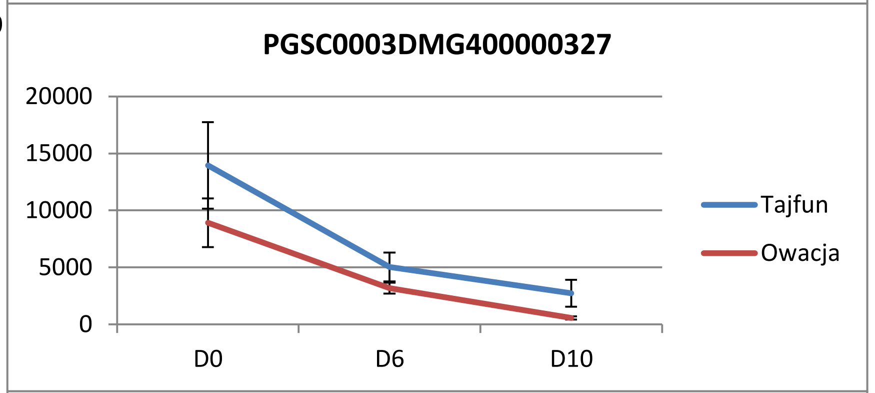

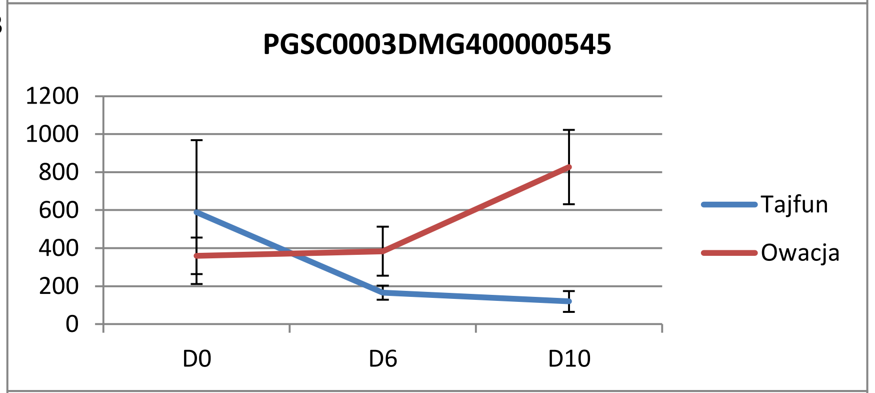

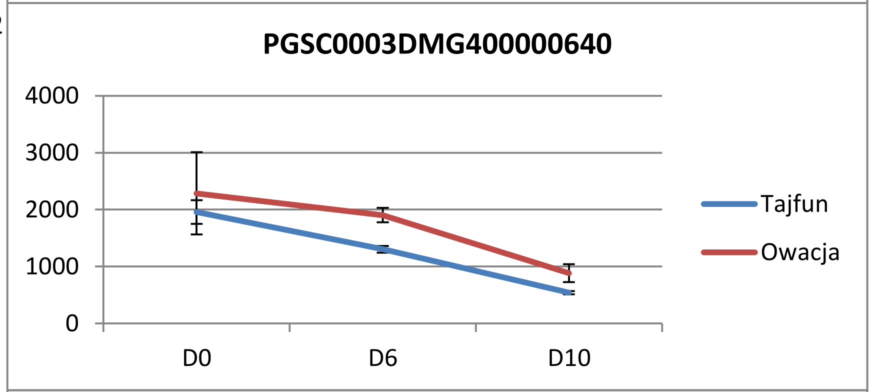

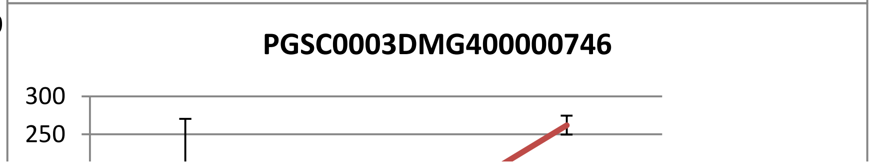

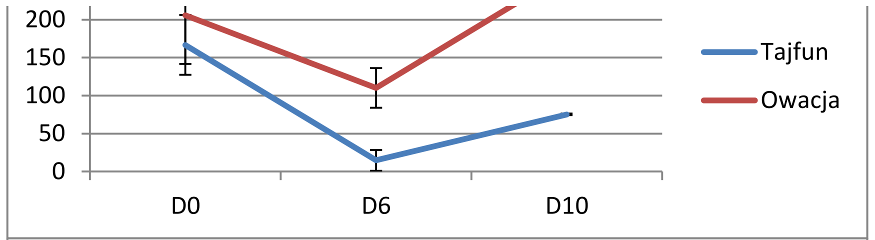

**PGSC0003DMG400000988**	677,33	292	151,33	412	109,33	43,33	134,84	89,6	50,96	72,33	35,8	23,09
**PGSC0003DMG400001368**	8867,3	5782	4345,33	9539,3	9451,3	12827	1859,2	1416,2	1579,16	3656,5	1897,76	521,92
**PGSC0003DMG400001570**	120355	72361	46061,3	104264	96101	69657	11888	13591	21455,6	48156	9750,84	4343
**PGSC0003DMG400001576**	722,67	344	306,67	1047,3	172	155,33	79,1	22,72	12,22	390,01	5,29	25,32
**PGSC0003DMG400001589**	408,67	260,67	39,33	336	90,67	0	75,16	37,22	34,08	81,46	21,2	0

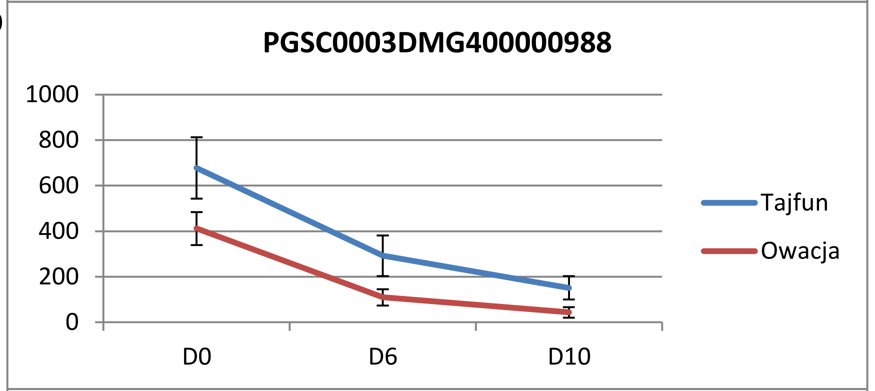

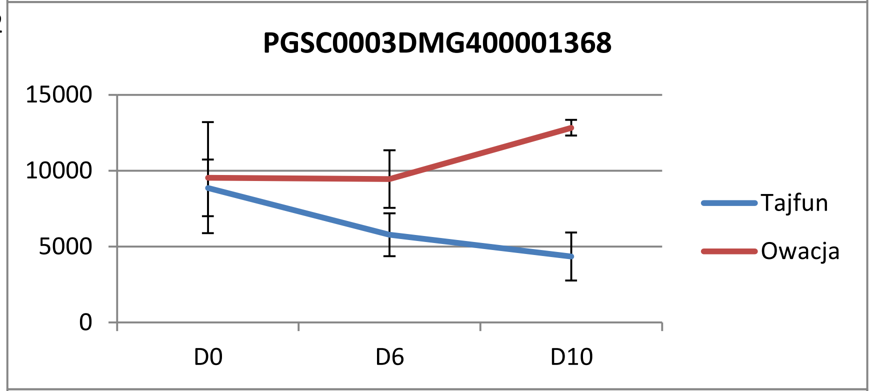

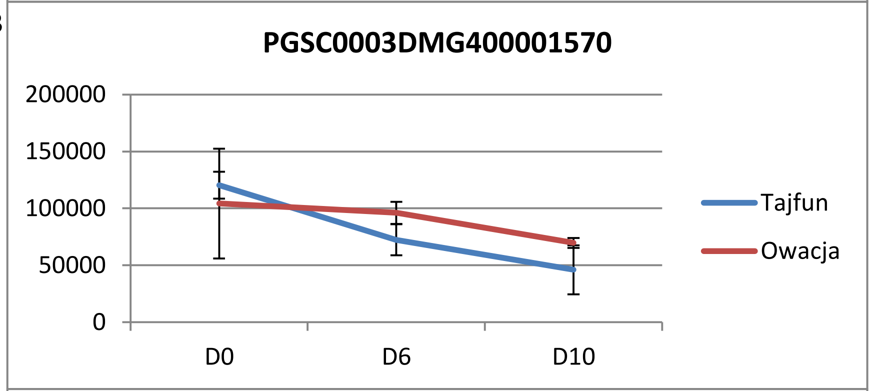

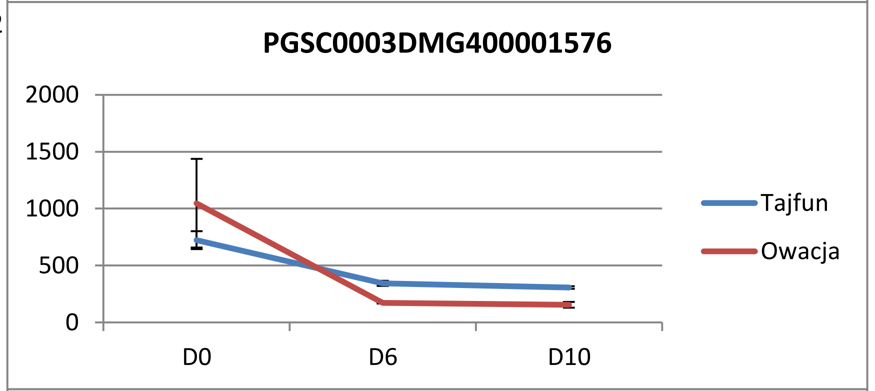

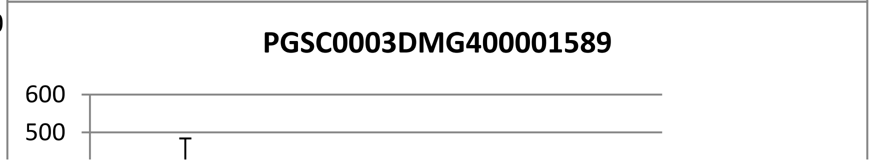

**PGSC0003DMG400001684**	782	226	193,33	586,67	112,67	340,67	41,62	51,07	43,47	110,44	28,94	95,51
**PGSC0003DMG400002233**	2858,7	1894	1450,67	2757,3	3051	2364	627,93	464,68	343,7	635,08	592,91	279,65
**PGSC0003DMG400002420**	401,33	40	28	216,67	4,67	2	151,23	22,54	22,54	93,84	4,16	3,46
**PGSC0003DMG400002787**	283,33	68,67	0	238,67	20	11,33	112,38	30,09	0	65,43	9,17	7,57
**PGSC0003DMG400002789**	1636,7	800,67	534,67	1191,3	1647	738,67	835,38	158,57	46,06	606,57	506,44	161,77

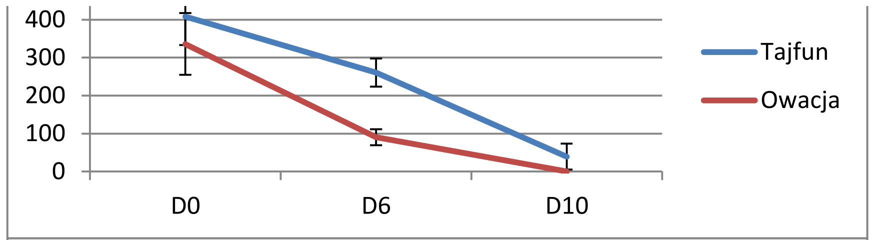

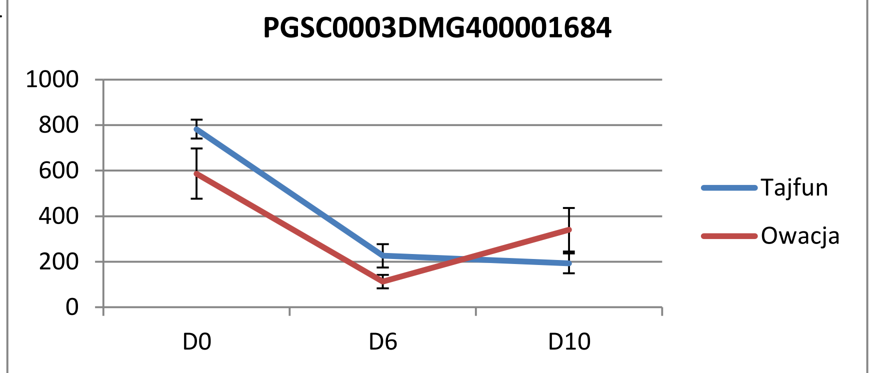

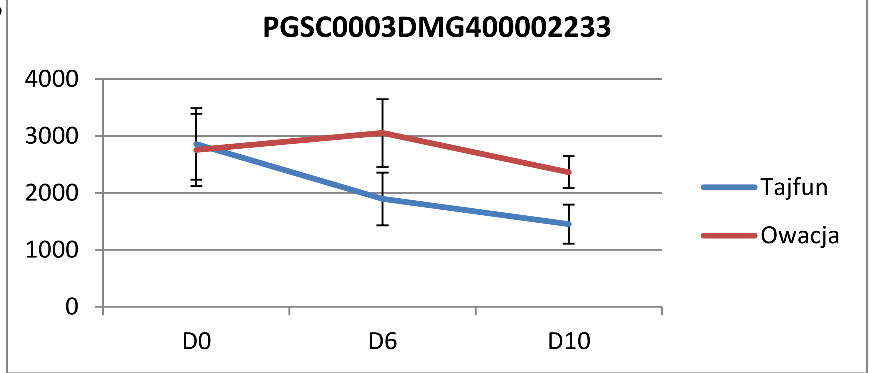

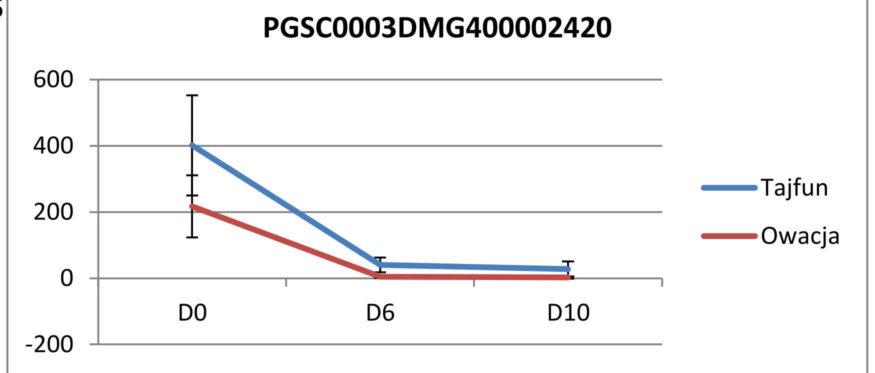

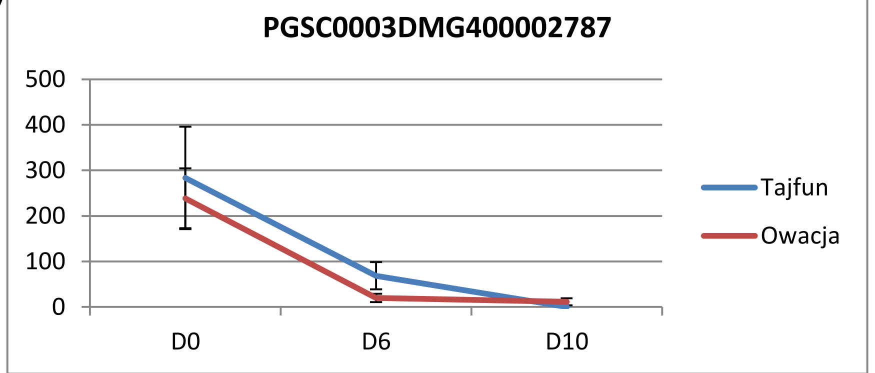

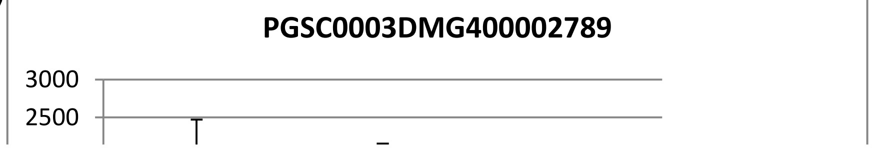

**PGSC0003DMG400002883**	2060	367,33	144,67	1444,7	149,33	0	216,04	94,45	73,11	472,57	128,02	0
**PGSC0003DMG400003036**	440	254	260	322,67	135,33	116	34,7	25,53	106,71	34,43	9,87	15,87
**PGSC0003DMG400003155**	2596	1995,3	1227,33	2892	2748	2026	673,57	313,42	173,6	589,12	102,84	357,66
**PGSC0003DMG400003774**	1556,7	587,33	554,33	1622,7	322,67	153,33	429,72	205,23	206,34	394,68	11,37	77,31
**PGSC0003DMG400003788**	3730,7	48	22	3820,7	186	122	1484,3	6,93	8,72	1337,9	54,37	19,7

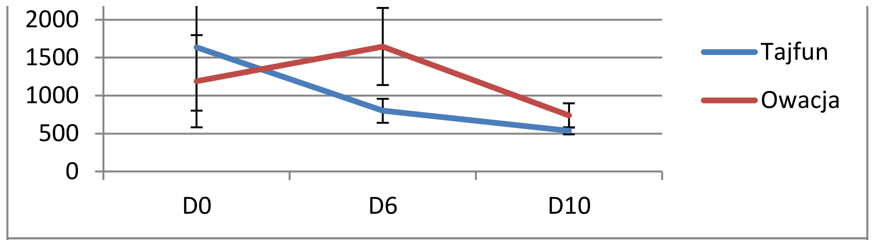

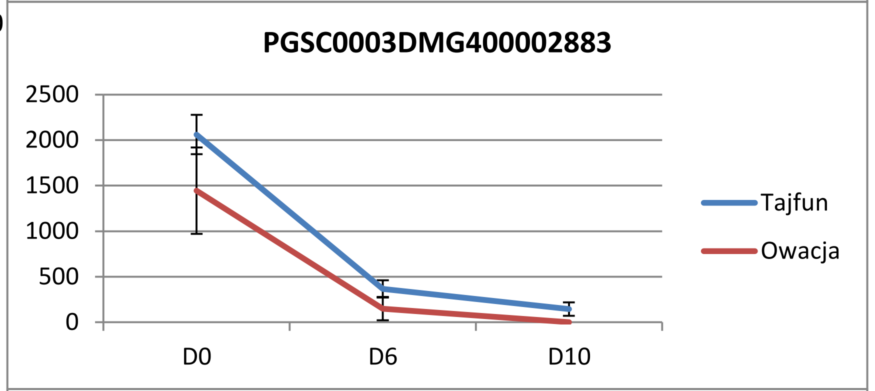

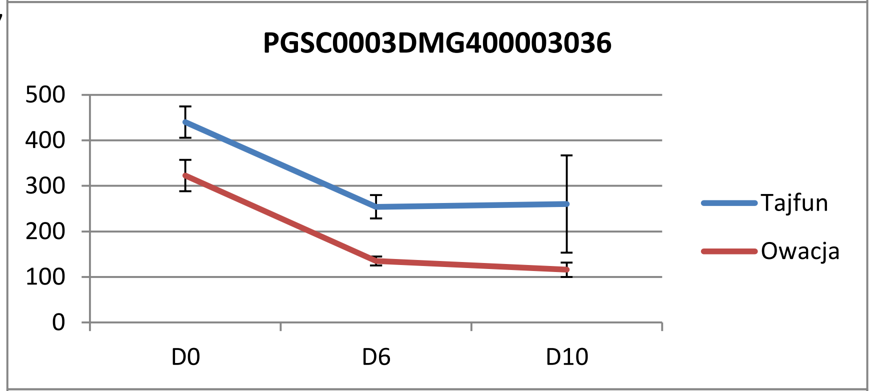

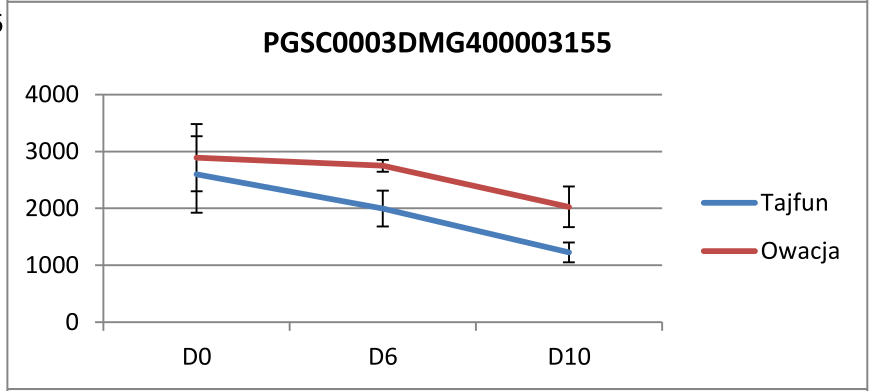

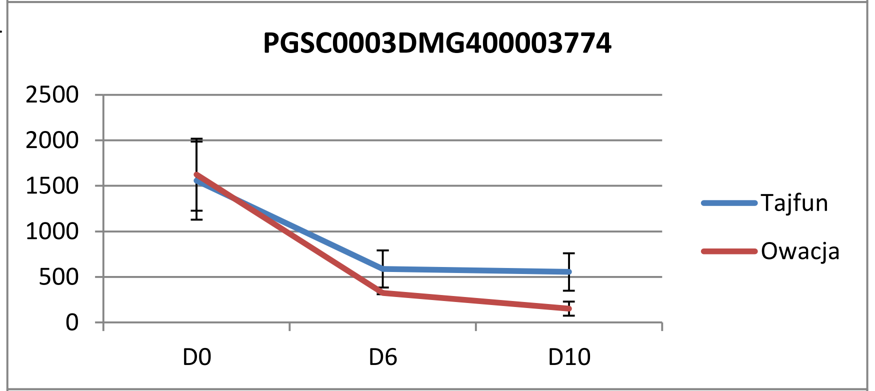

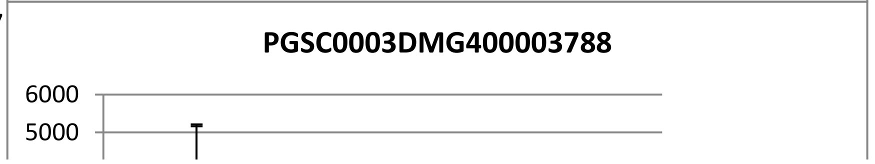

**PGSC0003DMG400003887**	4434,7	2500,7	2704	4206,7	4562	8744	682,56	564,9	1389,91	859,24	1739,55	1267,4
**PGSC0003DMG400003901**	236,67	135,33	70,67	132	55,33	10	105,08	33,25	16,77	68,79	17,93	5,29
**PGSC0003DMG400003913**	1960	359,33	148	2507,3	720,67	237,33	256,02	43,75	80,3	289,17	143,87	45,8
**PGSC0003DMG400004251**	529,67	44	0	557,67	196,67	112,67	224,04	38,57	0	129	30,55	3,06
**PGSC0003DMG400004601**	1237,3	200,33	185	840,67	324,33	377,33	187,79	28,57	131,27	162,86	65,5	70,04

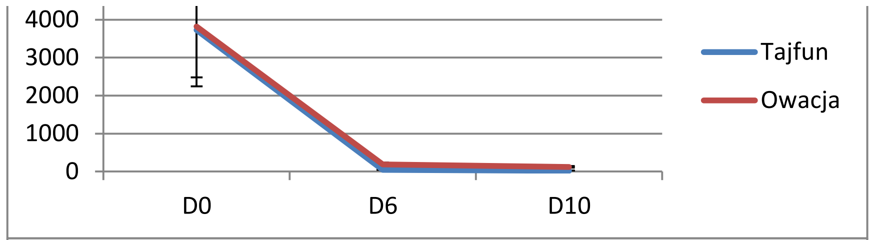

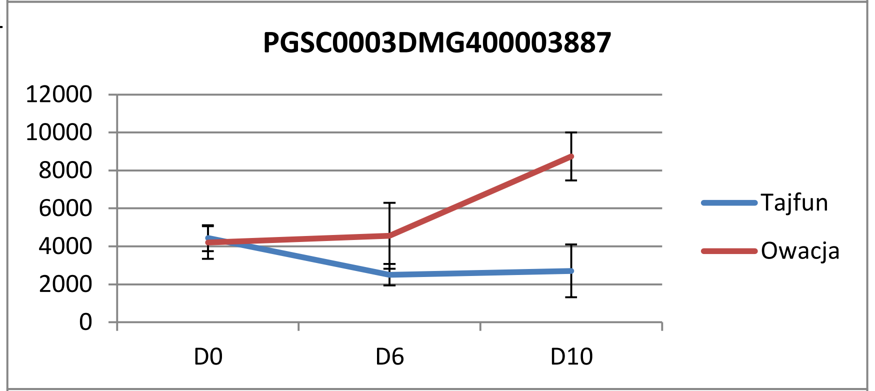

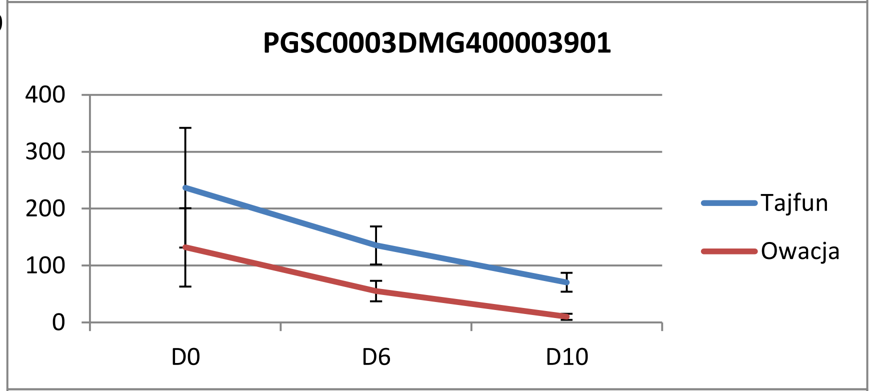

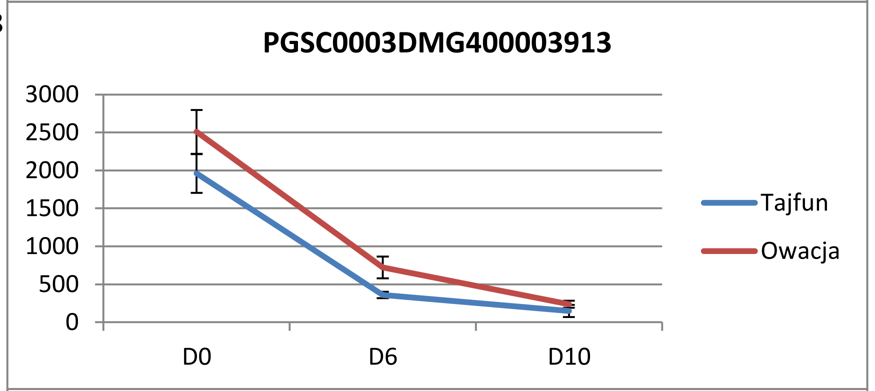

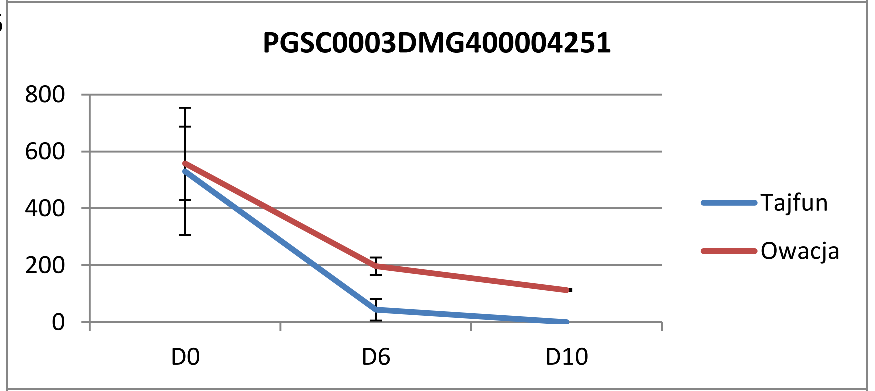

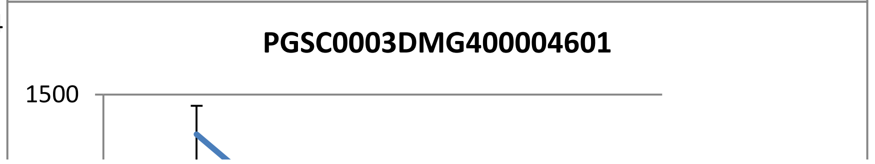

**PGSC0003DMG400004639**	23225	3264	2724	13637	1503,3	720	6220,2	936,78	1466,43	5047,6	670,81	240,9
**PGSC0003DMG400004708**	559,33	200,67	47,33	344,67	330	150,67	71,45	40,61	41,3	88,75	67,91	88,82
**PGSC0003DMG400004842**	240	22,67	0	164	0	86	18,33	23,01	0	51,42	0	21,63
**PGSC0003DMG400005031**	424,67	168	139,33	516,67	408	374	122,87	23,07	12,7	173,51	45,74	57,17
**PGSC0003DMG400005035**	808	507,33	305,33	754,67	773,33	1090,3	170,04	3,06	68,16	225,31	57,35	145,79

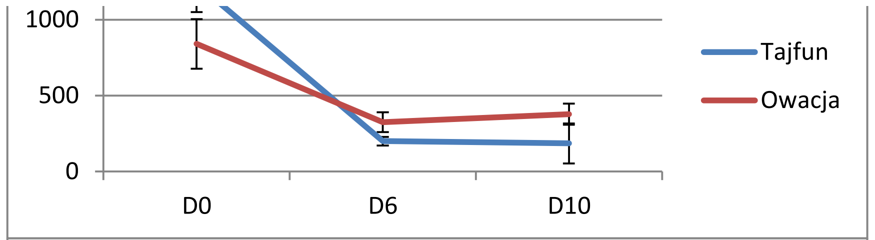

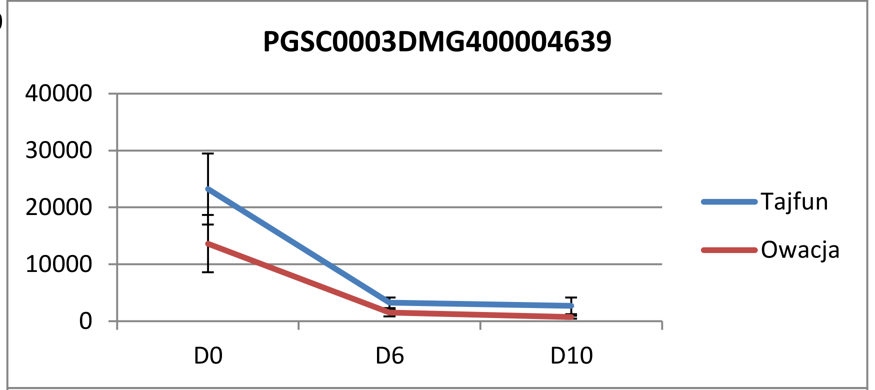

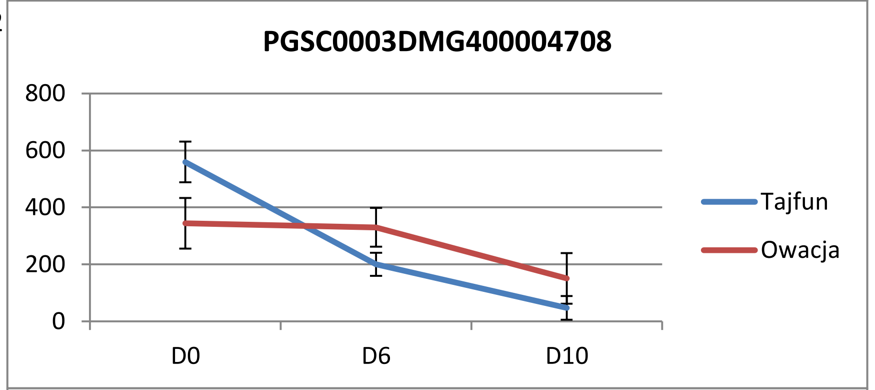

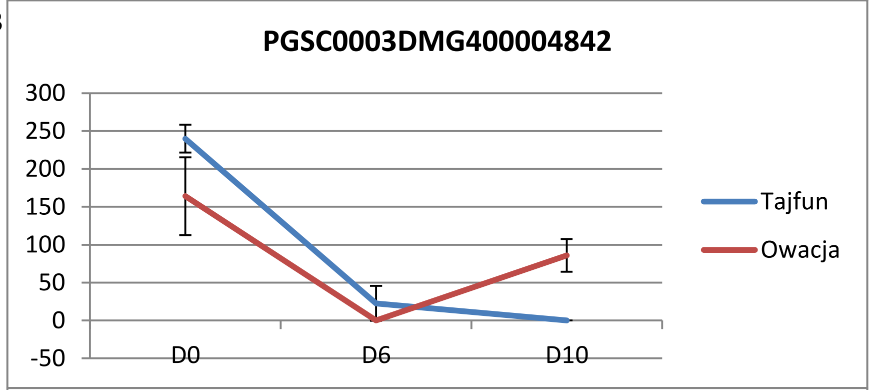

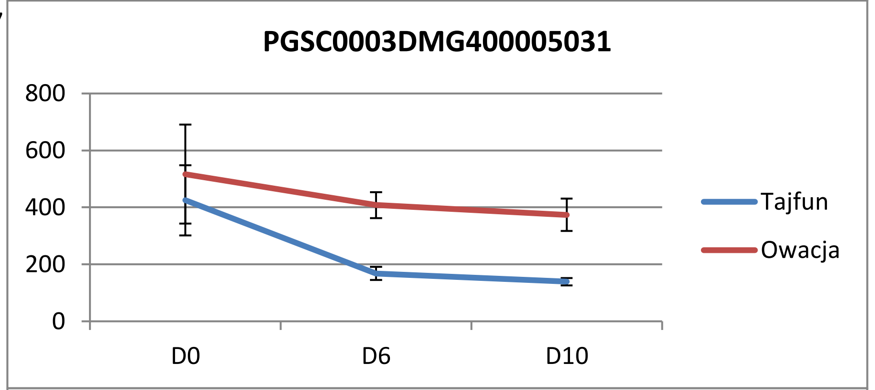

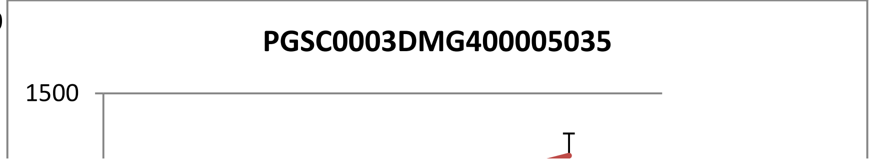

**PGSC0003DMG400005214**	2212	1449,3	198	2108,7	832,67	432	496,89	339,04	85,35	555,73	254,06	110,92
**PGSC0003DMG400005361**	6133,3	4442,7	2120,67	5646	3188	3971,3	297,2	213,85	120,21	1337,5	875,17	914,43
**PGSC0003DMG400005409**	1331,3	230,67	108,67	812,67	70	40	505,06	39,21	43	477,41	36,06	14,42
**PGSC0003DMG400005633**	13475	2504,7	601,33	12495	1345,3	77,33	3440,8	304,54	471,99	1928,4	206,03	63,57
**PGSC0003DMG400005661**	875,33	576	598,67	754	375,33	1188	148,44	90,8	255,91	84,07	144,46	209,98

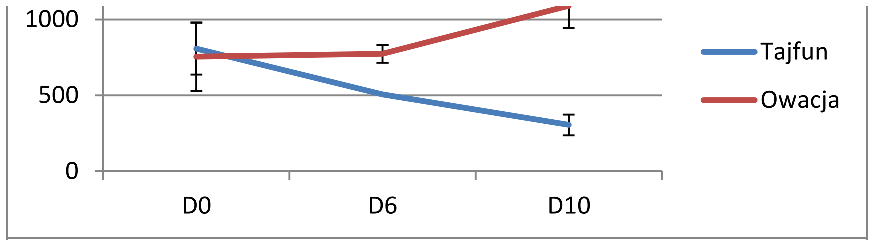

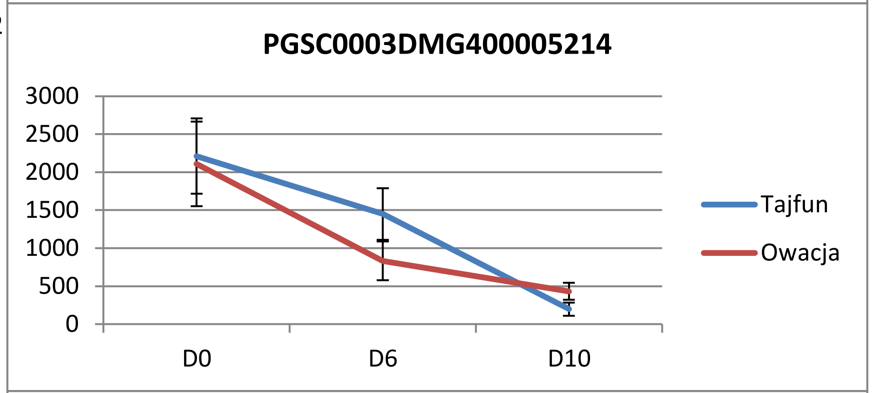

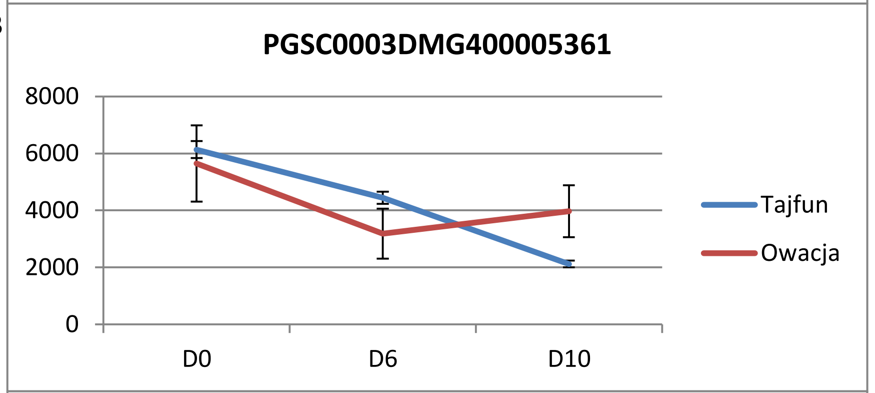

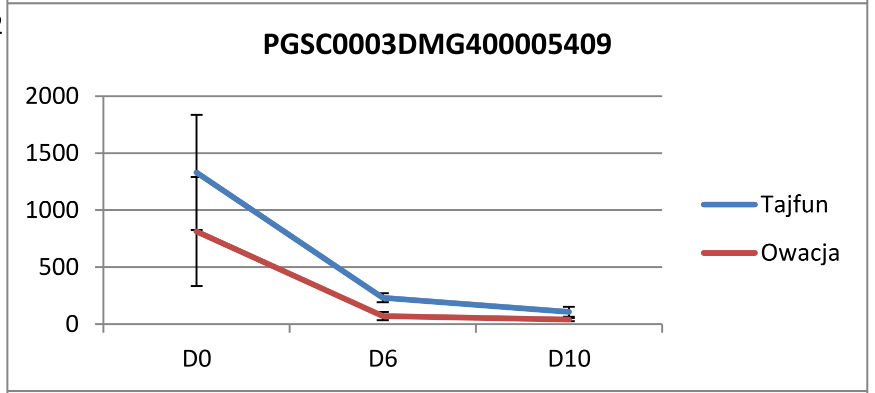

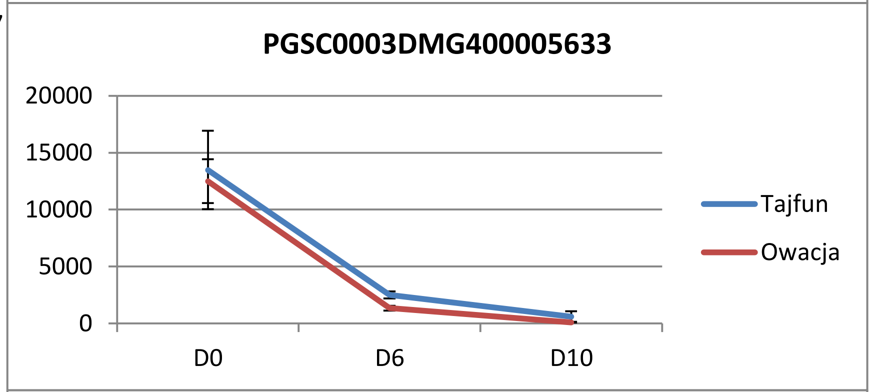

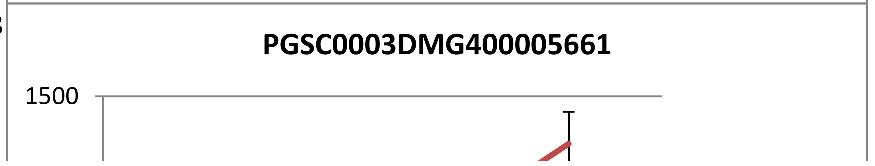

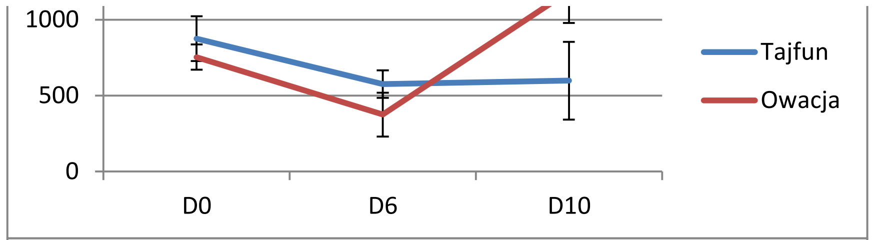

**PGSC0003DMG400005936**	1798	757,33	295,33	1093,3	458	154	600,36	56,01	50,65	430,55	88,45	47,29
**PGSC0003DMG400006508**	3449,3	1869,3	722,67	2346,7	1085,3	367,33	401,52	21,39	103,47	158,07	134,48	49,57
**PGSC0003DMG400006770**	2380,7	1597,7	1660	2681	2409	2439,3	441,41	321,57	294,2	194,87	390,83	195,36
**PGSC0003DMG400006828**	13247	11855	9302,67	9172	8346,3	4497,3	3569,4	1341,4	1914,51	2734,3	417,49	614,39
**PGSC0003DMG400006913**	1040	346	367,33	1402	2497,3	2618	300,75	60,53	78,01	271,91	115,11	581,57

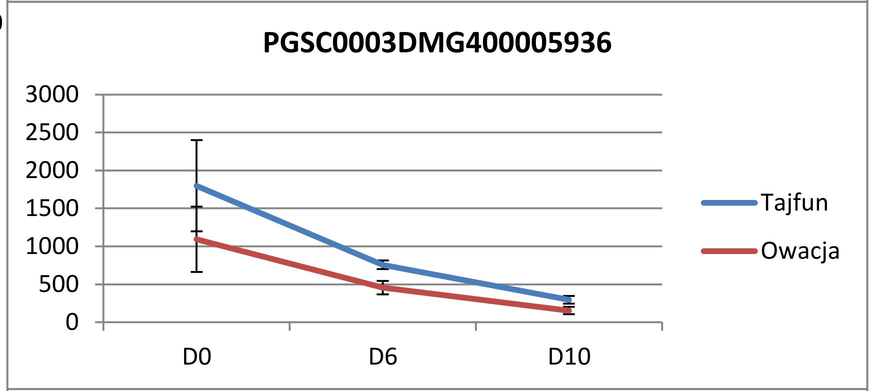

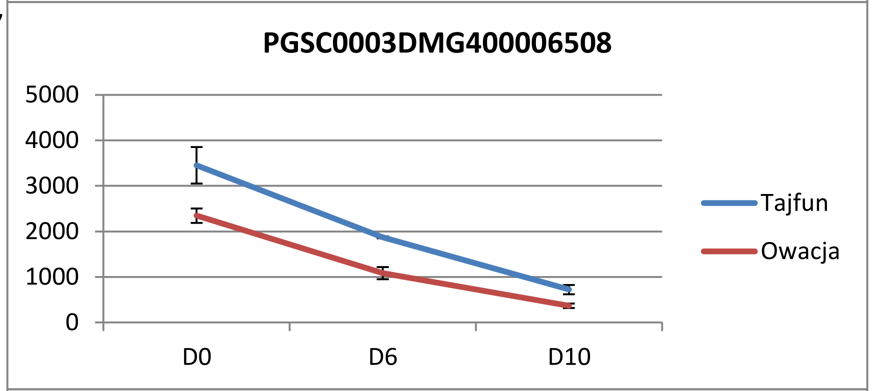

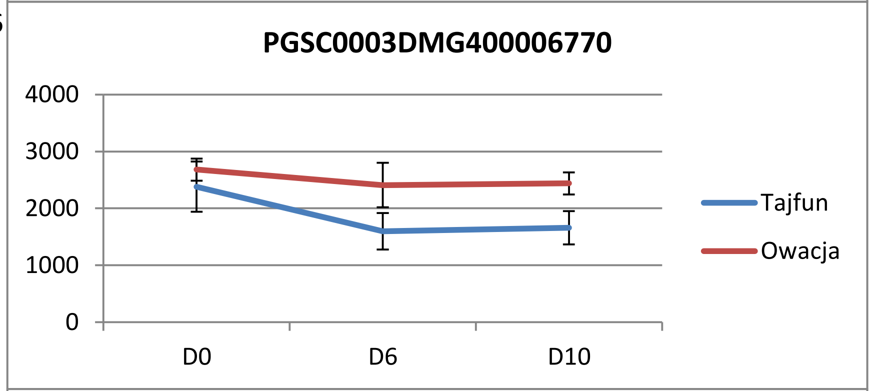

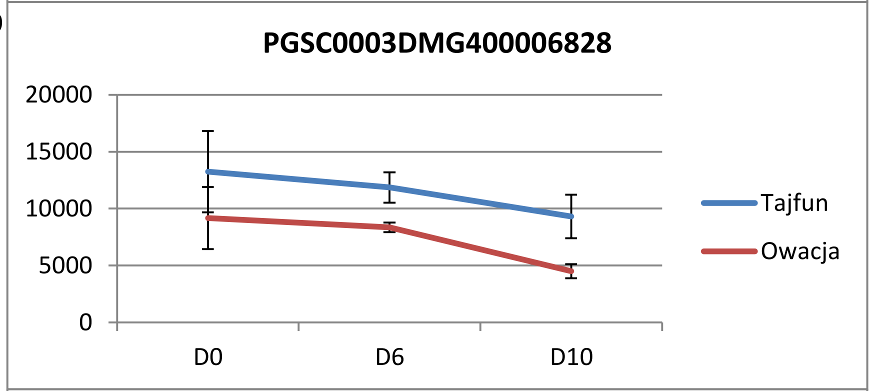

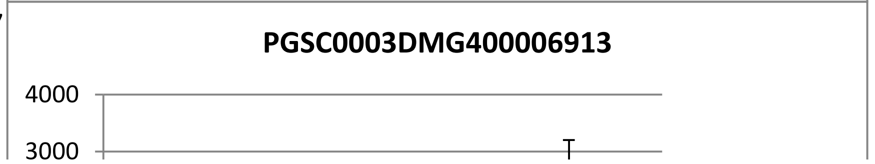

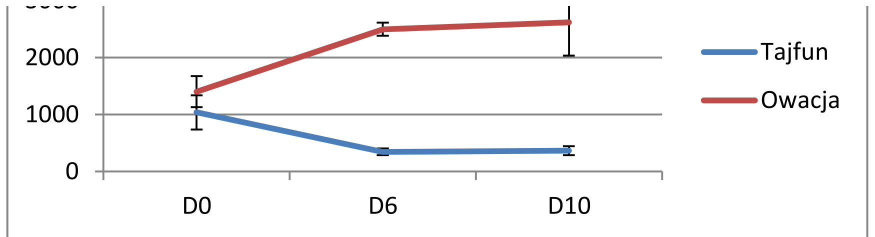

**PGSC0003DMG400007054**	647	389,67	318	825,33	617	605	197,76	45,63	48,5	27,3	80,29	61,83
**PGSC0003DMG400007058**	12421	4086,3	4263,33	8113	5406,7	6992,3	2282,6	888,32	1191,68	2044,3	351,19	1265,1
**PGSC0003DMG400007374**	4502,7	358,67	1636,67	4144	768	15446	1076,5	106,81	2089,71	1035,6	86,81	3415
**PGSC0003DMG400007427**	291,33	80	97,33	339,33	315,33	366	31,77	38,16	35,8	118,02	27,01	83,74
**PGSC0003DMG400007518**	55916	31276	41574,7	70563	49231	115515	1705,4	4086	19781,6	16712	6348,26	20018

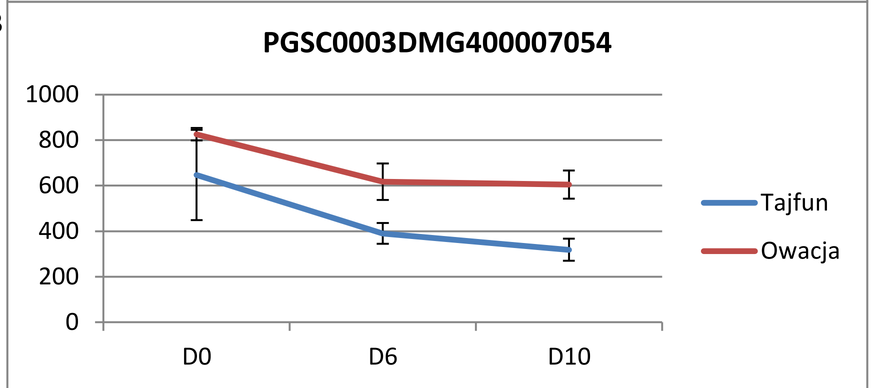

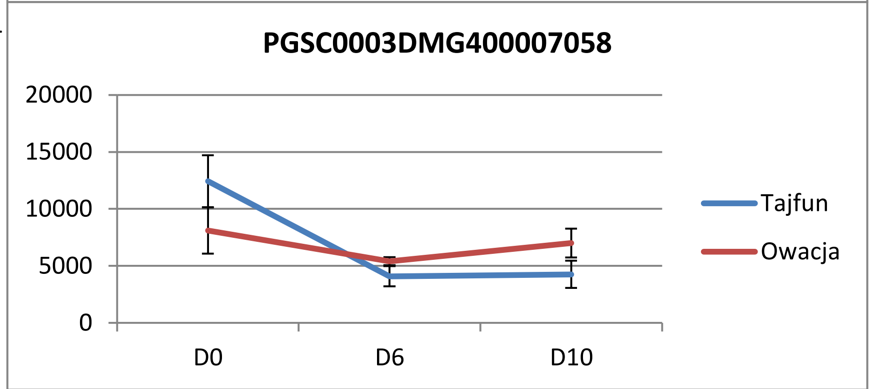

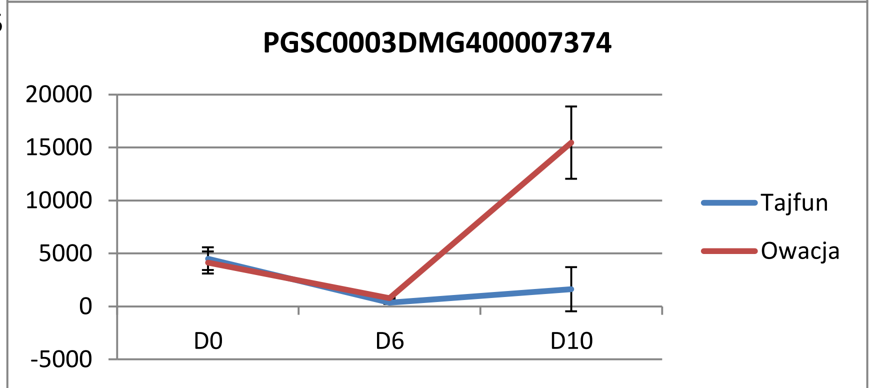

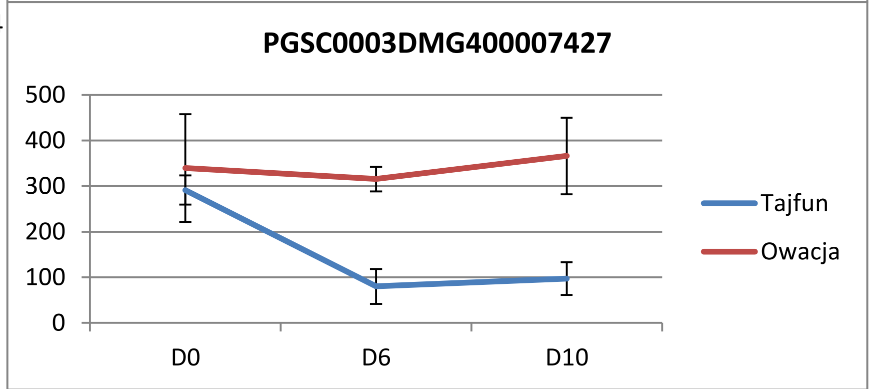

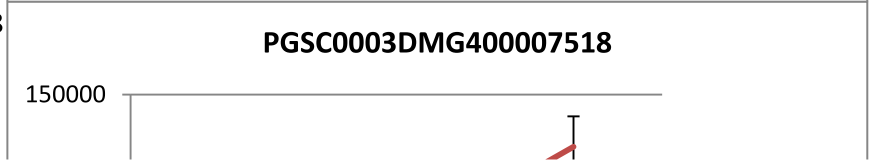

**PGSC0003DMG400007678**	194	41,33	42	149	88,67	4	22,54	15,01	32,74	51,1	15,53	6,93
**PGSC0003DMG400007693**	407,33	232,67	243,33	329,33	414	472,67	62,14	21,94	34,2	48,01	58,41	70,15
**PGSC0003DMG400007740**	583,33	120,67	24,67	430,67	47,33	3,33	100,17	25,32	17,24	138,66	9,24	1,15
**PGSC0003DMG400007787**	20541	2098,7	589,33	21307	1057,3	78,67	2975,8	37,11	388,64	7300,5	188,35	39,11
**PGSC0003DMG400007978**	185,33	28	35,33	178,67	92	98	58,53	2	18,04	23,01	14,42	39,85

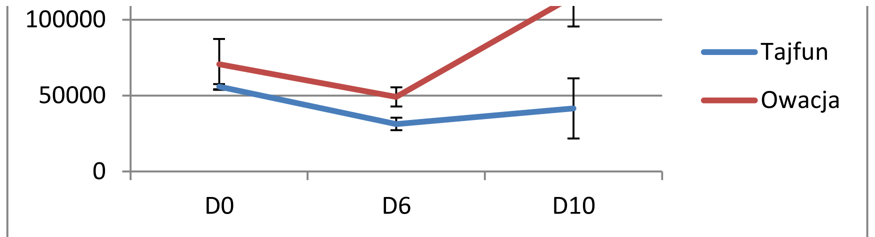

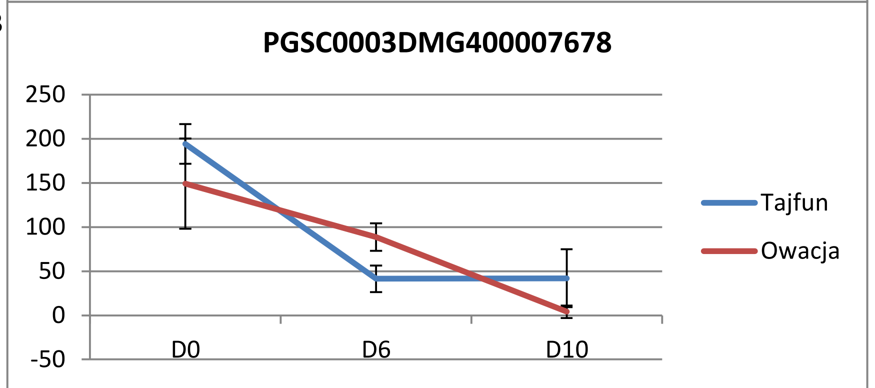

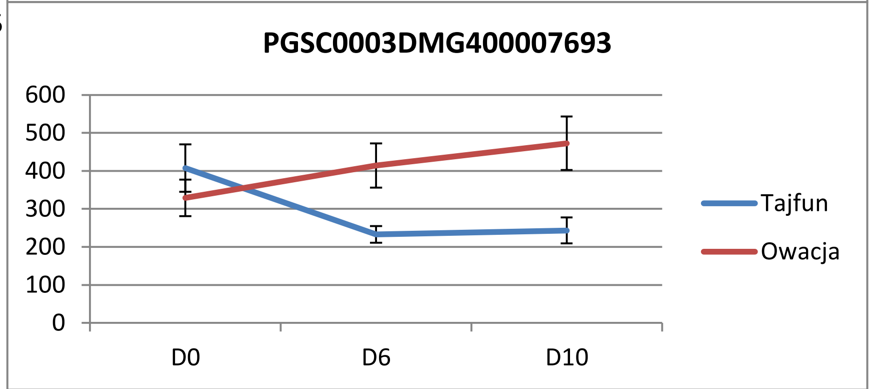

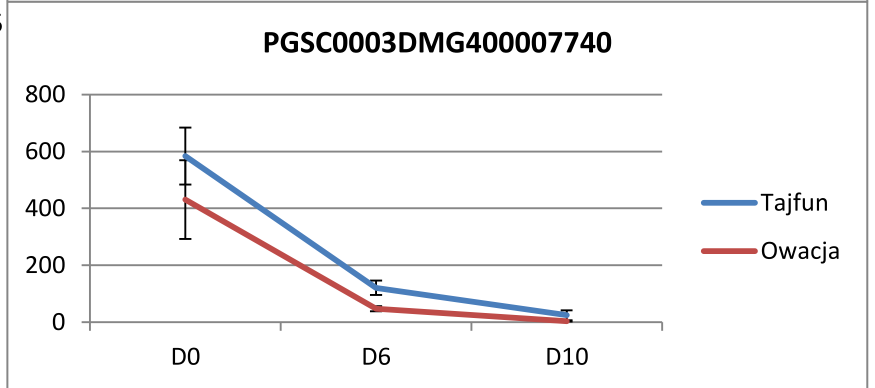

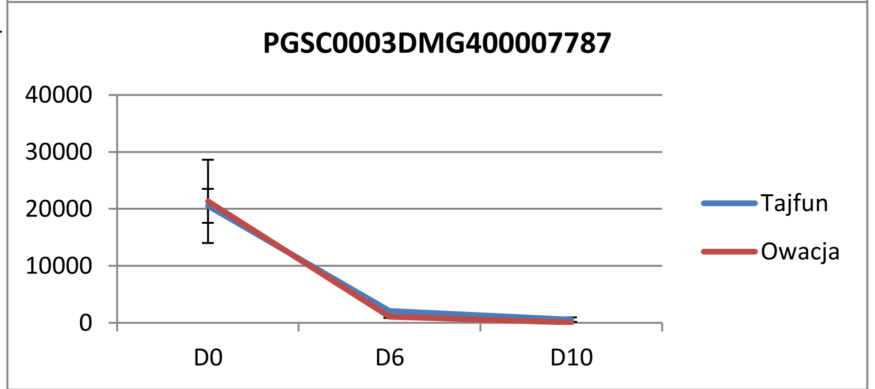

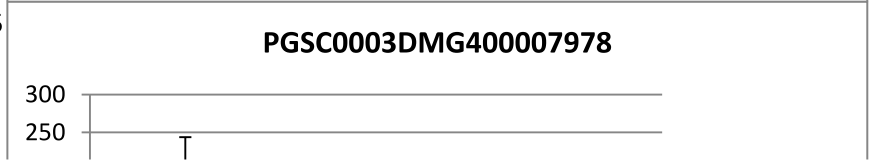

**PGSC0003DMG400007992**	264	102,67	25,33	148	0	0	131,19	35,12	25,01	53,7	0	0
**PGSC0003DMG400008018**	2184	1382,3	330,33	1866,7	2481,3	1976,3	273,73	249,49	309,65	525,96	285,58	442,36
**PGSC0003DMG400008021**	472	48	228	518	166	531,33	109,23	43,86	184,52	190,71	36,72	124,54
**PGSC0003DMG400008262**	948	528,67	498	541,33	805,33	1767,3	391,65	65,43	358,57	147,84	110,59	153,13
**PGSC0003DMG400008326**	2612,7	1906	1736,67	3036,7	2621,3	3592,7	461,87	491,97	340,41	532,79	360,98	238,17

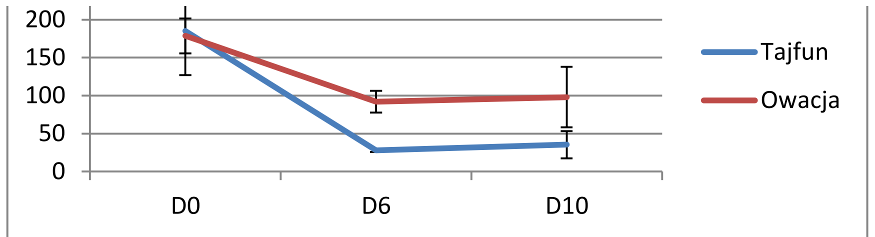

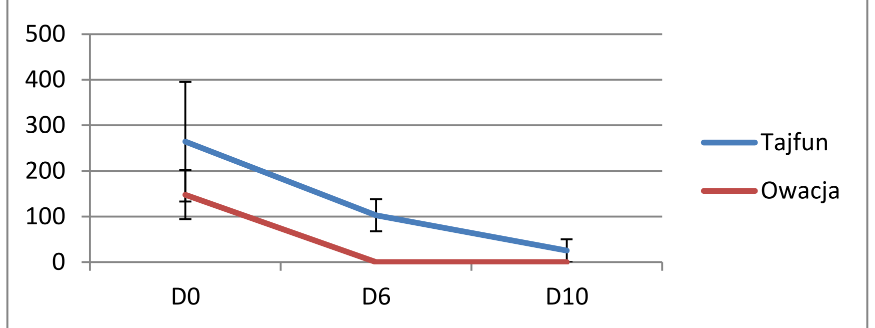

PGSC0003DMG400007992

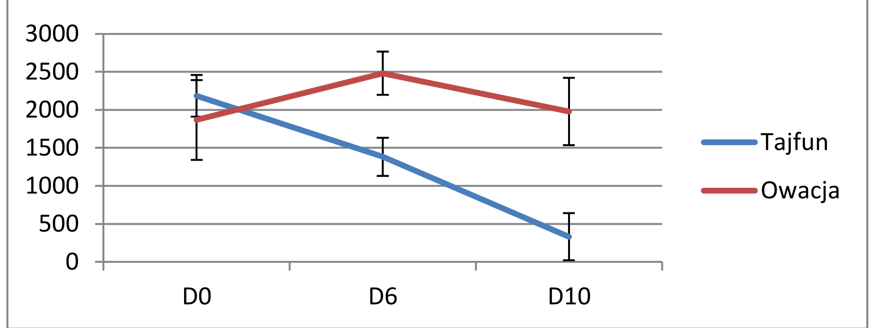

PGSC0003DMG400008018

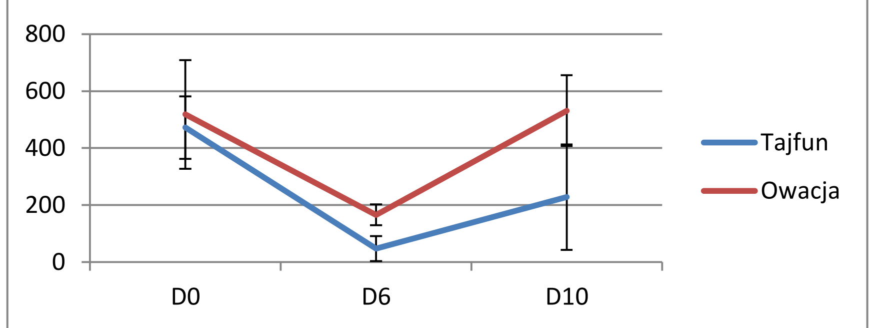

PGSC0003DMG400008021

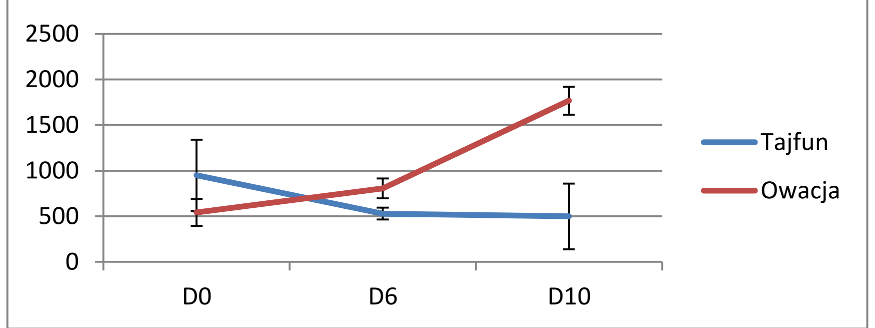

PGSC0003DMG400008262

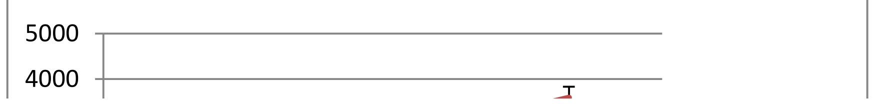

PGSC0003DMG400008326

**PGSC0003DMG400008357**	1234	296,67	156	1141,3	160	46	423,83	47,17	47,03	697,37	54,81	30
**PGSC0003DMG400008504**	7657,3	1325,3	2066	6326	860	5292,7	714,24	41,68	784,53	1528,9	65,57	354,54
**PGSC0003DMG400008584**	19122	6236,7	3015,33	12203	4060	4591,3	4736,9	170,58	127,22	4783,8	534,71	358,12
**PGSC0003DMG400008587**	2751,3	1369,3	1102	2230	1968,7	1564,7	596,74	133,78	321,74	455,08	265,01	61,33
**PGSC0003DMG400008834**	890,67	171,33	81,33	716,67	42,67	16	216,37	55,94	34,02	85,33	8,08	8,72

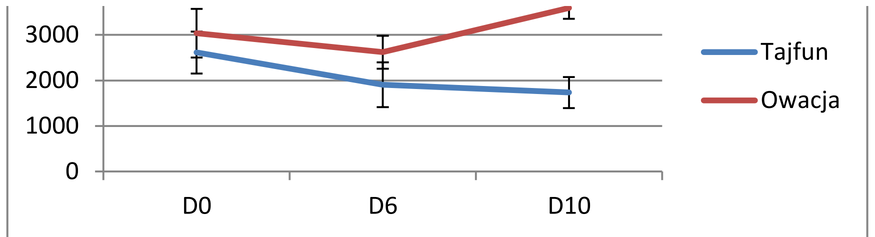

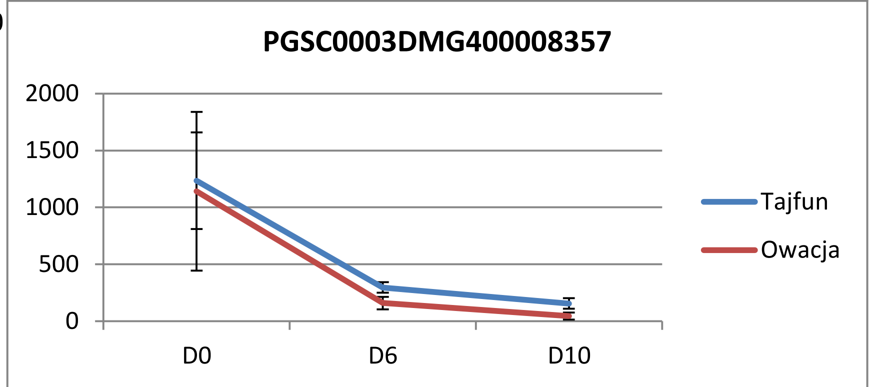

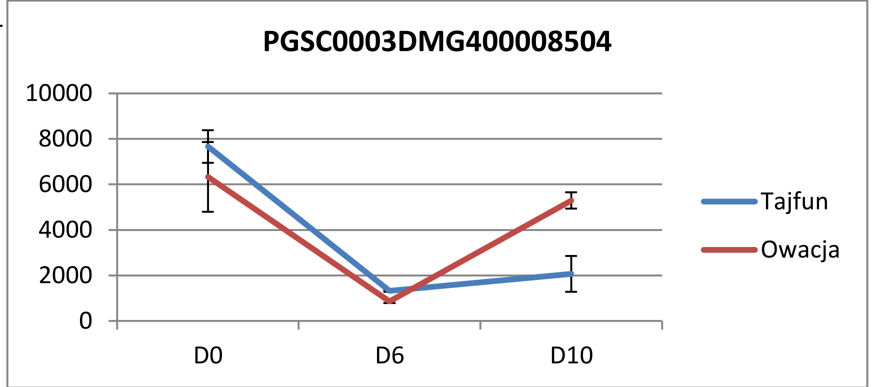

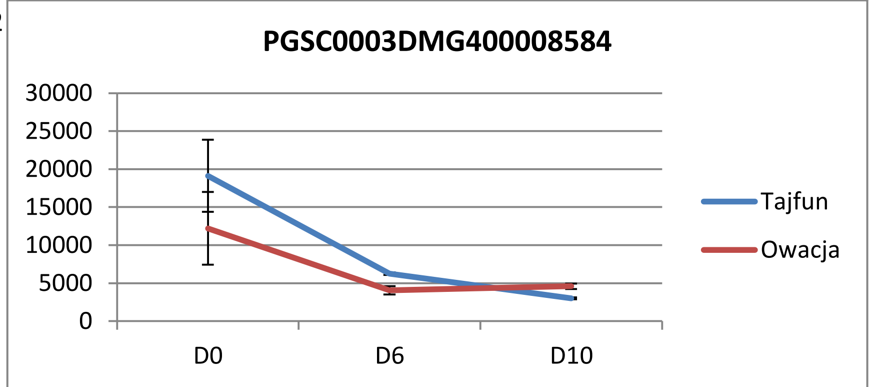

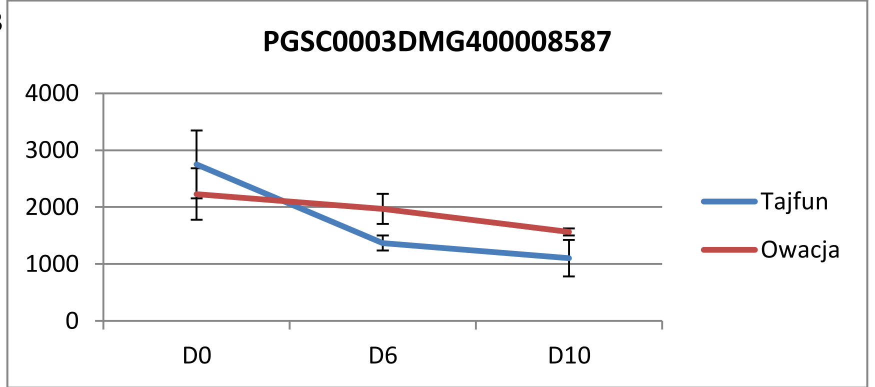

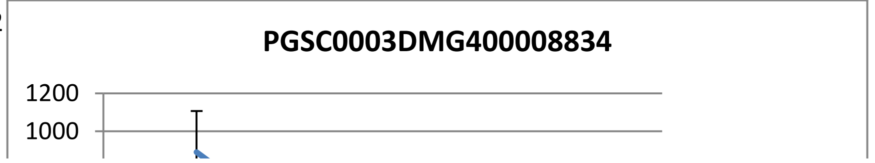

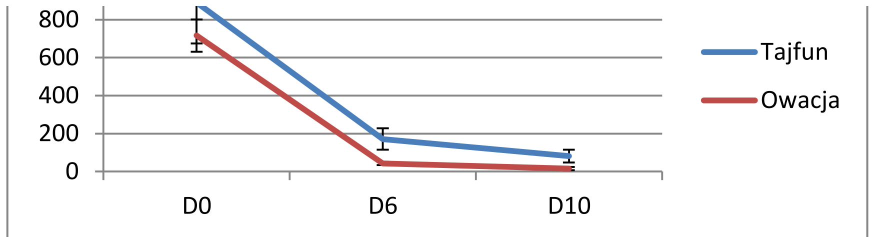

**PGSC0003DMG400009072**	8898	6984	3076	6968	11353	5940,7	2405,9	503,07	604,66	2749,7	408,25	1038,1
**PGSC0003DMG400009456**	6700,7	2695	3100,33	8294,7	4900	4411	1157,1	814,8	454,93	707,8	1026,02	350,25
**PGSC0003DMG400009984**	893,33	346	364,67	868	676	576,33	86,63	125,97	35,12	250,14	184,52	177,33
**PGSC0003DMG400010010**	1410,7	672,67	475,33	1801	1312,7	849	98,66	163,02	209,42	251,49	266,55	280,34
**PGSC0003DMG400010468**	1444	1240	951,67	960,67	527,33	223	391,8	161,65	347,84	338,03	124,38	39,36

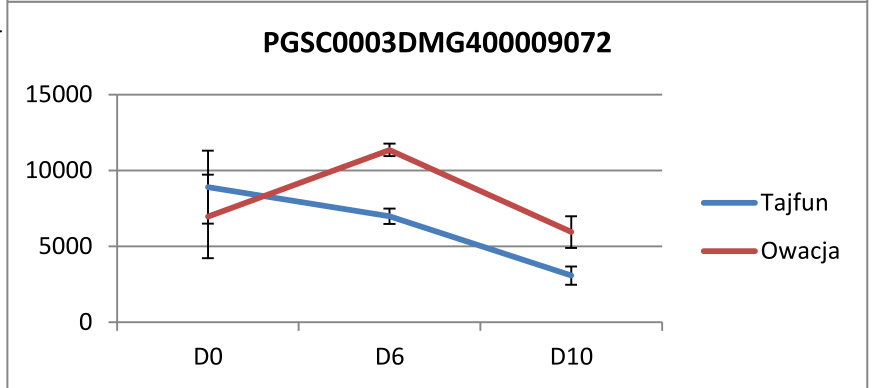

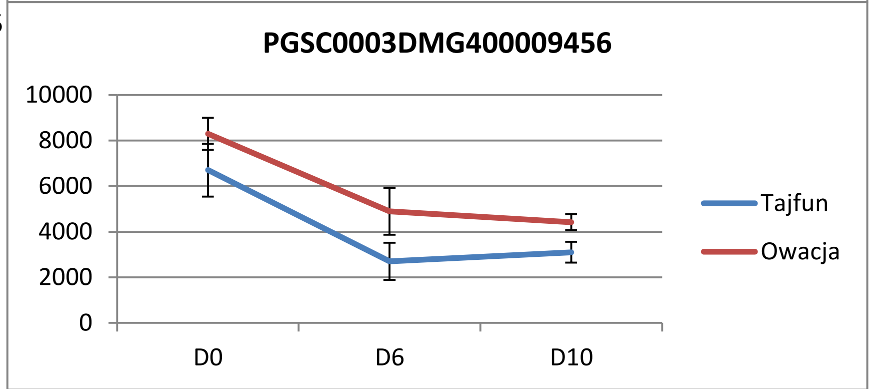

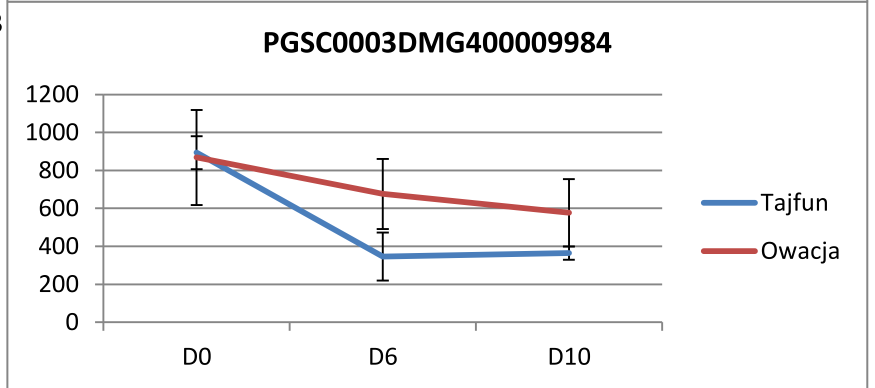

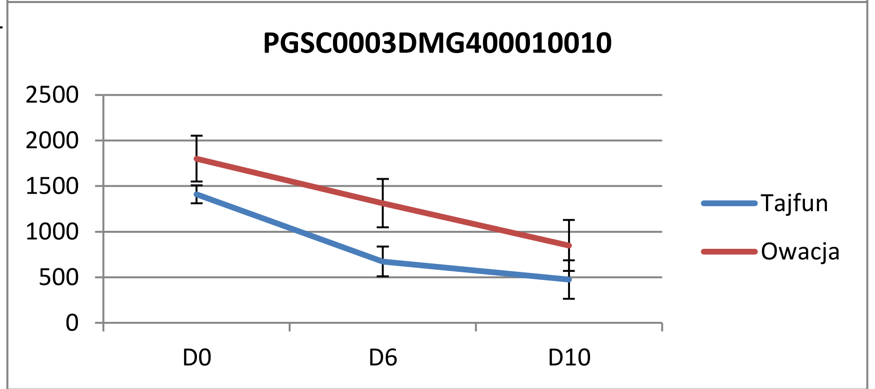

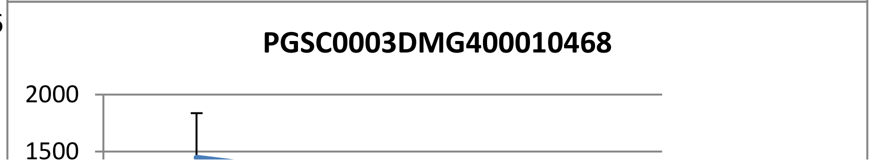

**PGSC0003DMG400010477**	7500,7	280	112	5094	158	14	1024,4	26,91	32,92	102,06	47,29	13,11
**PGSC0003DMG400011013**	2249,3	1842	1027,33	1726,7	1168,7	372	276,1	307,82	308,05	346,07	215,5	111,12
**PGSC0003DMG400011044**	1820,7	384	320	2278,7	652	498,67	63,07	121,01	57,86	453,26	66,36	63,29
**PGSC0003DMG400011321**	3656,3	892,67	1289,33	3765	2092	3904	507,04	123	90,72	967,16	173,01	605,12
**PGSC0003DMG400011406**	2555,3	569,33	90,67	1644,7	310,67	26,67	141,57	132,85	50,29	669,9	163,59	7,02

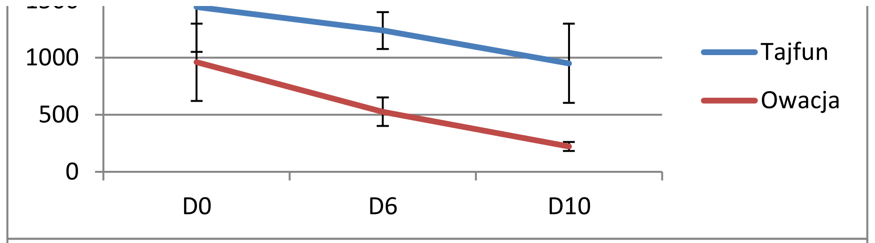

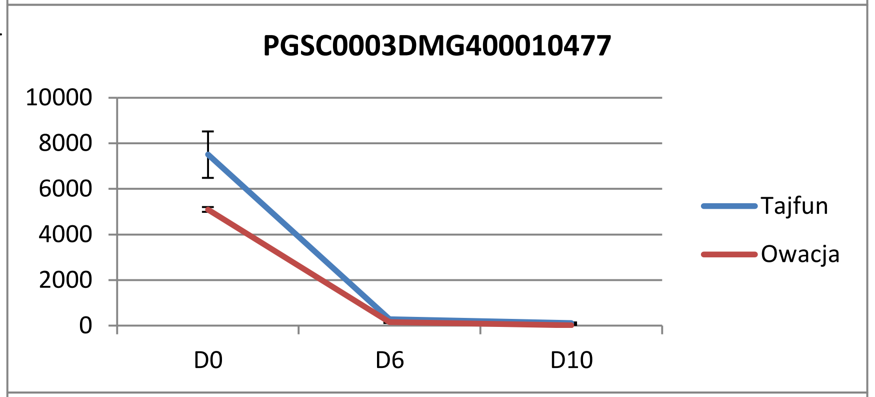

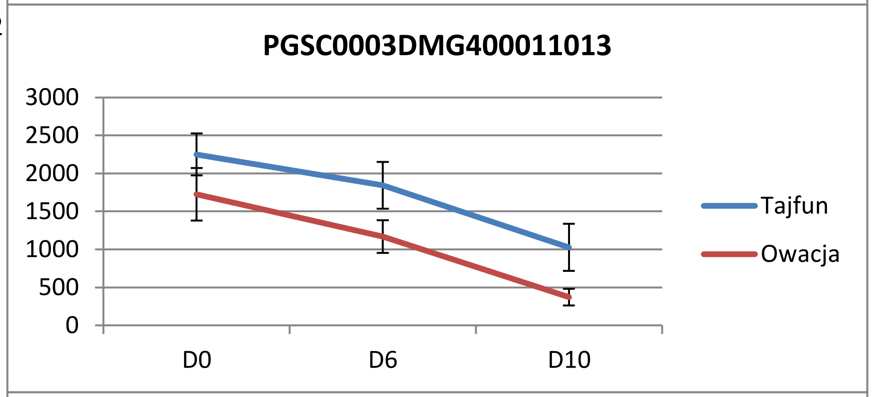

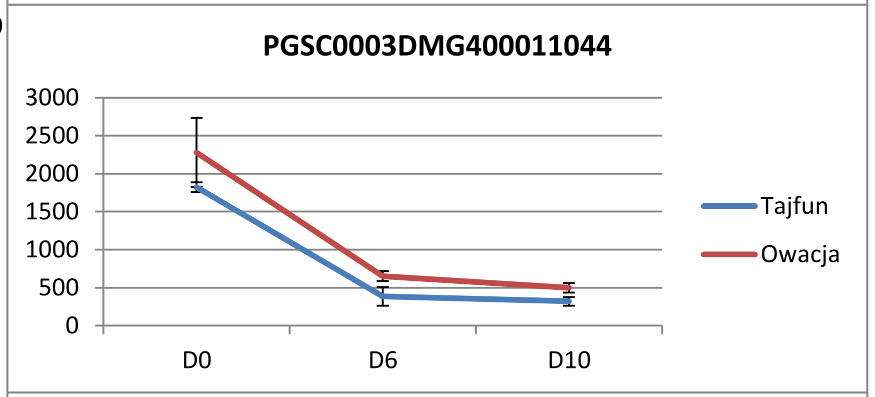

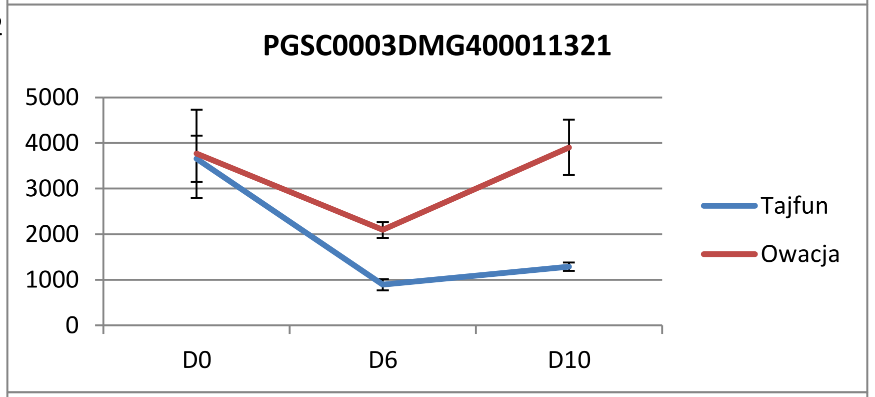

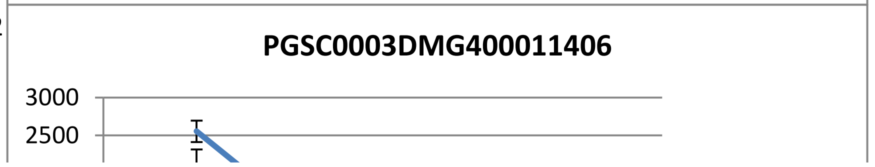

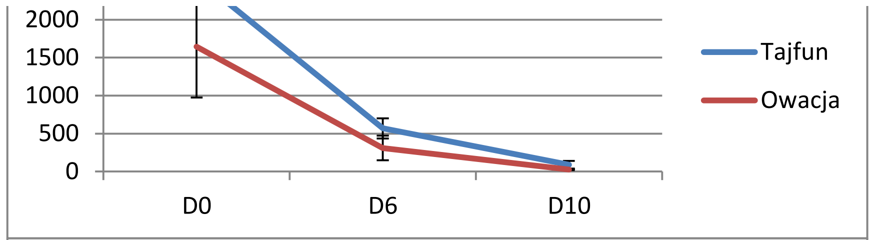

**PGSC0003DMG400011750**	1726	726	931,33	3052	361,33	482	229,63	162,37	43	1442,8	112,43	145,09
**PGSC0003DMG400011811**	1329,3	915,33	756,67	1230,7	1378	1242	206,73	100,29	126,64	96,03	139,56	53,33
**PGSC0003DMG400011842**	480,67	164	100	604	256	318	133,81	24,33	83,74	111,34	31,43	68,23
**PGSC0003DMG400012224**	5594,7	1340	629,33	3619,3	800,67	122,67	276,55	179,21	464,13	543,31	242,79	9,24
**PGSC0003DMG400012265**	5136	2379,3	2039,33	4414,7	1722	724,67	694,73	353,28	550,22	289,62	277,01	141

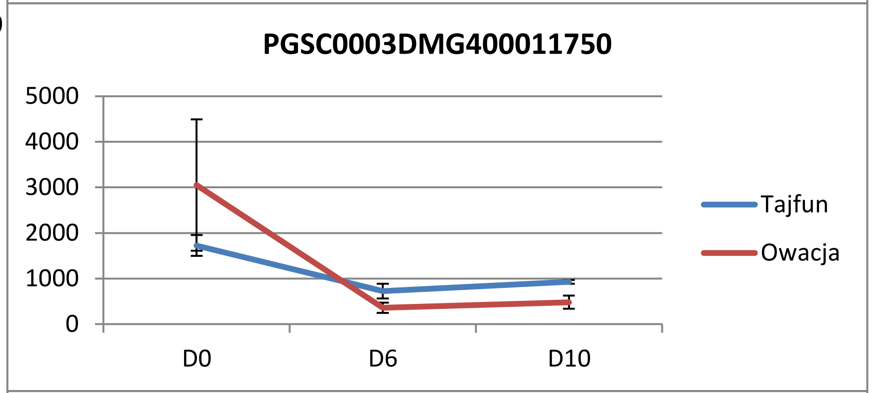

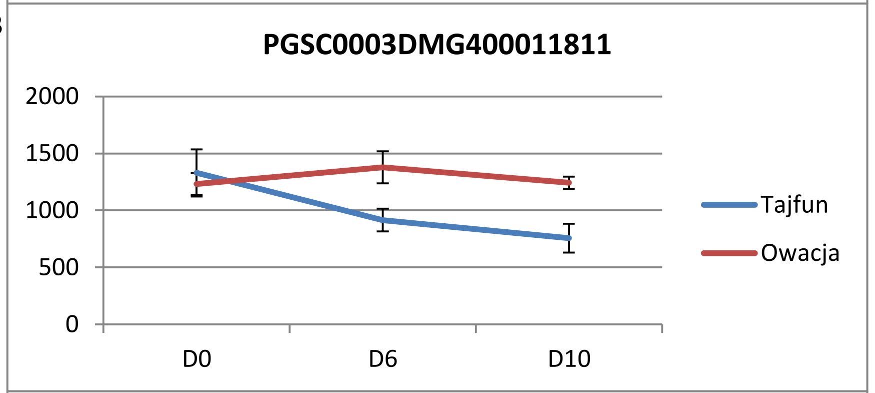

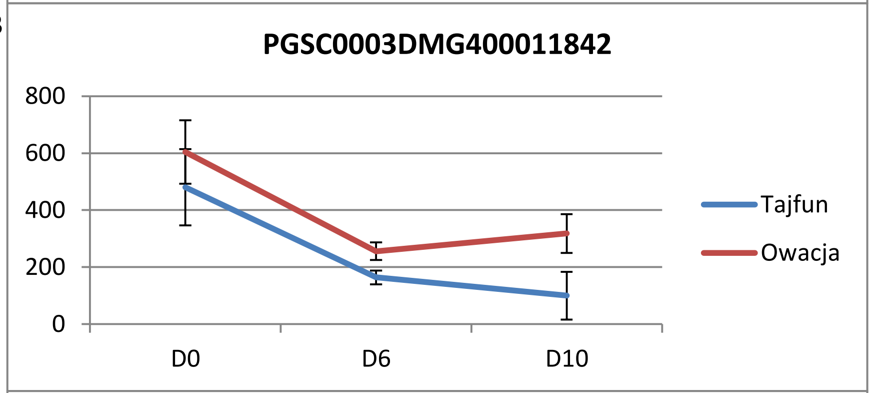

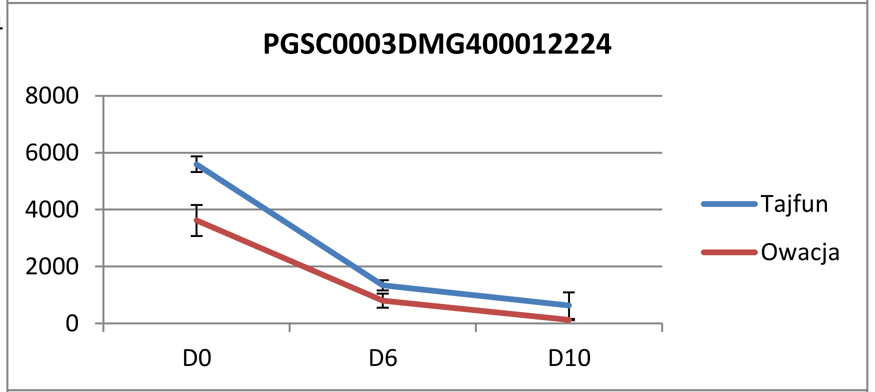

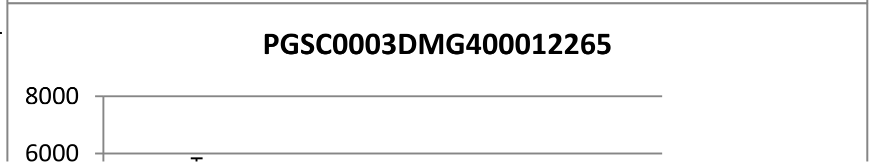

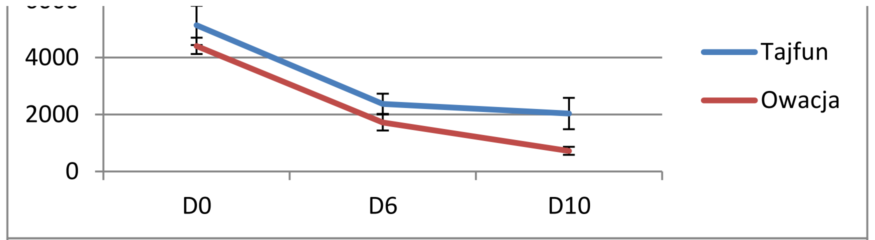

**PGSC0003DMG400012571**	2768,7	1439,3	1952,67	2729,3	2028,7	3285,3	376,43	183,04	757,96	464,57	202,43	321,65
**PGSC0003DMG400012614**	3917,3	1041,3	1286,67	4444	1748	2703,3	251,92	327,84	97	936,34	435,67	292,24
**PGSC0003DMG400012631**	2911,3	2197,3	816,67	1906,7	3282,7	1845,3	523,09	318,95	308	543,33	424,38	22,3
**PGSC0003DMG400012696**	3997,3	1924,7	1797,33	3713,3	2602	2849	676,21	439,05	175,3	1201,8	98,24	112,53
**PGSC0003DMG400013297**	7328	4362,7	4072	8132,7	7874	28241	590,5	1512,6	2504,12	797,39	1612,37	1369,9

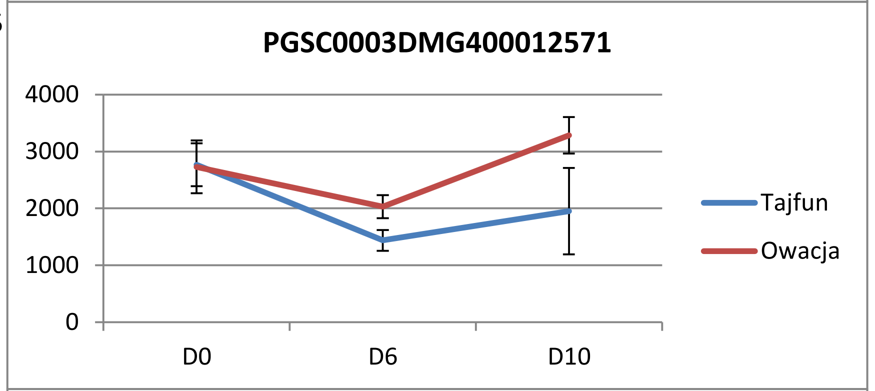

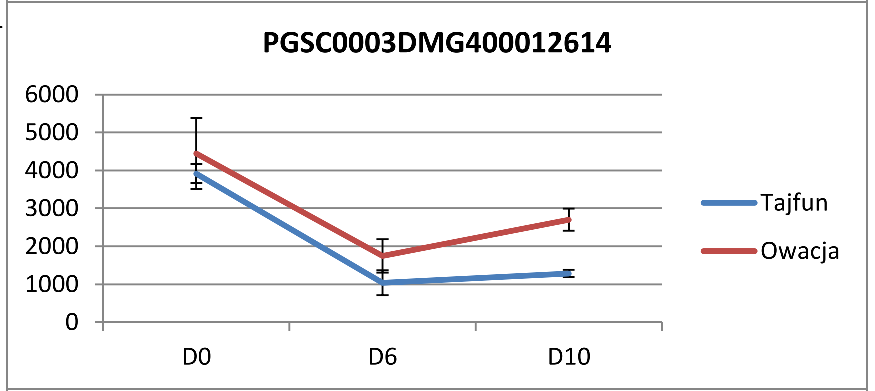

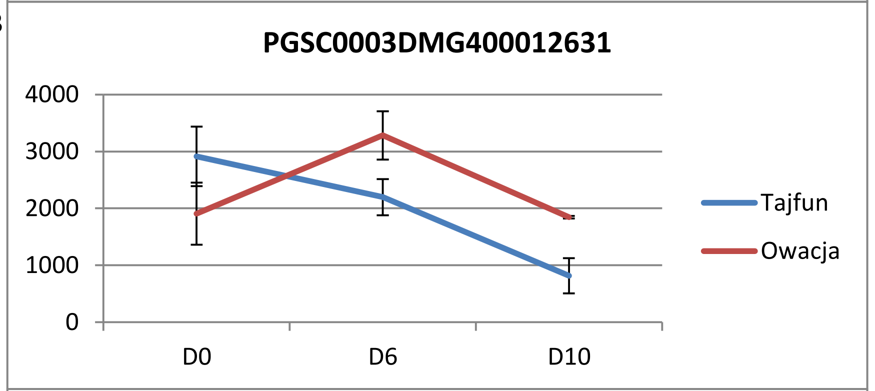

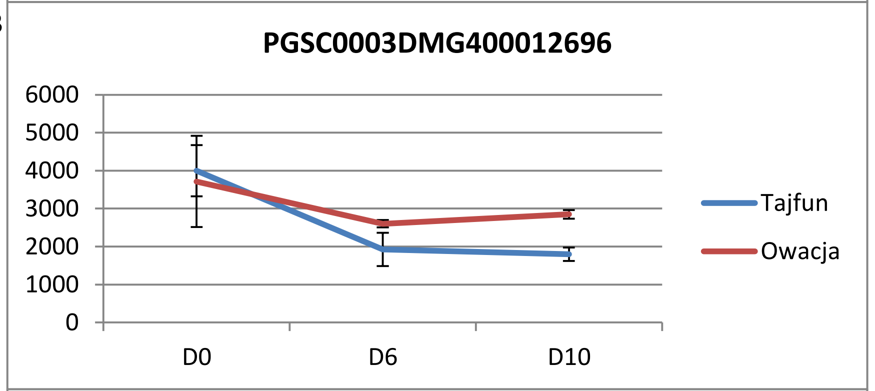

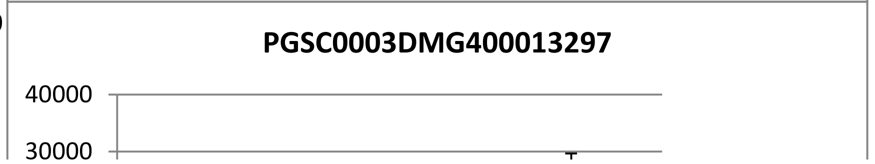

**PGSC0003DMG400013412**	40714	14623	8560	28406	3745,3	1642	13070	1656,1	6007,76	10712	280,02	558,87
**PGSC0003DMG400013437**	4422,7	1789,3	1798	4722	2618,7	2820,7	789,95	239,84	26,15	480,01	330,03	281,21
**PGSC0003DMG400013461**	7899,3	837,33	230	5462	174,67	25,33	3291,3	183,09	149,08	2063,1	36,3	5,03
**PGSC0003DMG400013537**	370,67	39,33	113,33	366	158	800	192,79	17,93	87,76	173,52	53,25	148,96
**PGSC0003DMG400013660**	458	328	155,33	587,33	634	248,67	67,02	66,09	39,31	195,14	147,25	27,15

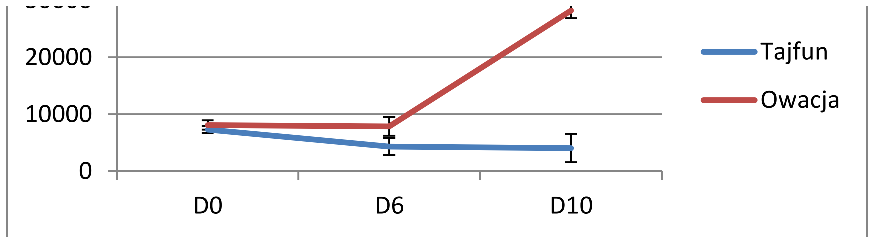

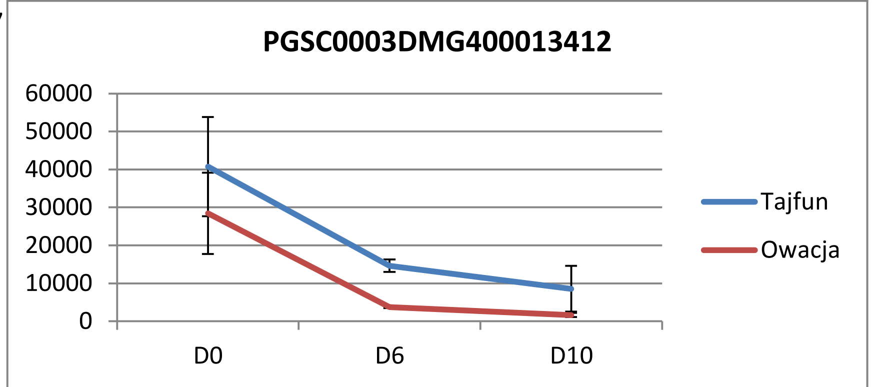

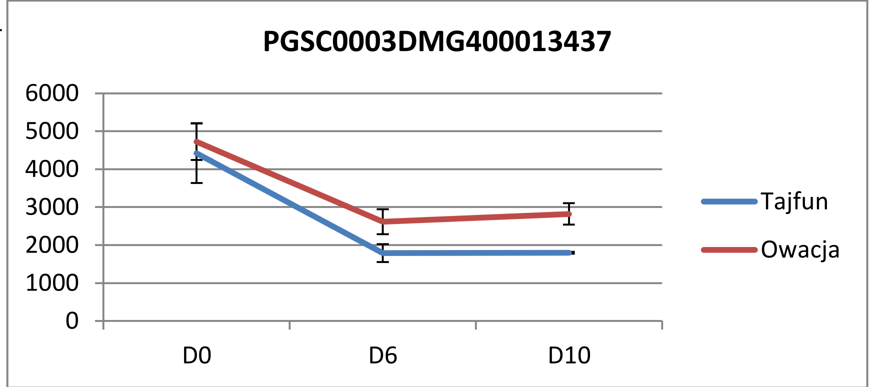

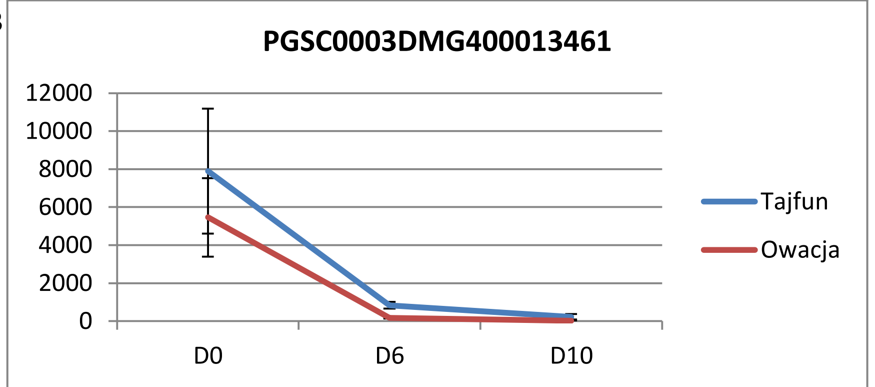

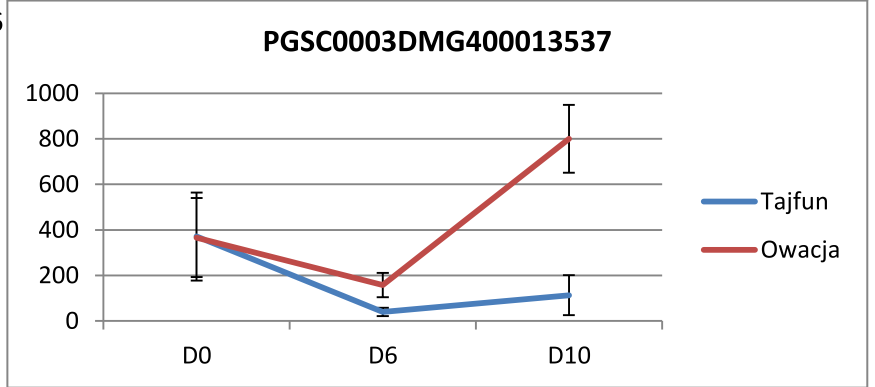

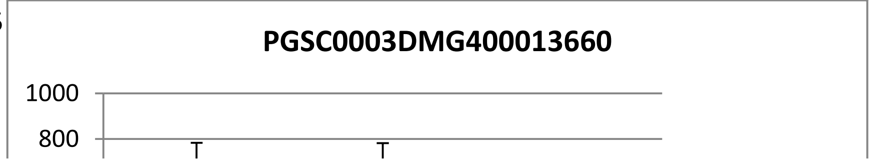

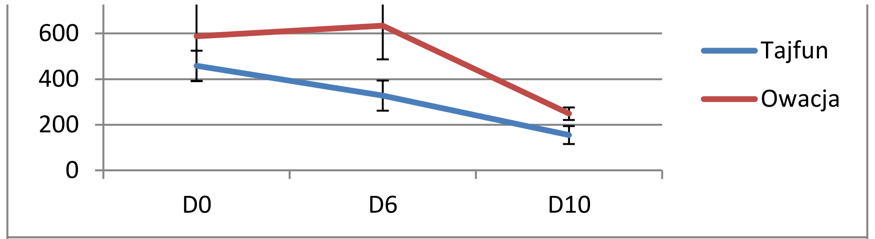

**PGSC0003DMG400014421**	5730	2969,7	3027,33	5305,3	4569,3	4059,3	1231,8	293,16	430,59	961,39	316,31	513,9
**PGSC0003DMG400014954**	1480	183,33	94,67	1076,7	878,67	662,67	76,08	34,02	55,22	420,82	137,44	91,24
**PGSC0003DMG400015154**	1528	1317,3	927,33	1919,3	2706	1977,3	233,62	163,64	223,27	485,92	281,25	347,71
**PGSC0003DMG400015347**	278	64,67	33,33	250	24	8,67	54,15	35,57	6,43	87,16	5,29	3,06
**PGSC0003DMG400015665**	858,67	429,33	388	1270,7	745,33	754,67	149,56	47,38	124,72	451,27	97,29	240,01

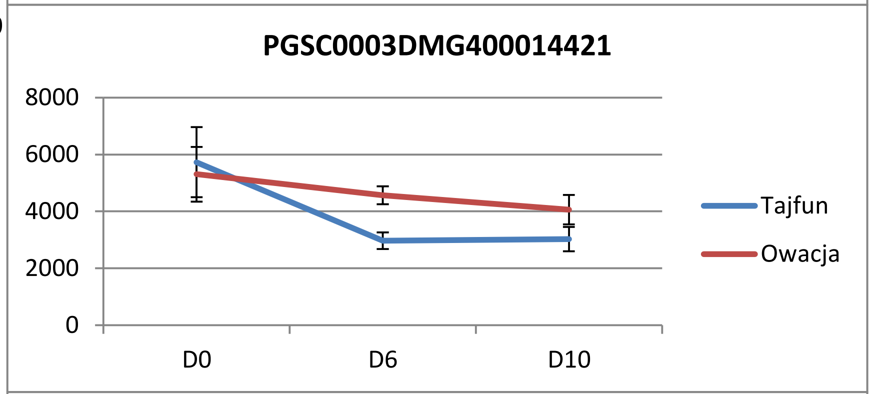

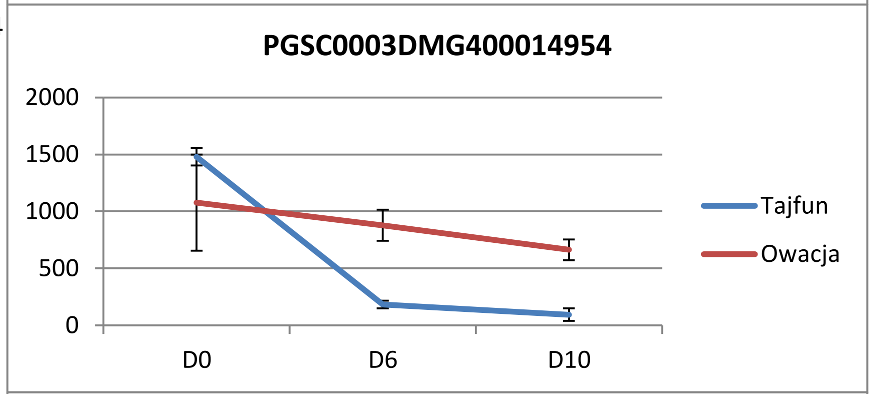

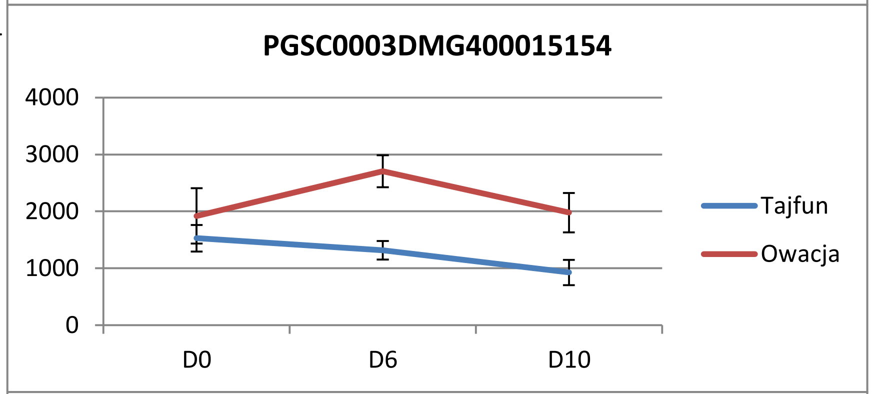

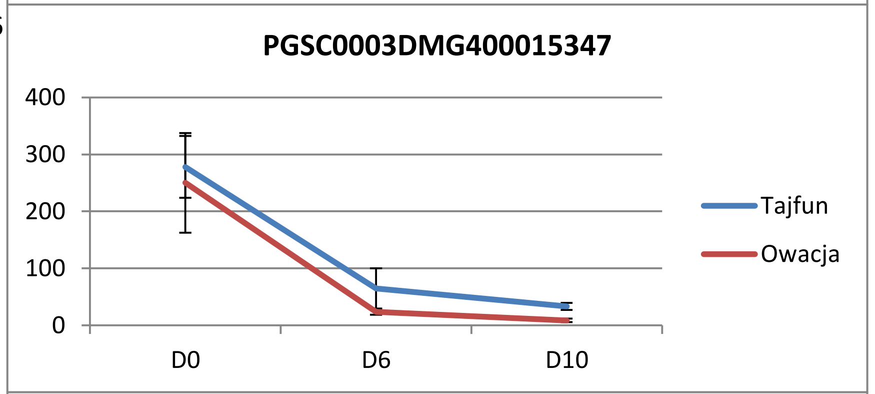

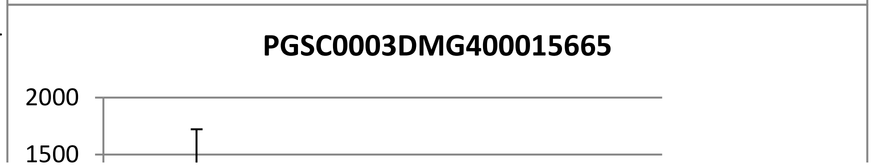

**PGSC0003DMG400016120**	2231,3	909,33	845,33	1954,7	1226	2032	511,55	107,04	126,26	724,74	92,26	342,41
**PGSC0003DMG400016221**	968,67	310	139,33	1159,3	166,67	288	192,25	31,43	57,49	434,98	45,09	51,26
**PGSC0003DMG400016317**	4080	1608	1136,67	2854,7	885,33	386,67	574,32	336,07	133,06	432,99	436,58	66,4
**PGSC0003DMG400016319**	3812,7	822,67	787,33	3760	2178	2569,7	792,11	119,49	462,09	1124,2	563,96	389,3
**PGSC0003DMG400016829**	6476	2955,3	2957	7968	4583	4055	1379,7	209,06	895,61	556,02	1119,66	459,84

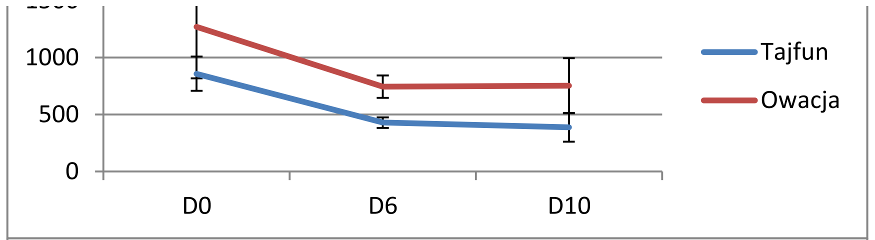

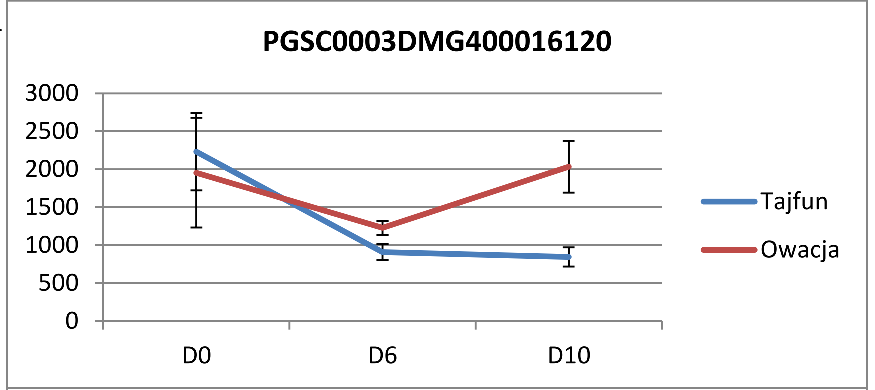

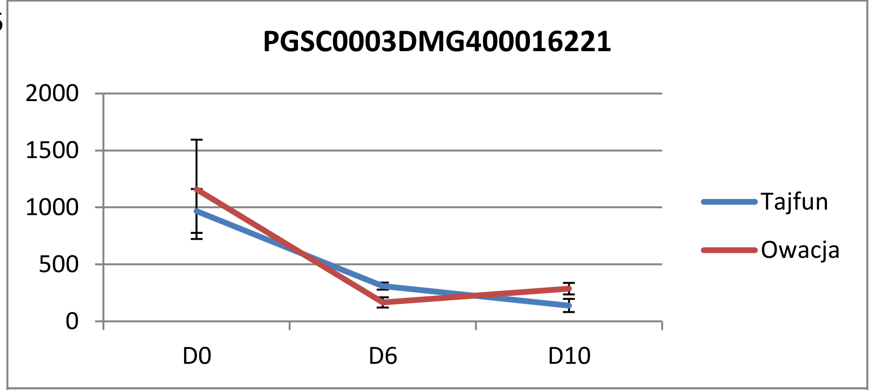

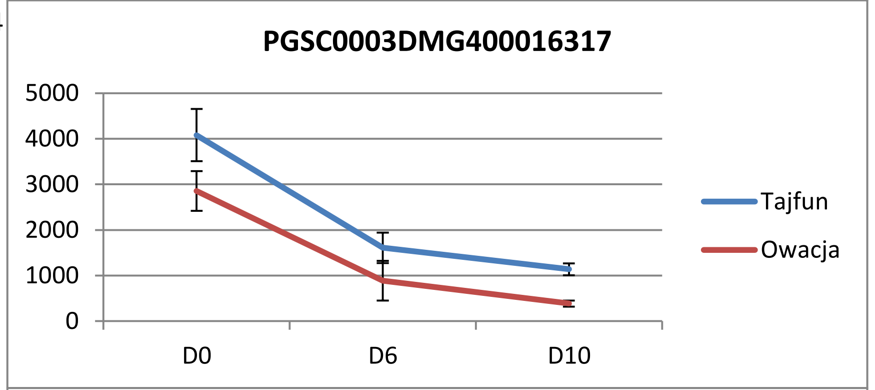

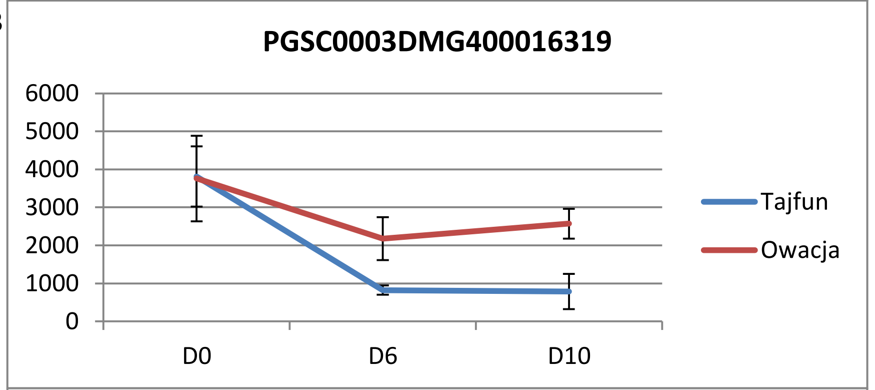

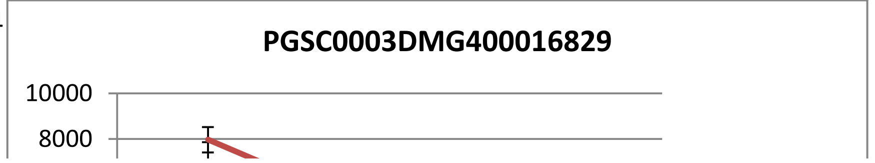

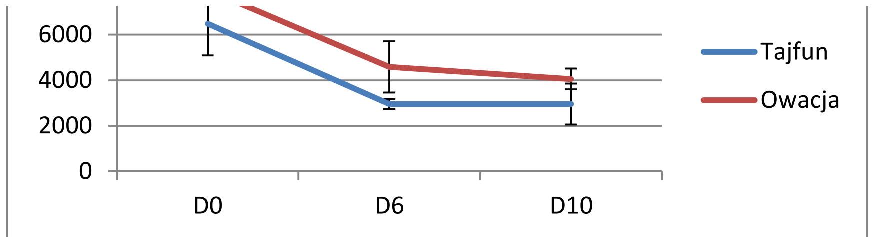

**PGSC0003DMG400017110**	15622	1470	1246	10831	3128	5432,7	3407,1	326,9	792,55	3494,2	164,88	1234,3
**PGSC0003DMG400017181**	887,33	317,33	467,33	1023,3	500,67	1399,3	192	26,1	264,06	194,22	67,89	167,02
**PGSC0003DMG400017396**	1940,7	1600,7	1330	1519,3	2267,3	1789,3	495,91	195,81	313,58	787,8	268,96	93,09
**PGSC0003DMG400017560**	389,33	93,33	125,33	470,67	259,33	486,67	63,29	28,02	49,89	106,31	24,11	88,12
**PGSC0003DMG400017570**	11946	3442	2656,67	10702	2422,7	1387,3	1949	558,76	527,18	1797,9	379,92	306,34

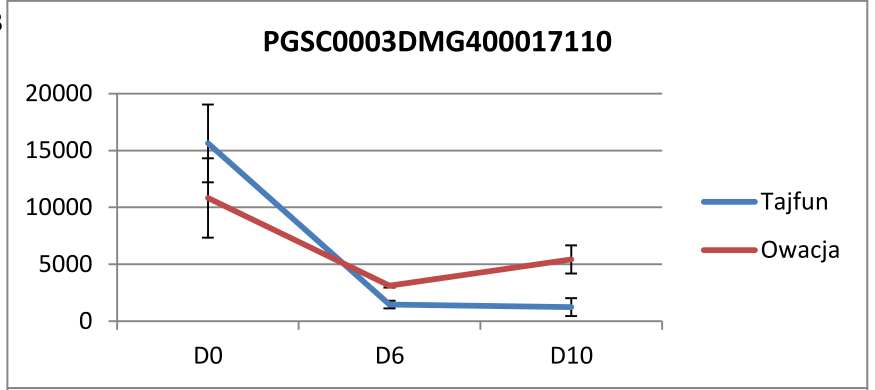

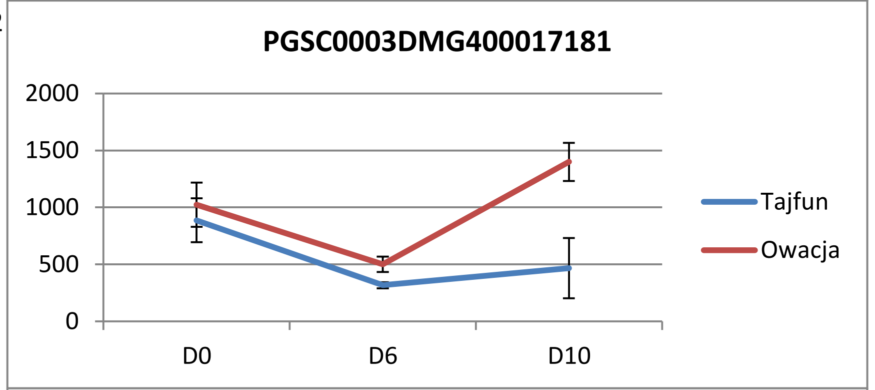

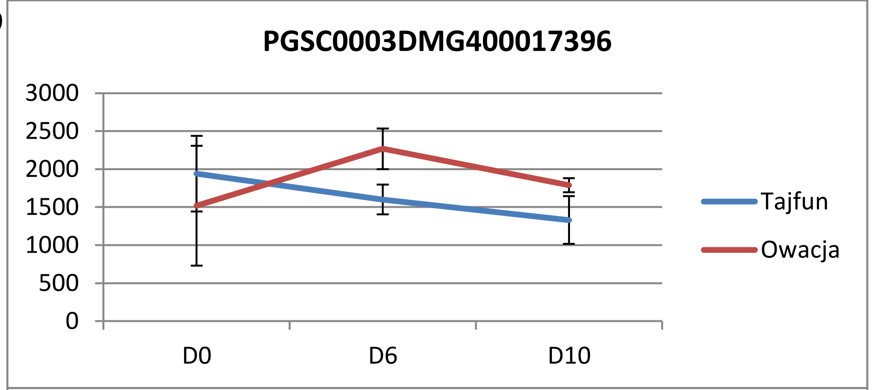

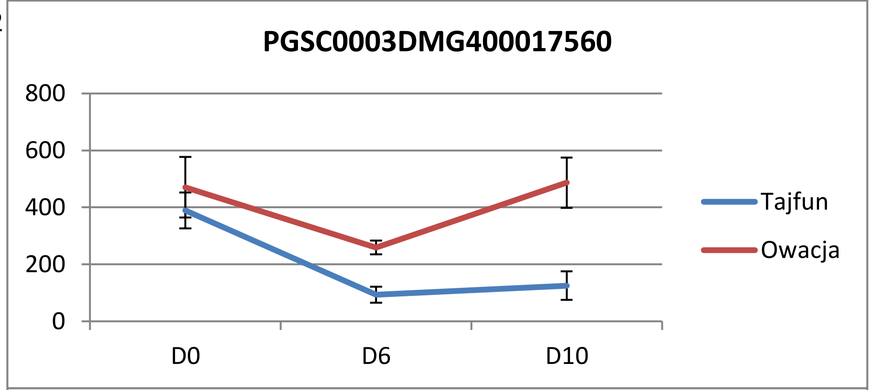

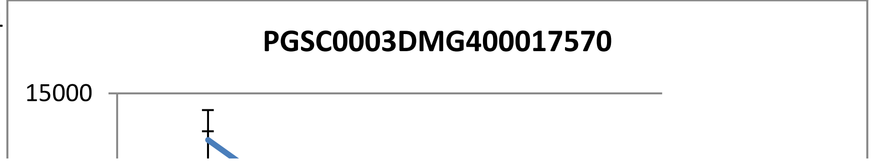

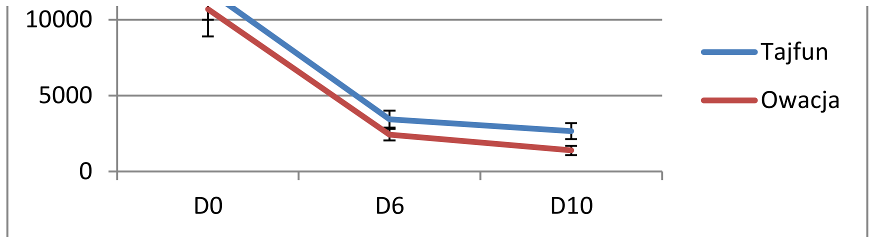

**PGSC0003DMG400017738**	438	58,67	22,67	763,67	117,67	130	78,71	16,65	10,07	430,59	14,29	23,07
**PGSC0003DMG400018141**	3108,7	424	210,67	3292,7	820	924,67	705,41	68,79	158,51	1263,3	119,72	172,39
**PGSC0003DMG400018194**	2164	888,67	499,33	2008	2540,7	1122	502,3	359,58	166,14	659,57	494,52	161,15
**PGSC0003DMG400018224**	3640	1080	632	4438	1619,3	1181,3	243,01	124,71	64,28	681,7	191,34	40,81
**PGSC0003DMG400018422**	4148	1426	1533,33	2852,7	2342	857	598,82	183,01	209,42	1563,5	364,81	154,62

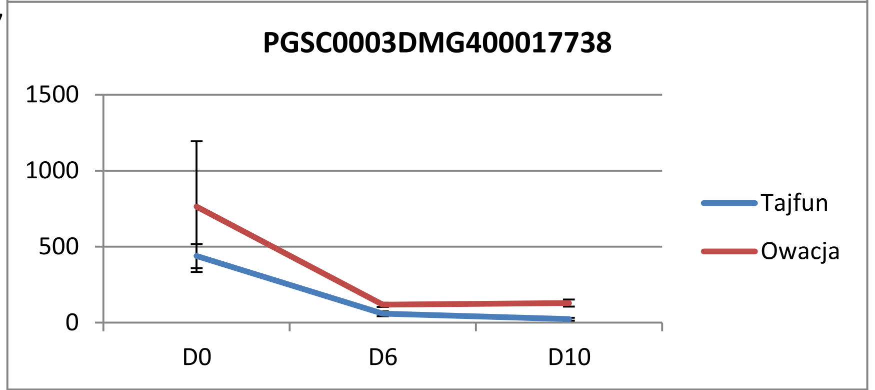

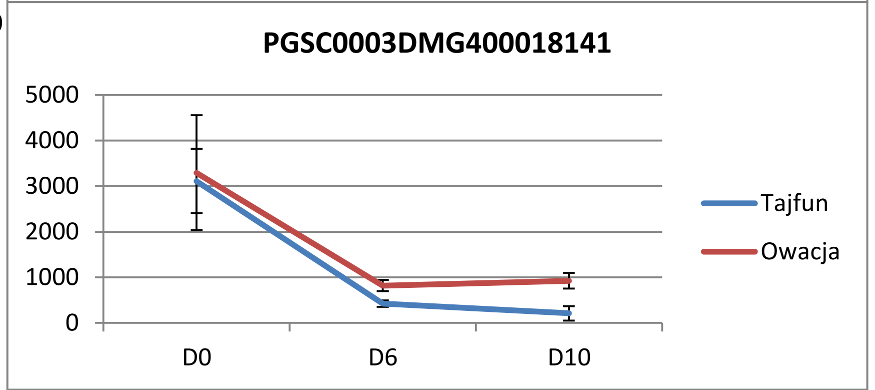

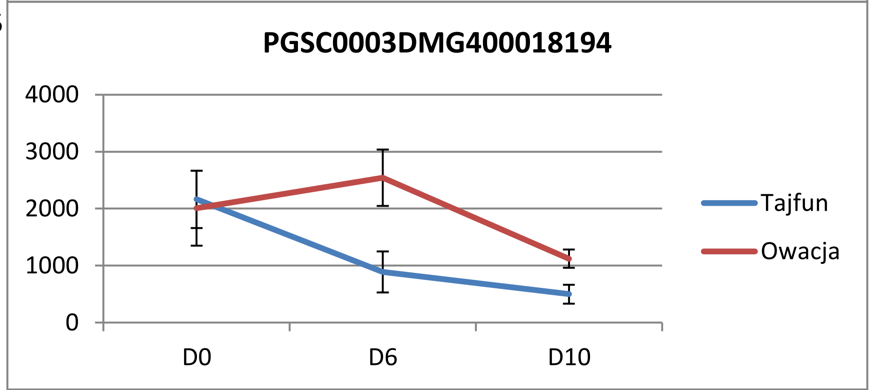

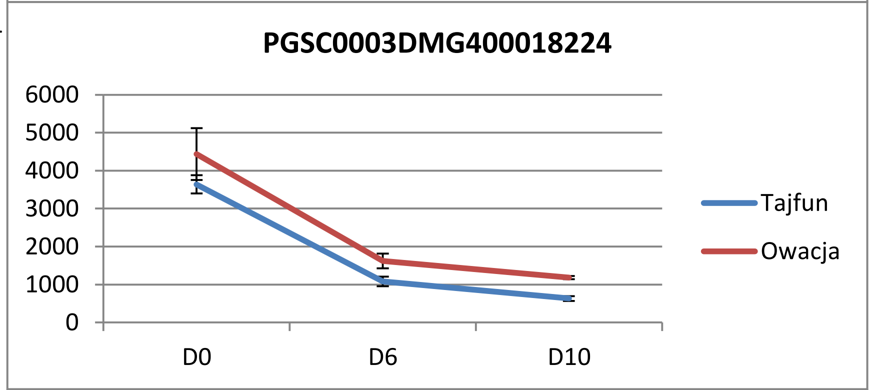

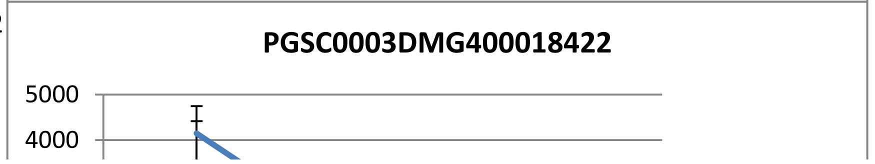

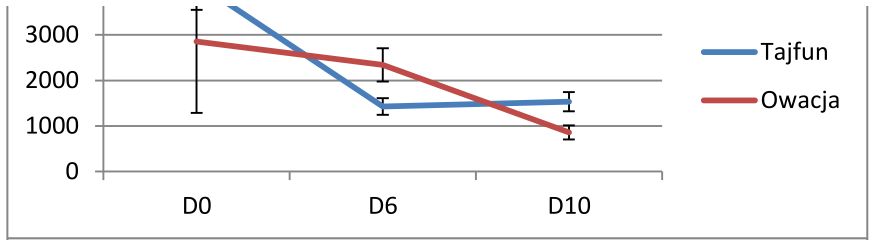

**PGSC0003DMG400018598**	3083	2726	1334,67	2475,3	1803,7	682,67	536,74	377,23	465,94	924,39	169,82	69,12
**PGSC0003DMG400018678**	889,33	374	197,33	821,33	1014,7	548,67	232,49	25,06	85,1	323,62	135,6	77,78
**PGSC0003DMG400019191**	1098	950	823,33	757,33	660	470	145,01	90,07	142,57	200,26	238,6	83,59
**PGSC0003DMG400019241**	2358	482,67	1258,67	1502	857,33	2610	236,08	58,53	111,5	411,97	277,1	620,08
**PGSC0003DMG400019254**	42336	15199	9906	28557	8912,7	5660	2367	627,46	2670,02	2833,8	371,05	238,97

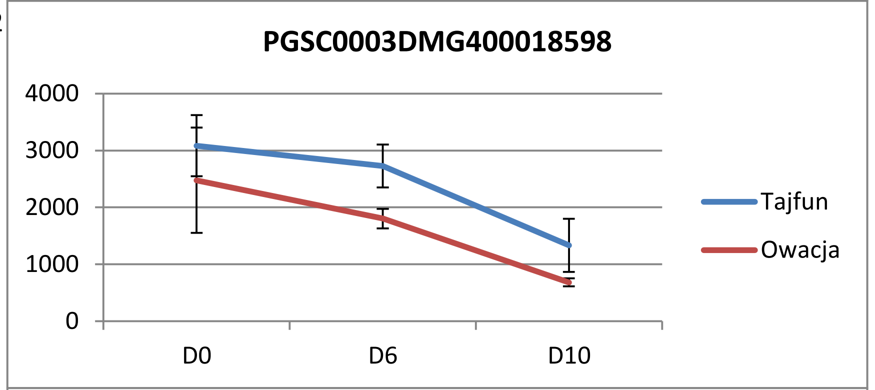

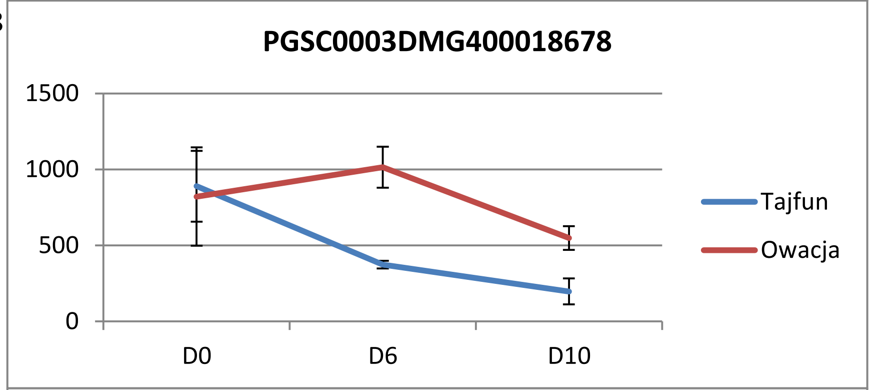

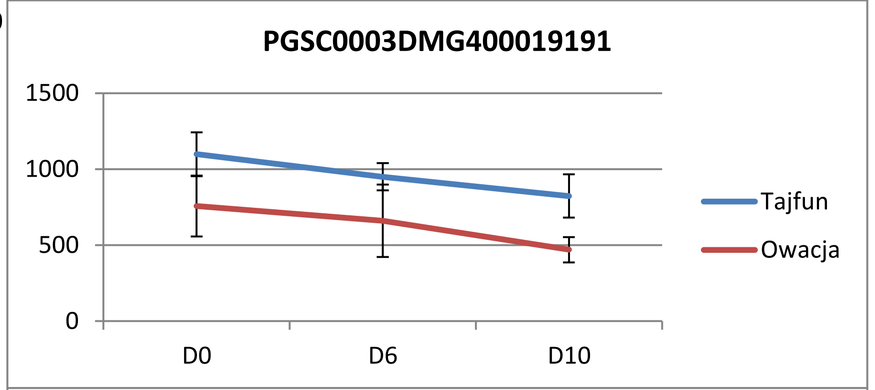

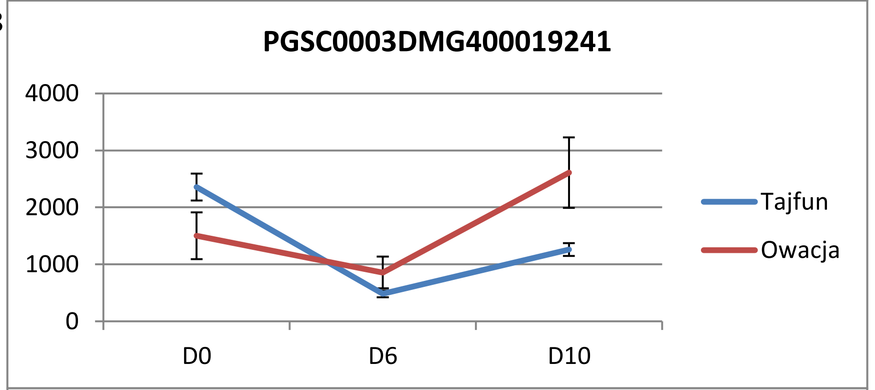

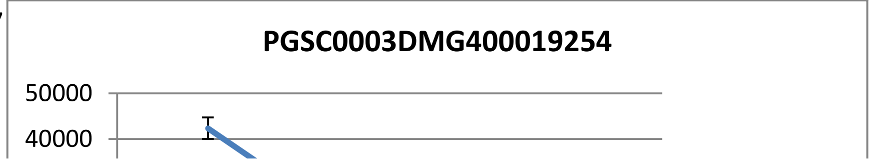

**PGSC0003DMG400019521**	71709	40012	27690,7	46518	28124	12751	1528,4	3361,4	10645,2	7493,9	691,18	1489,3
**PGSC0003DMG400019872**	2980	1322,7	877,33	2143,3	2816	3519,3	1294,6	471,3	61,23	404,26	273,43	799,08
**PGSC0003DMG400020105**	3674	1624	1024,67	2784,7	2168,7	1439,3	868,39	114,84	238,46	1300,7	159,53	99,12
**PGSC0003DMG400020118**	519,67	176	248	472,67	444,33	615	145,31	26	102,35	90,16	56	113,45
**PGSC0003DMG400020619**	1345,3	870	249,33	1346,7	1433,3	820	397,6	98,97	54,93	189,17	195,14	287,76

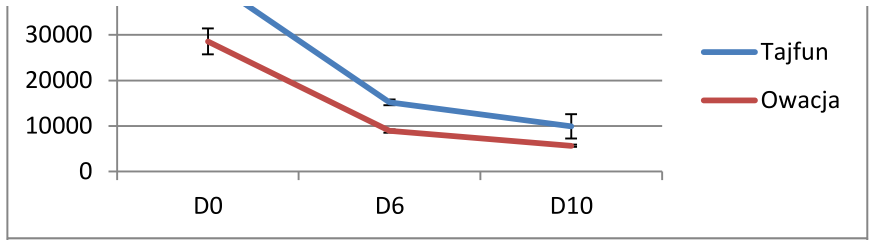

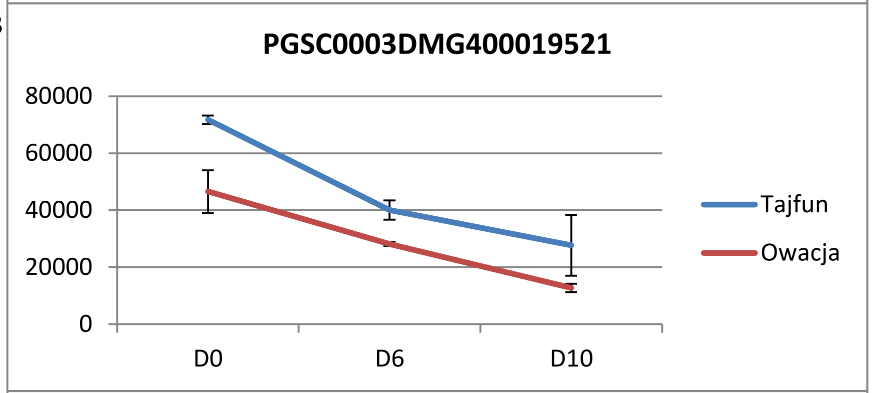

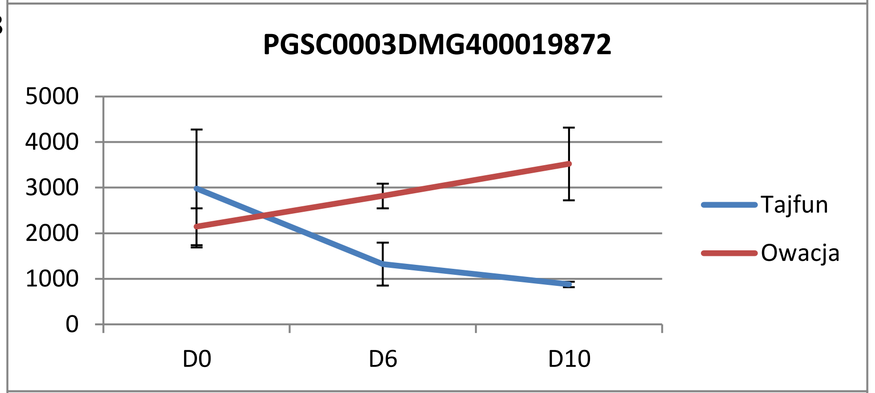

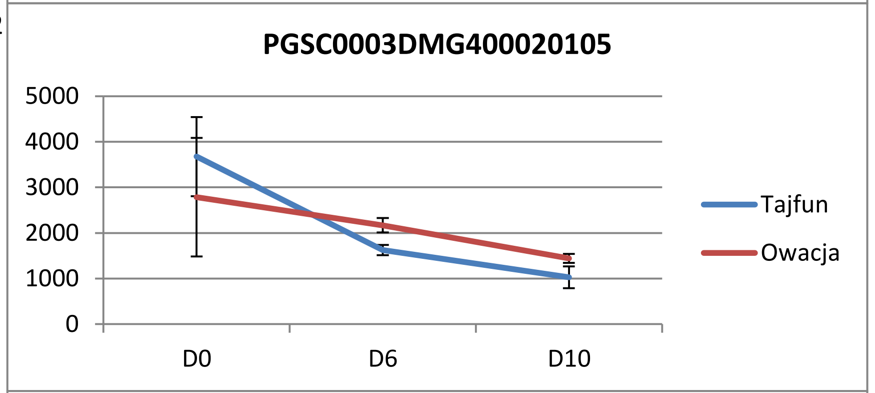

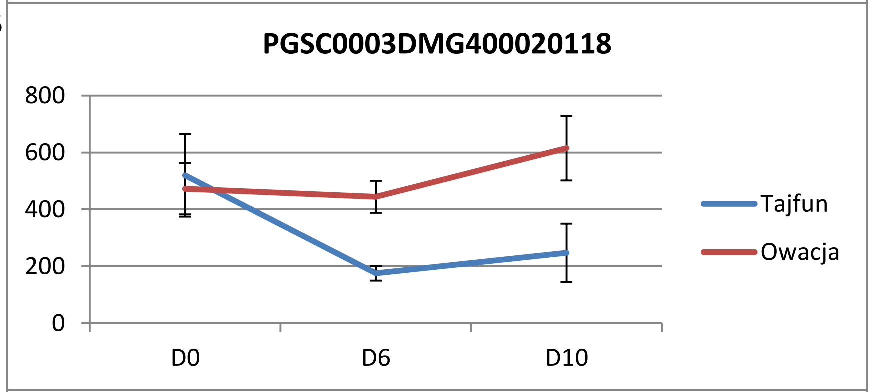

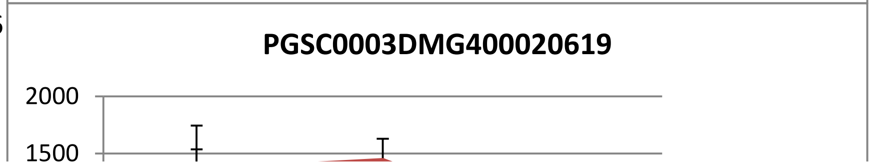

**PGSC0003DMG400020906**	2748,7	1786	353,33	1862,7	916,67	89,33	552,21	562,61	144,46	599	222,03	13,01
**PGSC0003DMG400021008**	230	76	24	184,67	134	4,67	95,14	27,78	14	25,48	36,66	1,15
**PGSC0003DMG400021072**	1860,7	482,67	638	2168,7	762,67	1054,7	273,22	56,08	287,06	427,65	79,36	58,05
**PGSC0003DMG400021398**	11040	6467	960,67	9965	4110,7	390,67	305,29	1724,7	432,15	2512,1	248,78	82,4
**PGSC0003DMG400021550**	366,67	100,67	46,67	467,33	326,67	124	24,44	42,06	7,02	173,87	67,21	24,58

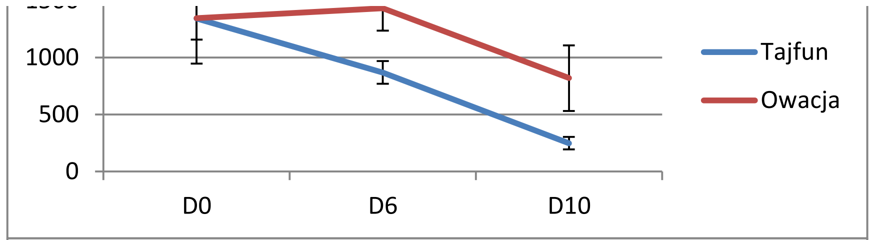

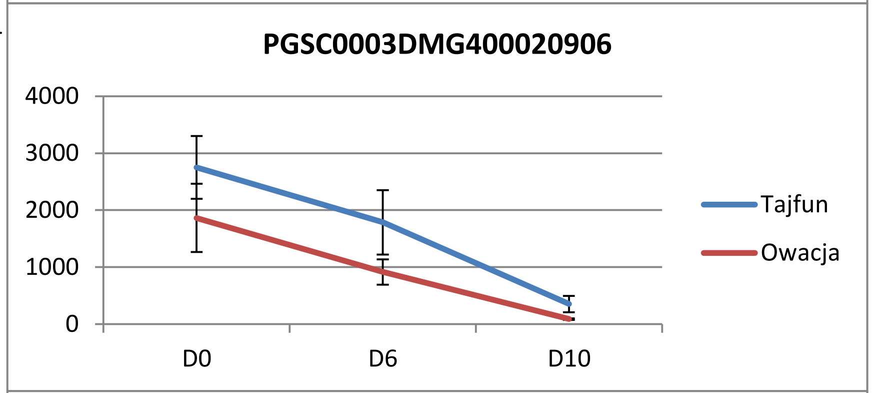

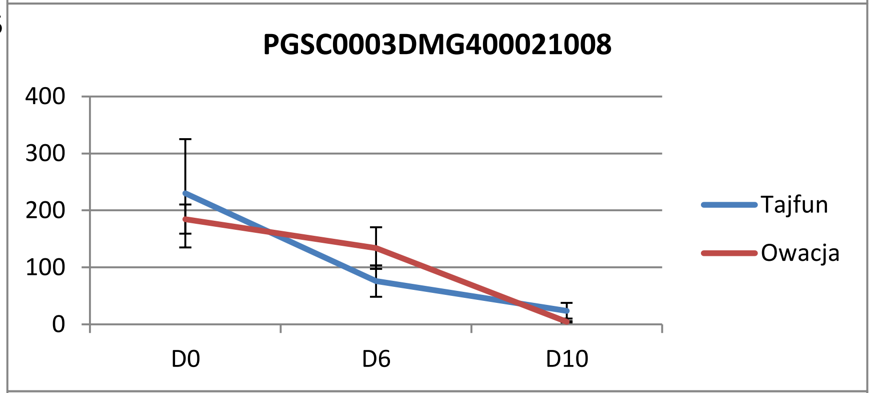

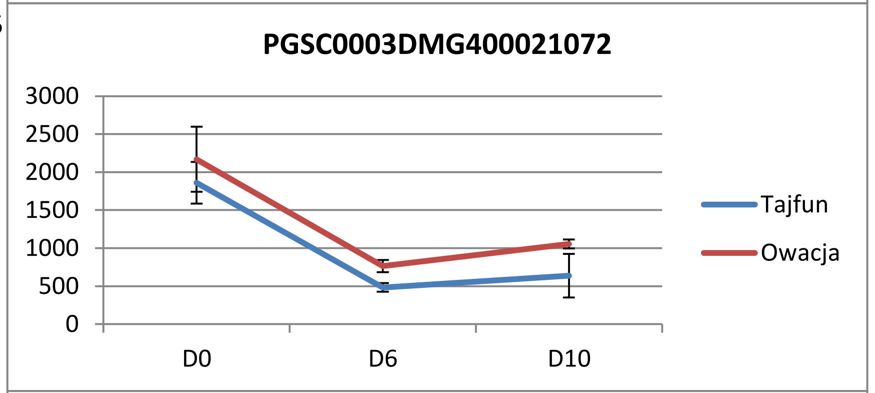

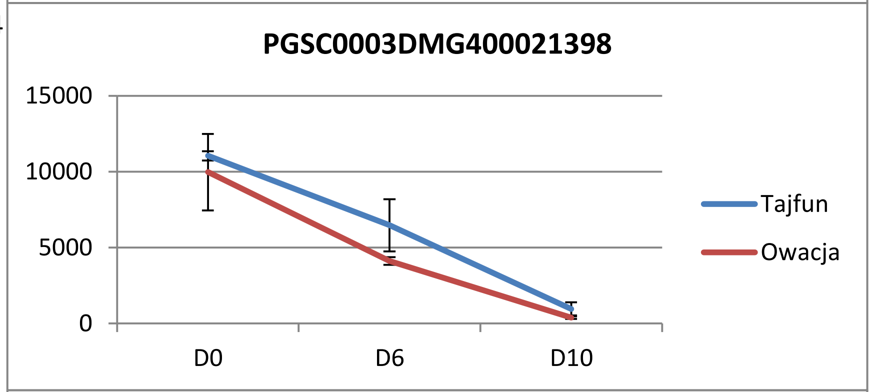

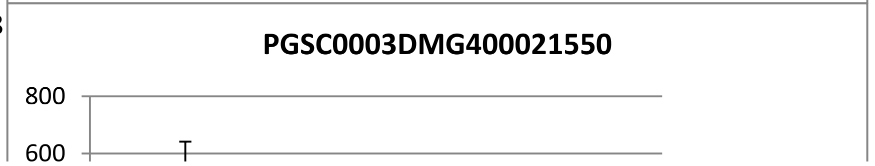

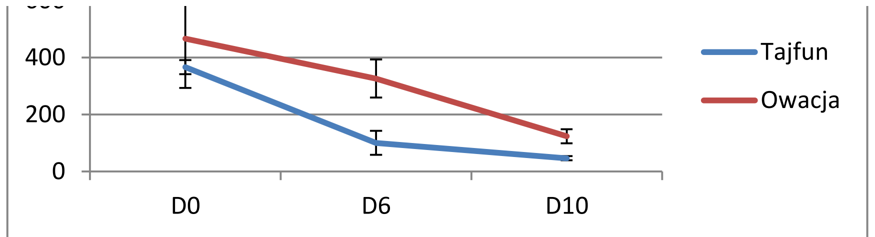

**PGSC0003DMG400021769**	1186	140,67	40,67	758	50,67	3,33	217,17	58,29	31,01	391,4	20,03	3,06
**PGSC0003DMG400022225**	2925,3	571,33	449,33	2494	1451,3	2228,7	239,75	116,57	128,33	365,51	171,28	588,68
**PGSC0003DMG400022299**	902,67	452	316,67	573,33	170,67	86	27,3	48,5	47,43	101,87	42,25	10
**PGSC0003DMG400022426**	4354	997	379,67	3000	1529,3	1338	1086,4	38,2	143,12	795,76	153,44	96,25
**PGSC0003DMG400022779**	527,33	164	266,67	666,67	371,33	512	122,84	35,16	80,41	152,06	82,86	95,31

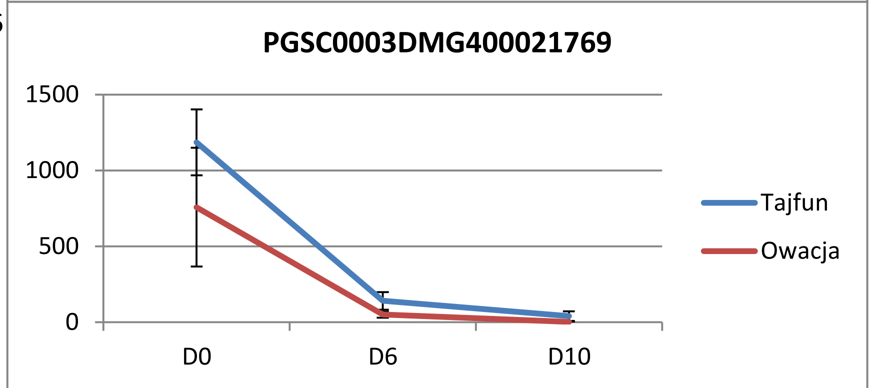

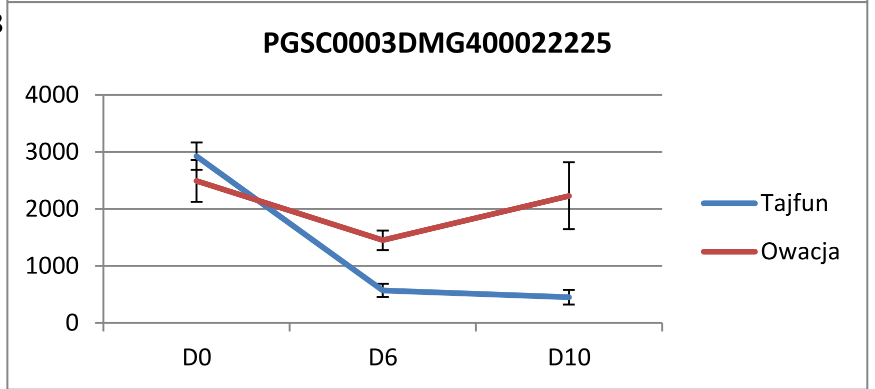

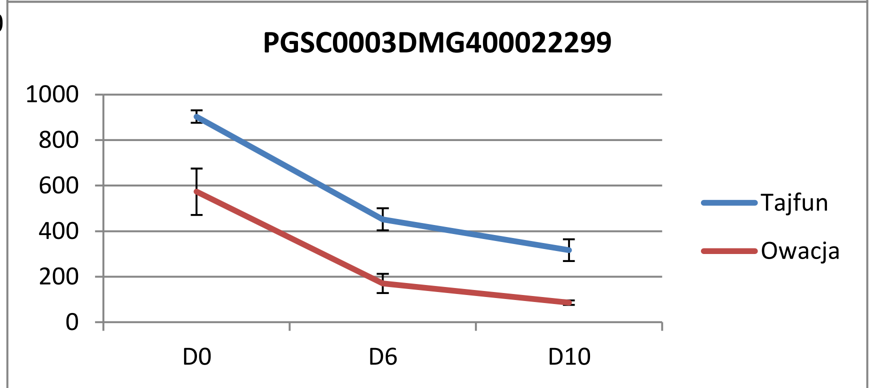

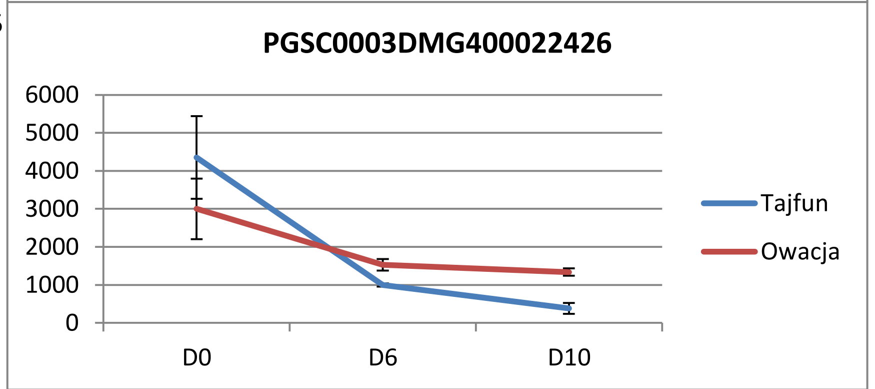

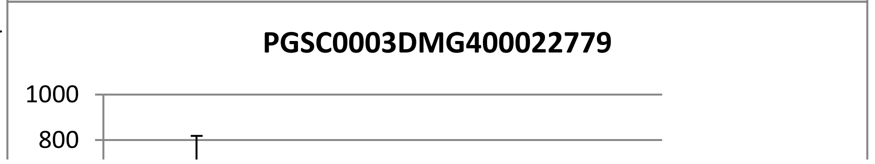

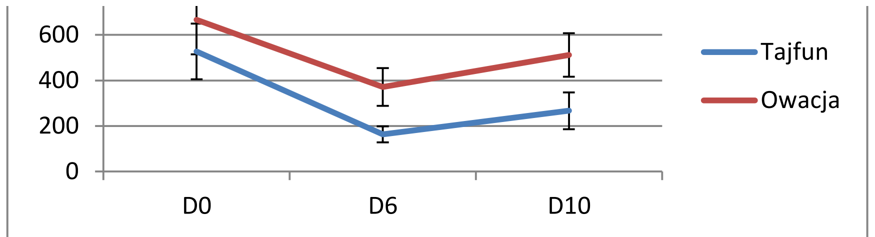

**PGSC0003DMG400023416**	15974	5423,3	2923,33	11355	3171,3	1131,7	1358,8	1286,9	253,74	2342,5	491,08	71,43
**PGSC0003DMG400023602**	450,67	201,33	218	476	400	1276	35	41,1	57,69	164,33	72,75	300,69
**PGSC0003DMG400024197**	8219,3	2921,3	4135,33	10328	4449,3	8762,7	1667,2	155,31	612,02	288,81	1177,75	1553,9
**PGSC0003DMG400024633**	7078,7	3326	2319,33	4911,3	2326	1174,7	1560,3	511,89	634,11	669,13	190,34	83,86
**PGSC0003DMG400024656**	266,67	152	160,67	163,33	84	70,67	49,41	37,36	11,02	56,08	31,75	30,09

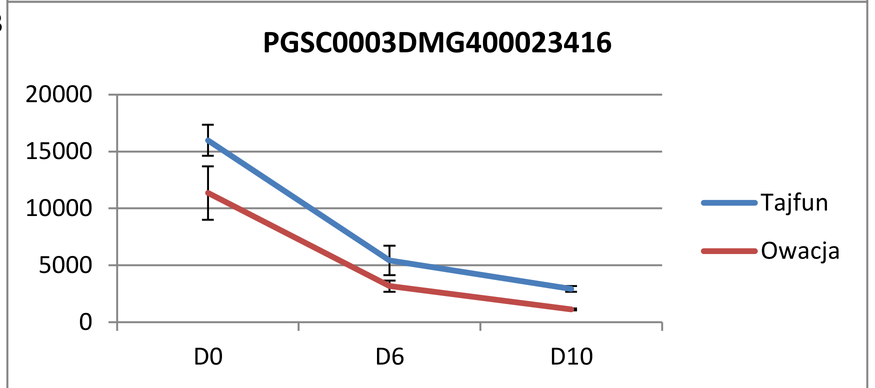

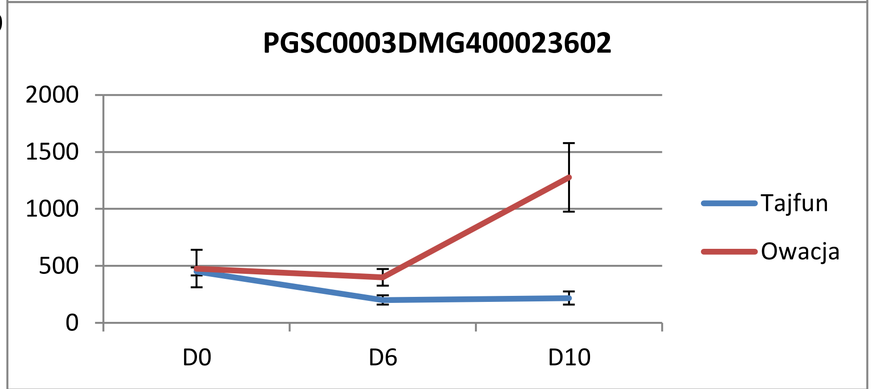

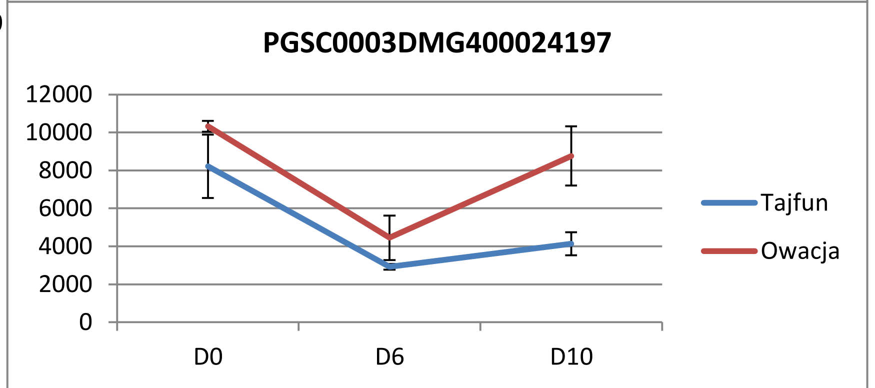

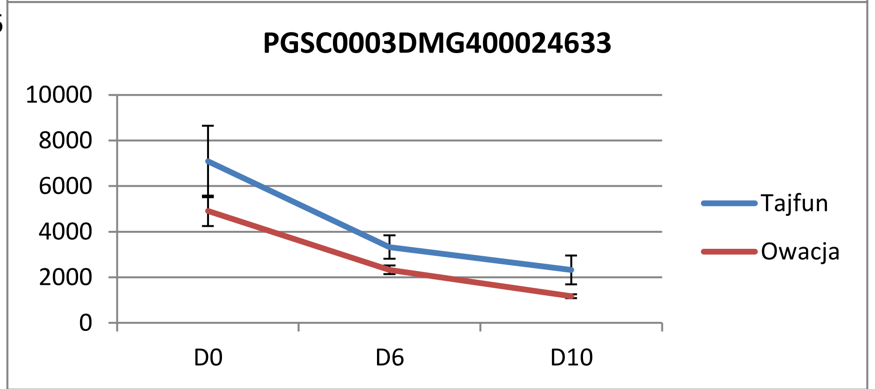

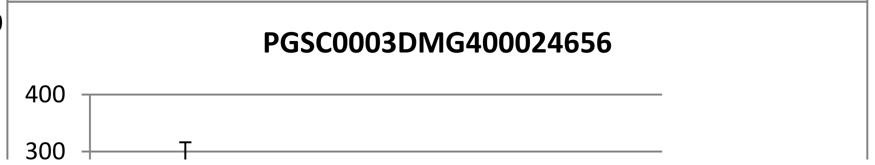

**PGSC0003DMG400025007**	792	301,33	243,33	646,67	174,67	80	168,3	90,78	18,58	107,93	31,39	13,86
**PGSC0003DMG400025102**	2190,7	992	573,33	1908,7	2053,3	1007,3	534,03	374,64	75,75	601,08	513	274,81
**PGSC0003DMG400025814**	1354,7	708,67	603,33	1058,7	1234,7	1070	189,74	106,59	388,72	146,84	401,82	57,24
**PGSC0003DMG400025862**	2610,7	778,67	700	3157,3	1582,7	2101,3	945,11	171,08	352,12	920,32	218,91	90,69
**PGSC0003DMG400026106**	434	291,33	254,67	299,33	164,67	118,67	135,1	81,22	98,68	56,9	51	54,45

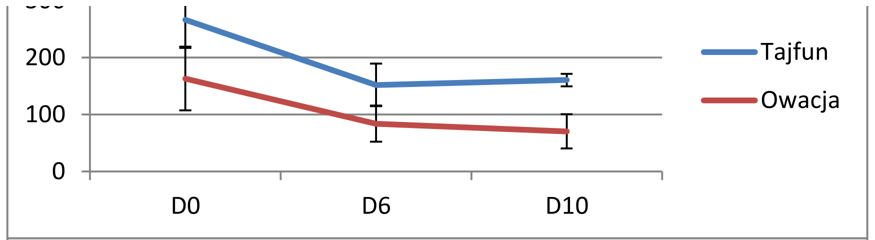

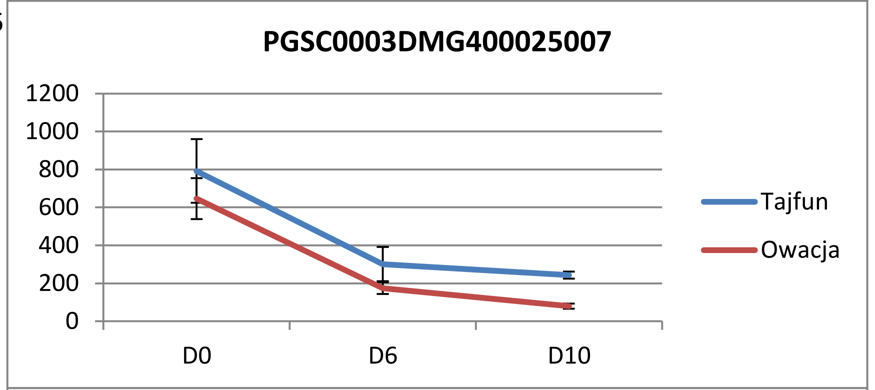

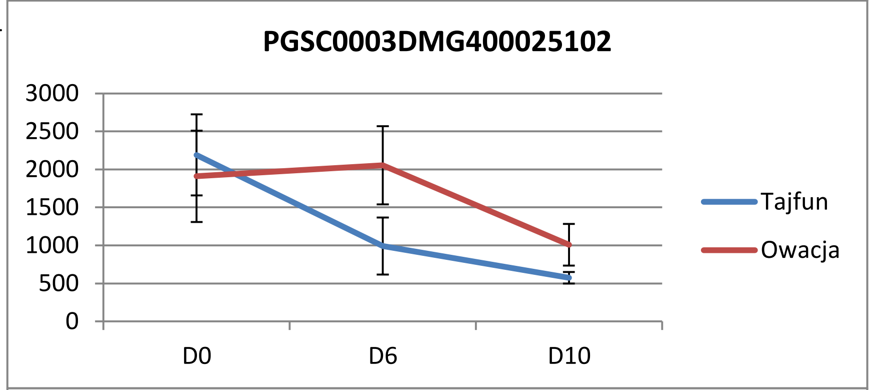

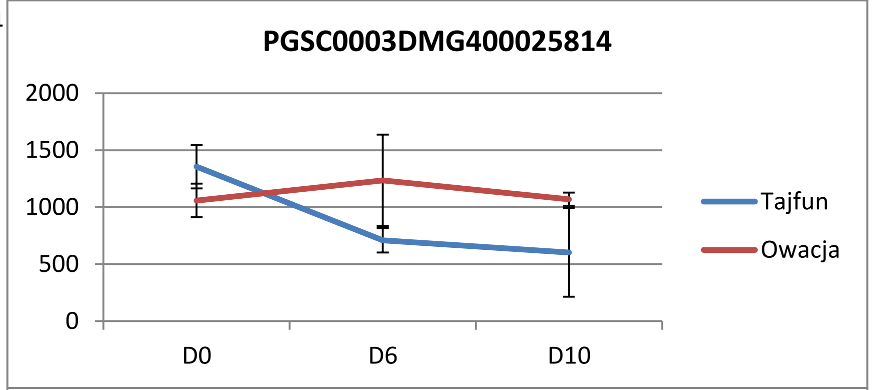

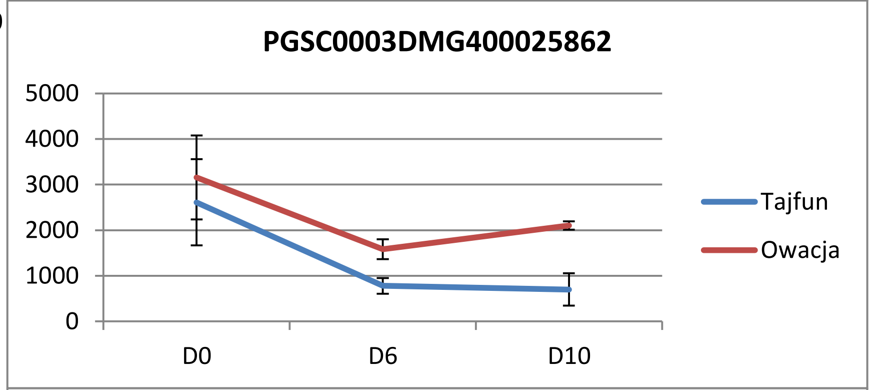

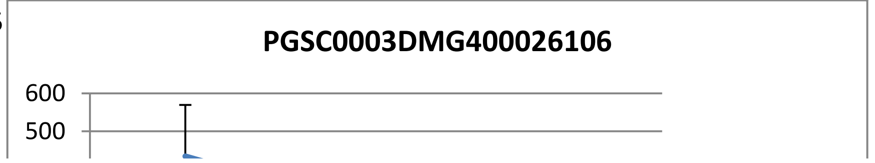

**PGSC0003DMG400026199**	3797	2465	2167,33	3494	3506	2993,7	515,15	149,89	882,54	1967,4	279,16	238,43
**PGSC0003DMG400026446**	964,67	291,33	236	744,67	119,33	440,67	86,31	11,02	74,32	164,1	61,33	111,93
**PGSC0003DMG400026472**	3708	378,67	260,67	4674,7	1152,7	893,33	724,39	107,82	87,69	1429,1	257,42	208,71
**PGSC0003DMG400026516**	824	395,33	135,33	784	134	54	303,13	85,19	50,65	150,57	56,71	29,6
**PGSC0003DMG400027156**	6934	3035,3	2380	7788,7	4105,3	4701,3	1182,2	549,04	202,26	2424,1	446,52	442,73

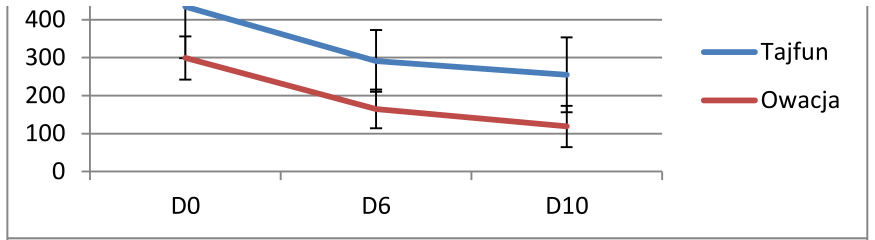

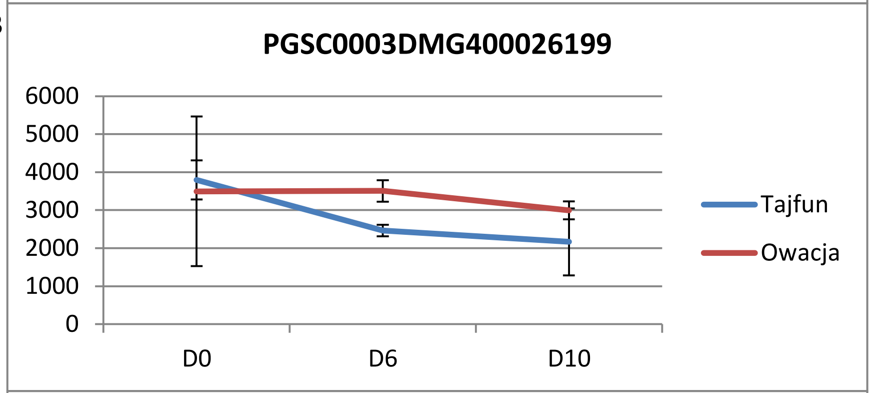

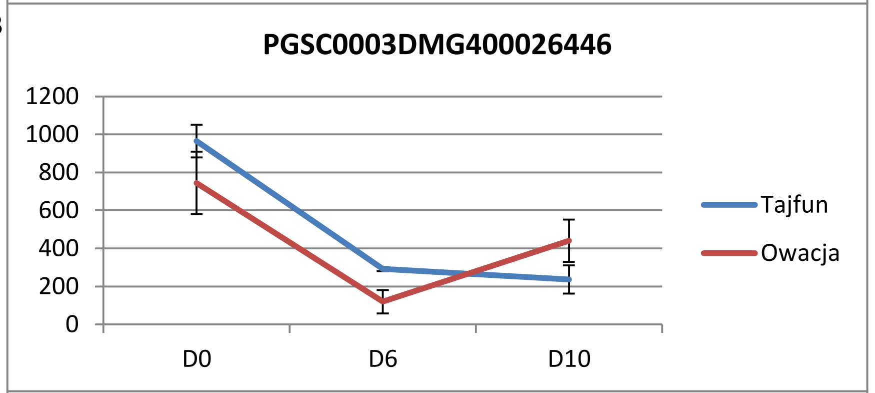

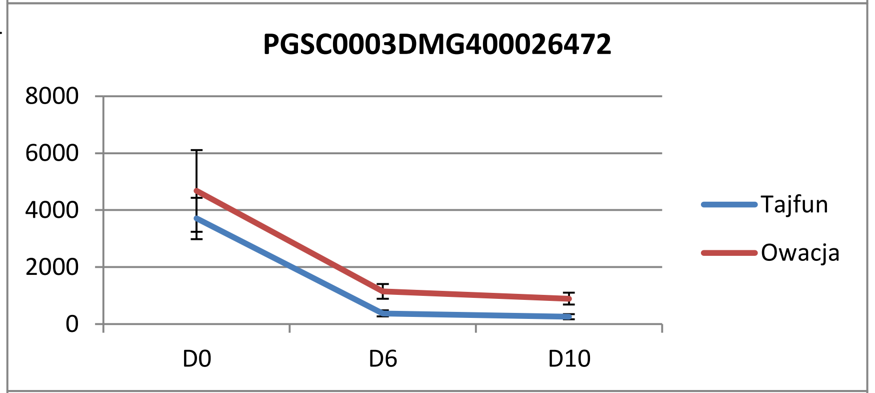

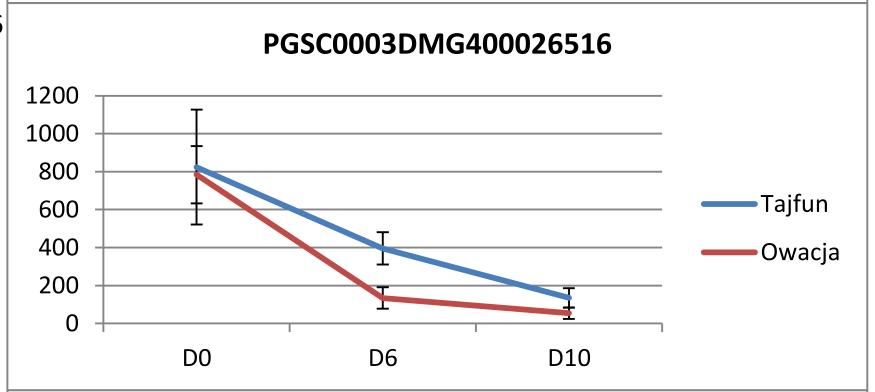

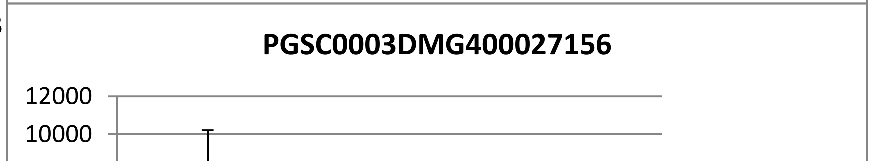

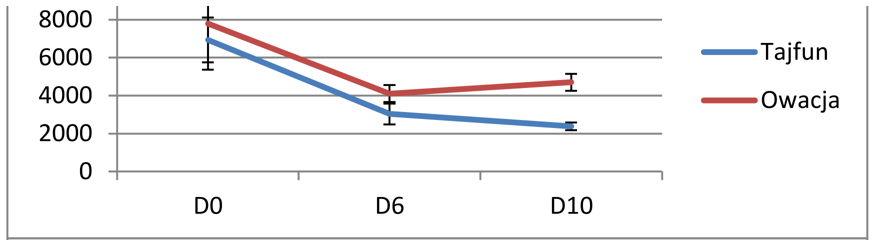

**PGSC0003DMG400027746**	14301	7722	3948	12280	5451,3	2234	2984,9	784,7	883,49	2566,2	200,25	356,34
**PGSC0003DMG400027951**	517,67	342	336,67	380,33	214,67	105	5,51	86,81	80	80,58	56,15	11,79
**PGSC0003DMG400028137**	2912,7	935,33	277,33	1762,7	454	118	689,9	236,48	79,25	825,9	140,47	21,07
**PGSC0003DMG400028164**	652,67	178	184,67	402	311,33	1273,3	279,84	64,47	122,15	210,63	37,43	287,33
**PGSC0003DMG400028288**	2459,3	1282	1709,33	3303,3	2045,3	3913,3	240,45	142,51	745,92	821,38	235,37	174,73

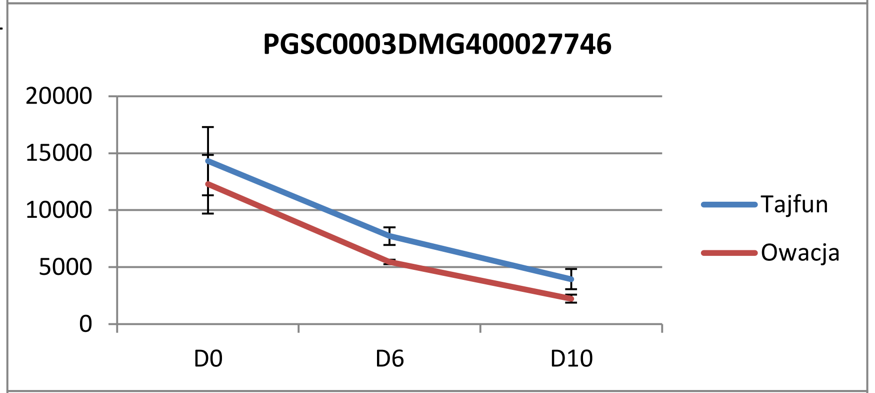

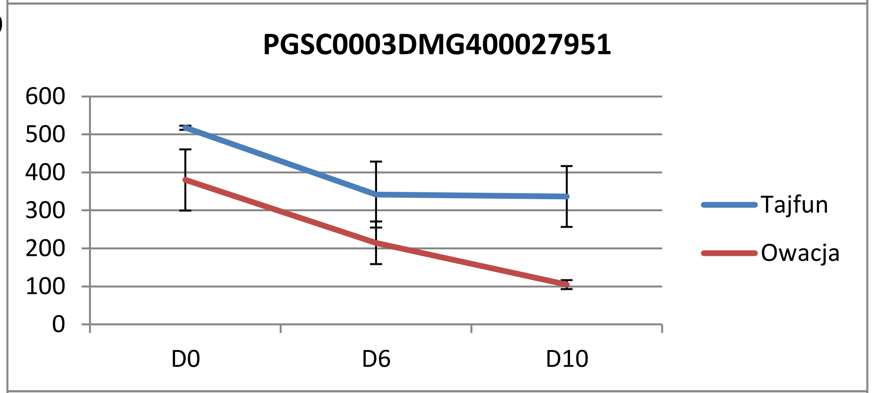

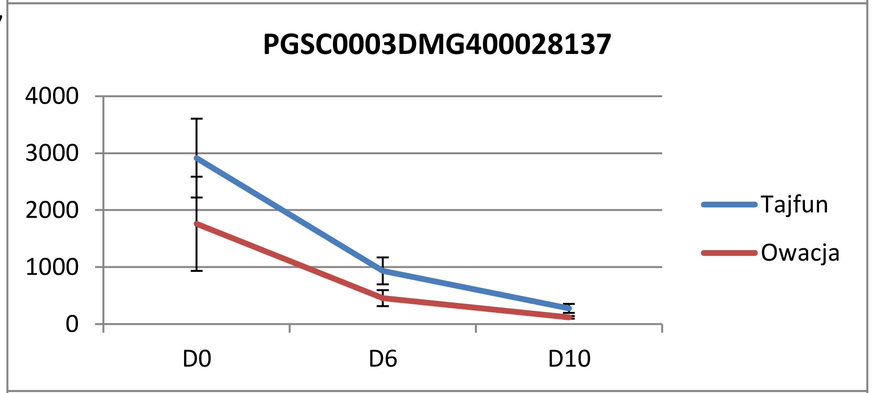

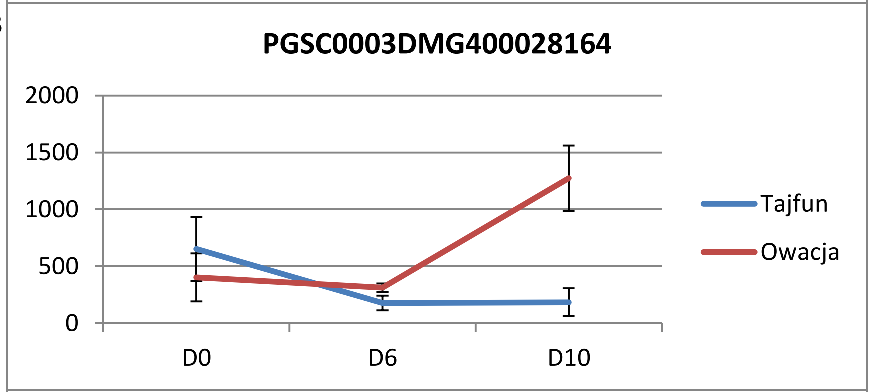

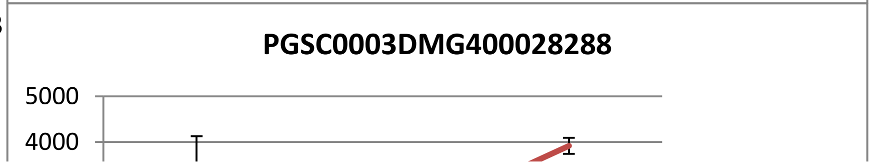

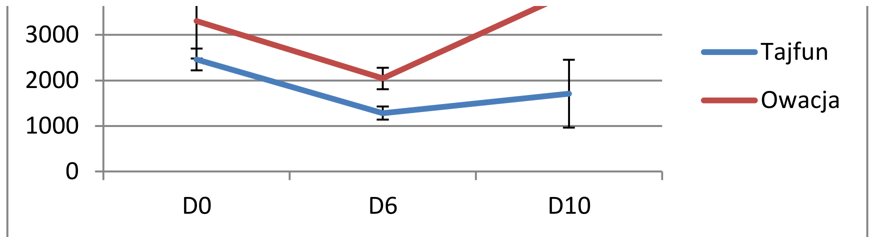

**PGSC0003DMG400028363**	5805,3	3539,3	2745,33	4611,3	2566	1477,3	2027,5	267,33	30,02	839,61	473,32	234,95
**PGSC0003DMG400028977**	2700	1067,3	1527,33	2422	1646,7	3530,7	100,54	151,93	283,25	516,21	511,73	352,87
**PGSC0003DMG400029086**	18289	7368	1968	22286	4014	6670,7	4931	1841,4	930,63	4334,7	2172,97	812,88
**PGSC0003DMG400029294**	963,33	692	676	486	370,67	387,33	29,14	159,96	110,11	439,06	169,4	53
**PGSC0003DMG400029355**	1458,7	945,33	765,33	1653,3	558,67	325,33	294,08	196,17	46,19	388,98	102,1	21,01

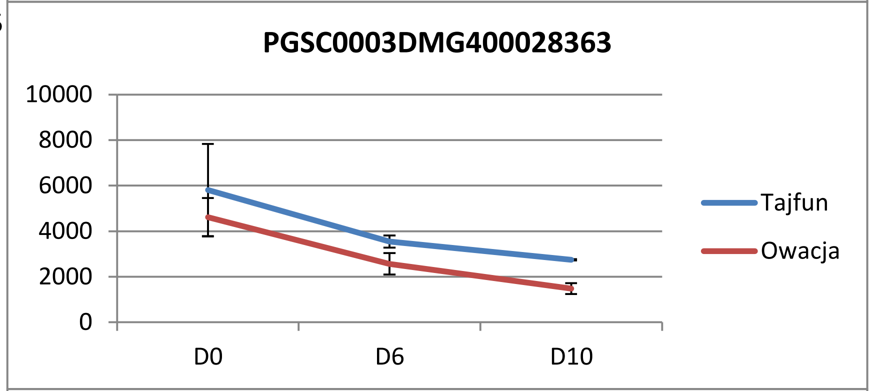

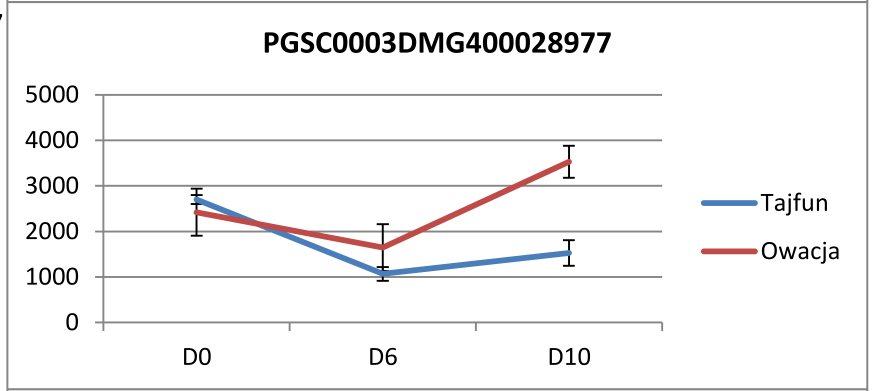

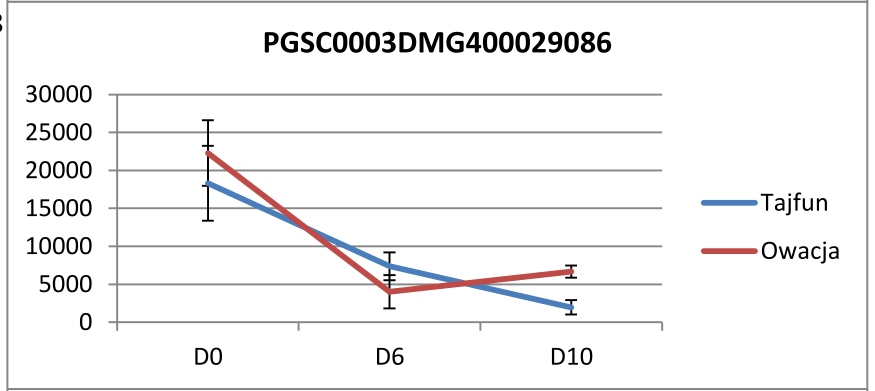

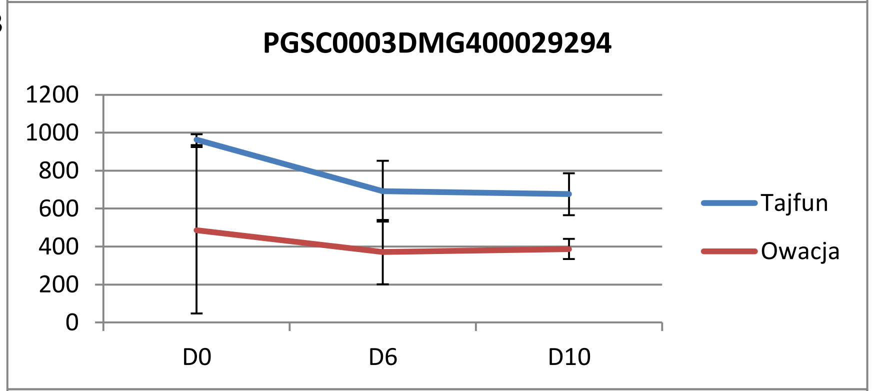

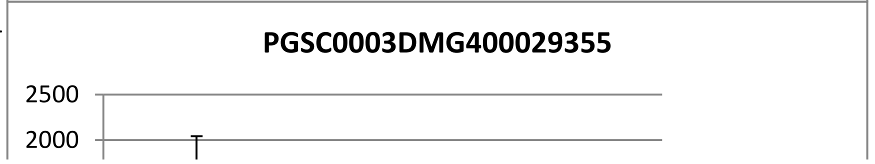

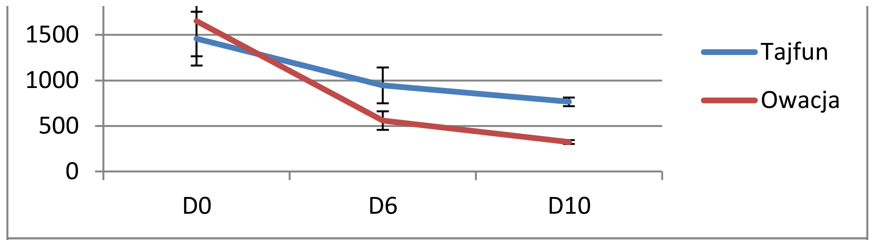

**PGSC0003DMG400029373**	515,33	198,67	54	532,67	108	122,67	147,31	18,15	49,76	76,79	24,33	38,44
**PGSC0003DMG400029505**	185,33	47,33	48,67	243,33	156,67	93,33	43,14	22,12	27,23	73,82	8,08	17,01
**PGSC0003DMG400029570**	297,33	79,33	33,33	444	166,67	240,67	68,42	9,87	31,9	169,72	9,02	51,59
**PGSC0003DMG400029693**	5336,7	2794,7	2224,67	5537,3	4038	5338,7	1433,4	128,02	377,42	1190,7	444,01	415,52
**PGSC0003DMG400029803**	573,33	360	320	746,67	1210	526	172,7	90,35	32,19	72,4	18,33	155,58

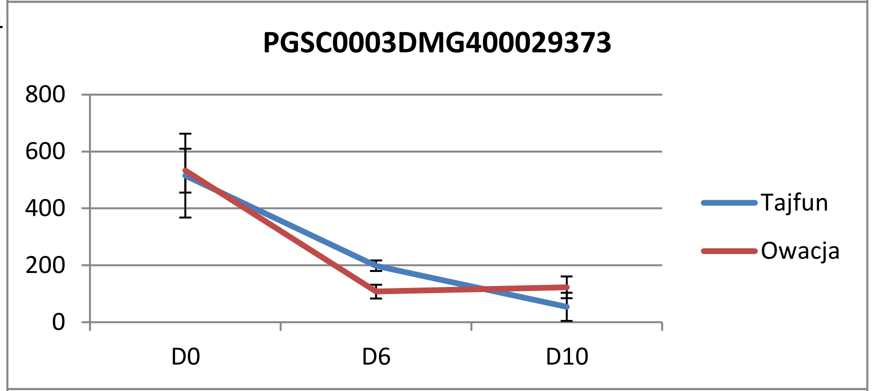

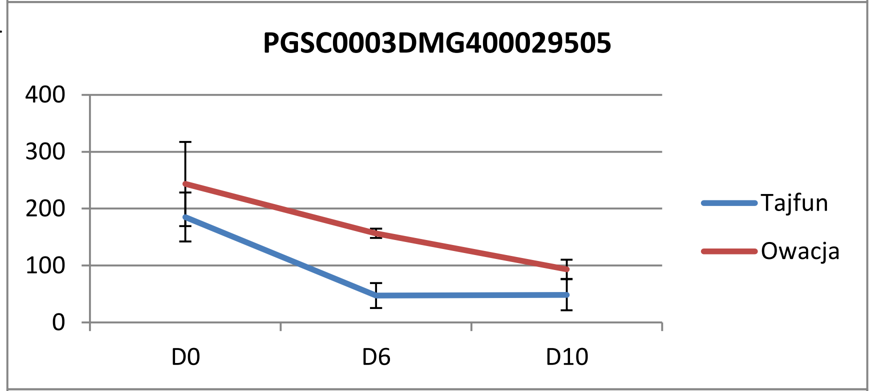

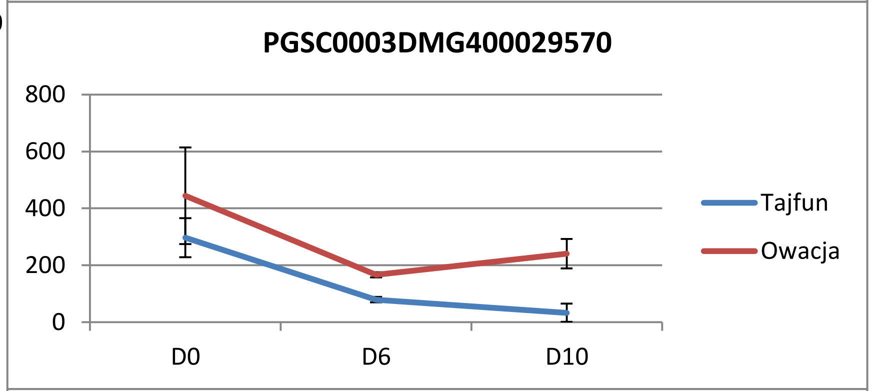

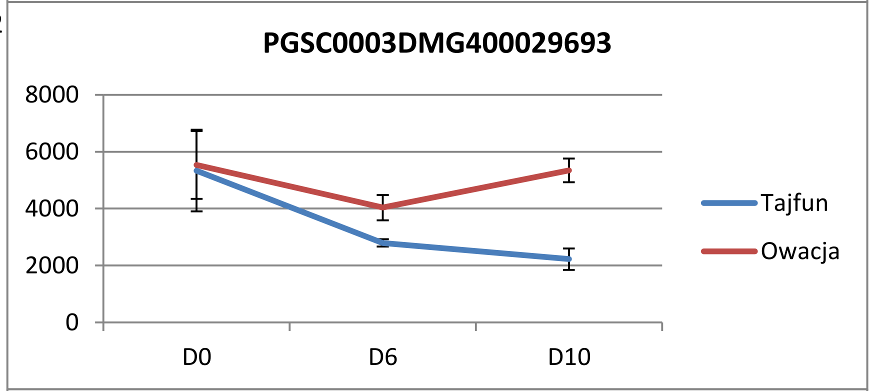

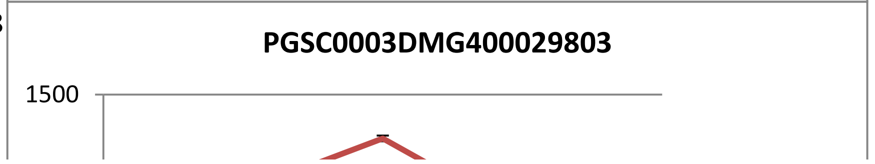

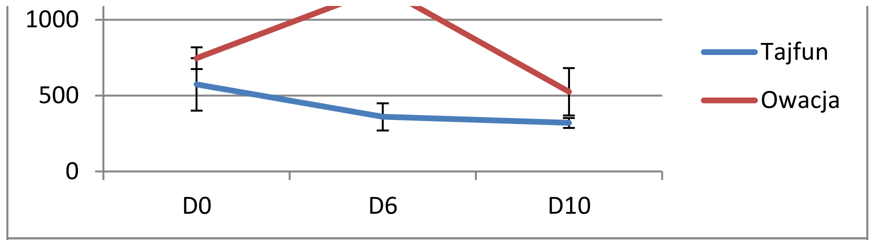

**PGSC0003DMG400030220**	1151,3	548	301,33	1492,7	840,67	542,67	277,95	48,87	44,24	387,27	105,84	87,23
**PGSC0003DMG400030333**	538,67	308	294	622,67	510,67	424,67	202,15	86,28	19,7	106,01	77,67	22,03
**PGSC0003DMG400030359**	53755	15348	8018	51229	20885	19900	14144	3457,6	705,06	8777,2	2001,54	2115,6
**PGSC0003DMG400030391**	687,33	219,33	44	664,67	344,67	126	73,33	51,94	3,46	50,21	22,74	46,13
**PGSC0003DMG400030431**	704,67	331,33	250	980	501,33	604,67	191,84	54,45	17,44	240,76	7,57	108,1

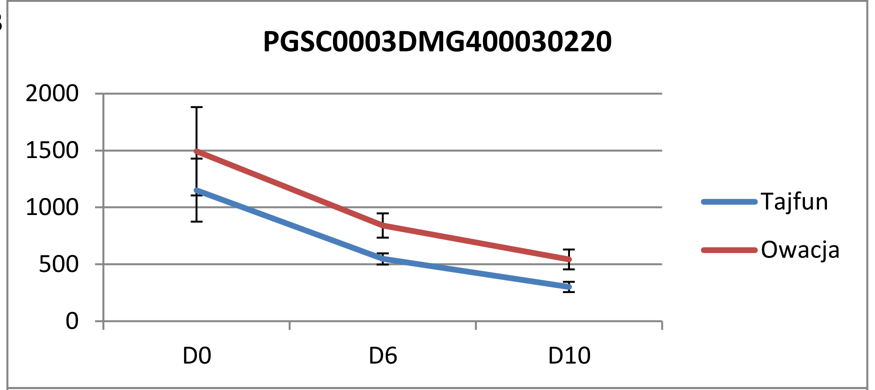

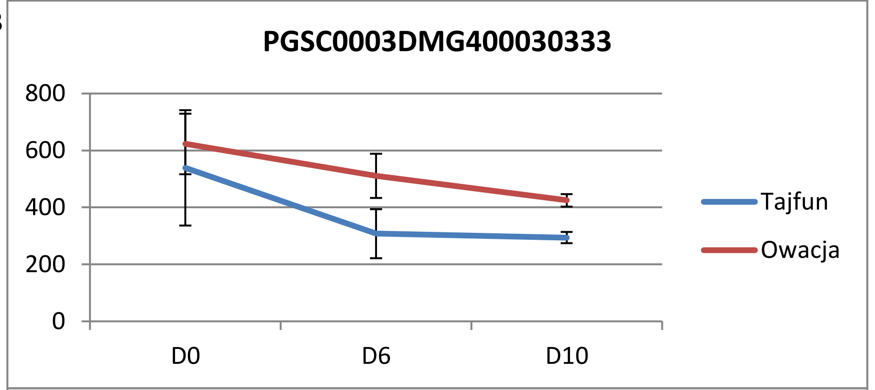

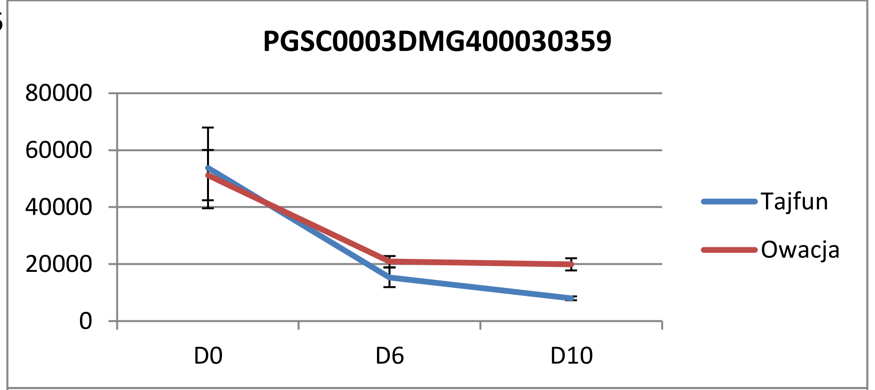

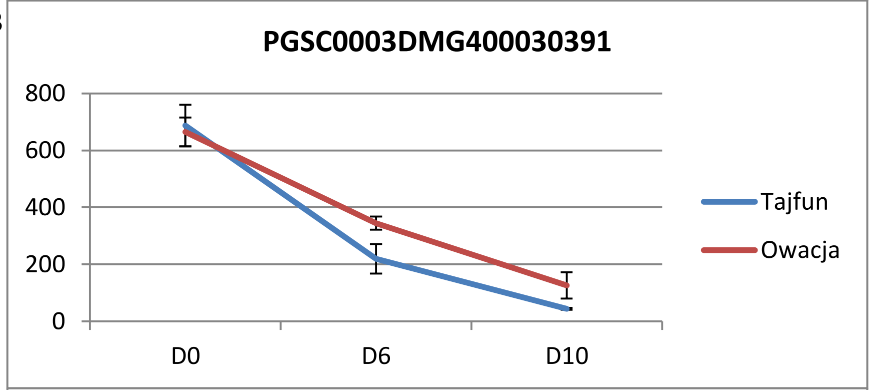

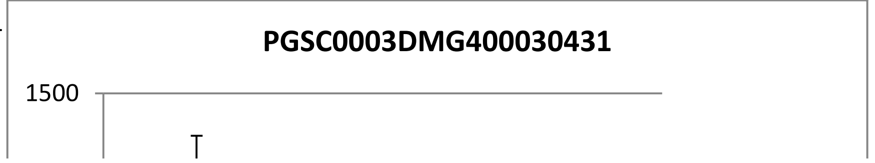

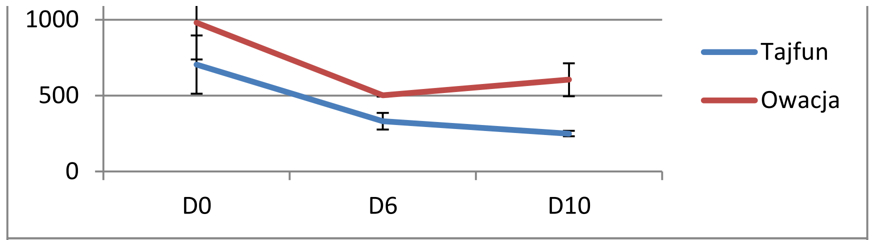

**PGSC0003DMG400030694**	782,67	350,67	330,67	945,33	840,67	503,33	110,01	33,55	187,15	234,65	47,72	68,77
**PGSC0003DMG400030731**	1150	0	220,67	464	16,67	5,33	552,04	0	186,43	230,99	17,01	4,62
**PGSC0003DMG400030905**	424	82,67	35,33	322	204,67	159,33	109,56	18,04	33,01	95,02	16,17	57,87
**PGSC0003DMG400030995**	529,33	83,33	110,67	501,33	224	278,67	180,71	32,15	86,12	225,76	60,63	52,78
**PGSC0003DMG400031031**	642,67	256	162,67	475,33	73,33	62,67	140,43	13,11	19,01	44,29	22,3	11,37

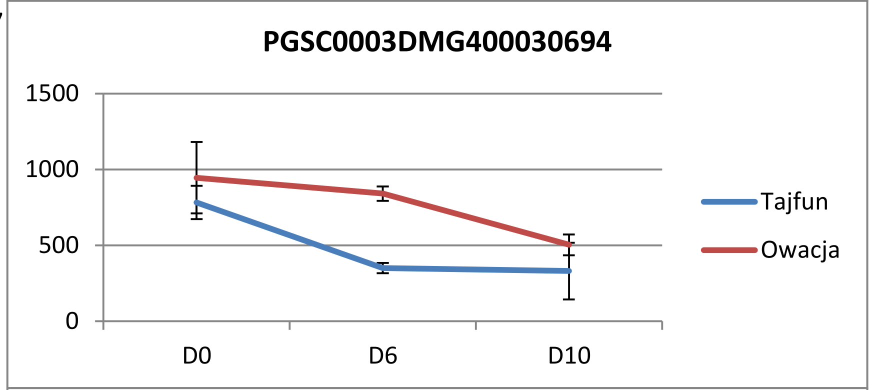

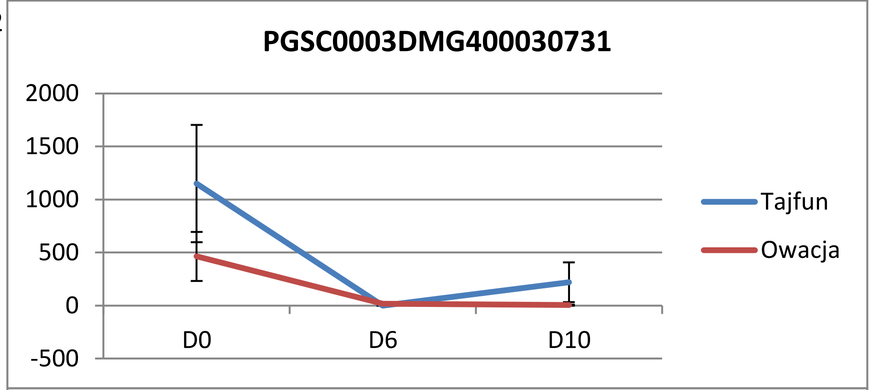

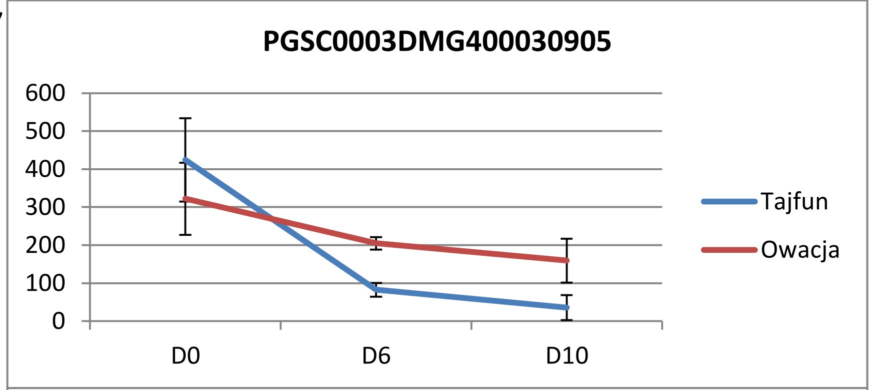

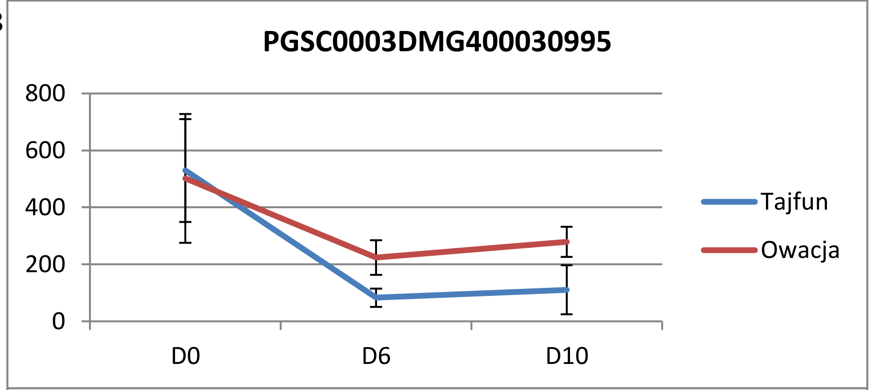

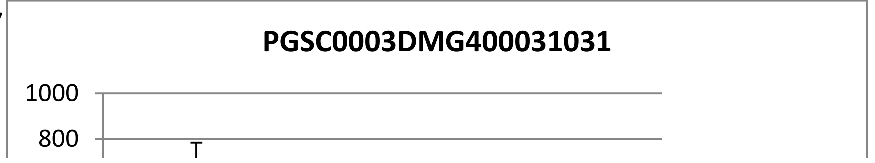

**PGSC0003DMG400031079**	359,33	47,33	18	358,67	128,67	140	127,32	20,23	8,72	172,62	60,01	33,05
**PGSC0003DMG400031081**	5654	3916	2504,67	4730,7	5284,7	7142,7	1010	353,41	1597,18	1531	396,08	1716,4
**PGSC0003DMG400031248**	1913,3	1178	844,67	2138,7	2204	1678	555,37	422,79	244,82	1244,8	508,41	248,14
**PGSC0003DMG400031262**	1126	420	362	906	628	1129,3	321,04	116,57	12,17	244,1	68,79	71,06
**PGSC0003DMG400031842**	965,33	36,67	26,67	876,67	98,67	265,33	335,57	19,73	20,43	424,83	11,72	89,11

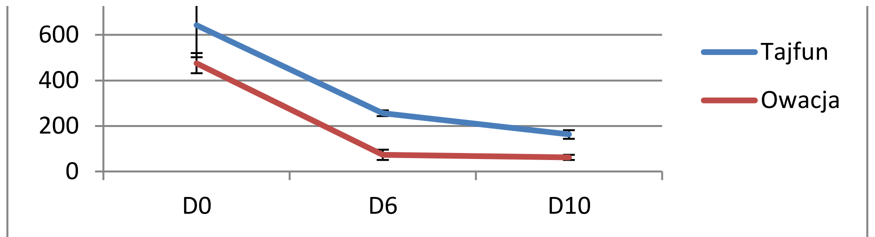

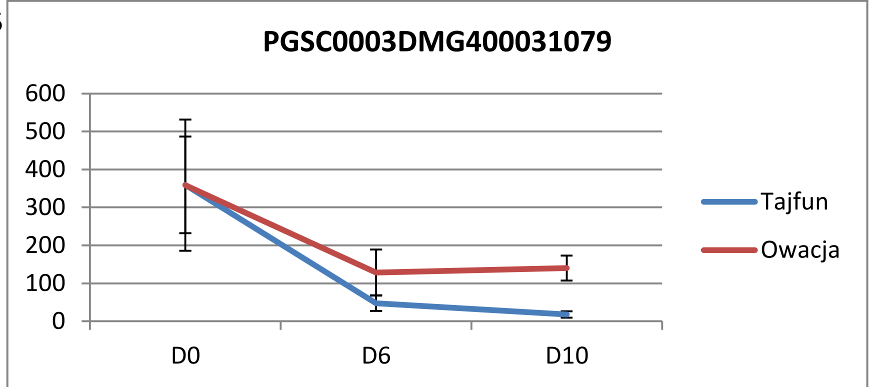

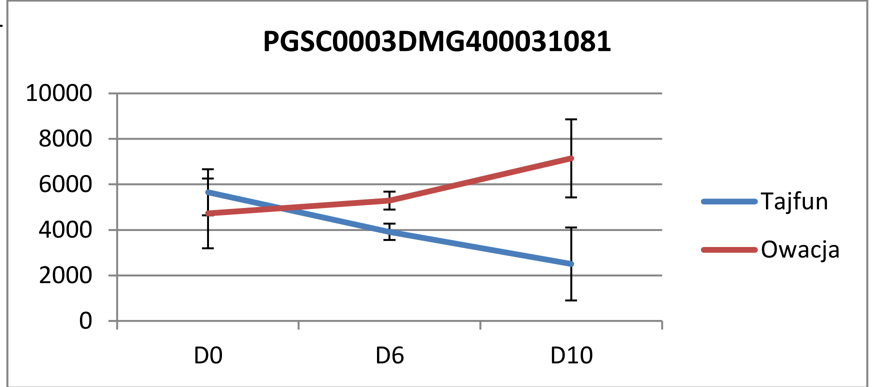

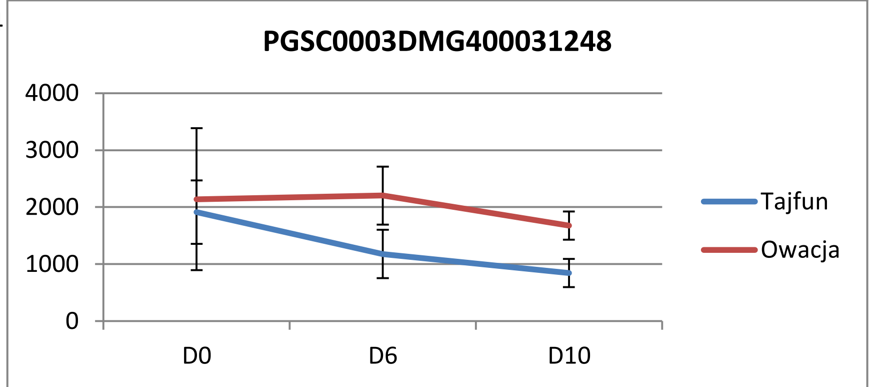

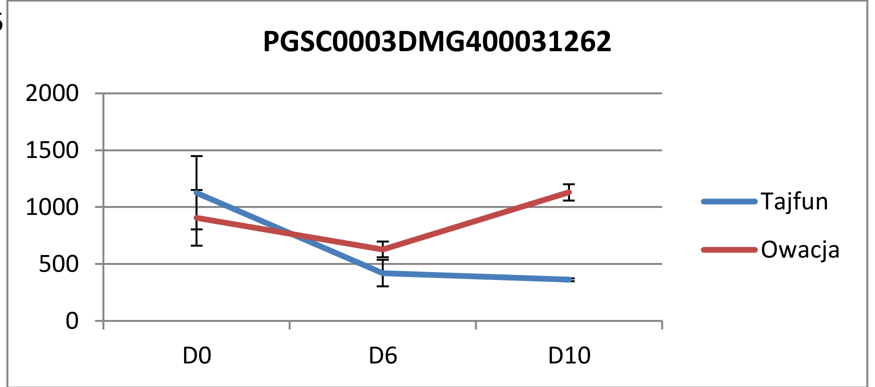

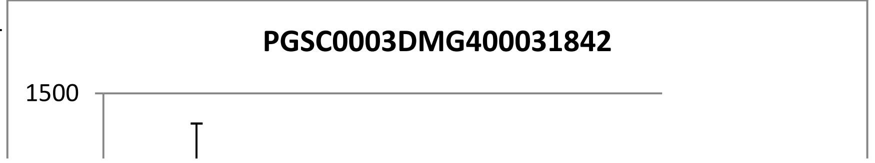

**PGSC0003DMG400032537**	496	167,33	204	484	576,67	330,67	84,29	22,74	101,49	121,01	91,57	97,99
**PGSC0003DMG400033099**	5564,7	582	244	9119,3	934	1136,7	2359,9	35,16	30	3207,3	122,57	142,47
**PGSC0003DMG400035823**	724,67	490,67	506,67	836,67	892	1130,7	95,71	63,13	152,45	57,77	76,24	69,12
**PGSC0003DMG400044958**	4122,7	3594,7	2268	3640,7	2628	1328,7	1164,6	328,94	251,15	1326,2	259,1	216,3
**PGSC0003DMG401004500**	206	40	36,67	515,33	127,33	158,67	66	7,21	23,44	321,56	20,53	42,72

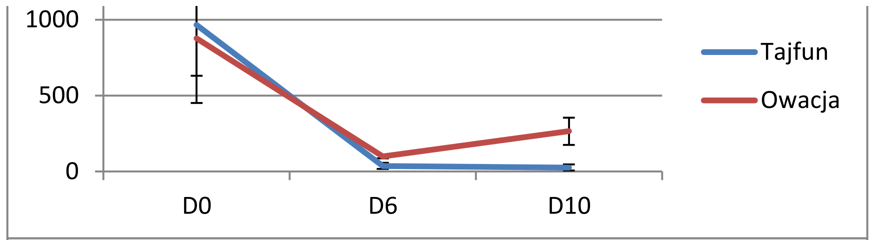

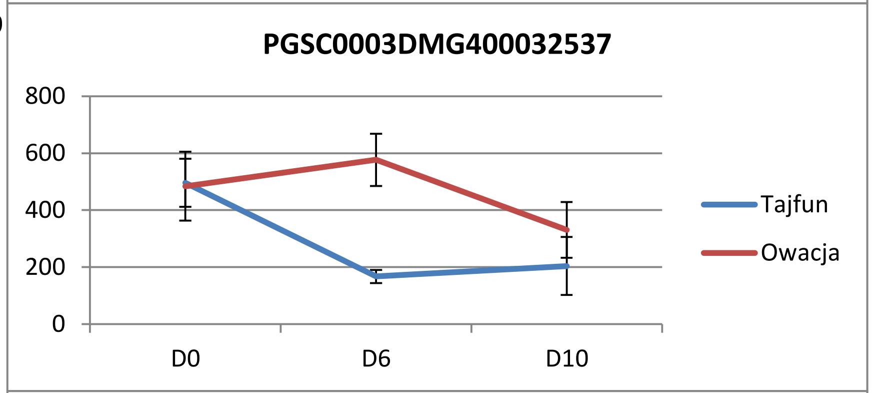

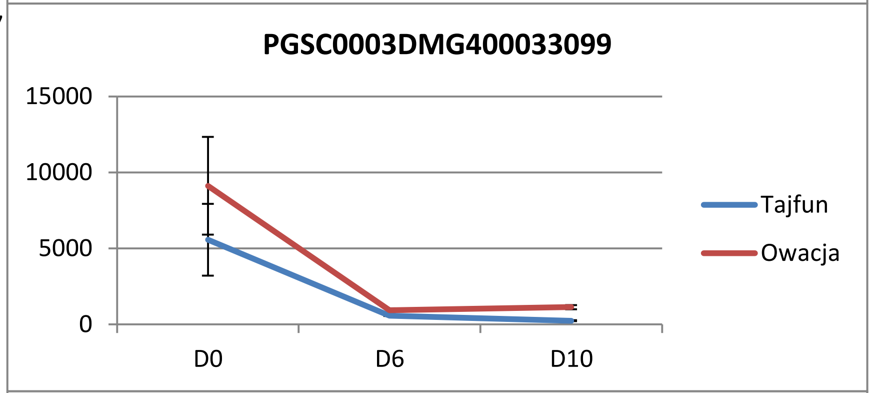

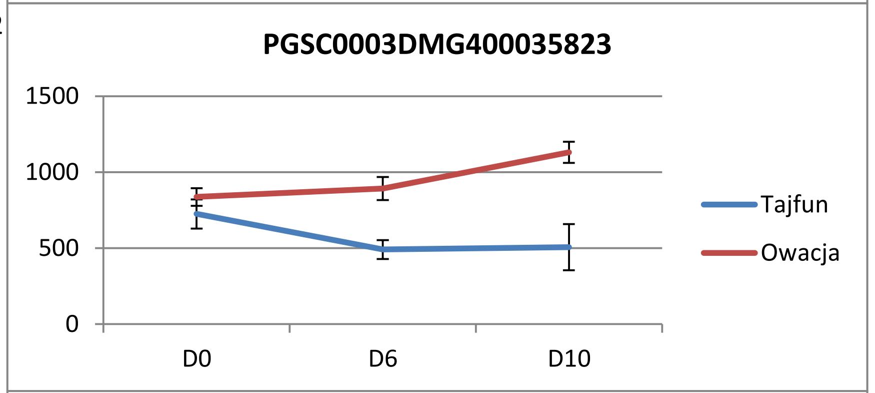

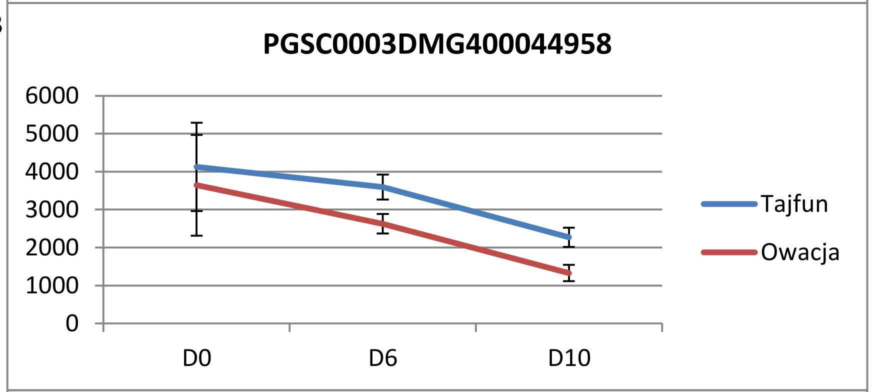

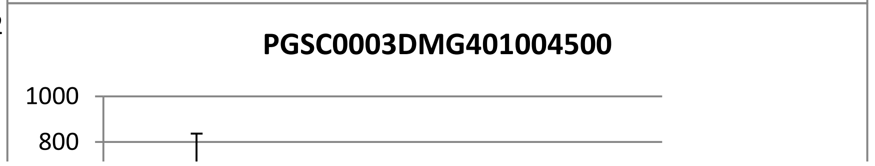

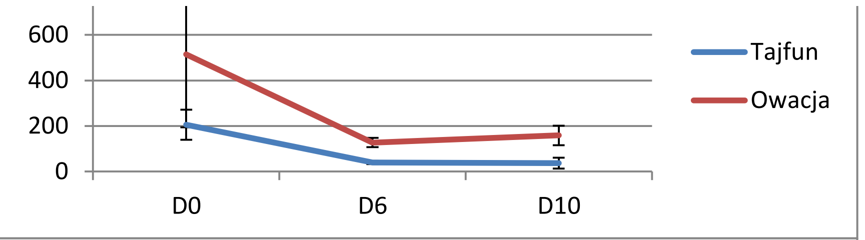

**PGSC0003DMG401004637**	1576,7	686	464	1014,7	990	1024,7	123,16	108,06	32	332,42	129,68	104,89
**PGSC0003DMG401004779**	7029	2604	3026	4885,7	1363,7	789,33	1947,6	297,64	386,58	1433,6	313,82	84,25
**PGSC0003DMG401012244**	3811,3	1635,3	1282,67	2551,3	3564	3741,3	565,11	224,02	491,39	964,83	533,52	550,93
**PGSC0003DMG401013418**	7357,3	2550	1446,67	4409,3	519,33	335,33	1685,1	648,11	753,01	1816,5	125,23	118,5
**PGSC0003DMG401015682**	139,33	56	52,67	86	180	170	63,76	15,1	17,24	51,42	70,31	60,4

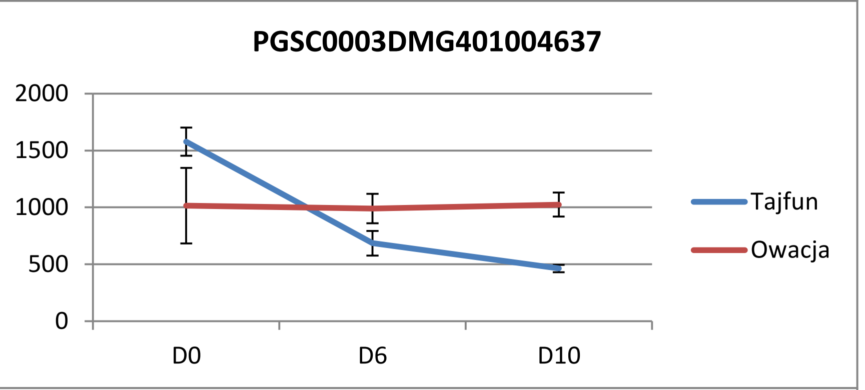

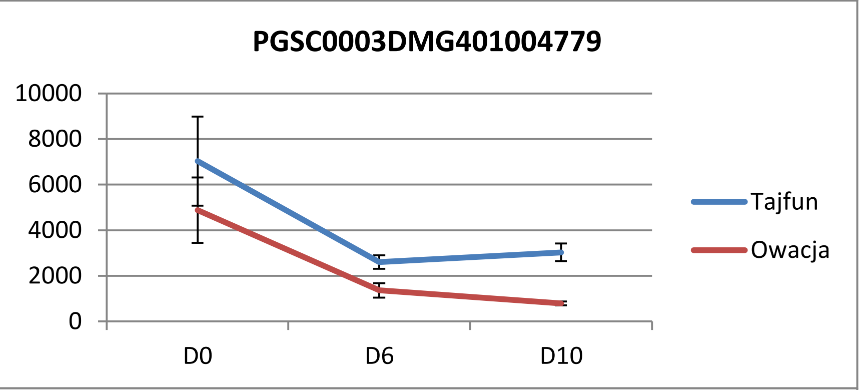

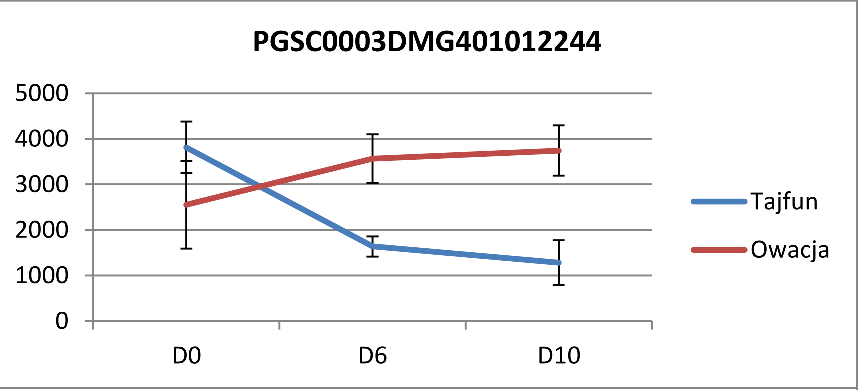

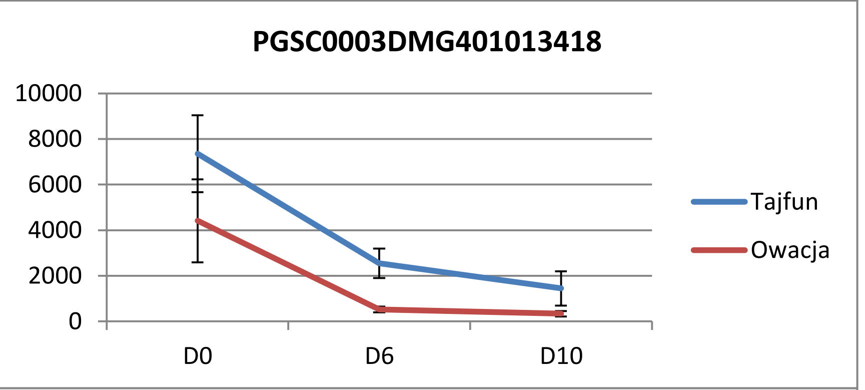

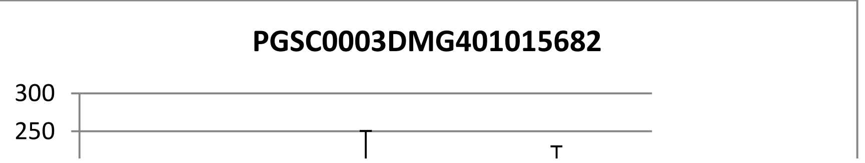

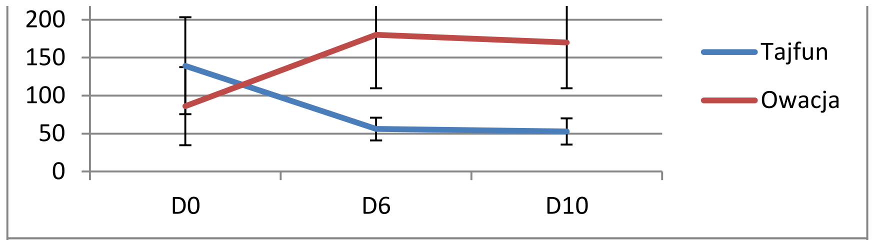

**PGSC0003DMG401023562**	1986,7	770	298,67	2554,7	409,33	147,33	44,47	290,17	30,09	615,31	134,08	20,03
**PGSC0003DMG401025150**	13500	2174	1789,33	24122	4286	3100	5113,5	708,38	636,1	7768,3	1448,84	296,18
**PGSC0003DMG401025831**	61147	13664	7527,33	54346	9376	1096,7	25250	2422,7	4683,62	12574	2480,17	1899,5
**PGSC0003DMG401026043**	136	67,33	37,33	169,33	142,67	77,33	14	21,57	14,47	45,88	58,39	11,55
**PGSC0003DMG401031741**	1448	917,33	419,33	1002,7	1260,7	174,67	333,15	235,84	108,71	497,78	105,31	33,61

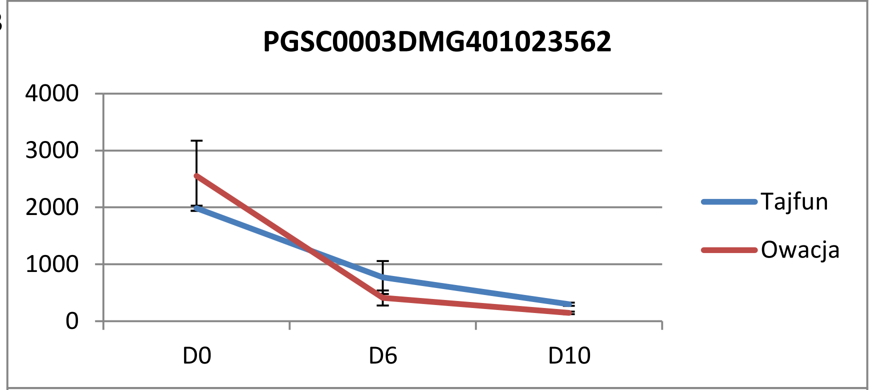

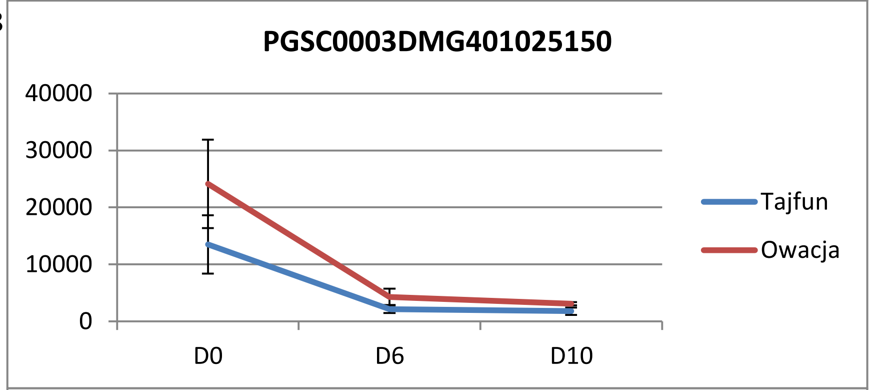

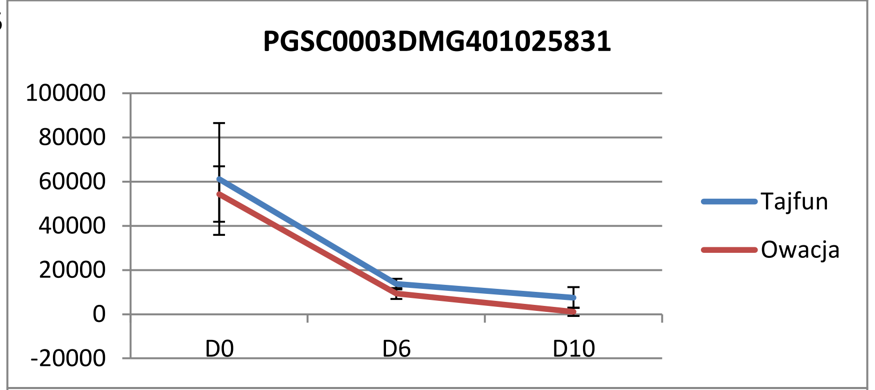

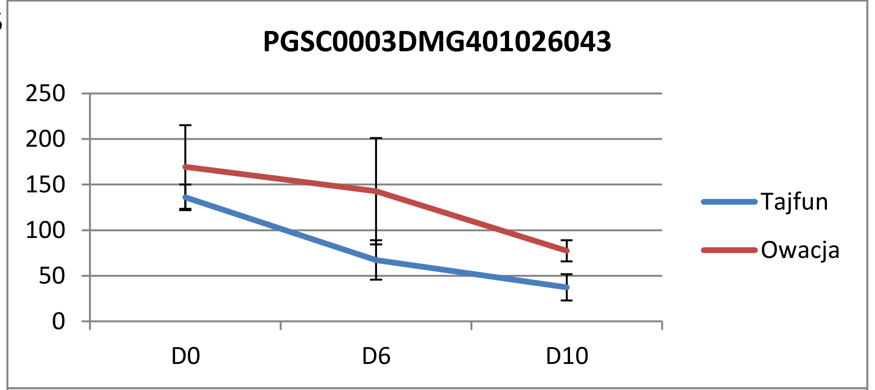

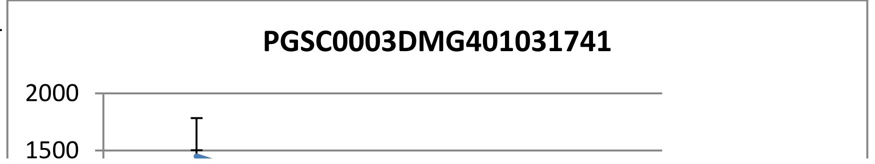

**PGSC0003DMG402002542**	638,67	510,67	471,33	774,67	736	819,33	34,02	86,63	55,22	89,94	63,02	154,61
**PGSC0003DMG402003286**	14164	6425	1692	9309,7	3638	408,67	1349,7	1024,4	461,18	3067,4	437,73	62,32
**PGSC0003DMG402004120**	1330,7	823	676,67	1429,7	1444	1429,7	270,94	118,9	126,26	251,95	136,57	138,44
**PGSC0003DMG402010056**	26340	10164	7093,33	20765	6882,7	2424,7	4375,5	1920,2	2721,07	1443,7	1127,27	117,41
**PGSC0003DMG402014796**	4173,3	3110,7	2778	5358,7	4480,7	4166,7	261,6	611,23	1064,16	467,92	440,08	277,45

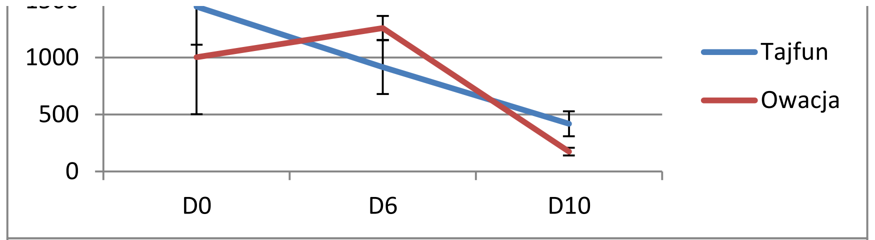

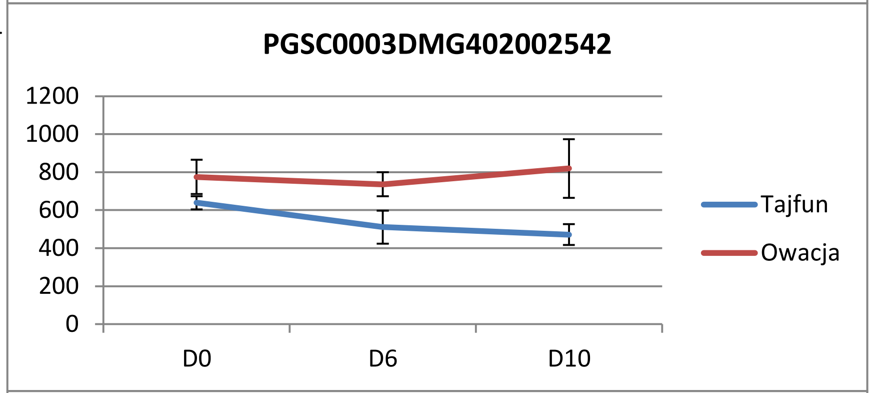

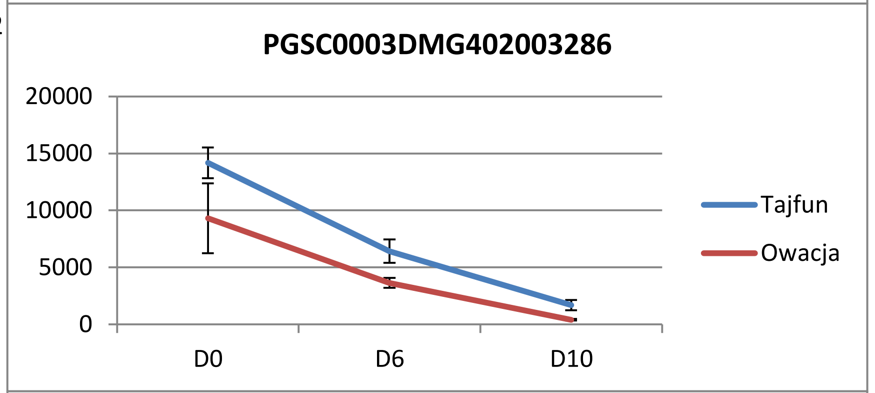

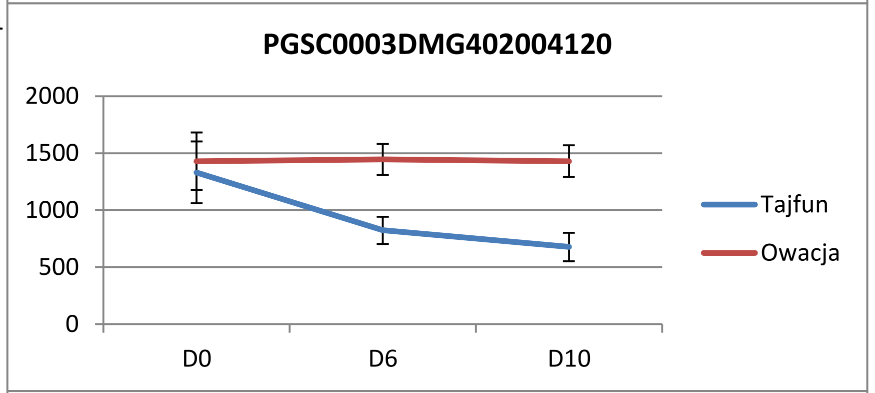

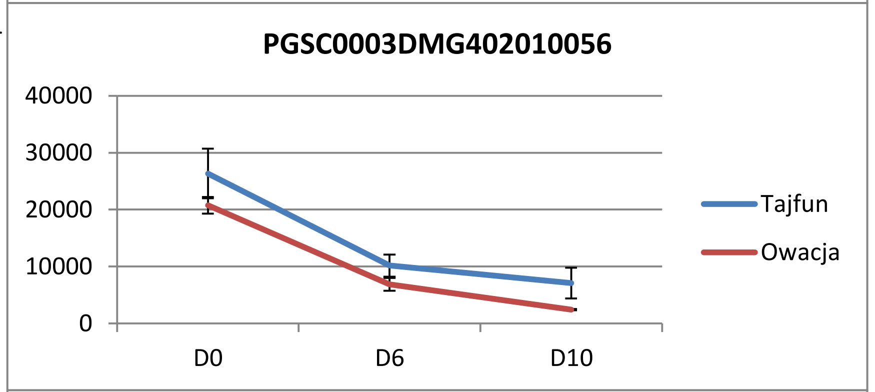

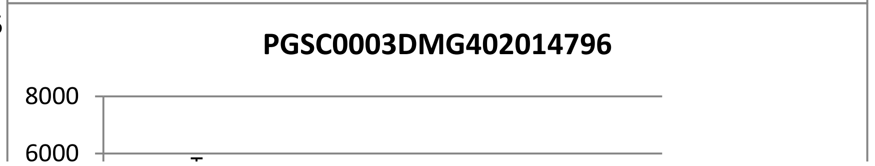

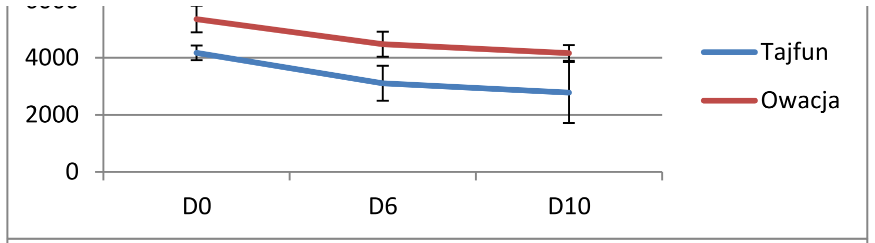

**PGSC0003DMG402015926**	3180	1423,3	525,33	2560	1962	954	605,28	177,86	216,82	697,11	133,1	122
**PGSC0003DMG402019151**	6645,3	3599,3	2640	5761,3	2338	1534	1109,7	598,14	210,27	243,49	687,24	52
**PGSC0003DMG402019982**	187,67	54,67	60,67	192,67	112	551,67	69,64	11,37	7,57	128,52	17,44	48,44
**PGSC0003DMG402030822**	2927,3	1398	2244	3022	2047,3	4882,7	398,56	98,65	417,43	522,41	79,43	566,47

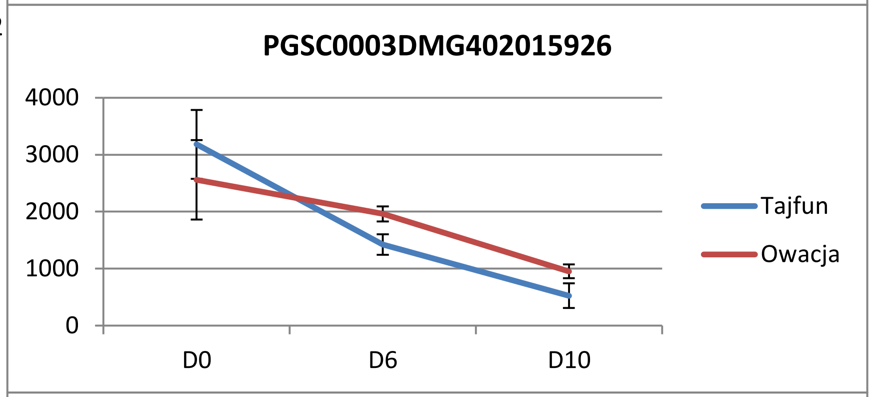

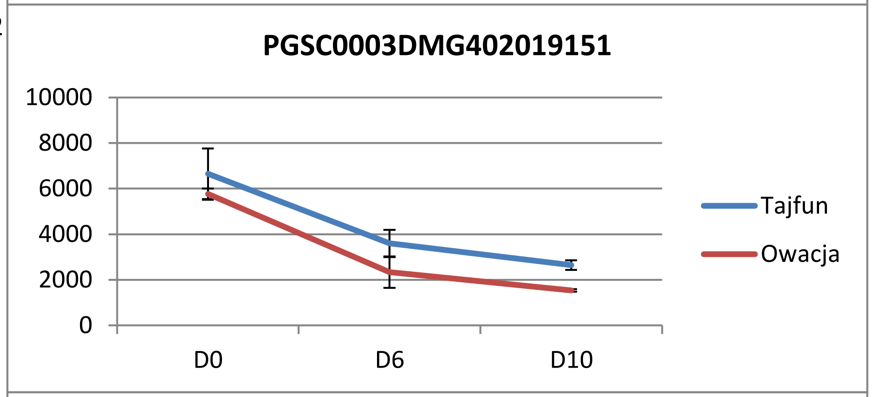

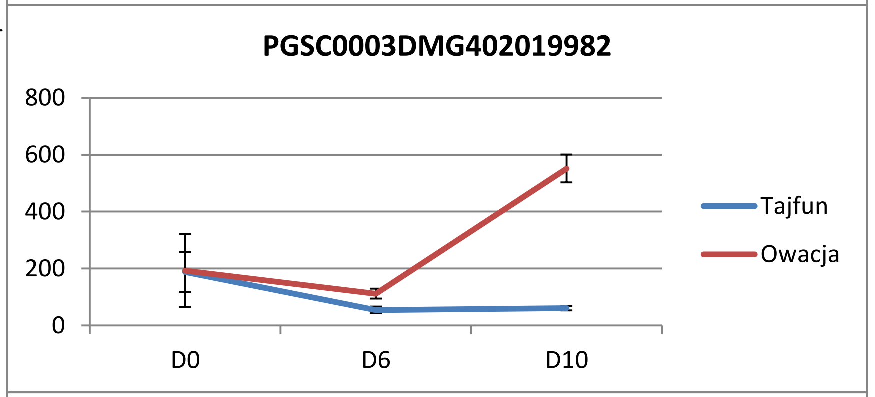

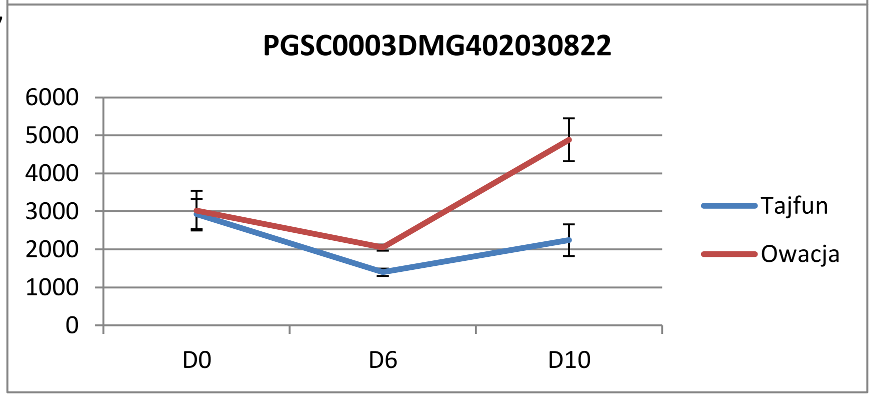

Table S1 The comparison of normalized number of reads for transcripts derived from genes searched out in the first round of selection (594 genes) during the time course of drought experiment. The gray color indicates the selected genes after the second round of selection.

Mean value of normalised reads per million for selected potato transcripts that were up-regulated in Gwiazda comparing to Oberon. Data are shown for days: 0, 6, and 10 of drought experiment and were taken from three biological replicates. SD at D0 was always not statisticly significant (*P* >0,05), while SD at D6 and D10 were always statisticly significant (*P* < 0,05, see also Fig 5). Potato gene accesion numbers were taken from Spud DB (www.potato.plantbiology.msu.edu). SD - standard deviations.

Gene accesion numbers	Gwiazda reads mean value			Oberon reads mean value			Gwiazda SD			Owacja SD		
	D0	D6	D10	D0	D6	D10	D0	D6	D10	D0	D6	D10
**PGSC0003DMG400001181**	56,67	1344,7	10047	51,33	2554,67	6480	36,46	390	1385,43	42,72	371,07	1841,31
**PGSC0003DMG400001621**	39,33	651,33	284,67	8,67	270	105	24,03	243,64	44,06	13,32	130,95	26,87
**PGSC0003DMG400002484**	220,67	628,33	613	251,33	1109,33	1326,5	35,39	240,14	93,5	54,37	103,27	47,38
**PGSC0003DMG400003079**	195,33	436,67	971,33	272,67	789,33	1495	28,02	67,66	142,9	16,17	328,68	97,58
**PGSC0003DMG400003978**	394,67	617,33	1011,3	355,33	254,67	627	143,53	126,21	227,74	164,76	134,53	100,41

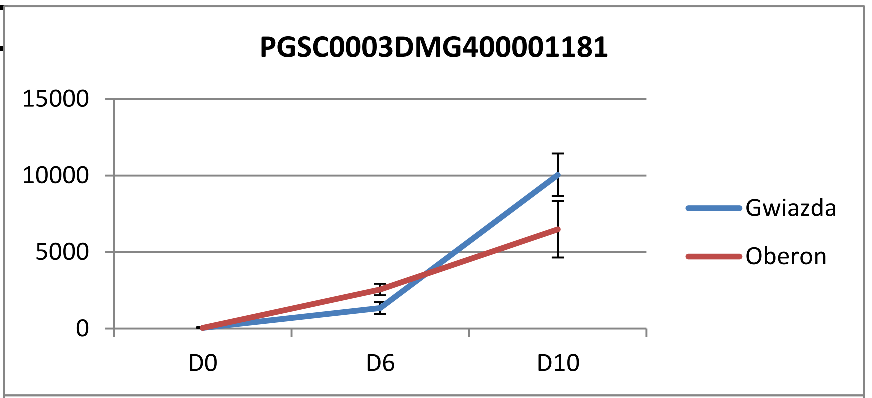

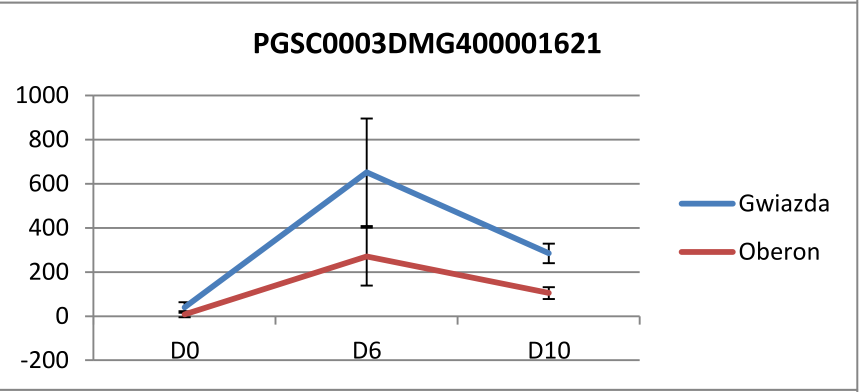

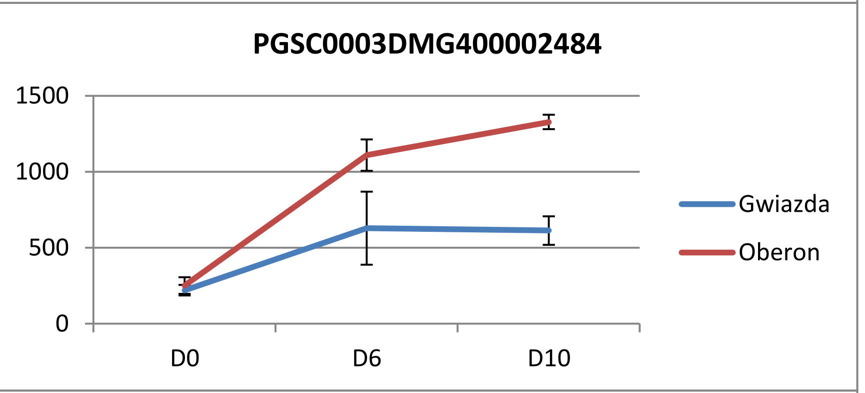

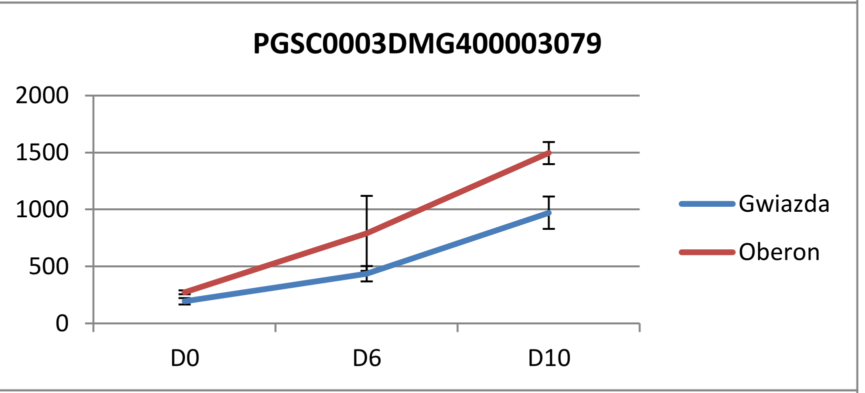

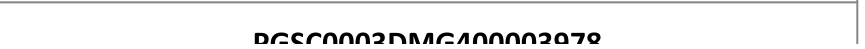

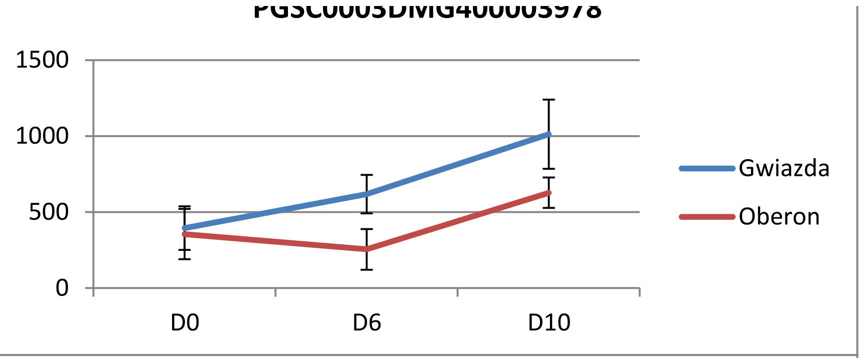

**PGSC0003DMG400006221**	333,33	590	916	418,67	1243,33	2042	61,78	131,19	117,12	60,14	266,19	489,32
**PGSC0003DMG400006796**	575,33	1474,7	1169,3	402,67	742	536,5	241,93	143,87	215	73,33	213,04	19,09
**PGSC0003DMG400006797**	2226,33	4478	3774,3	2181	2517,33	1642	759,96	321,27	432,03	512,59	801,07	15,56
**PGSC0003DMG400007109**	30,67	132	110	11,33	39,33	41	7,02	39,85	29,87	7,02	33,55	12,73
**PGSC0003DMG400007597**	54	243,33	279,33	92,67	98	141	29,05	45,45	35,8	93,39	69,2	46,67

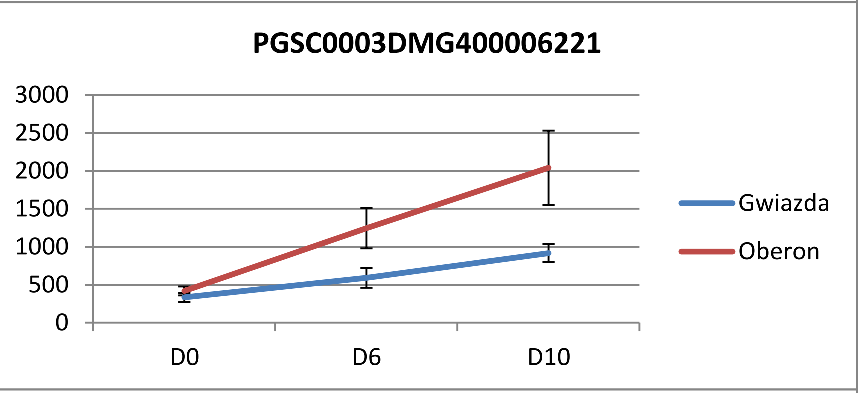

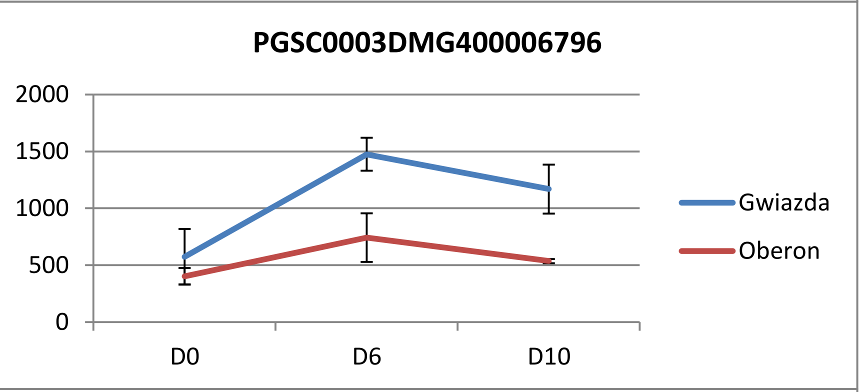

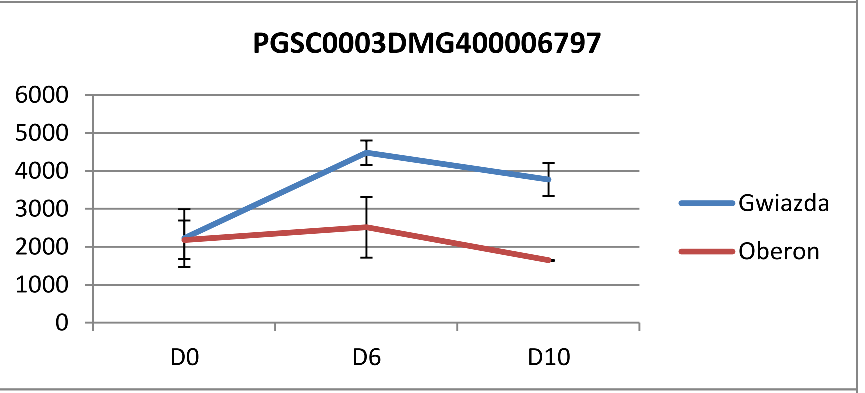

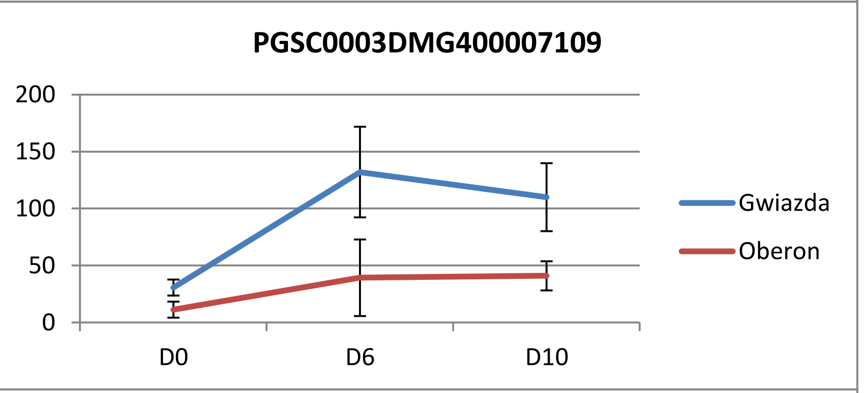

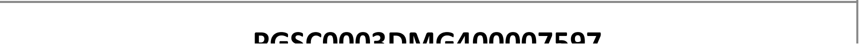

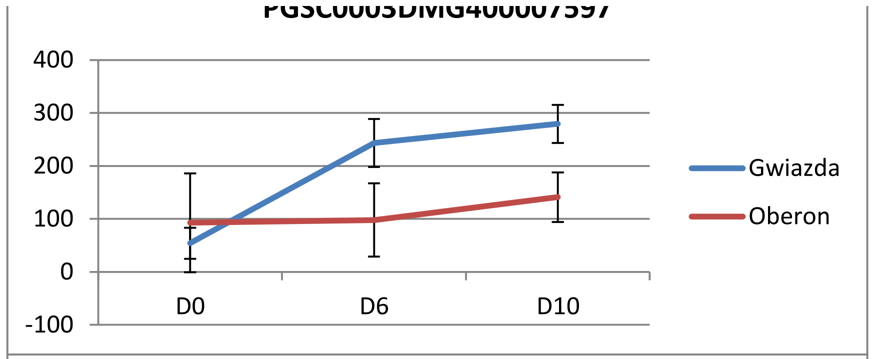

**PGSC0003DMG400007752**	170	315,33	535,33	85,33	148,67	280	52	45,71	91,53	11,02	32,88	45,25
**PGSC0003DMG400008164**	19,33	833,33	1064	2	406	581	3,06	412,82	286,19	3,46	74,32	100,41
**PGSC0003DMG400008833**	774	2652,7	2398	433,33	1324,67	903	213,23	726,39	140,76	121,17	224,9	9,9
**PGSC0003DMG400010065**	3506,67	5636	8460	3279,3	3064	5273	2188,34	285,51	1807,75	914,79	1243,41	482,25
**PGSC0003DMG400010172**	3,33	332	214	2,67	152	53	4,16	119,26	47,29	3,06	35,38	1,41

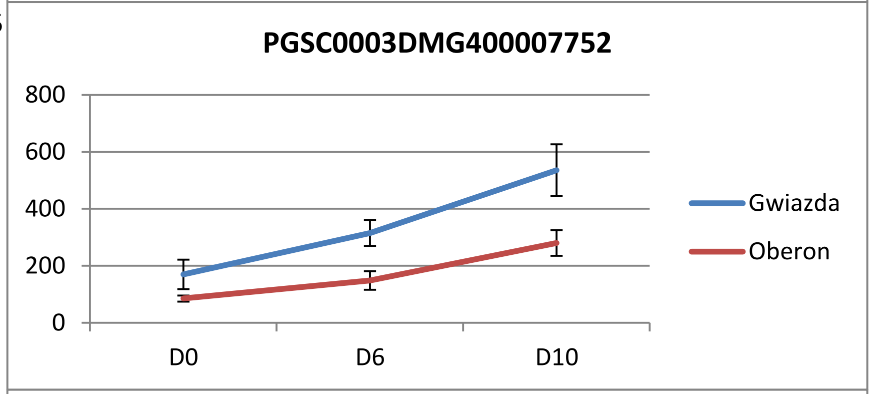

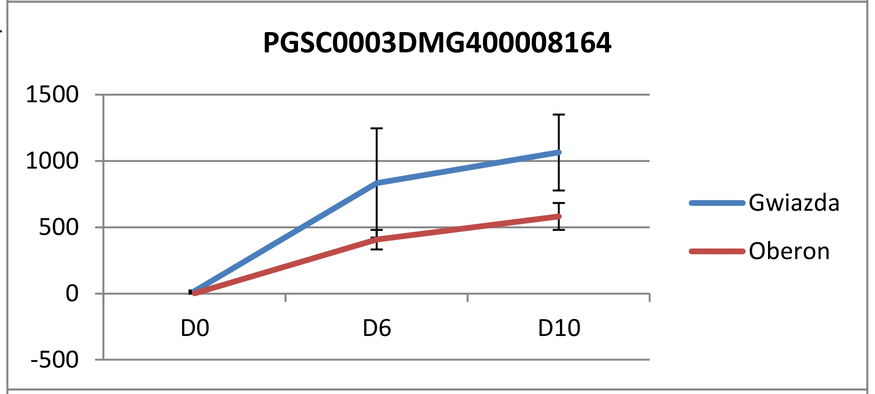

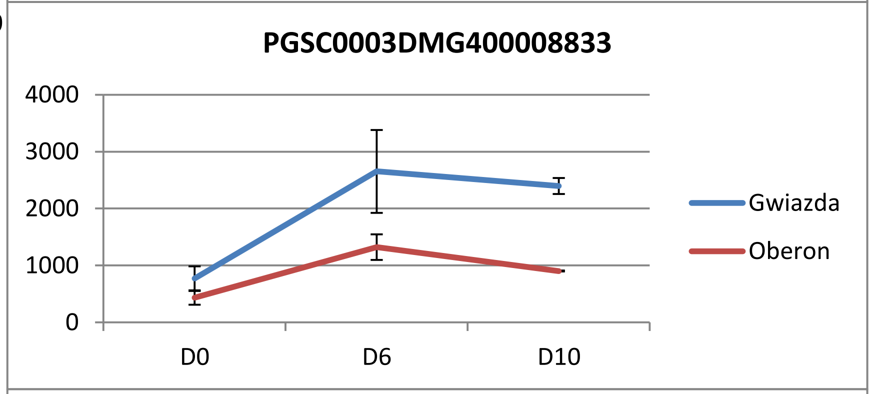

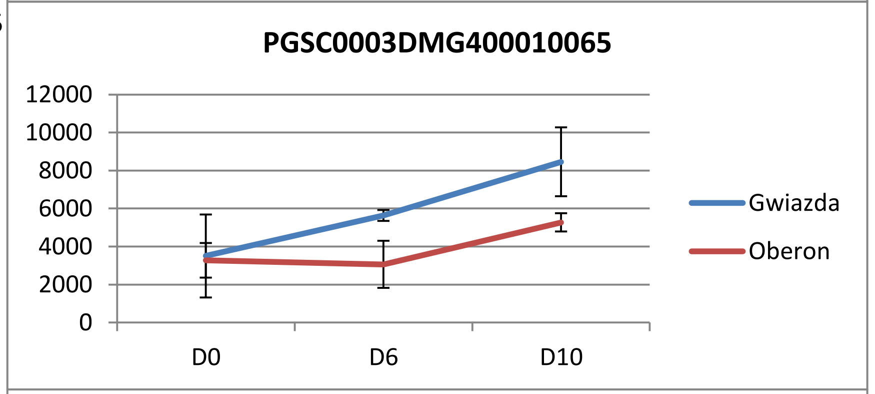

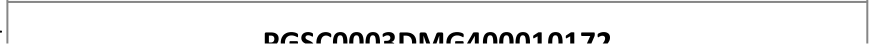

**PGSC0003DMG400010740**	1983,33	3169,3	6871,7	1703,3	1830	4015	113,74	456,25	719,79	126,02	743,29	250,32
**PGSC0003DMG400011208**	306,67	3442,7	827,33	189,33	1102,67	447	64,04	1742,87	166,76	59,54	391,22	117,38
**PGSC0003DMG400011630**	20,67	218,67	4656,7	42	478,67	12511	9,24	76,27	1052,9	17,78	126,64	3494,52
**PGSC0003DMG400011704**	52	244	228,67	18	86,67	94	8,72	52	23,86	7,21	57,49	0
**PGSC0003DMG400012166**	5305	8882,7	9824,3	6630,7	19694,3	17290	162,15	1848,15	115,52	1192,99	2262,5	401,64

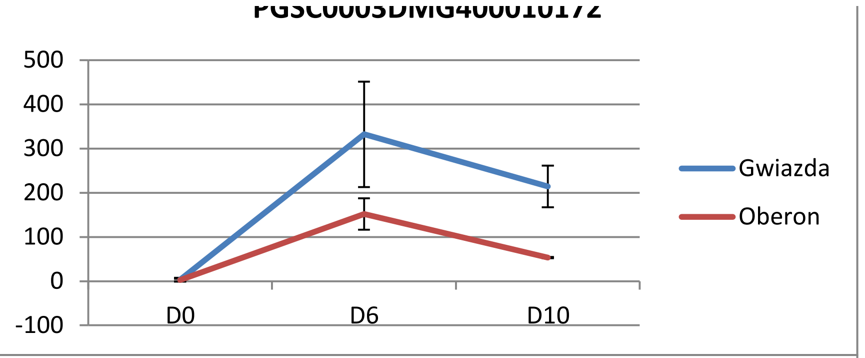

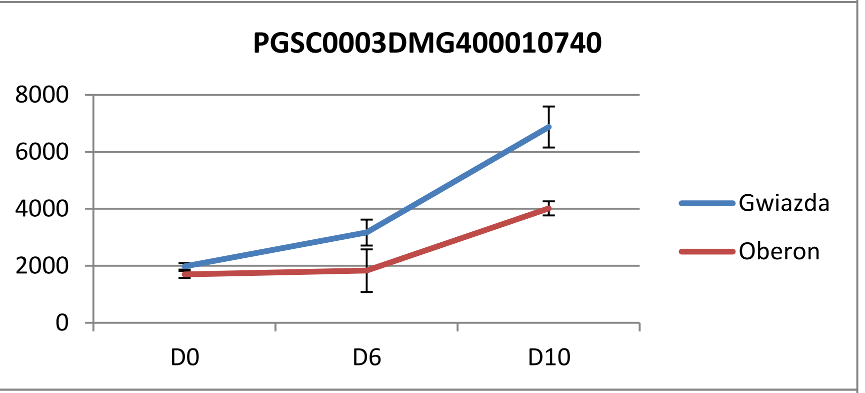

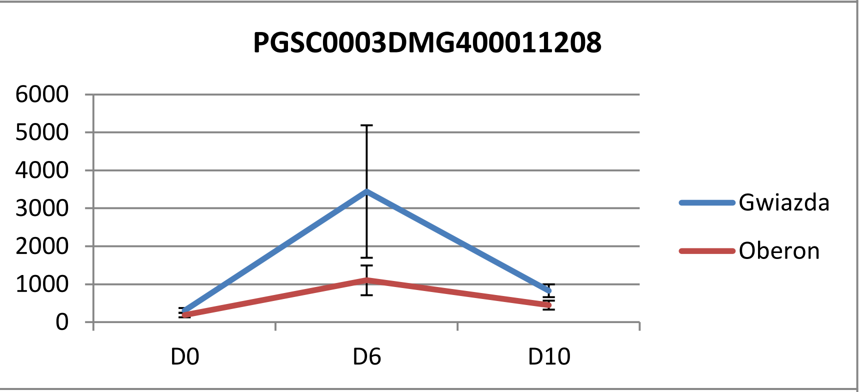

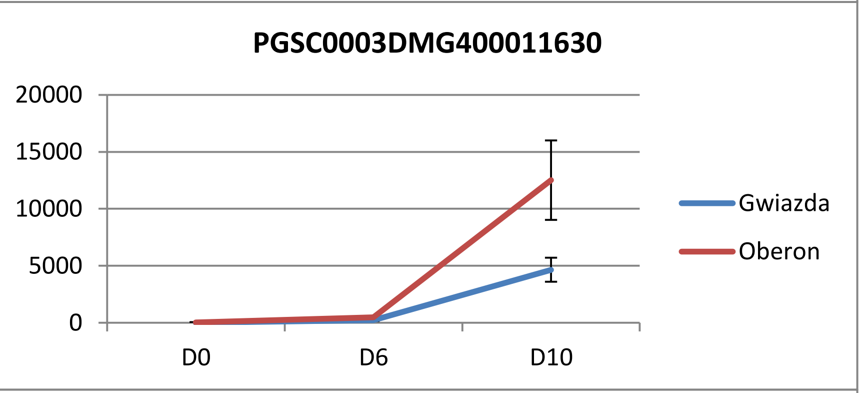

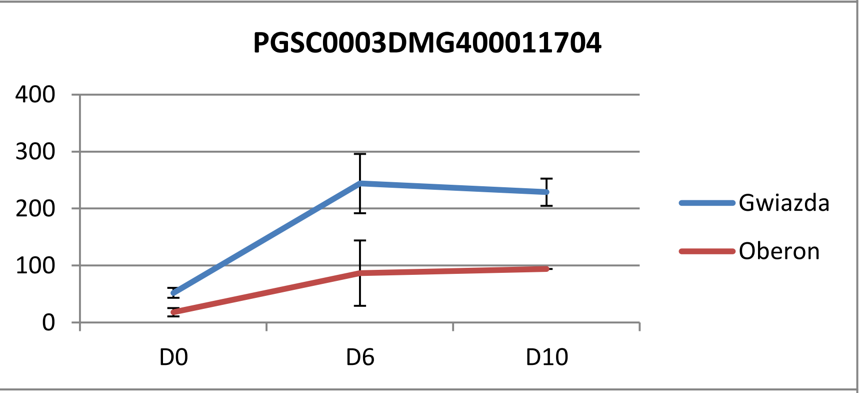

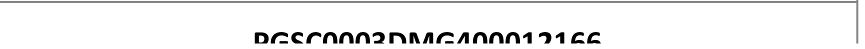

PGSC0003DMG400012174	77,33	10105	4630,7	19,33	4017	2168,5	68,86	4973,46	250,33	17,01	2451,5	526,79
PGSC0003DMG400012819	260,67	488	581,33	147,33	146	270	54,01	160,36	59,41	147	126,76	16,97
PGSC0003DMG400013977	54,67	230,67	126,67	22	48	56	37,65	155,31	31,01	20,3	6,93	0
PGSC0003DMG400014174	339,33	1033,3	1116,7	340,67	600	620	114,13	81,05	326,86	53,15	187,29	48,08
PGSC0003DMG400014226	116,67	375,33	228,67	84	92,67	102	24,11	35	49,69	73,73	84,06	33,94

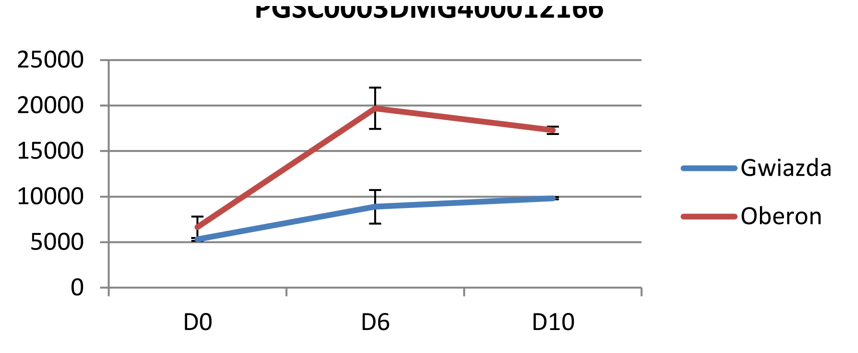

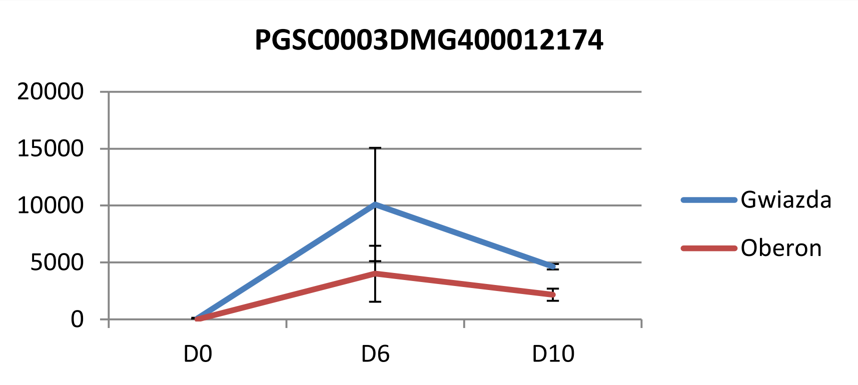

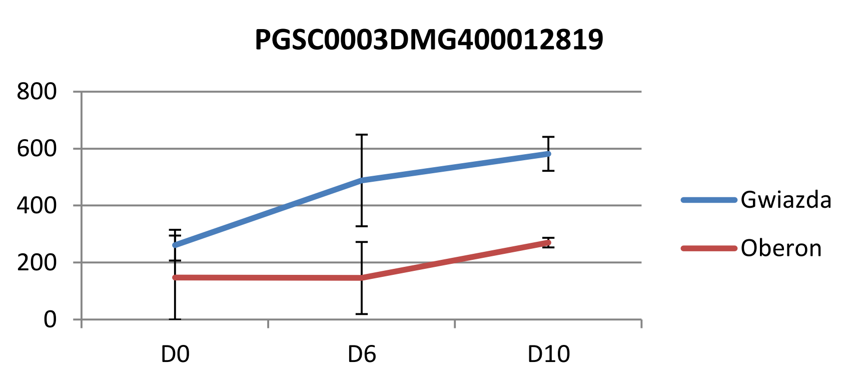

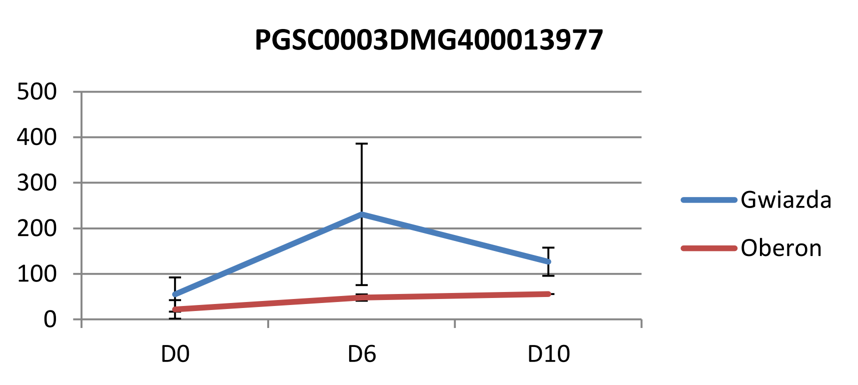

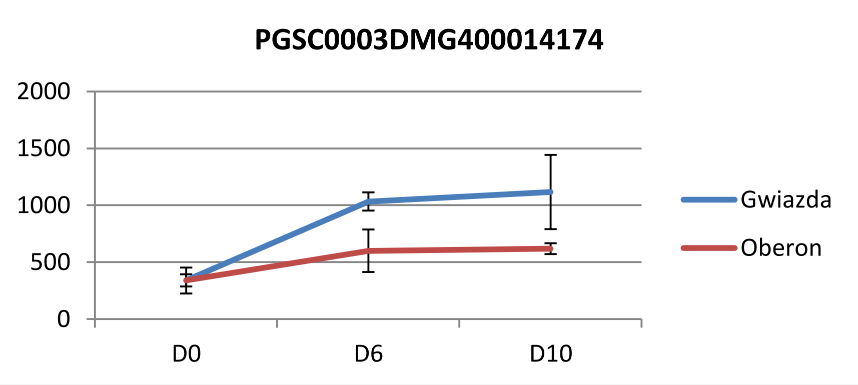

**PGSC0003DMG400014293**	179,33	34411	7558,7	24,67	9750,67	2654	158,31	14909,4	1139,27	16,17	981,43	675,99
**PGSC0003DMG400014449**	38	142	122	14	31,33	50	18	27,78	52	6	20,82	2,83
**PGSC0003DMG400015019**	15,33	198	258	10,67	89,33	100	23,09	96,99	84,64	5,03	12,06	56,57
**PGSC0003DMG400016032**	2,67	58,67	276	4	14	6	4,62	23,01	167,61	4	15,87	5,66
**PGSC0003DMG400016462**	44	477,33	266	8	74	0	76,21	36,02	97,08	13,86	69,54	0

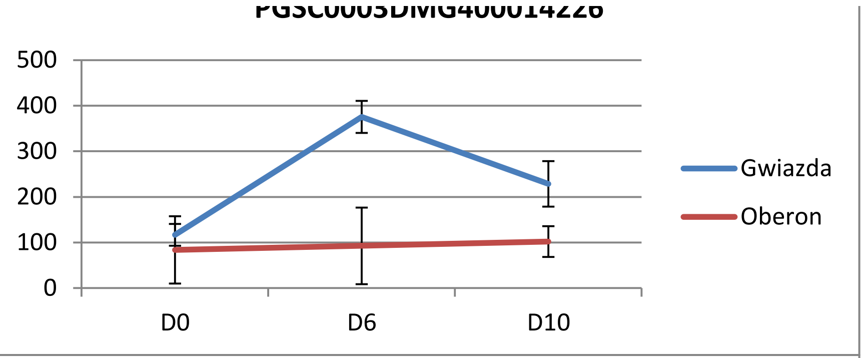

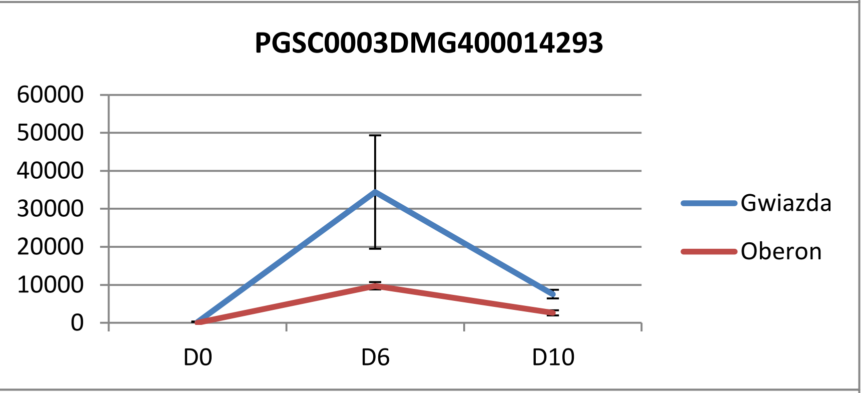

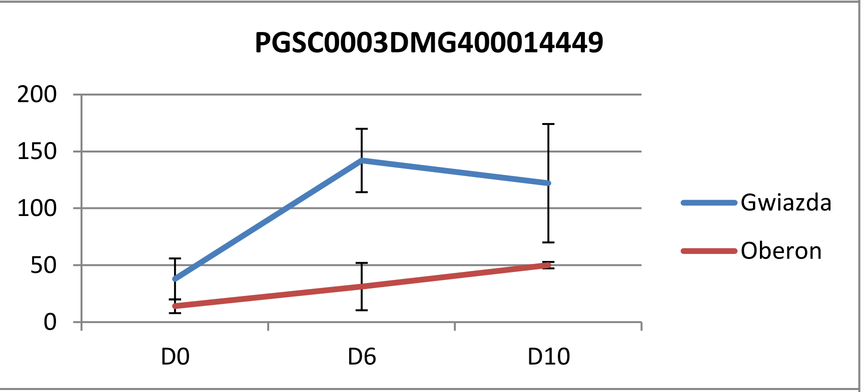

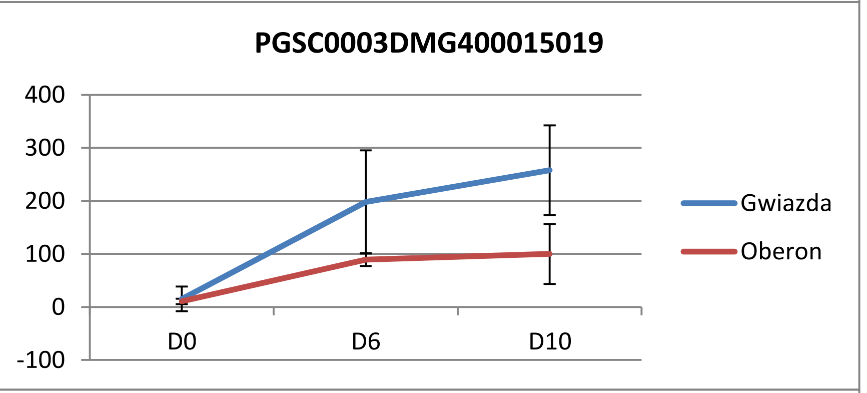

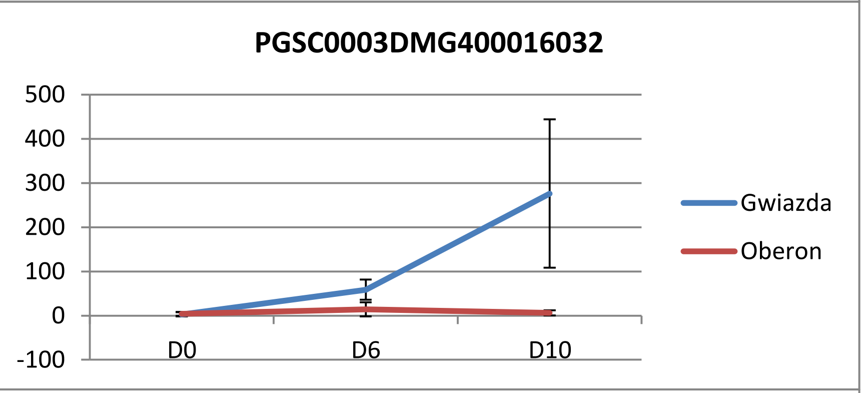

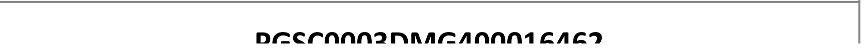

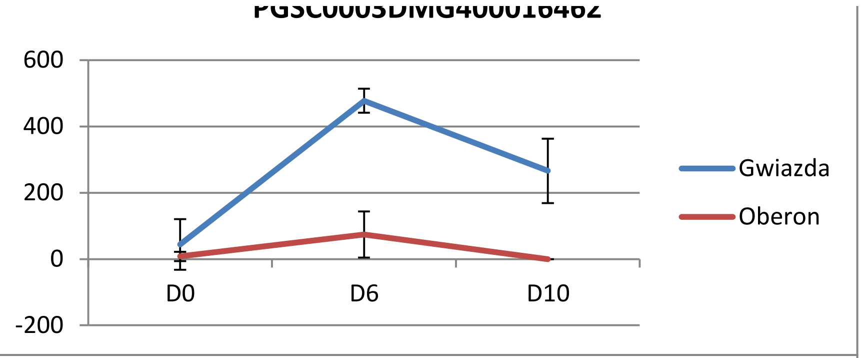

**PGSC0003DMG400016765**	13,33	496,67	141,33	4,67	1708	321	21,39	305,67	41,1	6,43	674,61	29,7
**PGSC0003DMG400017098**	1,33	46,67	627,33	1,33	8	126	1,15	33,01	275,23	1,15	2	82,02
**PGSC0003DMG400018116**	91,33	321,33	218	45,33	112,67	107	22,12	43	7,21	24,03	40,86	21,21
**PGSC0003DMG400018236**	4606,67	13853	17632	3239,3	5791,67	8150	375,3	6889,19	702,93	805,99	683,36	545,89
**PGSC0003DMG400018616**	644	3144	6009,3	416	1735,33	3591	71,02	1143,83	816,14	19,08	1022,59	182,43

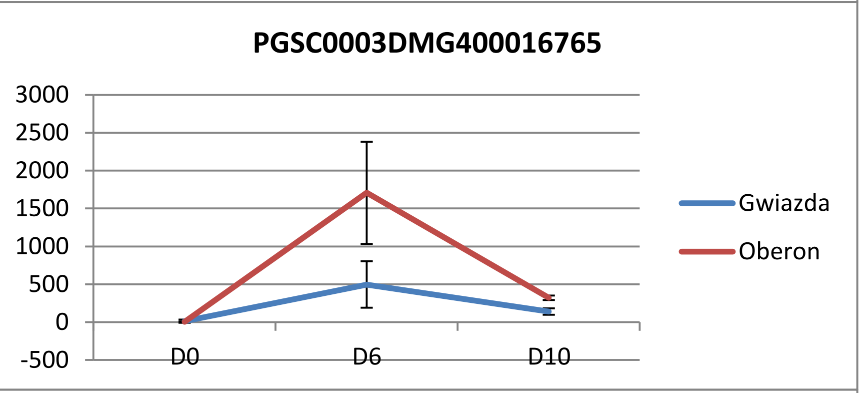

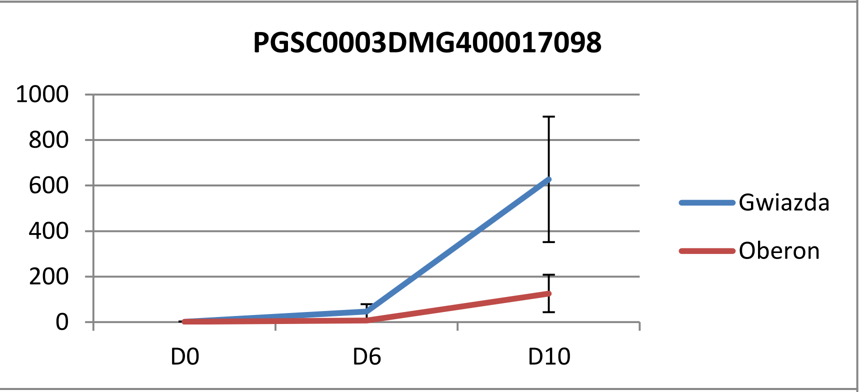

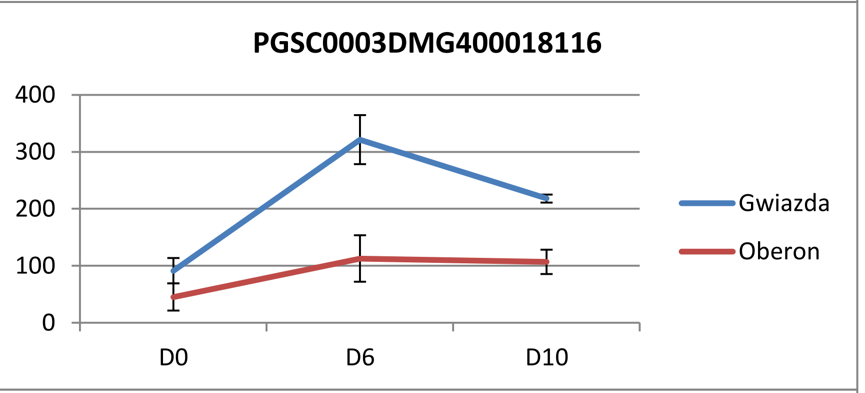

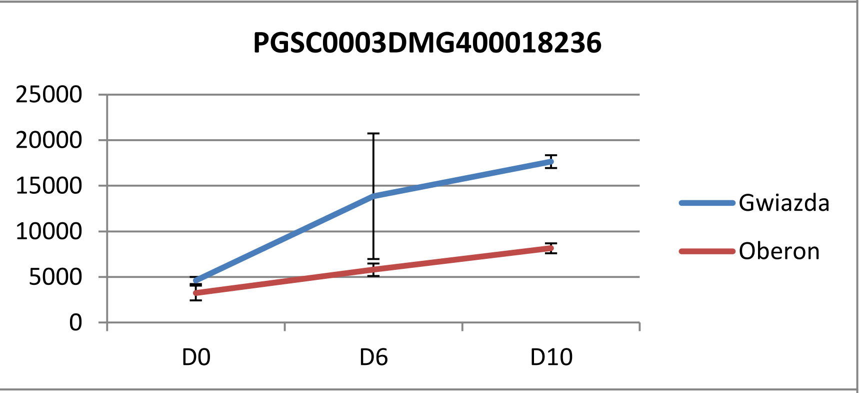

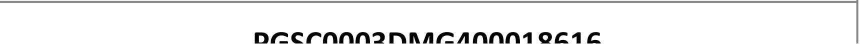

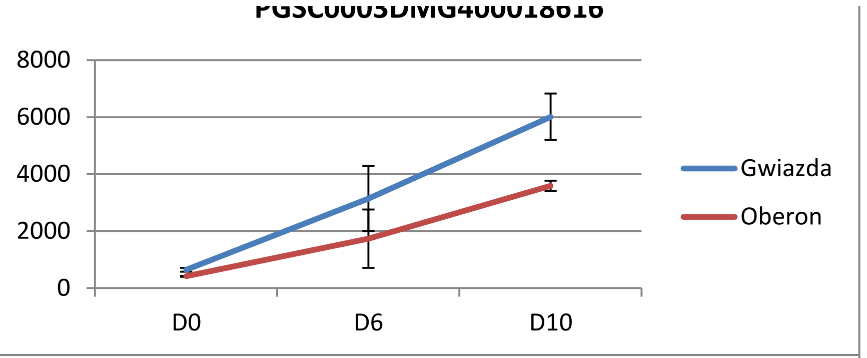

**PGSC0003DMG400019944**	88	460	2128	181,33	900,67	5097	26,91	204,66	353,65	163,15	316,89	15,56
**PGSC0003DMG400019976**	0,67	329,33	226,67	0	137,33	26	1,15	166,1	87,85	0	26,86	2,83
**PGSC0003DMG400020708**	700	2489,3	3800,7	438	922,67	2042	269,14	510,12	1052,92	151,95	277,87	562,86
**PGSC0003DMG400022056**	12	138	232	2,67	17,33	31	6	79,9	49,03	3,06	8,33	18,38
**PGSC0003DMG400022591**	57,33	347,33	228,67	18	124	99	5,03	145,18	71,45	6	69,54	21,21

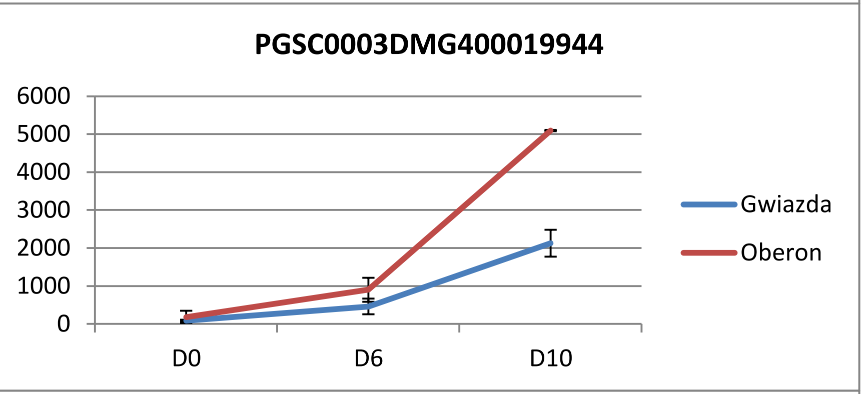

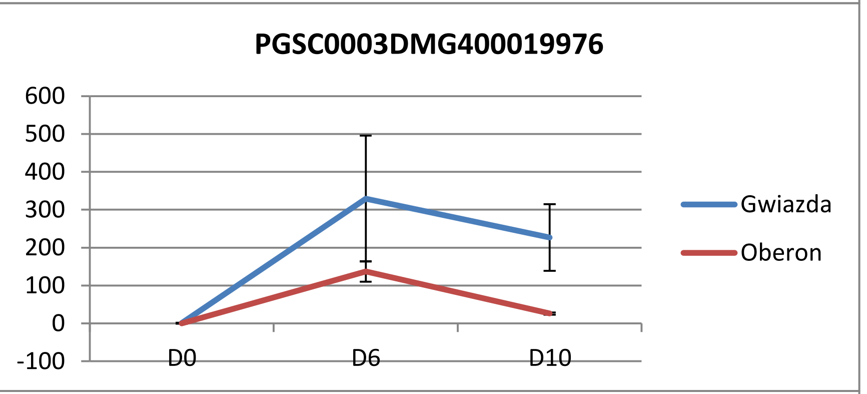

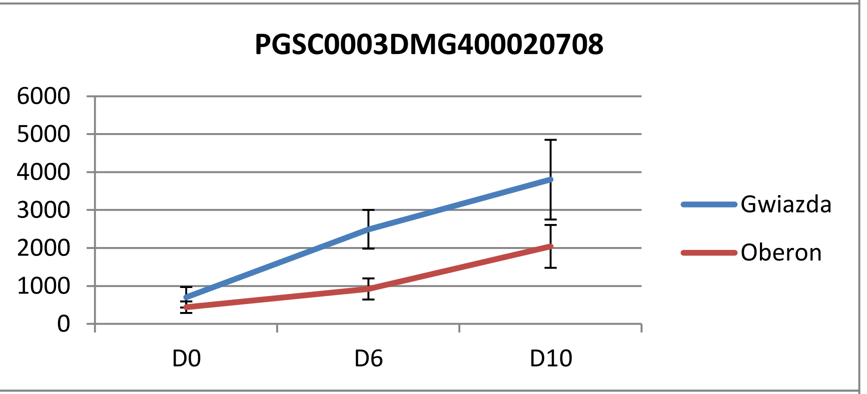

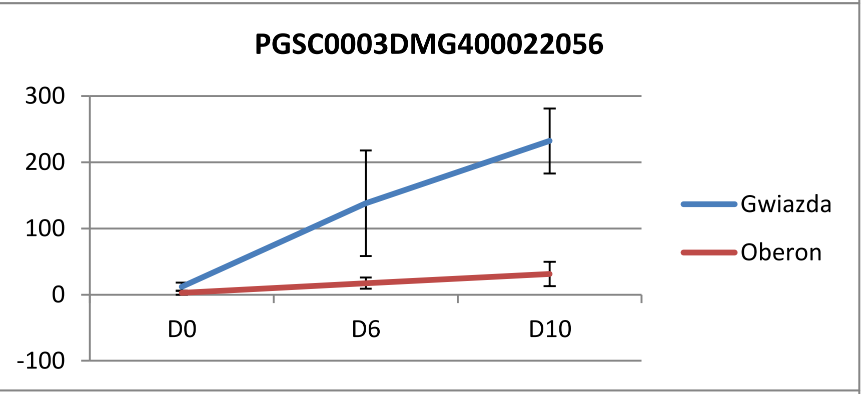

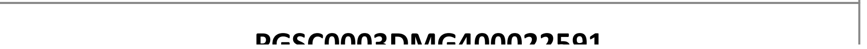

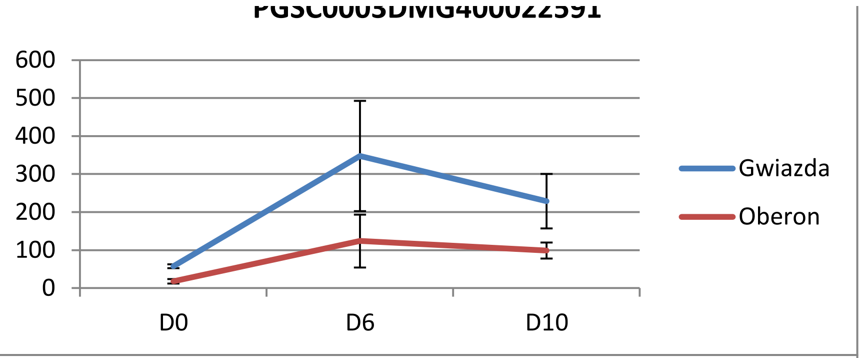

**PGSC0003DMG400024849**	17,33	1072,7	316	0	266,67	119	30,02	735,62	27,78	0	89,47	60,81
**PGSC0003DMG400025029**	72	160,67	180	32,67	64,67	90	68,35	47,38	15,1	30,35	59,14	25,46
**PGSC0003DMG400025299**	65,33	568	780,67	42,67	1183,33	1749	22,12	78	101,12	18,04	333,01	77,78
**PGSC0003DMG400026229**	54,67	134,67	208	68	418	480	32,88	28,31	45,74	24,58	220,03	25,46
**PGSC0003DMG400027143**	591,33	1363,3	5393,7	524	438	3194,5	150,43	250,65	428,94	223,17	62,48	309,01

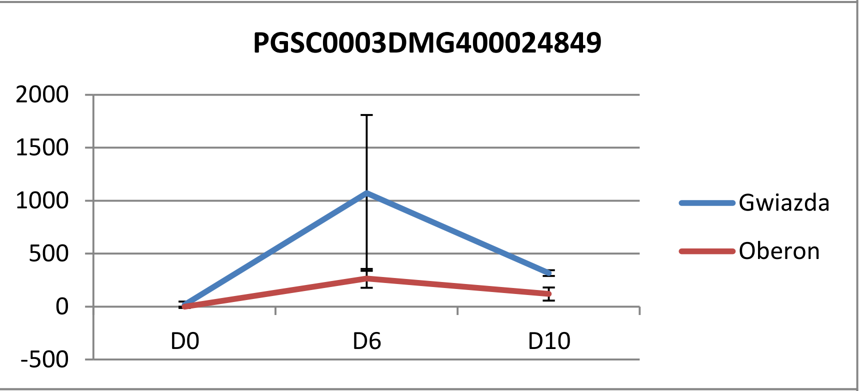

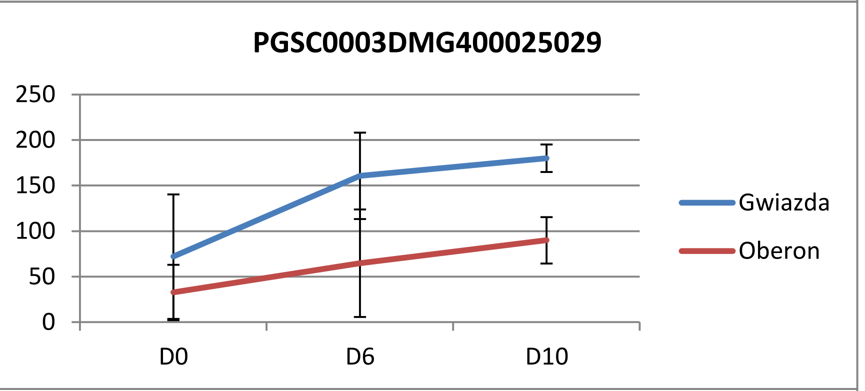

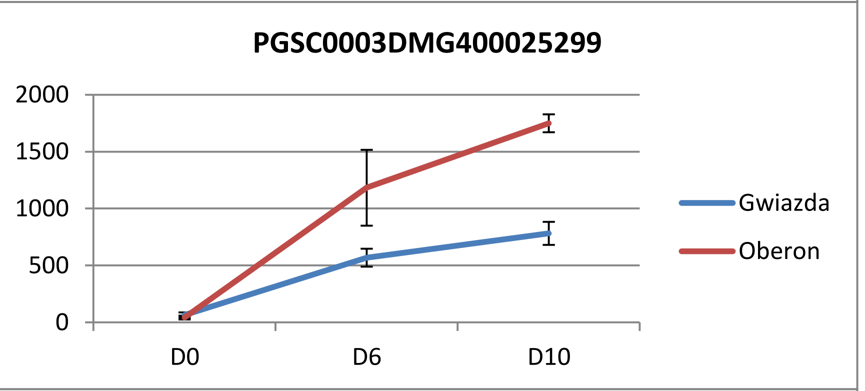

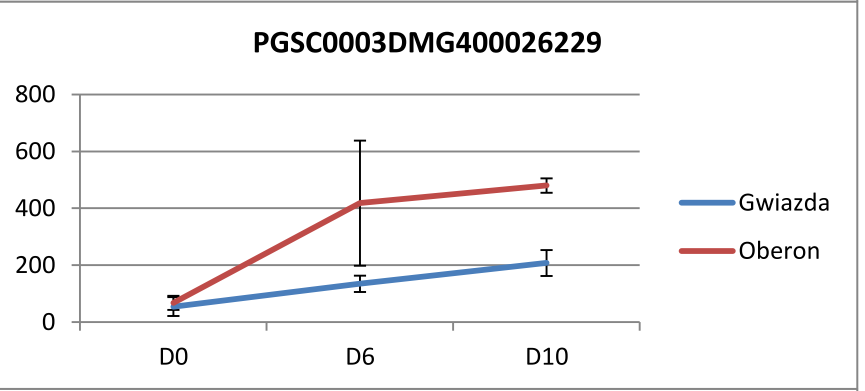

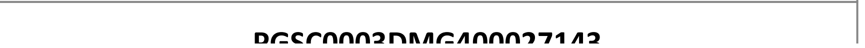

**PGSC0003DMG400027839**	354	879,33	2266	354	4409,33	3611	117,01	443,35	691,78	302,18	826,29	881,06
**PGSC0003DMG400029618**	3649,33	6872,7	11815	3884	12034,7	17711	775,85	3390,8	818,03	555,74	3326,03	1475,02
**PGSC0003DMG400029706**	1,33	140,67	150	0	44,67	35	1,15	64,29	47,16	0	9,87	18,38
**PGSC0003DMG400029822**	1019,33	2188,7	2085,3	590,67	701,33	909	641,94	416,08	333,95	239,12	435,37	287,09
**PGSC0003DMG400030427**	13,33	116,67	2426	24,67	334	7697	8,33	83,34	408,88	7,57	42,76	2832,67

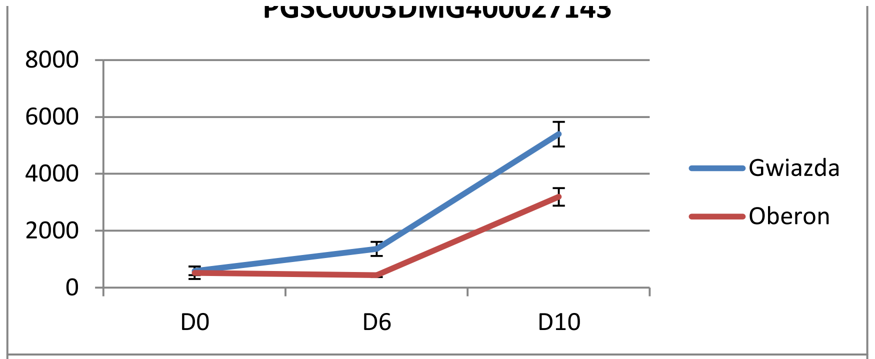

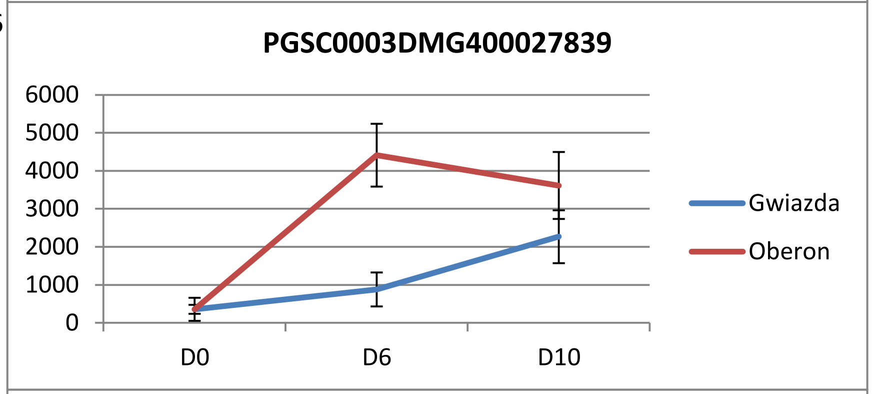

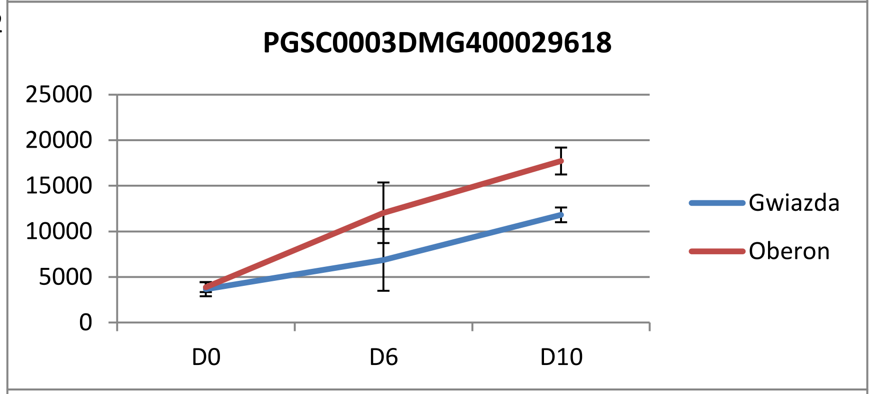

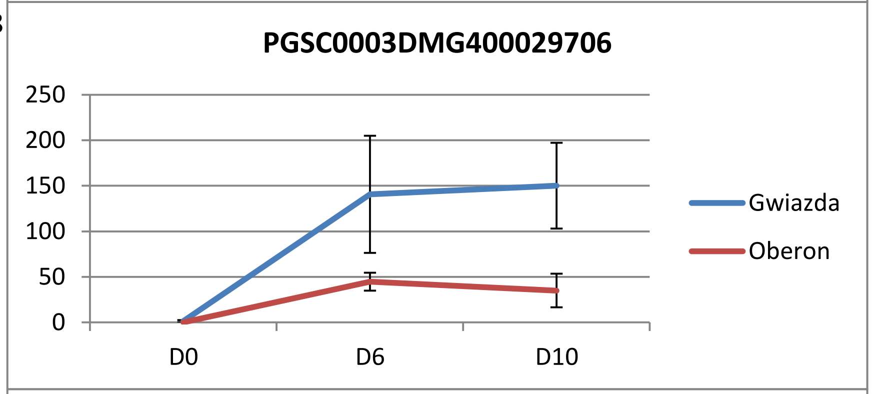

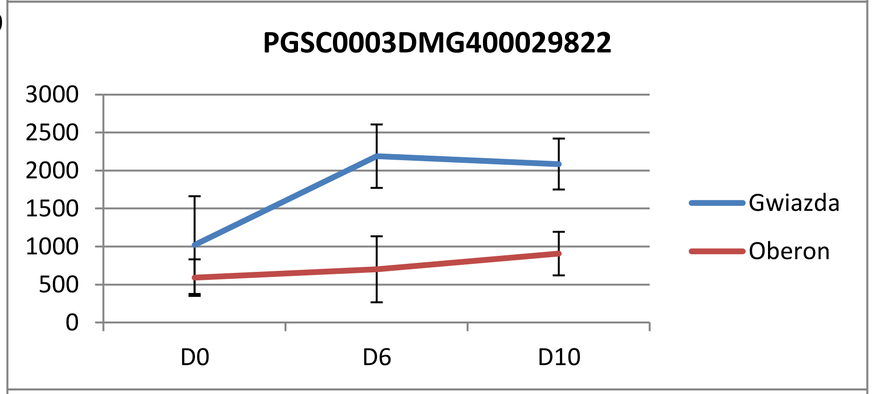

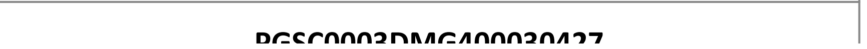

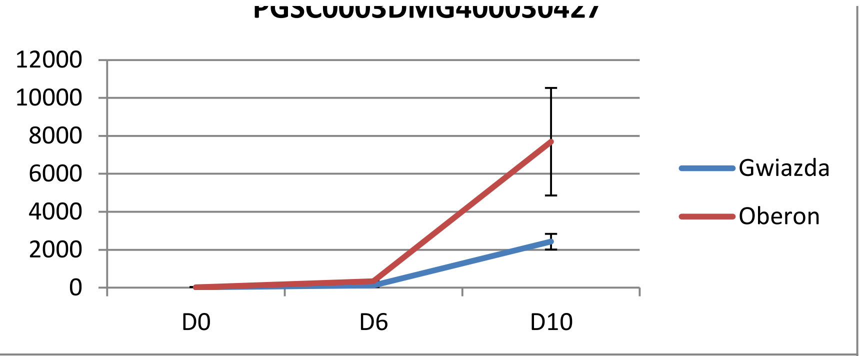

**PGSC0003DMG400032124**	1174	2409,3	2122,7	946,67	4040	4109	36,66	769,48	243,07	101,28	83,81	41,01
**PGSC0003DMG400033931**	11,33	71,33	400	1,33	222	43	3,06	52,17	172,94	2,31	67,73	1,41
**PGSC0003DMG400034307**	8239,33	13506	11898	4480,7	8089,33	8027	3153,69	2153,97	1529,67	1212,31	3226,37	595,38
**PGSC0003DMG400043108**	36,67	196	232,67	27,33	392,67	110	11,72	60	63,51	11,72	129,56	11,31
**PGSC0003DMG400045865**	104	377,67	1271,7	444,33	1701	4487	180,13	119,44	472,45	544,69	572,42	609,53

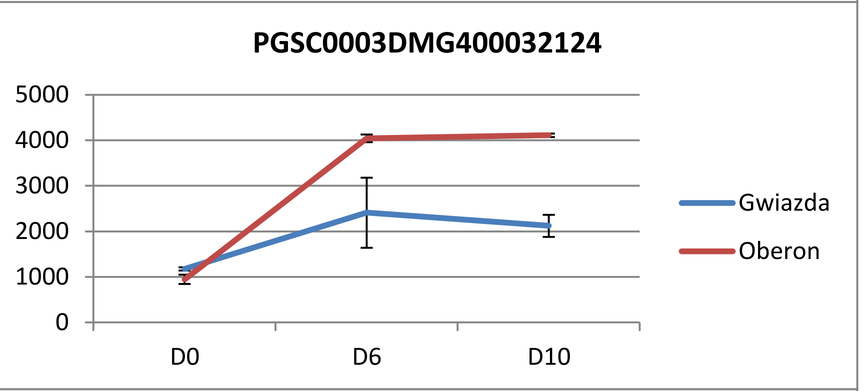

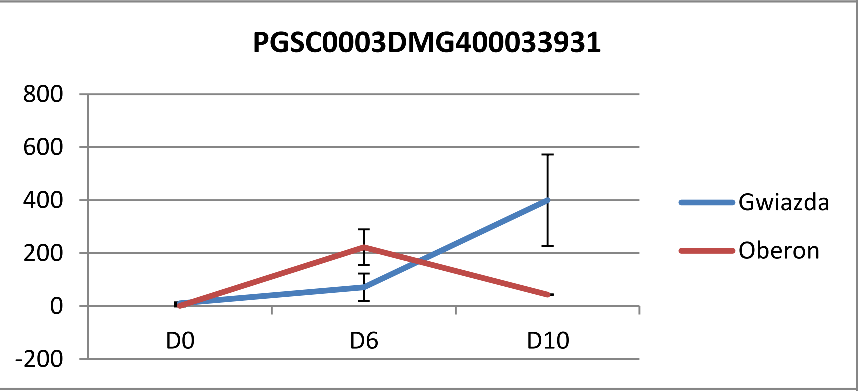

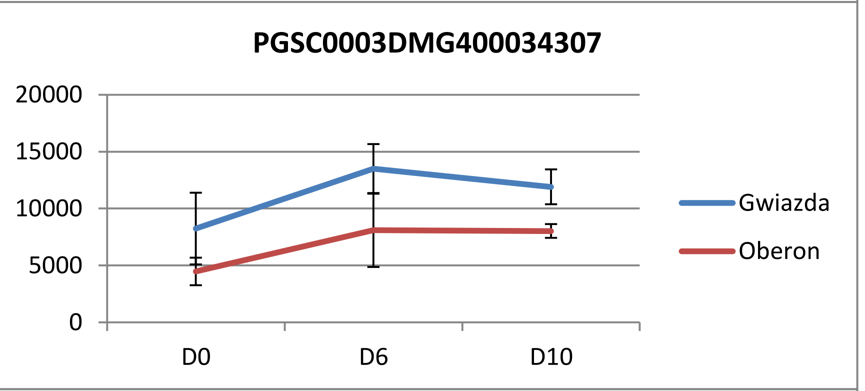

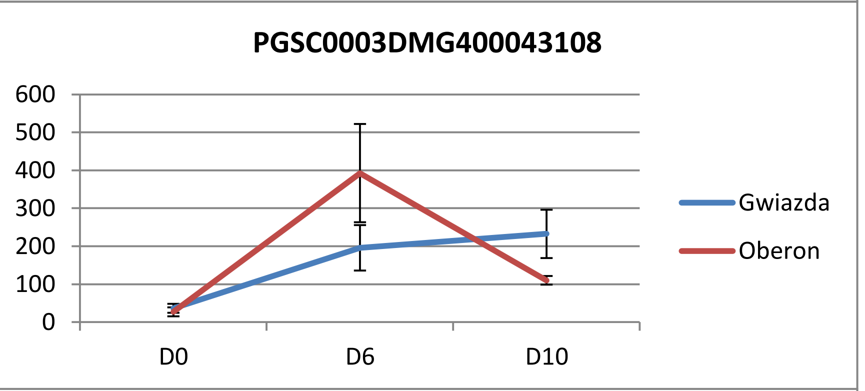

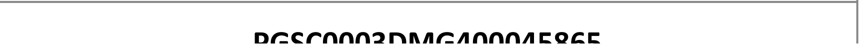

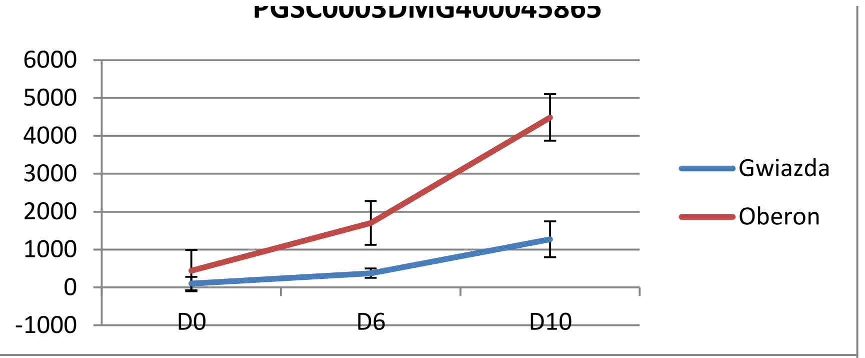

**PGSC0003DMG400047155**	11,33	230	376,67	0,67	78,67	136	3,06	113,86	114,01	1,15	11,02	42,43
**PGSC0003DMG401005180**	92,33	366	257,33	63	20,67	40,5	85,94	91,1	78,69	55,56	35,8	36,06
**PGSC0003DMG401015935**	11,33	490,67	2334,7	10	133,33	910	14,47	327,82	572,47	3,46	12,22	534,57
**PGSC0003DMG401017626**	395,33	1315,3	4904,7	354	732,67	2584	49,08	533,4	545,58	102,24	255,22	531,74
**PGSC0003DMG401020664**	631,67	2111,3	1150,7	455	1225,33	365	220,18	732,78	96,69	104,73	218,05	18,38

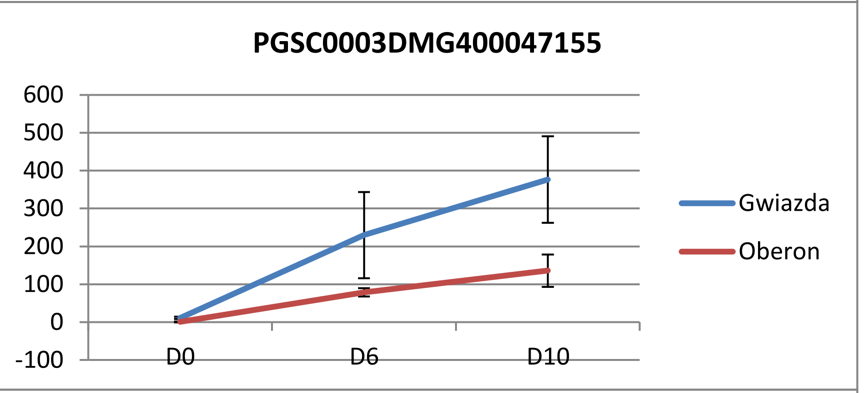

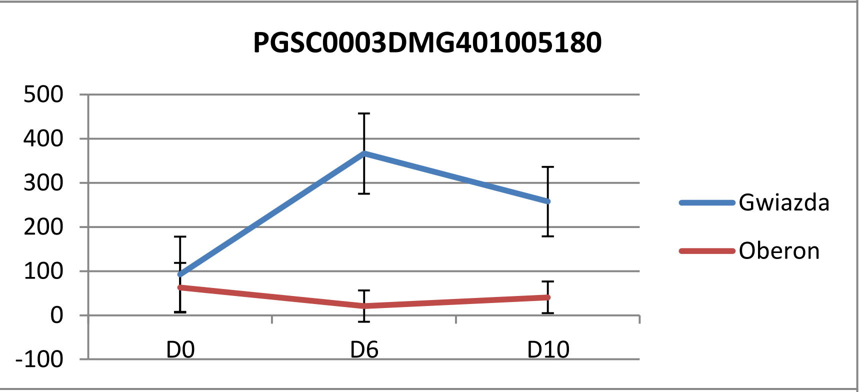

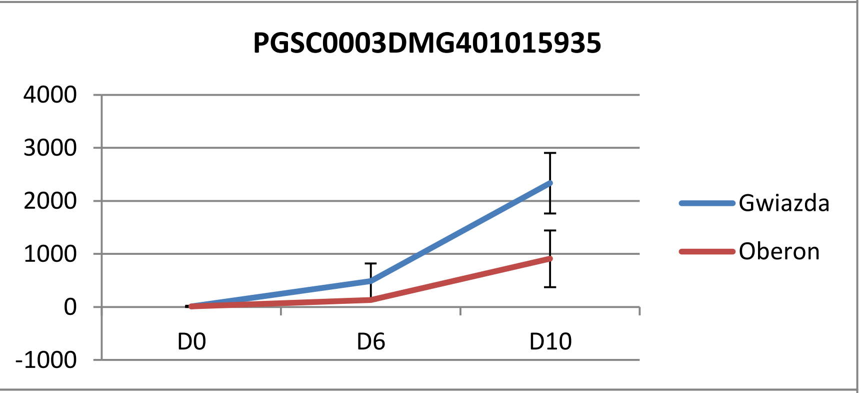

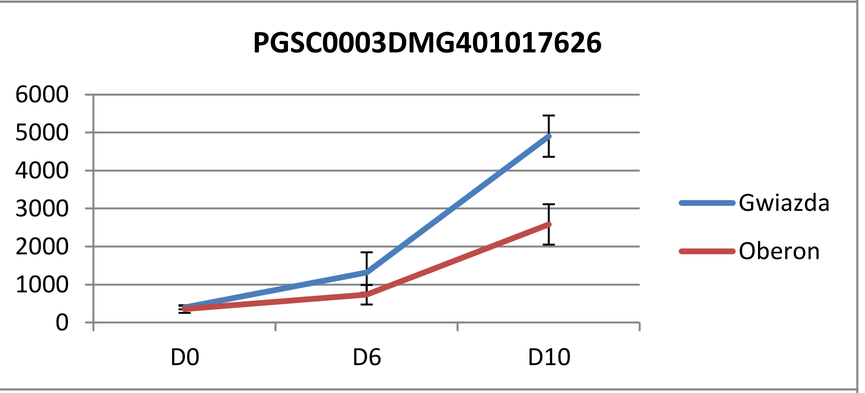

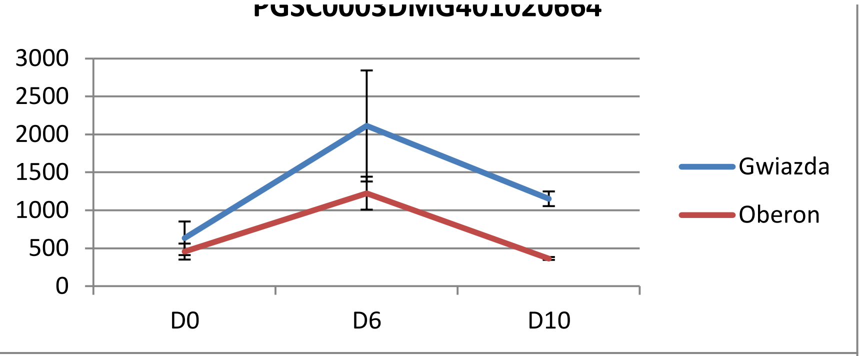

**PGSC0003DMG401023446**	226	848,67	452	125,33	322,67	237	27,78	453,98	83,59	28,87	208,1	77,78
**PGSC0003DMG401026044**	618	2813,3	2977	419,33	1503,67	1819	43,59	808,52	473,36	63,76	516,09	193,75
**PGSC0003DMG402007944**	10	716,67	162,67	1,33	5410,67	727	9,17	491,29	72,01	1,15	2681,69	97,58
**PGSC0003DMG402022579**	50,67	229,33	838	16	66	203	15,01	12,06	259,35	7,21	10,58	9,9
**PGSC0003DMG402028907**	473,33	3156,7	49239	619	5430,33	109157	59,01	723,34	5235,15	68,51	1344,33	53572,53

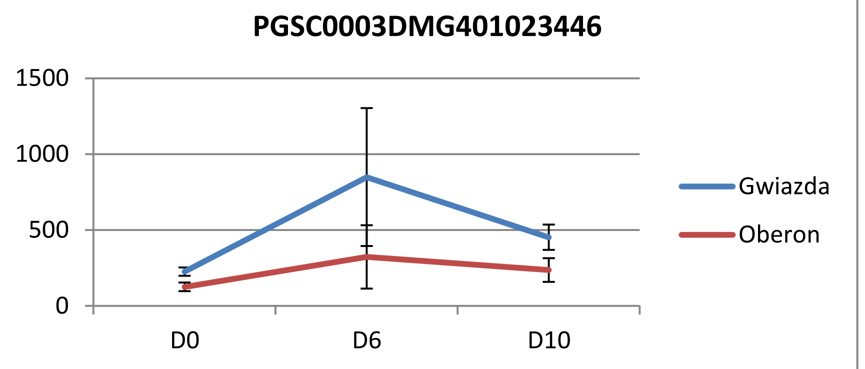

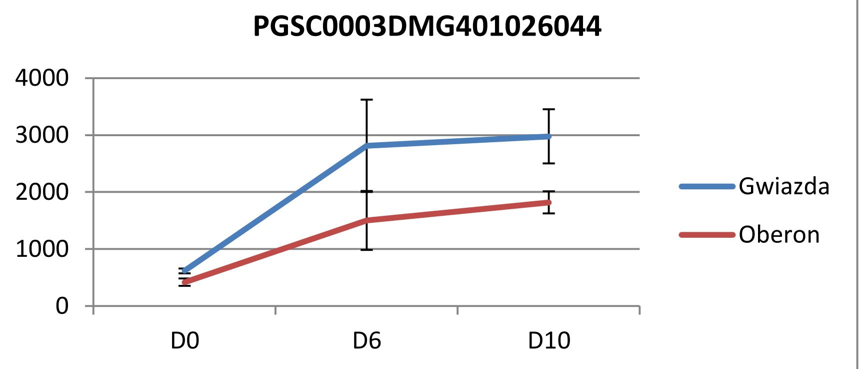

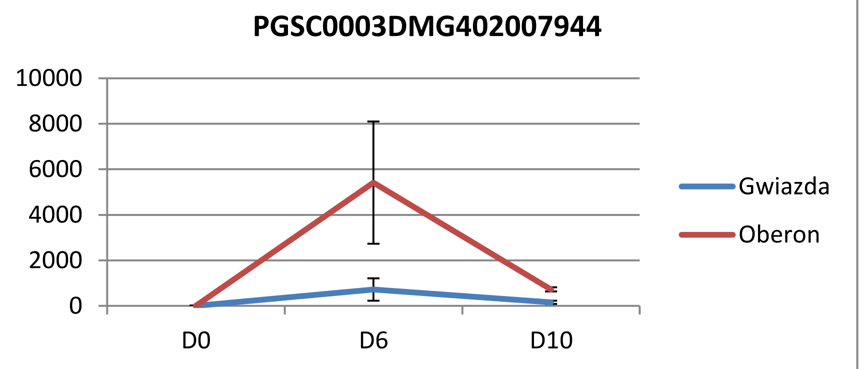

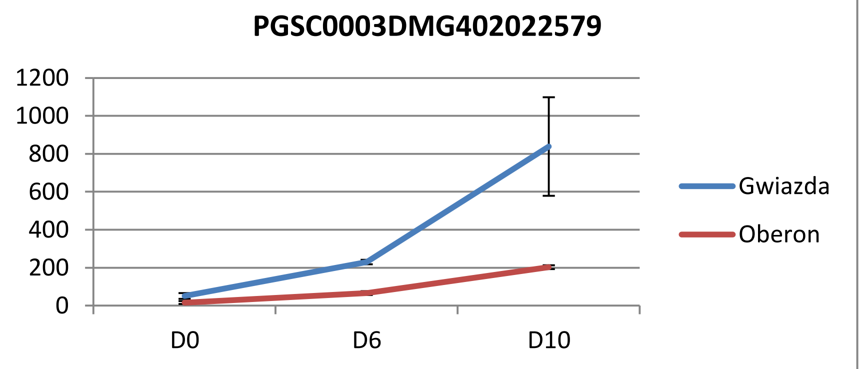

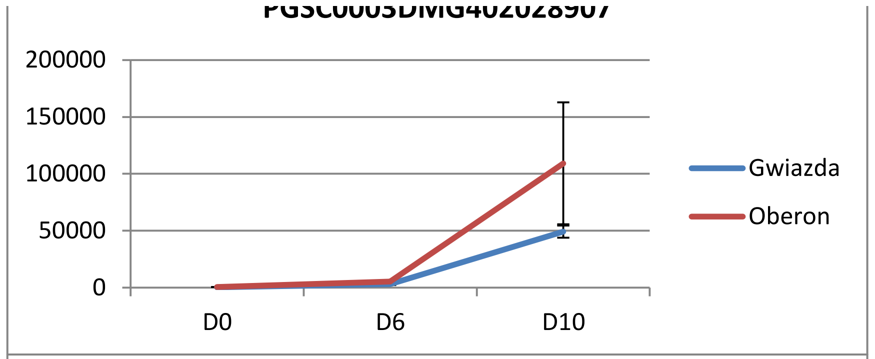

**PGSC0003DMG402031741**	954	1432,7	1620	612,67	630,67	539	272,08	318,18	322,16	202,33	408,59	179,61

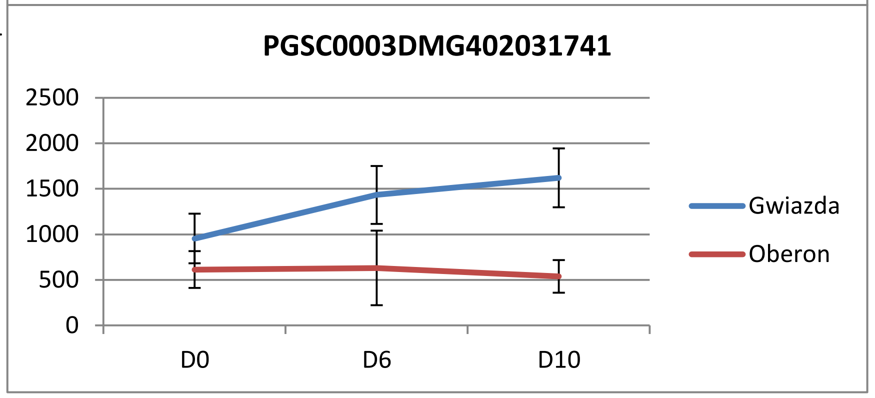

Table S1 Comparison of the normalized number of reads for transcripts derived from genes identified in the first round of selection (594 genes) during the time course of the drought experiment. Gray color indicates the selected genes after the second round of selection. The mean value of the normalized reads per million is shown for selected potato transcripts that were downregulated in the Gwiazda plants compared to those in the Oberon plants. The data are shown for days 0, 6, and 10 of the drought experiment and were obtained from three biological replicates. The SD at day 0 was never statistically significant (P > 0.05), while the SDs at day 6 and day 10 were always statistically significant (P < 0.05, see also Fig. 5). Potato gene accession numbers were obtained from the Spud DB (www.potato.plantbiology.msu.edu). SD - standard deviations.

Gene accesion numbers	Gwiazda reads mean value			Oberon reads mean value			Gwiazda SD			Owacja SD		
	D0	D6	D10	D0	D6	D10	D0	D6	D10	D0	D6	D10
PGSC0003I	154	0	0	176	126,67	38	72,99	0	0	55,75	9,24	8,49
PGSC0003I	447,33	119,33	169,33	384,67	256,67	375	59,34	33,25	50,77	37,86	76,56	69,3
PGSC0003I	365,33	168,67	41,33	607,33	652,67	278	139,03	31,01	37,75	254,26	454,02	104,65
PGSC0003I	487,33	238	148	622	855,33	435	161,86	73,51	28,35	241,72	463,43	89,1
PGSC0003I	24316	13704,7	3838,7	16590	8044	2247	5757,96	2149,29	595,34	1424,32	1845,73	598,21

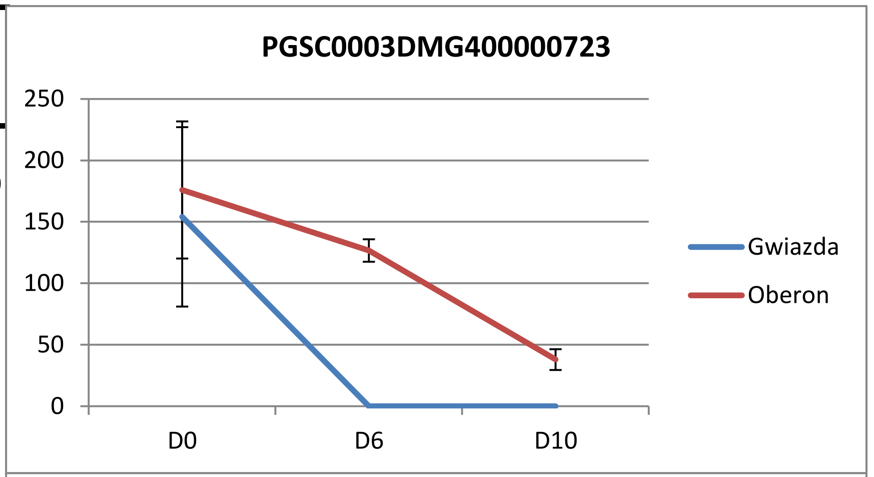

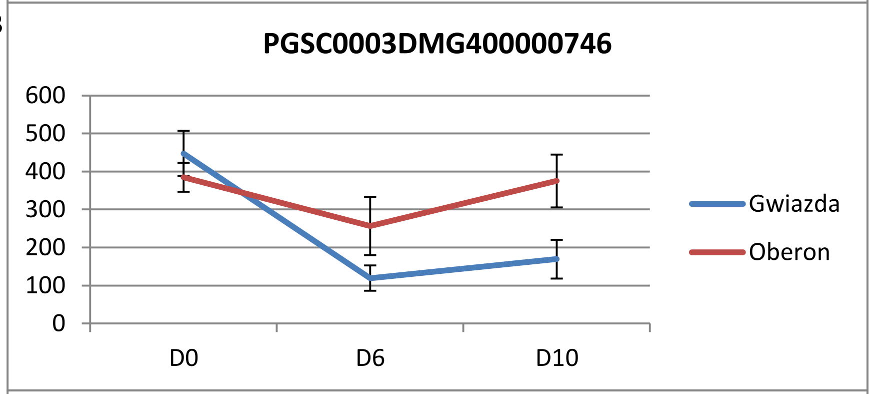

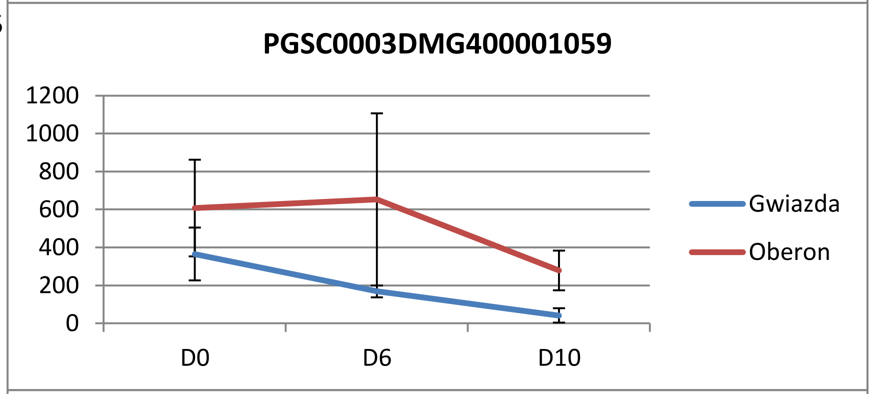

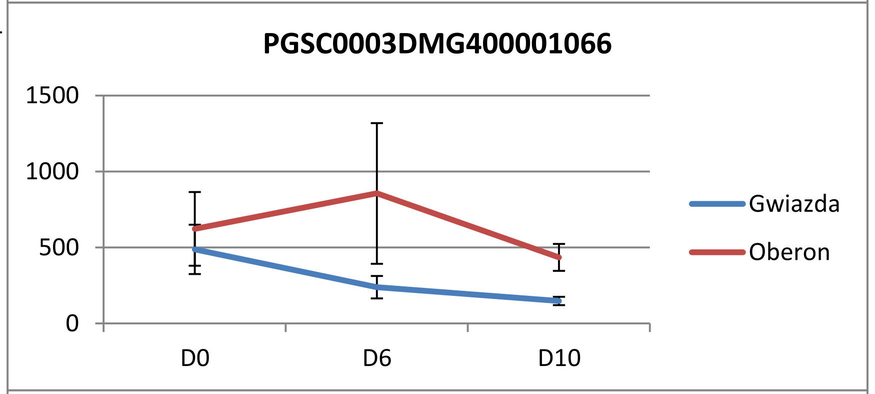

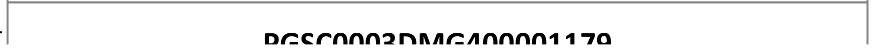

**PGSC0003I**	3528,67	828,67	226,67	5106,67	3750	966	1660,81	372,22	93,09	2109,04	865,04	311,13
**PGSC0003I**	12024,7	4533,33	2997,3	15102,7	9184	5500	3681,99	1120,61	487,5	3814,03	4348,95	299,81
**PGSC0003I**	17990	2802	1880	23061,3	8048	3806	2235,49	107,01	330,29	1090,95	934,62	755,19
**PGSC0003I**	333,33	57,33	20,67	586	800,67	104	260,04	57,77	18,58	260,37	583,21	42,43
**PGSC0003I**	1843,33	288	64	1988,67	791,33	191	634,46	57,17	13,11	508,13	515,3	15,56

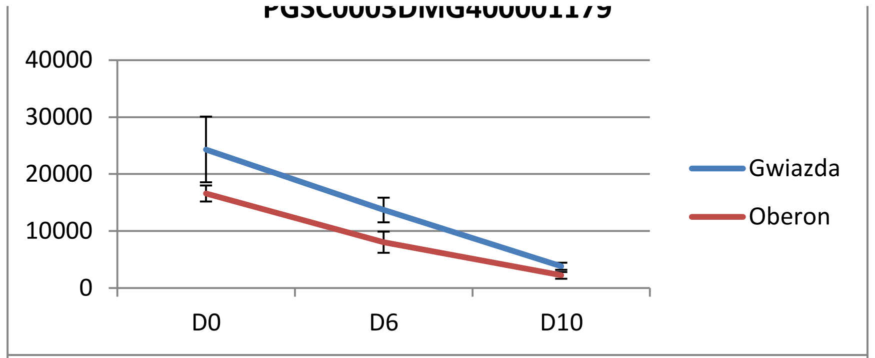

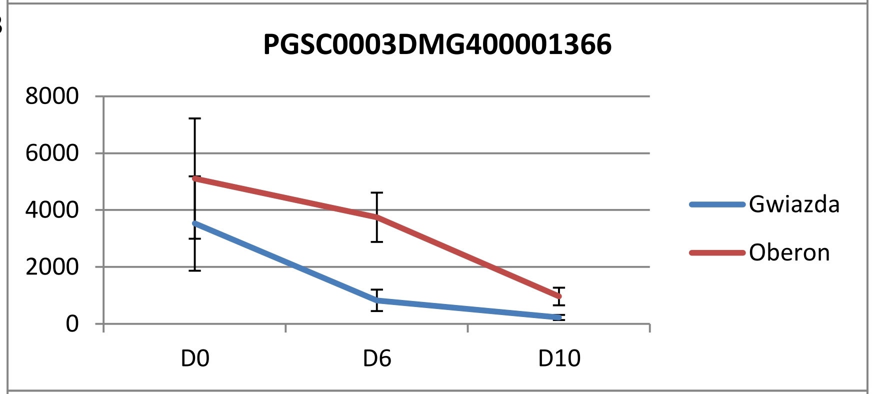

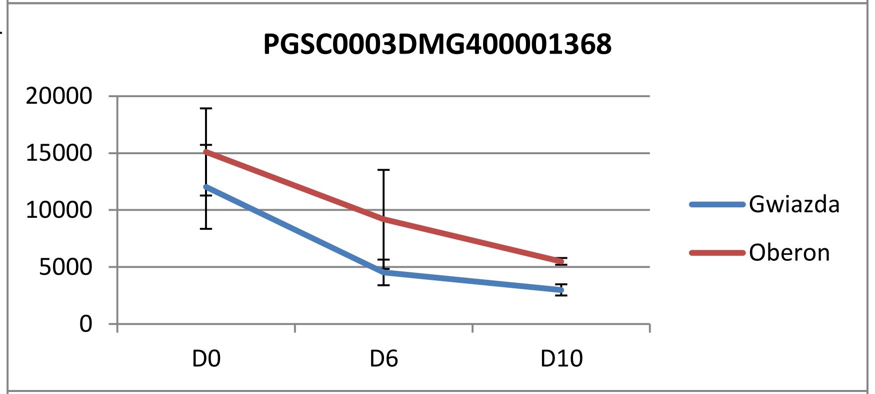

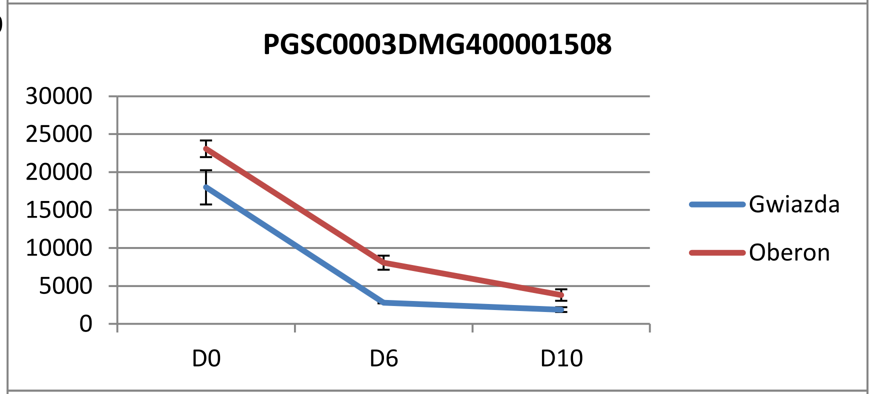

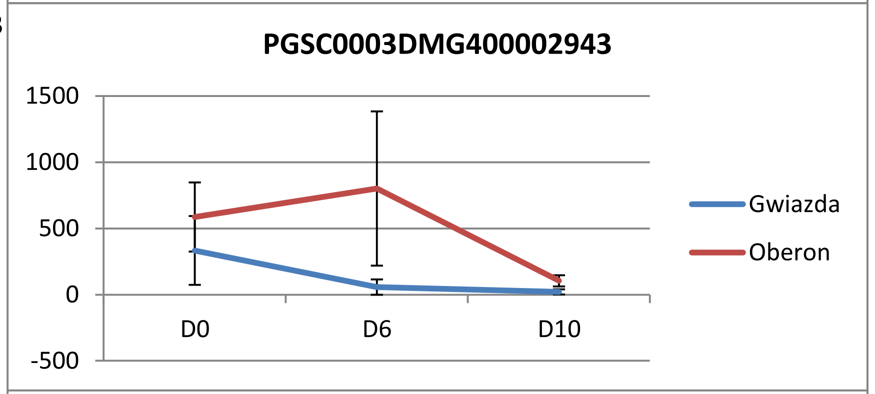

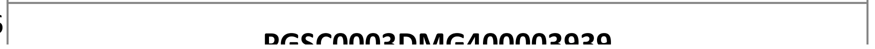

**PGSC0003I**	1294,67	137,33	113,33	862	318	242	584,69	67,12	35,12	225,25	64,37	28,28
**PGSC0003I**	445,33	86	75,33	292	196	171	92,46	43,86	40,07	86,56	53,03	60,81
**PGSC0003I**	1655,33	868	914,67	1687,33	1927,33	1426	364,12	411,13	133,05	279,82	299,07	45,25
**PGSC0003I**	556	248,67	162	661,33	505,33	314	71,36	17,47	66,9	141,89	117,17	65,05
**PGSC0003I**	213,33	79,33	85,33	239,33	199,33	180	24,11	47,43	26,63	14,47	66,16	8,49

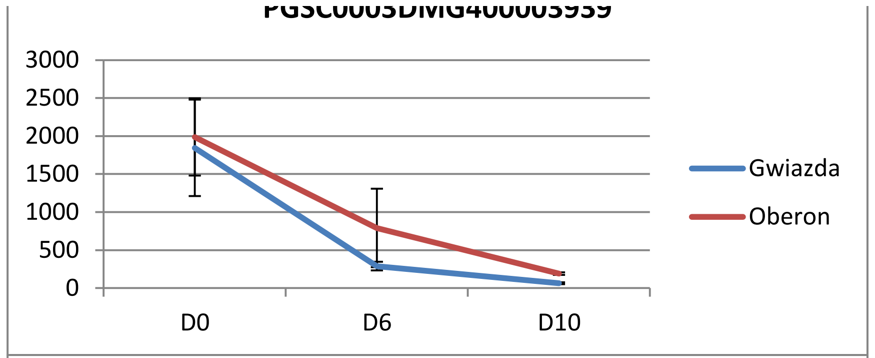

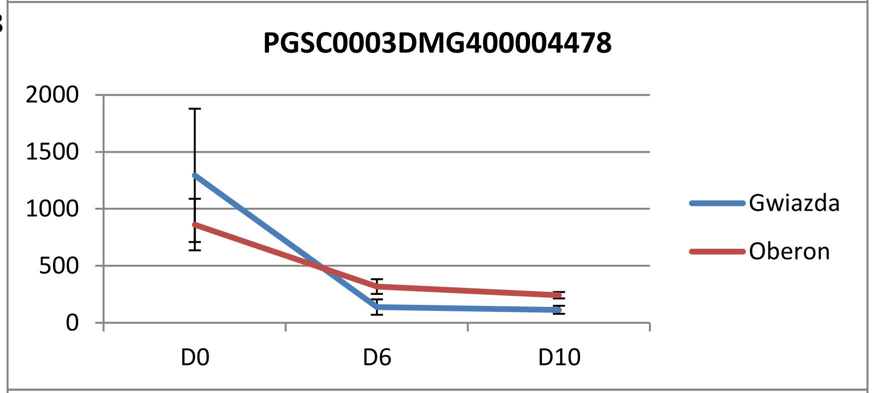

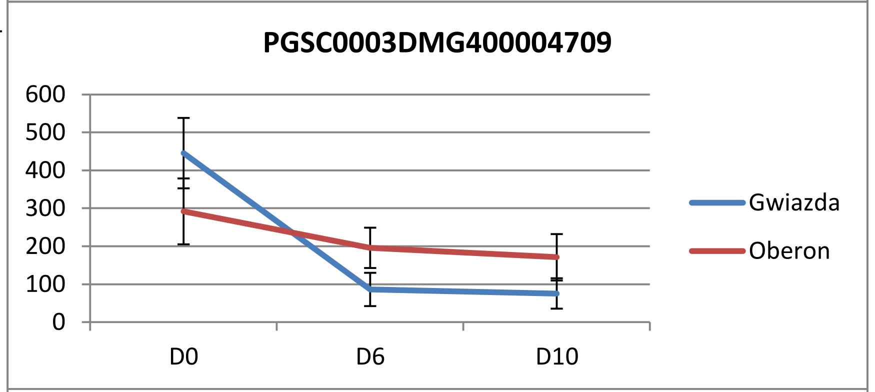

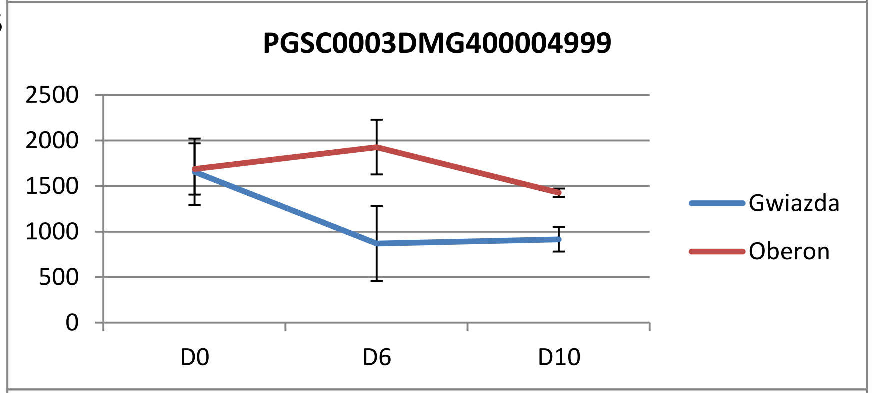

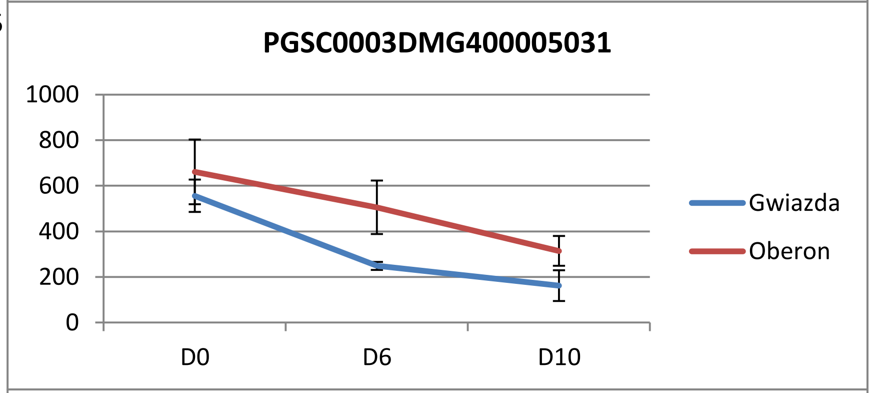

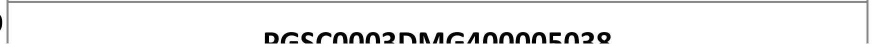

**PGSC0003I**	1272,67	590	628,67	1245,33	1232,67	1106	103,56	178,3	61	115,49	128,1	203,65
**PGSC0003I**	1047,33	219,33	67,33	1400,67	790,67	160	342,07	54,05	45,71	38,44	593,03	36,77
**PGSC0003I**	729,33	186,67	100,67	776,67	824,67	417	396,63	109,28	31,01	333,52	388,34	239
**PGSC0003I**	986,67	352	490	1194,67	779,33	909	246,98	69,4	55,68	272,15	464,66	89,1
**PGSC0003I**	342	0	47,33	446,67	66,67	179	58,92	0	41,3	141,74	76,38	49,5

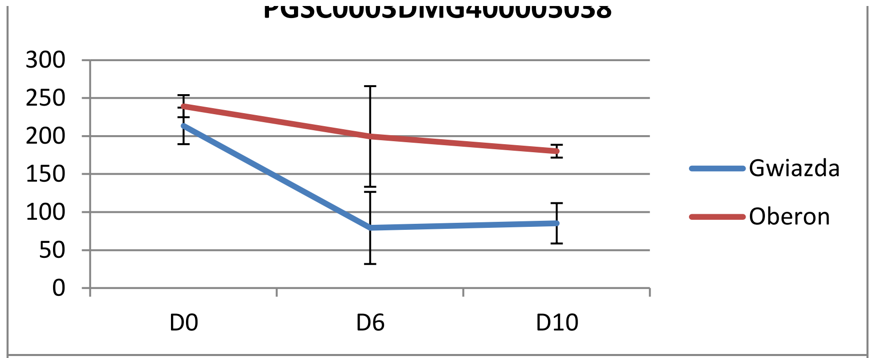

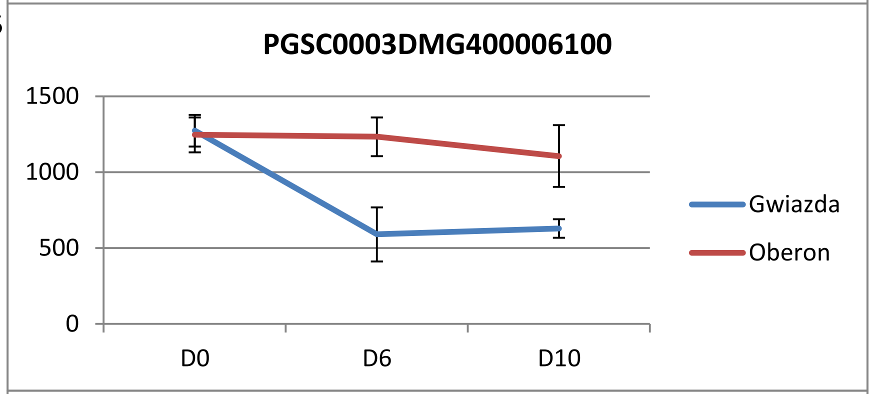

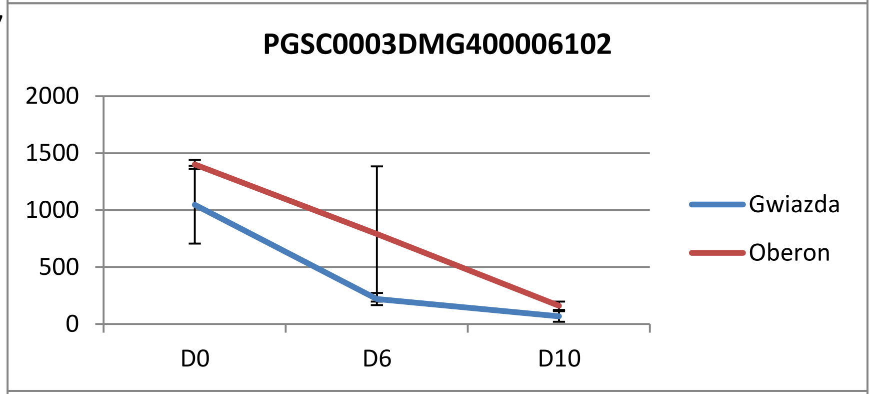

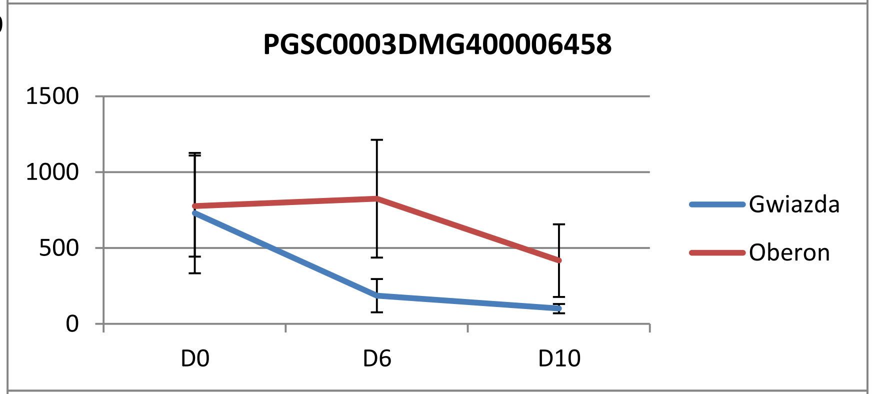

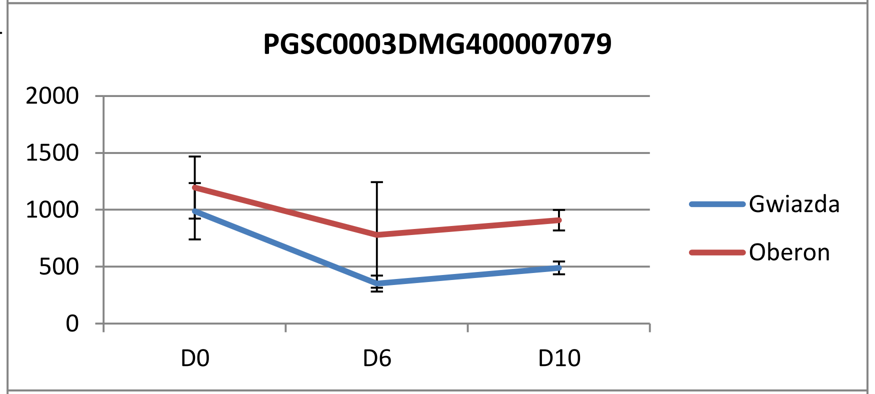

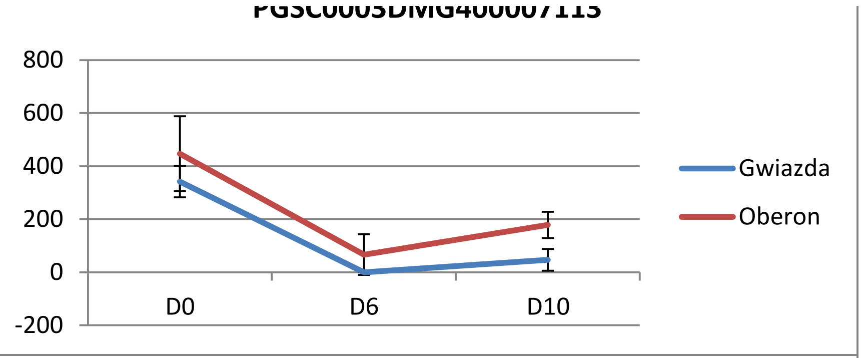

**PGSC0003I**	703,33	38,67	51,33	878	161,33	182	100,11	40,07	23,09	303,11	46,36	87,68
**PGSC0003I**	494,67	120	82	558	270,67	246	106,01	2	34,12	122,05	119,1	62,23
**PGSC0003I**	264,67	32,67	25,33	388	132,67	72	138,02	25,79	12,22	53,03	30,62	8,49
**PGSC0003I**	1106,67	530,67	404,67	1347,33	1898,33	1086	89,27	89,32	81,05	252,11	731,87	2,83
**PGSC0003I**	3052,67	438	164,67	4667,33	1912	821	536,77	113,42	19,22	1690,01	477,06	227,69

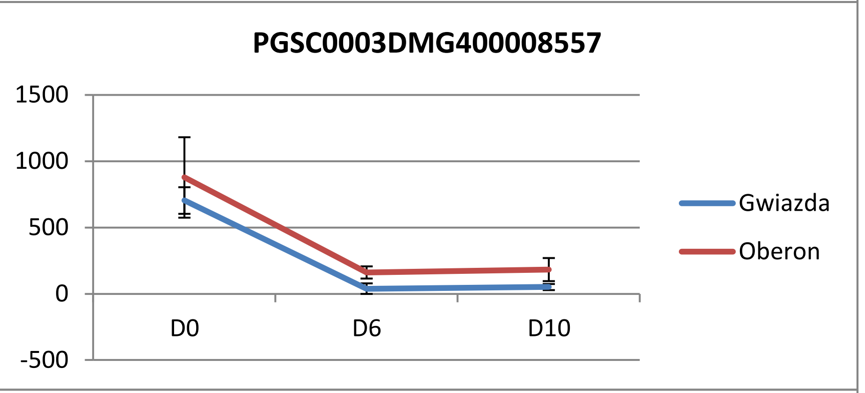

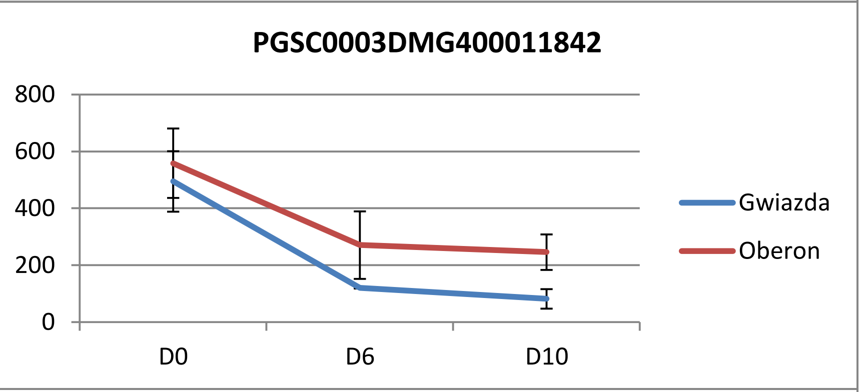

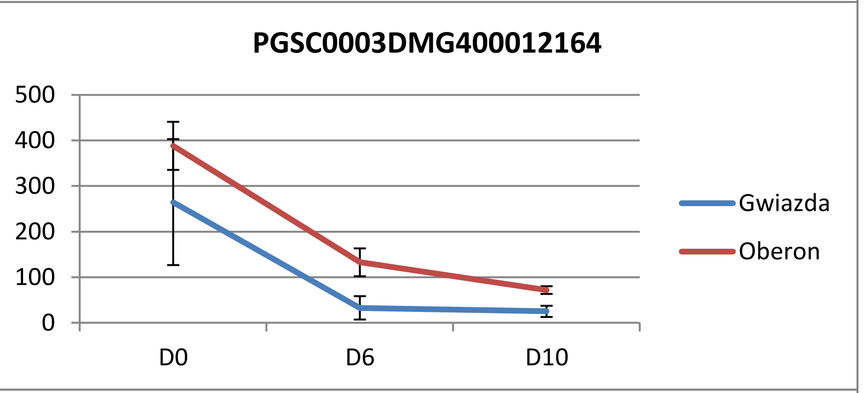

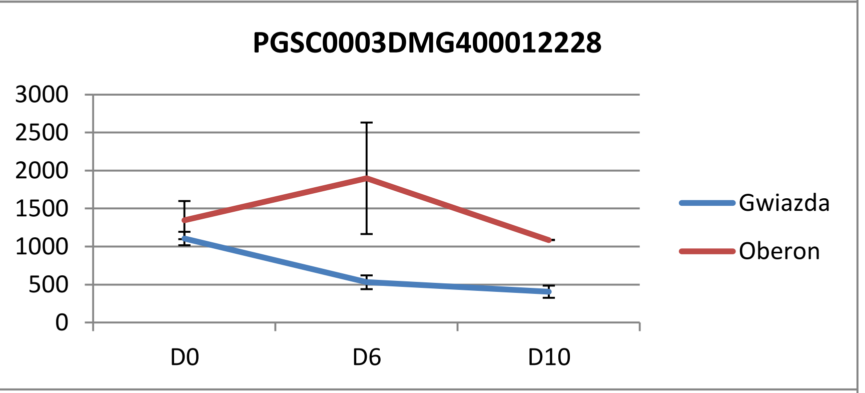

**PGSC0003I**	1071,33	108,67	23,33	1211,33	747,33	137	760,62	50,85	24,03	244,01	653,76	100,41
**PGSC0003I**	10578	1413,33	298	13800,7	5364	2037	3031,66	443,33	66,09	2986,94	3359,53	278,6
**PGSC0003I**	395,33	78,67	81,33	439,33	264	168	124,09	17,93	8,33	122,66	79,77	8,49
**PGSC0003I**	1732	1014	349,33	1610,67	532,67	174	147,63	309	33,61	54,78	304,22	14,14
**PGSC0003I**	40451,3	15839,3	4060	37915,3	47421,3	15041	6668,71	1953,1	195,47	2792,33	5787,03	3132,48

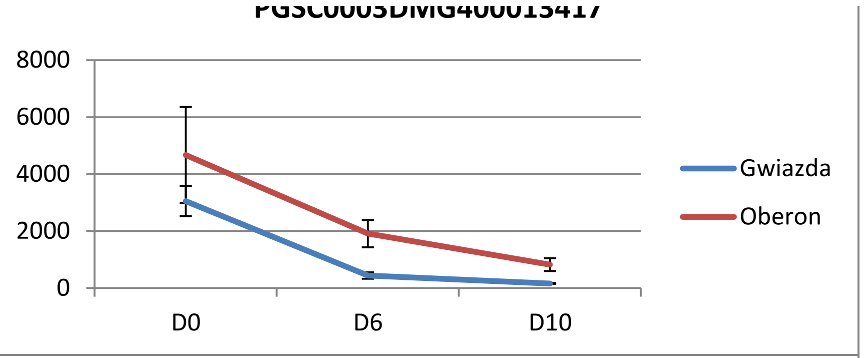

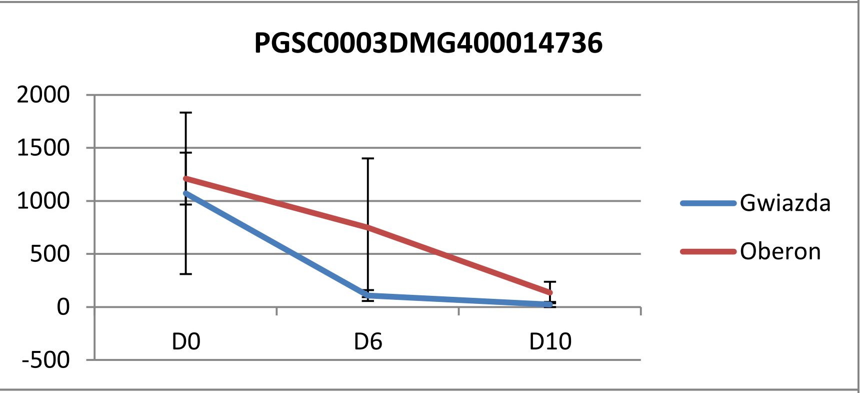

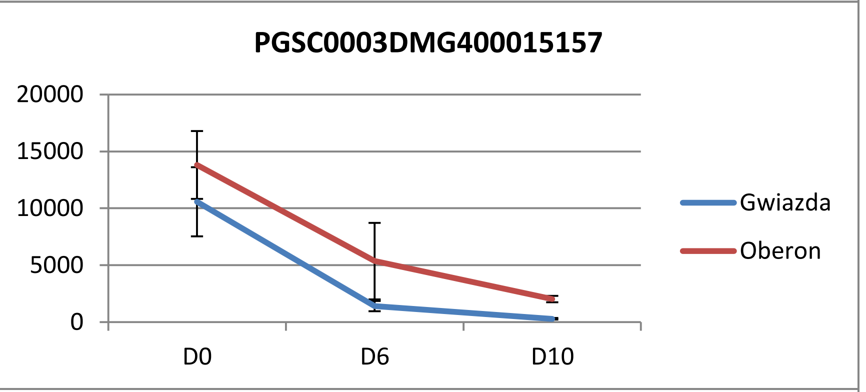

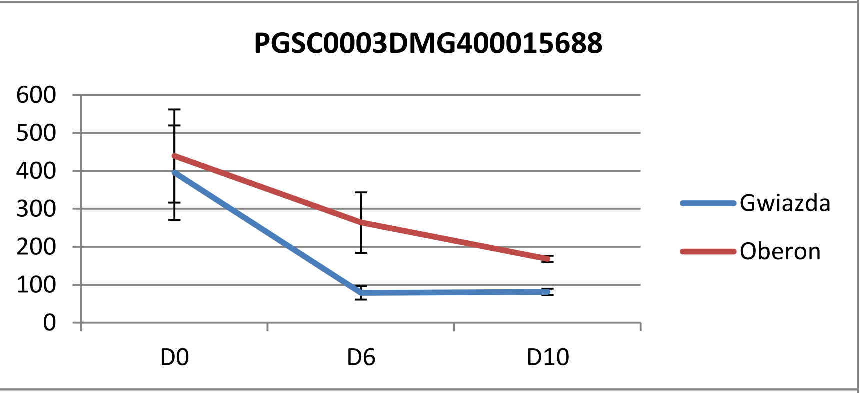

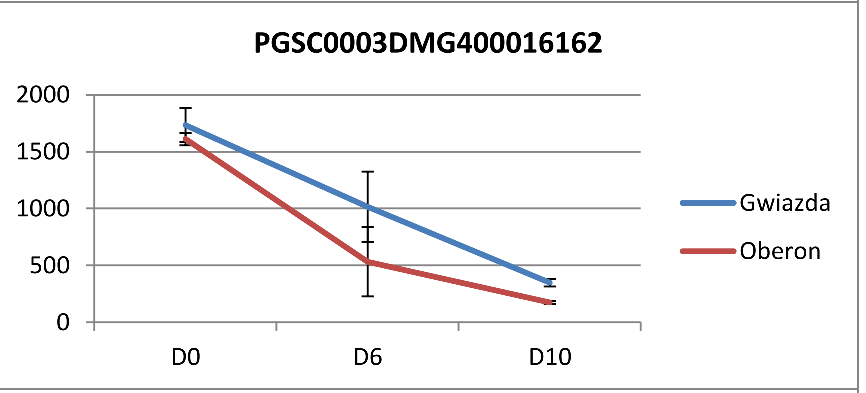

**PGSC0003I**	316,67	10	3,33	344,67	499,33	75	222,39	14	3,06	127,91	412,08	57,98
**PGSC0003I**	2710	1626,67	1584	3016	3442	2875	302,67	302,11	197,8	345,2	596,71	338
**PGSC0003I**	4072,67	1912,67	1988,7	3954	3384,67	3376	340,71	387,53	277,38	282,89	699,61	98,99
**PGSC0003I**	1574	285,33	480,67	1648,67	1152	2091	785,59	178,99	75,08	686,02	599,04	1288,35
**PGSC0003I**	740	244	288	835,33	846,67	507	399,39	76,6	73,02	178,91	619	148,49

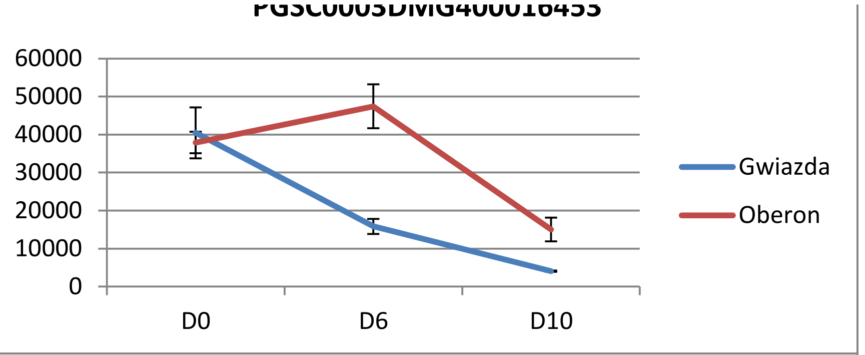

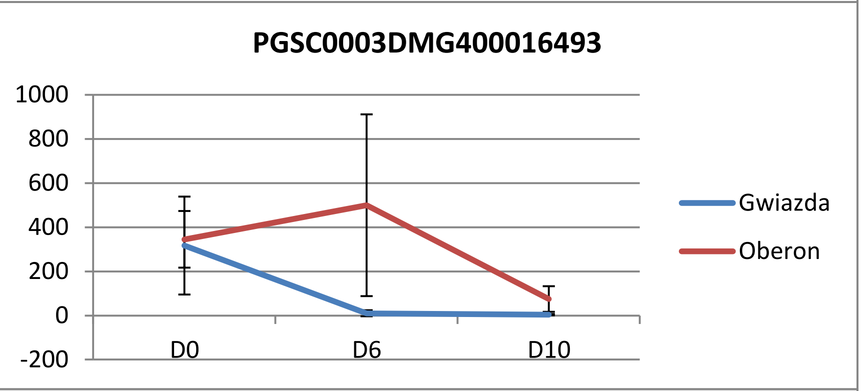

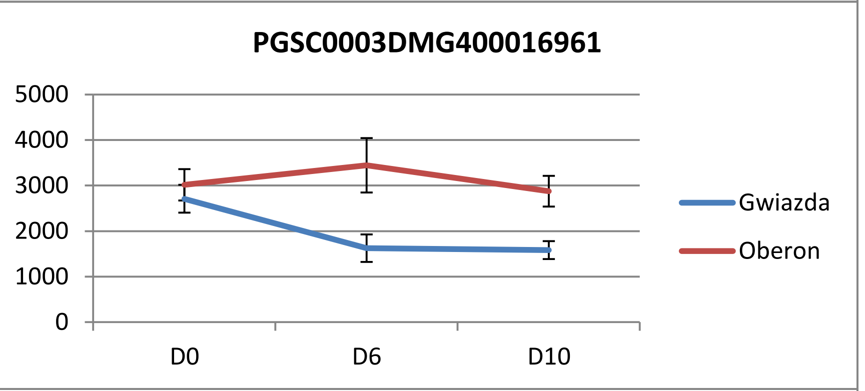

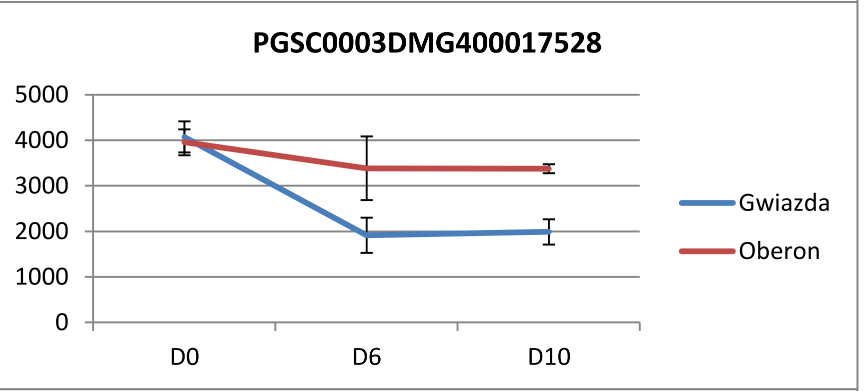

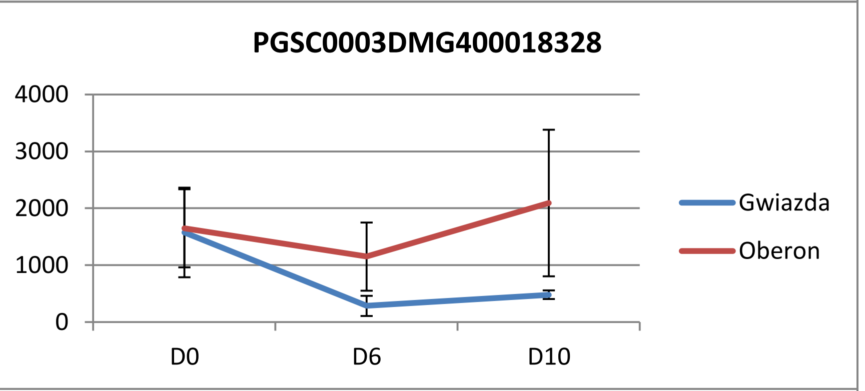

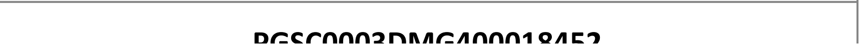

**PGSC0003I**	314,67	28	34,67	436,67	114	112	114,09	48,5	6,11	90,72	29,05	31,11
**PGSC0003I**	1705,33	270,67	68	1471,33	988,67	255	696,92	63,13	48,12	61	643,66	52,33
**PGSC0003I**	146,67	22,67	17,33	109,33	105,33	113	45	20,53	30,02	52,2	40,41	38,18
**PGSC0003I**	256,67	38	6	306	134	40	138,16	20,88	5,29	82,78	99,02	0
**PGSC0003I**	4218,67	1955,33	1872,7	3266	3644	2820	1075,33	335,16	372,29	488,77	1022,29	229,1

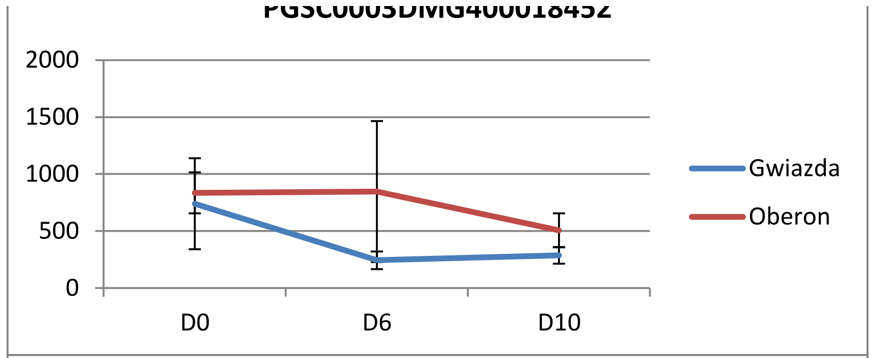

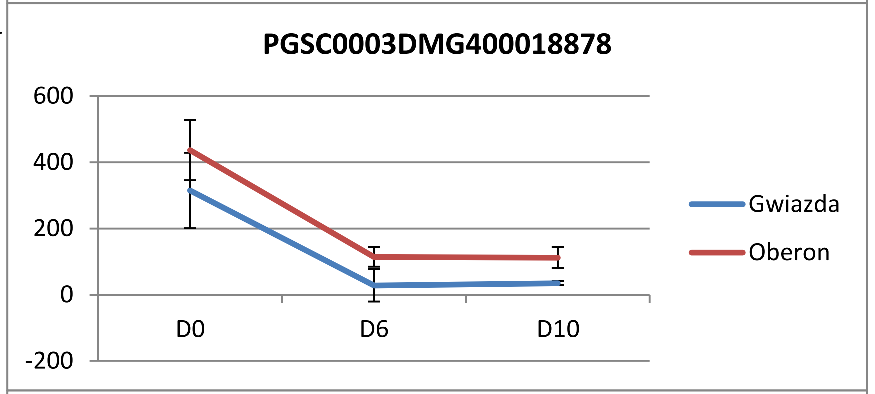

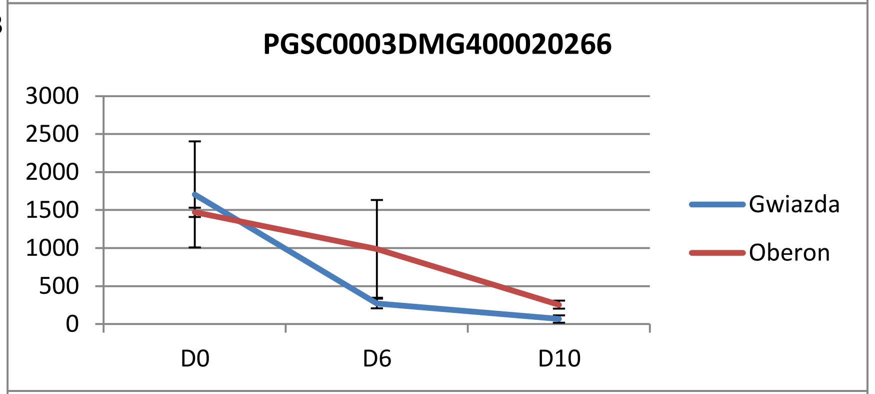

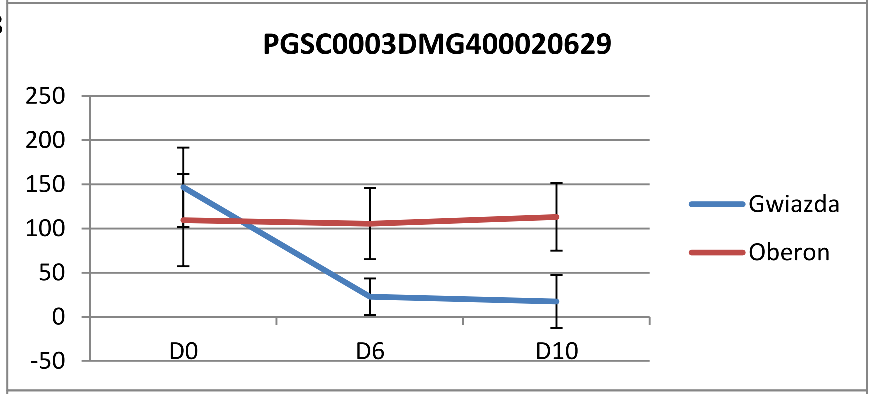

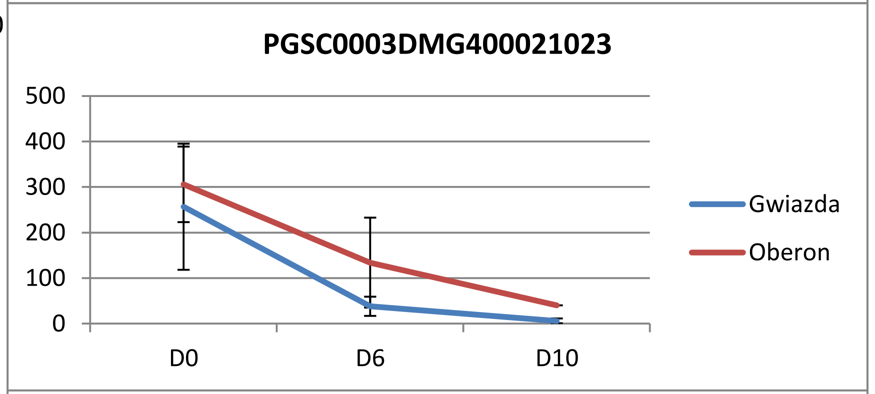

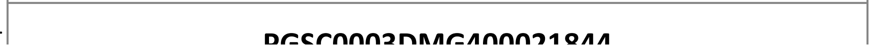

**PGSC0003I**	608	314	238	556,67	832,67	449	112,41	54,99	39,4	37,22	78,62	69,3
**PGSC0003I**	2088,67	618,67	208,67	2398	1419,33	621	787,33	77,6	18,15	542,21	551,24	346,48
**PGSC0003I**	2474,67	820	467,33	3124,67	1548	1265	153,13	184,81	115,49	959,18	245,5	485,08
**PGSC0003I**	754,67	168	254,67	847	484,33	553	133,36	37,47	95,04	163,5	249,72	25,46
**PGSC0003I**	336,67	115,33	93,33	425,33	347,33	225	95,02	12,86	3,06	69,41	89,72	4,24

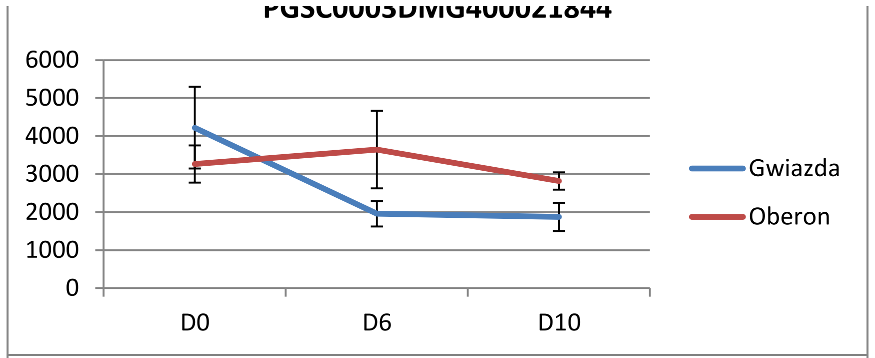

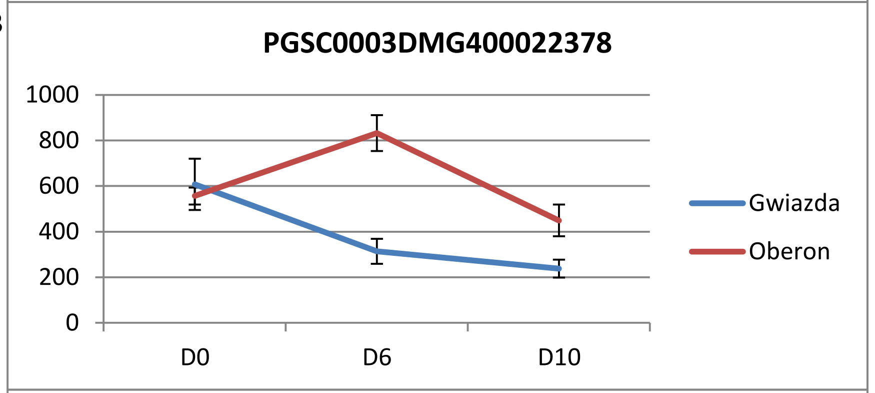

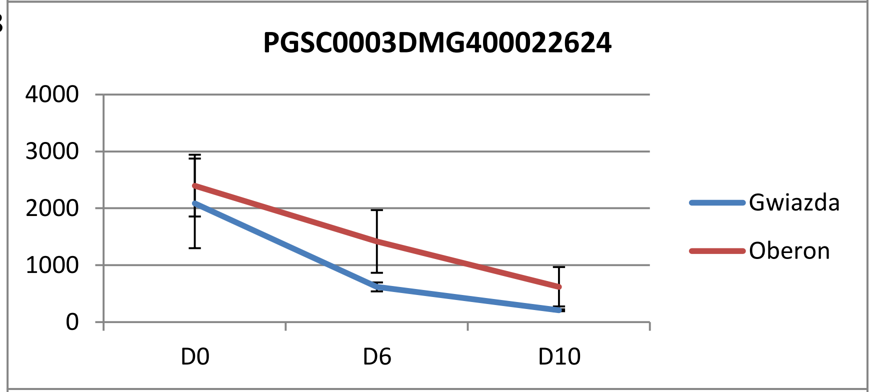

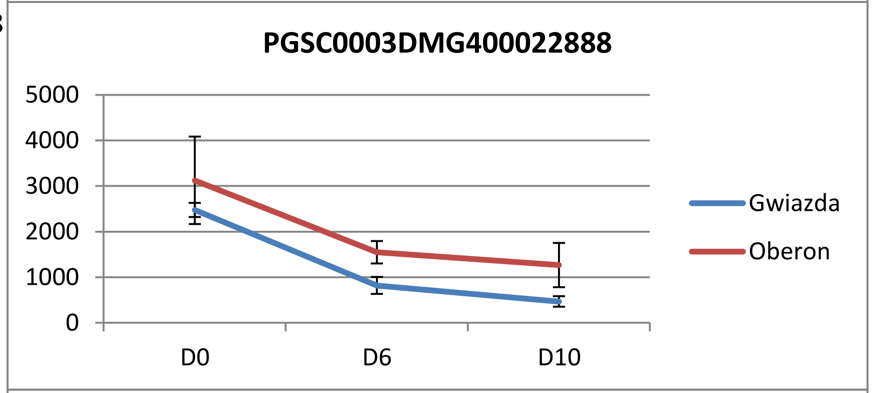

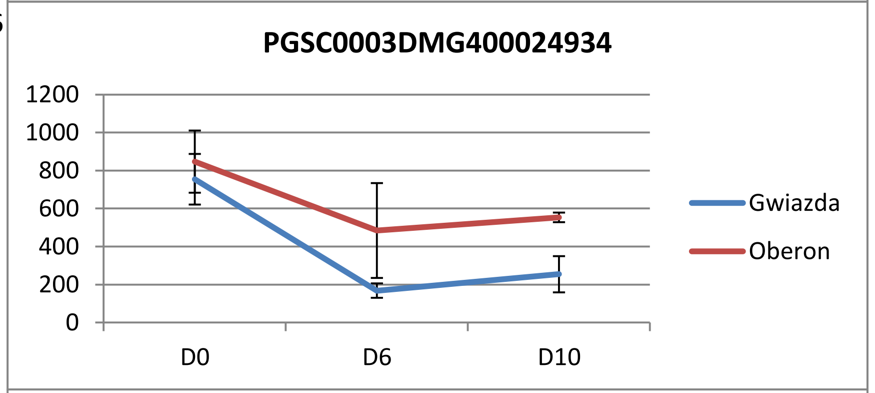

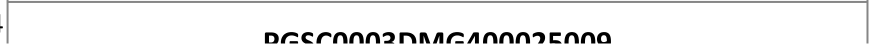

**PGSC0003I**	14122,7	5350,67	5410,7	9586	10309,3	12310	2014,1	1589,25	760,08	1907,22	1633,9	285,67
**PGSC0003I**	192	21,33	4	184,67	154	29	74,48	24,44	6,93	42,44	28	1,41
**PGSC0003I**	376,67	108	77,33	488	34,67	154	35,91	36,06	23,86	175,01	30,29	19,8
**PGSC0003I**	224,67	68	78	262	325,33	253	90,16	17,78	2	149,81	122,48	117,38
**PGSC0003I**	2824,67	422,67	139,33	3402,67	1030,67	323	931,63	155,39	30,62	866,42	455,11	176,78

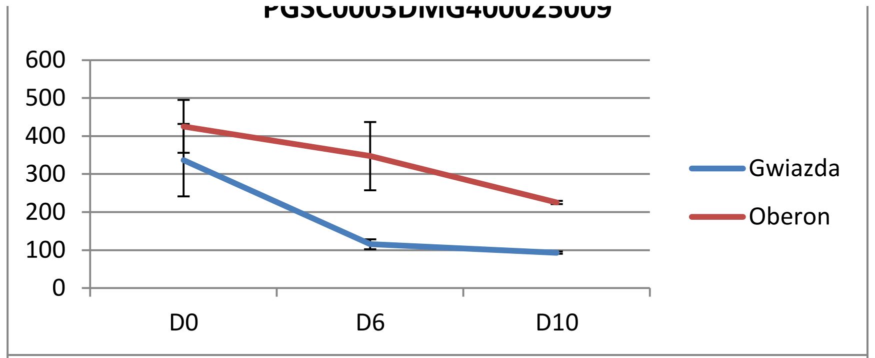

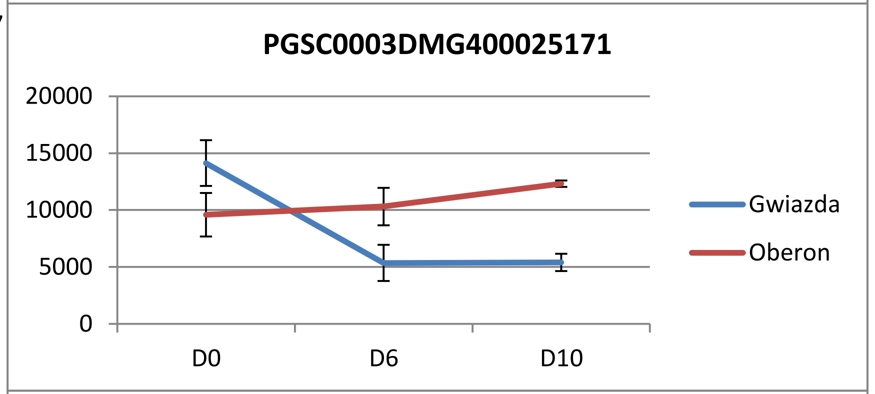

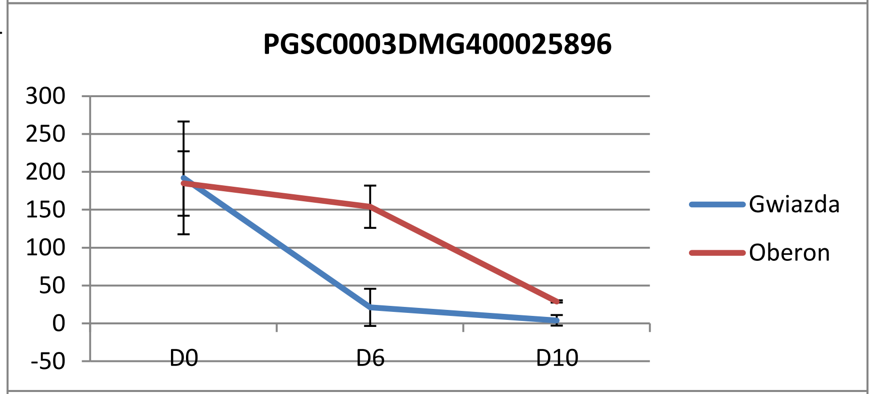

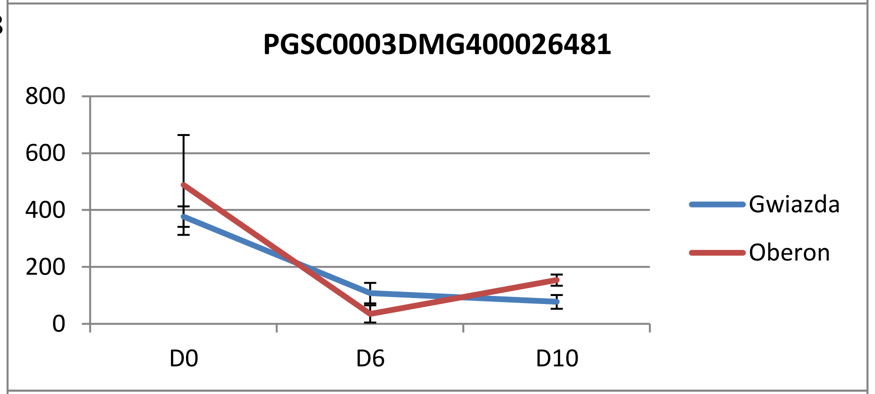

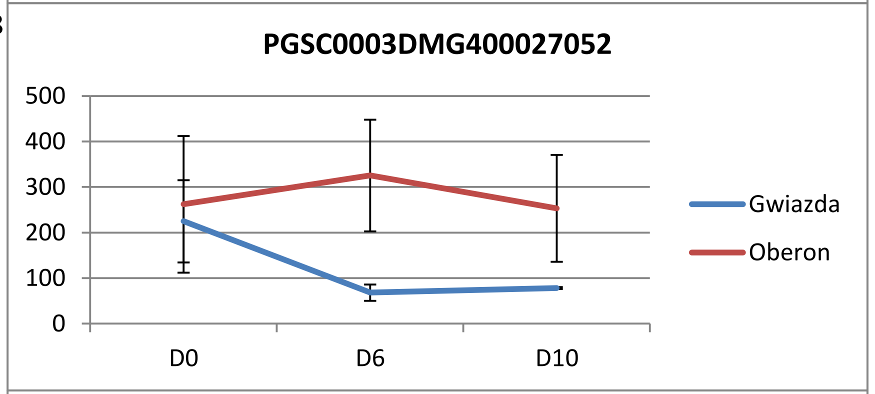

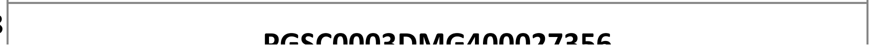

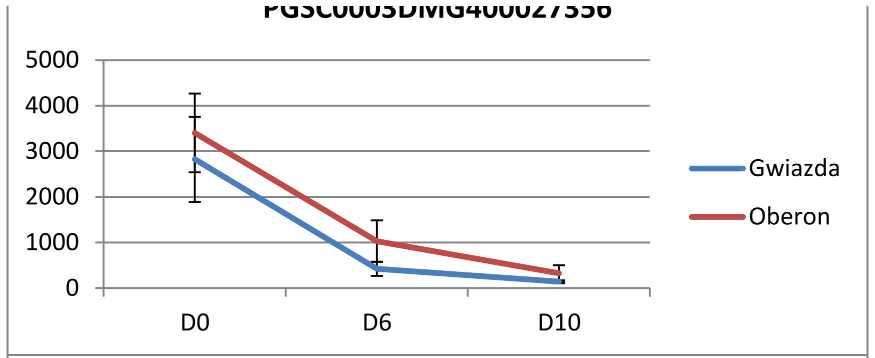

**PGSC0003I**	750	85,33	0	435,33	340	33	229,5	86,01	0	204,36	180,08	46,67
**PGSC0003I**	2913	773,33	697,33	3227	2113,33	1572	816,11	124,78	167,44	663,22	1214,37	647,71
**PGSC0003I**	244	116,67	107,33	143,33	5,33	29	48,54	30,75	48,01	42,72	9,24	9,9
**PGSC0003I**	1066	572,67	688,67	1217,33	1062,67	1141	107,13	266,55	73,38	238,11	301,96	400,22
**PGSC0003I**	266,67	12	1,33	127,33	364	37	132,79	12	2,31	58,05	154,26	26,87

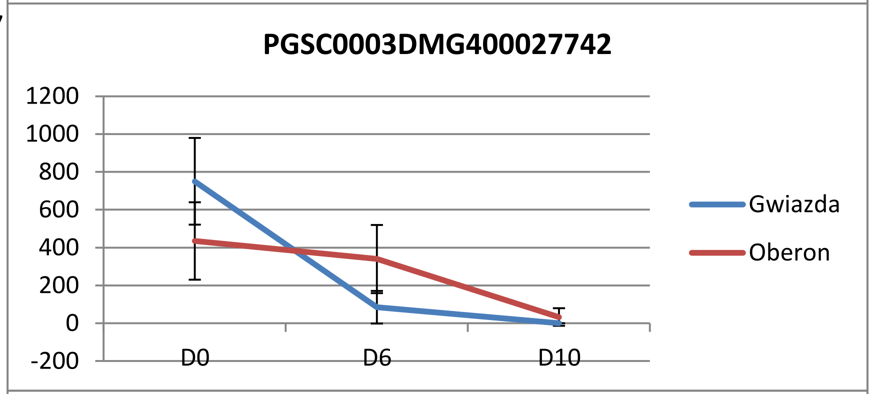

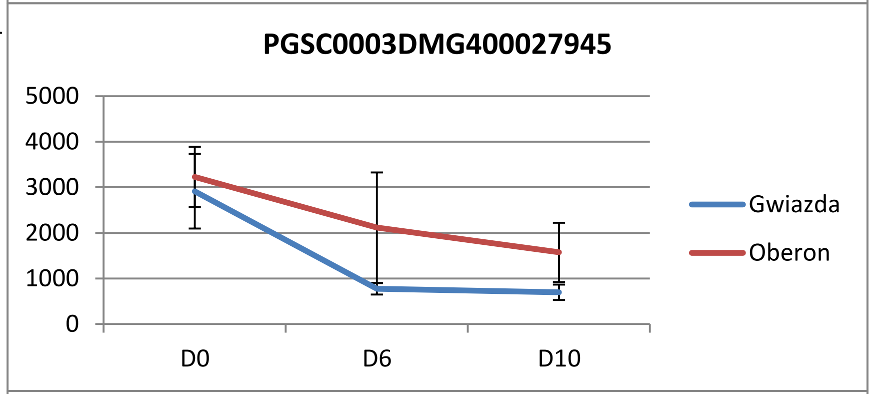

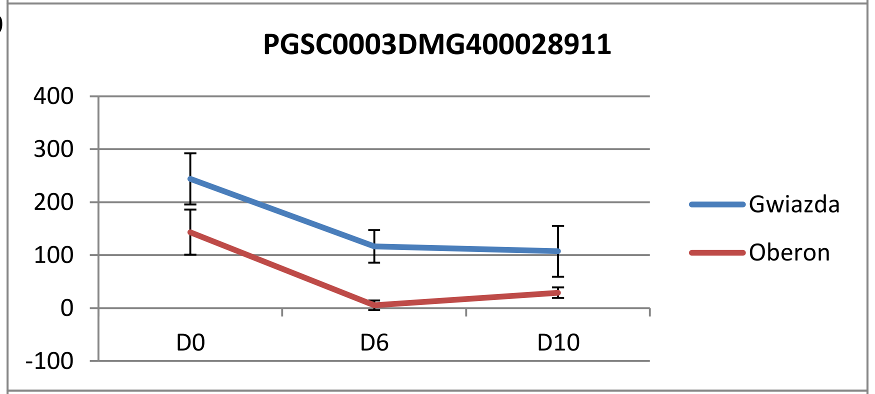

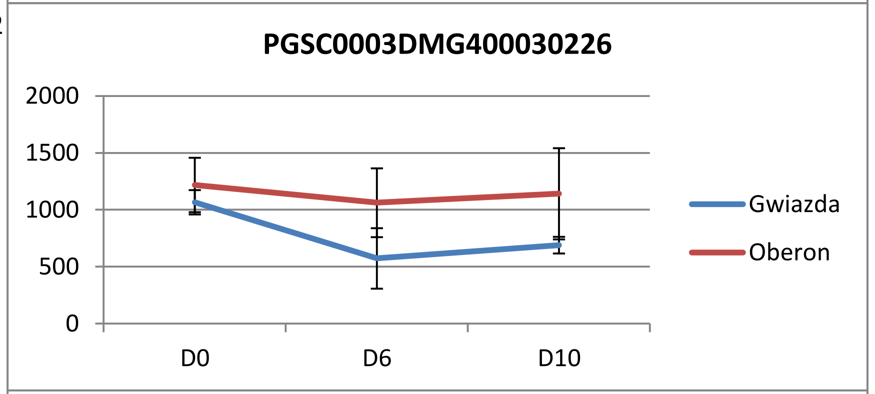

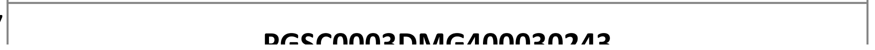

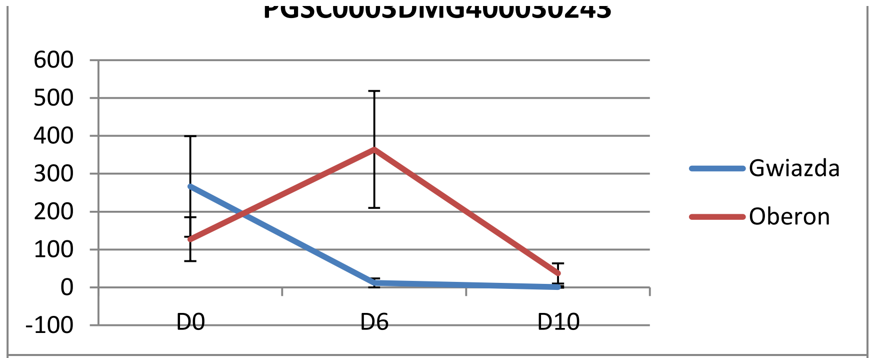

**PGSC0003I**	1052,67	260	110	848,67	663,33	214	391,36	19,08	7,21	255,47	146,11	70,71
**PGSC0003I**	256,67	18	28,67	240	304,67	81	114,15	21,63	11,02	107,46	302,89	46,67
**PGSC0003I**	118	45,33	39,33	140	158	116	44,23	15,01	25,72	59,23	112,48	14,14
**PGSC0003I**	395,33	158,67	110,67	526,67	474	432	107,06	49,17	67,69	195,72	324,07	48,08
**PGSC0003I**	1518	744,67	574	1699,33	2122,67	972	500,57	113,44	47,62	312,03	958,02	84,85

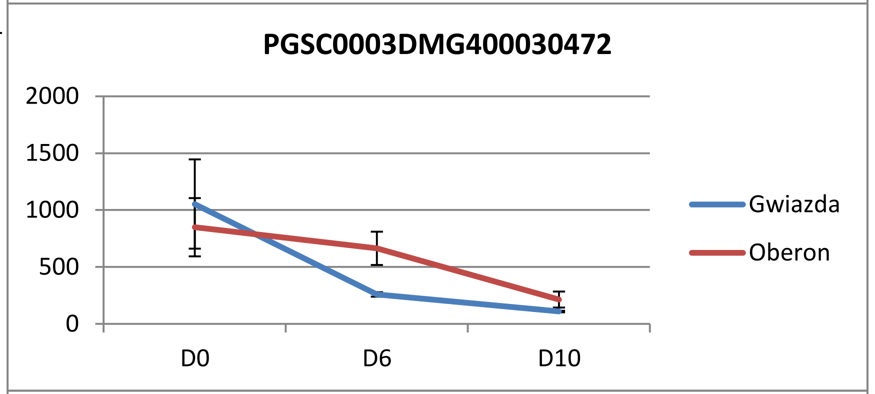

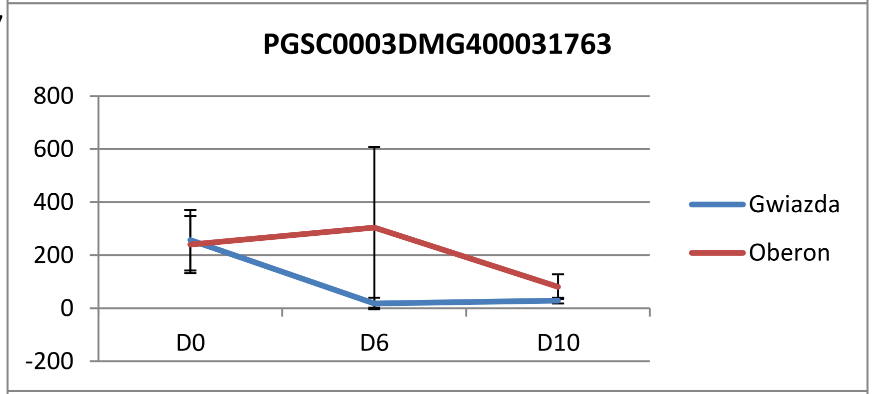

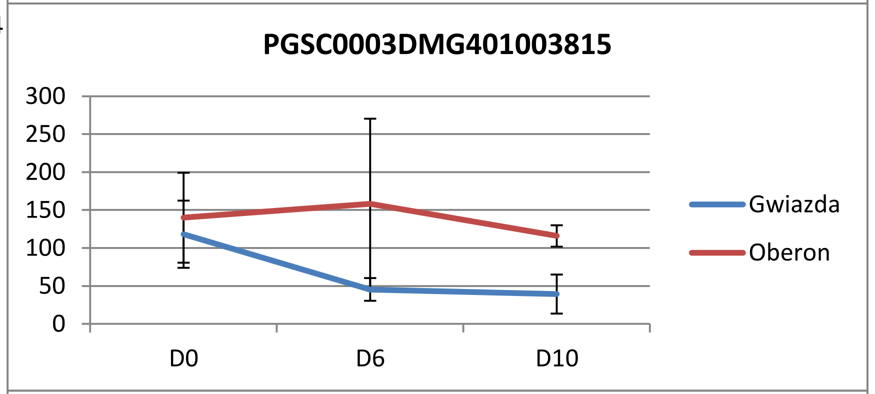

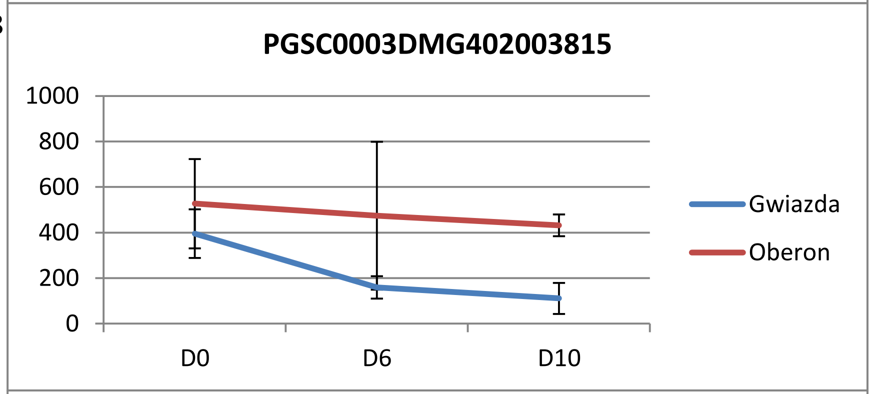

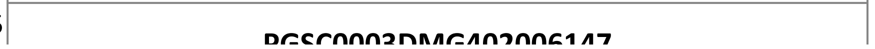

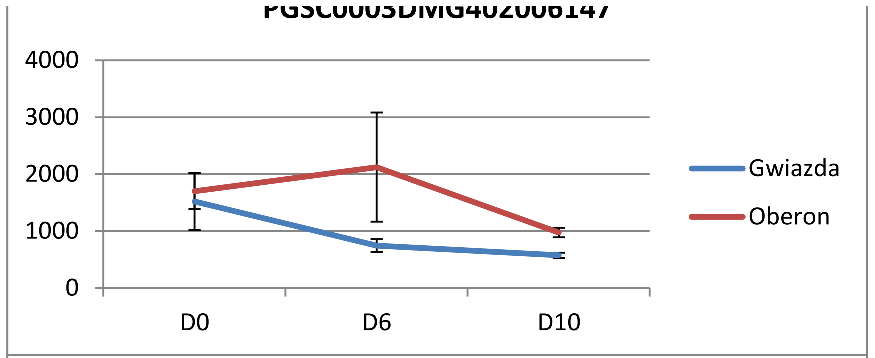

**PGSC0003I**	265	0	0	699,67	269,67	299,5	260,62	0	0	1211,86	467,08	62,93

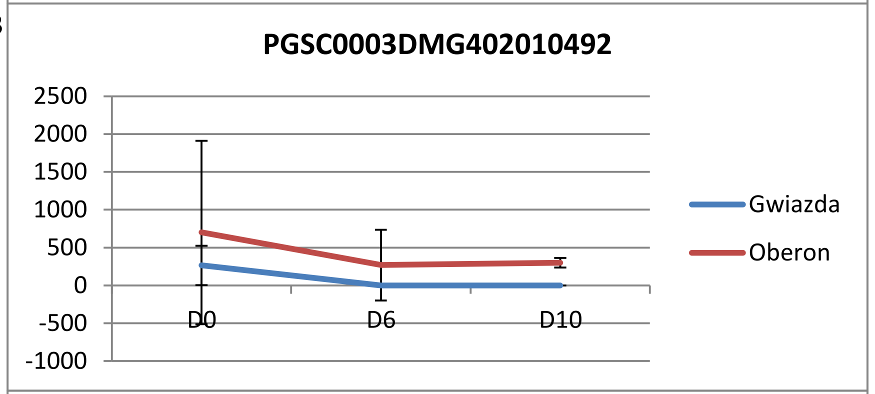

**PGSC0003I**	1418	347,33	302	1192,67	1316,67	769	733,23	90,18	98,97	380,23	992,22	349,31

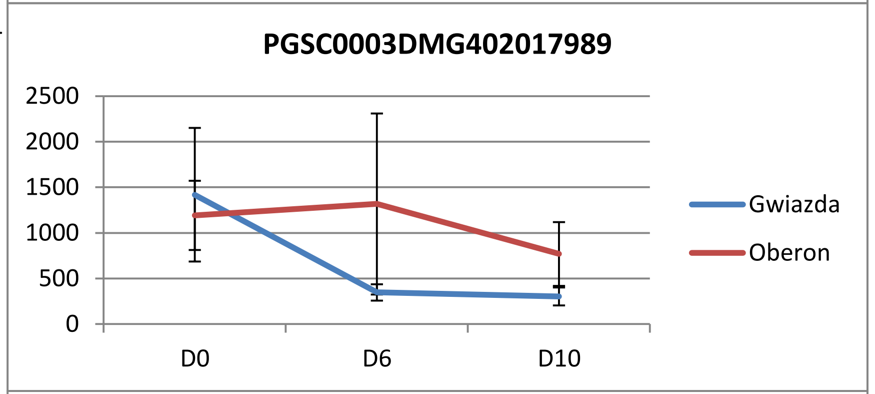

**PGSC0003I**	532,67	106,67	64,67	586	567,33	231	91,24	37,17	17,93	80,07	247,42	131,52

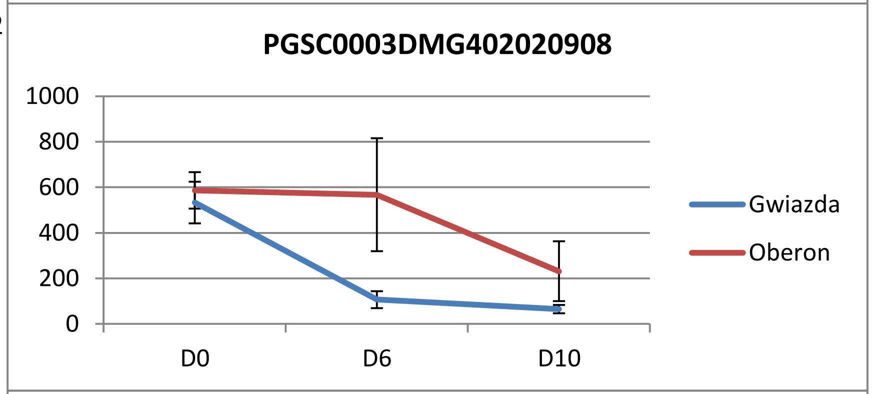

**PGSC0003I**	357,33	59,33	8,67	478,67	244	43	103,85	33,61	11,72	83,91	130,22	18,38

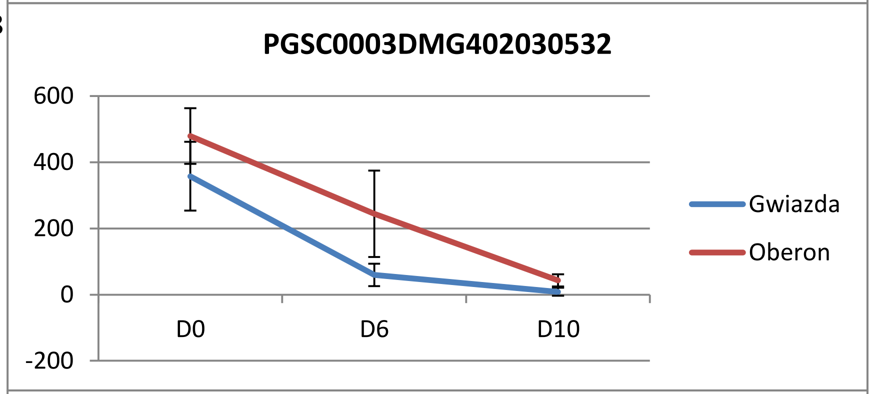

**PGSC0003I**	3098	1492	1548,7	2805,33	2787,33	3305	350,15	262,09	211,23	68,86	765,83	12,73

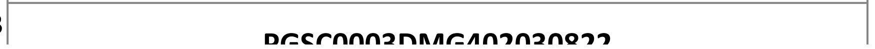

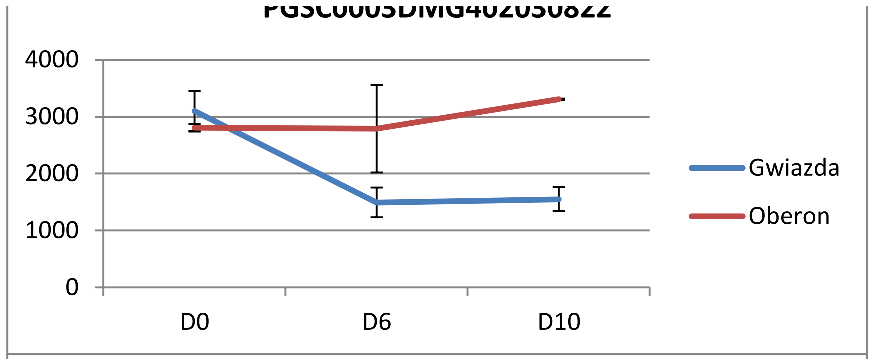

**PGSC0003I**	942,67	75,33	169,33	1102	848	387	227,21	130,48	44,38	225,49	129,57	35,36
**PGSC0003I**	626,67	155,33	202,67	386,67	519,33	722	342,48	48,22	67,45	103,47	381,75	16,97
**PGSC0003I**	660	108,67	49,33	708,67	544,33	114	341,18	30,62	12,22	136,08	360,78	53,74

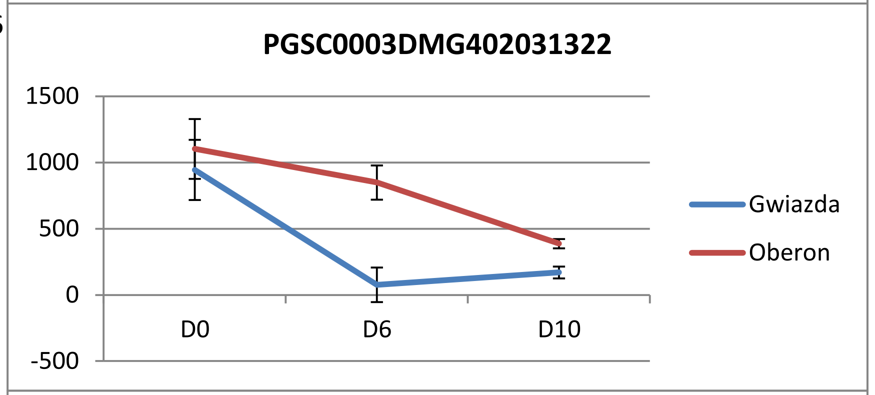

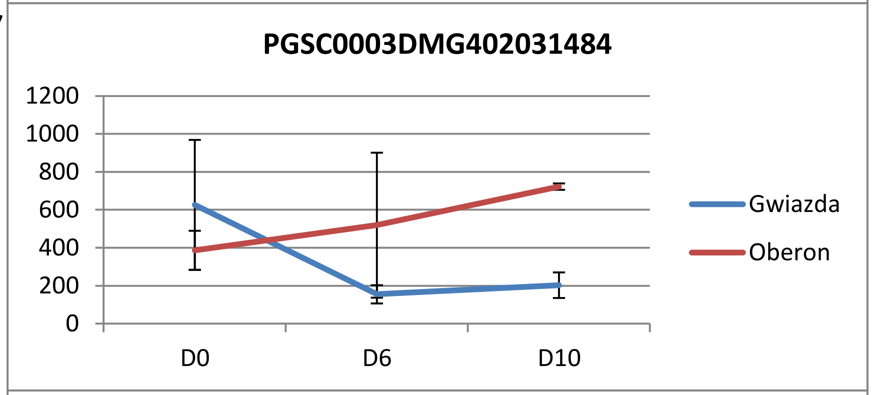

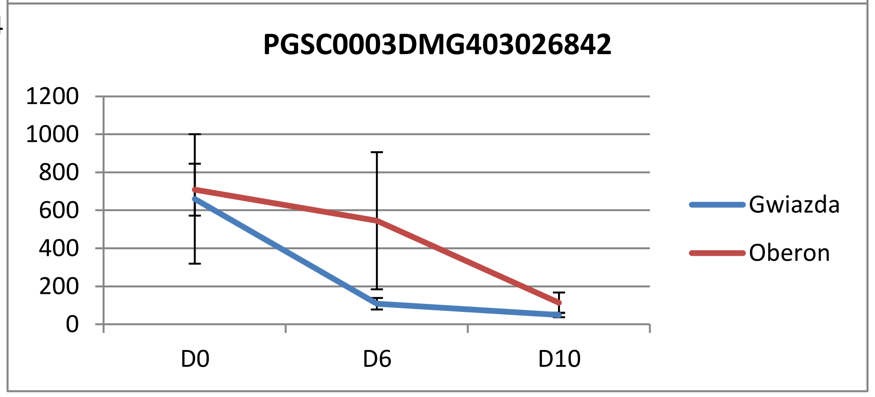

Supplement: Supplementary file 2 — Table S1 Comparison of the normalized number of reads for transcripts derived from genes identified in the first round of selection (594 genes) during the time course of the drought experiment. [file PBI-16-603-s002.pdf]
